# Gold(i)-catalyzed cycloisomerization of vinylidenecyclopropane-enes *via* carbene or non-carbene processes†
†Electronic supplementary information (ESI) available: Experimental procedures, characterization data of new compounds, and CCDC 996502, 980558, 997410 and 996499. See DOI: 10.1039/c5sc01806d


**DOI:** 10.1039/c5sc01806d

**Published:** 2015-06-24

**Authors:** De-Yao Li, Yin Wei, Ilan Marek, Xiang-Ying Tang, Min Shi

**Affiliations:** a State Key Laboratory of Organometallic Chemistry , Shanghai Institute of Organic Chemistry , Chinese Academy of Sciences , 345 Lingling Road , Shanghai 200032 , P. R. China . Email: siocxiangying@mail.sioc.ac.cn ; Email: mshi@mail.sioc.ac.cn; b Schulich Faculty of Chemistry , Technion–Israel Institute of Technology , Technion City , Haifa 32000 , Israel . Email: chilanm@tx.technion.ac.il

## Abstract

Gold catalyzed cycloisomerization of aromatic ring tethered vinylidenecyclopropane-enes provides a divergent synthetic protocol for the construction of O-containing fused heterocycles through controllable carbene or non-carbene related processes. The carbene induced process features a new amphiphilic strategy to generate a gold carbene *via* a rearrangement of vinylidenecyclopropane. Whereas, the electronic effect of the *ortho*-substituents switches the reaction mode onto the non-carbene related process, from which five- or six-membered rings are selectively produced through allyl-migration.

## Abstract 2

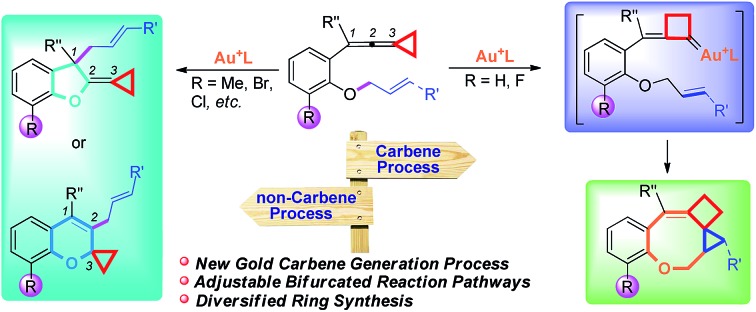
Gold catalyzed cycloisomerization of aromatic ring tethered vinylidenecyclopropane-enes provides a divergent synthetic protocol for the construction of O-containing fused heterocycles through controllable carbene or non-carbene related processes.

## 

Recently, the field of gold catalysis has witnessed significant developments.^1^ The exploration of new reaction modes in this arena has emerged as one of those at the forefront of current research. Due to the relativistic effect observed with gold and gold carbene complexes,^2^ these species present unique properties and reactivities. Impressive work has appeared recently,^3^ providing novel reaction pathways that could be summarized in Scheme 1: (1) Nuc–E strategies have been widely used to form α-functionalized gold carbenes;^4^ (2) retro-Buchner reaction can be easily used *in situ* to generate gold carbenes as a potentially common method;^5^ (3) the Au-σ-activated alkynes could also produce gold vinylidene species, which can act as gold carbenes through versatile reaction pathways.^6^ However, to the best of our knowledge, there are barely any reports on one precursor being able to rapidly generate molecular complexity^7^*via* either carbene or non-carbene pathways in gold catalysis.^8^ On the basis of our ongoing investigation on metal-catalyzed transformations of vinylidenecyclopropanes (VDCPs),^9^ we envisaged that VDCPs could be excellent candidates for the exploration of new reaction modes in gold catalysis because of their multiple reaction sites. Previously, Toste and co-workers reported novel intramolecular cyclopropanation and allyl-transfer, both through gold carbene intermediates (Scheme 2).4q–s Interestingly, during our study on the gold catalyzed cycloisomerization of aromatic ring tethered VDCP-enes, we found that cyclopropanation and allyl transfer through controllable carbene/non-carbene related processes could be achieved, respectively, featuring a new gold carbene generation process. The VDCP acts as a nucleophile under activation by the gold(i) complex and then undergoes ring expansion as an electrophile due to its amphiphilic electronic nature to generate gold carbene species. Herein, we wish to report these intriguing new gold-catalyzed transformations, which afford an easy and efficient access to fused five-, six- and eight-membered ring systems (Scheme 2).

**Scheme 1:**
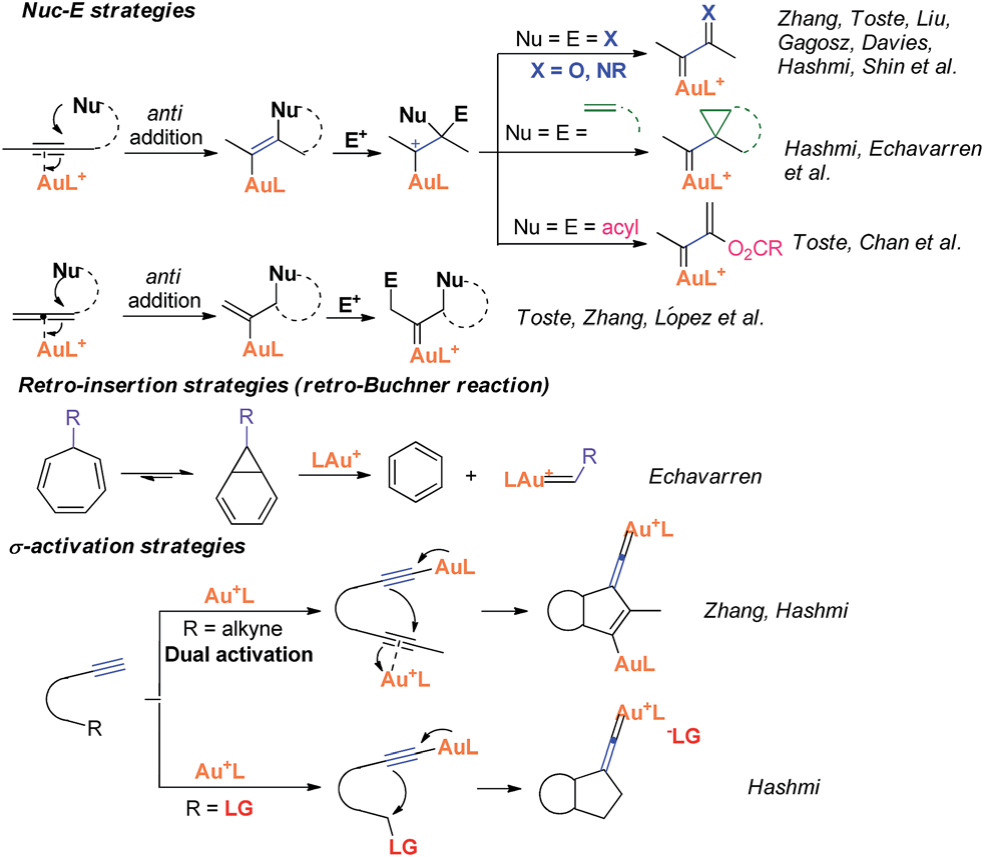
Previous strategies for the generation of gold carbenes.

**Scheme 2:**
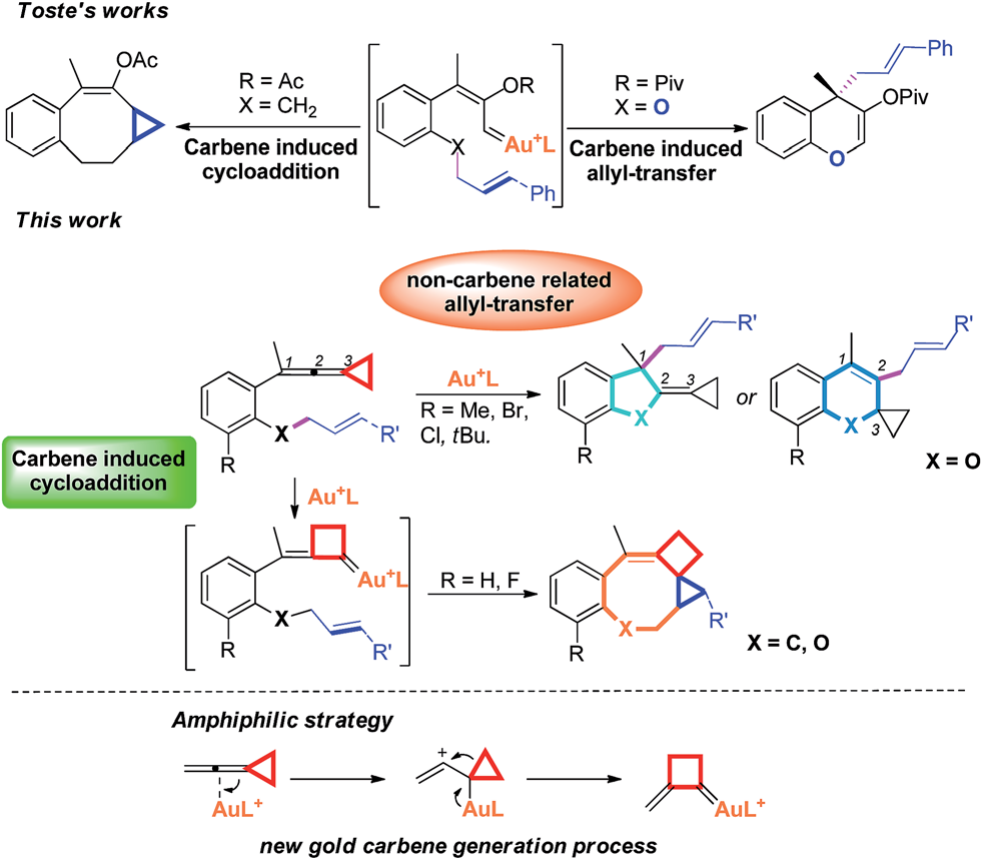
Modulable reaction processes of VDCP-enes catalyzed by gold(i) complexes along with a new gold carbene generation process.

Upon reaction condition screening (see ESI† for the details), we identified JohnPhosAuCl (10 mol%) as the best gold catalyst together with AgNTf_2_ (10 mol%) to carry out the reaction of **1a**. Product **2a**^10^ was formed in 93% yield at ambient temperature in DCE within 5 minutes (Scheme 3).

**Scheme 3:**
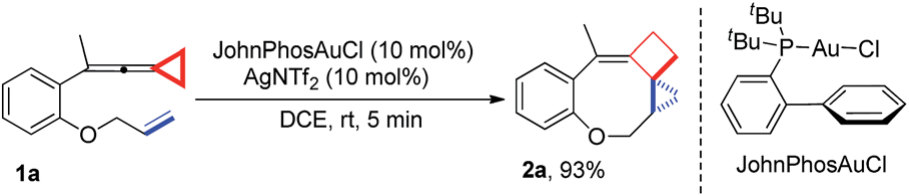
Optimal conditions for the synthesis of **2a**.

Having the optimal reaction conditions in hand, we next investigated the scope of the reaction with respect to various VDCP-enes. As shown in Table 1, these cyclization reactions proceeded smoothly, affording the desired products in moderate to excellent yields. Although this transformation proceeds in high yields for alkyl-substituted VDCPs (R^2^ = alkyl), yield is slightly lower for phenyl-substituted VDCP (R^2^ = Ph, **2e**). As for the substituents at the benzene ring, no obvious decrease of yields was observed when different halogen atoms such as F, Cl or Br were introduced. Substrate **1l** with electron-donating substituent MeO at the benzene ring afforded product **2l** in 47% yield.^11^ The naphthalene tethered substrate gave the expected product **2p** in good yield. Substrates **1q** and **1r** bearing a methyl substituent at the allyl group also afforded the corresponding products **2q** and **2r** in 90% and 82% yields, respectively. When a homoallylic group was introduced instead of an allyloxy group, the desired product **2s** could be given in 93% yield. It should be noted that only product **2l** was obtained in lower yield but this may come from the instability of the starting material. The DFT calculations indicated that the formation of **2** is an exothermic process (see ESI†).

**Table 1:** Reaction scope of VDCP-enes **1** to polycyclic products **2**^*a*^

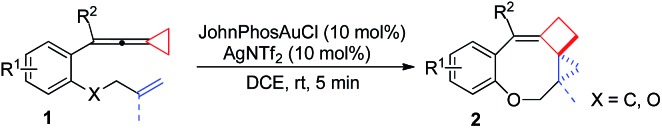
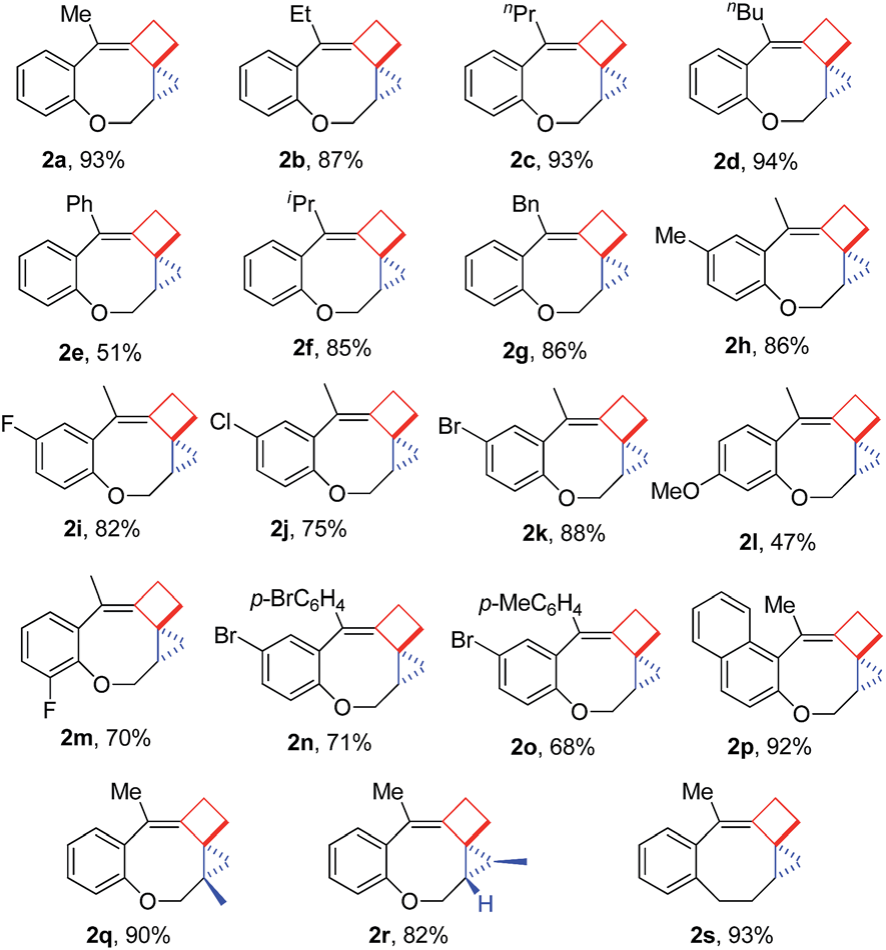

^*a*^The reaction free energy Δ*G*_rxn,298_ is –17.9 kcal mol^–1^ (obtained by DFT calculations, for details, see ESI). This result suggests that the formation of product **2** is exothermic.

We next synthesized substrate **3a** from 3,5-di-^*t*^butyl salicylaldehyde to examine the reaction outcome. However, we found that an allyl-transferred product **4a** derived from a non-carbene process was formed in 83% yield rather than the cyclopropanation product (Table 2).^12^ To have better insight on the substituent effects, we synthesized a library of substrates to clarify the scope. Consistent with the above results, all of these VDCP-enes having a substituent such as ^*t*^Bu, Me or Cl at 3-position gave the allyl-transferred products in good yields. Only when a fluorine atom was introduced at the 3-position, did the reaction proceed *via* a carbene process, giving **2m** in 70% yield as shown in Table 1. As for substrates **3k** and **3l** having two methyl groups or one phenyl group at the terminal position of the alkene, another type of allyl-transfer took place, affording **5k**^13^ and **5l** in 77% and 66% yields, respectively (Table 2). In the case of **3m**, two types of allyl-transfer took place at the same time to give **5m** and **4m** in good total yield as a product mixture (Table 2). **3k**'s regioisomer, *O*-(1,1-dimethylprop-2-enyl) derivative, is unavailable *via* the present synthetic method (see Scheme at page S8 in ESI† for the details).

**Table 2:** Gold-catalyzed cycloisomerization of VDCP-enes **3** to allyl-transferred products **4** and **5**

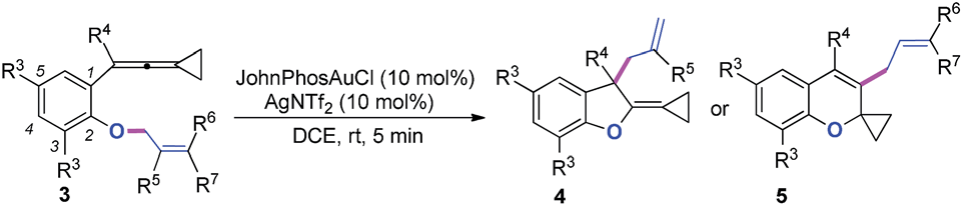
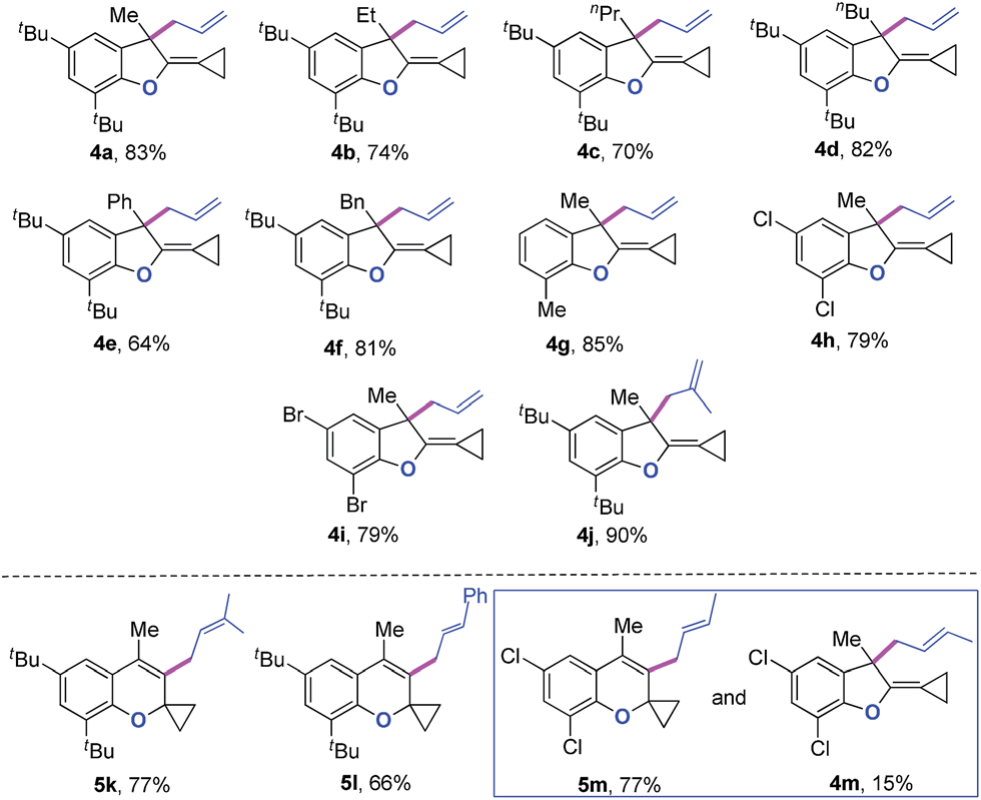

Two mechanistic experiments were conducted to clarify the allyl-transfer process of the allyloxyl group (Scheme 4). Treatment of [D]-**3a** under the standard conditions produced [D]-**4a** in 79% yield along with >99% D content. The deuterium labeling experiments suggesting that the allyl-transfer might proceed through a 1,3-shift of the allylic oxonium intermediate rather than a 3,3-shift1g,4s because no allylic inversion was observed. Furthermore, the double cross-over experiment using **3k** and **3n** as substrates under the standard conditions only produced the corresponding products **5k** and **5n**, respectively and no cross-over products were observed, indicating that the allyl-transfer proceeded *via* an intimate ion-pair.

**Scheme 4:**
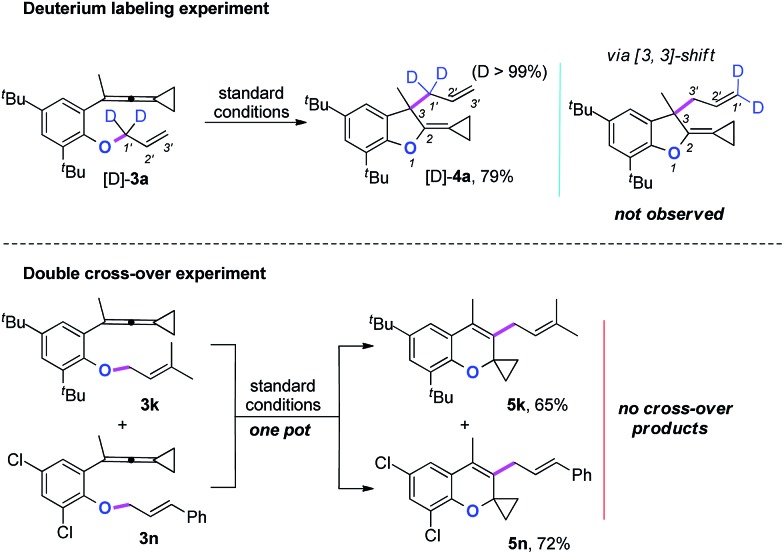
Mechanistic experiments.

Based on Toste's previous work and other groups' results, plausible mechanisms4s,^14^ for the above gold-catalyzed carbene and non-carbene related processes are outlined in Scheme 5. As for substrate **1a**, upon coordination of gold catalyst with VDCP intermediate **A** was formed, which initiates a ring expansion to give gold stabilized cation **B** and then carbene species **C**. Subsequent cyclopropanation produces polycyclic adduct **2a**. When the *ortho*-position is substituted by a Me, Cl, Br or ^*t*^Bu group, due to the increased nucleophilicity of the oxygen atom, **3g** attacks the middle carbon of the allenyl moiety in intermediate **D** to give the corresponding oxonium intermediate **E**, which undergoes S′_E_ allyl-transfer^15^ to afford product **4g***via* an intimate ion-pair **F**. In the case of **3k**, the oxygen atom exclusively attacks the terminal carbon at the allene moiety of VDCP in intermediate **G** to give the corresponding oxonium intermediate **H** presumably due to the steric bulkiness at the alkene site, which similarly undergoes the allyl-transfer to give **5k***via* an intimate ion-pair **I**. Overall, the gold species is finally quenched by the allylic cation to give the allyl-transferred product. The allyl-migration mode is different from previous work,4s,15b,c probably due to the steric hindrance of cyclopropane in the VDCP-type substrates.

**Scheme 5:**
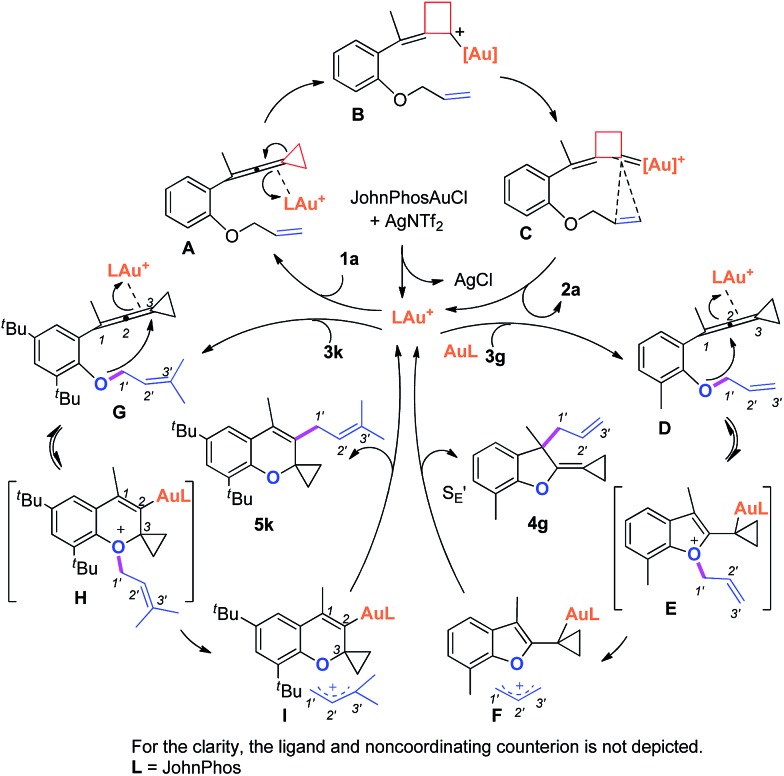
Proposed mechanisms.

The X-ray crystal structures of substrates **1n** and **3e** indicated that the distances between the oxygen and the central carbon of VDCP are very close (for details, see ESI†), suggesting that it is not the *ortho*-substituent that induces a proximity effect promoting the nucleophilic attack. We believe that the electronic effects of the *ortho*-substituents in the substrates influence the nucleophilicity of the oxygen atom, which is the origin for the different reactivities of substrates **2** and **3**. Calculation of Mulliken charge on the O atom influenced by X (a substituent or a substituted group at the *ortho*-position) was carried out on the basis of the B3LYP/6-31+G(d) level, inferring that different substituents led to different electron densities on the oxygen atom in the following order of ^*t*^Bu > Br > Cl > H > F (Table 3). In a similar manner, Mulliken charge on the O atom influenced by Y (a substituent at the *para*-position) was also calculated at the B3LYP/6-31+G(d) level, and the results show that the substituent at the *para*-position does not influence the electron density on the oxygen atom significantly (Table 3). These results suggest that the nucleophilicity of the oxygen is tunable by a judicious choice of substituents at the 3-position of the benzene ring, controlling therefore the carbene or non-carbene related processes at the very beginning. The thermodynamic stability of the subsequent oxonium intermediate **E** with different *ortho*-substituents was also investigated by DFT calculations (see ESI† for the details). The reactions were probably controlled by kinetic factors judging by the reaction conditions (5 minutes at room temperature). In order to understand why the substituents on the terminal alkene can affect the reaction outcome, we performed DFT calculations on key steps in the reaction of substrate **3k**. All calculations have been performed at the B3LYP/6-31+G(d)/SDD level with the Gaussian 09 program (see ESI† for the details). We investigated two reaction pathways starting from gold complex **3k-D** in Scheme 6. In path **a**, the oxygen atom in gold complex **3k-D** attacks the terminal carbon at the allene moiety to give an intermediate **3k-I***via* transition state **3k-TS1** with an energy barrier of 7.7 kcal mol^–1^. Subsequently, the intermediate **3k-I** passes through transition state **3k-TS2** with an energy barrier of 2.3 kcal mol^–1^, giving product complex **5k-P**. On the other hand, in path **b**, the oxygen atom in gold complex **3k-D** attacks the middle carbon at the allene moiety to give an intermediate **3k-F***via* transition state **3k-TS1′** with an energy barrier of 11.2 kcal mol^–1^. The energy of **3k-TS1′** is higher than that of **3k-TS1** by 3.5 kcal mol^–1^, presumably due to the steric repulsions among the substituents on the terminal alkene, the *t*-Bu substituent, and the ligand (for optimized structures of **3k-TS1′** and **3k-TS1**, see Fig. 1). Subsequently, the intermediate **3k-F** passes through transition state **3k-TS2′** with an energy barrier of 9.0 kcal mol^–1^, giving product complex **4k-P**. The calculation results show that all intermediates along path **a** are thermodynamically more stable than those along path **b**, meanwhile path **a** is also kinetically favourable, indicating that the product **5k** is the major product. The calculation results are in line with experimental results which obtained the product **5k** using **3k** as starting material. For comparison, we also investigated two pathways for the reaction of **3a**. The relative energies of all intermediates and transitional states along the reaction pathway for the reaction of **3a** are shown in Scheme 7. Similarly, the oxygen atom in gold complex **3a-D** attacks the terminal carbon at the allene moiety to give an intermediate **3a-I***via* transition state **3a-TS1** with an energy barrier of 9.3 kcal mol^–1^ in path **a′**. Subsequently, the intermediate **3a-I** passes through transition state **3a-TS2** with an energy barrier of 8.4 kcal mol^–1^, giving product complex **5a-P**. On the other hand, in path **b′**, the oxygen atom in gold complex **3a-D** attacks the middle carbon at the allene moiety to give an intermediate **3a-F***via* transition state **3a-TS1′** with an energy barrier of 8.9 kcal mol^–1^. The energy of **3a-TS1′** is slightly lower than that of **3a-TS1** by 0.9 kcal mol^–1^, probably due to the lack of steric repulsions among the substituents on terminal alkene, the *t*-Bu substituent, and the ligand. Subsequently, the intermediate **3a-F** passes through transition state **3a-TS2′** with an energy barrier of 6.3 kcal mol^–1^, giving product complex **4a-P**, which is lower than that of product complex **5a-P** by 1.8 kcal mol^–1^. The calculation results show that the path **b′** is also kinetically favourable, indicating that the product **4a** is the major product. The calculation results are in line with experimental results which obtained the product **4a** using **3a** as starting material.

**Table 3:** *Ortho*- and *para*-substituent effects

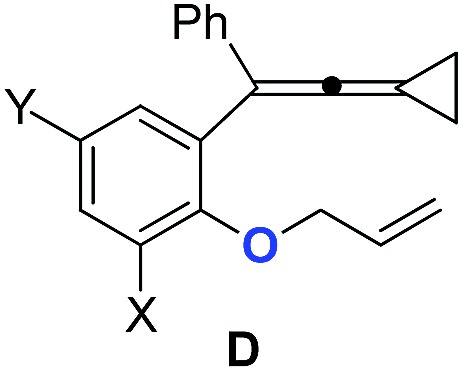		
X	Y	Mulliken charge on O atom^*a*^
F	H	–0.244
H	H	–0.301
Cl	H	–0.308
Br	H	–0.315
*t*Bu	H	–0.347
H	F	–0.242
H	Cl	–0.247
H	Br	–0.248

^*a*^Calculated at B3LYP/6-31+G(d) level.

**Scheme 6:**
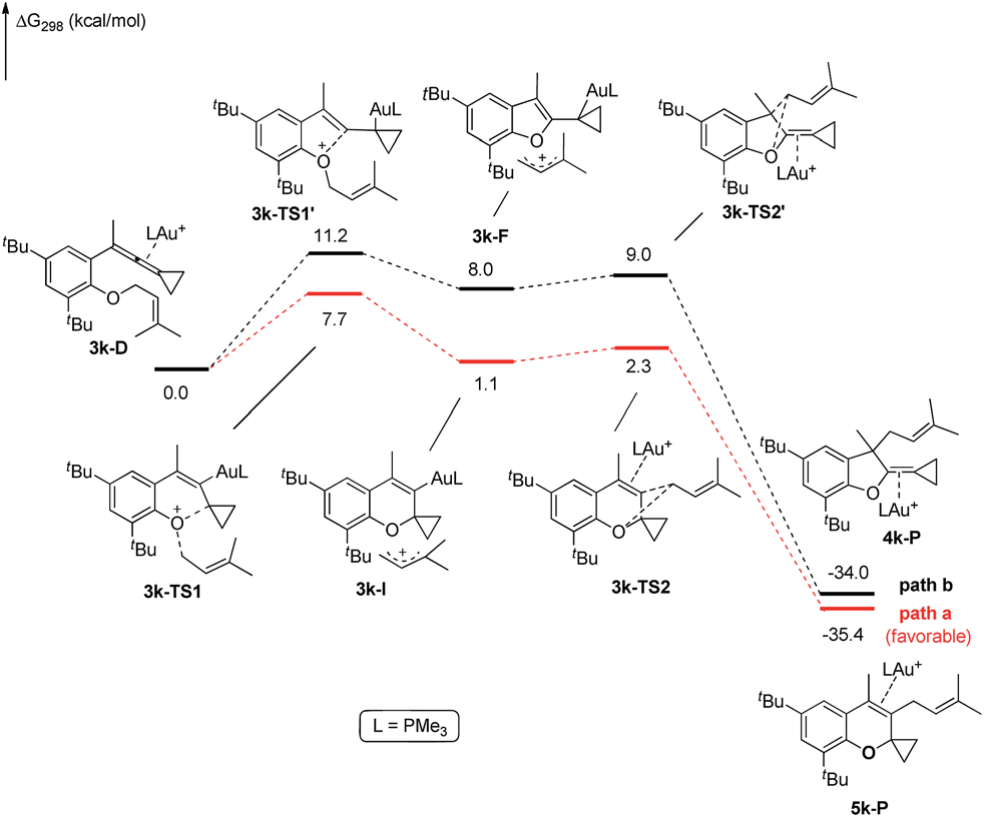
DFT studies on key reaction steps of the reaction of **3k**.

**Fig. 1:**
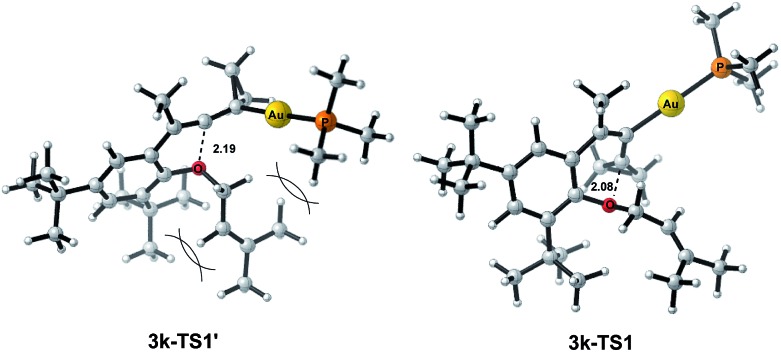
Optimized structures of **3k-TS1′** and **3k-TS1**.

**Scheme 7:**
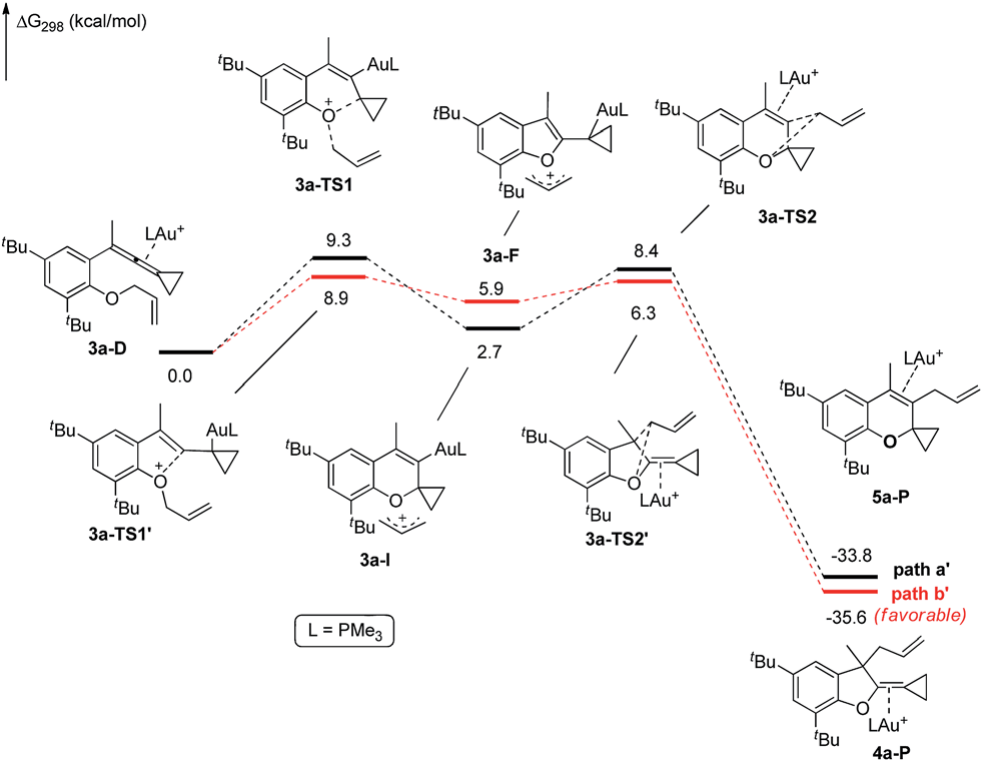
DFT studies on key reaction steps of the reaction of **3a**.

On the basis of the above results, we also attempted to develop an asymmetric variant for the two intramolecular cyclizations. The optimization of these asymmetric gold catalyses revealed that xyl-BINAP ligand coordinated gold complex gave the highest ee values in the carbene induced process, while DM-SegPhos ligand coordinated gold complex was the best one for the non-carbene induced process (see ESI† for the details). As shown in Scheme 8, the corresponding product **2** could be obtained in good yields along with 80–87% ee values whereas the allyl-transferred product **4a** was obtained in moderate yield along with 67% ee value. Using 20 mol% of AgSbF_6_ did not improve the ee value of **4a** (see ESI†).

**Scheme 8:**
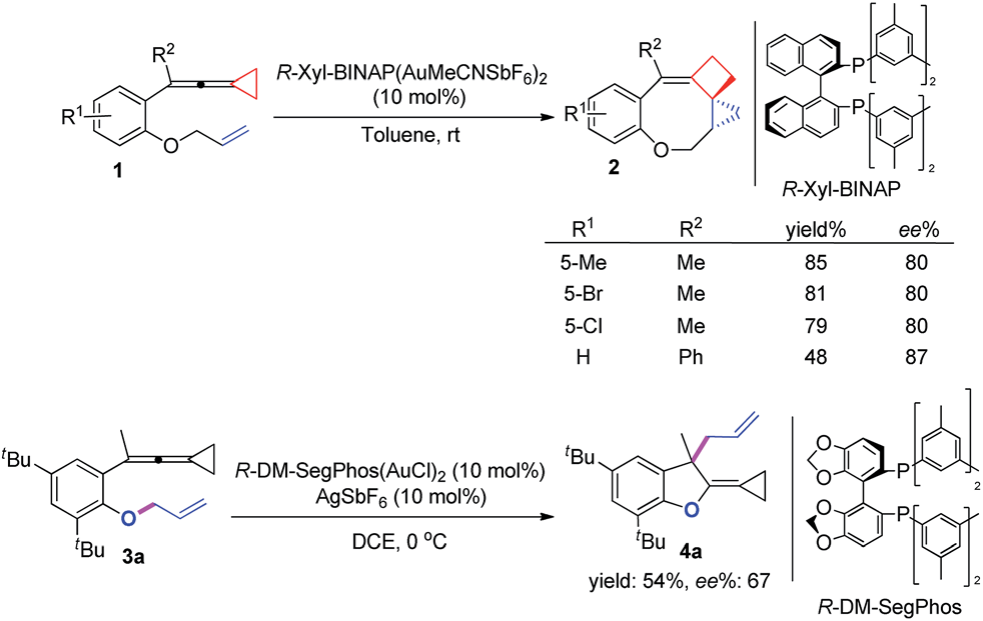
Asymmetric versions of the two processes.

The product **4a** could be easily transformed into benzofuran derivative **6** in 82% yield in the presence of a Brønsted acid such as HBr *via* a [3,3]-sigmatropic rearrangement (Scheme 9) (see ESI† for the details on screening of the reaction conditions).^16^ The compound **6** could be easily transformed to the masked alcoholic products *via* normal processes (see ESI† for the details).^17^

**Scheme 9:**
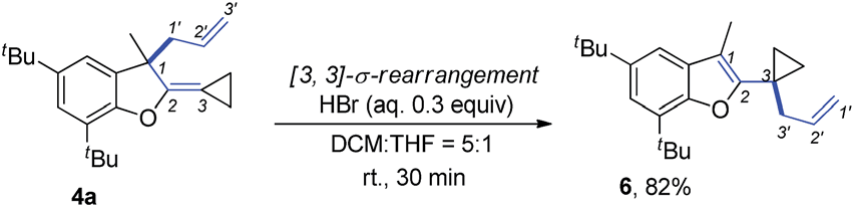
Further transformation of **4a**.

In summary, we have explored a novel gold(i)-catalyzed cycloisomerization of VDCP-ene derivatives *via* carbene or non-carbene processes, in which a substituent adjacent to the oxygen atom could switch the reaction modes. The reaction features a new amphiphilic strategy for gold carbene generation. For non-carbene cyclization, the regioselectivity is dependent upon the steric effect at the alkene moiety. These carbene or non-carbene processes can provide a new synthetic protocol for the divergent synthesis of O-containing heterocyclic scaffolds including a fused tricyclic system and fused five-, and six-membered ring systems. Further investigations to examine the mechanistic details more extensively and exploration of new methodology based on gold catalyzed transformations of VDCPs as well as their asymmetric variants are currently underway in our laboratory.

## Supplementary Material

Supplementary information

Crystal structure data
