# Supplementary material for: Gold(i)-catalyzed cycloisomerization of vinylidenecyclopropane-enes via carbene or non-carbene processes
Source: Chem Sci. 2015 Jun 24;6(10):5519–25. doi: 10.1039/c5sc01806d (PMC5949847; doi:10.1039/c5sc01806d)
Supplement: Supplementary file 1 [file SC-006-C5SC01806D-s001.pdf]

Electronic Supplementary Material (ESI) for Chemical Science.
This journal is © The Royal Society of Chemistry 2015

Supporting Information

Gold(I)-Catalyzed Cycloisomerization of Vinylidenecyclopropane-enes via Carbene or non-Carbene Processes

De-Yao Li,[†] *Yin Wei,*[†] *Ilan Marek,*[‡] *Xiangying Tang,*[†*] *Min Shi*[†*]

[†]State Key Laboratory of Organometallic Chemistry, Shanghai Institute of Organic Chemistry, Chinese Academy of Sciences, 345 Ling-Ling Lu, Shanghai, 200032, China.

siocxiangying@mail.sioc.ac.cn. Mshi@mail.sioc.ac.cn. *Fax* 86-21-64166128

[‡]Schulich Faculty of Chemistry, Technion–Israel Institute of Technology, Technion City, Haifa 32000, Israel

CONTENTS

1. Additional references……………………………………………………..……………..S2-S3
2. General Remarks…………………………………………………………..……………S4
3. General Reaction Procedure……………………………………………………........S5-9
4. Deuterium Labeling Experiment………………………………………....................S10-12
5. Condition Screening on the Transformation of **1a** to **2a**…………………………………S13-14
6. Condition Screening on Asymmetric Version…………………………………………S15-17
7. Condition Screening and Procedures of Transformation of **4a** to **6**…………………..S18
8. Spectroscopic Data…………………………………………………………………...S19-168
9. X-ray Crystal Data of **3e**, **1n**, **2n** and **5k**……………………………………………..S169-172
10. The structure analysis of **4a** and **6**………………………………………………S173-175
11. Computational Details...…………………………………………………………...S176-178
12. Archive Entries……………………………………………………………………..S179-208
13. Procedures of Transformation of **6**……………………………………………....S209
14. Reference…………………………………………………………………………...S210

Additional references

Additional references for ref. [1]: (a) A. Fürstner, *Chem. Soc. Rev.* **2009**, *38*, 3208-3221. (b) D. Wang, R. Cai, S. Sharma, J. Jirak, S. K. Thummanapelli, N. G. Akhmedov, H. Zhang, X. Liu, J. L. Petersen, X. Shi, *J. Am. Chem. Soc.* **2012**, *134*, 9012-9019.

Additional references for ref. [3], For α-diazo carbonyl compounds as gold carbene precursors, see (a) E. Rossi, P. Woehl, M. Maggini, *Org. Process Res. Dev.* **2012**, *16*, 1146-1149. (b) M. R. Fructos, T. R. Belderrain, P. de Fremont, N. M. Scott, S. P. Nolan, M. M. Diaz-Requejo, P. J. Perez, *Angew. Chem., Int. Ed.* **2005**, *44*, 5284-5288. (c) A. Prieto, M. R. Fructos, M. Mar Díaz-Requejo, P. J. Pérez, P. Pérez-Galán, N. Delpont, A. M. Echavarren, *Tetrahedron* **2009**, *65*, 1790-1793. (d) S. K. Pawar, C.-D. Wang, S. Bhunia, A. M. Jadhav, R.-S. Liu, *Angew. Chem., Int. Ed.* **2013**, *52*, 7559-7563. (e) V. V. Pagar, A. M. Jadhav, R.-S. Liu, *J. Org. Chem.* **2013**, *78*, 5711-5716. (f) A. M. Jadhav, V. V. Pagar, R.-S. Liu, *Angew. Chem., Int. Ed.* **2012**, *51*, 11809-11813.

For evidence of gold carbenoids, see (g) A. Fürstner, P. W. Davies, *Angew. Chem. Int. Ed.* **2007**, *46*, 3410-3449. (h) A. Fürstner, *Chem. Soc. Rev.* **2009**, *38*, 3208-3221. (c) C. Obradors, A. M. Echavarren, *Chem. Commun.* **2014**, *50*, 16-28. (i) R. E. M. Brooner, T. J. Brown, R. S. Widenhoefer, *Angew. Chem. Int. Ed.* **2013**, *52,* 6259-6261. (j) T. Lauterbach, S. Gatzweiler, P. Nösel, M. Rudolph, F. Rominger, A. S. K. Hashmi, *Adv. Synth. Catal.* **2013**, *355*, 2481–2487. (k) T. Lauterbach, M. Ganschow, M. W. Hussong, M. Rudolph, F. Rominger, A. S. K. Hashmi, *Adv. Synth. Catal.* **2014**, *356*, 680-686.

Additional references for ref. [4]: (a) B. Lu, C. Li, L. Zhang, *J. Am. Chem. Soc.* **2010**, *132*, 14070-14072. (b) E. L. Noey, Y. Luo, L. Zhang, K. N. Houk, *J. Am. Chem. Soc.* **2012**, *134*, 1078-1084. (c) Y. Wang, A. Yepremyan, S. Ghorai, R. Todd, D. H. Aue, L. Zhang, *Angew. Chem., Int. Ed.* **2013**, *52*, 7795-7799. (d) L. Ye, W. He, L. Zhang, *J. Am. Chem. Soc.* **2010**, *132*, 8550-8551. (e) C.-W. Li, K. Pati, G.-Y. Lin, S. M. A. Sohel, H.-H. Hung, R.-S. Liu, *Angew. Chem., Int. Ed.* **2010**, *49*, 9891-9894. (f) R. B. Dateer, K. Pati, R.-S. Liu, *Chem. Commun.* **2012**, *48*, 7200-7202. (g) H. S. Yeom, J. E. Lee, S. Shin, *Angew. Chem., Int. Ed.* **2008**, *47*, 7040-7043. (h) M. Xu, T.-T. Ren, C.-Y. Li, *Org. Lett.* **2012**, *14*, 4902-4905. (i) L. Wang, X. Xie, Y. Liu, *Angew. Chem., Int. Ed.* **2013**, *52*, 13302-13306. (j) C. Nieto-Oberhuber, P. Perez-Galan, E. Herrero-Gomez, T. Lauterbach, C. Rodriguez, S. Lopez, C. Bour, A. Rosellon, D. J. Cardenas, A. M. Echavarren, *J. Am. Chem. Soc.*

2008, *130*, 269-279. (k) C. Obradors, D. Leboeuf, J. Aydin, A. M. Echavarren, *Org. Lett.* **2013**, *15*, 1576-1579.

Additional references for ref. [8], (a) C. H. Oh, A. Kim, W. Park, D. I. Park, N. Kim, *Synlett* **2006**, 2781-2784. (b) Y. Su, Y. Zhang, N. G. Akhmedov, J. L. Petersen, X. Shi, *Org. Lett.* **2014**, *16*, 2478-2481.

Additional references for ref. [9], For selected reviews, and articles, see (a) A. Brandi, A. Goti, *Chem. Rev.* **1998**, *98*, 589-636. (b) A. Brandi, S. Cicchi, F. M. Cordero, A. Goti, *Chem. Rev.* **2003**, *103*, 1213-1270. (c) L.-X. Shao, M. Shi, *Curr. Org. Chem.* **2007**, *11*, 1135. (d) G. Audran, H. Pellissier, *Adv. Synth. Catal.* **2010**, *352*, 575-608.

General Remarks. ^{1}H NMR spectra were recorded on a Varian Mercury-300 and 400 spectrometer for solution in $CDCl_3$ with tetramethylsilane (TMS) as an internal standard; coupling constants J are given in Hz. ^{13}C NMR spectra were recorded on a Varian Mercury-300 and 400 spectrophotometers (75 or 100 MHz) with complete proton decoupling spectrophotometers ($CDCl_3$: 77.0 ppm). Mass and HRMS spectra were recorded by EI or ESI method. Organic solvents used were dried by standard methods when necessary. Infrared spectra were recorded on a Perkin-Elmer PE-983 spectrometer with absorption in cm^{-1}. Melting points were determined on a digital melting point apparatus and temperatures were uncorrected. Commercially obtained reagents were used without further purification. All these reactions were monitored by TLC with silica gel coated plates. Flash column chromatography was carried out using silica gel at increased pressure.

The compound of 1-(phenylsulfonyl)cyclopropanol and Bestmann-Ohira reagent were prepared following slightly modified literature procedures.[1]

S1 and **S2** are known compounds and prepared according to the previous literature.[2]

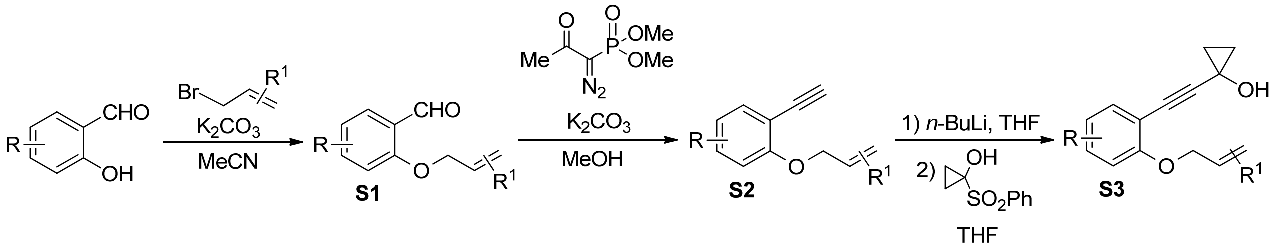

Representative procedure for the preparation of 2-(allyloxy)benzaldehyde (S1):

Substrates **S1** were synthesized following slightly modified literature procedures.[2] To a 250 mL round-bottom flask was added dry K_2CO_3 (5.528 g, 40 mmol) and salicylaldehyde (2.442 g, 20 mmol). Then 40 mL MeCN was added as the solvent and 3-bromopropene (3.630 g, 30 mmol) was added dropwise via a syringe. The resulting yellow-green reaction mixture was stirred at 60 ^{o}C for 6 h. After that, the reaction mixture was filtered to remove the resulting salts and redundant K_2CO_3. The organic phase was concentrated under reduced pressure and the residue was used directly without purification.

Representative procedure for the preparation of 1-(allyloxy)-2-ethynylbenzene (S2):

Substrates **S2** were synthesized following slightly modified literature procedures.[3] To a flame dried, argon purged round bottom flask was added 2-(allyloxy)benzaldehyde (used directly without purification approximately 20 mmol), dissolved in anhydrous methanol (40 mL), and K_2CO_3 (8.294 g, 60 mmol) was added. Then, Bestmann-Ohira reagent (5.761 g, 30 mmol) was added slowly via a syringe. The reaction mixture was stirred at room temperature for 1 h. After that, the solvent was removed under reduced pressure. Then water (20 mL) was added and the mixture was extracted with ethyl acetate (45 mL) three times. The organic phase was concentrated under reduced pressure and the residue was purified by a silica gel flash column chromatography with petroleum ether-EtOAc (20:1) as an eluent.

Representative procedure for the preparation of 1-((2-(allyloxy)phenyl)ethynyl)cyclopropanol (S3):

Substrates **S3** were synthesized following slightly modified literature procedures.[4] To a flame dried, argon purged three-necked flask (marked 1) was added 2-(allyloxy)benzaldehyde substrate (2.371 g, 15 mmol) which was dissolved in THF (30 mL). To another flame dried, argon purged three-necked

flask (marked 2) was added 1-(phenylsulfonyl)cyclopropanol (3.267 g, 16.5 mmol) which was also dissolved in THF (30 mL). The two flasks were set in dry ice-acetone bath and cooled down to -78 °C. When the temperature reached, *n*-BuLi (6.6 mL, 2.5 M/L in THF) was added dropwise to each of the flasks via syringes at the same temperature. The reaction mixtures were stirred at that temperature for 2 h. Then, the resulting mixture in flask 1 was transferred to flask 2 and the reaction vessel was removed from the bath and naturally warmed to room temperature. The reaction mixture was stirred at room temperature for 24 h. After that, the resulting dark mixture was quenched with water (20 mL) and extracted with ethyl acetate (45 mL) three times. The organic phase was concentrated under reduced pressure and the residue was purified by a silica gel flash column chromatography with petroleum ether-EtOAc (10:1) as an eluent.

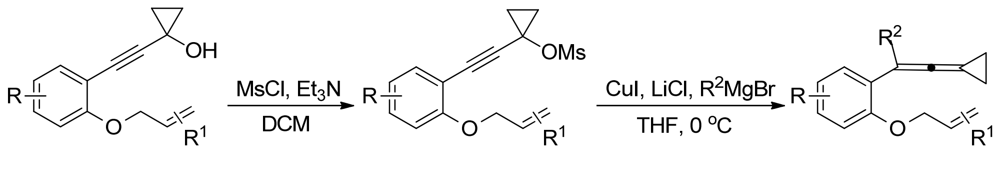

Representative procedure for the preparation of 1-((2-(allyloxy)phenyl)ethynyl)cyclopropyl methanesulfonate (S4):

Substrates **S4** were synthesized following slightly modified literature procedures.[4] To a flame dried, argon purged round bottom flask was added 1-((2-(allyloxy)phenyl)ethynyl)cyclopropanol (1.070 g, 5 mmol), dissolved in dry dichloromethane (20 mL), then freshly redistilled triethylamine (758 mg, 7.5 mmol) was added. The reaction mixture was stirred and cooled down to 0 °C with an ice-water bath. When the temperature reached, methanesulfonyl chloride (684 mg, 6 mmol) was added dropwise into the flask via a syringe. The reaction mixture was further stirred for 1 h. After that, the reaction was quenched by the addition of water (10 mL) and the organic mixture was extracted with dichloromethane (30 mL) three times. The organic phase was concentrated under reduced pressure and the residue was purified by a silica gel flash column chromatography with petroleum ether-EtOAc (10:1) as an eluent.

Representative procedure for the synthesis of substrates 1:

To a flame dried, argon purged Schlenk tube was added LiCl (336 mg, 8.0 mmol), heated

vigorously, evacuated to dry, and purged with argon three times. Then CuI (1.519 g, 8.0 mmol) was added into the reaction vessel under argon. The reaction tube was set in ice-water bath and was added with THF (5 mL) to generate a white suspension. Grignard reagent (8.0 mmol) was added and the reaction mixture was stirred for 10 min. After that, 1-((2-(allyloxy)phenyl)ethynyl)cyclopropyl methanesulfonate (3.0 mmol) dissolved in THF (10 mL) was added into the reaction tube. The reaction was carried out at 0 °C for 4 h. After that, the reaction was quenched by water (5 mL) and the reaction mixture was extracted with petroleum ether. The organic phase was concentrated under reduced pressure and the residue was purified by a silica gel flash column chromatography with petroleum ether as an eluent.

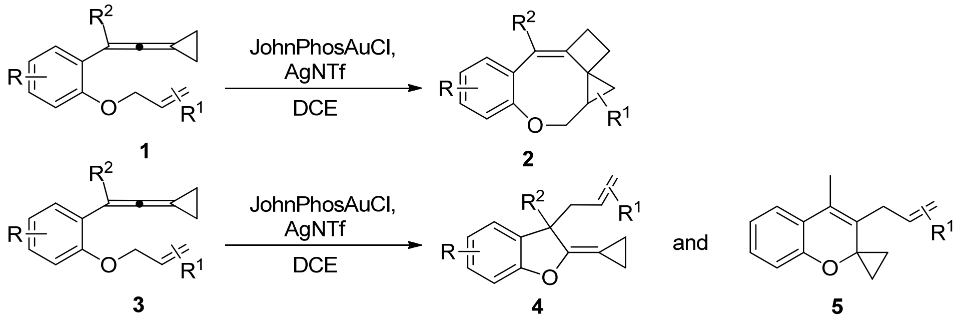

General procedure for the synthesis of compound 2, 4 & 5:

To a flame dried, argon purged Schlenk tube were added Au cat. (25 mg, 0.05 mmol) and Ag salt (19 mg, 0.05 mmol). Then DCE (2 mL) was added to generate a white suspension in which AgCl was generated. After 5 min, compound **1** (0.5 mmol) dissolved in DCE (2 mL) was added into the reaction tube. The reaction was carried out at room temperature for 5 minutes. After that, the reaction mixture was filtered through a thin layer of celite. The organic phase was concentrated under reduced pressure and the residue was purified by a silica gel flash column chromatography with petroleum ether as an eluent.

3k's regioisomer, O-(1,1-dimethylprop-2-enyl) derivative, is unavailable via the present synthetic method:

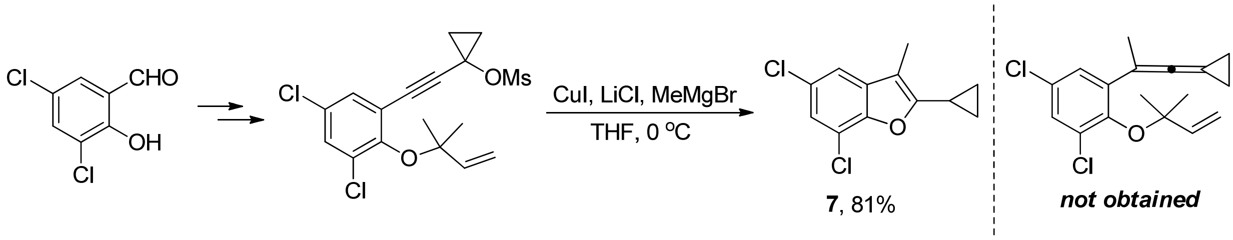

Compound **7**. 486 mg, yield: 81%; white solid. MP: 63-64 °C. ^{1}H NMR ($CDCl_3$, 400 MHz, TMS) δ 0.98-1.03 (m, 2H, CH_2), 1.08-1.12 (m, 2H, CH_2), 1.94-2.00 (m, 1H, CH), 2.16 (s, 3H, CH_3), 7.119-7.124 (d, *J* = 1.6 Hz, 1H, Ar), 7.190-7.194 (d, *J* = 1.6 Hz, 1H, Ar). ^{13}C NMR ($CDCl_3$, 100 MHz, TMS) δ 6.96, 7.72, 13.7, 109.9, 116.3, 116.5, 122.7, 127.8, 133.2, 147.4, 156.8. IR (neat) ν 3089, 3010, 2921, 1604, 1576, 1439, 1428, 1324, 1221, 1176, 1089, 936, 843, 770 cm^{-1}. MS (%) m/e 242 (64.19), 241 (37.00), 240 (M^+, 100.00), 239 (36.88), 227 (32.94), 225 (50.94), 215 (20.12), 213 (32.22). HRMS (EI) calcd. for $C_{12}H_{10}Cl_2O$: 240.0107, Found: 240.0109.

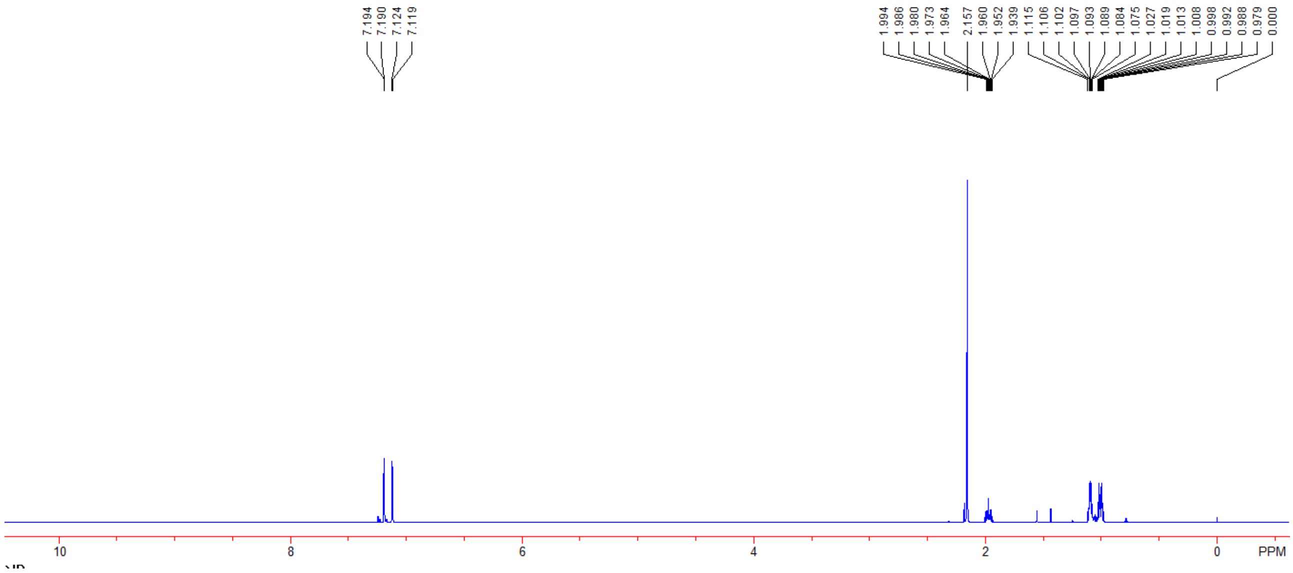

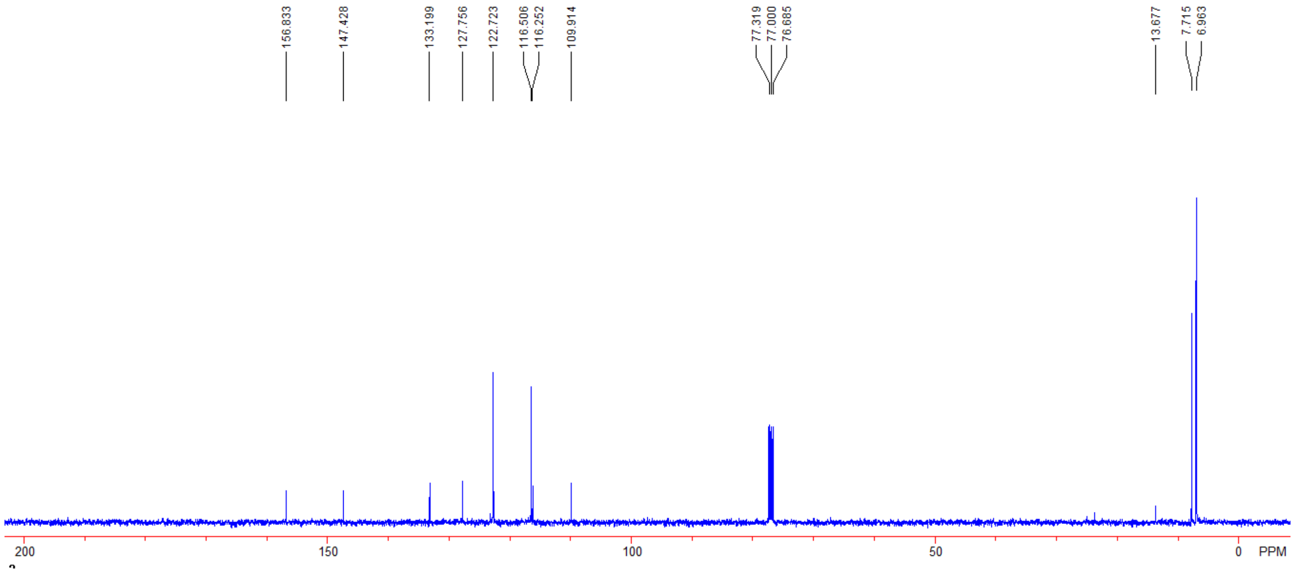
156.833
147.428
133.199
127.756
122.723
116.506
116.252
109.914
77.319
77.000
76.685
13.677
7.715
6.963
200
150
100
50
0
PPM

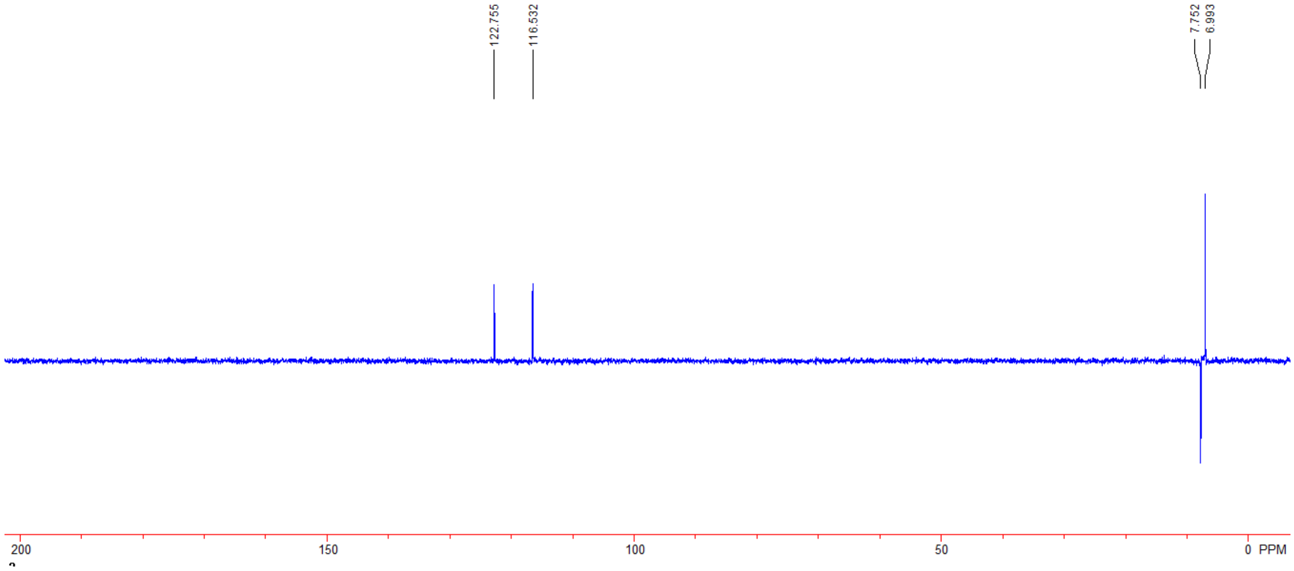
122.755
116.532
7.752
6.993
200
150
100
50
0 PPM

Deuterium Labeling Experiment

1)

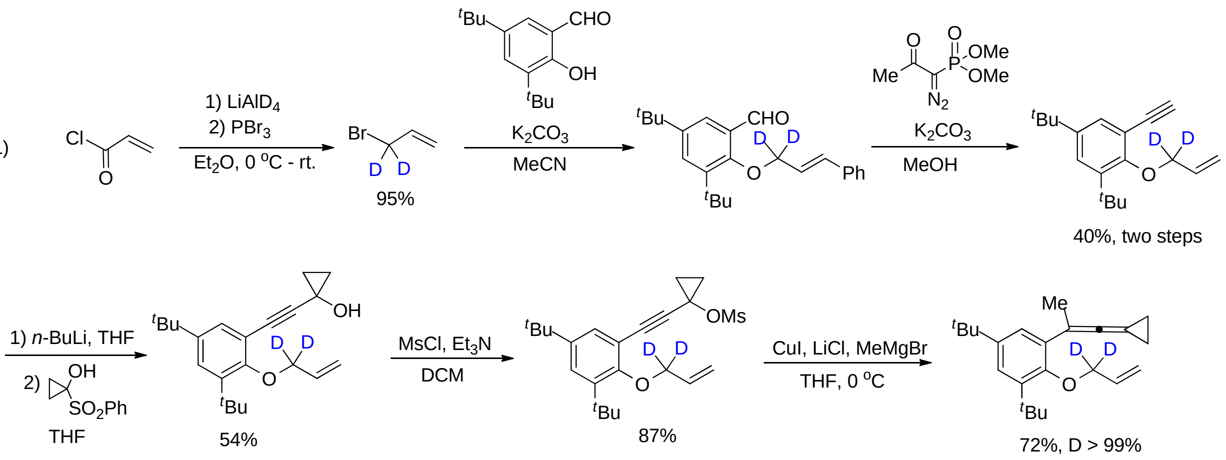

2)

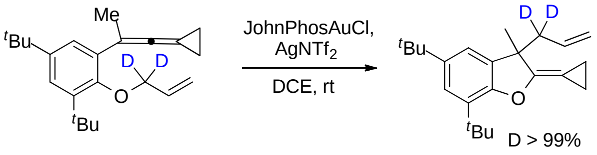

[D]-**3a** [D]-**4a**

3)

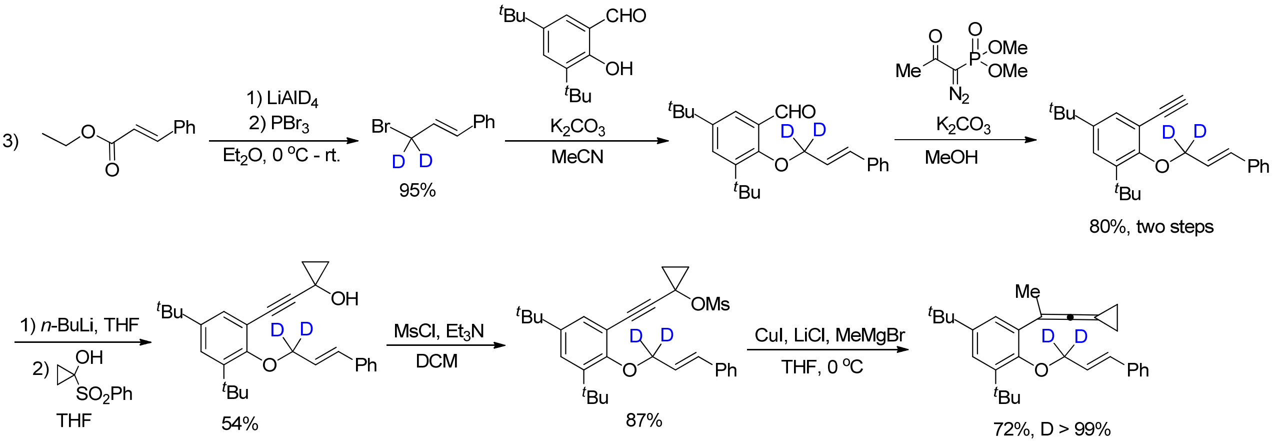

4)

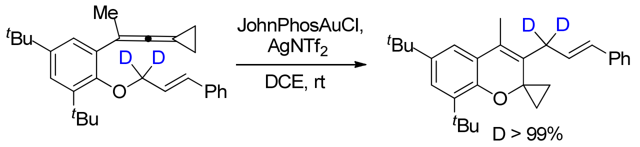

[D]-**3l** [D]-**5l**

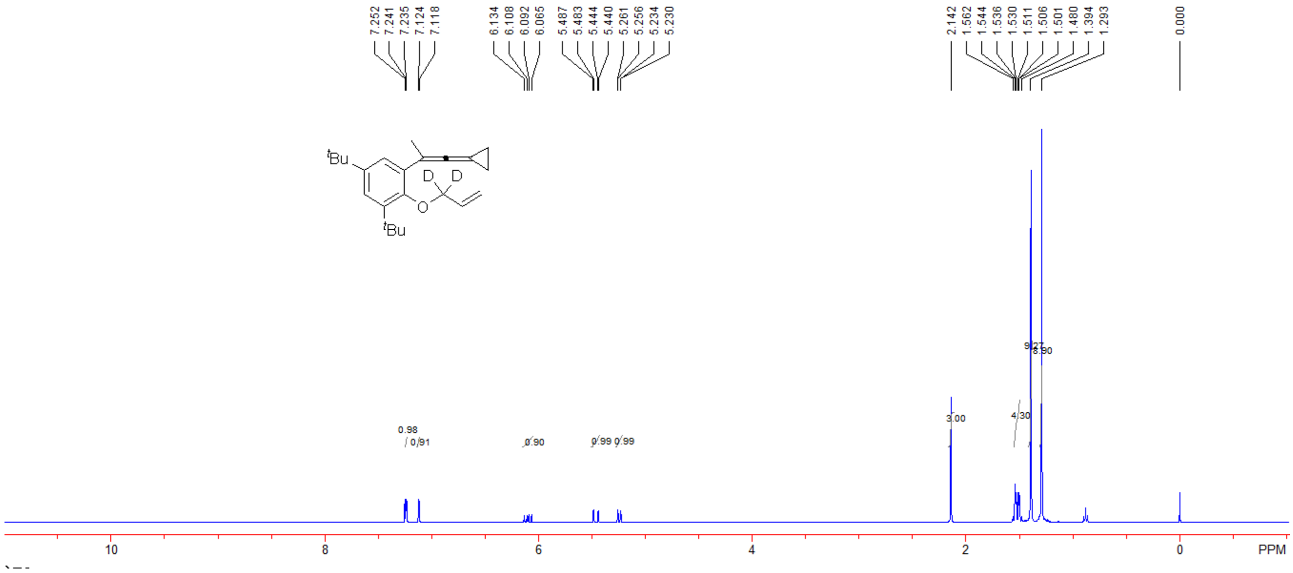

[D]-**3a**

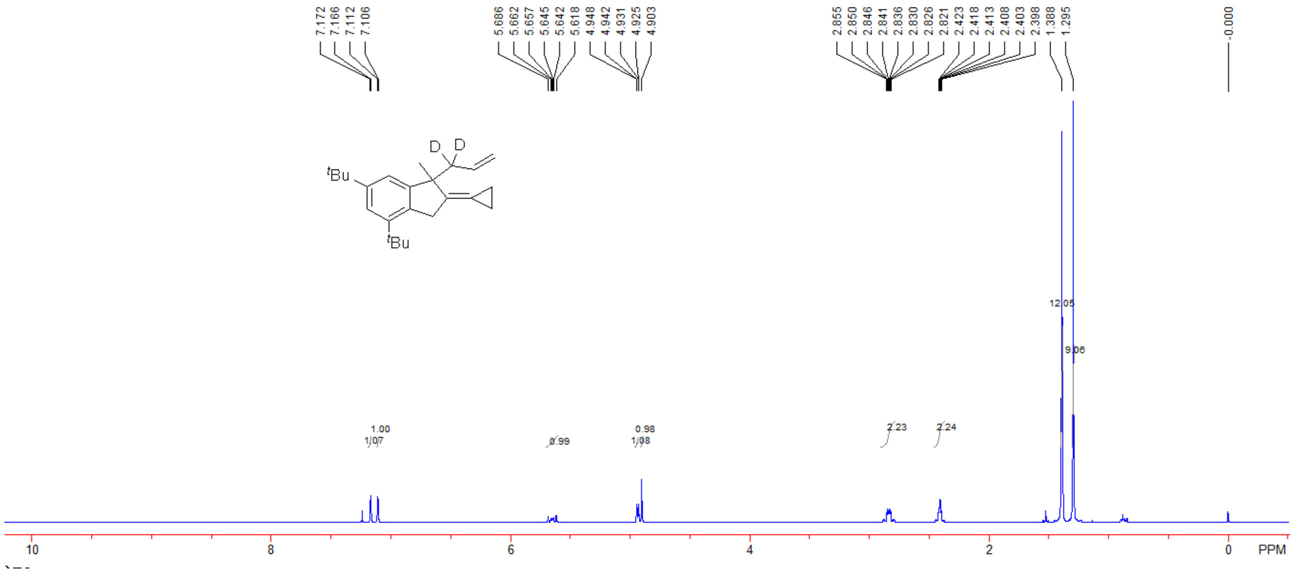

[D]-**4a**

Additional Deuterium labeling experiment

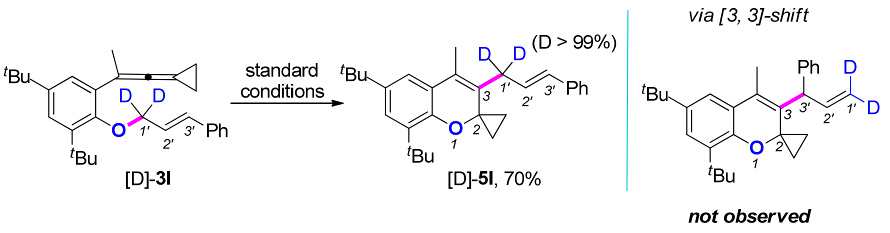

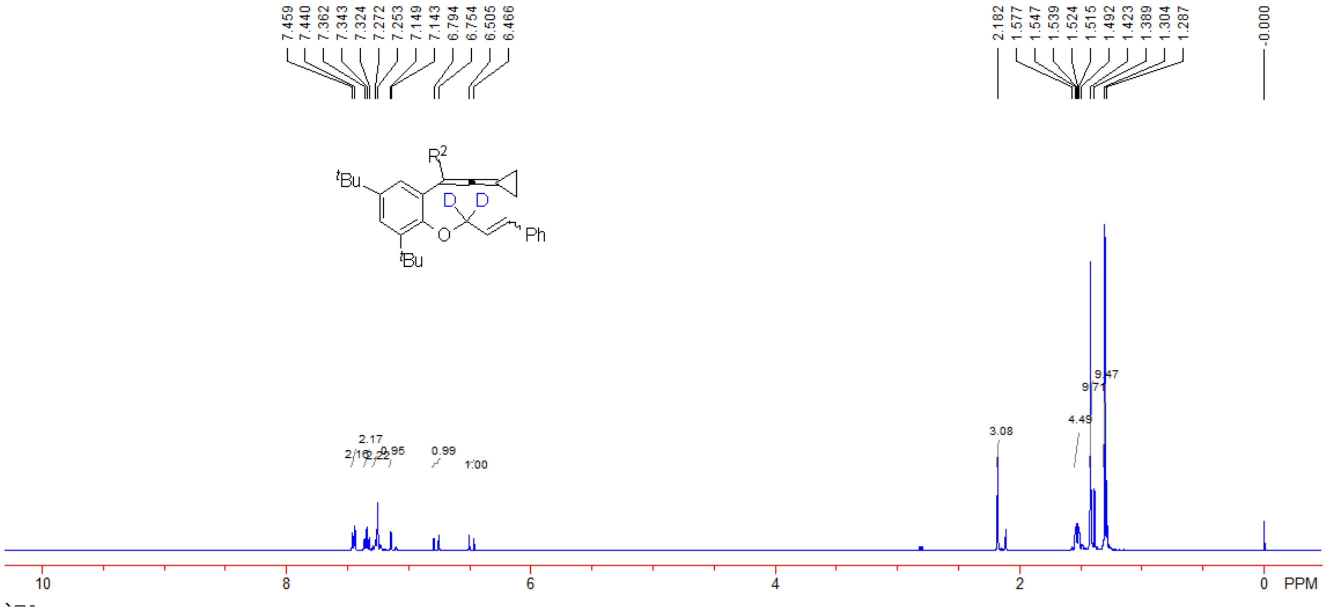

[D]-**3l**

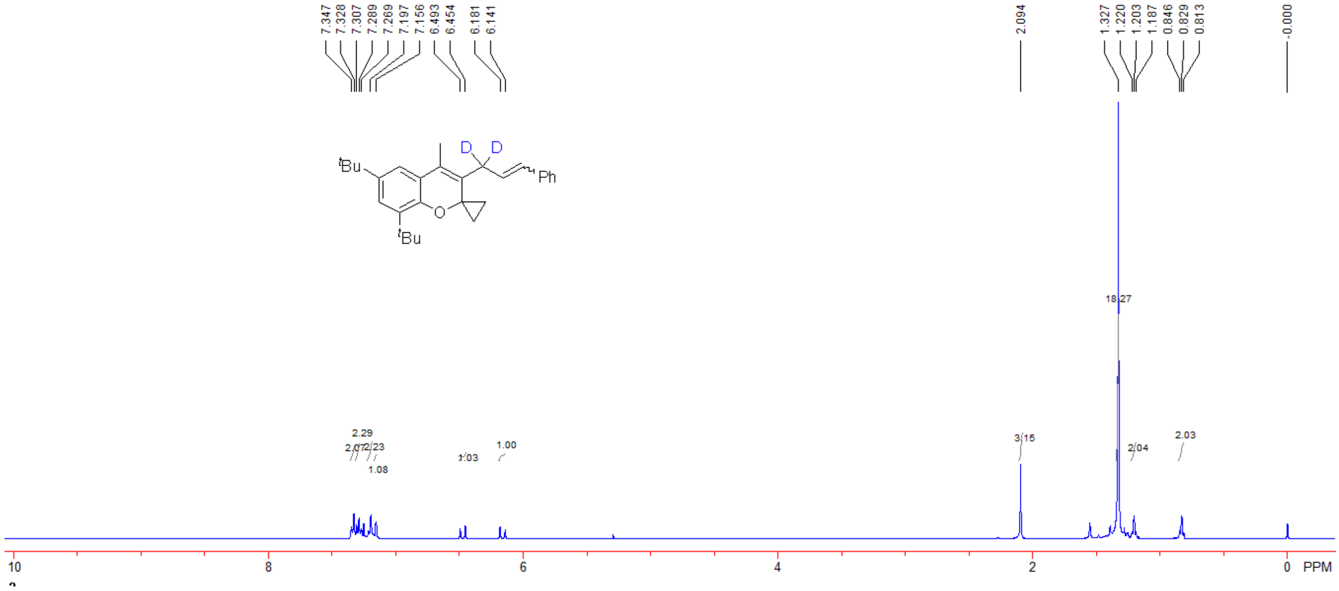

[D]-**5l**

Condition Screening on the Transformations of 1a to 2a

First, $PCy_3AuCl/AgSbF_6$ (entry 1) was employed and it is gratifying that the reaction was completed within 10 minutes and the expected product can be cleanly separated, but the yield is unacceptably low. To optimize the reaction condition, we performed the reactions of substrate **1a** with different gold catalysts and different silver salts with different counterions as well as the solvents (Table S1). Commonly used phosphinegold(I), NHC-gold(I) and two-coordinated phosphinegold(I) complexes all gave depressing results (entries 1-5). However, we found that the employed biphenyl-type phosphinegold(I) dramatically increased the yields of **2a**. Thus, this type of several gold catalysts was evaluated and we found that $JohnPhosAuCl/AgSbF_6$ led to the formation of **2a** in a yield of 78%. Then, using JohnPhosAuCl as the preferred gold catalyst, different Ag salts were employed to examine the counterion effects (entries 8, 14-16). It was found that NTf_2^- as the counterion gave the best result. Besides, reducing the catalyst loading, exposure to moisture, or using directly $JohnPhosAuNTf_2$ did not significantly affect the yields of **2a**.

Table S1. Condition Survey for the Transformation of VDCP to Polycyclic Compound 2a

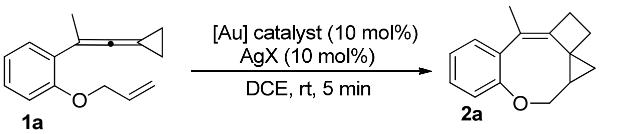

entry	[Au] catalyst	AgX	yield (%)[b]
1	PPh_3AuCl	$AgSbF_6$	complex
2	PCy_3AuCl	$AgSbF_6$	trace
3	IPrAuCl	$AgSbF_6$	complex
4	DPE-PhosAuCl	$AgSbF_6$	trace
5	$dppe(AuCl)_2$	$AgSbF_6$	trace
6	XPhosAuCl	$AgSbF_6$	64
7	^{t}BuXPhosAuCl	$AgSbF_6$	53
8	JohnPhosAuCl	$AgSbF_6$	78
9	Cy_2JohnPhosAuCl	$AgSbF_6$	37
10	$Me_4{}^t$BuXPhosAuCl	$AgSbF_6$	40
11	JakiePhosAuCl	$AgSbF_6$	33
12	SPhosAuCl	$AgSbF_6$	complex
13	-	$AgSbF_6$	n.r.
14	JohnPhosAuCl	AgOTf	trace
15	JohnPhosAuCl	$AgNTf_2$	93
16	JohnPhosAuCl	$AgBF_4$	trace
17[c]	JohnPhosAuCl	$AgNTf_2$	81
18	$JohnPhosAuNTf_2$	-	92
19[d]	JohnPhosAuCl	$AgNTf_2$	70

[a]Reactions were conducted with 0.5 mmol of **1a**, 0.05 mmol of gold catalyst, and 0.05 mmol of silver salt in 5.0 mL of solvent under Ar. [b]Isolated yields. [c]0.025 mmol of gold catalyst and 0.025 mmol of silver salt were loaded. [d]1.0 eq. of water was added.

Solvents Screening

DCE has been proved to be the best solvent.

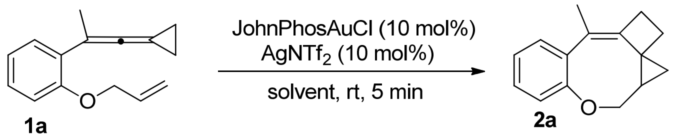

1a **2a**

entry	solvents	yield (%)[a]
1	DCM	90
2	toluene	85
3	MeCN	17
4	THF	24
5	acetone	40
6	DCE	93

[a] Isolated yields

Asymmetric Version, Screening of the Reaction Conditions of 1h

Using **1h** as substrate to screen the reaction conditions:

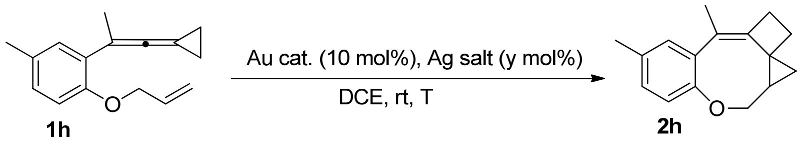

entry[a]	Au cat.	Ag salt cat. y (mol%)	yield (%)[b]	ee (%)
1	xyl-PHANEPhos$(AuCl)_2$	$AgNTf_2$, 10	35	-30
2	*R*-Xyl-BINAP$(AuMeCNSbF_6)_2$	-	52	70
3	*R*-DM-SegPhos$(AuCl)_2$	$AgSbF_6$, 10	-	-
4	*R*-DTMB-SegPhos$(AuCl)_2$	$AgSbF_6$, 10	-	-
5	Au cat. **L1**	-	30	-7
6	Au cat. **L2**	-	24	-13
7	Au cat. **L3**	-	-	-
8	Au cat. **L4**	-	39	-24
9	Au cat. **L5**	-	51	-46
10	*R*-Xyl-BINAP$(AuCl)_2$	$AgSbF_6$, 10	31	23
11	*R*-Xyl-BINAP$(AuCl)_2$	NaBArF, 10	-	-
12	*R*-Xyl-BINAP$(AuCl)_2$	AgOTf, 10	-	-
13	*R*-Xyl-BINAP$(AuCl)_2$	$AgNTf_2$, 10	47	50
14[c]	*R*-Xyl-BINAP$(AuMeCNSbF_6)_2$	-	15	24
15[d]	*R*-Xyl-BINAP$(AuMeCNSbF_6)_2$	-	80	81

[a] The reaction was carried out on a 0.1 mmol scale in DCE (2.0 mL). [b] Isolated yield. [c] CH_3CN was used as the solvent. [d] Toluene was used as the solvent.

Additional results:

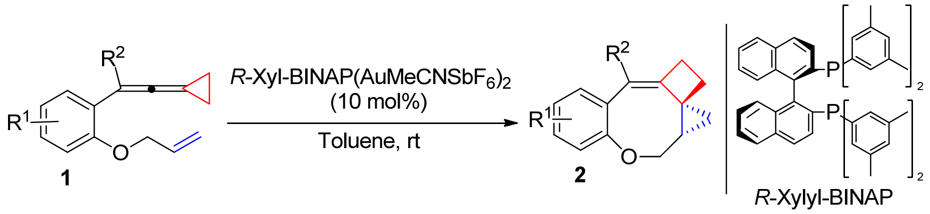

R^1	R^2	yield%	*ee*%
5-Br	p-MeC_6H_4	68	68
5-Br	p-BrC_6H_4	53	69

For the employed ligands, see next page.

Asymmetric Version, Screening of the Reaction Conditions using 3a

Using **3a** as substrate to screen the reaction conditions:

Temperature and solvents were screened and no better results were obtained

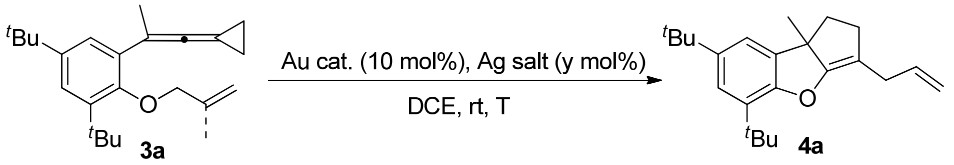

entry[a]	Au cat.	Ag salt cat. y (mol%)	yield (%)[b]	ee (%)
1	xyl-PHANEPhos$(AuCl)_2$	$AgNTf_2$, 10	36	59
2	*R*-Xyl-BINAP$(AuMeCNSbF_6)_2$	-	32	30
3	*R*-DM-SegPhos$(AuCl)_2$	$AgSbF_6$, 10	42	67
4	*R*-DM-SegPhos$(AuCl)_2$	$AgSbF_6$, 20	42	54
5	*R*-DTBM-SegPhos$(AuCl)_2$	$AgSbF_6$, 10	24	40
6	Au cat. **L1**	-	10	-
7	Au cat. **L2**	-	24	-13
8	Au cat. **L3**	-	7	-13
9	Au cat. **L5**	-	51	-46
10	*R*-Xyl-BINAP$(AuCl)_2$	$AgNTf_2$, 10	64	22

[a] The reaction was carried out on a 0.1 mmol scale in solvents (2.0 mL). [b] Isolated yield.

Chiral Ligands:

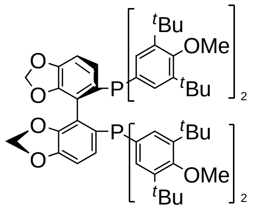

R-DTBM-SegPhos

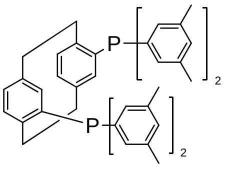

xyl-PHANEPhos

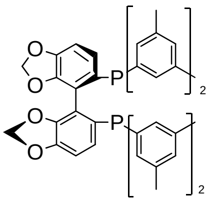

R-DM-SegPhos

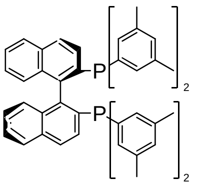

R-Xyl-BINAP

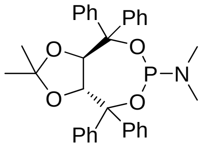

L1

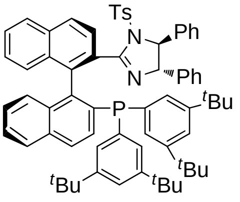

L2

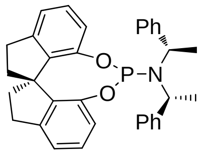

L3

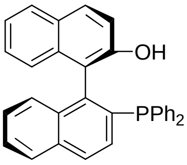

L4

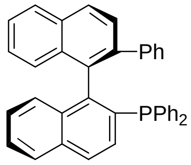

L5

Additional results:

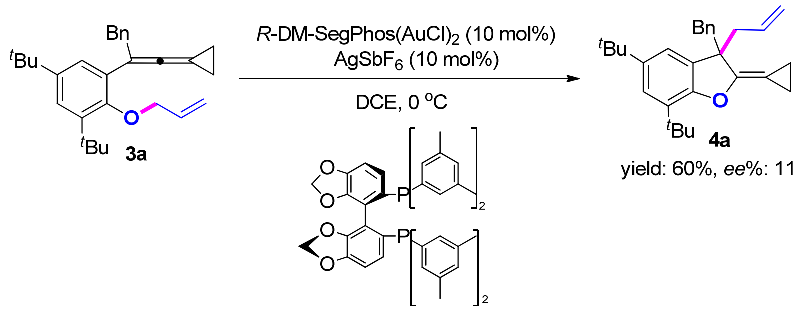

Bn
tBu
tBu
3a
R-DM-SegPhos(AuCl)2 (10 mol%)
AgSbF6 (10 mol%)
DCE, 0 oC
Bn
tBu
tBu
4a
yield: 60%, ee%: 11

Condition Screening on the Transformations of 4a to 6

According to the work of Gagné's group, different Lewis acid, Brønsted acid and π acid are preliminary tested. We found that Brønsted acid HBr gave the best result, affording product **6** in 82% yield.

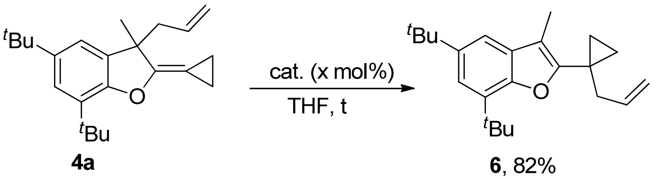

entry	cat.	x (mol%)	t (min)	yield (%)[a]
1	PPh_3AuNTf_2	5	60 min	32
2	HBr	30	30 min	82
3	$BF_3 \cdot Et_2O$	100	30 min	77

[a] Isolated yields.

Procedure for the synthesis of compound 6 with modified reaction conditions:

To a flame dried, argon purged Schlenk tube was added a solution of **4a** in THF (0.2 M, 5mL), then HBr (0.3 mmol) was added into the reaction tube. The reaction was carried out at room temperature for 30 minutes. After that, the organic phase was concentrated under reduced pressure and the residue was purified by a silica gel flash column chromatography with petroleum ether as an eluent.

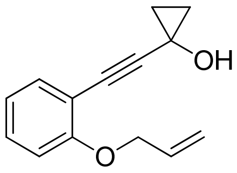

Compound **S3a**. 2.272 g, yield: 69%; yellow oil. ^{1}H NMR ($CDCl_3$, 400 MHz, TMS) δ 1.07-1.12 (m, 2H, CH_2), 1.14-1.19 (m, 2H, CH_2), 3.24 (br, 1H, OH), 4.58-4.60 (m, 2H, CH_2), 5.26-5.30 (m, 1H, =CH_2), 5.44-5.49 (m, 1H, =CH_2), 6.00-6.10 (m, 1H, =CH), 6.82-6.89 (m, 2H, Ar), 7.20-7.25 (m, 1H, Ar), 7.35-7.38 (m, 1H, Ar). ^{13}C NMR ($CDCl_3$, 100 MHz, TMS) δ 17.5, 46.0, 69.1, 78.3, 95.1, 112.3, 112.5, 117.1, 120.6, 129.3, 132.9, 133.3, 158.9. IR (neat) ν 3359, 3080, 2866, 2194, 1713, 595, 1490, 1445, 1423, 1277, 1225, 1185, 1046, 994, 872, 748 cm^{-1}. MS (%) m/e 214 (M^+, 6.67), 157 (62.52), 145 (66.98), 131 (87.71), 128 (65.00), 115 (100.00), 89 (56.08), 55 (83.52), 41 (49.04). HRMS (EI) calcd. for $C_{14}H_{14}O_2$: 214.0994, Found: 214.0989.

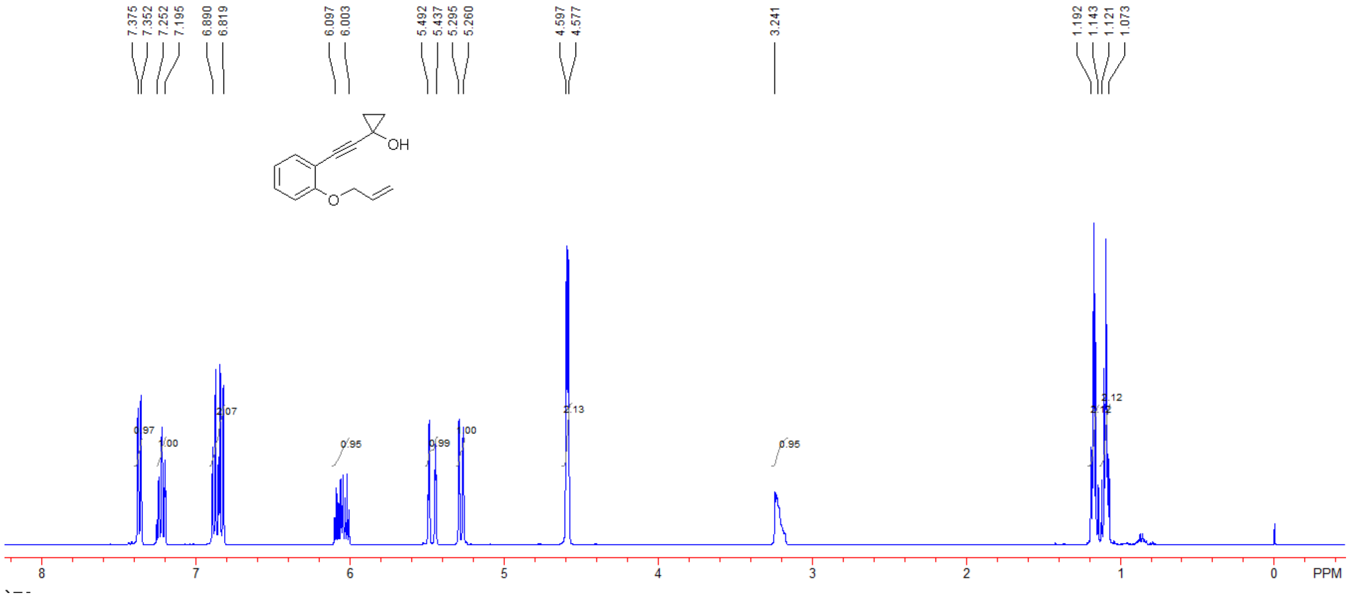

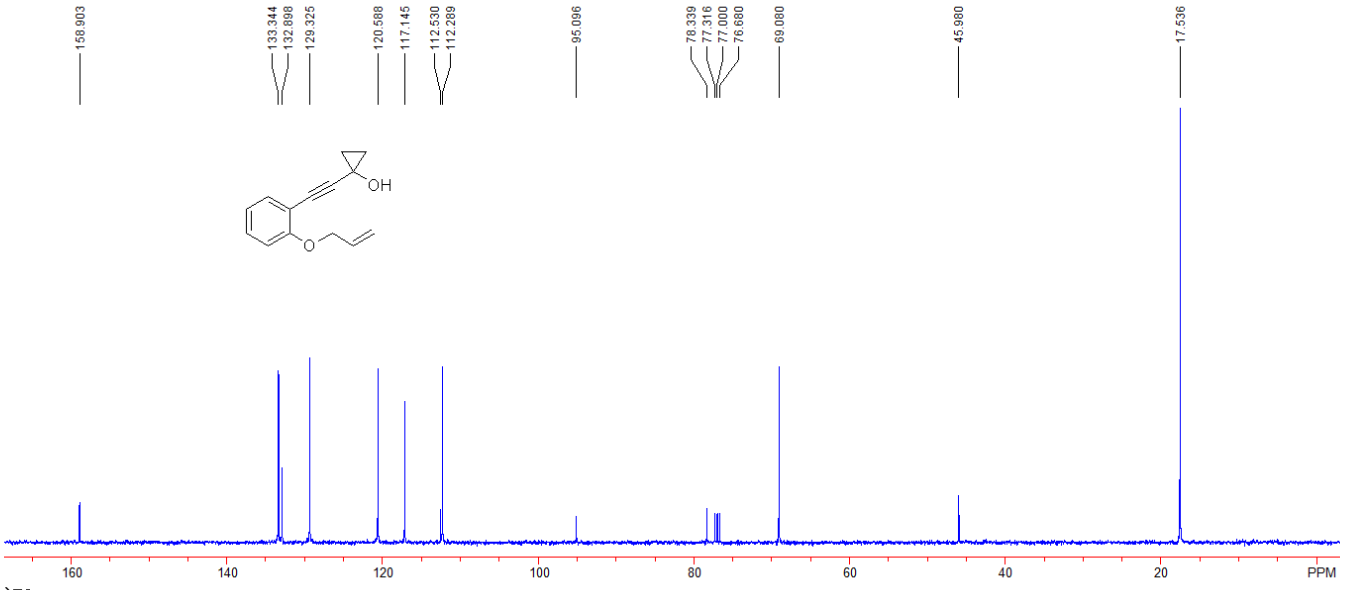

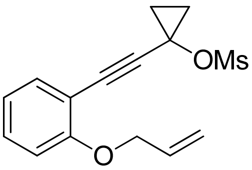

Compound **S4a**. 452 mg, yield: 52%; yellow oil. ^{1}H NMR ($CDCl_3$, 400 MHz, TMS) δ 1.34-1.38 (m, 2H, CH_2), 1.63-1.67 (m, 2H, CH_2), 3.31 (s, 3H, CH_3), 4.56-4.58 (m, 2H, CH_2), 5.29-5.33 (m, 1H, $=CH_2$), 5.42-5.47 (m, 1H, $=CH_2$), 6.00-6.09 (m, 1H, =CH), 6.85-6.93 (m, 2H, Ar), 7.26-7.31 (m, 1H, Ar), 7.34-7.37 (m, 1H, Ar). ^{13}C NMR ($CDCl_3$, 100 MHz, TMS) δ 16.9, 39.9, 55.0, 69.1, 82.9, 89.5, 111.1, 111.9, 117.9, 120.6, 130.3, 132.6, 133.2, 159.3. IR (neat) ν 3020, 2961, 2870, 2226, 1594, 1491, 1359, 1280, 1245, 1157, 1113, 995, 948, 801, 750 cm^{-1}. MS (%) m/e 292 (M^+, 4.18), 171 (32.32), 128 (100.00), 127 (34.37), 116 (32.06), 115 (45.85), 88 (29.42), 55 (26.60), 41 (25.67). HRMS (EI) calcd. for $C_{15}H_{16}O_4S$: 292.0769, Found: 292.0767.

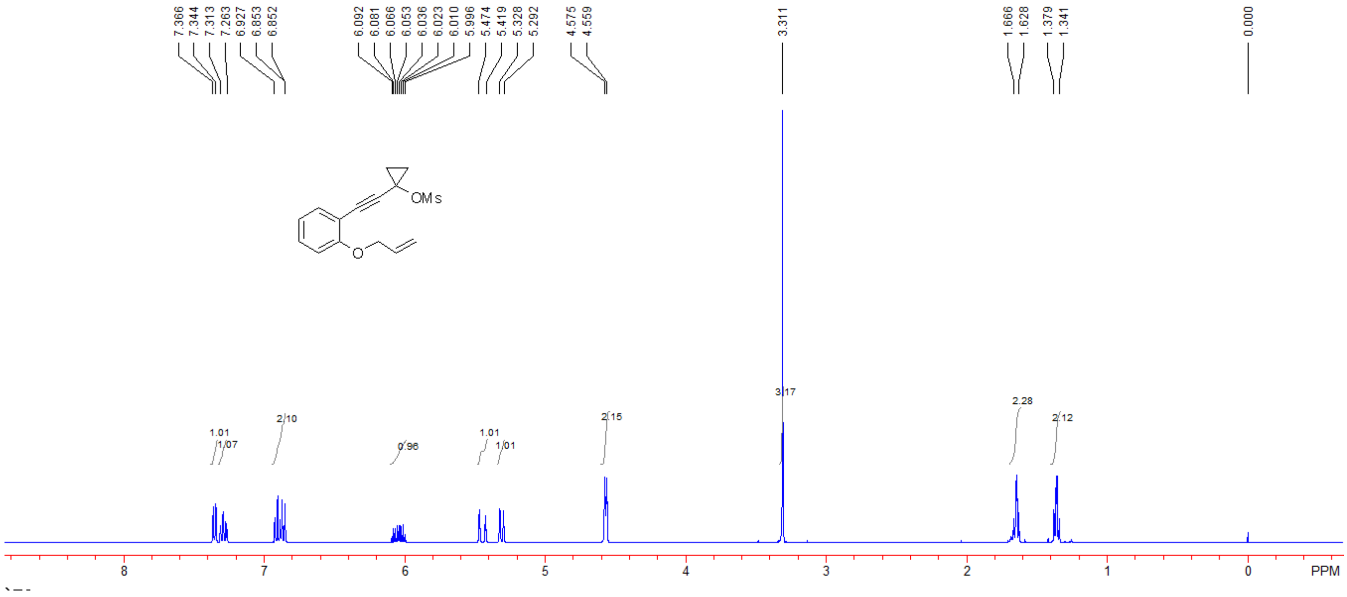

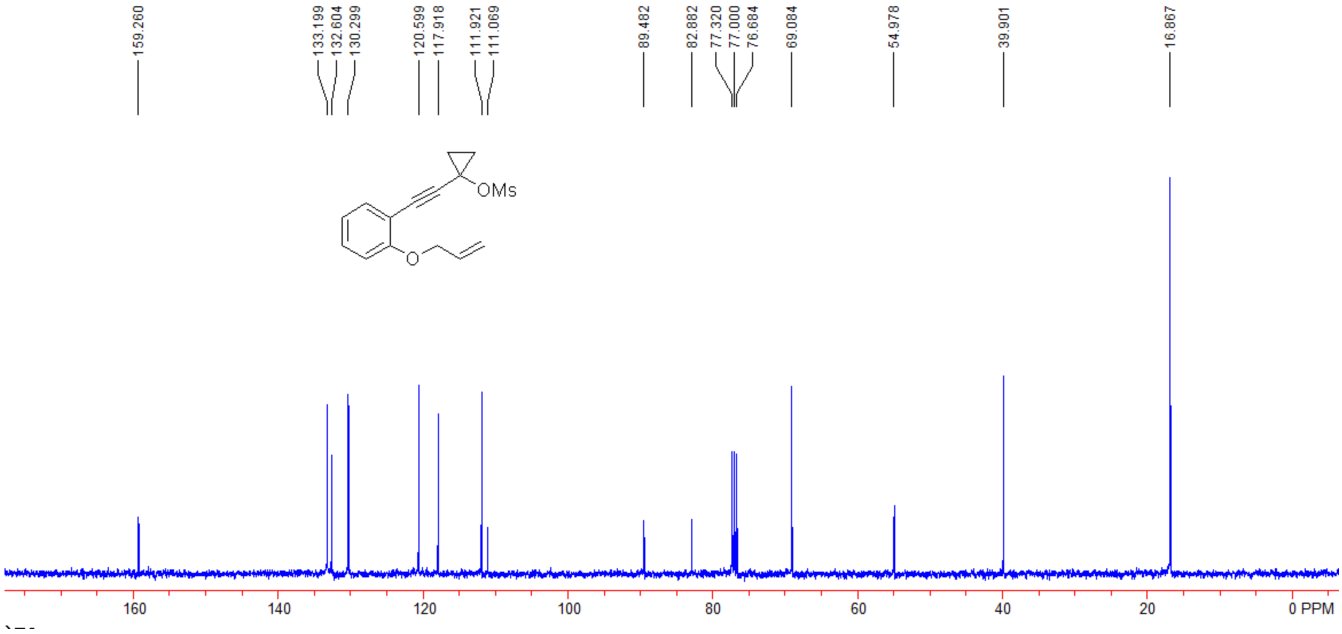

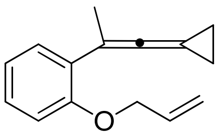

Compound **1a**. 212 mg, yield: 79%; colorless oil. ^{1}H NMR ($CDCl_3$, 400 MHz, TMS) δ 1.46-1.50 (m, 2H, CH_2), 1.51-1.56 (m, 2H, CH_2), 2.18 (s, 3H, CH_3), 4.50 (d, J = 4.2 Hz, 2H, CH_2), 5.22-5.25 (m, 1H, =CH_2), 5.35-5.40 (m, 1H, =CH_2), 5.96-6.06 (m, 1H, =CH), 6.83 (d, J = 8.0 Hz, 1H, Ar), 6.91 (t, J = 8.0 Hz, 1H, Ar), 7.12-7.16 (m, 1H, Ar), 7.26 (d, J = 8.0 Hz, 1H, Ar). ^{13}C NMR ($CDCl_3$, 100 MHz, TMS) δ 7.5, 20.1, 69.2, 75.3, 100.4, 112.6, 117.3, 120.7, 127.6, 128.6, 128.8, 133.5, 155.9, 190.6. IR (neat) ν 3053, 2979, 2910, 2005, 1591, 1488, 1420, 1299, 1282, 1246, 1133, 1041,

1013, 929, 744 cm^{-1}. MS (%) m/e 212 (M^+, 0.98), 171 (100.00), 153 (15.53), 152 (27.14), 141 (14.72), 128 (68.25), 127 (20.67), 115 (26.14), 43 (22.62). HRMS (EI) calcd. for $C_{15}H_{16}O$: 212.1201, Found: 212.1202.

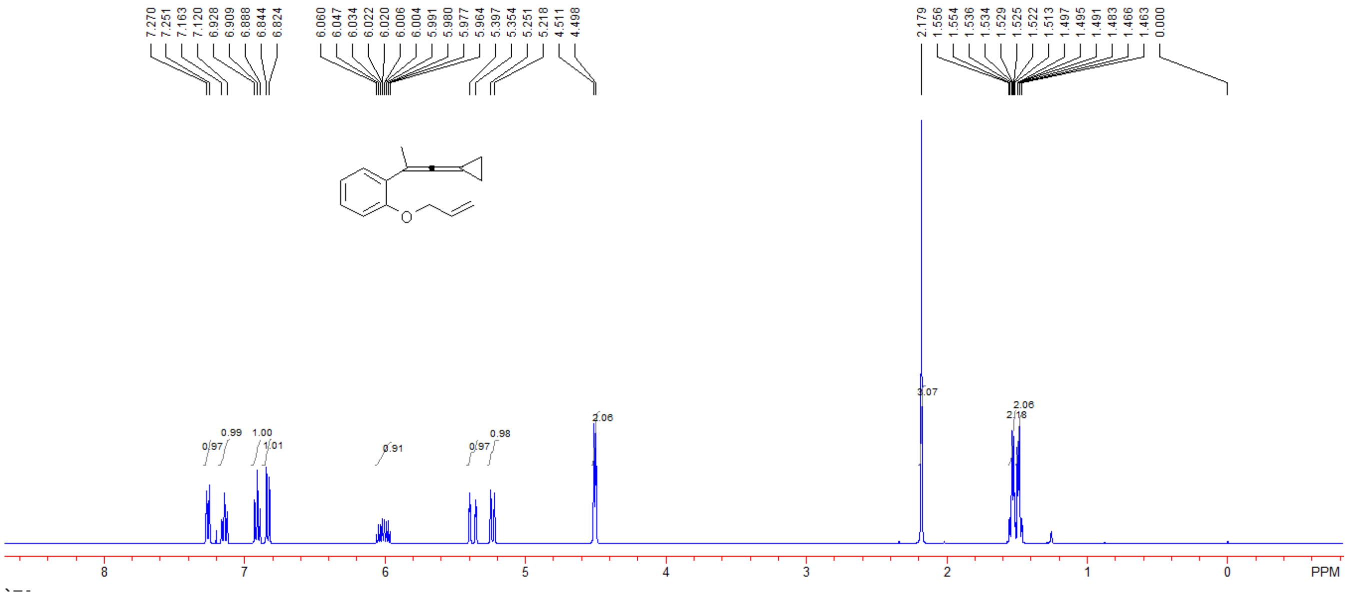

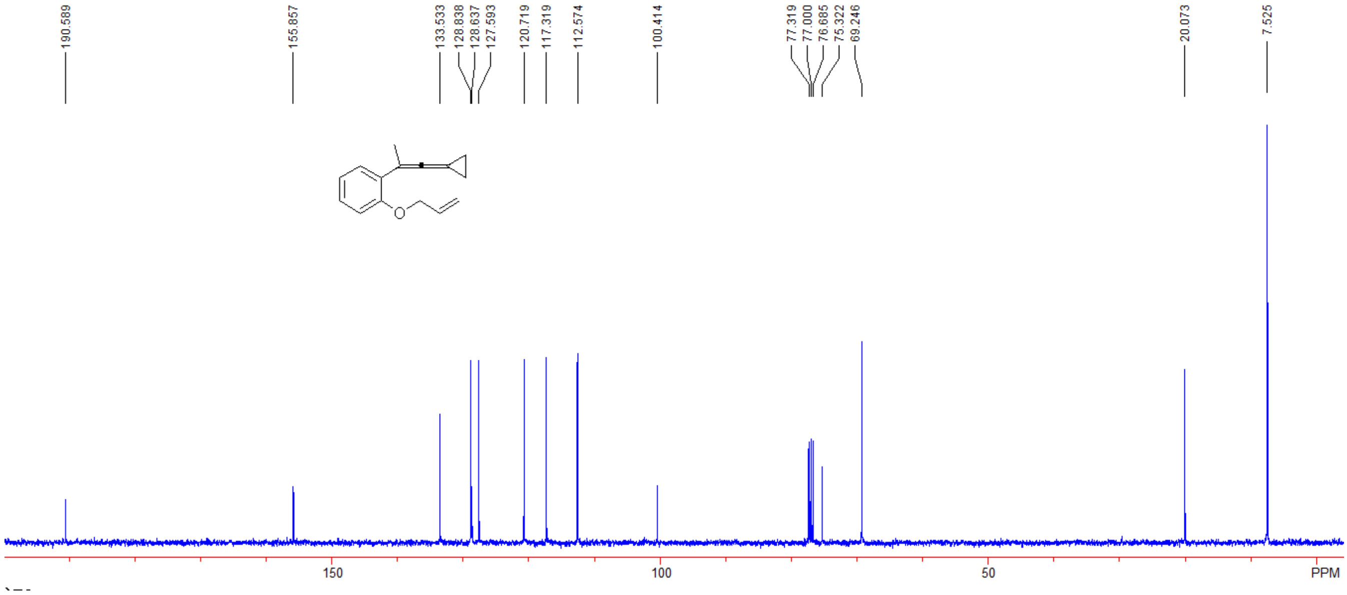

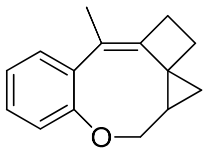

Compound **2a**. 98 mg, yield: 93%; colorless oil. ^{1}H NMR ($CDCl_3$, 400 MHz, TMS) δ 0.34 (dd, J_1 = 4.8 Hz, J_2 = 7.2 Hz, 1H, CH_2), 0.46 (dd, J_1 = 4.8 Hz, J_2 = 9.6 Hz, 1H, CH_2), 1.14-1.19 (m, 1H, CH), 1.95 (s, 3H, CH_3), 2.12-2.25 (m, 2H, CH_2), 2.78-2.86 (m, 1H, CH_2), 2.98-3.06 (m, 1H, CH_2), 4.36

(d, J = 12.8 Hz, 1H, CH_2), 4.83 (dd, J_1 = 6.0 Hz, J_2 = 12.8 Hz, 1H, CH_2), 6.94-6.96 (m, 1H, Ar), 6.99-7.03 (m, 1H, Ar), 7.15-7.19 (m, 1H, Ar), 7.22-7.24 (m, 1H, Ar). ^{13}C NMR ($CDCl_3$, 100 MHz, TMS) δ 15.3, 17.4, 24.6, 27.6, 27.7, 32.2, 70.6, 119.2, 121.0, 122.9, 126.7, 128.0, 134.6, 142.1, 155.7. IR (neat) ν 3052, 2924, 2863, 1486, 1440, 1371, 1283, 1272, 1231, 1208, 1005, 975, 925, 767, 748 cm^{-1}. MS (%) m/e 212, 197, 183, 182, 171, 169, 158, 145, 141. HRMS (EI) calcd. for $C_{15}H_{16}O$: 212.1201, Found: 212.1202.

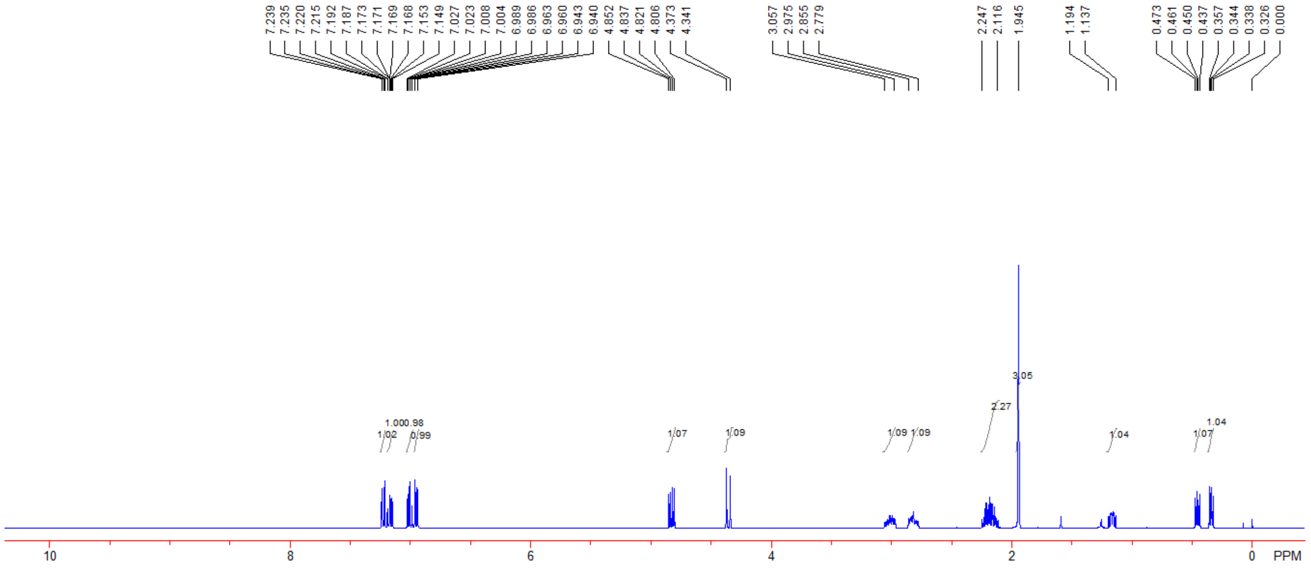

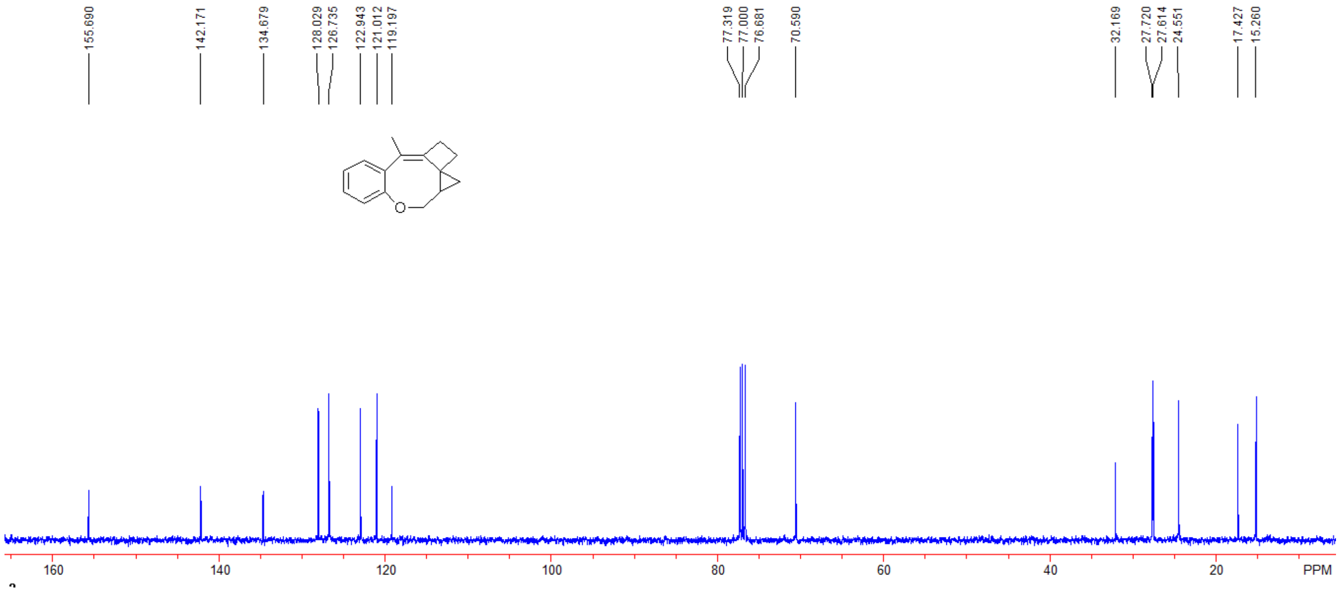

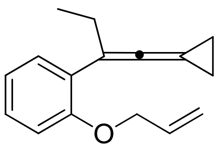

Compound **1b**. 460 mg, yield: 68%; colorless oil. ^{1}H NMR ($CDCl_3$, 400 MHz, TMS) δ 1.06 (t, J =

7.2 Hz, 3H, CH_3), 1.47-1.56 (m, 4H, CH_2), 2.52 (q, *J* = 7.2 Hz, 2H, CH_2), 4.52 (d, *J* = 5.2 Hz, 2H, CH_2), 5.22-5.25 (m, 1H, =CH_2), 5.36-5.41 (m, 1H, =CH_2), 5.98-6.07 (m, 1H, =CH), 6.84 (d, *J* = 8.0 Hz, 1H, Ar), 6.91 (t, *J* = 8.0 Hz, 1H, Ar), 7.13-7.17 (m, 1H, Ar), 7.25-7.27 (m, 1H, Ar). ^{13}C NMR ($CDCl_3$, 100 MHz, TMS) δ 7.5, 12.8, 25.8, 69.2, 76.7, 107.6, 112.7, 117.1, 120.7, 127.6, 128.5, 129.6, 133.6, 155.8, 189.4. IR (neat) ν 3056, 2965, 2930, 2010, 1736, 1595, 1578, 1486, 1445, 1283, 1241, 1122, 1016, 994, 923, 747 cm^{-1}. MS (%) m/e 226 (M^+, 0.65), 185 (100.00), 170 (23.91), 169 (29.77), 152 (20.29), 142 (29.13), 141 (23.67), 128 (21.57), 115 (27.53). HRMS (EI) calcd. for $C_{16}H_{18}O$: 226.1358, Found: 226.1355.

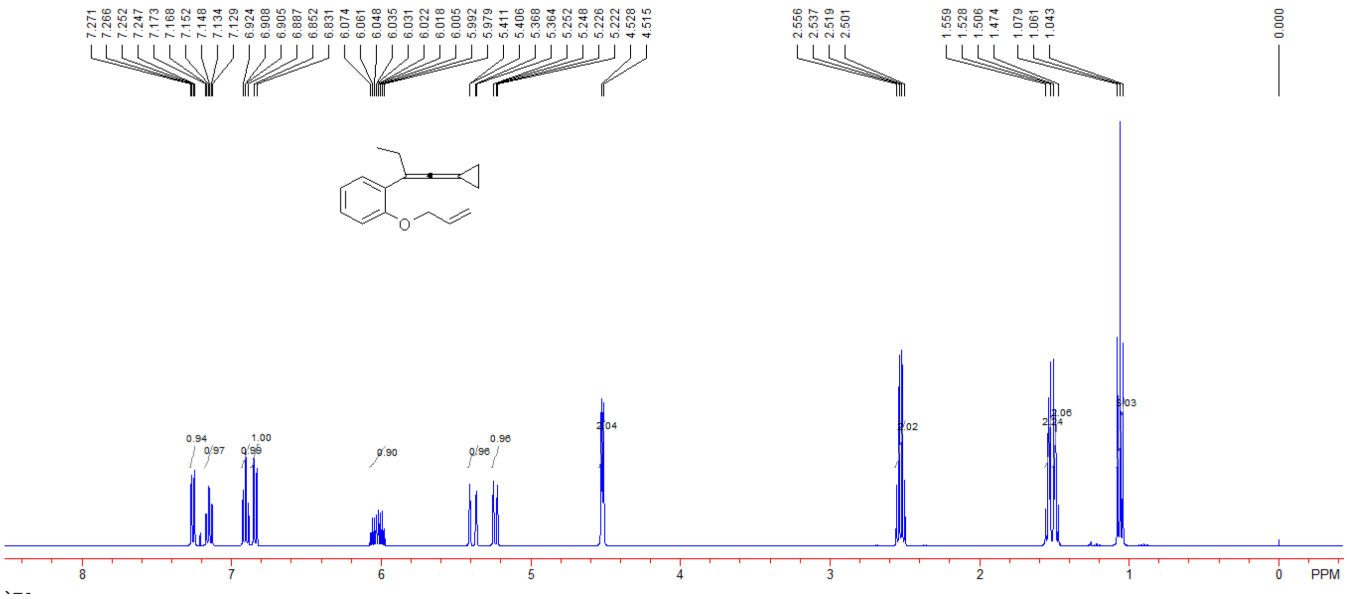

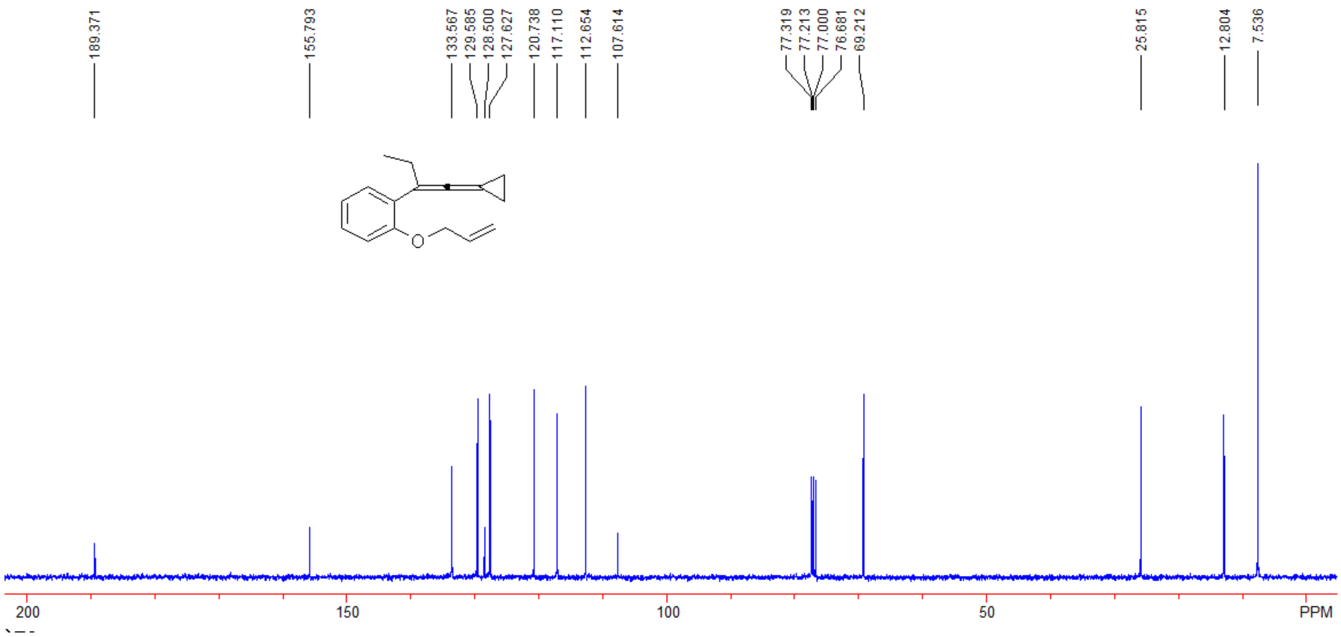

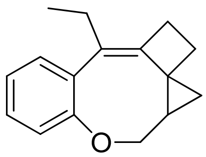

Compound **2b**. 98 mg, yield: 87%; colorless oil. ^{1}H NMR ($CDCl_3$, 400 MHz, TMS) δ 0.32 (dd, J_1 = 4.8 Hz, J_2 = 7.6 Hz, 1H, CH_2), 0.44 (dd, J_1 = 4.8 Hz, J_2 = 9.2 Hz, 1H, CH_2), 0.91 (t, J = 7.2 Hz, 3H, CH_3), 1.14-1.19 (m, 1H, CH), 2.16-2.22 (m, 2H, CH_2), 2.42 (q, J = 7.2 Hz, 2H, CH_2), 2.82-2.89 (m, 1H, CH_2), 3.00-3.08 (m, 1H, CH_2), 4.36 (d, J = 12.8 Hz, 1H, CH_2), 4.85 (dd, J_1 = 5.6 Hz, J_2 = 12.8 Hz, 1H, CH_2), 6.96-7.03 (m, 2H, Ar), 7.16-7.26 (m, 2H, Ar). ^{13}C NMR ($CDCl_3$, 100 MHz, TMS) δ 12.9, 15.1, 24.6, 24.8, 27.3, 27.9, 32.2, 70.7, 121.1, 122.8, 125.6, 126.7, 128.0, 133.2, 141.3, 156.7. IR (neat) ν 3060, 2961, 2866, 1568, 1485, 1369, 1284, 1274, 1231, 1172, 1134, 1081, 1021, 875, 799, 767 cm^{-1}. MS (%) m/e 226 (M^+, 16.00), 197 (76.11), 196 (56.65), 185 (25.05), 181 (44.68), 169 (24.62), 167 (34.82), 86 (63.89), 84 (100.00). HRMS (EI) calcd. for $C_{16}H_{18}O$: 226.1358, Found: 226.1359.

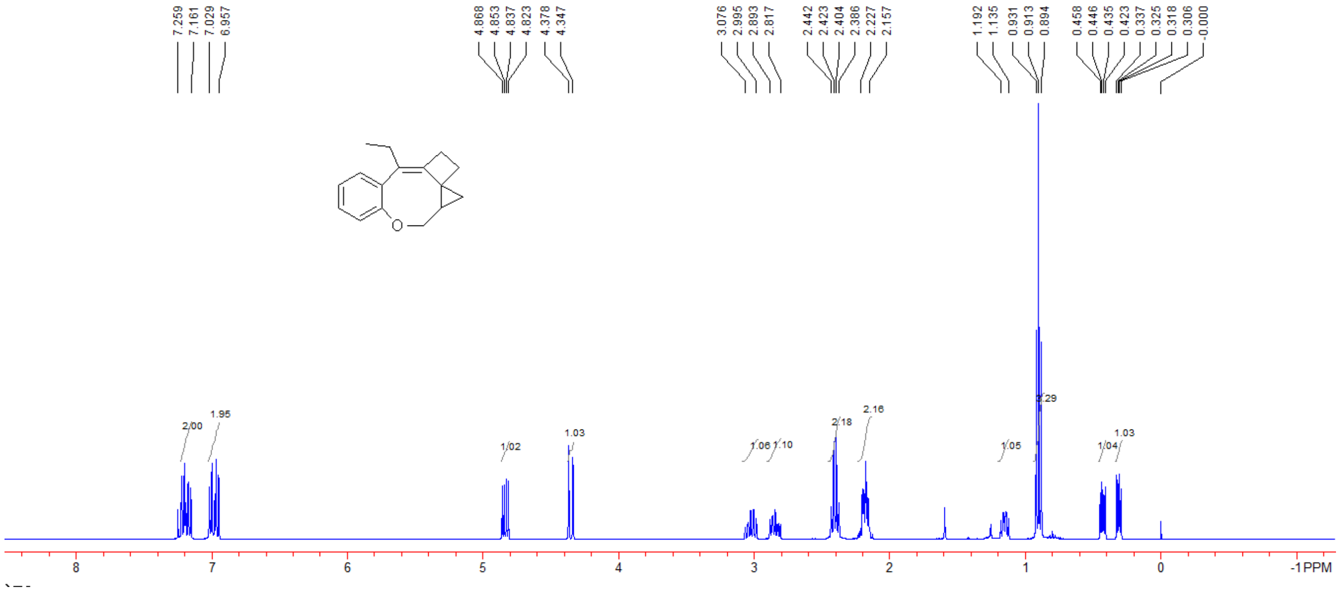

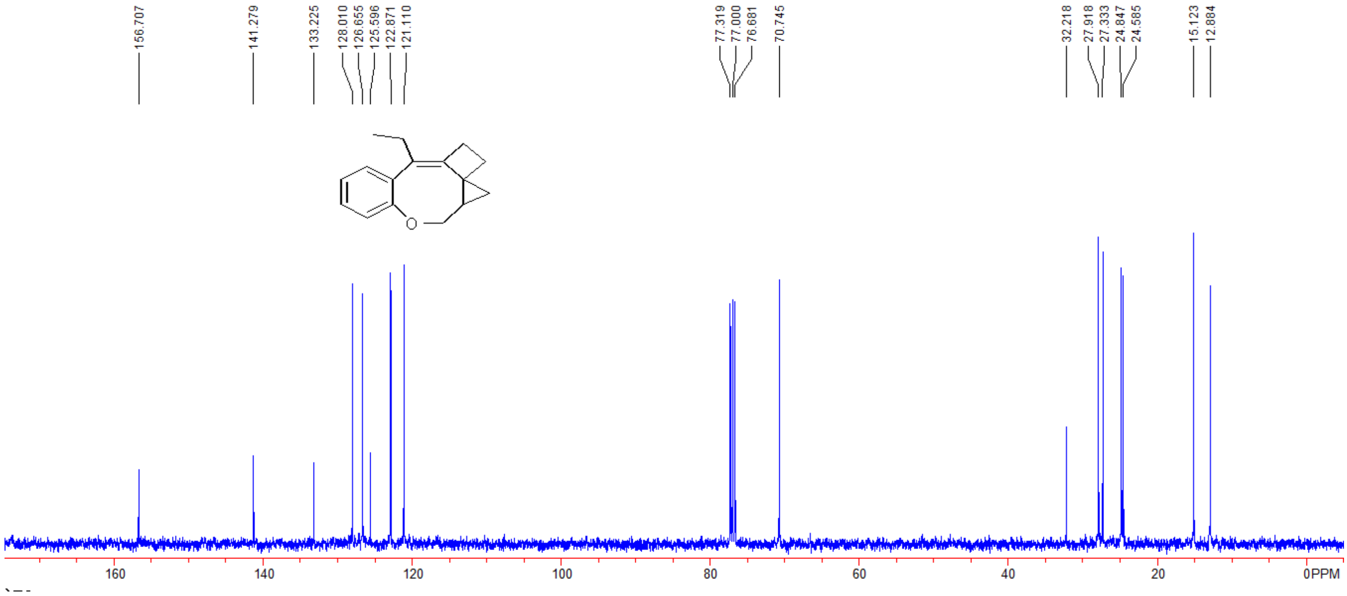

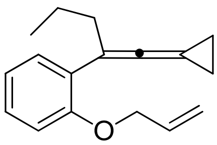

Compound **1c**. 497 mg, yield: 67%; colorless oil. ^{1}H NMR ($CDCl_3$, 400 MHz, TMS) δ 0.94 (t, J = 7.2 Hz, 3H, CH_3), 1.43-1.51 (m, 4H, CH_2), 1.52-1.55 (m, 2H, CH_2), 2.49 (q, J = 7.2 Hz, 2H, CH_2), 4.51-4.53 (m, 2H, CH_2), 5.22-5.26 (m, 1H, $=CH_2$), 5.37-5.42 (m, 1H, $=CH_2$), 5.98-6.08 (m, 1H, =CH), 6.84 (d, J = 8.8 Hz, 1H, Ar), 6.91 (t, J = 8.0 Hz, 1H, Ar), 7.13-7.17 (m, 1H, Ar), 7.26 (d, J = 9.6 Hz, 1H, Ar). ^{13}C NMR ($CDCl_3$, 100 MHz, TMS) δ 7.4, 13.8, 21.4, 34.9, 69.2, 76.5, 105.8, 112.6, 117.1, 120.7, 127.6, 128.5, 129.7, 133.5, 155.8, 189.7. IR (neat) ν 3057, 2928, 2010, 1594, 1486, 1422, 1376, 1243, 1124, 1096, 1017, 961, 925, 833, 746 cm^{-1}. MS (%) m/e 240 (M^+, 0.60), 199 (100.00), 184 (29.14), 169 (44.90), 157 (21.71), 141 (27.89), 129 (21.17), 128 (36.89), 115 (31.52). HRMS (EI) calcd. for $C_{17}H_{20}O$: 240.1514, Found: 240.1517.

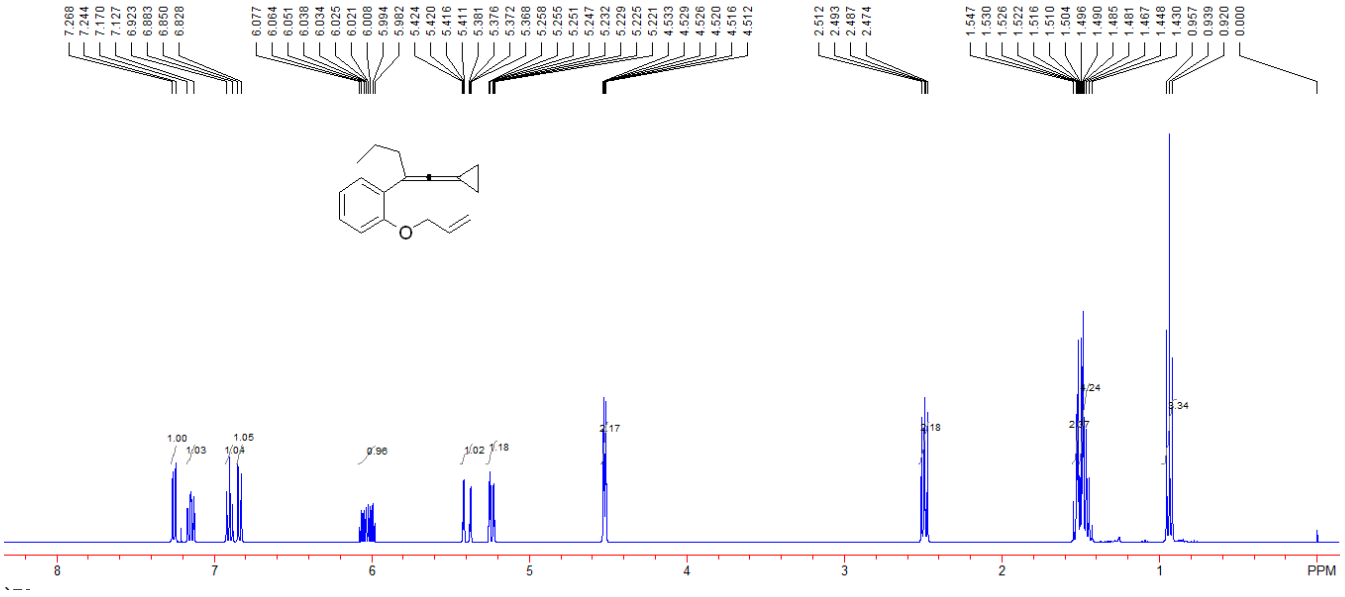

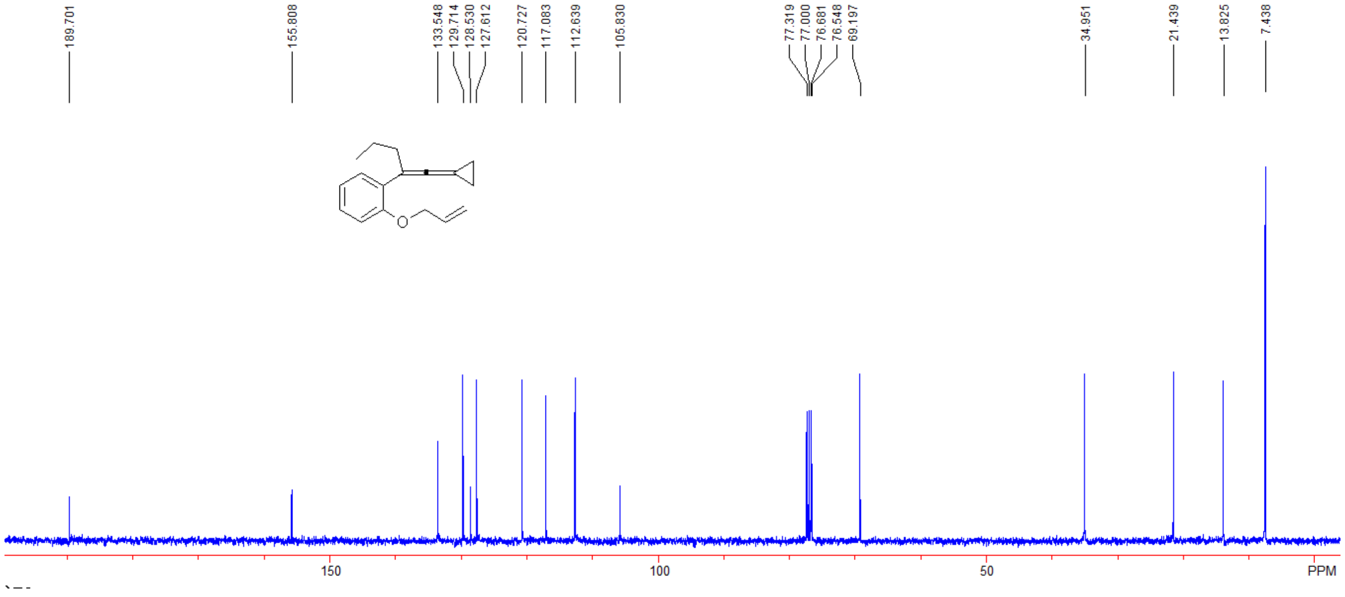

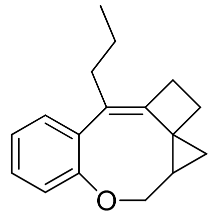

Compound **2c**. 112 mg, yield: 93%; colorless oil. ^{1}H NMR ($CDCl_3$, 400 MHz, TMS) δ 0.32 (dd, J_1 = 4.8 Hz, J_2 = 7.6 Hz, 1H, CH_2), 0.44 (dd, J_1 = 4.8 Hz, J_2 = 9.6 Hz, 1H, CH_2), 0.85 (t, J = 7.2 Hz, 3H, CH_3), 1.15-1.21 (m, 1H, CH), 1.25-1.38 (m, 2H, CH_2), 2.20 (t, J = 7.2 Hz, 2H, CH_2), 2.29-2.44 (m, 2H, CH_2), 2.83-2.91 (m, 1H, CH_2), 2.99-3.07 (m, 1H, CH_2), 4.37 (d, J = 12.8 Hz, 1H, CH_2), 4.85 (dd, J_1 = 5.6 Hz, J_2 = 12.4 Hz, 1H, CH_2), 6.97-7.03 (m, 2H, Ar), 7.17-7.25 (m, 2H, Ar). ^{13}C

NMR ($CDCl_3$, 100 MHz, TMS) δ 13.7, 15.0, 21.4, 24.9, 27.5, 27.9, 32.1, 33.3, 70.6, 121.1, 122.8, 124.1, 126.7, 128.0, 133.5, 141.8, 156.6. IR (neat) ν 3063, 2929, 1596, 1486, 1371, 1273, 1207, 1134, 1083, 979, 929, 863, 768, 673, 656 cm^{-1}. MS (%) m/e 240 (M^+, 20.02), 210 (52.06), 199 (26.87), 197 (84.27), 195 (37.36), 182 (33.15), 181 (100.00), 169 (32.40), 115 (29.63). HRMS (EI) calcd. for $C_{17}H_{20}O$: 240.1514, Found: 240.1511.

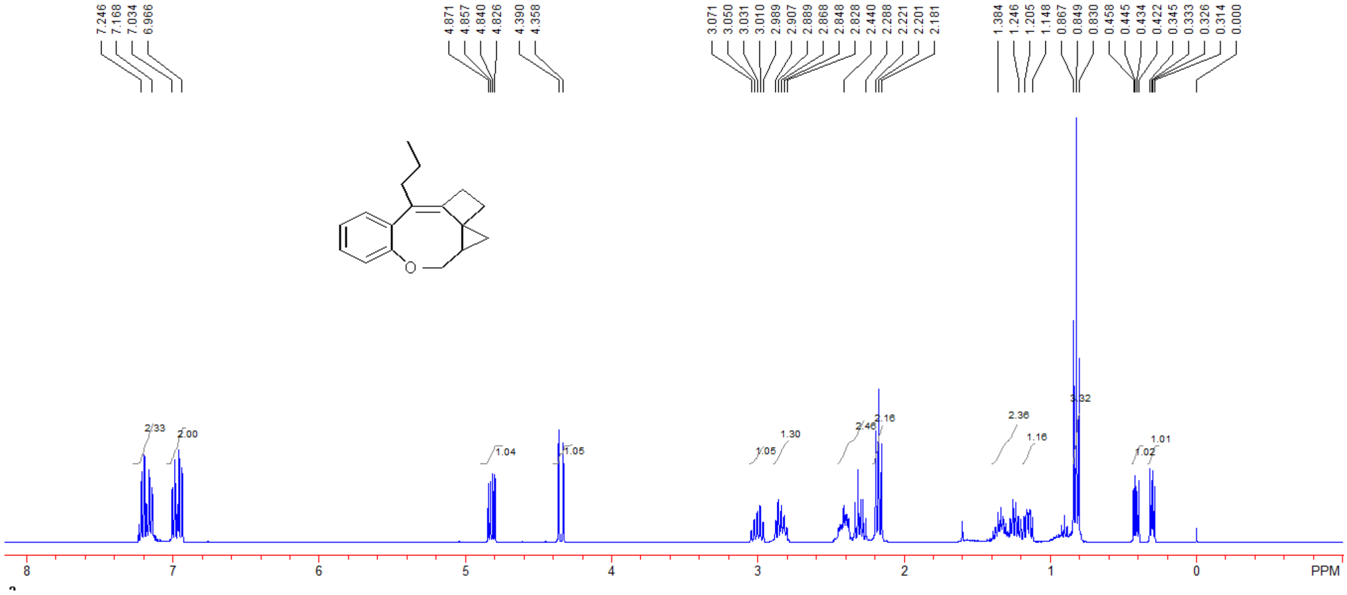

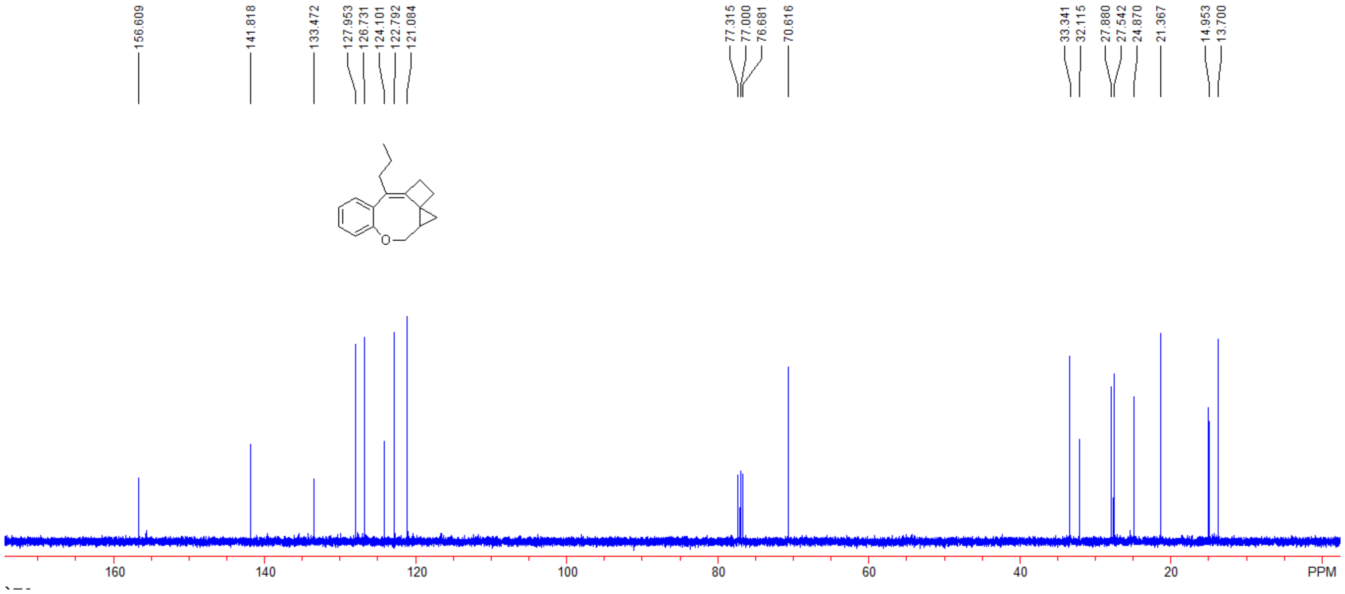

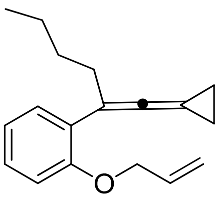

Compound **1d**. 610 mg, yield: 80%; colorless oil. ^{1}H NMR ($CDCl_3$, 400 MHz, TMS) δ 0.88 (t, *J* =

7.6 Hz, 3H, CH_3), 1.34-1.50 (m, 6H, CH_2), 1.52-1.55 (m, 2H, CH_2), 2.52 (t, J = 7.6 Hz, 2H, CH_2), 4.52-4.53 (m, 2H, CH_2), 5.24 (dd, J_1 = 1.2 Hz, J_2 = 10.4 Hz, 1H, $=CH_2$), 5.39 (dd, J_1 = 1.2 Hz, J_2 = 17.2 Hz, 1H, $=CH_2$), 5.98-6.08 (m, 1H, =CH), 6.84 (d, J = 8.0 Hz, 1H, Ar), 6.88-6.92 (m, 1H, Ar), 7.13-7.17 (m, 1H, Ar), 7.24-7.26 (m, 1H, Ar). ^{13}C NMR ($CDCl_3$, 100 MHz, TMS) δ 7.5, 14.0, 22.1, 30.4, 32.5, 69.2, 76.6, 106.0, 112.7, 117.1, 120.7, 127.6, 128.6, 129.7, 133.6, 155.8, 189.6. IR (neat) ν 3056, 2858, 2010, 1594, 1486, 1422, 1283, 1243, 1043, 1017, 995, 924, 833, 750 cm^{-1}. MS (%) m/e 254 (M^+, 0.40), 213 (79.84), 183 (18.06), 171 (100.00), 169 (30.04), 141 (18.88), 131 (15.18), 128 (40.13), 115 (23.45). HRMS (EI) calcd. for $C_{18}H_{22}O$: 254.1671, Found: 254.1666.

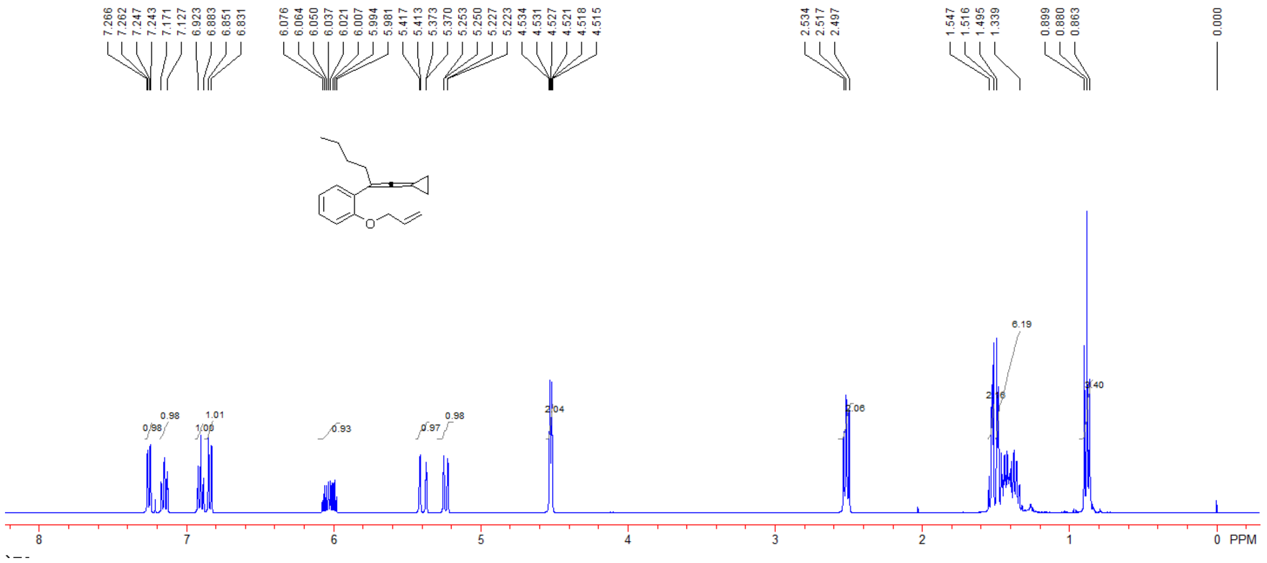

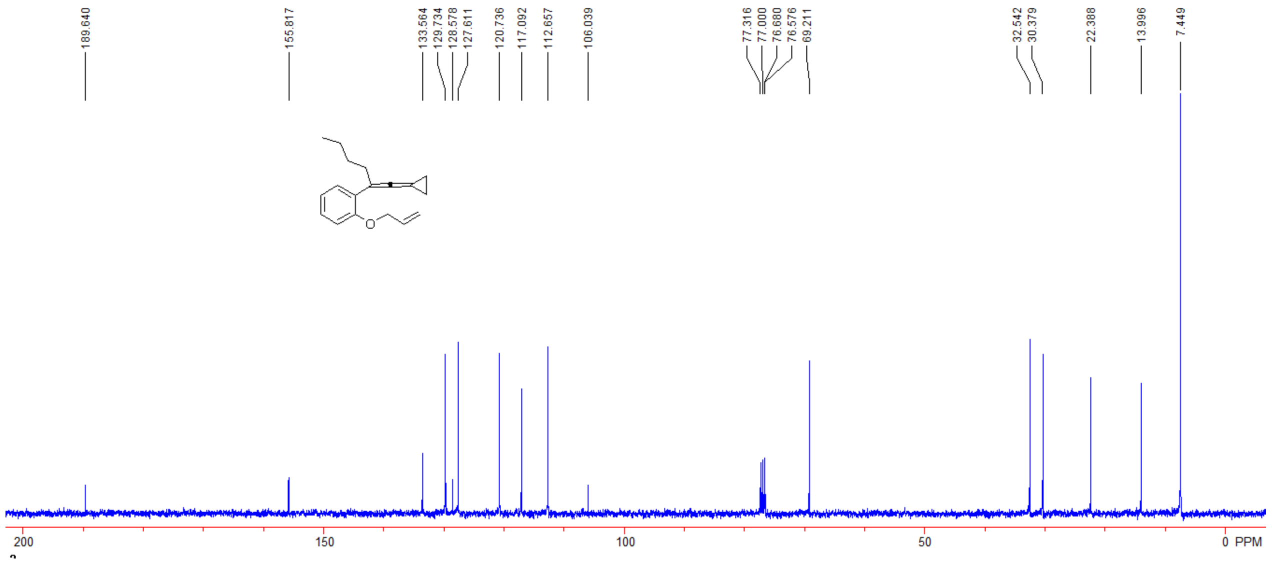

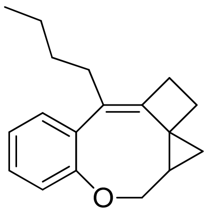

Compound **2d**. 119 mg, yield: 94%; colorless oil. ^{1}H NMR ($CDCl_3$, 400 MHz, TMS) δ 0.30 (dd, J_1 = 4.8 Hz, J_2 = 7.6 Hz, 1H, CH_2), 0.42 (dd, J_1 = 4.8 Hz, J_2 = 9.6 Hz, 1H, CH_2), 0.82 (t, J = 6.8 Hz, 3H, CH_3), 1.12-1.18 (m, 1H, CH), 1.19-1.30 (m, 4H, CH_2), 2.18 (t, J = 8.4 Hz, 2H, CH_2), 2.35-2.40 (m, 2H, CH_2), 2.81-2.88 (m, 1H, CH_2), 2.81-3.05 (m, 1H, CH_2), 4.36 (d, J = 12.0 Hz, 1H, CH_2), 4.83 (dd, J_1 = 6.0 Hz, J_2 = 12.4 Hz, 1H, CH_2), 6.94-7.02 (m, 2H, Ar), 7.15-7.23 (m, 2H, Ar). ^{13}C NMR ($CDCl_3$, 100 MHz, TMS) δ 14.0, 15.0, 22.4, 24.9, 27.5, 27.9, 30.4, 31.1, 32.2, 70.7, 121.1, 122.8, 124.3, 126.7, 128.0, 133.5, 141.6, 156.6. IR (neat) ν 3060, 2928, 2857, 1594, 1486, 1440, 1371, 1282, 1209, 1134, 1053, 1016, 938, 768, 737 cm^{-1}. MS (%) m/e 254 (M^+, 24.50), 225 (35.26), 224 (60.46), 197 (100.00), 195 (67.56), 182 (44.90), 181 (58.17), 169 (31.11), 145 (26.67). HRMS (EI) calcd. for $C_{18}H_{22}O$: 254.1671, Found: 254.1669.

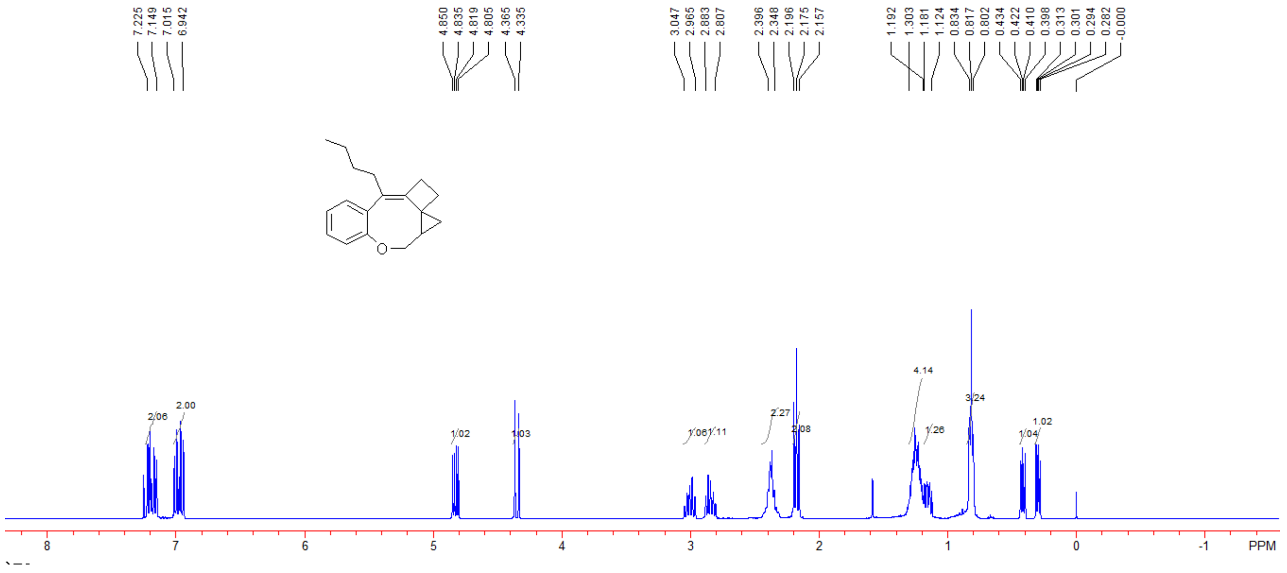

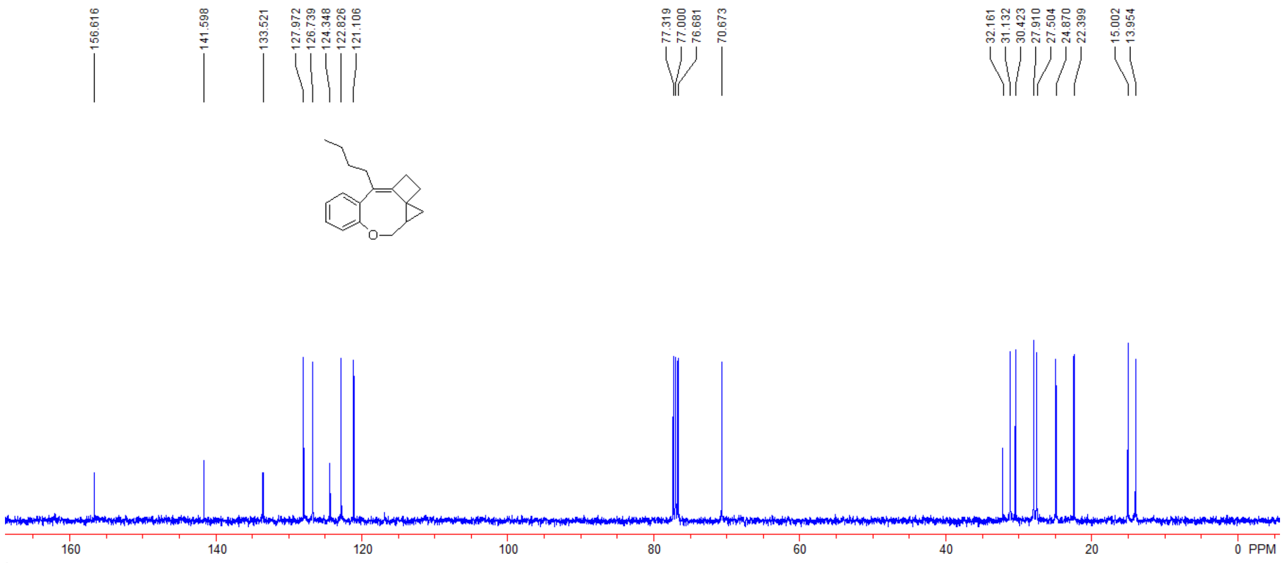

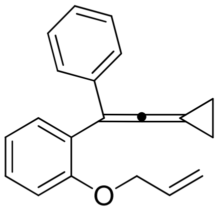

Compound **1e**. 765 mg, yield: 93%; colorless oil. ^{1}H NMR (CDCl$_3$, 400 MHz, TMS) δ 1.62-1.67 (m, 2H, CH$_2$), 1.68-1.74 (m, 2H, CH$_2$), 4.43 (d, *J* = 4.8 Hz, 2H, CH$_2$), 5.03-5.10 (m, 2H, =CH$_2$), 5.69-5.77 (m, 1H, =CH), 6.90 (d, *J* = 8.0 Hz, 1H, Ar), 6.98 (t, *J* = 8.0 Hz, 1H, Ar), 7.12-7.16 (m, 1H, Ar), 7.21-7.29 (m, 5H, Ar), 7.33 (d, *J* = 8.0 Hz, 1H, Ar). ^{13}C NMR (CDCl$_3$, 100 MHz, TMS) δ 8.7, 70.0, 78.4, 108.0, 113.0, 116.5, 120.9, 126.0, 126.7, 126.9, 127.9, 128.6, 131.7, 133.1, 138.3, 156.2, 190.2. IR (neat) ν 3052, 2983, 2919, 2002, 1595, 1490, 1443, 1409, 1221, 1160, 1045, 1015, 923, 827, 749 cm^{-1}. MS (%) m/e 274 (M$^+$, 0.65), 233 (89.33), 231 (41.11), 218 (74.66), 215 (100.00), 205 (62.60), 203 (42.49), 202 (53.26), 189 (48.32). HRMS (EI) calcd. for $C_{20}H_{18}O$: 274.1358, Found: 274.1359.

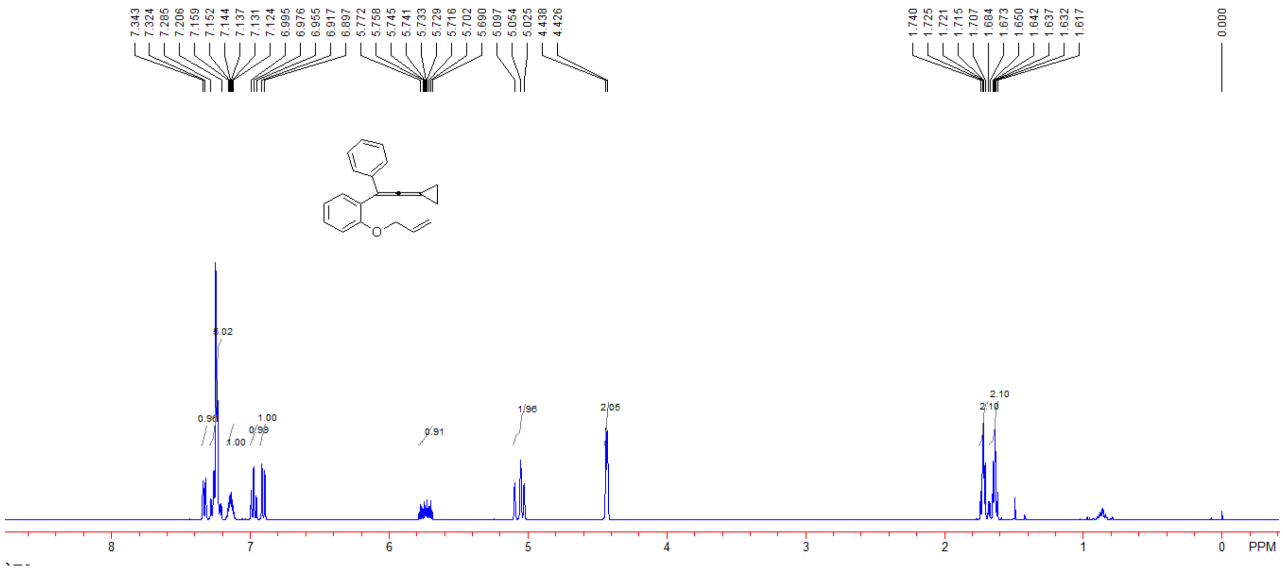

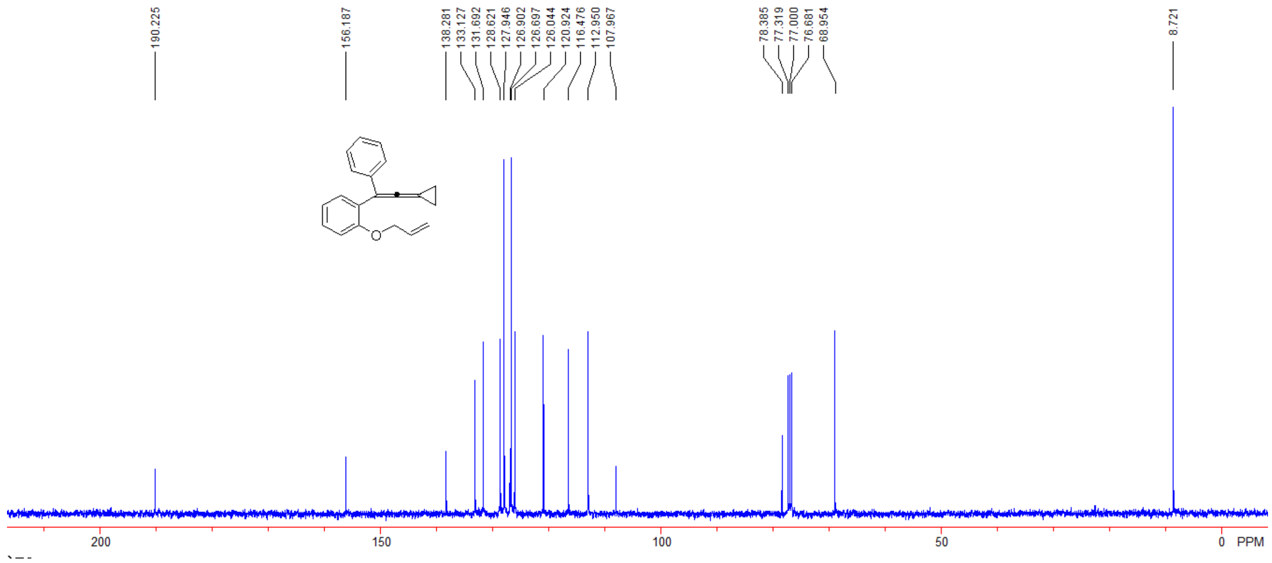

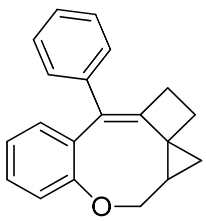

Compound **2e**. 70 mg, yield: 51%; colorless oil. ^{1}H NMR ($CDCl_3$, 400 MHz, TMS) δ 0.45-0.48 (m, 1H, CH_2), 0.60-0.63 (m, 1H, CH_2), 1.21-1.27 (m, 1H, CH), 2.19-2.33 (m, 2H, CH_2), 2.92-3.01 (m, 1H, CH_2), 3.23-3.31 (m, 1H, CH_2), 4.42 (d, J = 12.0 Hz, 1H, CH_2), 4.87 (dd, J_1 = 6.0 Hz, J_2 = 12.0 Hz, 1H, CH_2), 6.86-6.93 (m, 2H, Ar), 7.03 (d, J = 8.4 Hz, 1H, Ar), 7.17-7.25 (m, 4H, Ar), 7.27-7.31 (m, 2H, Ar). ^{13}C NMR ($CDCl_3$, 100 MHz, TMS) δ 15.5, 26.3, 28.3, 28.5, 33.0, 70.3, 120.8, 122.9, 126.1, 127.9, 128.6, 129.3, 129.9, 130.1, 134.2, 141.3, 145.3, 156.6, 173.1. IR (neat) ν 3052, 3020,

2866, 1594, 1486, 1441, 1278, 1236, 1213, 1108, 794, 764, 751, 699, 668 cm^{-1}. MS (%) m/e 274 (M^+, 3.85), 233 (50.47), 218 (39.14), 215 (53.22), 205 (37.21), 202 (30.34), 189 (26.29), 86 (61.36), 84 (100.00). HRMS (EI) calcd. for $C_{20}H_{18}O$: 274.1358, Found: 274.1362; Enantiomeric excess was determined by HPLC with a Chiralcel AD-H column [λ = 214 nm; eluent: Hexane/Isopropanol = 99/1; Flow rate: 0.60 mL/min; t_{minor} = 20.44 min, t_{major} = 16.79 min; ee% = 87%; $[\alpha]^{20}_{D}$ = -0.7960 (c 1.20, CH_2Cl_2)].

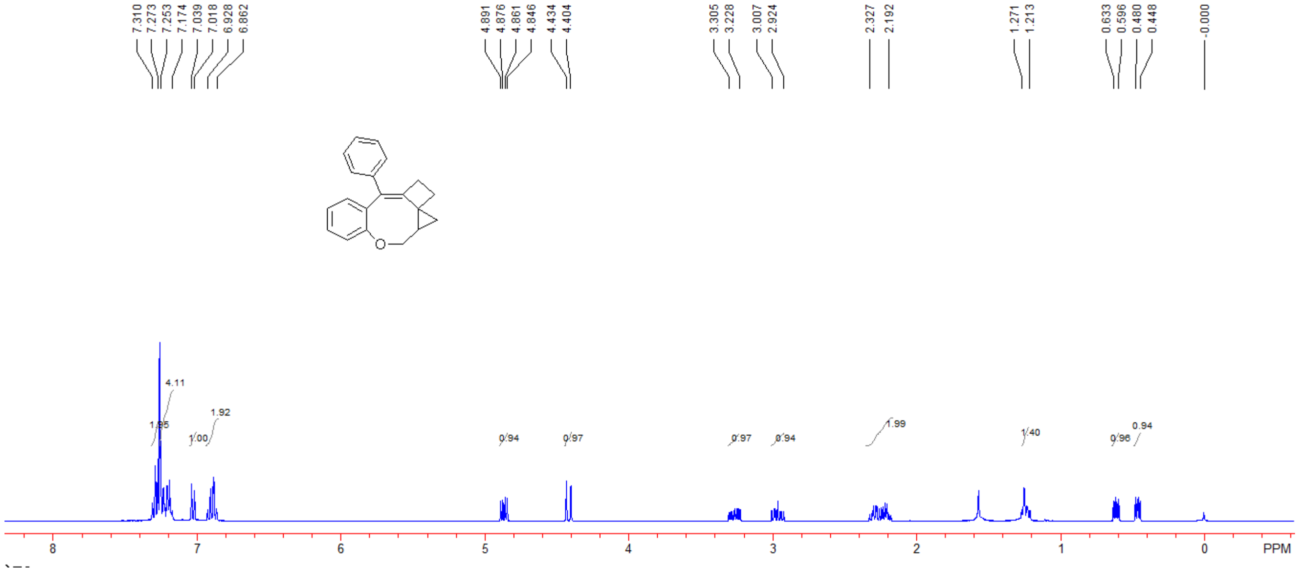

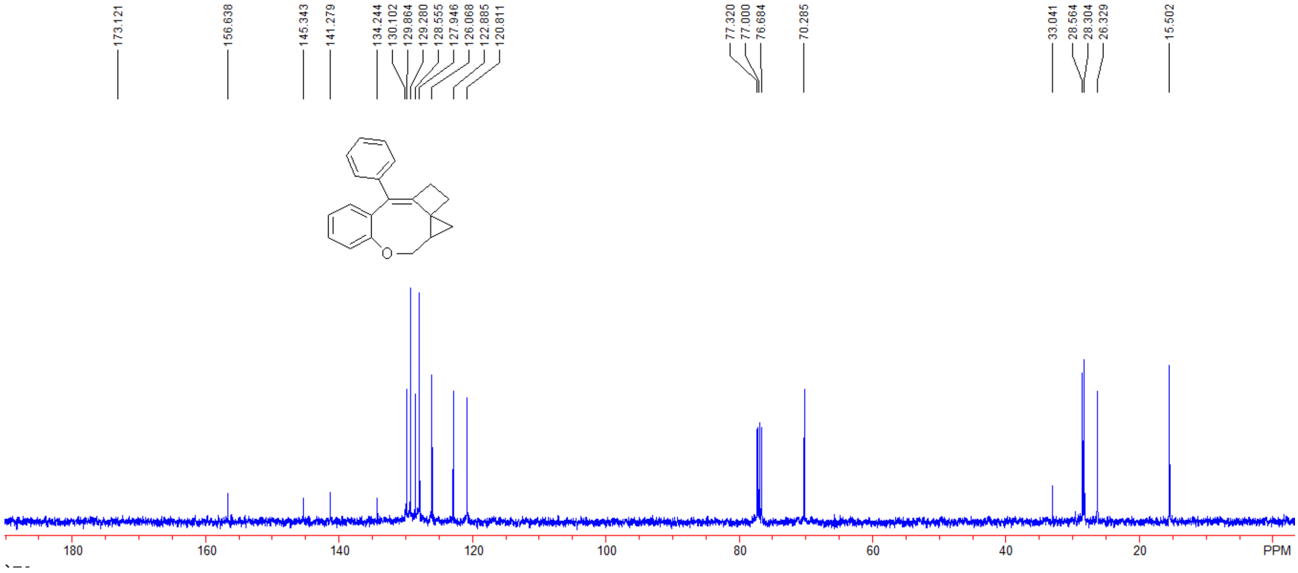

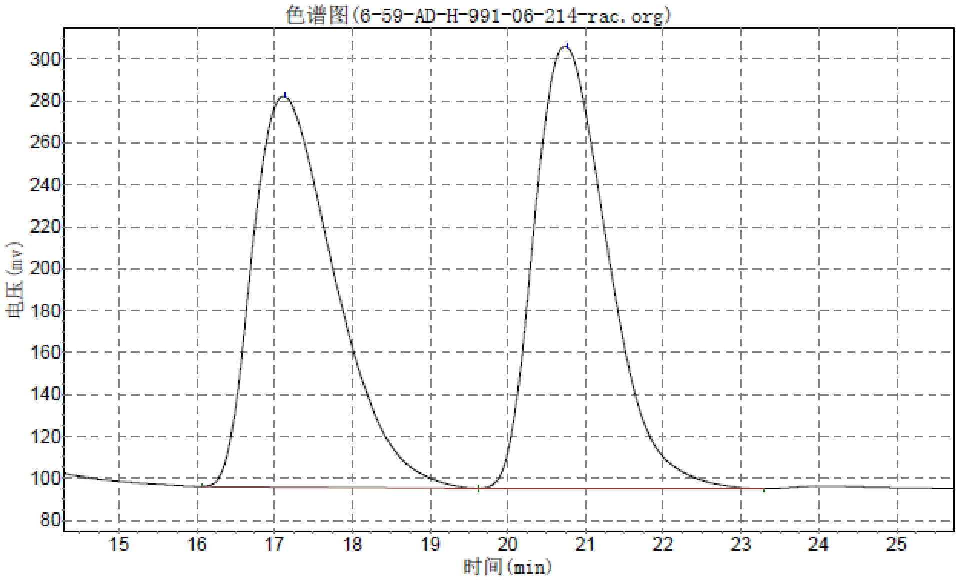

分析结果表

峰号	峰名	保留时间	峰高	峰面积	含量
1		17.132	189080.219	14290012.000	50.2539
2		20.765	212104.031	14145642.000	49.7461
总计			401184.250	28435654.000	100.0000

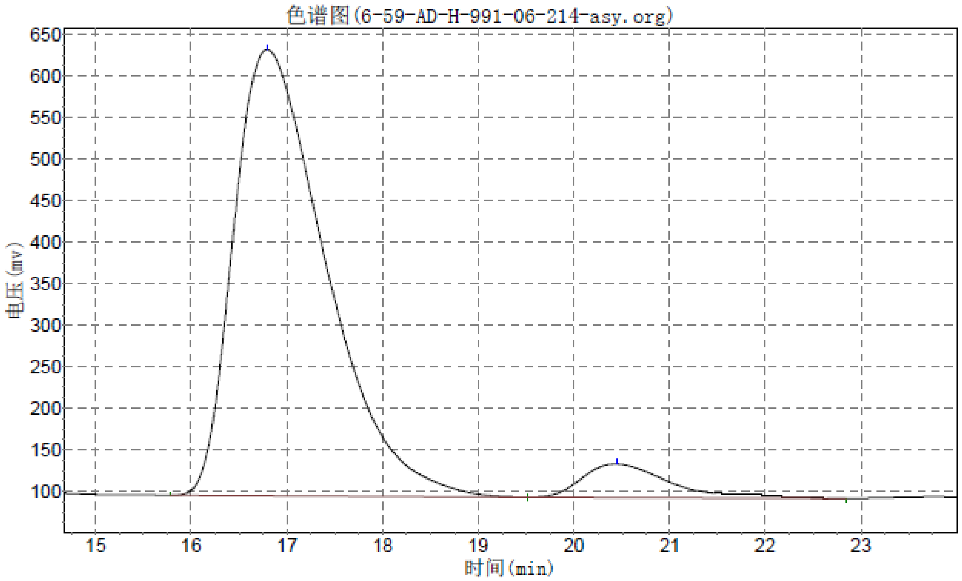

分析结果表

峰号	峰名	保留时间	峰高	峰面积	含量
1		16.798	537369.125	36002816.000	93.4100
2		20.440	40477.188	2539983.250	6.5900
总计			577846.313	38542799.250	100.0000

Translation: a Chiralcel AD-H column [λ = 214 nm; eluent: Hexane/Isopropanol = 99/1; Flow rate: 0.60 mL/min; t_{minor} = 20.44 min, t_{major} = 16.79 min; ee% = 87%]

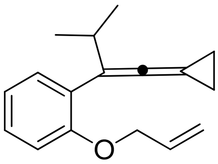

Compound **1f**. 384 mg, yield: 53%; yellow oil. ^{1}H NMR ($CDCl_3$, 400 MHz, TMS) δ 1.05 (d, J = 6.8 Hz, 6H, CH_3), 1.46-1.49 (m, 2H, CH_2), 1.52-1.55 (m, 2H, CH_2), 2.91 (hept, J = 6.8 Hz, 1H, CH), 4.54 (d, J = 5.2 Hz, 2H, CH_2), 5.23-5.25 (m, 1H, =CH_2), 5.38-5.43 (m, 1H, =CH_2), 5.99-6.09 (m, 1H, =CH), 6.85 (d, J = 8.0 Hz, 1H, Ar), 6.88-6.92 (m, 1H, Ar), 7.14-7.19 (m, 1H, Ar), 7.22-7.25 (m, 1H, Ar). ^{13}C NMR ($CDCl_3$, 100 MHz, TMS) δ 7.5, 22.0, 30.4, 69.2, 78.0, 112.7, 113.3, 116.9, 120.7, 127.7, 128.8, 130.5, 133.6, 155.8, 188.2. IR (neat) ν 3052, 2959, 2866, 2012, 1593, 1495, 1422, 1409, 1360, 1161, 1043, 997, 959, 923, 747 cm^{-1}. MS (%) m/e 240 (M^+, 0.26), 200 (24.33), 199 (100.00), 184 (29.74), 183 (23.77), 169 (37.11), 128 (25.34), 115 (25.25), 43 (21.20). HRMS (EI) calcd. for $C_{17}H_{20}O$: 240.1514, Found: 240.1515.

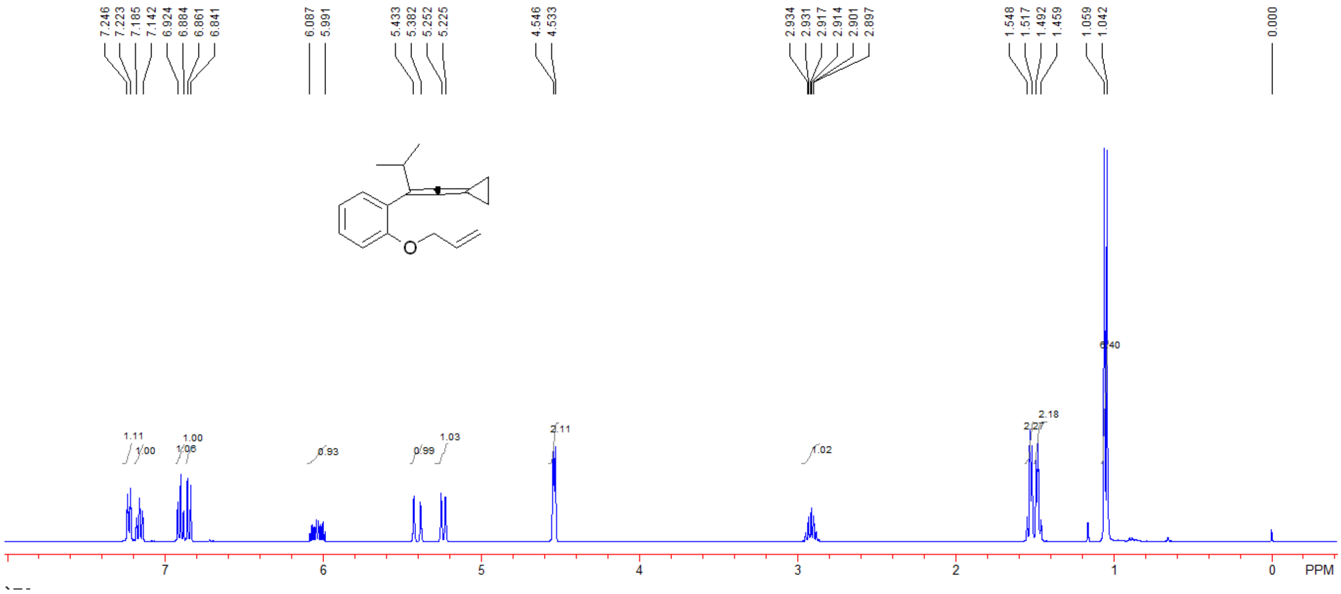

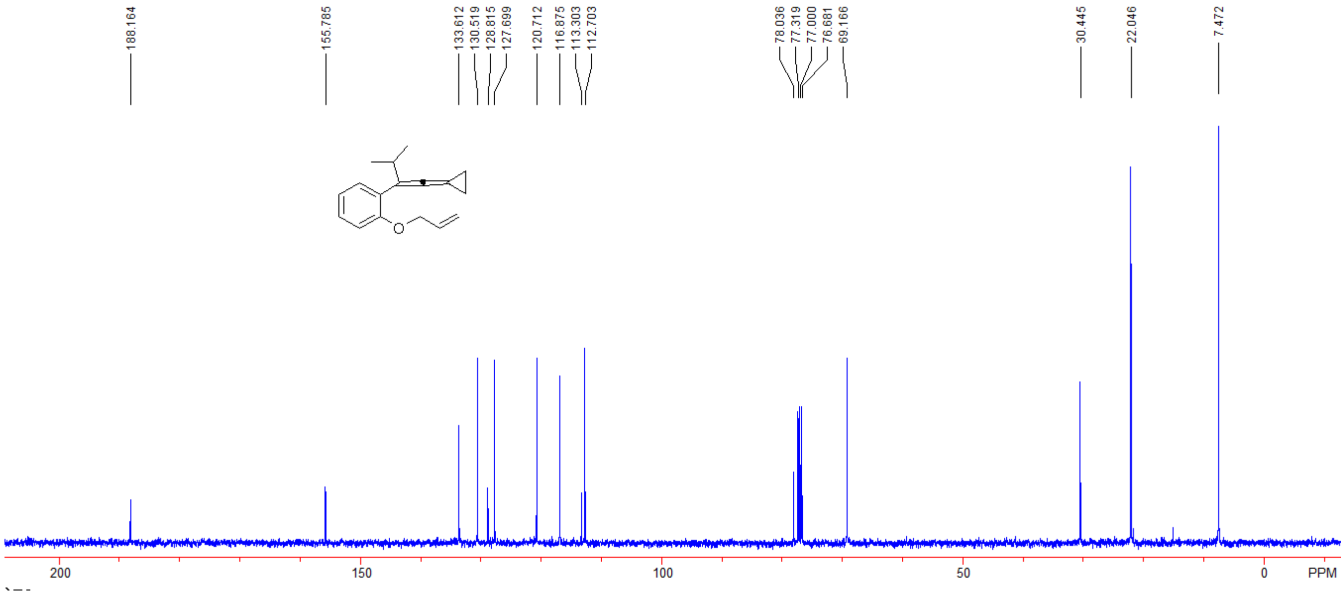

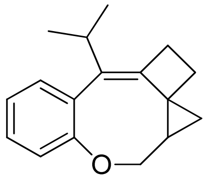

Compound **2f**. 102 mg, yield: 85%; colorless oil. ^{1}H NMR ($CDCl_3$, 400 MHz, TMS) δ 0.18 (dd, J_1 = 4.8 Hz, J_2 = 7.6 Hz, 1H, CH_2), 0.37 (dd, J_1 = 4.8 Hz, J_2 = 9.6 Hz, 1H, CH_2), 1.00 (d, J = 7.2 Hz, 3H, CH_3), 1.05-1.11 (m, 1H, CH), 1.25 (d, J = 7.2 Hz, 3H, CH_3), 2.07-2.13 (m, 1H, CH_2), 2.19-2.26 (m, 1H, CH_2), 2.79 (hept, J = 7.2 Hz, 1H, CH), 2.83-2.91 (m, 1H, CH_2), 3.08-3.16 (m, 1H, CH_2), 4.38 (d, J = 12.4 Hz, 1H, CH_2), 4.80 (dd, J_1 = 5.6 Hz, J_2 = 12.4 Hz, 1H, CH_2), 6.95-6.98 (m, 2H, Ar), 7.16-7.20 (m, 1H, Ar), 7.27-7.30 (m, 1H, Ar). ^{13}C NMR ($CDCl_3$, 100 MHz, TMS) δ 14.3, 20.9, 22.6, 25.6, 27.8, 28.4, 32.1, 32.4, 70.9, 121.1, 122.4, 127.6, 128.0, 128.9, 133.6, 141.3, 156.5. IR (neat) ν 3060, 2928, 2866, 1566, 1485, 1440, 1360, 1208, 1132, 1088, 1040, 987, 860, 825, 797, 750 cm^{-1}. MS (%) m/e 240 (M^+, 9.18), 210 (25.59), 197 (100.00), 195 (31.71), 181 (28.76), 169 (26.22), 167 (20.22), 141 (20.43), 115 (21.28). HRMS (EI) calcd. for $C_{17}H_{20}O$: 240.1514, Found: 240.1518.

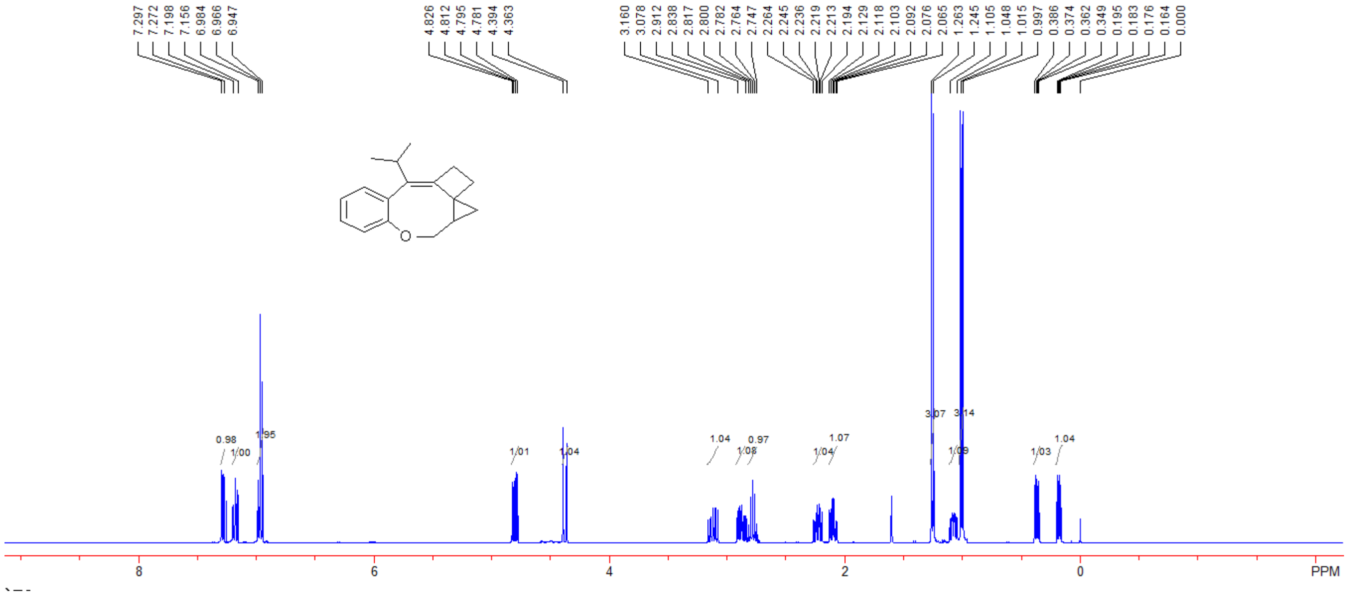

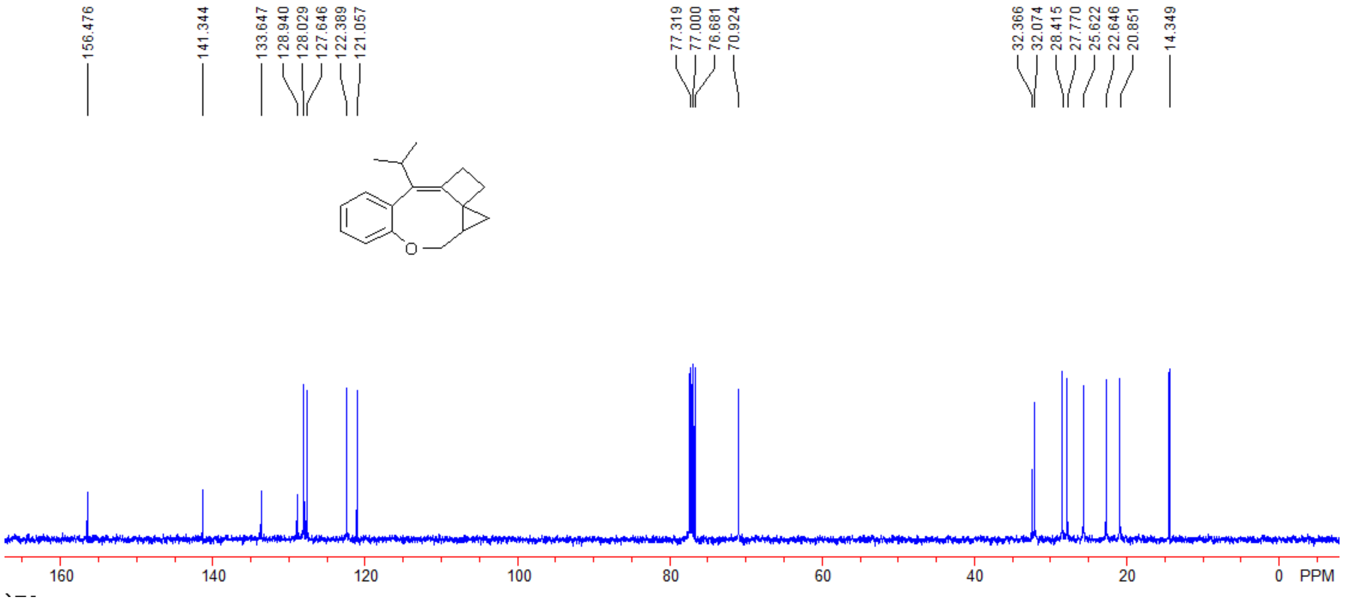

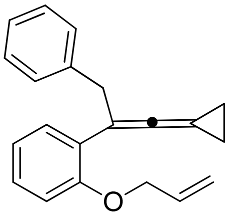

Compound **1g**. 575 mg, yield: 66%; colorless oil. ^{1}H NMR ($CDCl_3$, 400 MHz, TMS) δ 1.38-1.48 (m, 4H, CH_2), 3.88 (s, 2H, CH_2), 4.52 (d, *J* = 5.2 Hz, 2H, CH_2), 5.23-5.26 (m, 1H, $=CH_2$), 5.38-5.43 (m, 1H, $=CH_2$), 6.00-6.07 (m, 1H, =CH), 6.81-6.86 (m, 2H, Ar), 7.10-7.13 (m, 2H, Ar), 7.17-7.24 (m, 5H, Ar). ^{13}C NMR ($CDCl_3$, 100 MHz, TMS) δ 7.7, 39.6, 69.2, 76.8, 105.1, 112.6, 117.3, 120.7, 125.7, 127.8, 127.9, 128.9, 129.9, 133.5, 140.4, 155.8, 191.3. IR (neat) ν 3060, 2956, 2011, 1738,

1594, 1445, 1408, 1284, 1243, 1113, 1017, 925, 805, 746, 697 cm^{-1}. MS (%) m/e 288 (M^+, 1.20), 247 (23.59), 205 (20.13), 92 (19.59), 91 (93.62), 71 (100.00), 57 (26.00), 43 (58.95), 41 (29.54). HRMS (EI) calcd. for $C_{21}H_{20}O$: 288.1514, Found: 288.1516.

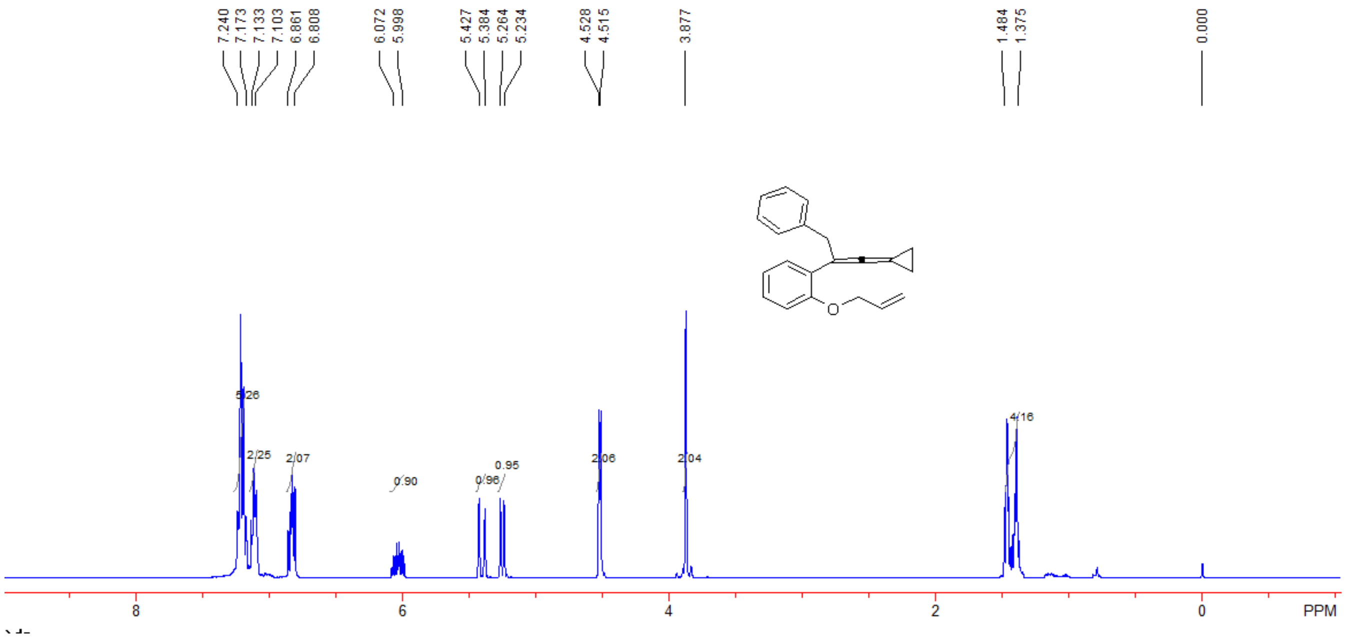

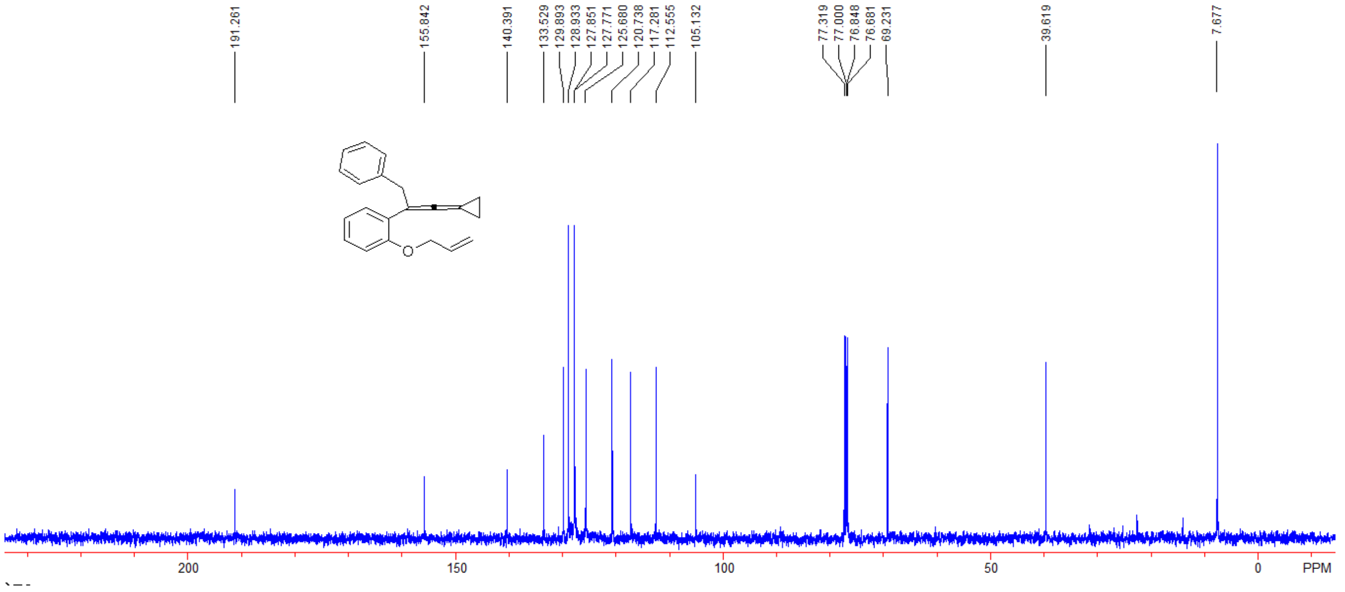

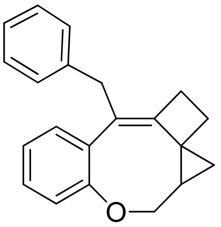

Compound **2g**. 124 mg, yield: 86%; colorless oil. ^{1}H NMR ($CDCl_3$, 400 MHz, TMS) δ 0.40 (dd, J_1

= 4.8 Hz, J_2 = 7.6 Hz, 1H, CH_2), 0.50 (dd, J_1 = 4.8, J_2 = 10.0, 1H, CH_2), 1.19-1.26 (m, 1H, CH), 2.22 (t, J = 8.0 Hz, 2H, CH_2), 2.83-2.91 (m, 1H, CH_2), 2.98-3.07 (m, 1H, CH_2), 3.62-3.81 (m, 2H, CH_2), 4.36 (d, J = 12.4 Hz, 1H, CH_2), 4.85 (dd, J_1 = 6.0 Hz, J_2 = 12.4 Hz, 1H, CH_2), 6.88-6.92 (m, 2H, Ar), 7.08-7.24 (m, 7H, Ar). ^{13}C NMR ($CDCl_3$, 100 MHz, TMS) δ 15.3, 25.1, 27.80, 27.83, 32.2, 37.5, 70.5, 121.0, 121.8, 122.9, 125.6, 126.7, 127.9, 128.2, 128.9, 133.6, 140.3, 144.9, 156.4. IR (neat) ν 3059, 2922, 2866, 1598, 1486, 1440, 1283, 1210, 1079, 1008, 987, 907, 863, 828, 768, 728 cm^{-1}. MS (%) m/e 288 (M^+, 1.08), 107 (8.96), 93 (9.63), 88 (10.96), 86 (63.20), 84 (100.00), 57 (13.37), 49 (13.43), 47 (16.04). HRMS (EI) calcd. for $C_{21}H_{20}O$: 288.1514, Found: 288.1509.

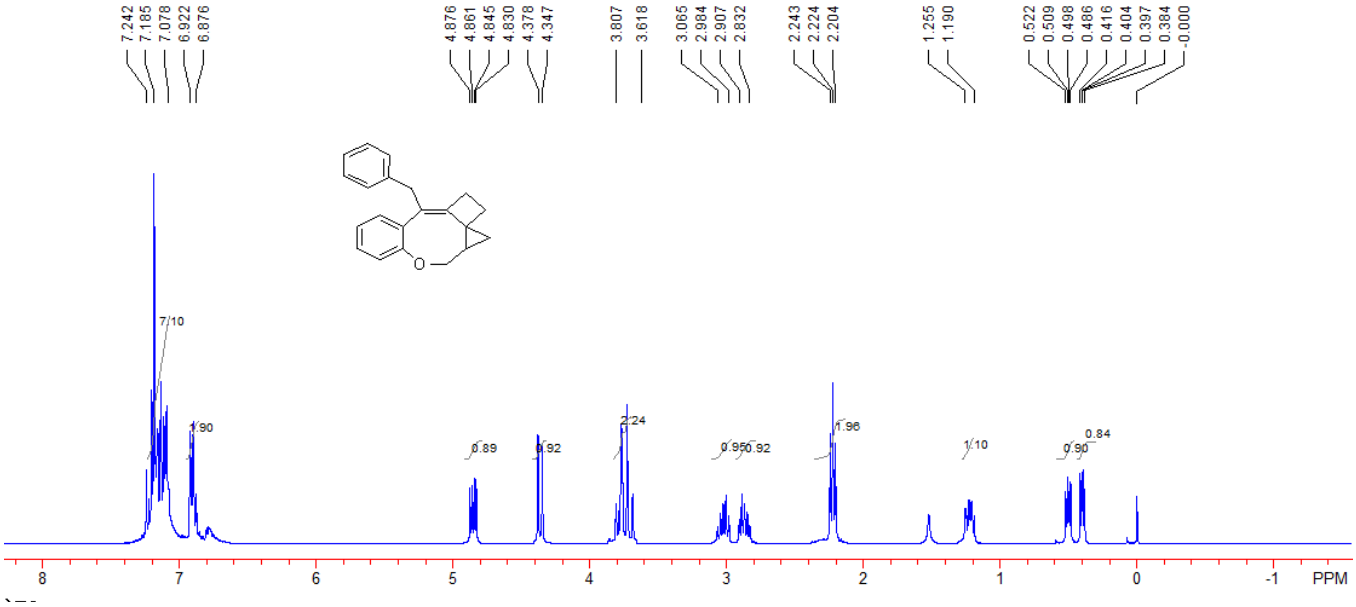

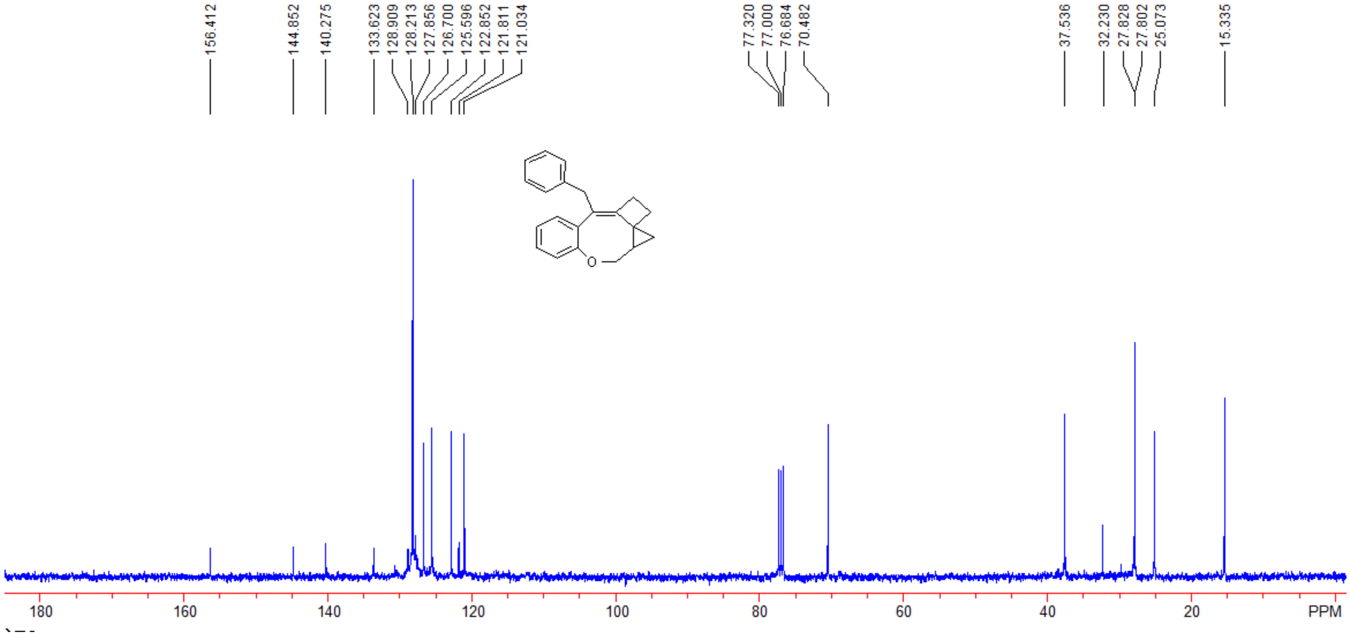

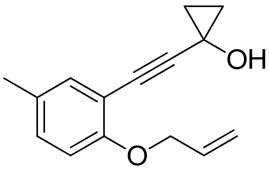

Compound **S3h**. 3.648 g, yield: 73%; yellow oil. ^{1}H NMR ($CDCl_3$, 400 MHz, TMS) δ 1.09-1.12 (m, 2H, CH_2), 1.15-1.19 (m, 2H, CH_2), 2.23 (s, 3H, CH_3), 2.94 (br, 1H, OH), 4.55-4.57 (m, 2H, CH_2), 5.25-5.29 (m, 1H, =CH_2), 5.43-5.48 (m, 1H, =CH_2), 6.00-6.10 (m, 1H, =CH), 6.73-6.75 (m, 1H, Ar), 7.01-7.03 (m, 1H, Ar), 7.18-7.19 (m, 1H, Ar). ^{13}C NMR ($CDCl_3$, 100 MHz, TMS) δ 17.6, 20.2, 46.1, 69.4, 78.6, 94.7, 112.3, 112.5, 117.1, 129.9, 133.2, 133.8, 156.9. IR (neat) ν 3372, 3010, 2188, 1647, 1605, 1454, 1379, 1282, 1158, 1020, 995, 923, 878, 804, 737 cm^{-1}. MS (%) m/e 228 (M^+, 7.62), 159 (61.64), 145 (97.03), 129 (65.31), 128 (59.12), 115 (97.80), 91 (61.29), 77 (70.90), 55 (100.00). HRMS (EI) calcd. for $C_{15}H_{16}O_2$: 228.1150, Found: 228.1151.

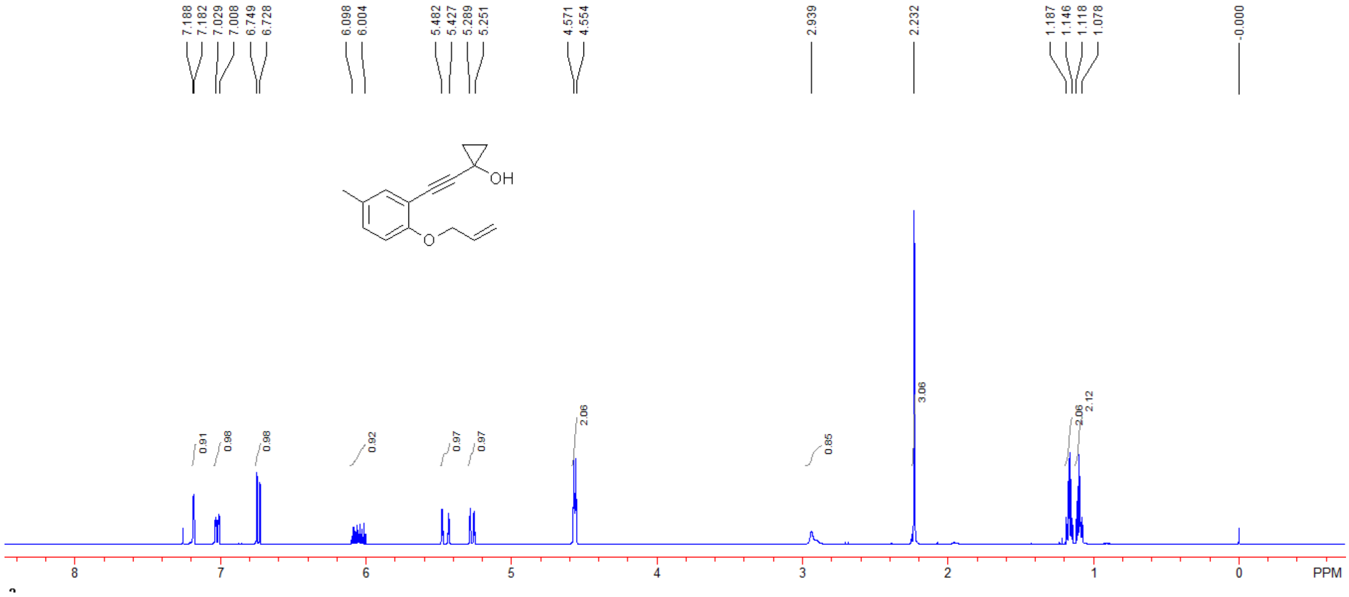

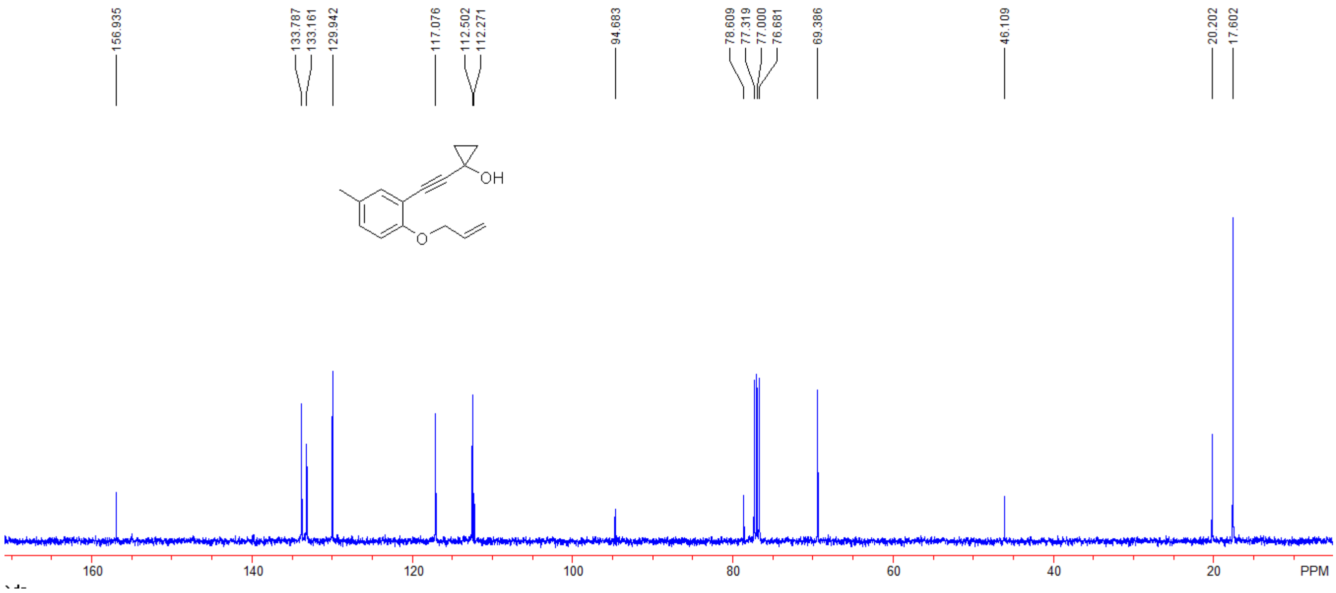

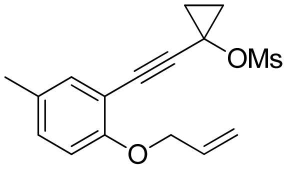

Compound **S4h**. 3.367 g, yield: 69%; yellow oil. ^{1}H NMR ($CDCl_3$, 400 MHz, TMS) δ 1.33-1.37 (m, 2H, CH_2), 1.63-1.66 (m, 2H, CH_2), 2.25 (s, 3H, CH_3), 3.31 (s, 3H, CH_3), 4.53-4.55 (m, 2H, CH_2), 5.28-5.31 (m, 1H, =CH_2), 5.41-5.45 (m, 1H, =CH_2), 5.99-6.08 (m, 1H, =CH), 6.75-6.77 (m, 1H, Ar), 7.07-7.10 (m, 1H, Ar), 7.167-7.172 (m, 1H, Ar). ^{13}C NMR ($CDCl_3$, 100 MHz, TMS) δ 16.9, 20.1, 39.9, 55.0, 69.3, 83.1, 89.1, 110.8, 112.0, 117.8, 130.0, 130.9, 132.8, 133.6, 157.3. IR (neat) ν 3023, 2924, 2227, 1606, 1497, 1455, 1359, 1285, 1170, 1157, 1128, 996, 974, 935, 814 cm^{-1}. MS (%) m/e 306 (M^+, 16.28), 185 (54.99), 170 (45.22), 142 (61.56), 141 (64.56), 129 (100.00), 128 (80.40), 115 (80.34), 55 (52.49). HRMS (EI) calcd. for $C_{16}H_{18}O_4S$: 306.0926, Found: 306.0930.

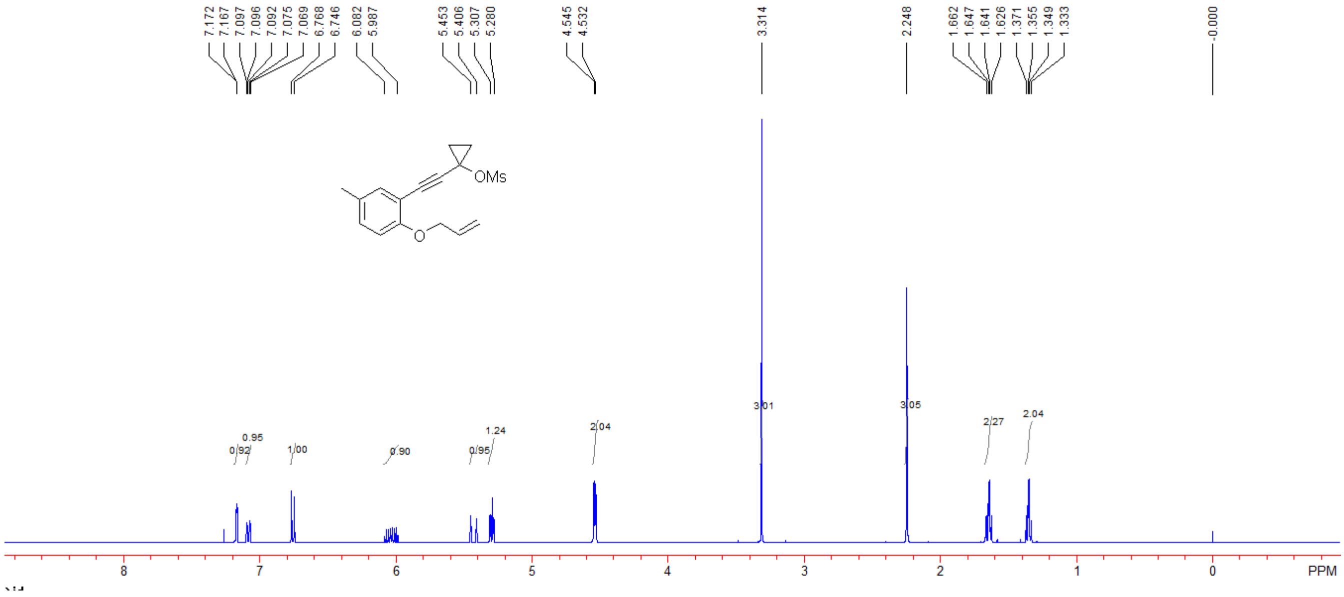

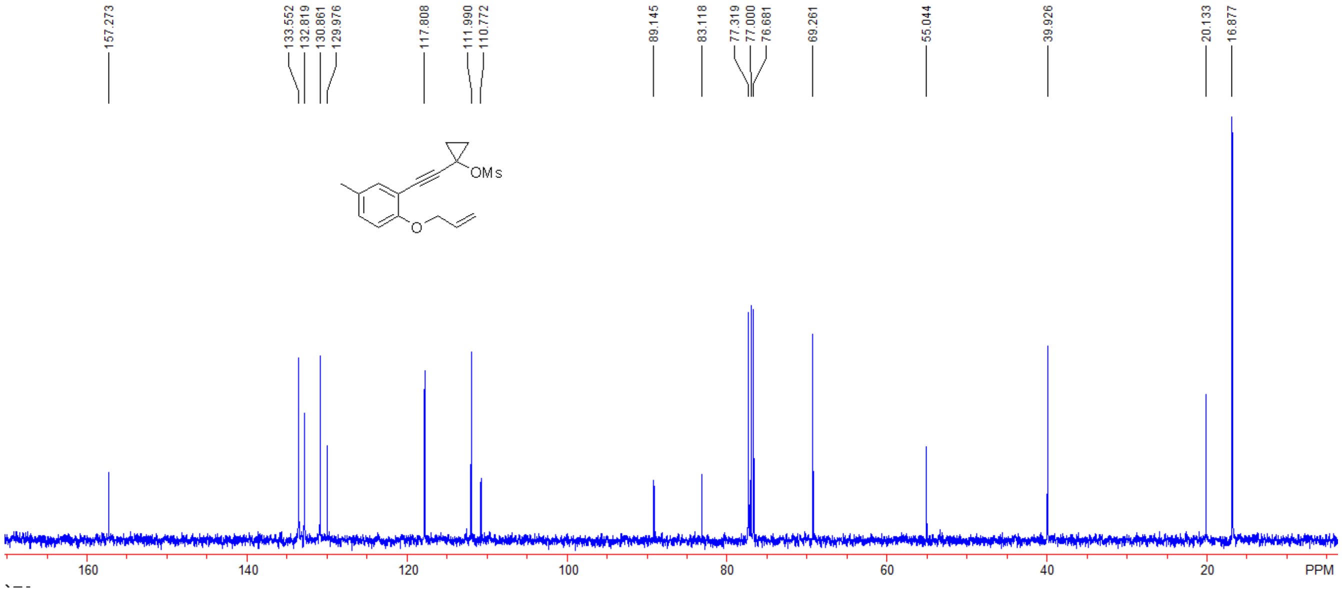

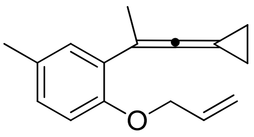

Compound **1h**. 520 mg, yield: 80%; colorless oil. ^{1}H NMR ($CDCl_3$, 400 MHz, TMS) δ 1.48-1.51 (m, 2H, CH_2), 1.53-1.56 (m, 2H, CH_2), 2.17 (s, 3H, CH_3), 2.27 (s, 3H, CH_3), 4.50 (d, J = 6.8 Hz, 2H, CH_2), 5.22-5.25 (m, 1H, =CH_2), 5.36-5.40 (m, 1H, =CH_2), 5.97-6.07 (m, 1H, =CH), 6.75 (d, J = 8.4 Hz, 1H, Ar), 6.94-6.97 (m, 1H, Ar), 7.064-7.069 (m, 1H, Ar). ^{13}C NMR ($CDCl_3$, 100 MHz, TMS) δ 7.5, 20.1, 20.5, 69.6, 75.4, 100.5, 112.9, 117.2, 128.0, 128.5, 129.5, 130.0, 133.8, 153.8, 190.4. IR (neat) ν 2981, 2914, 2860, 2009, 1604, 1502, 1454, 1257, 1228, 1147, 1075, 1021, 994, 800, 668

cm^{-1}. MS (%) m/e 226 (M$^+$, 7.20), 186 (19.91), 185 (100.00), 170 (24.09), 142 (52.15), 141 (40.45), 128 (25.46), 115 (36.51), 43 (20.43). HRMS (EI) calcd. for $C_{16}H_{18}O$: 226.1358, Found: 226.1361.

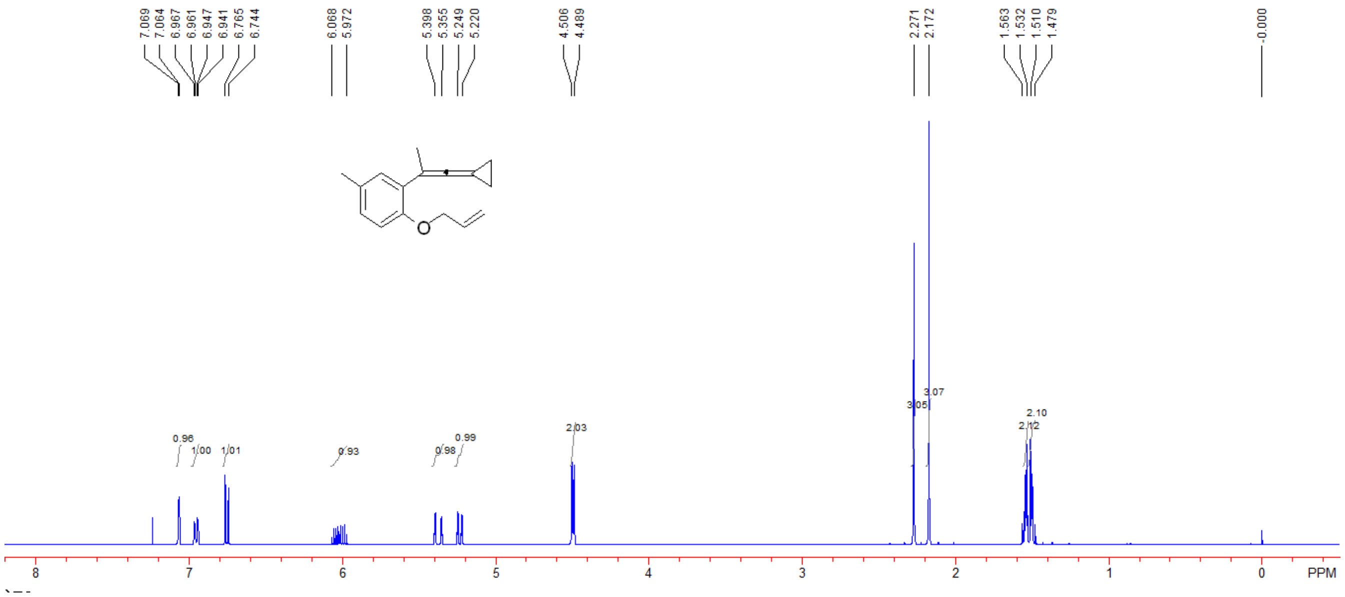

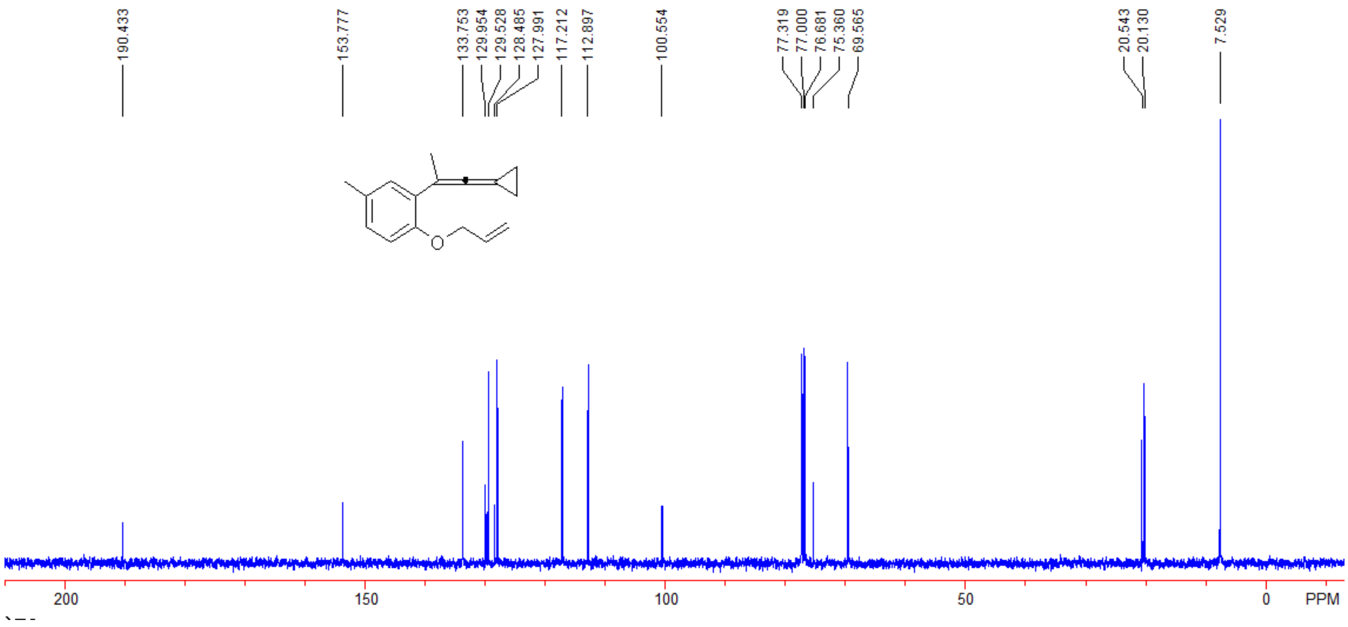

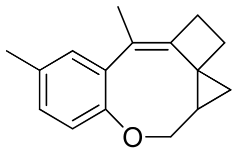

Compound **2h**. 97 mg, yield: 86%; colorless oil. ^{1}H NMR ($CDCl_3$, 400 MHz, TMS) δ 0.36 (dd, J_1 = 4.8 Hz, J_2 = 7.2 Hz, 1H, CH_2), 0.45 (dd, J_1 = 4.8 Hz, J_2 = 9.6 Hz, 1H, CH_2), 1.13-1.18 (m, 1H, CH), 1.94 (s, 3H, CH_3), 2.13-2.23 (m, 2H, CH_2), 2.28 (s, 3H, CH_3), 2.78-2.85 (m, 1H, CH_2), 2.97-3.05 (m, 1H, CH_2), 4.32 (d, J = 12.8 Hz, 1H, CH_2), 4.79 (dd, J_1 = 6.0 Hz, J_2 = 12.8 Hz, 1H, CH_2), 6.84 (d, J

= 8.4 Hz, 1H, Ar), 6.96-7.00 (m, 1H, Ar), 7.02 (s, 1H, Ar). ^{13}C NMR ($CDCl_3$, 100 MHz, TMS) δ 15.3, 17.4, 20.8, 24.7, 27.6, 27.8, 32.2, 70.5, 119.3, 120.7, 127.2, 128.7, 132.2, 134.3, 142.0, 153.5. IR (neat) ν 2915, 2860, 1489, 1455, 1403, 1290, 1234, 1211, 1134, 1057, 1011, 976, 819, 864, 755 cm^{-1}. MS (%) m/e 226 (M^+, 3.21), 196 (14.10), 95 (10.54), 93 (32.01), 86 (64.27), 84 (100.00), 63 (20.26), 49 (14.82), 47 (17.33). HRMS (EI) calcd. for $C_{16}H_{18}O$: 226.1358, Found: 226.1355; Enantiomeric excess was determined by HPLC with a Chiralcel PA-2 column [λ = 214 nm; eluent: Hexane/Isopropanol = 90/10; Flow rate: 0.70 mL/min; t_{minor} = 14.71 min, t_{major} = 16.81 min; ee% = 80%; $[\alpha]^{20}_D$ = -0.9170 (c 0.93, CH_2Cl_2)].

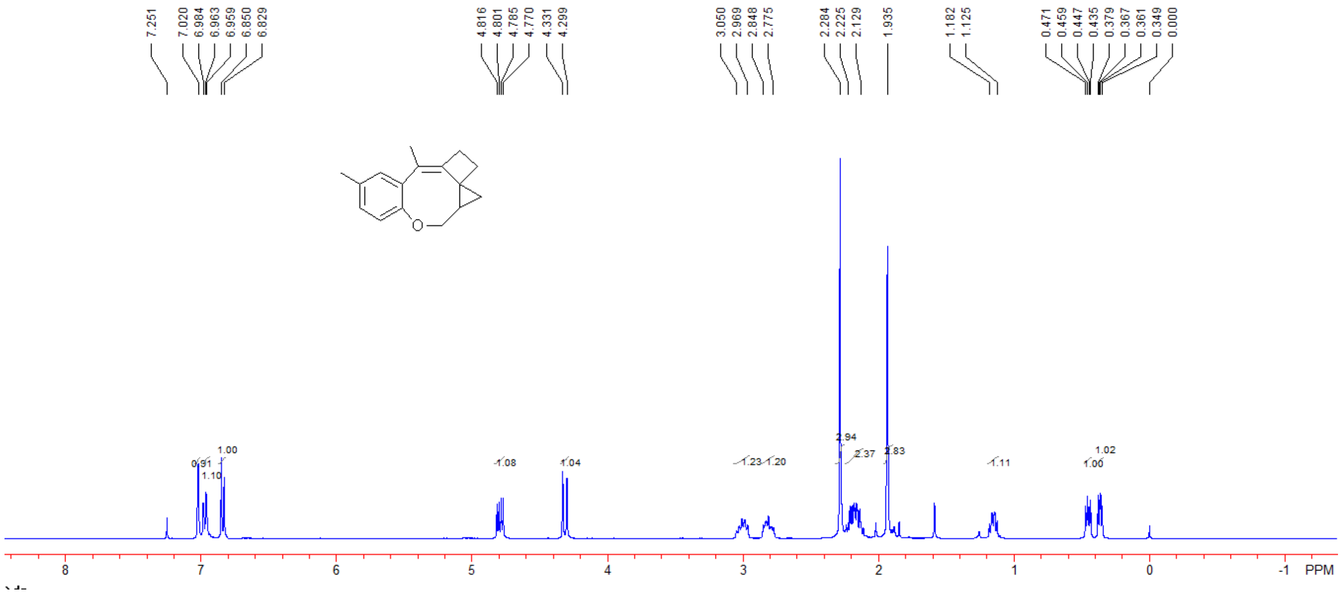

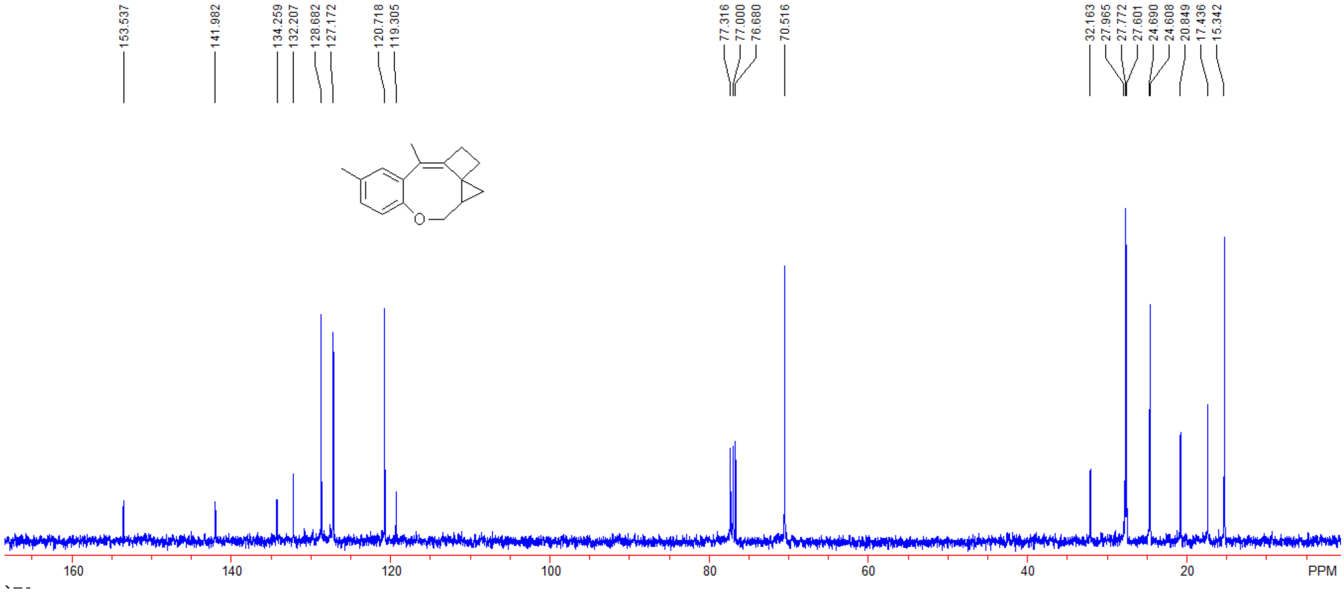

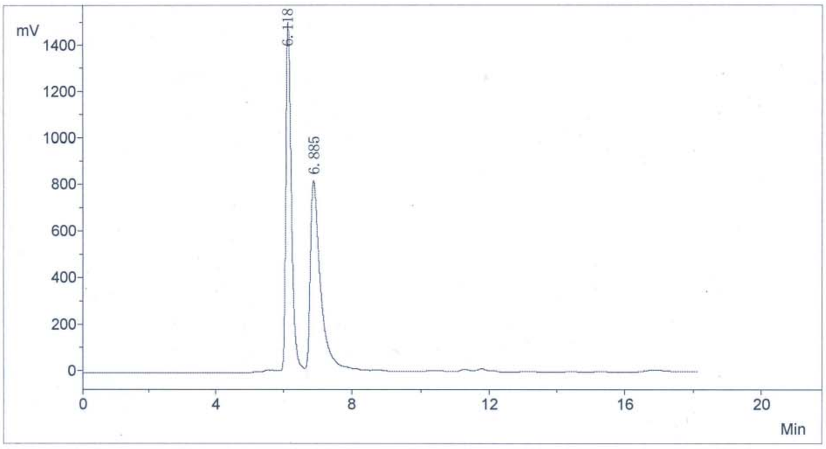

No.	PeakNo	ID.Name	R.Time	PeakHeight	PeakArea	PerCent
1	1		6.118	1466322.4	16868525.8	49.0482
2	2		6.885	818142.1	17523209.8	50.9518
Total				2284464.5	34391735.6	100.0000

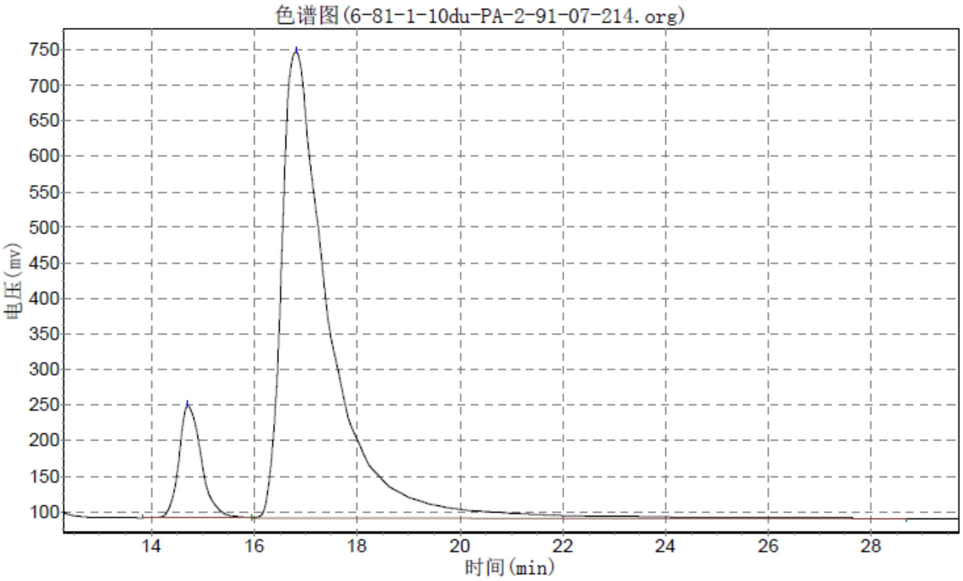

分析结果表

峰号	峰名	保留时间	峰高	峰面积	含量
1		14.712	157226.625	4802006.000	10.1423
2		16.813	656023.000	42544100.000	89.8577
总计			813249.625	47346106.000	100.0000

Translation: a Chiralcel PA-2 column [λ = 214 nm; eluent: Hexane/Isopropanol = 90/10; Flow rate: 0.70 mL/min; t_{minor} = 14.71 min, t_{major} = 16.81 min; ee% = 80%.]

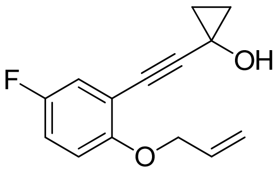

Compound **S3i**. 3.712 g, yield: 70%; yellow oil. ^{1}H NMR ($CDCl_3$, 400 MHz, TMS) δ 1.07-1.11 (m, 2H, CH_2), 1.17-1.21 (m, 2H, CH_2), 3.45 (br, 1H, OH), 4.54-4.56 (m, 2H, CH_2), 5.26-5.29 (m, 1H, =CH_2), 5.42-5.47 (m, 1H, =CH_2), 5.98-6.08 (m, 1H, =CH), 6.75-6.78 (m, 1H, Ar), 6.89-6.94 (m, 1H, Ar), 7.03-7.06 (m, 1H, Ar). ^{13}C NMR ($CDCl_3$, 100 MHz, TMS) δ 17.6, 45.8, 69.9, 96.2, 113.7 (d, $J_{C\text{-}F}$ = 8.9 Hz), 113.9 (d, $J_{C\text{-}F}$ = 5.7 Hz), 115.7 (d, $J_{C\text{-}F}$ = 23.4 Hz), 117.4, 119.4 (d, $J_{C\text{-}F}$ = 24.3 Hz), 132.8, 155.3, 156.4 (d, $J_{C\text{-}F}$ = 238.2 Hz). ^{19}F NMR (376 MHz, $CDCl_3$, $CFCl_3$): δ -123.26 ~ -123.19 (m, 1F). IR (neat) ν 3337, 3097, 3008, 2226, 1606, 1491, 1422, 1261, 1210, 1149, 1020, 964, 871, 803, 732 cm^{-1}. MS (%) m/e 232 (M^+, 4.48), 175 (55.56), 163 (63.31), 149 (95.64), 146 (47.38), 133 (74.37), 115 (43.74), 107 (64.39), 55 (100.00). HRMS (EI) calcd. for $C_{14}H_{12}O_2F[M-H]^+$: 231.0821, Found: 231.0819.

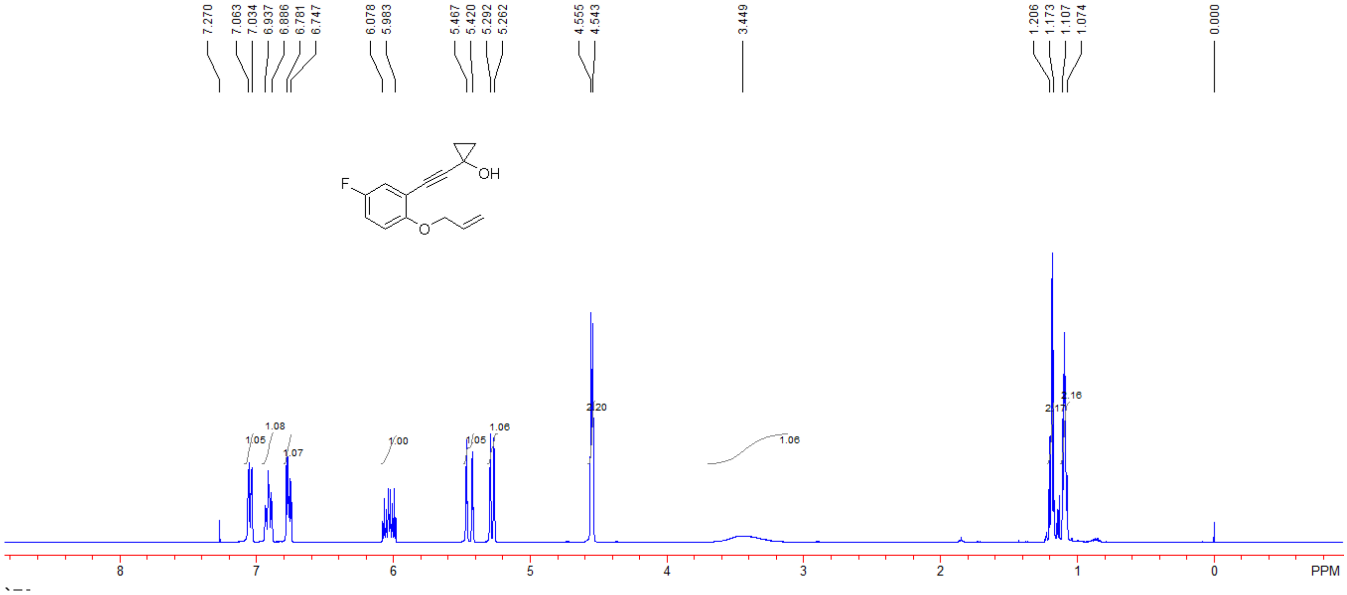

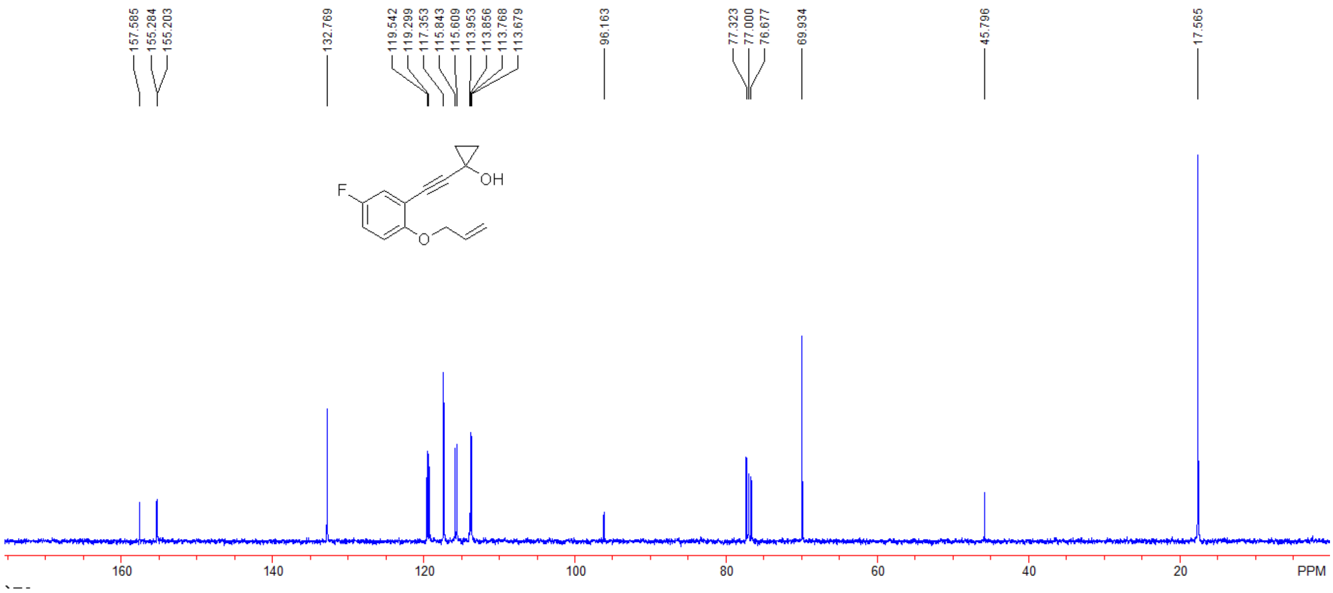

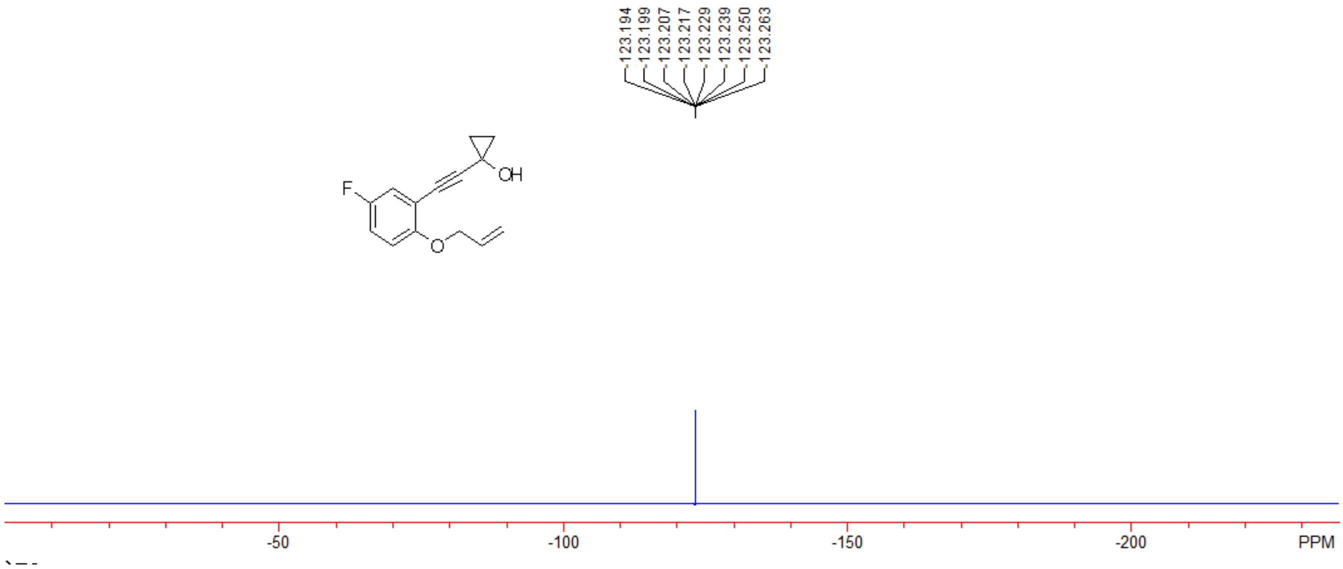

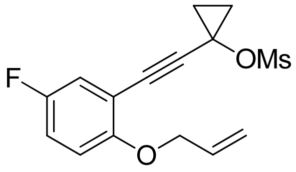

Compound **S4i**. 3.413 g, yield: 69%; yellow oil (viscous oil contains little DCM). ^{1}H NMR ($CDCl_3$, 400 MHz, TMS) δ 1.35-1.39 (m, 2H, CH_2), 1.64-1.68 (m, 2H, CH_2), 3.30 (s, 3H, CH_3), 4.54 (s, 2H, CH_2), 5.30-5.33 (m, 1H, $=CH_2$), 5.41-5.46 (m, 1H, $=CH_2$), 5.98-6.06 (m, 1H, =CH), 6.79-6.82 (m, 1H, Ar), 6.97-7.07 (m, 2H, Ar). ^{13}C NMR ($CDCl_3$, 100 MHz, TMS) δ 16.9, 39.9, 54.6, 69.8, 81.6 (d, J_{C-F} = 3.3 Hz), 90.4, 112.2 (d, J_{C-F} = 9.7 Hz), 113.2 (d, J_{C-F} = 8.8 Hz), 116.7 (d, J_{C-F} = 22.6 Hz), 118.1, 119.3 (d, J_{C-F} = 24.3 Hz), 132.5, 155.6, 156.2 (d, J_{C-F} = 239.0 Hz). ^{19}F NMR (376 MHz,

CDCl3, CFCl3): δ -123.16 ~ -123.09 (m, 1F). IR (neat) ν 3085, 3020, 2259, 1586, 1493, 1423, 1360, 1261, 1168, 1148, 976, 939, 893, 817, 730 cm^{-1}. MS (%) m/e 310 (M$^+$, 4.91), 199 (26.11), 189 (31.68), 162 (29.64), 149 (29.55), 146 (100.00), 134 (38.90), 133 (44.37), 106 (36.19). HRMS (EI) calcd. for $C_{15}H_{15}O_4SF$: 310.0675, Found: 310.0680.

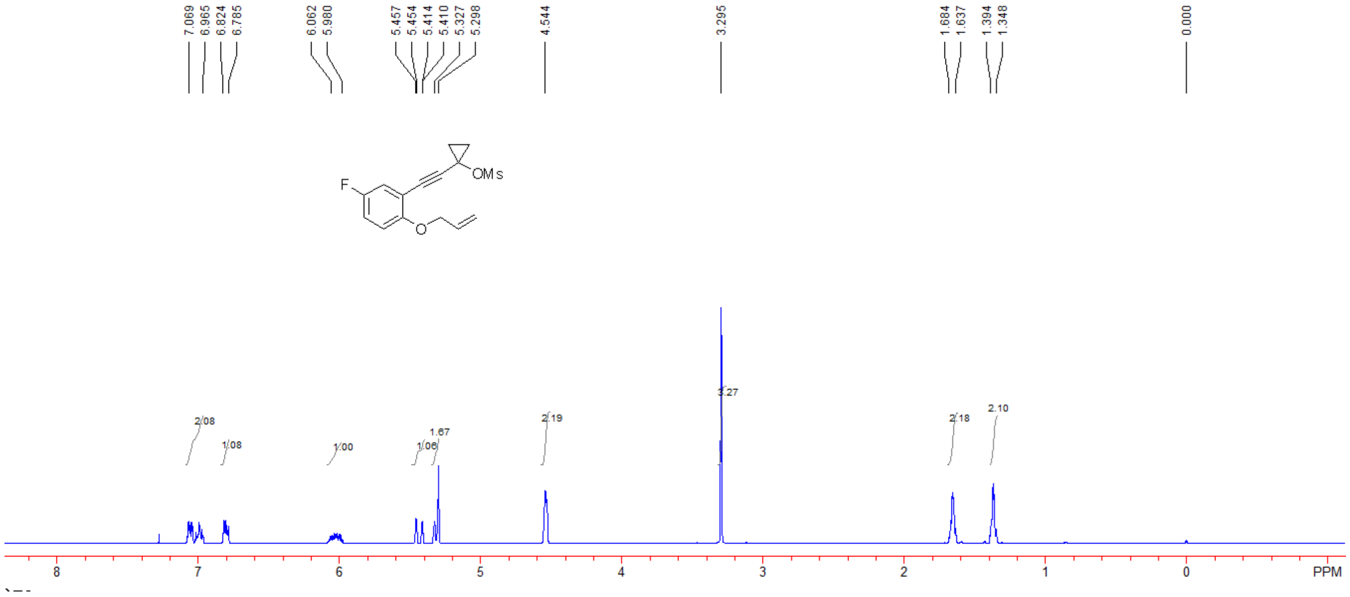

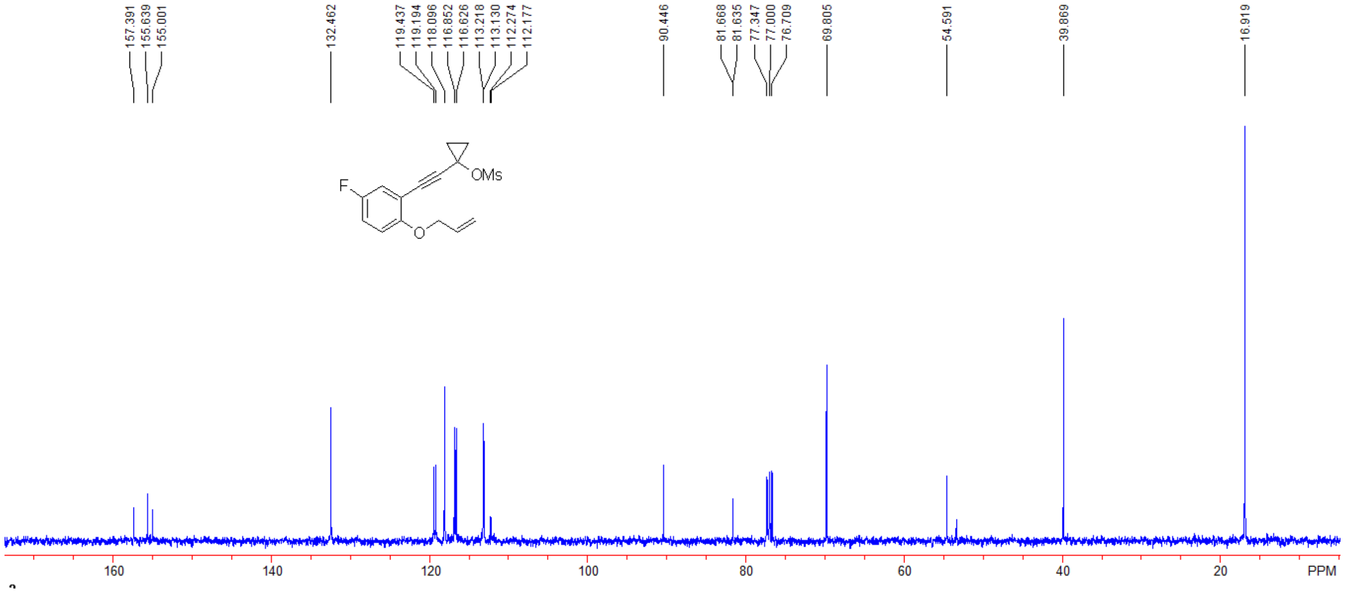

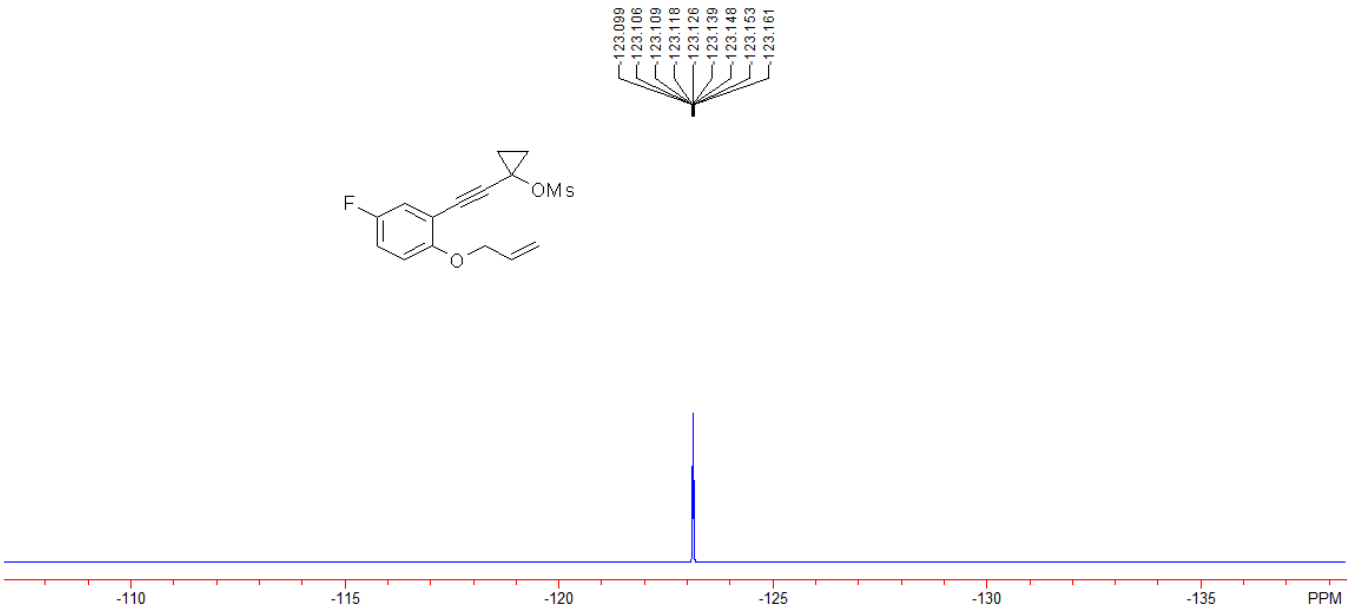

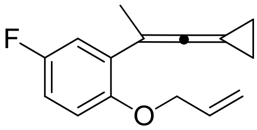

Compound **1i**. 530 mg, yield: 77%; colorless oil. ^{1}H NMR ($CDCl_3$, 400 MHz, TMS) δ 1.51-1.54 (m, 2H, CH_2), 1.56-1.59 (m, 2H, CH_2), 2.16 (s, 3H, CH_3), 4.47 (d, J = 5.6 Hz, 2H, CH_2), 5.24-5.26 (m, 1H, $=CH_2$), 5.35-5.39 (m, 1H, $=CH_2$), 5.95-6.04 (m, 1H, =CH), 6.75-6.79 (m, 1H, Ar), 6.81-6.85 (m, 1H, Ar), 6.96-6.99 (m, 1H, Ar). ^{13}C NMR ($CDCl_3$, 100 MHz, TMS) δ 7.9, 19.9, 70.1, 75.7, 99.5, 113.2 (d, $J_{C\text{-}F}$ = 22.7 Hz), 113.8 (d, $J_{C\text{-}F}$ = 8.4 Hz), 115.3 (d, $J_{C\text{-}F}$ = 23.6 Hz), 117.6, 130.3 (d, $J_{C\text{-}F}$ = 7.2 Hz), 133.4, 152.1, 157.1 (d, $J_{C\text{-}F}$ = 236.8 Hz), 190.9. ^{19}F NMR (376 MHz, $CDCl_3$, $CFCl_3$): δ -123.61 ~ -123.53 (m, 1F). IR (neat) ν 2984, 2913, 2862, 2008, 1606, 1499, 1417, 1368, 1219, 1174, 1052, 1019, 995, 895, 800 cm^{-1}. MS (%) m/e 230 (M^{+}, 10.67), 190 (19.35), 189 (100.00), 170 (19.92), 149 (20.80), 146 (67.75), 133 (31.80), 115 (15.59), 43 (21.28). HRMS (EI) calcd. for $C_{15}H_{15}OF$: 230.1107, Found: 230.1109.

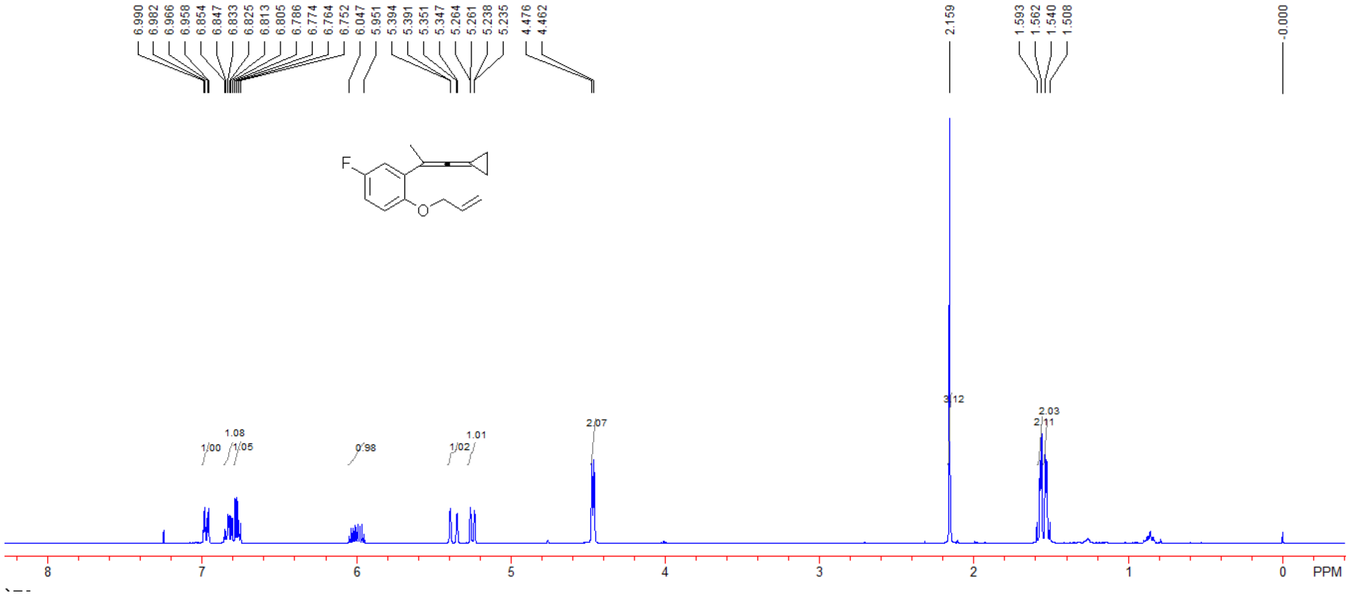
6.990
6.982
6.966
6.958
6.854
6.847
6.833
6.825
6.813
6.805
6.786
6.774
6.764
6.752
6.047
5.951
5.394
5.391
5.351
5.347
5.264
5.261
5.238
5.235
4.476
4.462
2.159
1.593
1.562
1.540
1.508
-0.000
F
O
PPM

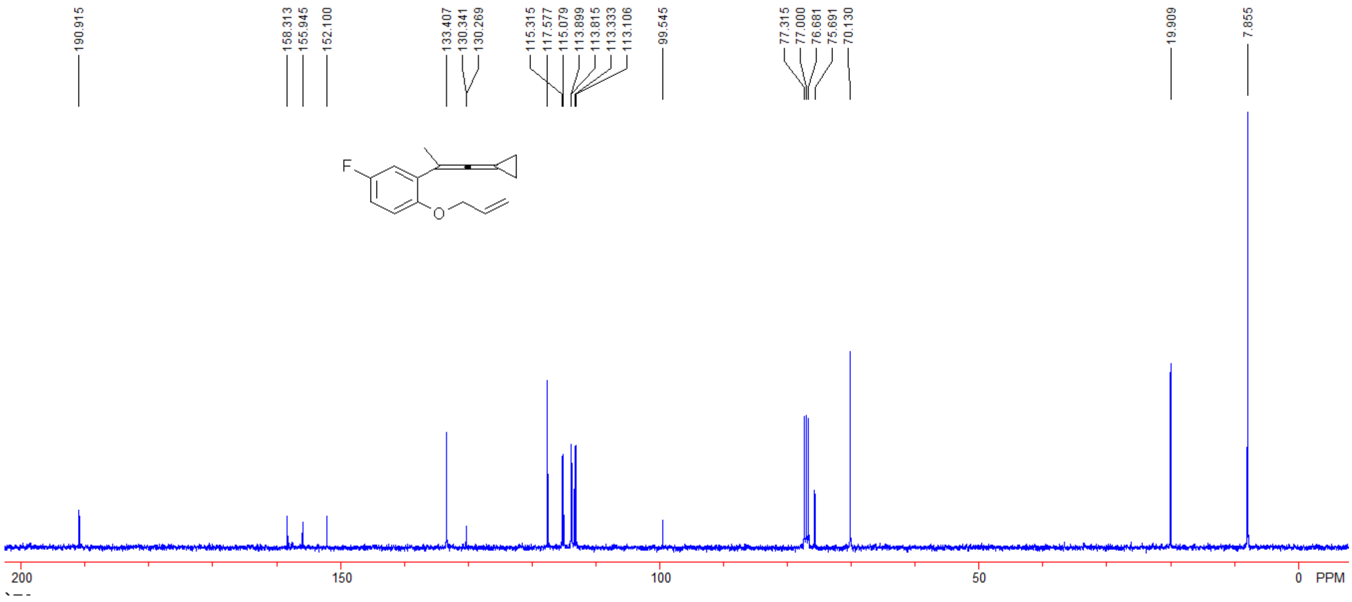
190.915
158.313
155.945
152.100
133.407
130.341
130.269
115.315
117.577
115.079
113.899
113.815
113.333
113.106
99.545
77.315
77.000
76.681
75.691
70.130
19.909
7.865
F
O
PPM

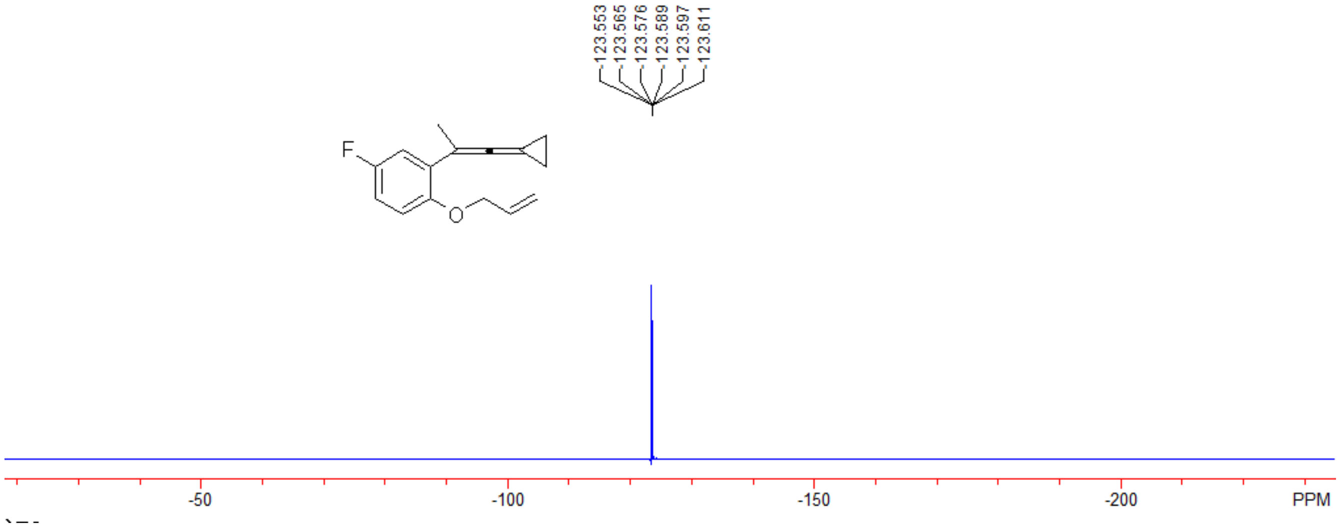
-123.553
-123.565
-123.576
-123.589
-123.597
-123.611
F
O
PPM

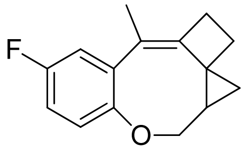

Compound **2i**. 94 mg, yield: 82%; colorless oil. ^{1}H NMR ($CDCl_3$, 400 MHz, TMS) δ 0.40 (dd, J_1 = 4.8 Hz, J_2 = 7.2 Hz, 1H, CH_2), 0.50 (dd, J_1 = 4.8 Hz, J_2 = 9.6 Hz, 1H, CH_2), 1.15-1.19 (m, 1H, CH), 1.91 (s, 3H, CH_3), 2.12-2.26 (m, 2H, CH_2), 2.78-2.85 (m, 1H, CH_2), 2.97-3.04 (m, 1H, CH_2), 4.32 (d, J = 12.4 Hz, 1H, CH_2), 4.82 (dd, J_1 = 5.6 Hz, J_2 = 12.4 Hz, 1H, CH_2), 6.85-6.91 (m, 3H, Ar). ^{13}C NMR ($CDCl_3$, 100 MHz, TMS) δ 15.6, 17.4, 24.5, 27.7, 29.7, 32.1, 70.9, 77.2, 112.9 (d, J_{C-F} = 22.7 Hz), 114.4 (d, J_{C-F} = 23.0 Hz), 118.4, 122.3 (d, J_{C-F} = 8.9 Hz), 136.2 (d, J_{C-F} = 7.8 Hz), 143.7, 151.8, 158.3 (d, J_{C-F} = 240.1 Hz). ^{19}F NMR (376 MHz, $CDCl_3$, $CFCl_3$): δ -120.96 ~ -120.04 (m, 1F). IR (neat) ν 3060, 2915, 2861, 1608, 1483, 1413, 1373, 1174, 1009, 979, 922, 866, 818, 747, 713 cm^{-1}. MS (%) m/e 230 (M^+, 53.01), 215 (59.95), 201 (52.34), 200 (100.00), 189 (40.61), 185 (60.16), 163 (44.46), 146 (32.87). HRMS (EI) calcd. for $C_{15}H_{15}OF$: 230.1107, Found: 230.1111.

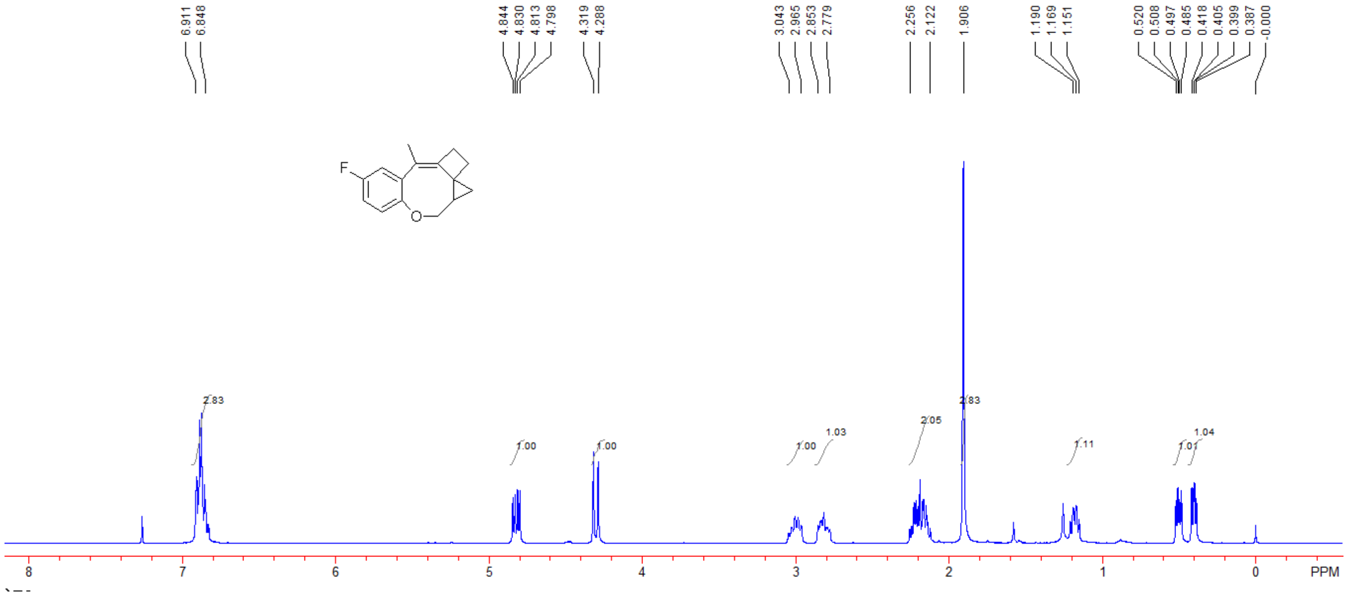

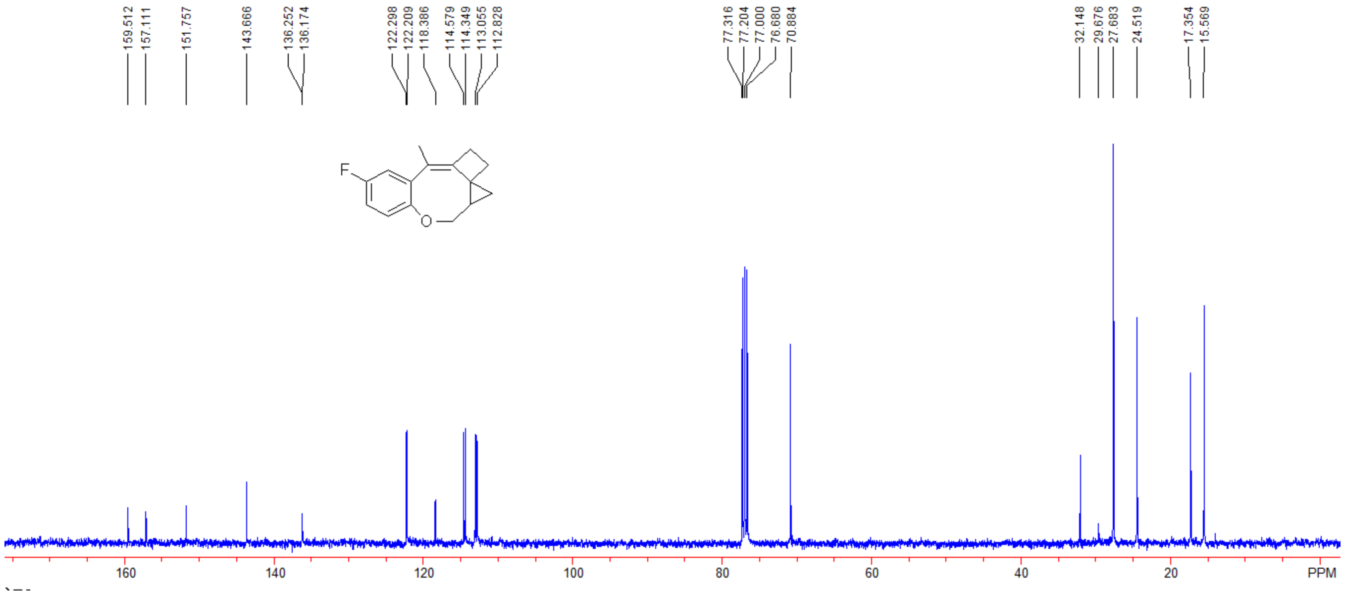

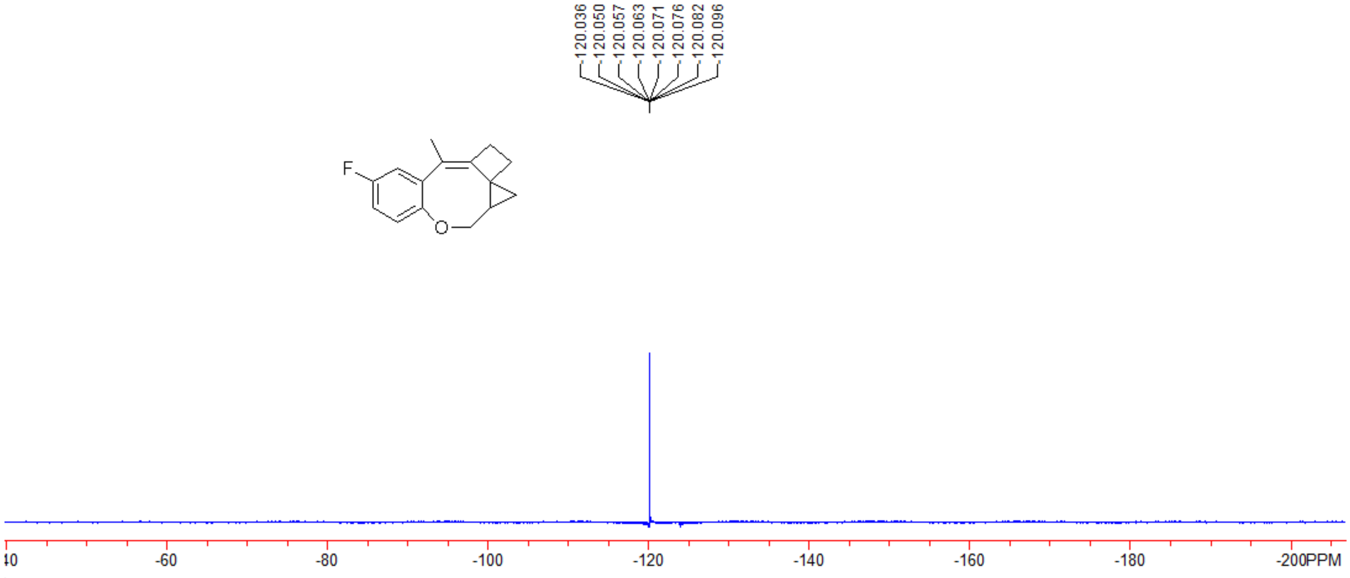

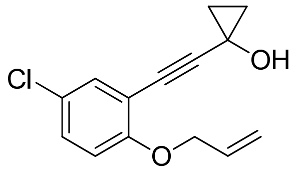

Compound **S3j**. 2.485 g, yield: 42%; yellow oil. ^{1}H NMR ($CDCl_3$, 400 MHz, TMS) δ 1.08-1.13 (m, 2H, CH_2), 1.15-1.21 (m, 2H, CH_2), 3.04 (br, 1H, OH), 4.56 (d, J = 6.4 Hz, 2H, CH_2), 5.29 (dd, J_1 = 1.2 Hz, J_2 = 10.8 Hz, 1H, =CH_2), 5.45 (dd, J_1 = 1.2 Hz, J_2 = 12.0 Hz, 1H, =CH_2), 5.98-6.08 (m, 1H, =CH), 6.75 (d, J = 8.4 Hz, 1H, Ar), 7.17 (dd, J_1 = 3.2 Hz, J_2 = 8.4 Hz, 1H, Ar), 7.32 (d, J = 2.0 Hz, 1H, Ar). ^{13}C NMR ($CDCl_3$, 100 MHz, TMS) δ 17.7, 46.0, 69.5, 77.3, 96.2, 113.5, 114.1, 117.5, 125.3, 129.1, 132.5, 132.7, 157.6. IR (neat) ν 3335, 3089, 2914, 2221, 1486, 1401, 1283, 1228,

1134, 1020, 994, 908, 802, 730 cm^{-1}. MS (%) m/e 248 (M^+, 6.41), 179 (44.22), 165 (72.40), 144 (39.97), 128 (44.55), 116 (49.63), 115 (94.41), 55 (100.00), 41 (47.54). HRMS (EI) calcd. for $C_{14}H_{13}O_2Cl$: 248.0604, Found: 248.0608.

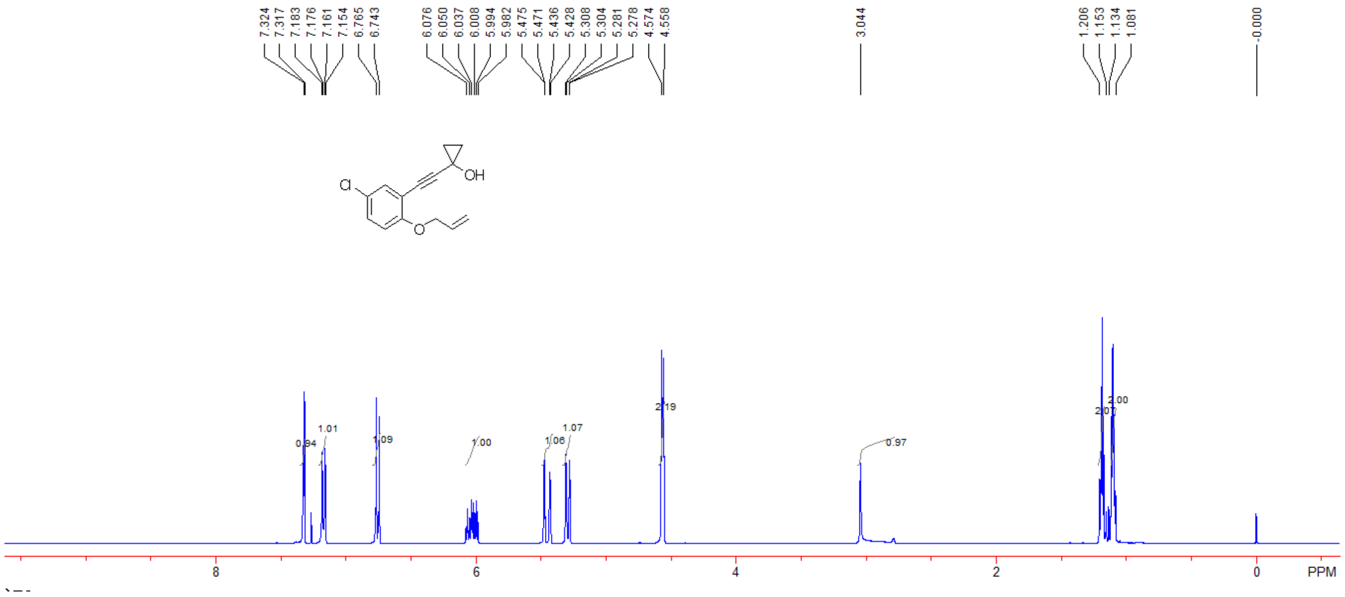

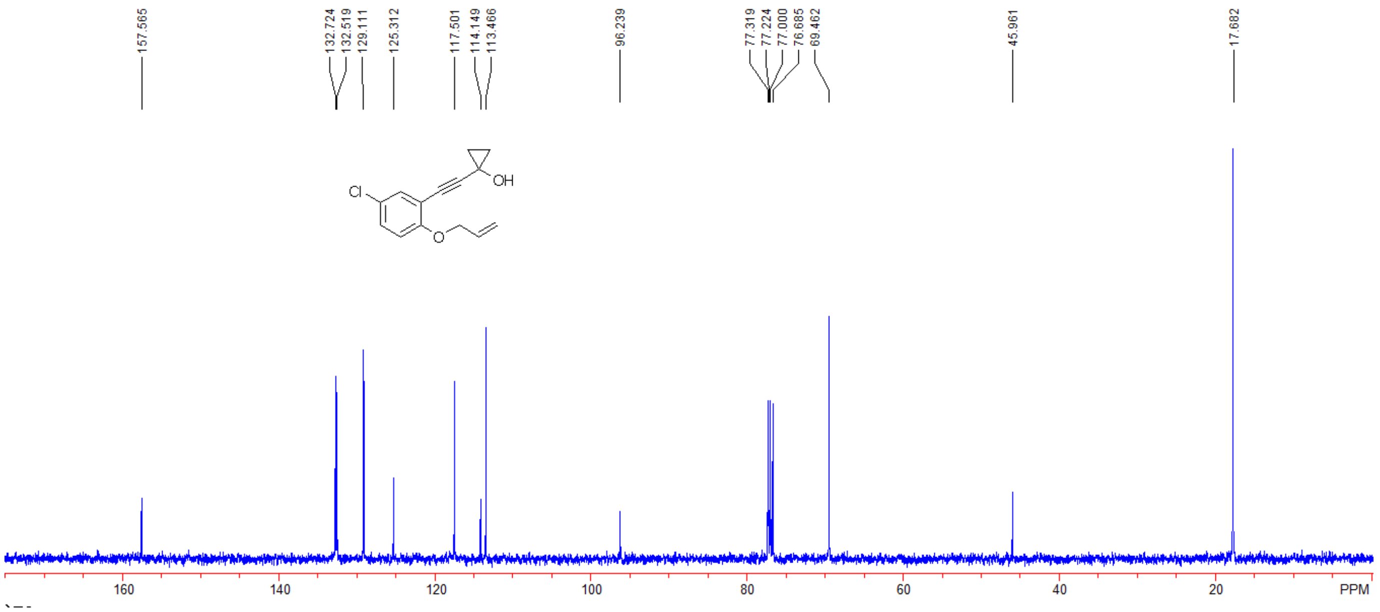

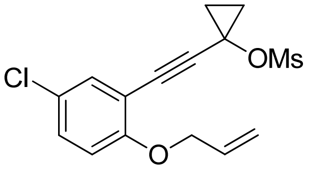

Compound **S4j**. 2.347 g, yield: 90%; yellow oil. ^{1}H NMR ($CDCl_3$, 400 MHz, TMS) δ 1.35-1.38 (m, 2H, CH_2), 1.64-1.68 (m, 2H, CH_2), 3.29 (s, 3H, CH_3), 4.55 (d, J = 4.8 Hz, 2H, CH_2), 5.30-5.34 (m,

1H, =CH_2), 5.42-5.46 (m, 1H, =CH_2), 5.97-6.07 (m, 1H, =CH), 6.79 (d, J = 8.8 Hz, 1H, Ar), 7.22-7.25 (m, 1H, Ar), 7.32 (d, J = 2.4 Hz, 1H, Ar). ^{13}C NMR ($CDCl_3$, 100 MHz, TMS) δ 16.9, 39.9, 54.6, 69.4, 81.4, 90.6, 112.6, 113.1, 118.2, 125.2, 130.0, 132.2, 132.5, 157.8. IR (neat) ν 3016, 2935, 2866, 2259, 1487, 1360, 1281, 1247, 1159, 1134, 995, 934, 906, 808, 728 cm^{-1}. MS (%) m/e 326 (M^+, 7.65), 170 (63.95), 155 (52.03), 150 (55.11), 128 (86.00), 127 (100.00), 115 (58.52), 55 (66.33), 41 (61.34). HRMS (EI) calcd. for $C_{15}H_{15}O_4SCl$: 326.0380, Found: 326.0383.

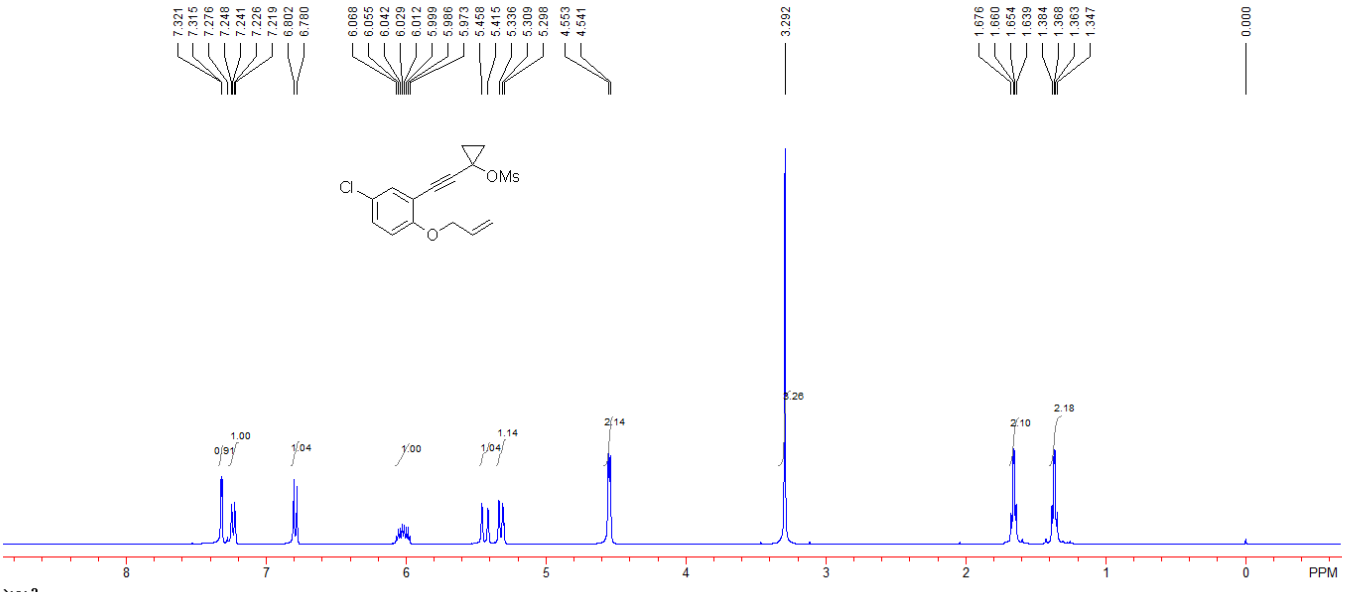

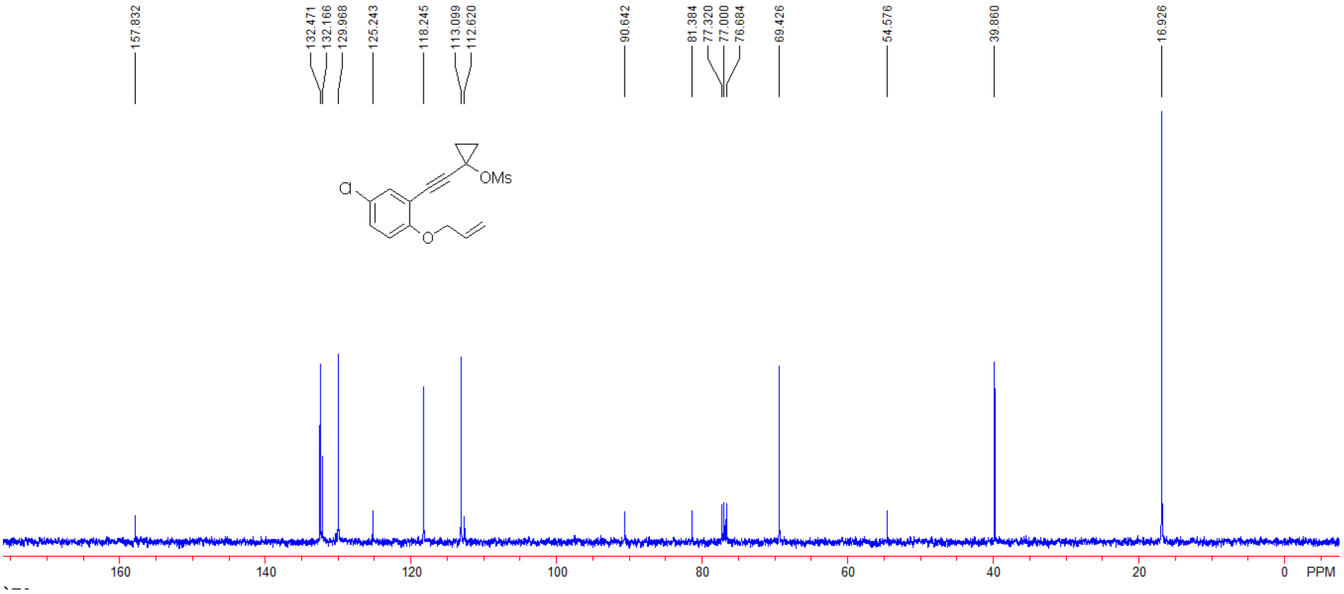

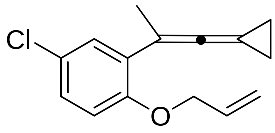

Compound **1j**. 594 mg, yield: 80%; colorless oil. ^{1}H NMR ($CDCl_3$, 400 MHz, TMS) δ 1.50-1.58 (m, 4H, CH_2), 2.15 (s, 3H, CH_3), 4.46 (d, J = 5.2 Hz, 2H, CH_2), 5.23-5.26 (m, 1H, =CH_2), 5.34-5.39 (m, 1H, =CH_2), 5.93-6.02 (m, 1H, =CH), 6.73 (d, J = 8.8 Hz, 1H, Ar), 7.07-7.10 (m, 1H, Ar), 7.210-7.217 (m, 1H, Ar). ^{13}C NMR ($CDCl_3$, 100 MHz, TMS) δ 7.8, 19.9, 69.5, 75.7, 99.4, 113.6, 117.7, 125.5, 127.0, 128.4, 130.3, 133.1, 154.4, 190.8. IR (neat) ν 2982, 2912, 1861, 2007, 1588, 1486, 1394, 1251, 1219, 1140, 1058, 1015, 927, 801, 783 cm^{-1}. MS (%) m/e 246 (M^+, 1.95), 205 (100.00), 170 (81.59), 169 (80.47), 155 (47.41), 152 (34.95), 142 (44.65), 141 (67.90), 115 (48.76). HRMS (EI) calcd. for $C_{15}H_{15}OCl$: 246.0811, Found: 246.0809.

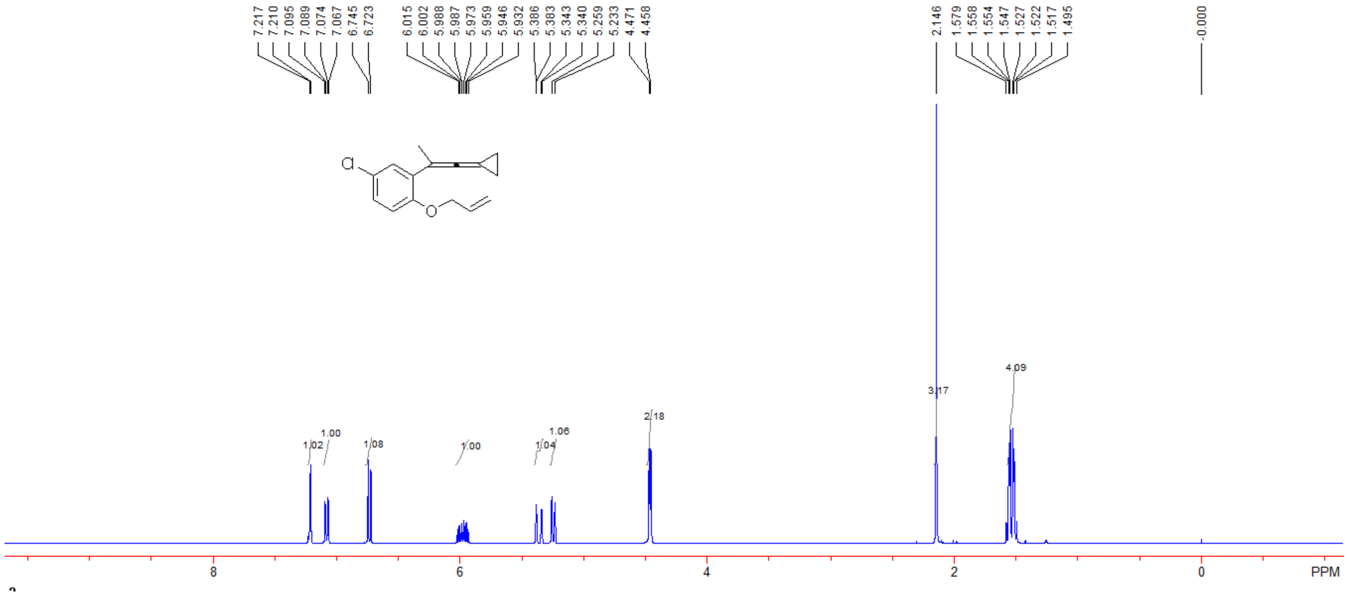

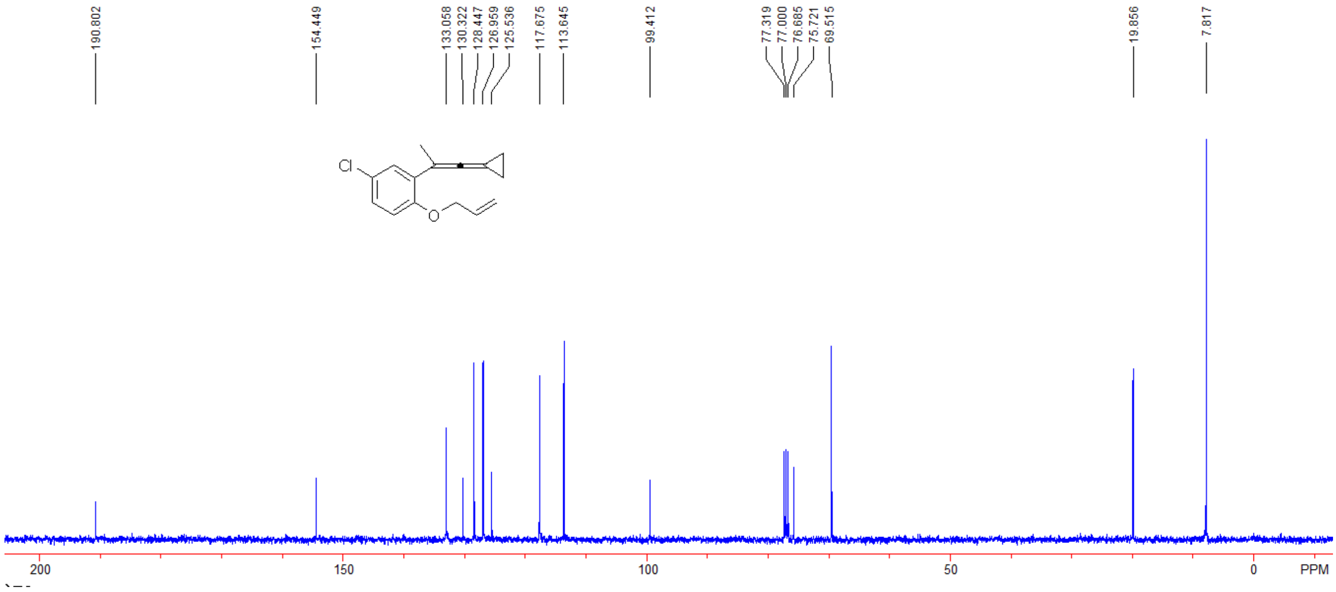

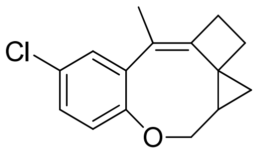

Compound **2j**. 103 mg, yield: 75%; colorless oil. ^{1}H NMR ($CDCl_3$, 400 MHz, TMS) δ 0.39-0.42 (m, 1H, CH_2), 0.49-0.52 (m, 1H, CH_2), 1.15-1.21 (m, 1H, CH), 1.91 (s, 3H, CH_3), 2.12-2.25 (m, 2H, CH_2), 2.77-2.85 (m, 1H, CH_2), 2.96-3.02 (m, 1H, CH_2), 4.31 (d, J = 12.4 Hz, 1H, CH_2), 4.83 (dd, J_1 = 5.6 Hz, J_2 = 12.4 Hz, 1H, CH_2), 6.87 (d, J = 8.8 Hz, 1H, Ar), 7.10-7.13 (m, 1H, Ar), 7.18-7.19 (m, 1H, Ar). ^{13}C NMR ($CDCl_3$, 100 MHz, TMS) δ 15.5, 17.3, 24.5, 27.6, 27.7, 32.2, 70.8, 118.1, 122.5, 126.7, 127.8, 128.1, 136.4, 143.9, 154.3. IR (neat) ν 3060, 2912, 2868, 1476, 1455, 1396, 1233, 1209, 1100, 1057, 1007, 973, 874, 820, 662 cm^{-1}. MS (%) m/e 246 (M$^+$, 34.74), 231 (81.99), 230 (93.62), 216 (100.00), 181 (92.99), 179 (56.79), 165 (68.52), 115 (62.25), 84 (56.85). HRMS (EI) calcd. for $C_{15}H_{15}OCl$: 246.0811, Found: 246.0812; Enantiomeric excess was determined by HPLC with a Chiralcel PA-2 column [λ = 214 nm; eluent: Hexane/Isopropanol = 90/10; Flow rate: 0.60 mL/min; t_{minor} = 14.37 min, t_{major} = 15.81 min; ee% = 81%; $[\alpha]^{20}_D$ = -3.4921 (c 0.97, CH_2Cl_2)].

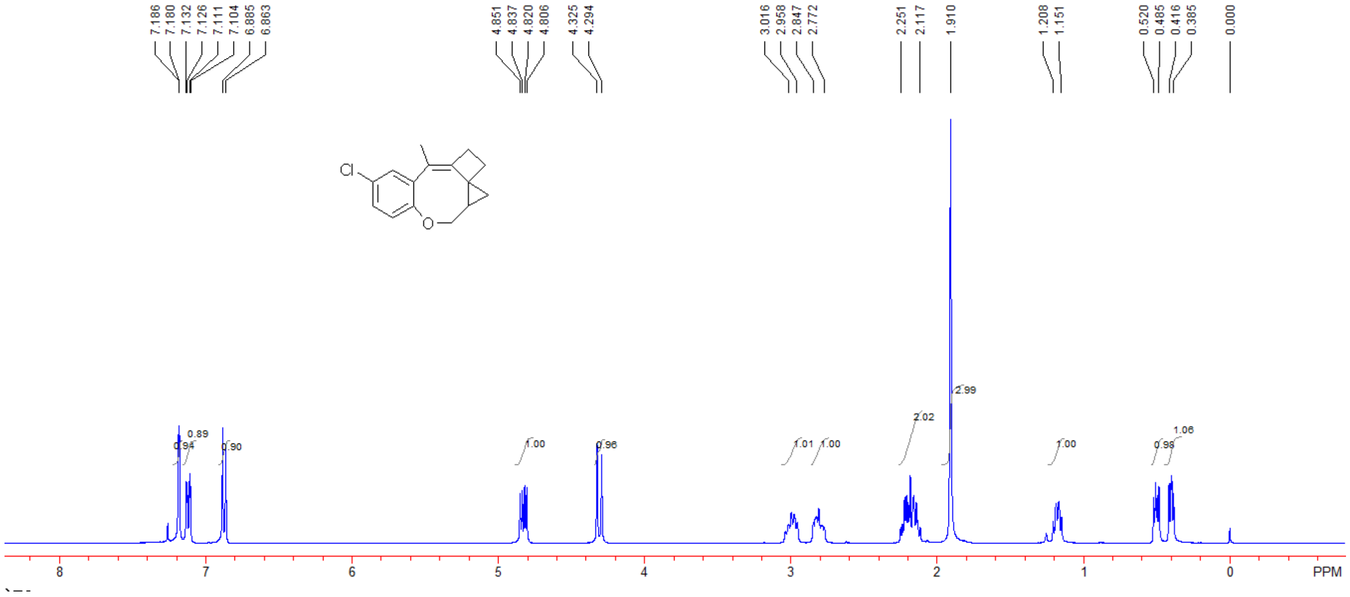

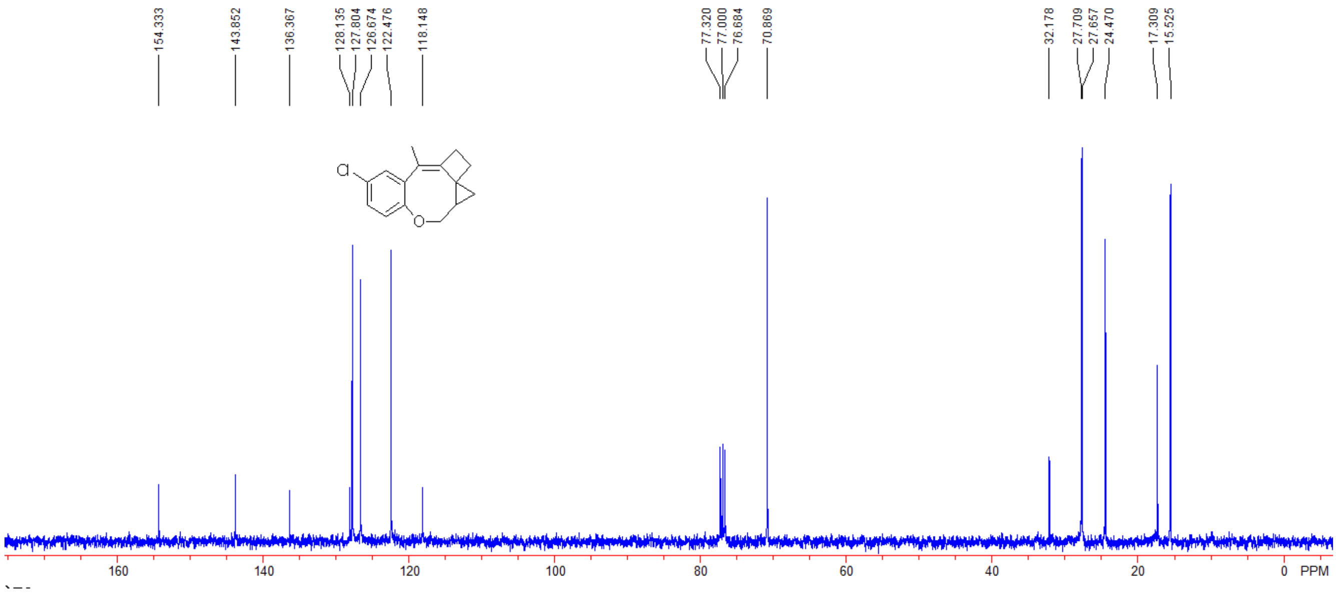

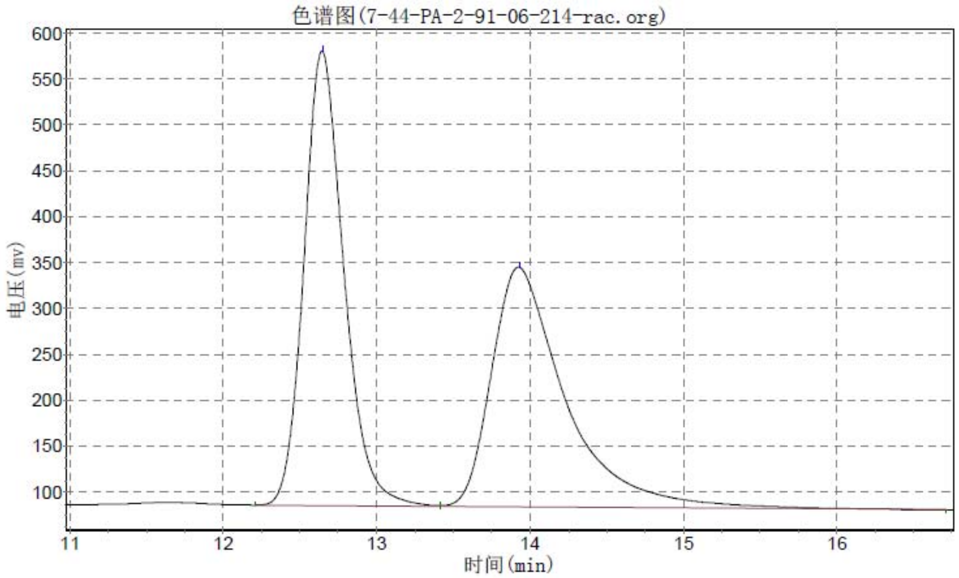

分析结果表

峰号	峰名	保留时间	峰高	峰面积	含量
1		12.648	496330.594	9025103.000	50.6127
2		13.932	261706.063	8806584.000	49.3873
总计			758036.656	17831687.000	100.0000

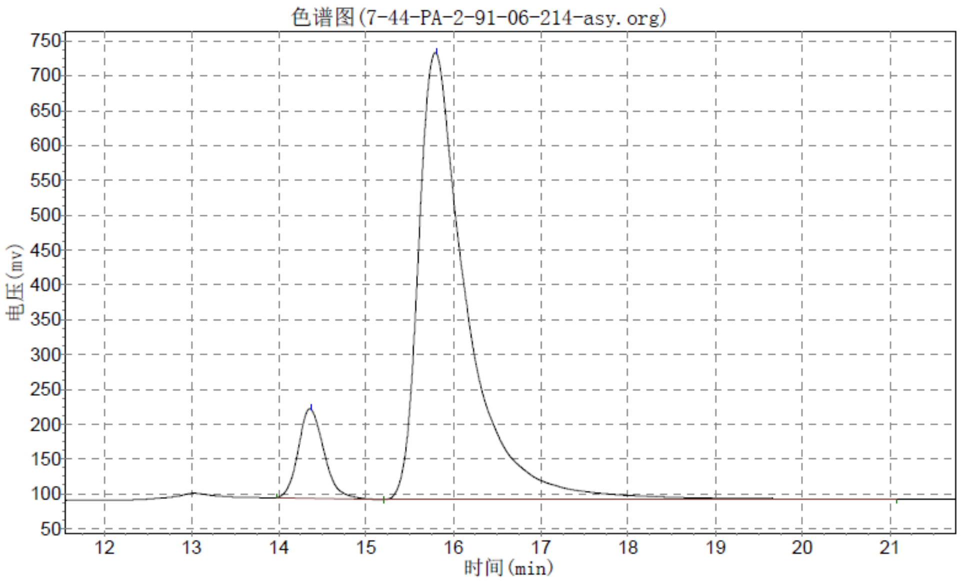

分析结果表

峰号	峰名	保留时间	峰高	峰面积	含量
1		14.373	128095.242	2626966.750	9.6369
2		15.807	640913.375	24632470.000	90.3631
总计			769008.617	27259436.750	100.0000

Translation: a Chiralcel PA-2 column [λ = 214 nm; eluent: Hexane/Isopropanol = 90/10; Flow rate: 0.60 mL/min; t_{minor} = 14.37 min, t_{major} = 15.81 min; ee% = 81%.]

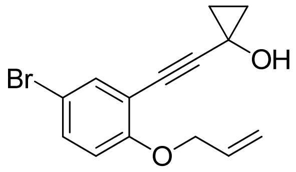

Compound **S3k**. 4.739 g, yield: 64%; yellow oil. ^{1}H NMR ($CDCl_3$, 400 MHz, TMS) δ 1.07-1.13 (m, 2H, CH_2), 1.15-1.20 (m, 2H, CH_2), 3.23 (br, 1H, OH), 4.55-4.56 (m, 2H, CH_2), 5.29 (dd, J_1 = 1.2 Hz, J_2 = 10.4 Hz, 1H, =CH_2), 5.45 (dd, J_1 = 1.6 Hz, J_2 = 17.2 Hz, 1H, =CH_2), 5.98-6.07 (m, 1H, =CH), 6.69 (d, J = 8.4 Hz, 1H, Ar), 7.29-7.31 (m, 1H, Ar), 7.45-7.46 (m, 1H, Ar). ^{13}C NMR ($CDCl_3$, 100 MHz, TMS) δ 17.6, 45.9, 69.3, 77.0, 96.4, 112.4, 113.8, 114.6, 117.5, 132.0, 132.4, 135.5, 158.0. IR (neat) ν 3351, 3076, 3010, 2927, 2190, 1648, 1486, 1448, 1280, 1227, 1132, 994, 926, 878, 802, 751 cm^{-1}. MS (%) m/e 292 (M^+, 1.40), 144 (64.59), 131 (41.53), 128 (57.19), 116 (38.71), 115 (100.00), 89 (38.66), 55 (94.90), 41 (59.35). HRMS (EI) calcd. for $C_{14}H_{13}O_2Br$: 292.0099, Found: 292.0097.

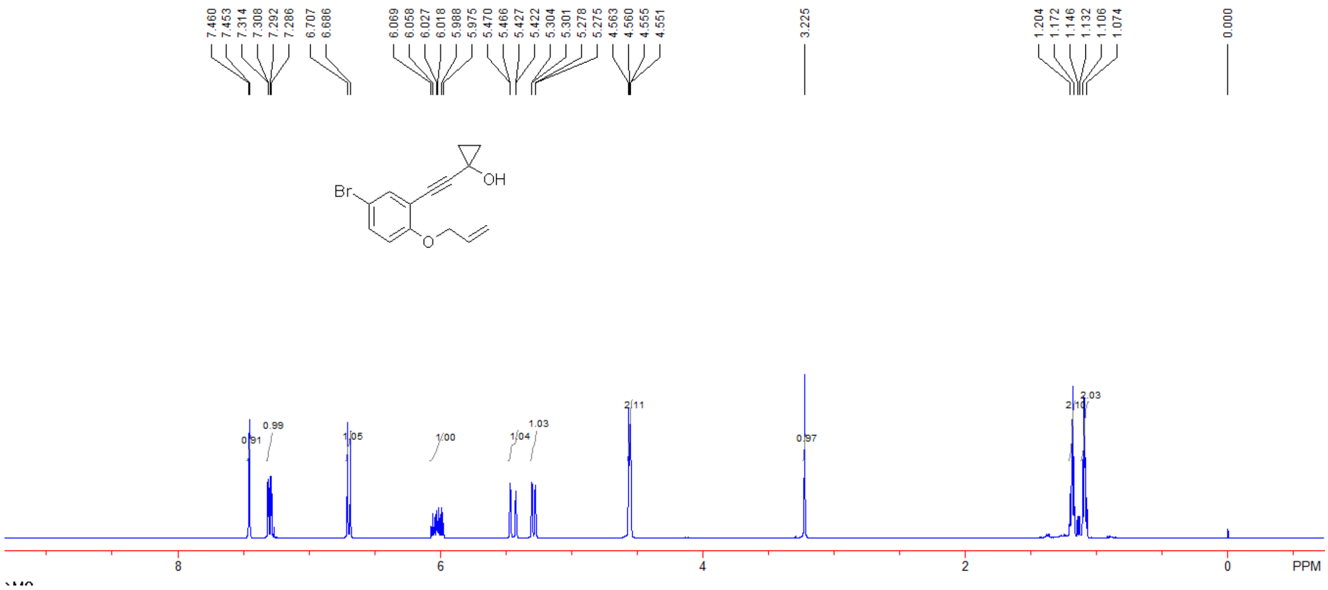

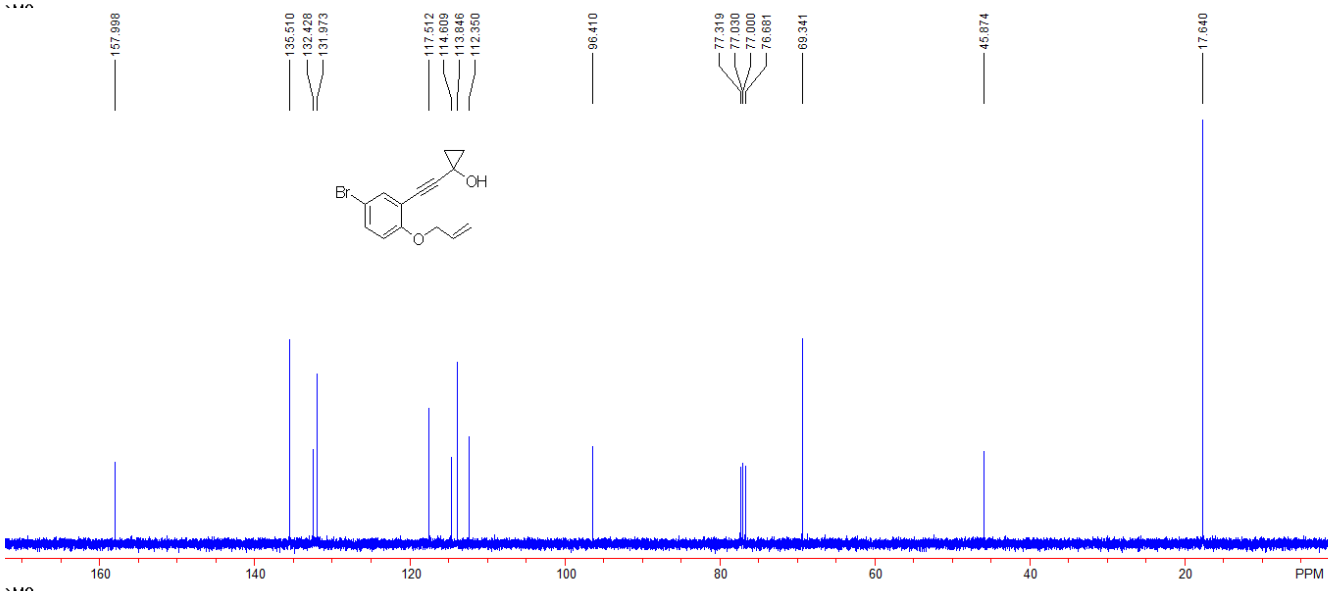

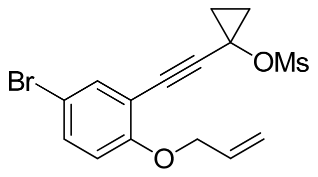

Compound **S4k**. 5.476 g, yield: 93%; yellow oil. ^{1}H NMR ($CDCl_3$, 400 MHz, TMS) δ 1.34-1.38 (m, 2H, CH_2), 1.64-1.68 (m, 2H, CH_2), 3.29 (s, 3H, CH_3), 4.55 (d, J = 5.2 Hz, 2H, CH_2), 5.32 (dd, J_1 = 1.2 Hz, J_2 = 10.4 Hz, 1H, =CH_2), 5.43 (dd, J_1 = 1.2 Hz, J_2 = 17.2 Hz, 1H, =CH_2), 5.98-6.05 (m, 1H, =CH), 6.74 (d, J = 8.8 Hz, 1H, Ar), 7.36-7.39 (m, 1H, Ar), 7.46 (d, J = 1.6 Hz, 1H, Ar). ^{13}C NMR ($CDCl_3$, 100 MHz, TMS) δ 17.0, 39.9, 54.6, 69.4, 81.3, 90.8, 112.3, 113.1, 113.5, 118.3, 132.1, 132.9, 135.3, 158.3. IR (neat) ν 3028, 2927, 2870, 2259, 1486, 1399, 1360, 1281, 1248, 1159, 1133, 997, 932, 905, 726 cm^{-1}. MS (%) m/e 370 (M^+, 5.51), 170 (76.78), 169 (100.00), 155 (54.03), 128

(59.10), 127 (58.77), 115 (42.45), 55 (49.97), 41 (48.45). HRMS (EI) calcd. for $C_{15}H_{15}O_4SBr$: 369.9877, Found: 369.9876.

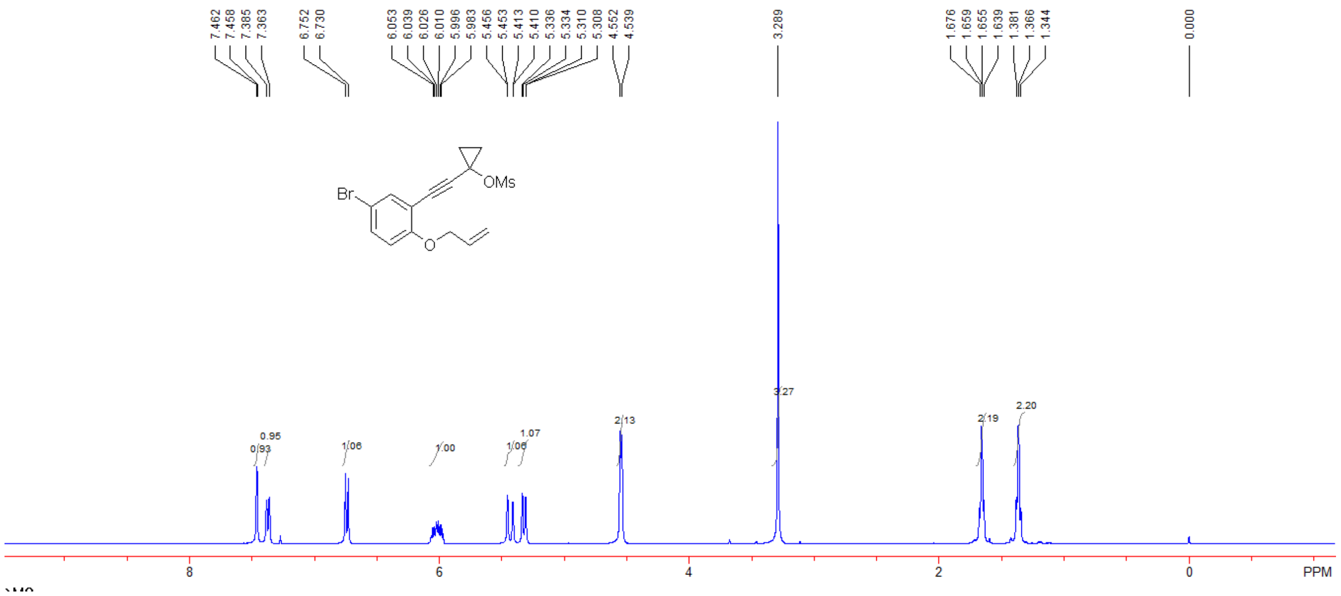

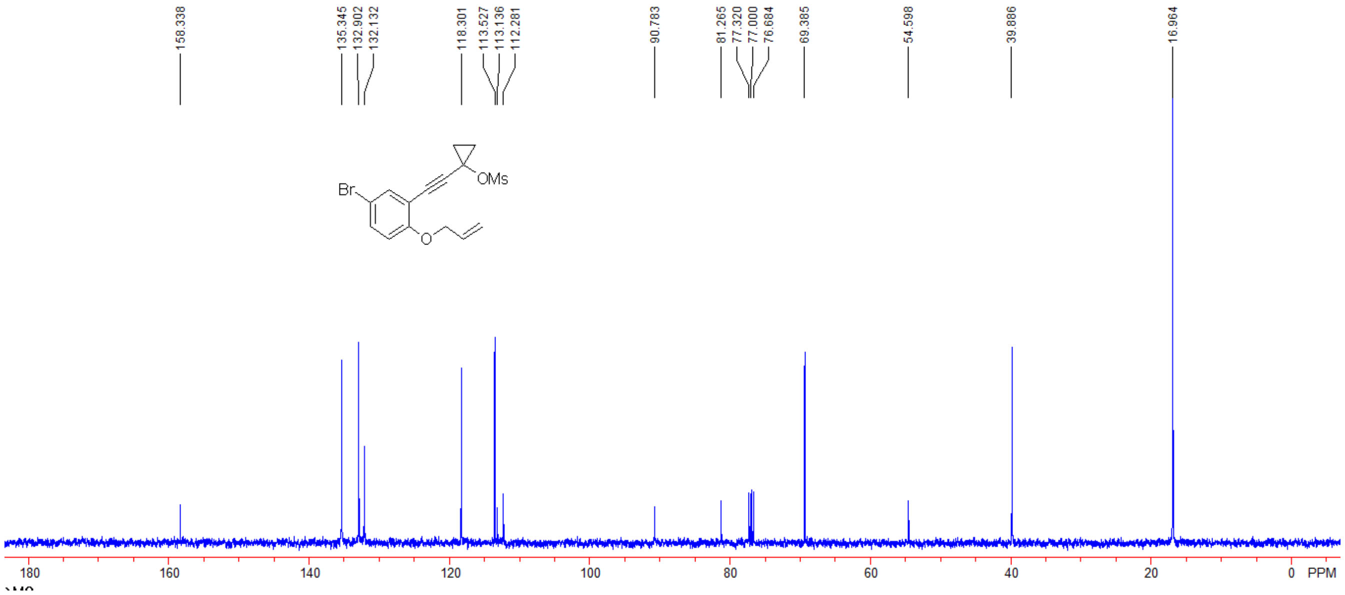

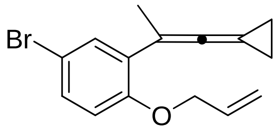

Compound **1k**. 719 mg, yield: 83%; colorless oil. ^{1}H NMR ($CDCl_3$, 400 MHz, TMS) δ 1.50-1.59 (m, 4H, CH_2), 2.14 (s, 3H, CH_3), 4.48 (d, J = 5.6 Hz, 2H, CH_2), 5.24-5.27 (m, 1H, $=CH_2$), 5.35-5.39 (m, 1H, $=CH_2$), 5.96-6.03 (m, 1H, =CH), 6.71 (d, J = 8.4 Hz, 1H, Ar), 7.22-7.25 (m, 1H, Ar), 7.35-7.36 (m, 1H, Ar). ^{13}C NMR ($CDCl_3$, 100 MHz, TMS) δ 7.9, 19.9, 69.5, 75.8, 99.4, 113.0,

114.1, 117.8, 130.0, 130.8, 131.4, 133.0, 155.0, 190.8. IR (neat) ν 2982, 2911, 2862, 1583, 1484, 1389, 1250, 1218, 1141, 1057, 1014, 926, 800, 778 cm^{-1}. MS (%) m/e 290 (M^+, 0.26), 251 (74.90), 249 (76.02), 170 (86.72), 169 (100.00), 155 (64.65), 142 (22.64), 141 (39.77), 115 (25.41). HRMS (EI) calcd. for $C_{15}H_{15}OBr$: 290.0306, Found: 290.0304.

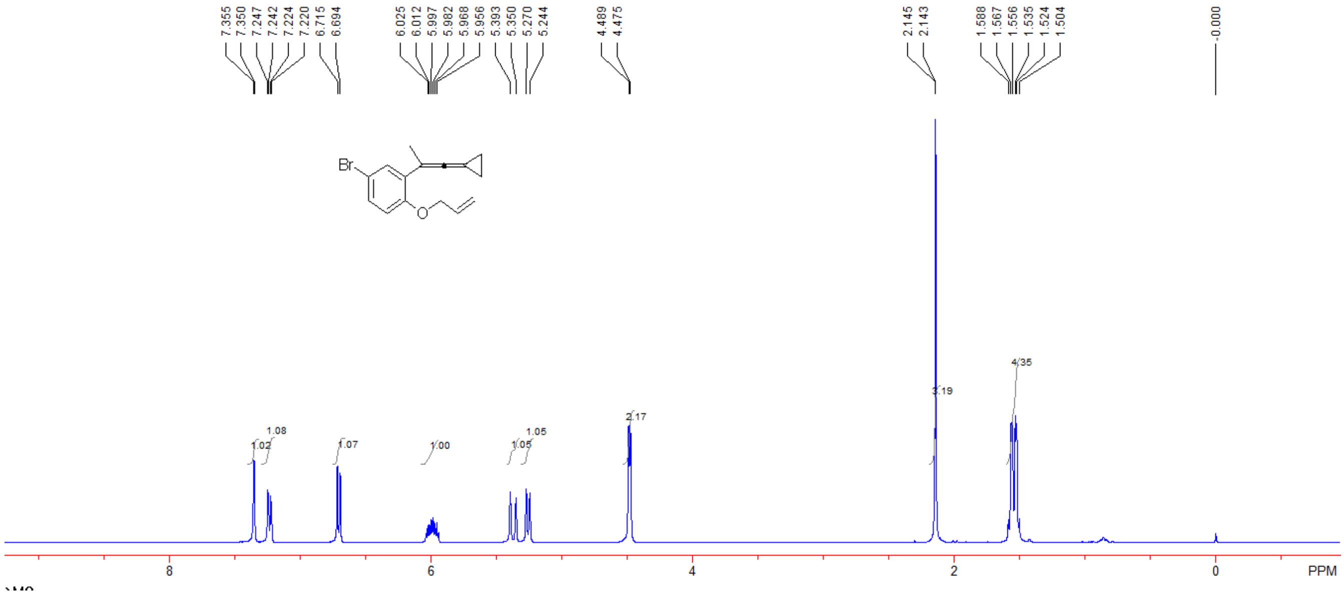

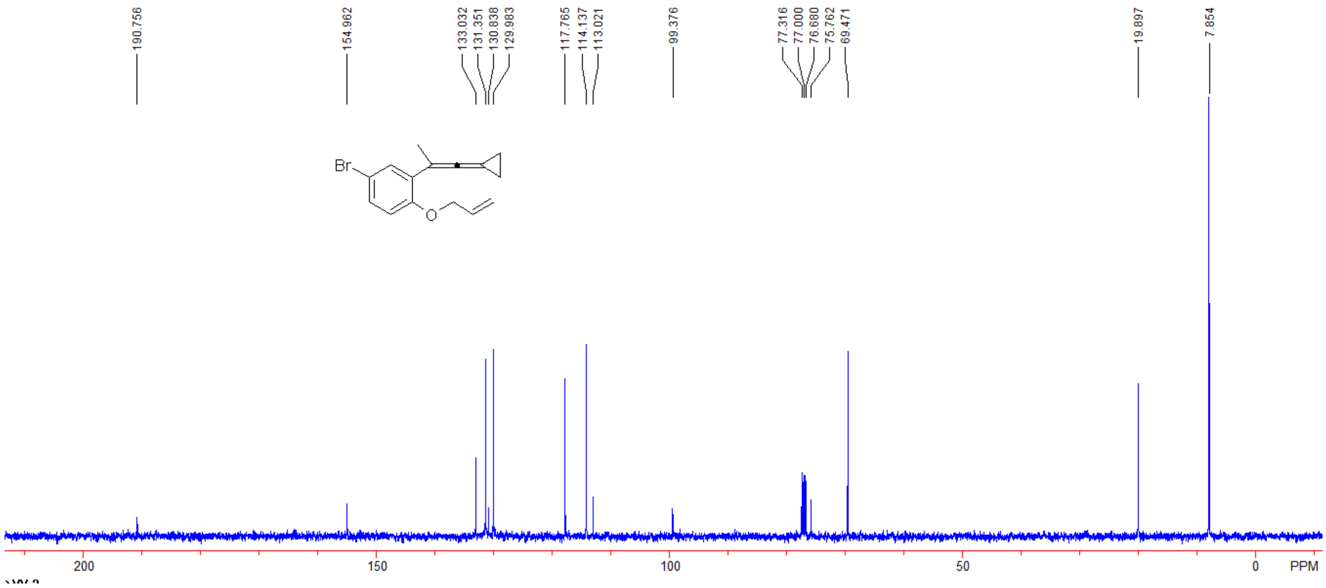

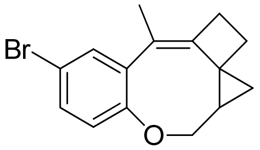

Compound **2k**. 126 mg, yield: 88%; colorless oil. ^{1}H NMR ($CDCl_3$, 400 MHz, TMS) δ 0.40 (dd, J_1 = 5.2 Hz, J_2 = 7.6 Hz, 1H, CH_2), 0.50 (dd, J_1 = 4.8 Hz, J_2 = 9.6 Hz, 1H, CH_2), 1.15-1.21 (m, 1H, CH), 1.91 (t, J = 1.6 Hz, 3H, CH_3), 2.11-2.25 (m, 2H, CH_2), 2.77-2.83 (m, 1H, CH_2), 2.95-3.04 (m,

1H, CH_2), 4.31 (d, J = 12.8 Hz, 1H, CH_2), 4.83 (dd, J_1 = 5.6 Hz, J_2 = 12.4 Hz, 1H, CH_2), 6.82 (d, J = 8.4 Hz, 1H, Ar), 7.25-7.27 (m, 1H, Ar), 7.33-7.34 (m, 1H, Ar). ^{13}C NMR ($CDCl_3$, 100 MHz, TMS) δ 15.5, 17.3, 24.4, 27.6, 27.7, 32.2, 70.8, 115.9, 118.1, 122.9, 129.7, 130.8, 136.9, 143.9, 154.8. IR (neat) ν 3060, 2913, 2869, 1475, 1392, 1283, 1233, 1208, 1092, 1055, 1005, 972, 906, 820, 727 cm^{-1}. MS (%) m/e 290 (M^+, 2.07), 231 (19.84), 230 (100.00), 202 (52.39), 201 (19.78), 200 (25.49), 101 (23.66), 86 (24.05), 84 (36.84). HRMS (EI) calcd. for $C_{15}H_{15}OBr$: 290.0306, Found: 290.0307; Enantiomeric excess was determined by HPLC with a Chiralcel PA-2 column [λ = 214 nm; eluent: Hexane/Isopropanol = 90/10; Flow rate: 0.60 mL/min; t_{minor} = 15.10 min, t_{major} = 16.70 min; ee% = 79%; $[\alpha]^{20}_D$ = -7.7684 (c 1.00, CH_2Cl_2)].

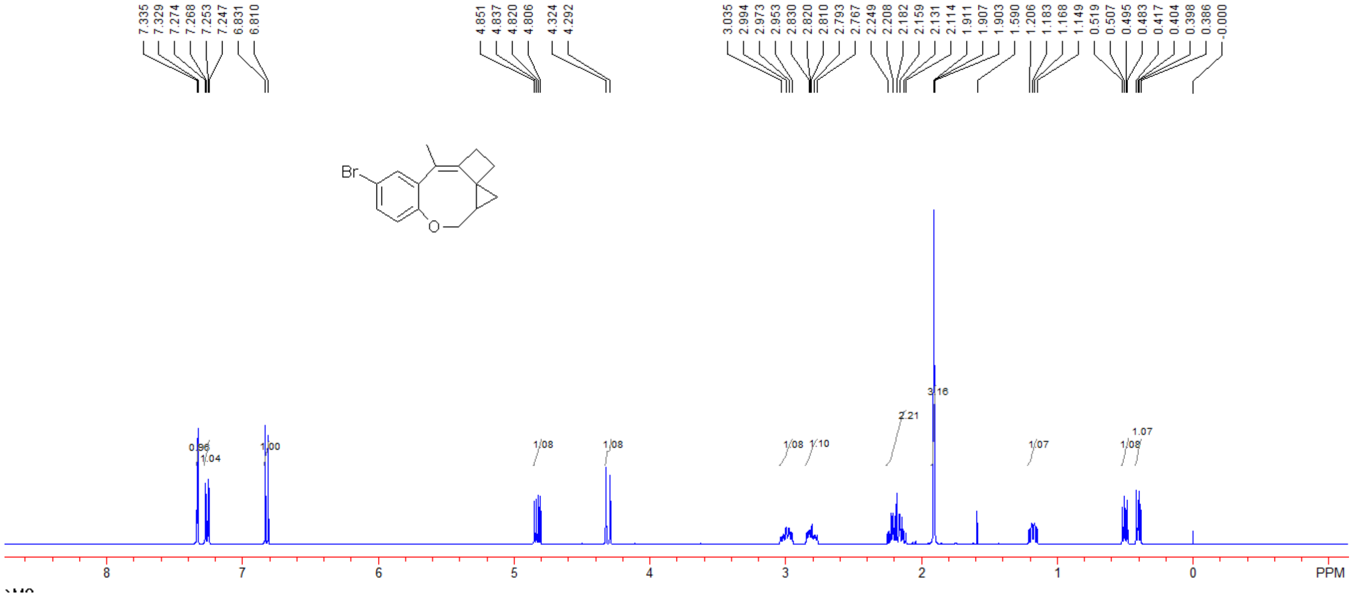

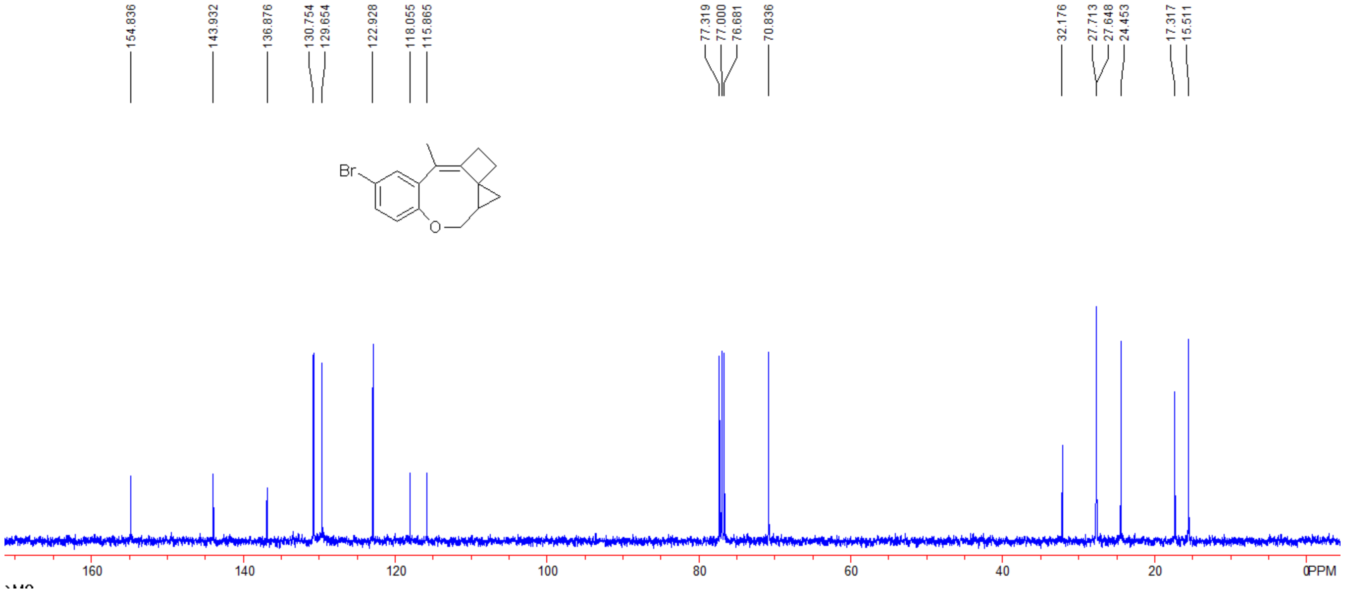

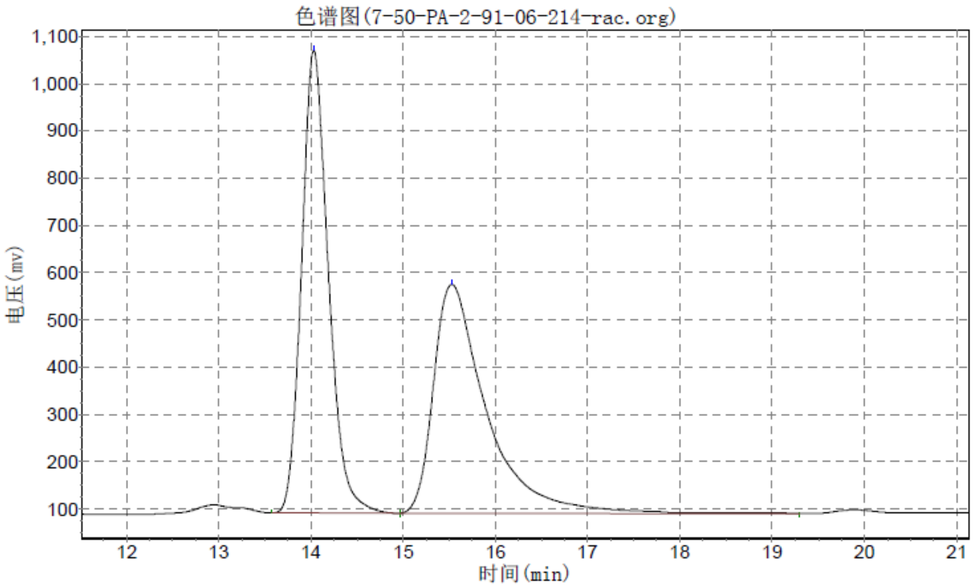

分析结果表

峰号	峰名	保留时间	峰高	峰面积	含量
1		14.030	978856.000	19723166.000	50.4125
2		15.527	484231.938	19400376.000	49.5875
总计			1463087.938	39123542.000	100.0000

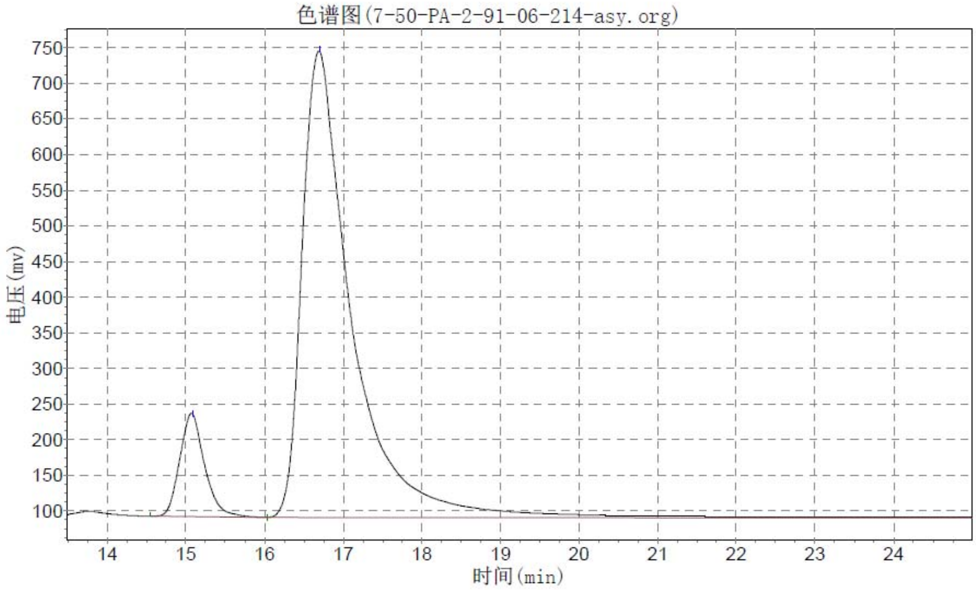

分析结果表

峰号	峰名	保留时间	峰高	峰面积	含量
1		15.098	146043.813	3361792.750	10.3420
2		16.698	654693.625	29144278.000	89.6580
总计			800737.438	32506070.750	100.0000

Translation: a Chiralcel PA-2 column [λ = 214 nm; eluent: Hexane/Isopropanol = 90/10; Flow rate: 0.60 mL/min; t_{minor} = 15.10 min, t_{major} = 16.70 min; ee% = 79%.]

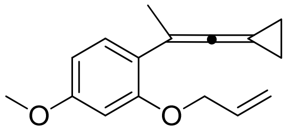

Compound **1l**. 414 mg, yield: 57%; colorless oil. ^{1}H NMR (CDCl$_3$, 400 MHz, TMS) δ 1.46-1.49 (m, 2H, CH$_2$), 1.51-1.55 (m, 2H, CH$_2$), 2.16 (s, 3H, CH$_3$), 3.78 (s, 3H, CH$_3$), 4.49 (d, J = 4.8 Hz, 2H, CH$_2$), 5.24-5.27 (m, 1H, =CH$_2$), 5.36-5.41 (m, 1H, =CH$_2$), 5.96-6.06 (m, 1H, =CH), 6.44-6.48 (m, 2H, Ar), 7.16-7.18 (m, 1H, Ar). ^{13}C NMR (CDCl$_3$, 100 MHz, TMS) δ 7.4, 20.2, 55.3, 69.3, 75.3, 100.01, 100.02, 104.6, 117.5, 121.3, 129.3, 133.4, 156.8, 159.5, 190.2. IR (neat) ν 3076, 2982, 2909, 2010, 1606, 1577, 1505, 1420, 1304, 1262, 1200, 1167, 1036, 928, 831 cm^{-1}. MS (%) m/e 202 (14.65), 201 (100.00), 186 (12.79), 171 (19.82), 158 (19.69), 141 (14.18), 128 (17.99), 115 (27.43). HRMS (EI) calcd. for $C_{16}H_{18}O_2$: 242.1307, Found: 242.1310.

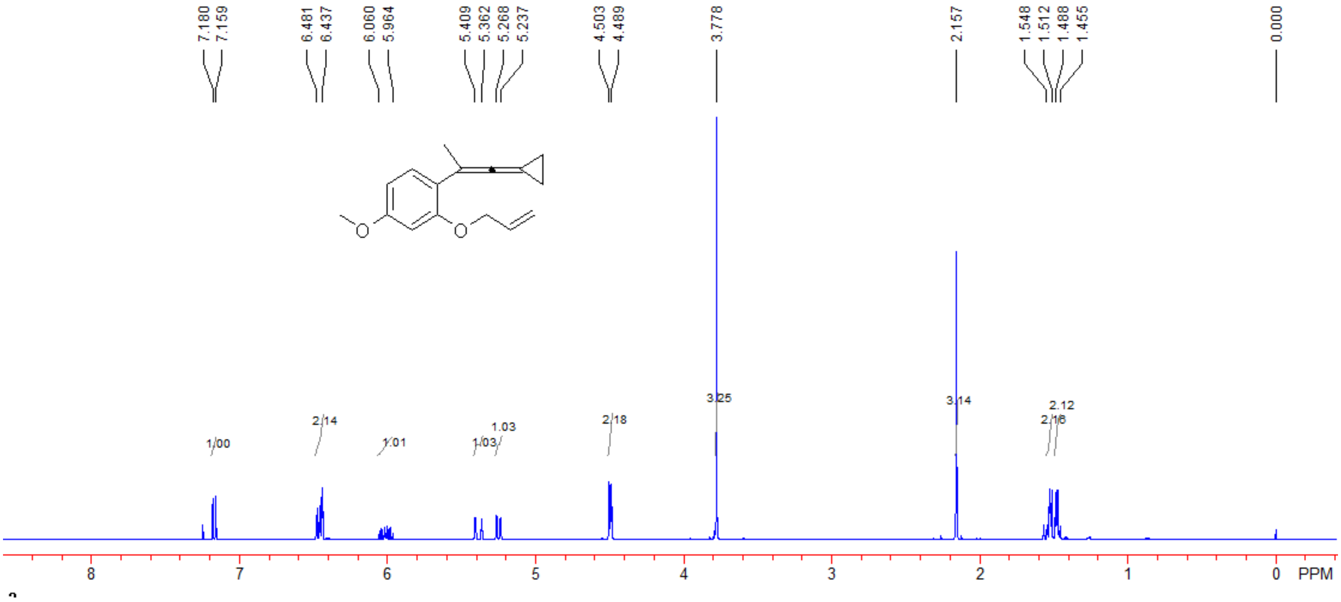

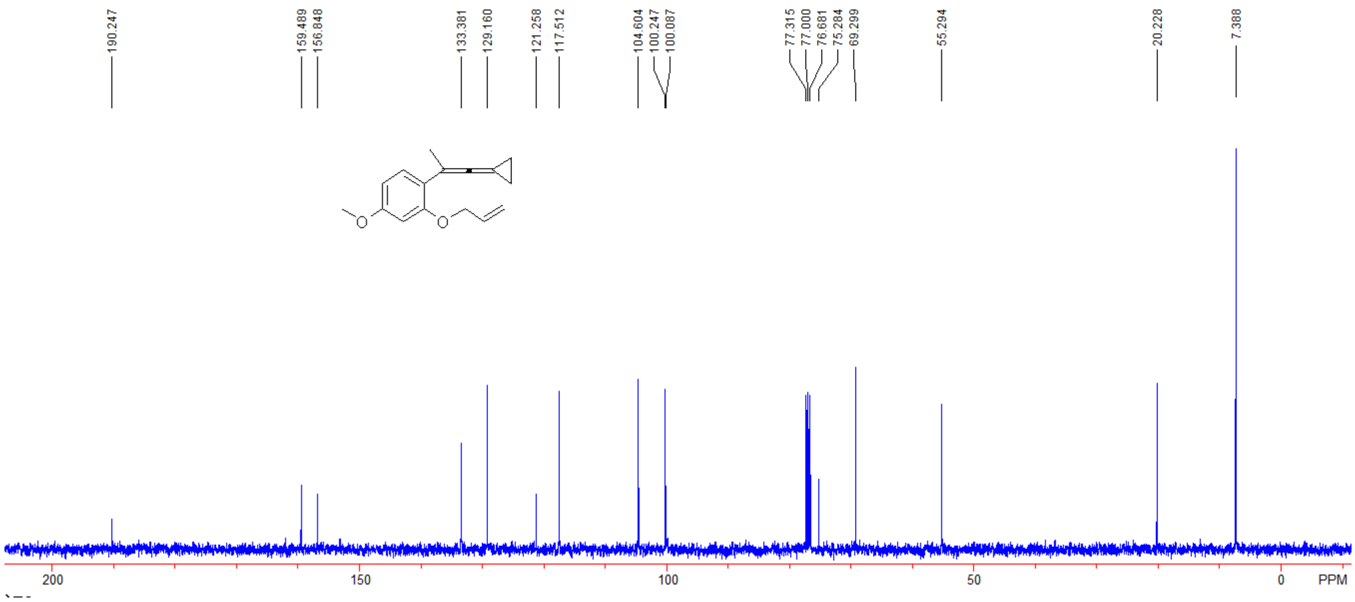

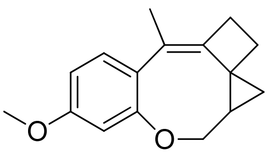

Compound **2l**. 57 mg, yield: 47%; yellow oil. ^{1}H NMR ($CDCl_3$, 400 MHz, TMS) δ 0.35 (dd, J_1 = 4.8 Hz, J_2 = 7.6 Hz, 1H, CH_2), 0.47 (dd, J_1 = 4.8 Hz, J_2 = 9.6 Hz, 1H, CH_2), 1.15-1.20 (m, 1H, CH), 1.92 (s, 3H, CH_3), 2.12-2.24 (m, 2H, CH_2), 2.77-2.85 (m, 1H, CH_2), 2.96-3.04 (m, 1H, CH_2), 3.78 (s, 3H, CH_3), 4.34 (d, J = 12.8 Hz, 1H, CH_2), 4.81 (dd, J_1 = 5.6 Hz, J_2 = 12.0 Hz, 1H, CH_2), 6.53 (d, J = 2.4 Hz, 1H, Ar), 6.58-6.61 (m, 1H, Ar), 7.13-7.15 (m, 1H, Ar). ^{13}C NMR ($CDCl_3$, 100 MHz, TMS) δ 15.2, 17.5, 24.7, 27.6, 27.8, 32.2, 55.3, 70.6, 77.2, 106.2, 109.1, 119.0, 127.4, 127.5, 140.6, 156.5, 159.6. IR (neat) ν 3060, 2929, 2855, 1606, 1567, 1500, 1440, 1287, 1246, 1159, 1147, 1092, 1007, 813 cm^{-1}. MS (%) m/e 242 (M^+, 7.34), 227 (25.63), 213 (31.40), 212 (100.00), 201 (24.63), 197 (26.02), 175 (23.85), 128 (22.56), 115 (27.63). HRMS (EI) calcd. for $C_{16}H_{18}O_2$: 242.1307, Found: 242.1311.

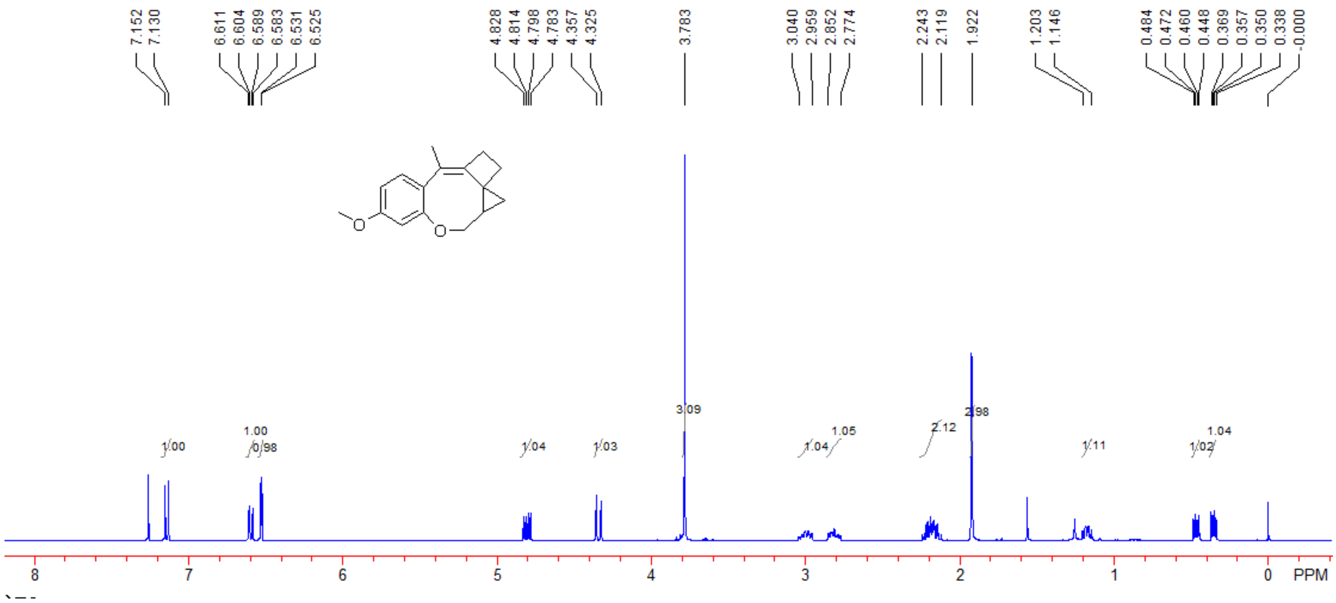

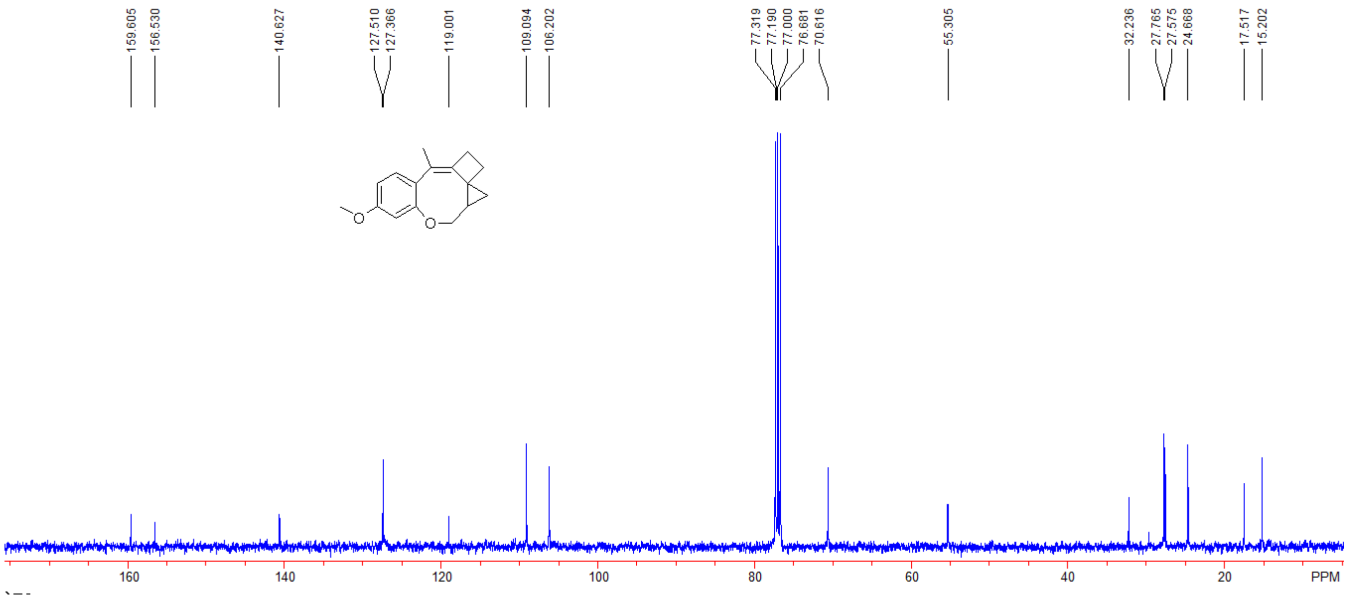

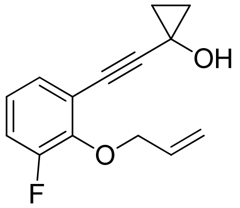

Compound **S3m**. 1.712 g, yield: 64%; yellow oil. ^{1}H NMR ($CDCl_3$, 400 MHz, TMS) δ 1.11-1.14 (m, 2H, CH_2), 1.21-1.24 (m, 2H, CH_2), 3.21 (br, 1H, OH), 4.68-4.70 (m, 2H, CH_2), 5.26-5.28 (m, 1H, =CH_2), 5.40-5.45 (m, 1H, =CH_2), 6.06-6.15 (m, 1H, =CH), 6.93-6.98 (m, 1H, Ar), 7.03-7.07 (m, 1H, Ar), 7.14-7.17 (m, 1H, Ar). ^{13}C NMR ($CDCl_3$, 100 MHz, TMS) δ 17.6, 45.9, 74.7 (d, J_{C-F} = 3.8 Hz), 77.55 (d, J_{C-F} = 4.5 Hz), 96.1, 116.8 (d, J_{C-F} = 19.7 Hz), 118.3, 118.9 (d, J_{C-F} = 3.0 Hz), 123.6

(d, $J_{C\text{-}F}$ = 8.3 Hz), 128.3 (d, $J_{C\text{-}F}$ = 3.0 Hz), 133.4, 147.1 (d, $J_{C\text{-}F}$ = 12.2 Hz), 155.5 (d, $J_{C\text{-}F}$ = 245.2 Hz). ^{19}F NMR (376 MHz, $CDCl_3$, $CFCl_3$): δ -129.86 ~ -129.82 (m, 1F). IR (neat) ν 3364, 3087, 3012, 2933, 2229, 1575, 1465, 1421, 1261, 1215, 1066, 969, 882, 785, 728 cm^{-1}. MS (%) m/e 232 (M^+, 2.14), 175 (54.30), 163 (72.28), 162 (39.19), 149 (100.00), 133 (51.42), 115 (50.21), 107 (52.34), 55 (63.96). HRMS (EI) calcd. for $C_{14}H_{12}O_2F[M\text{-}H]^+$: 231.0821, Found: 231.0825.

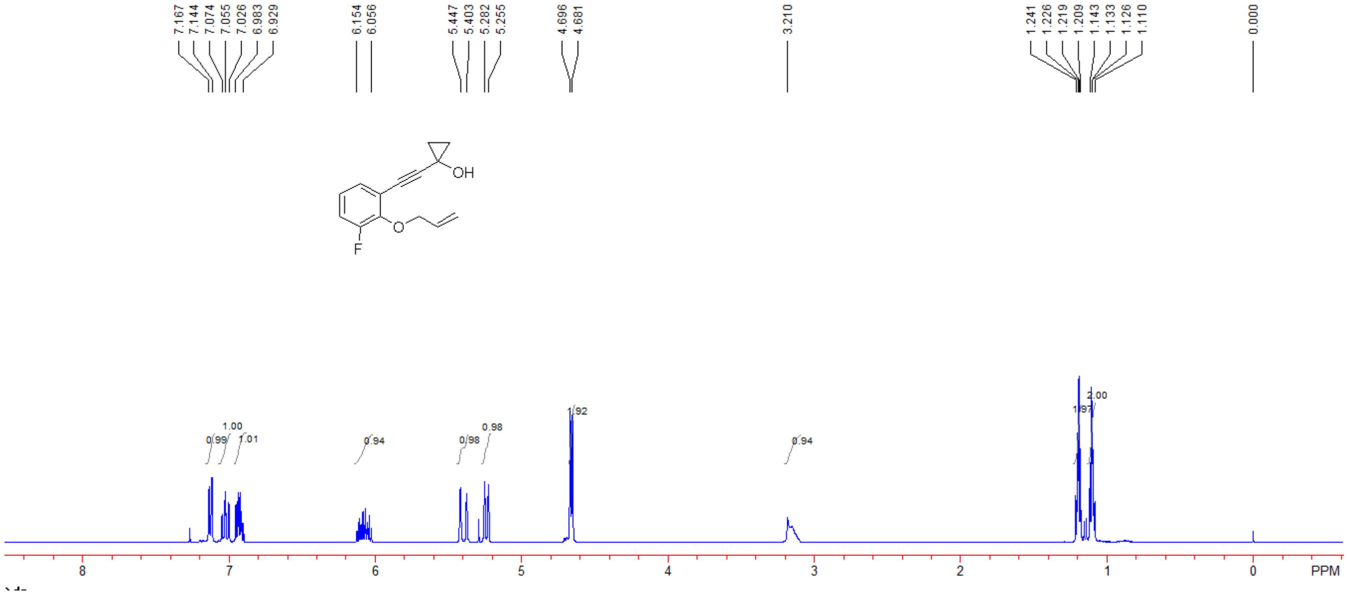

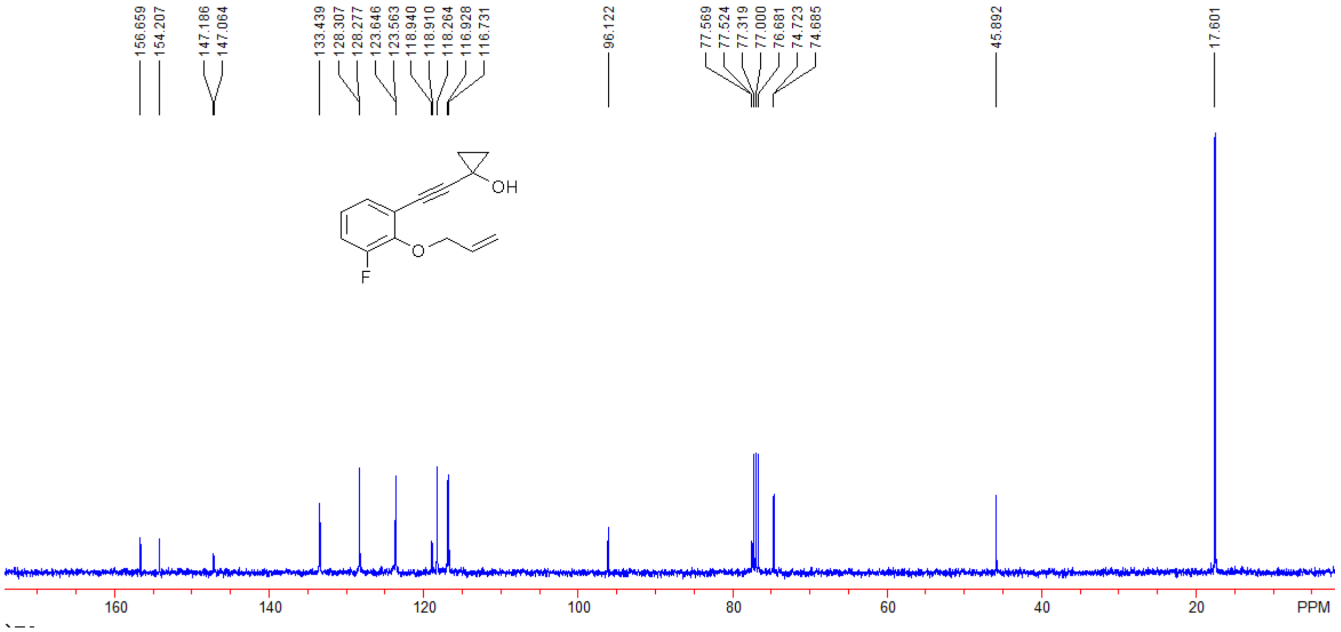

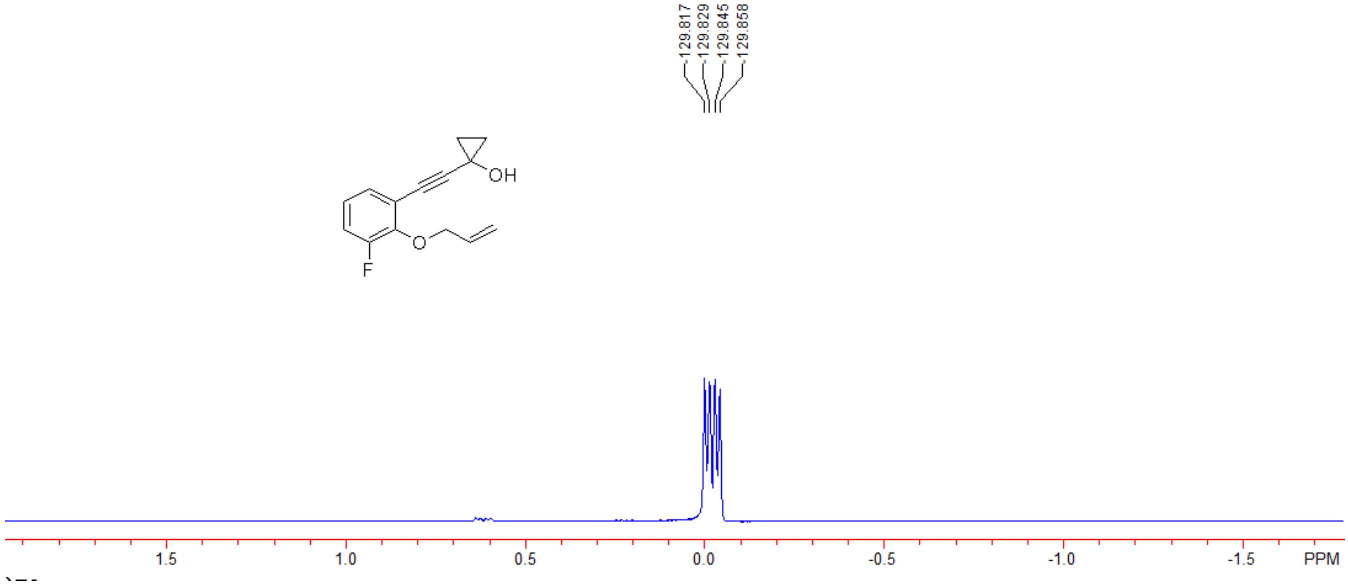

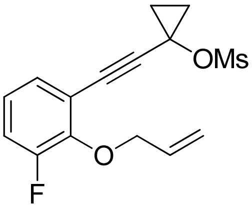

Compound **S4m**. 1.548 g, yield: 72%; yellow oil. ^{1}H NMR ($CDCl_3$, 400 MHz, TMS) δ 1.35-1.39 (m, 2H, CH_2), 1.65-1.69 (m, 2H, CH_2), 3.28 (s, 3H, CH_3), 4.67-4.69 (m, 2H, CH_2), 5.27 (dd, J_1 = 1.2 Hz, J_2 = 10.4 Hz, 1H, =CH_2), 5.40 (dd, J_1 = 1.6 Hz, J_2 = 17.2 Hz, 1H, =CH_2), 6.03-6.13 (m, 1H, =CH), 6.94-7.00 (m, 1H, Ar), 7.07-7.15 (m, 2H, Ar). ^{13}C NMR ($CDCl_3$, 100 MHz, TMS) δ 16.9, 39.8, 54.4, 74.8 (d, J_{C-F} = 4.8 Hz), 81.6 (d, J_{C-F} = 4.8 Hz), 90.4, 117.5 (d, J_{C-F} = 3.8 Hz), 117.8 (d, J_{C-F} = 19.3 Hz), 118.7, 123.7 (d, J_{C-F} = 8.2 Hz), 128.3 (d, J_{C-F} = 3.0 Hz), 133.1, 147.4 (d, J_{C-F} = 11.9 Hz), 155.2 (d, J_{C-F} = 245.8 Hz). ^{19}F NMR (376 MHz, $CDCl_3$, $CFCl_3$): δ -126.69 ~ -126.65 (m, 1F). IR (neat) ν 3076, 3018, 2933, 2227, 1467, 1421, 1361, 1264, 1166, 1067, 975, 939, 893, 787 cm^{-1}. MS (%) m/e 310 (M^+, 2.89), 199 (31.74), 189 (38.95), 162 (38.84), 149 (31.13), 146 (100.00), 134 (33.08), 133 (39.76), 41 (35.33). HRMS (EI) calcd. for $C_{15}H_{15}O_4FS$: 310.0675, Found: 310.0672.

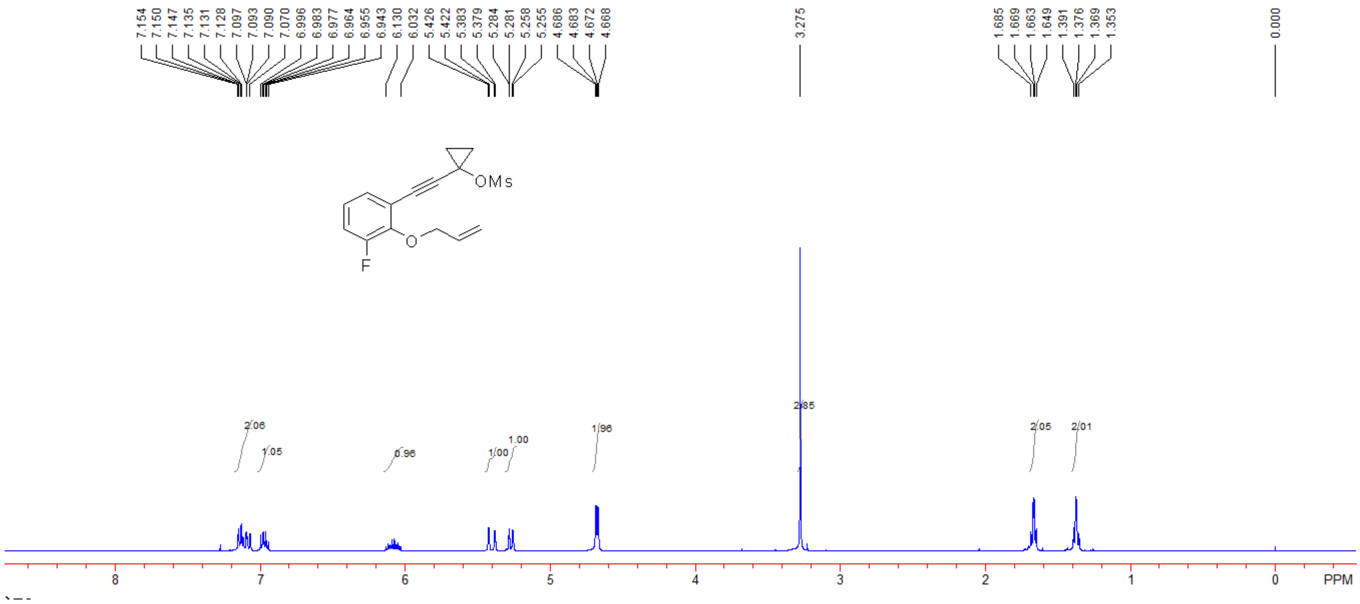
7.154
7.150
7.147
7.135
7.131
7.128
7.097
7.093
7.090
7.070
6.996
6.983
6.977
6.964
6.955
6.943
6.130
6.032
5.426
5.422
5.383
5.379
5.284
5.281
5.258
5.255
4.686
4.683
4.672
4.668
3.275
1.685
1.669
1.663
1.649
1.391
1.376
1.369
1.353
0.000
OMs
O
F
2.06
1.05
0.96
1.00
1.00
1.96
2.85
2.05
2.01
PPM

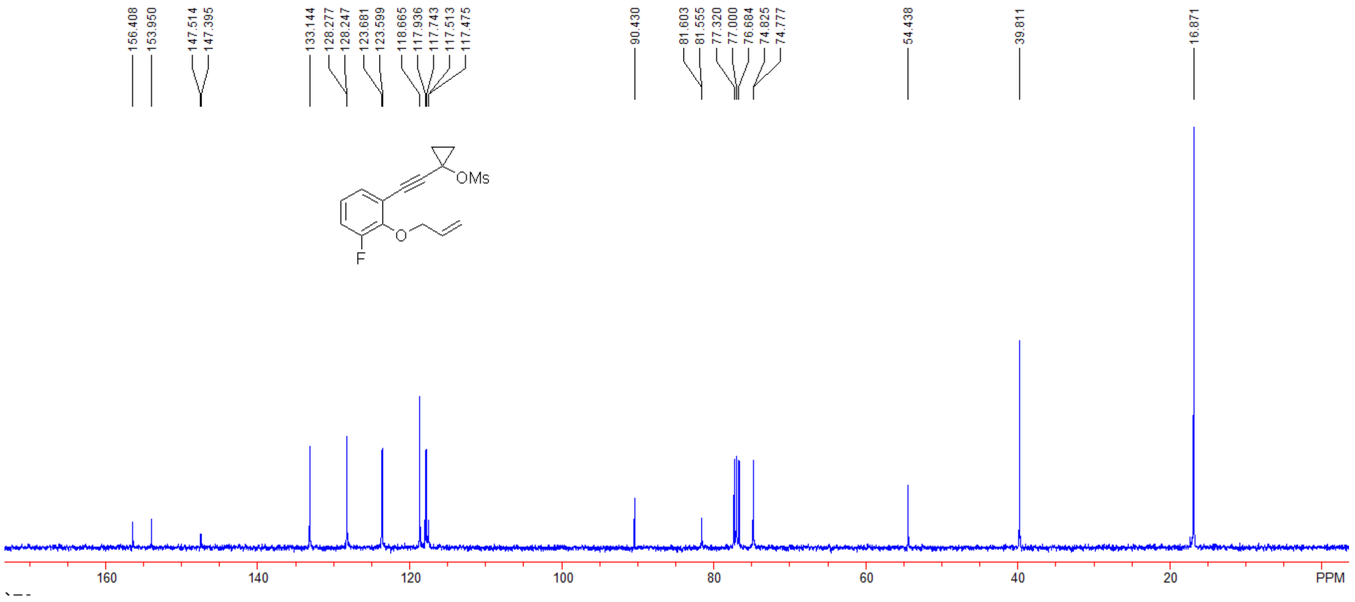
156.408
153.950
147.514
147.395
133.144
128.277
128.247
123.681
123.599
118.665
117.936
117.743
117.513
117.475
90.430
81.603
81.555
77.320
77.000
76.684
74.825
74.777
54.438
39.811
16.871
OMs
O
F
PPM

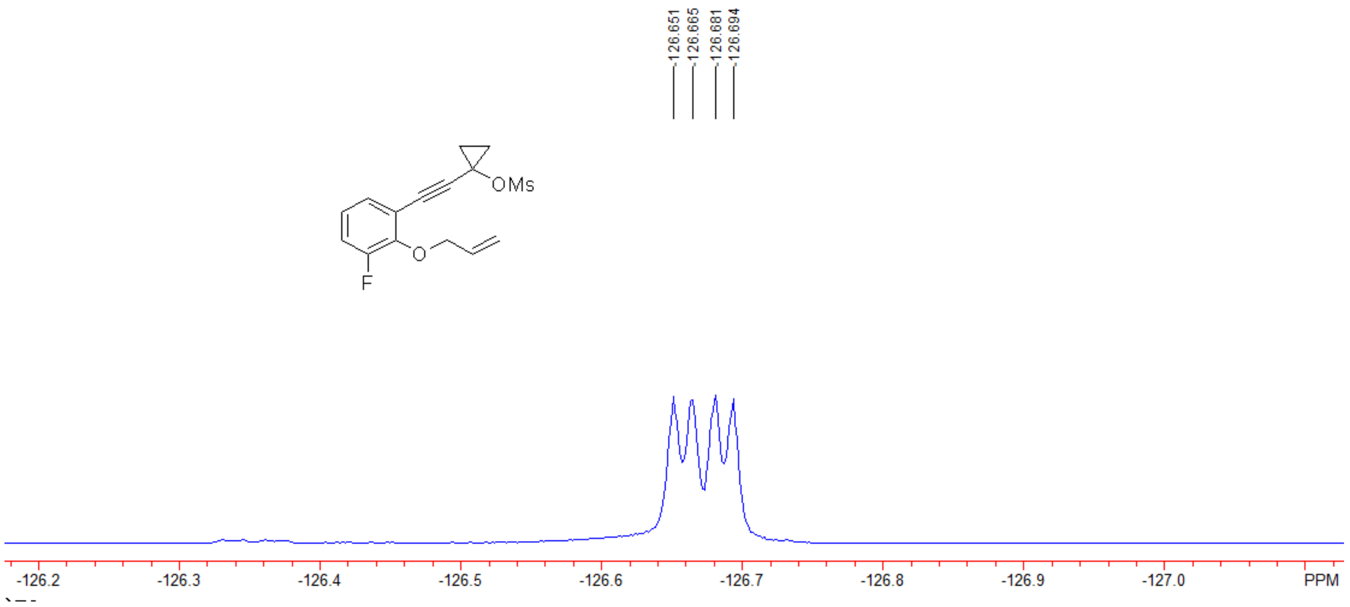
-126.651
-126.665
-126.681
-126.694
OMs
O
F
PPM

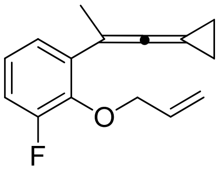

Compound **1m**. 350 mg, yield: 65%; yellow oil. ^{1}H NMR ($CDCl_3$, 400 MHz, TMS) δ 1.55-1.57 (m, 4H, $2CH_2$), 2.16 (s, 3H, CH_3), 4.50-4.51 (m, 2H, CH_2), 5.19-5.22 (m, 1H, $=CH_2$), 5.31-5.36 (m, 1H, $=CH_2$), 6.01-6.10 (m, 1H, =CH), 6.91-6.70 (m, 2H, Ar), 7.03-7.05 (m, 1H, Ar). ^{13}C NMR ($CDCl_3$, 100 MHz, TMS) δ 7.8, 20.1, 74.3 (d, $J_{C\text{-}F}$ = 4.6 Hz), 75.8, 99.2 (d, $J_{C\text{-}F}$ = 3.0 Hz), 114.6, 114.8, 117.6, 123.5 (d, $J_{C\text{-}F}$ = 4.4 Hz), 123.6 (d, $J_{C\text{-}F}$ = 3.0 Hz), 133.9, 134.9, 143.8 (d, $J_{C\text{-}F}$ = 12.2 Hz), 156.2 (d, $J_{C\text{-}F}$ = 244.4 Hz), 190.8. ^{19}F NMR (376 MHz, $CDCl_3$, $CFCl_3$): δ -130.26 ~ -130.22 (m, 1F). IR (neat) ν 3066, 2984, 2916, 2009, 1577, 1463, 1421, 1264, 1252, 1217, 1166, 985, 885, 785 cm^{-1}. MS (%) m/e 230 (M^+, 0.66), 190 (14.19), 189 (100.00), 149 (17.65), 146 (55.41), 141 (38.74), 133 (21.59), 115 (18.54), 43 (13.91). HRMS (EI) calcd. for $C_{15}H_{15}OF$: 230.1107, Found: 230.1103.

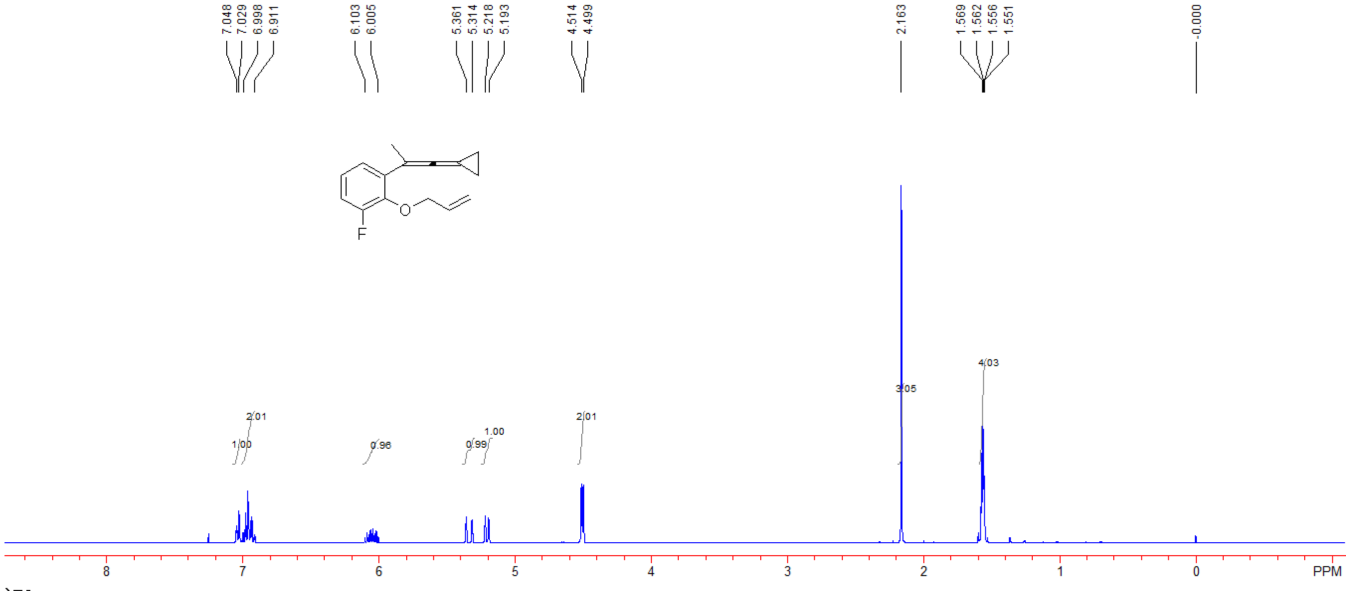

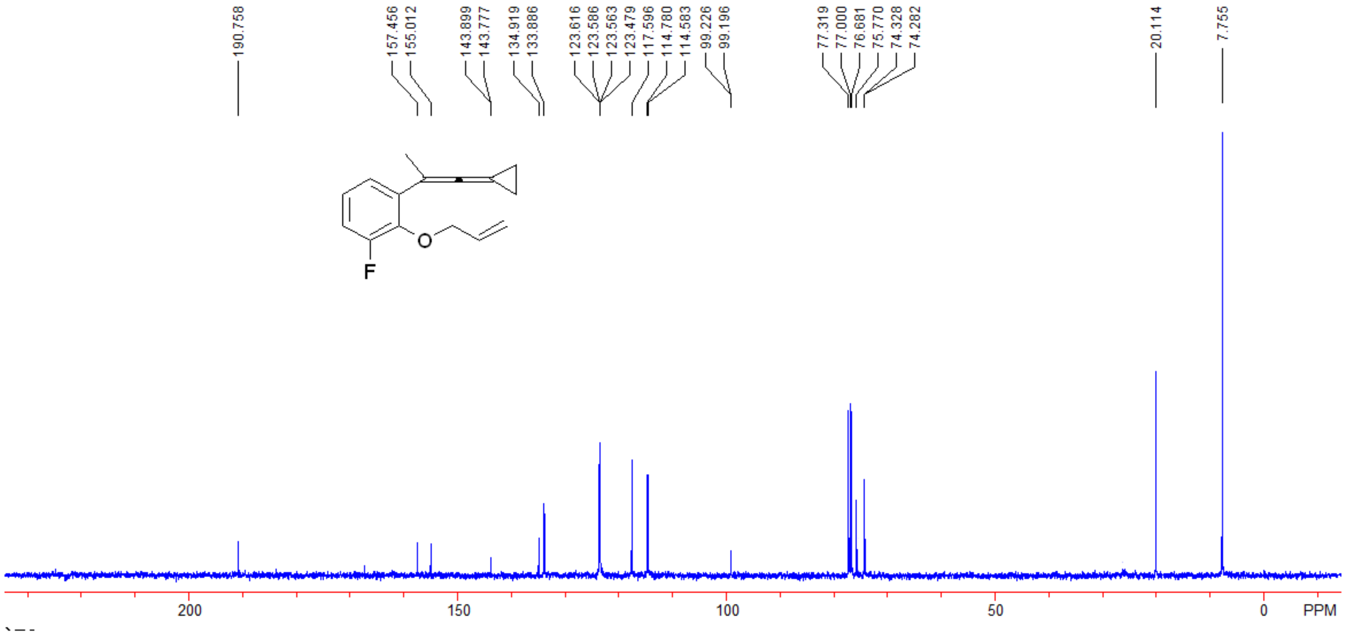

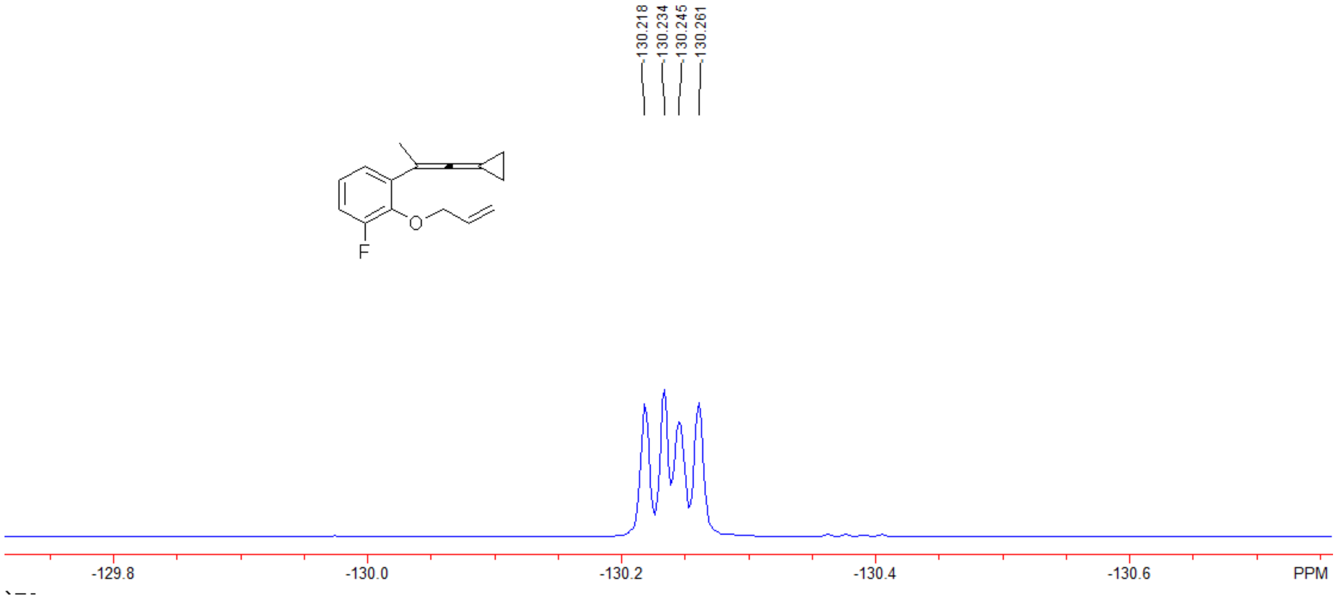

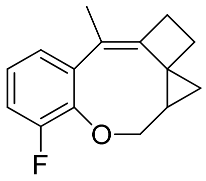

Compound **2m**. 81 mg, yield: 70%; yellow oil. ^{1}H NMR ($CDCl_3$, 400 MHz, TMS) δ 0.44 (dd, J_1 = 4.8 Hz, J_2 = 7.6 Hz, 1H, CH_2), 0.55 (dd, J_1 = 4.8 Hz, J_2 = 9.6 Hz, 1H, CH_2), 1.20-1.23 (m, 1H, CH), 1.92 (t, J = 1.6 Hz, 3H, CH_3), 2.13-2.27 (m, 2H, CH_2), 2.79-2.86 (m, 1H, CH_2), 2.97-3.05 (m, 1H, CH_2), 4.45 (d, J = 12.8 Hz, 1H, CH_2), 4.79 (dd, J_1 = 6.0 Hz, J_2 = 12.8 Hz, 1H, CH_2), 6.89-7.00 (m, 3H, Ar). ^{13}C NMR ($CDCl_3$, 100 MHz, TMS) δ 16.0, 17.6, 24.4, 27.7 (d, $J_{C\text{-}F}$ = 2.3 Hz), 32.3, 69.3 (d,

$J_{C\text{-}F}$ = 3.8 Hz), 114.1 (d, $J_{C\text{-}F}$ = 19.0 Hz), 118.5, 121.9 (d, $J_{C\text{-}F}$ = 3.0 Hz), 123.5 (d, $J_{C\text{-}F}$ = 8.3 Hz), 137.5, 143.2 (d, $J_{C\text{-}F}$ = 11.4 Hz), 143.5, 155.9 (d, $J_{C\text{-}F}$ = 245.1 Hz). ^{19}F NMR (376 MHz, $CDCl_3$, $CFCl_3$): δ -136.66 ~ -136.62 (m, 1F). IR (neat) ν 2929, 2881, 1679, 1573, 1467, 1452, 1292, 1247, 1212, 1172, 1021, 972, 920, 790, 749 cm^{-1}. MS (%) m/e 230 (M^+, 19.25), 215 (32.46), 201 (33.88), 200 (100.00), 199 (21.85), 189 (24.87), 185 (46.00), 163 (24.43), 133 (20.49). HRMS (EI) calcd. for $C_{15}H_{15}OF$: 230.1107, Found: 230.1105.

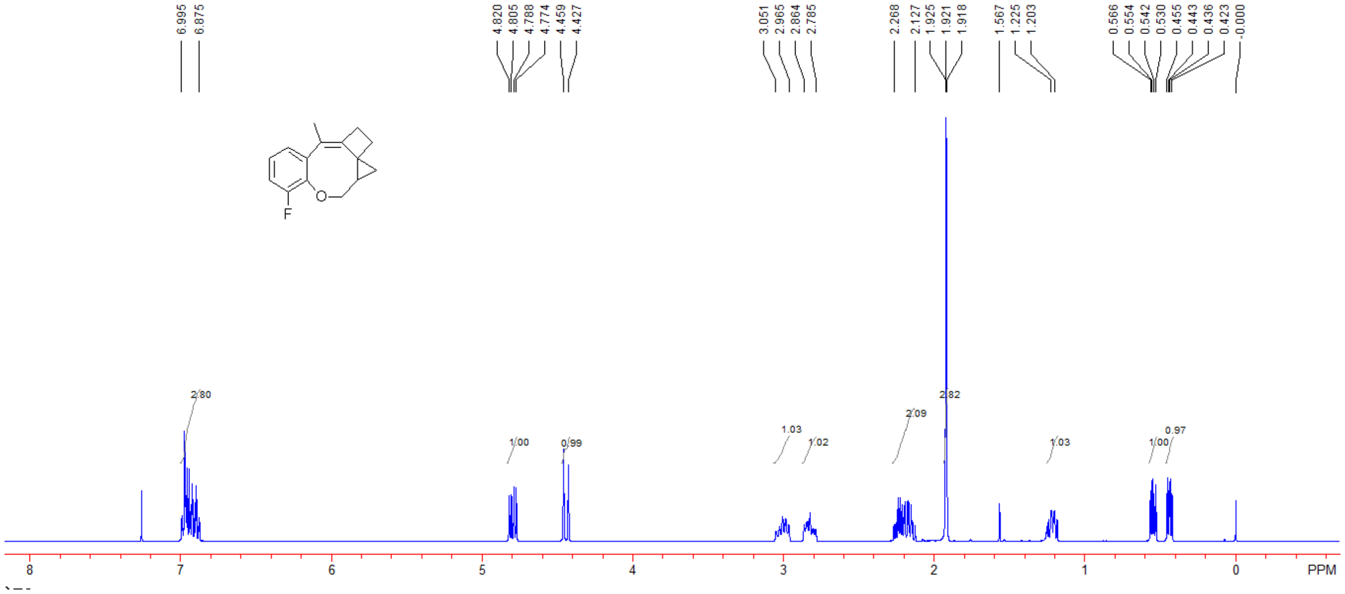

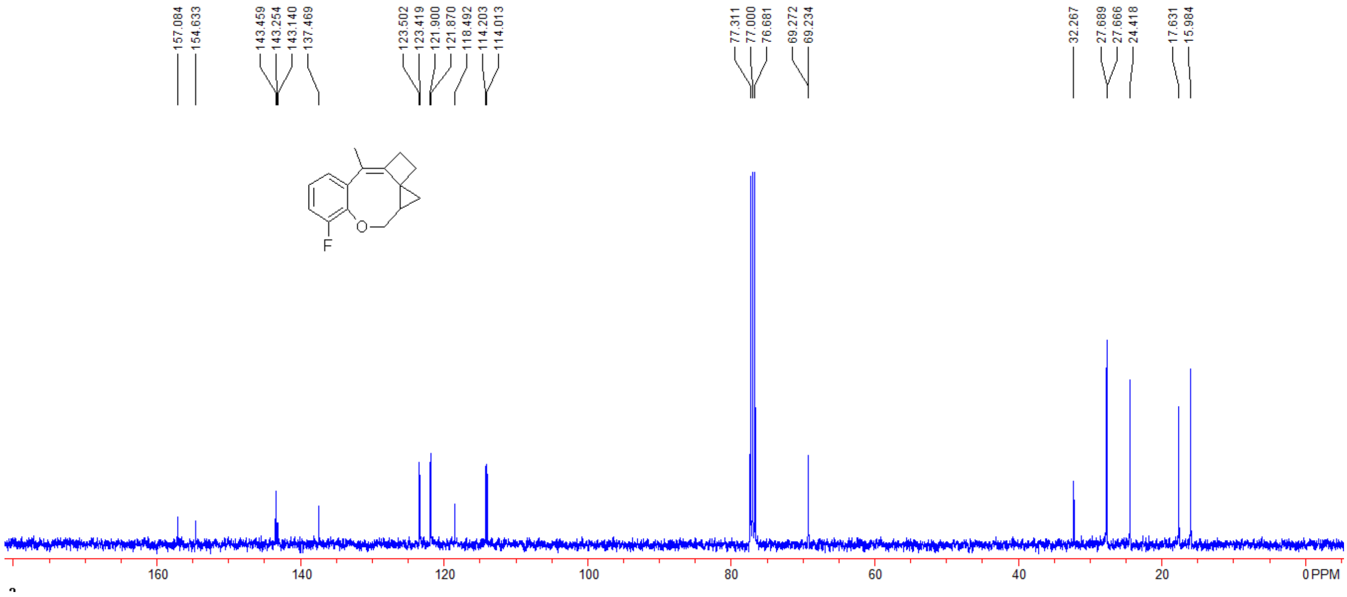

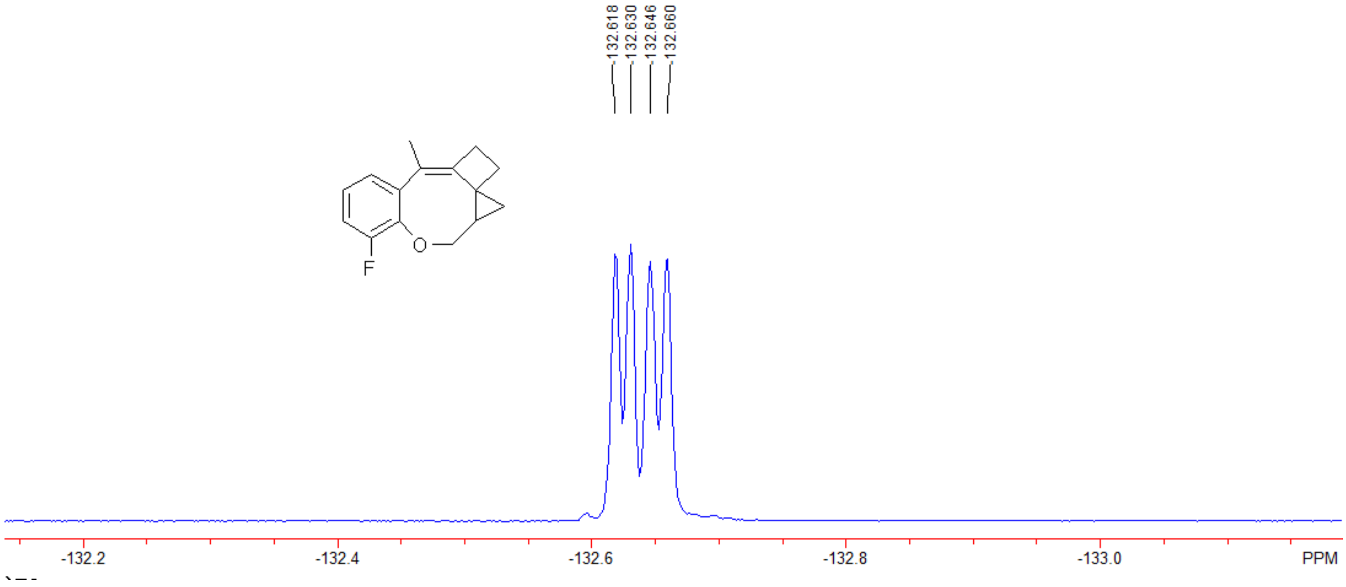

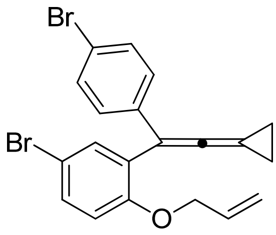

Compound **1n**. 230 mg, yield: 27%; pink solid. MP: 110-111 ^{o}C ^{1}H NMR ($CDCl_3$, 400 MHz, TMS) δ 1.68-1.72 (m, 2H, CH_2), 1.75-1.79 (m, 2H, CH_2), 4.40-4.41 (m, 2H, CH_2), 5.05-5.10 (m, 2H, $=CH_2$), 5.68-5.77 (m, 1H, =CH), 6.78 (d, J = 8.4 Hz, 1H, Ar), 7.08 (d, J = 8.0 Hz, 2H, Ar), 7.35-7.38 (m, 3H, Ar), 7.41-7.42 (m, 1H, Ar). ^{13}C NMR ($CDCl_3$, 100 MHz, TMS) δ 9.2, 69.2, 79.2, 106.3, 113.1, 114.5, 117.0, 119.9, 128.2, 128.5, 131.0, 131.4, 132.5, 134.1, 136.8, 155.2, 190.1. IR (neat) ν 3068, 2986, 2862, 2003, 1485, 1409, 1262, 1235, 1129, 1071, 1008, 927, 826, 804, 715 cm^{-1}. MS (%) m/e 430 (M^+, 1.08), 312 (62.40), 311 (38.30), 310 (68.04), 309 (25.33), 232 (27.48), 231 (43.78), 202 (62.68), 109 (100.00). HRMS (EI) calcd. for $C_{20}H_{16}OBr_2$: 429.9568, Found: 429.9570.

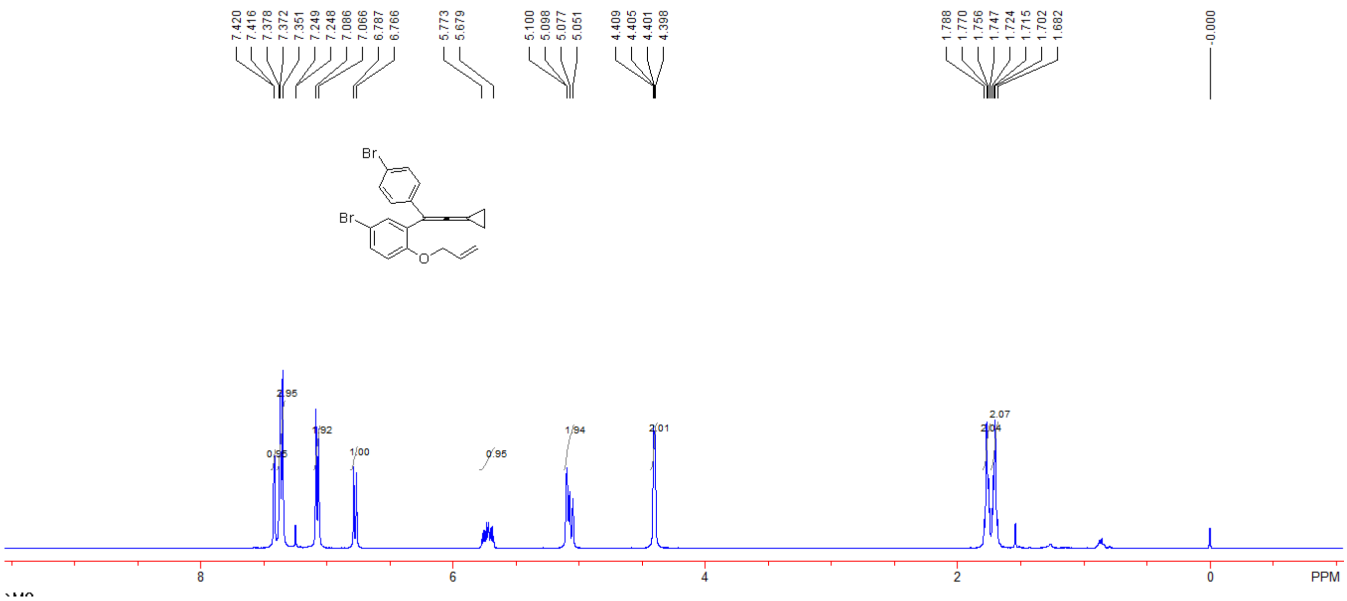

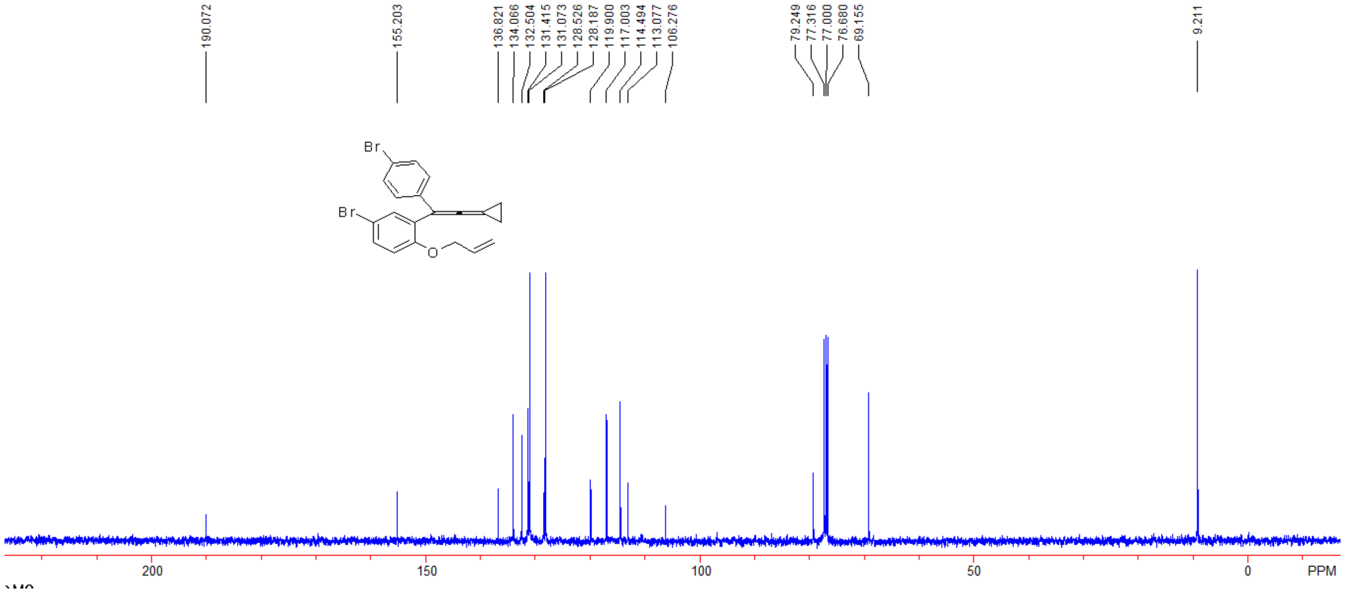

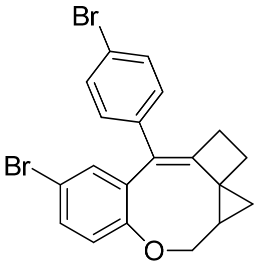

Compound **2n**. 153 mg, yield: 71%; pink solid. MP: 129-130 °C ^{1}H NMR ($CDCl_3$, 400 MHz, TMS) δ 0.52 (dd, J_1 = 5.2 Hz, J_2 = 7.6 Hz, 1H, CH_2), 0.66 (dd, J_1 = 5.2 Hz, J_2 = 9.6 Hz, 1H, CH_2), 1.23-1.29 (m, 1H, CH), 2.18-2.32 (m, 2H, CH_2), 2.88-2.96 (m, 1H, CH_2), 3.15-3.23 (m, 1H, CH_2), 4.36 (d, J = 12.0 Hz, 1H, CH_2), 4.85 (dd, J_1 = 6.0 Hz, J_2 = 12.4 Hz, 1H, CH_2), 6.90 (d, J = 8.8 Hz,

1H, Ar), 6.94 (d, J = 2.0 Hz, 1H, Ar), 7.09 (d, J = 8.4 Hz, 2H, Ar), 7.29-7.32 (m, 1H, Ar), 7.43 (d, J = 8.0 Hz, 2H, Ar). ^{13}C NMR ($CDCl_3$, 100 MHz, TMS) δ 15.8, 26.3, 28.1, 28.6, 33.1, 70.5, 115.8, 120.3, 122.9, 123.9, 130.9, 131.3, 131.7, 132.3, 135.9, 139.5, 147.8, 155.8. IR (neat) ν 3060, 2960, 2869, 1645, 1476, 1389, 1249, 1213, 1123, 1070, 982, 948, 906, 821, 729 cm^{-1}. MS (%) m/e 430 (M^+, 0.81), 242 (75.60), 215 (53.98), 202 (100.00), 189 (65.95), 176 (63.47), 165 (62.82), 113 (62.02), 101 (61.49). HRMS (EI) calcd. for $C_{20}H_{16}OBr_2$: 429.9568, Found: 429.9572; Enantiomeric excess was determined by HPLC with a Chiralcel AD-H column [λ = 214 nm; eluent: Hexane/Isopropanol = 99/1; Flow rate: 0.60 mL/min; t_{minor} = 22.31 min, t_{major} = 19.27 min; ee% = 69%; $[\alpha]^{20}_D$ = -2.3493 (c 0.73, CH_2Cl_2)].

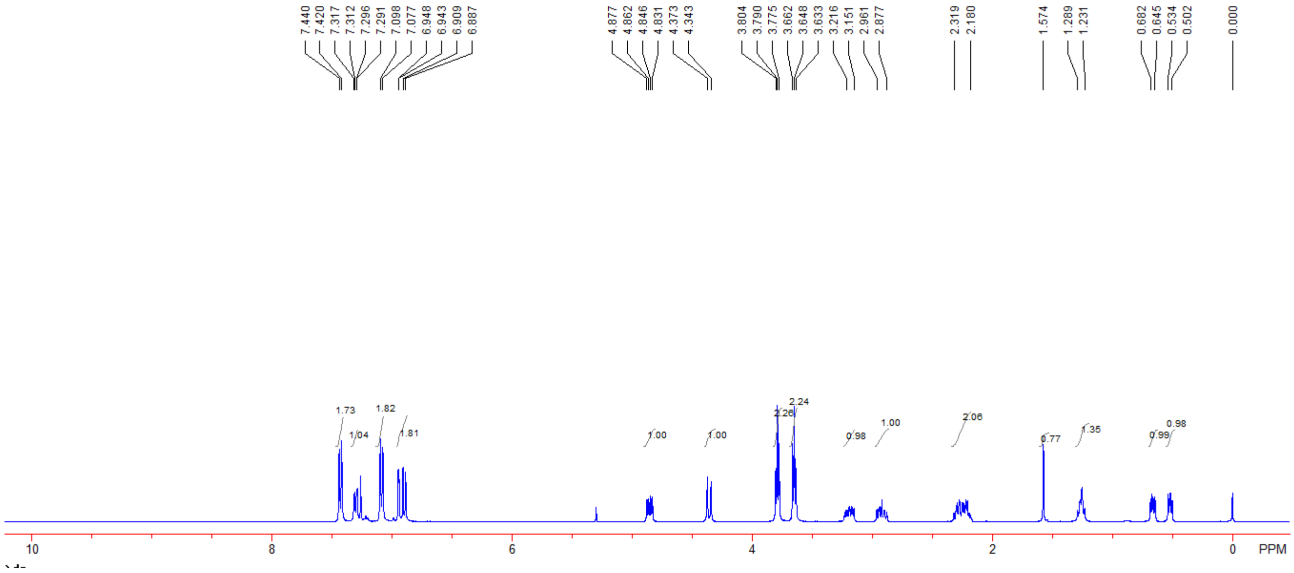

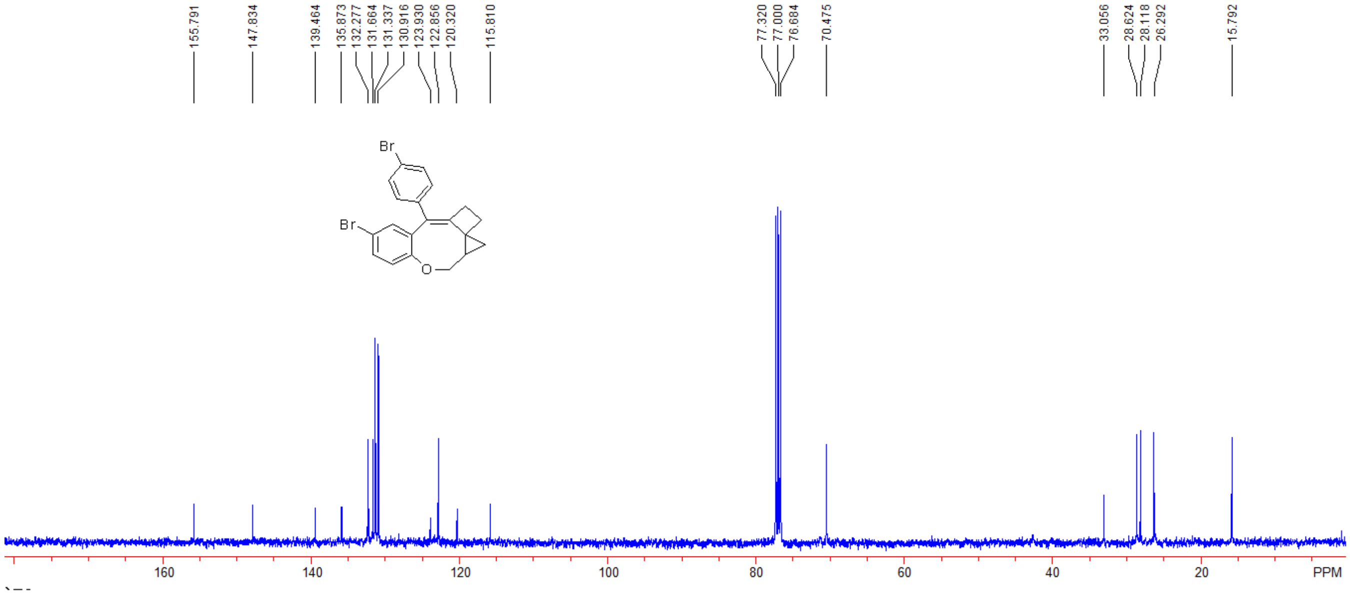

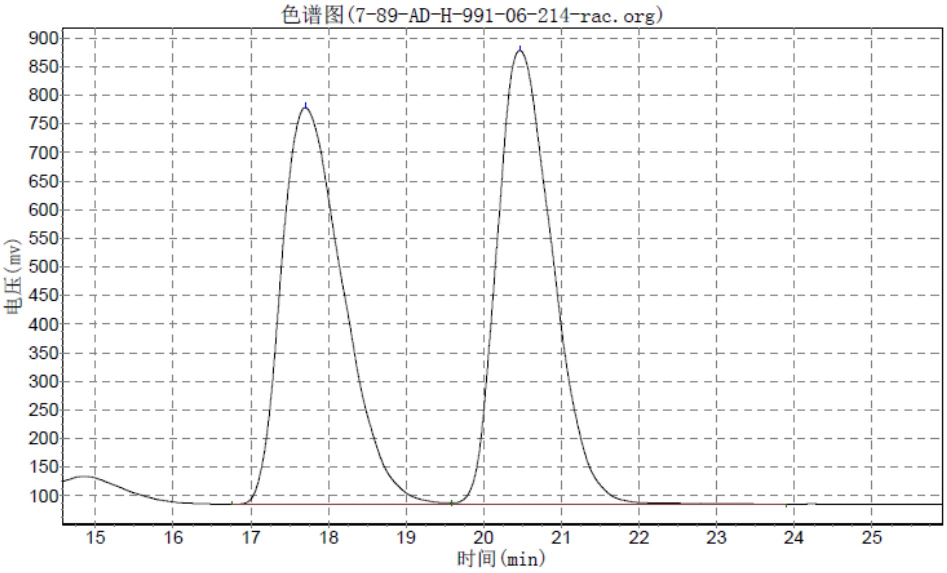

分析结果表

峰号	峰名	保留时间	峰高	峰面积	含量
1		17.702	693920.625	38412136.000	49.3143
2		20.463	793454.688	39480344.000	50.6857
总计			1487375.313	77892480.000	100.0000

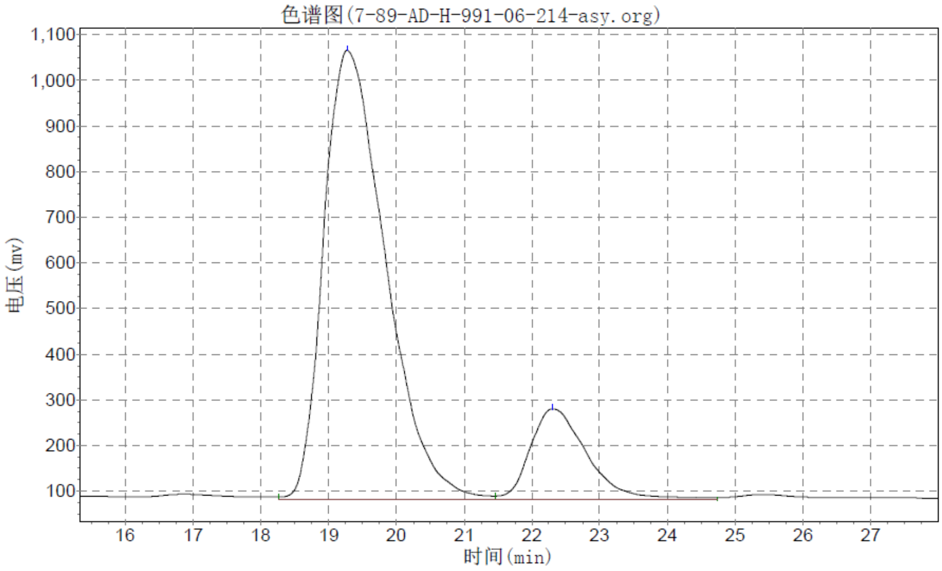

分析结果表

峰号	峰名	保留时间	峰高	峰面积	含量
1		19.272	982740.625	62227776.000	84.6045
2		22.308	197050.313	11323605.000	15.3955
总计			1179790.938	73551381.000	100.0000

Translation: a Chiralcel AD-H column [λ = 214 nm; eluent: Hexane/Isopropanol = 99/1; Flow rate: 0.60 mL/min; t_{minor} = 19.27 min, t_{major} = 22.31 min; ee% = 69%.]

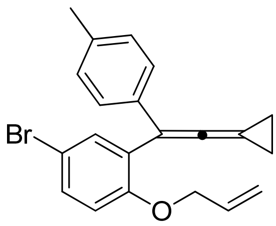

Compound **1o**. 426 mg, yield: 72%; white solid. MP: 109-110 °C ^{1}H NMR ($CDCl_3$, 400 MHz, TMS) δ 1.65-1.68 (m, 2H, CH_2), 1.71-1.72 (m, 2H, CH_2), 2.31 (s, 3H, CH_3), 4.40-4.41 (m, 2H, CH_2), 5.06-5.11 (m, 2H, =CH_2), 5.68-5.78 (m, 1H, =CH), 6.75-6.78 (m, 1H, Ar), 7.05-7.12 (m, 4H, Ar), 7.32-7.35 (m, 1H, Ar), 7.42-7.43 (m, 1H, Ar). ^{13}C NMR ($CDCl_3$, 100 MHz, TMS) δ 8.8, 21.1, 69.2, 78.8, 106.7, 113.0, 114.7, 116.8, 126.5, 128.8, 129.4, 130.0, 132.7, 134.0, 134.7, 135.9, 155.4, 189.9. IR (neat) ν 3019, 1958, 2919, 2003, 1510, 1488, 1462, 1410, 1233, 1129, 1043, 996, 927, 820, 804 cm^{-1}. MS (%) m/e 366 (M^+, 0.23), 325 (15.09), 247 (19.71), 246 (100.00), 245 (39.28), 231 (37.39), 203 (17.89), 202 (37.86), 129 (13.85). HRMS (EI) calcd. for $C_{21}H_{19}OBr$: 366.0619, Found: 366.0620.

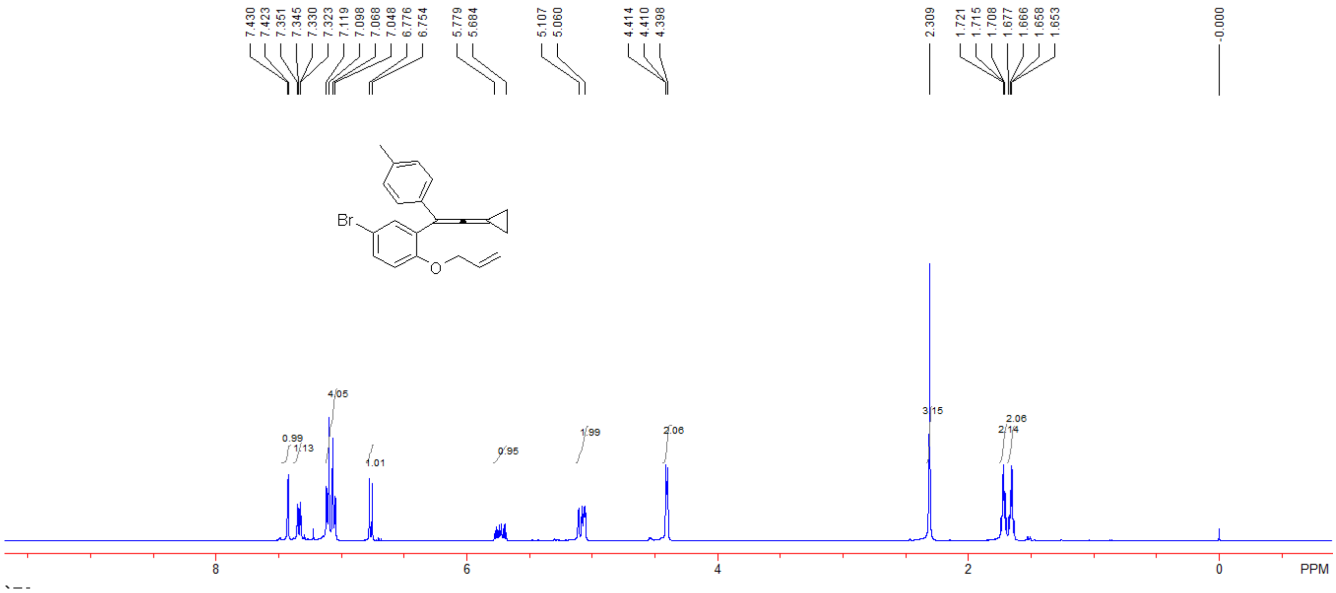

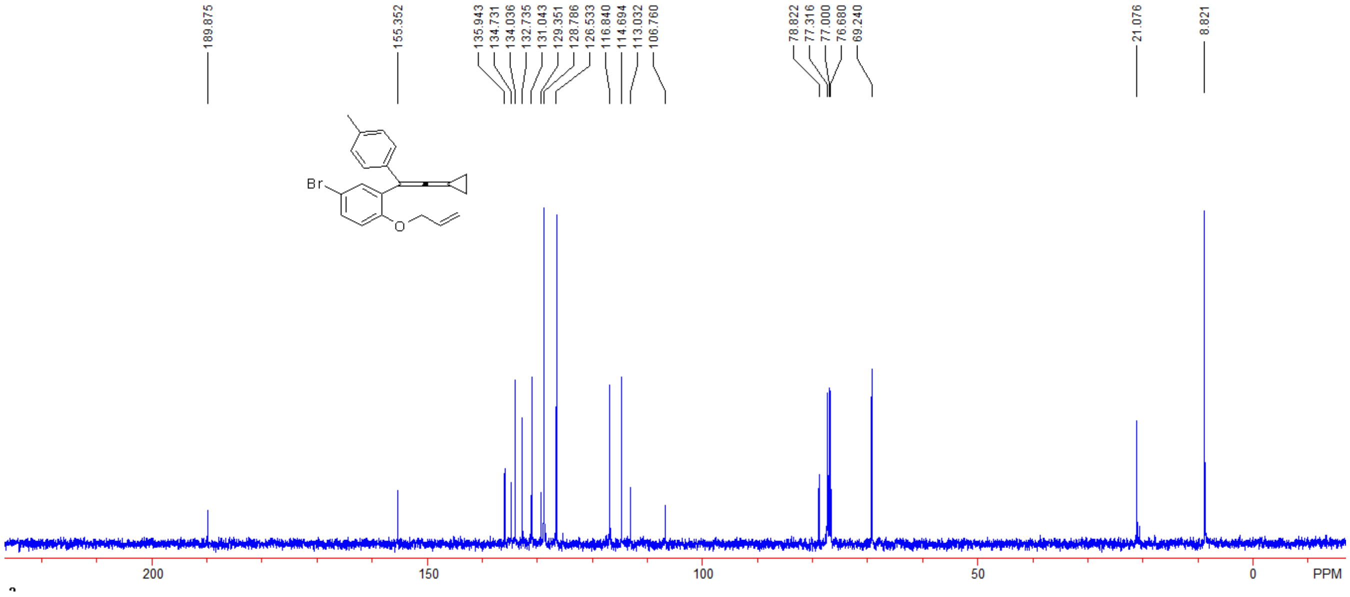

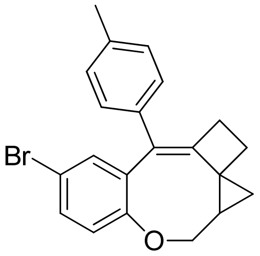

Compound **2o**. 124 mg, yield: 68%; colorless oil. ^{1}H NMR ($CDCl_3$, 400 MHz, TMS) δ 0.51 (dd, J_1 = 5.2 Hz, J_2 = 8.0 Hz, 1H, CH_2), 0.64 (dd, J_1 = 5.2 Hz, J_2 = 9.6 Hz, 1H, CH_2), 1.24-1.25 (m, 1H, CH), 2.16-2.31 (m, 2H, CH_2), 2.34 (s, 3H, CH_3), 2.89-2.97 (m, 1H, CH_2), 3.19-3.27 (m, 1H, CH_2), 4.36 (d, J = 12.4 Hz, 1H, CH_2), 4.86 (dd, J_1 = 6.0 Hz, J_2 = 12.4 Hz, 1H, CH_2), 6.89 (d, J = 8.4 Hz, 1H, Ar), 6.99 (d, J = 2.4 Hz, 1H, Ar), 7.11 (s, 4H, Ar), 7.27-7.30 (m, 1H, Ar). ^{13}C NMR ($CDCl_3$, 100 MHz, TMS) δ 15.6, 21.2, 26.1, 28.2, 28.6, 29.7, 33.0, 42.6, 70.5, 71.3, 115.7, 122.7, 124.9, 128.9, 129.1, 131.3, 132.4, 136.0, 136.6, 137.6, 146.3, 155.8. IR (neat) ν 2917, 2868, 2725, 1510, 1476, 1455, 1249, 1263, 1213, 1123, 1078, 983, 921, 819, 736 cm^{-1}. MS (%) m/e 366 (M^+, 7.43), 257 (68.65), 242 (71.53), 215 (63.82), 202 (100.00), 189 (75.77), 165 (83.62), 115 (60.35), 101 (56.61). HRMS (EI) calcd. for $C_{21}H_{19}OBr$: 366.0619, Found: 366.0615; Enantiomeric excess was determined by HPLC with a Chiralcel AD-H column [λ = 214 nm; eluent: Hexane/Isopropanol = 99/1; Flow rate: 0.60 mL/min; t_{minor} = 25.76 min, t_{major} = 22.72 min; ee% = 68%; $[\alpha]^{20}_D$ = -2.3695 (c 0.98, CH_2Cl_2)].

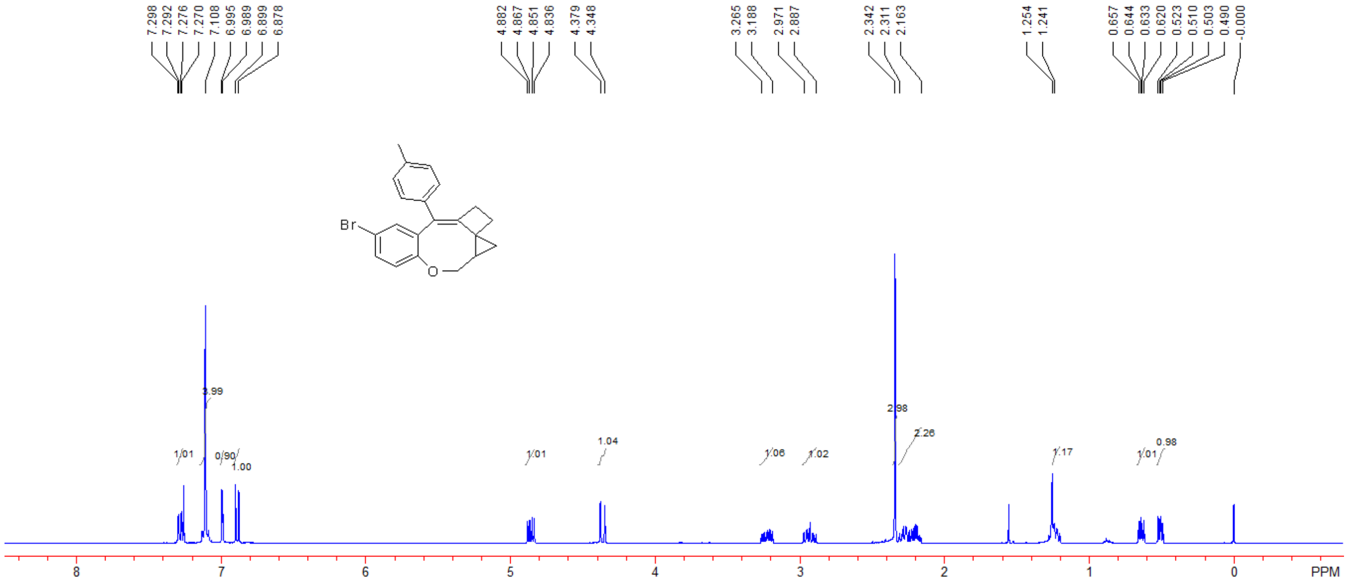
7.298
7.292
7.276
7.270
7.108
6.995
6.989
6.899
6.878
4.882
4.867
4.851
4.836
4.379
4.348
3.265
3.188
2.971
2.887
2.342
2.311
2.163
1.254
1.241
0.657
0.644
0.633
0.620
0.523
0.510
0.503
0.490
-0.000
Br
O
1.01
3.99
0.90
1.00
1.01
1.04
1.06
1.02
2.98
2.26
1.17
1.01
0.98
PPM

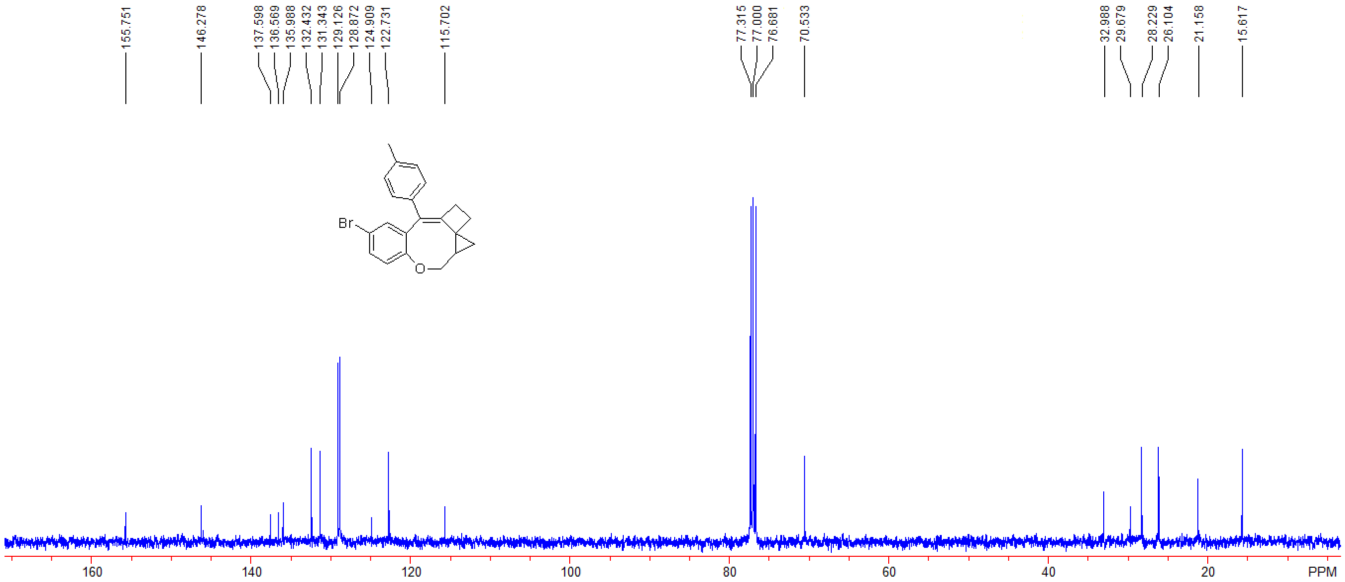
155.751
146.278
137.598
136.569
135.988
132.432
131.343
129.126
128.872
124.909
122.731
115.702
77.315
77.000
76.681
70.533
32.988
29.679
28.229
26.104
21.158
15.617
Br
O
PPM

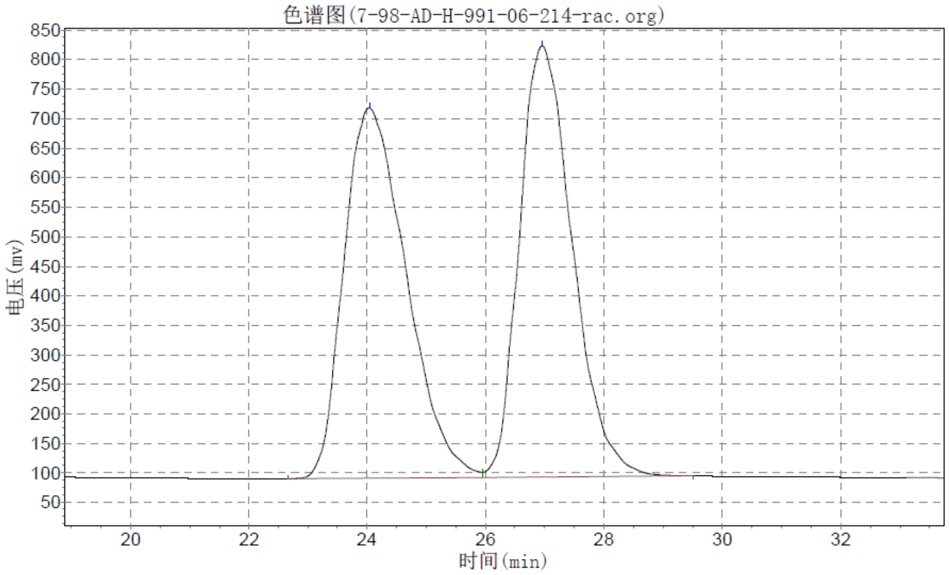

分析结果表

峰号	峰名	保留时间	峰高	峰面积	含量
1		24.032	627500.938	45840580.000	50.1875
2		26.957	730879.000	45498132.000	49.8125
总计			1358379.938	91338712.000	100.0000

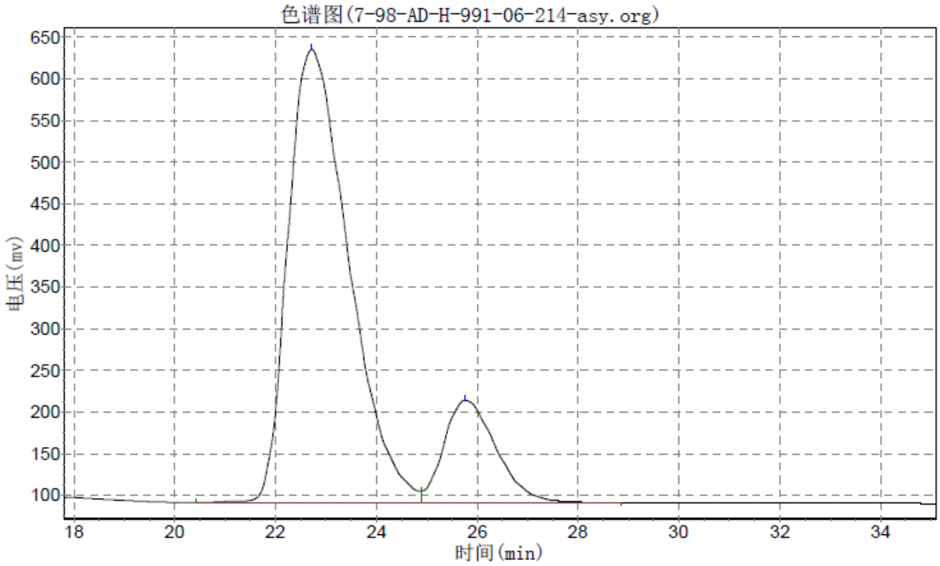

分析结果表

峰号	峰名	保留时间	峰高	峰面积	含量
1		22.717	544439.625	45482540.000	83.7667
2		25.757	122705.281	8814124.000	16.2333
总计			667144.906	54296664.000	100.0000

Translation: a Chiralcel AD-H column [λ = 214 nm; eluent: Hexane/Isopropanol = 99/1; Flow rate: 0.60 mL/min; t_{minor} = 25.76 min, t_{major} = 22.72 min; ee% = 68%.]

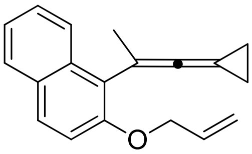

Compound **1p**. 365 mg, yield: 72%; colorless oil. ^{1}H NMR ($CDCl_3$, 400 MHz, TMS) δ 1.38-1.41 (m, 2H, CH_2), 1.51-1.55 (m, 2H, CH_2), 2.19 (s, 3H, CH_3), 4.70-4.71 (m, 2H, CH_2), 5.24-5.27 (m, 1H, =CH_2), 5.43-5.46 (m, 1H, =CH_2), 6.04-6.14 (m, 1H, =CH), 7.22-7.24 (m, 1H, Ar), 7.31-7.35 (m, 2H, Ar), 7.45-7.48 (m, 1H, Ar), 7.70-7.76 (m, 2H, Ar), 8.16-8.19 (m, 1H, Ar). ^{13}C NMR ($CDCl_3$, 100 MHz, TMS) δ 6.7, 20.7, 70.3, 76.1, 97.8, 115.5, 117.1, 123.6, 124.0, 125.1, 126.2, 127.9, 128.4, 129.5, 132.5, 133.8, 152.6, 189.3. IR (neat) ν 3057, 2980, 2907, 2017, 1590, 1507, 1429, 1327, 1264, 1245, 1220, 1054, 922, 803, 745 cm^{-1}. MS (%) m/e 262 (M^+, 9.74), 247 (15.70), 221 (32.88), 205 (20.08), 195 (16.77), 178 (16.48), 130 (18.68), 109 (100.00), 81 (15.54). HRMS (EI) calcd. for $C_{19}H_{18}O$: 262.1358, Found: 262.1355.

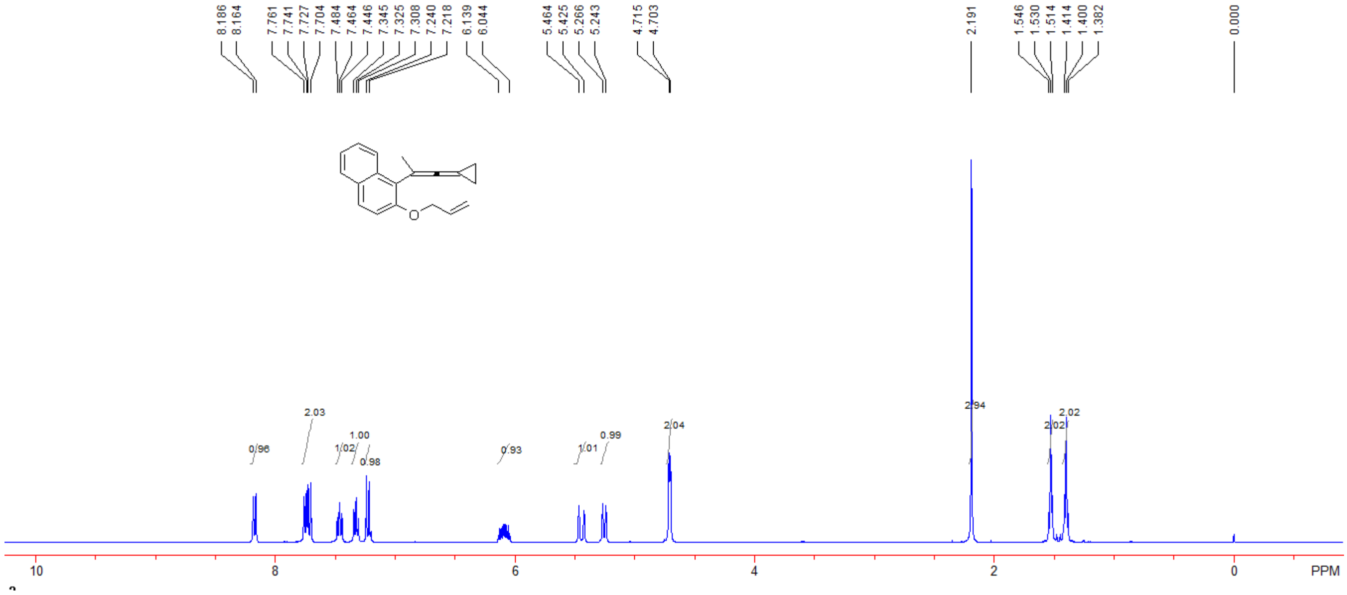

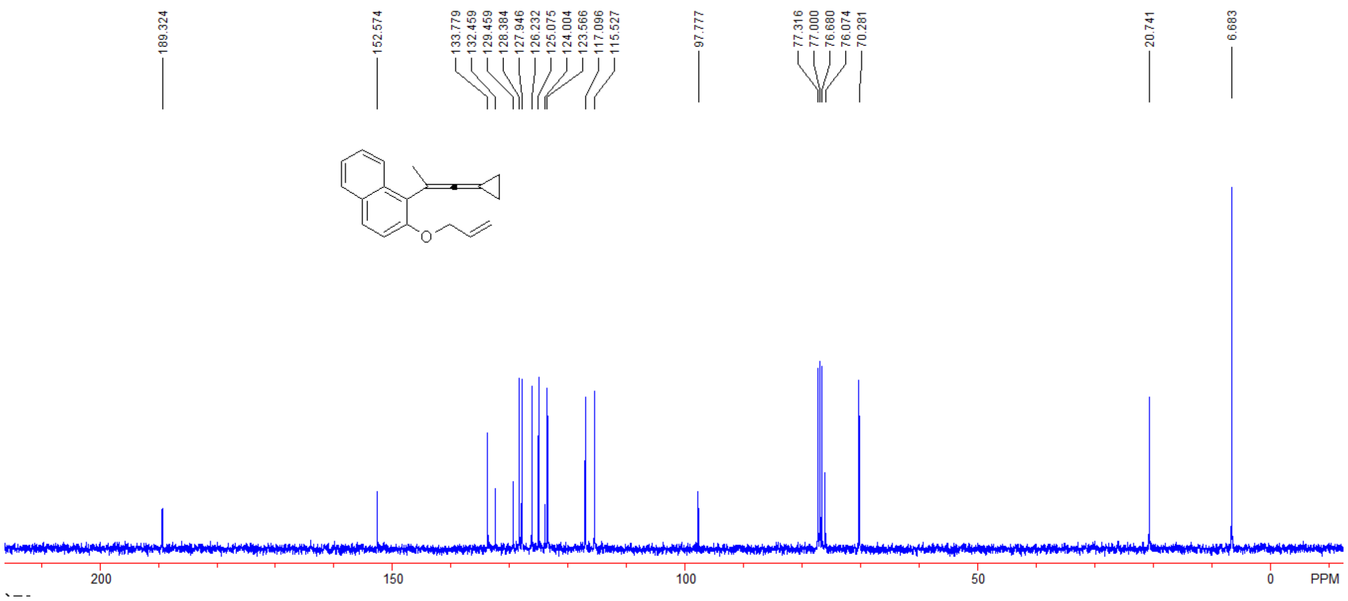

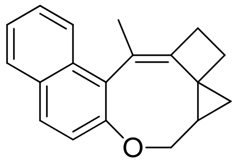

Compound **2p**. 120 mg, yield: 92%; yellow oil. ^{1}H NMR ($CDCl_3$, 400 MHz, TMS) δ 0.13 (dd, J_1 = 5.2 Hz, J_2 = 7.6 Hz, 1H, CH_2), 0.34 (dd, J_1 = 5.2 Hz, J_2 = 9.6 Hz, 1H, CH_2), 1.05-1.10 (m, 1H, CH), 2.08-2.16 (m, 4H, CH_3, CH_2), 2.26-2.33 (m, 1H, CH_2), 2.83-2.90 (m, 1H, CH_2), 3.09-3.17 (m, 1H, CH_2), 4.41 (d, J = 12.4 Hz, 1H, CH_2), 4.86 (dd, J_1 = 6.0 Hz, J_2 = 12.4 Hz, 1H, CH_2), 7.11-7.13 (m, 1H, Ar), 7.35-7.39 (m, 1H, Ar), 7.43-7.47 (m, 1H, Ar), 7.68-7.70 (m, 1H, Ar), 7.75-7.77 (m, 1H, Ar), 7.90-7.92 (m, 1H, Ar). ^{13}C NMR ($CDCl_3$, 100 MHz, TMS) δ 14.5, 18.8, 24.9, 27.6, 28.0, 32.3, 70.6, 117.5, 121.2, 124.3, 125.6, 126.4, 128.1, 129.0, 130.1, 130.5, 131.7, 144.1, 152.7. IR (neat) ν 3054, 2930, 2861, 1589, 1465, 1372, 1330, 1217, 1208, 1032, 1005, 908, 818, 729 cm^{-1}. MS (%) m/e 262 (M^+, 6.27), 247 (10.06), 221 (7.54), 195 (11.30), 109 (21.05), 95 (33.08), 93 (100.00), 65 (19.98), 63 (59.15). HRMS (EI) calcd. for $C_{19}H_{18}O$: 262.1358, Found: 262.1360.

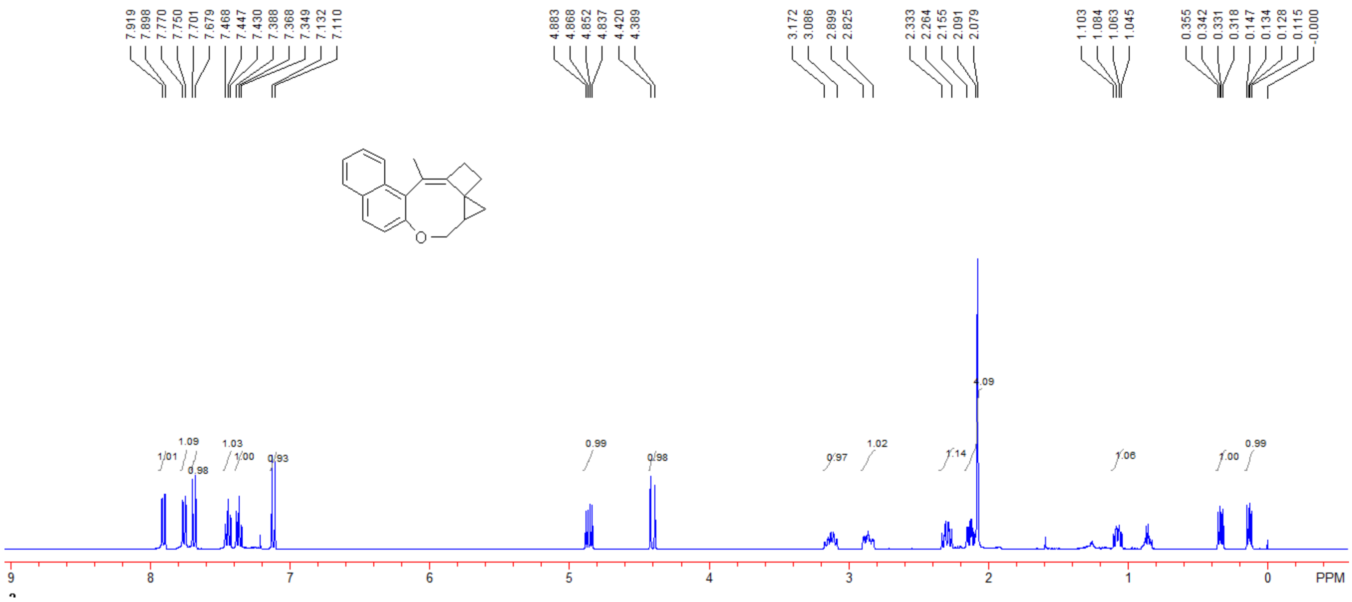

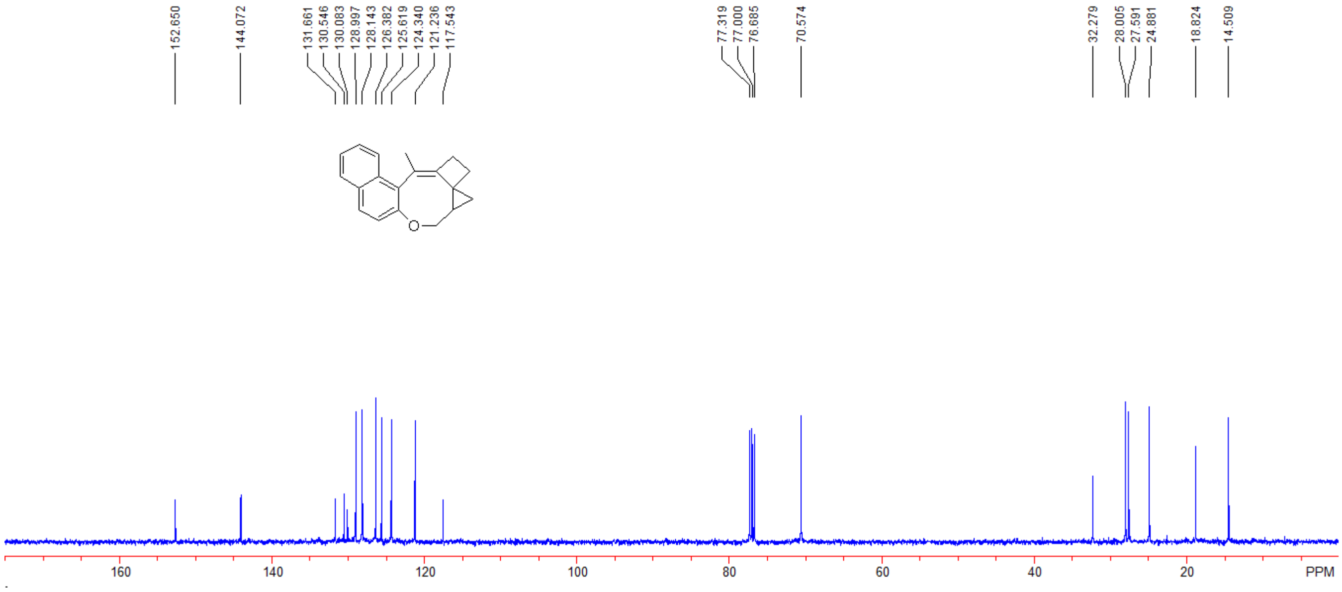

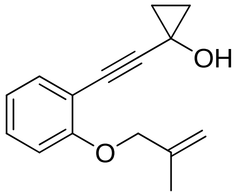

Compound **S3q**. 2.350 g, yield: 52%; yellow oil. ^{1}H NMR ($CDCl_3$, 400 MHz, TMS) δ 1.06-1.34 (m, 2H, CH_2), 1.15-1.19 (m, 2H, CH_2), 1.83 (s, 3H, CH_3), 3.22 (br, 1H, OH), 4.46 (s, 2H, CH_2), 4.98 (s, 1H, =CH_2), 5.15 (s, 1H, =CH_2), 6.81-6.88 (m, 2H, Ar), 7.20-7.24 (m, 1H, Ar), 7.35-7.37 (m, 1H, Ar). ^{13}C NMR ($CDCl_3$, 100 MHz, TMS) δ 17.5, 19.2, 46.0, 71.8, 78.3, 95.0, 112.16, 112.23, 112.5, 120.5, 129.3, 133.2, 140.4, 159.0. IR (neat) ν 3348, 2999, 2977, 2245, 1490, 1444, 1274, 1222,

1161, 1112, 1015, 970, 905, 748, 731 cm^{-1}. MS (%) m/e 228 (M^+, 4.06), 185 (51.58), 157 (51.92), 145 (75.01), 131 (100.00), 128 (62.29), 115 (80.57), 89 (50.17), 55 (98.19). HRMS (EI) calcd. for $C_{15}H_{15}O_2$[M-H]$^+$: 227.1072, Found: 227.1074.

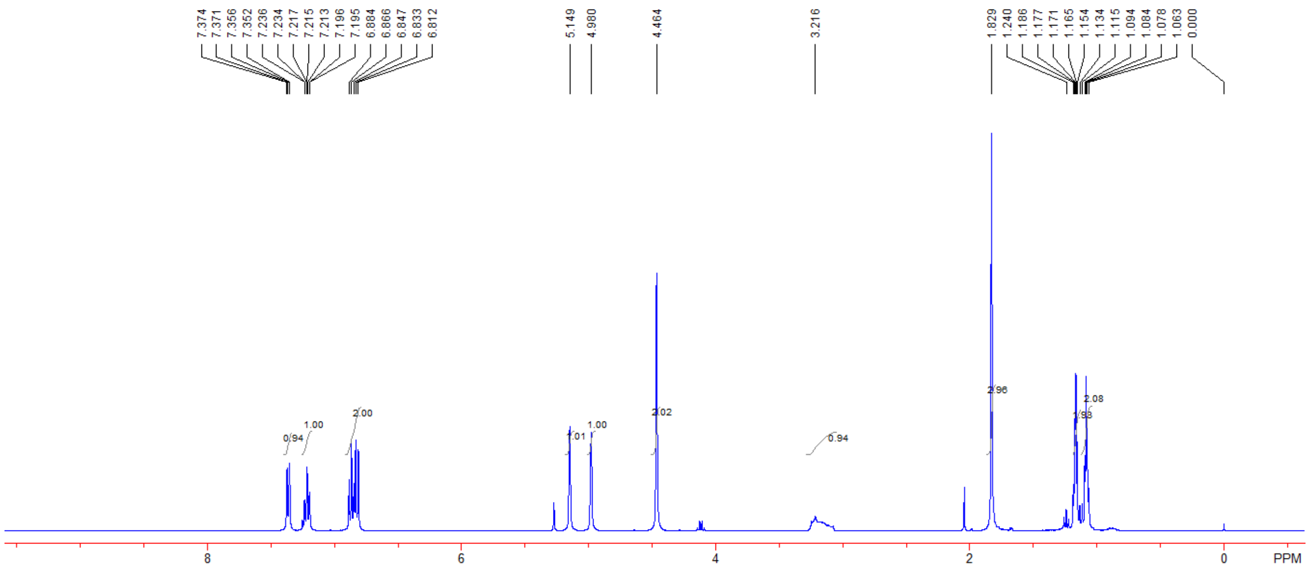

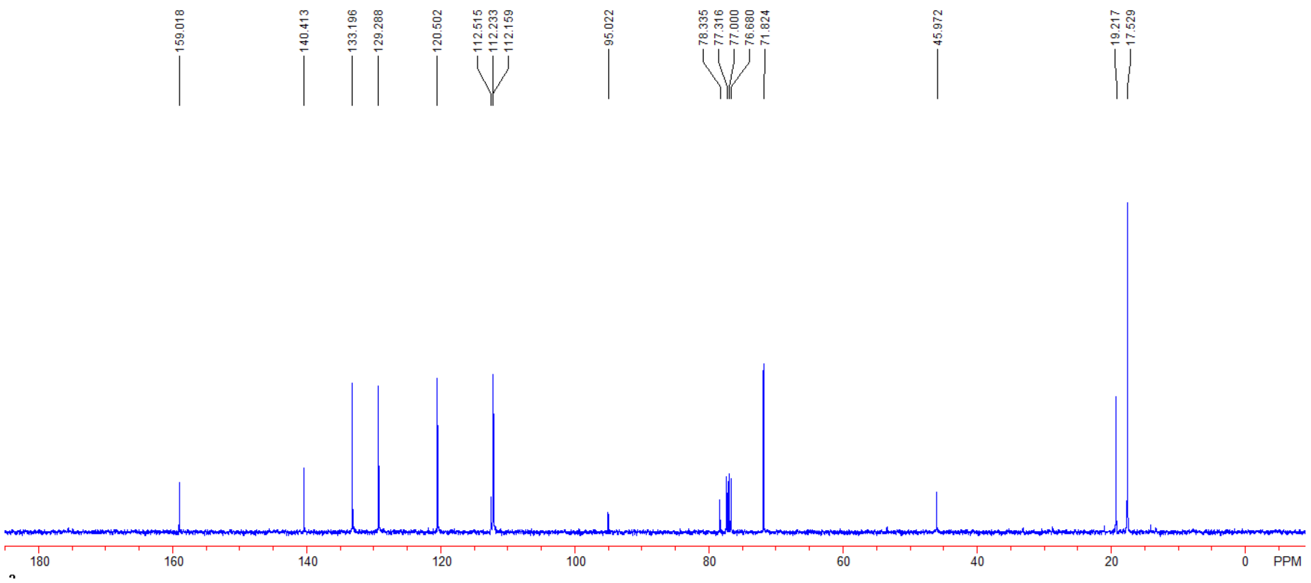

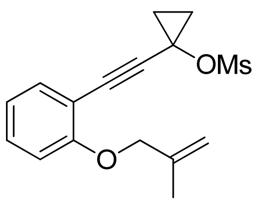

Compound **S4q**. 2.072 g, yield: 67%; yellow oil. ^{1}H NMR ($CDCl_3$, 400 MHz, TMS) δ 1.35-1.37 (m, 2H, CH_2), 1.62-1.64 (m, 2H, CH_2), 1.85 (s, 3H, CH_3), 3.29 (s, 3H, CH_3), 4.46 (s, 2H, CH_2), 5.01 (s, 1H, =CH_2), 5.12 (s, 1H, =CH_2), 6.84-6.90 (m, 2H, Ar), 7.26-7.30 (m, 1H, Ar), 7.35-7.37 (m, 1H, Ar). ^{13}C NMR ($CDCl_3$, 100 MHz, TMS) δ 16.8, 19.3, 39.7, 54.9, 71.8, 82.8, 89.4, 111.0, 111.9, 112.9,

120.5, 130.3, 133.1, 140.2, 159.4. IR (neat) ν 3021, 2937, 2857, 2256, 1596, 1360, 1245, 1158, 1113, 1007, 928, 904, 840, 729 cm^{-1}. MS (%) m/e 306 (M^+, 1.89), 185 (50.85), 145 (34.01), 131 (38.52), 128 (100.00), 116 (38.28), 115 (46.02), 88 (34.96), 55 (68.04). HRMS (EI) calcd. for $C_{16}H_{18}O_4S$: 306.0926, Found: 306.0930.

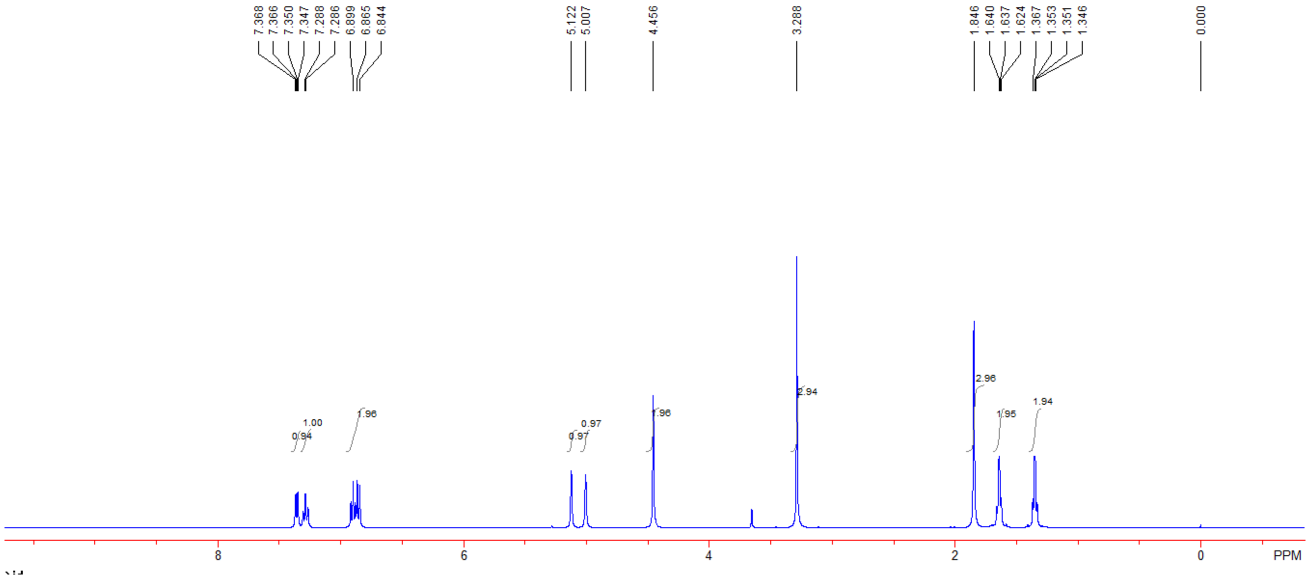

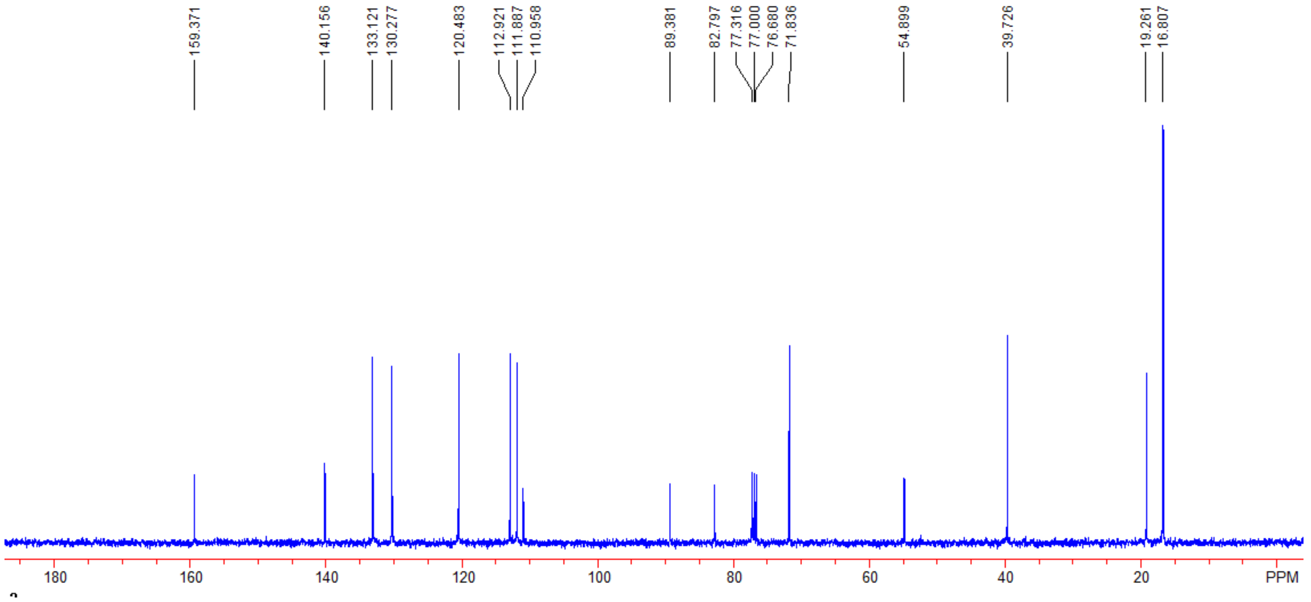

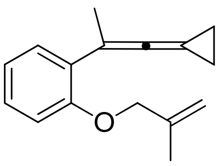

Compound **1q**. 450 mg, yield: 66%; colorless oil. ^{1}H NMR ($CDCl_3$, 400 MHz, TMS) δ 1.46-1.55 (m, 4H, CH_2), 1.82 (s, 3H, CH_3), 2.19 (s, 3H, CH_3), 4.42 (s, 2H, CH_2), 4.96 (s, 1H, =CH_2), 5.08 (s, 1H, =CH_2), 6.84 (d, *J* = 8.0 Hz, 1H, Ar), 6.91 (t, *J* = 7.2 Hz, 1H, Ar), 7.12-7.16 (m, 1H, Ar),

7.25-7.28 (m, 1H, Ar). ^{13}C NMR ($CDCl_3$, 100 MHz, TMS) δ 7.5, 19.5, 21.2, 53.4, 72.2, 75.4, 100.6, 112.4, 112.7, 120.6, 127.6, 128.6, 129.0, 141.1, 156.0, 190.4. IR (neat) ν 3054, 2981, 2913, 2010, 1594, 1488, 1443, 1367, 1246, 1214, 1121, 1012, 904, 729 cm^{-1}. MS (%) m/e 226 (M^+, 0.57), 171 (100.00), 153 (15.13), 152 (27.57), 141 (14.49), 128 (70.36), 127 (21.64), 115 (26.99), 43 (15.57). HRMS (EI) calcd. for $C_{16}H_{18}O$: 226.1358, Found: 226.1359.

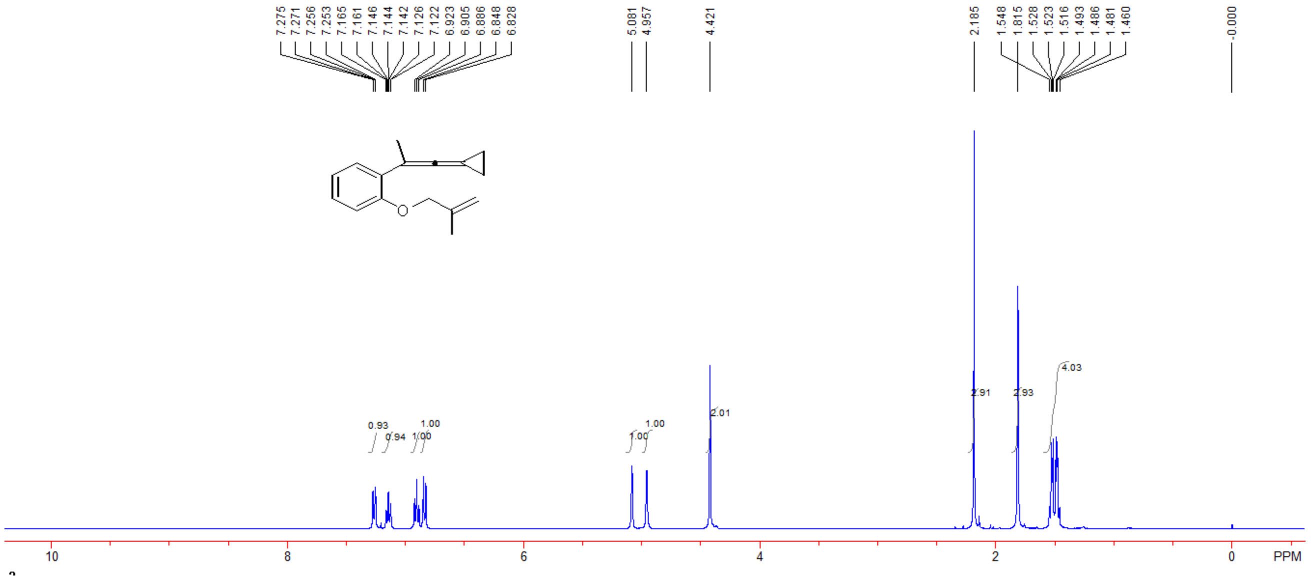

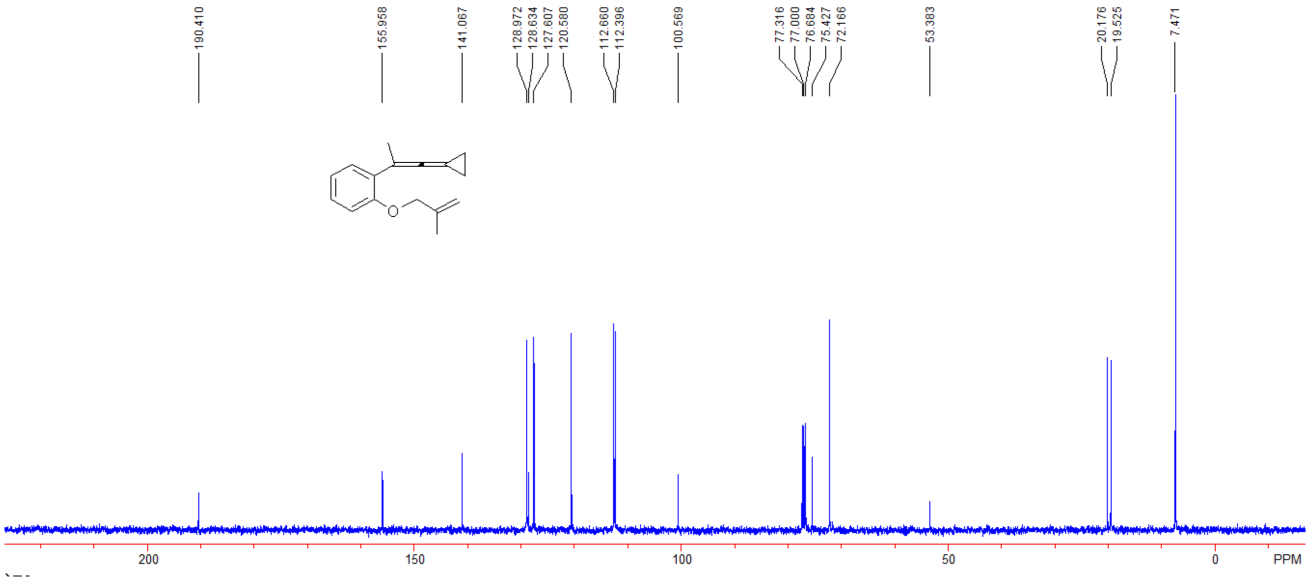

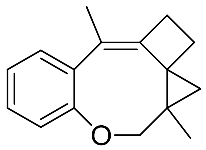

Compound **2q**. 102 mg, yield: 90%; colorless oil. ^{1}H NMR ($CDCl_3$, 400 MHz, TMS) δ 0.07-0.09 (m, 1H, CH_2), 0.57-0.58 (m, 1H, CH_2), 0.98 (s, 3H, CH_3), 1.91-1.98 (m, 4H, CH_3, CH), 2.27-2.29

(m, 1H, CH_2), 2.75-2.82 (m, 1H, CH_2), 2.90-2.99 (m, 1H, CH_2), 4.41-4.42 (m, 2H, CH_2), 6.95-7.03 (m, 2H, Ar), 7.15-7.25 (m, 2H, Ar). ^{13}C NMR ($CDCl_3$, 100 MHz, TMS) δ 17.4, 20.5, 20.9, 23.3, 25.3, 27.2, 37.2, 75.2, 119.0, 120.7, 123.0, 126.6, 127.9, 134.9, 142.6, 155.2. IR (neat) ν 3056, 2931, 2857, 1486, 1438, 1376, 1238, 1204, 1132, 1035, 956, 828, 761, 748 cm^{-1}. MS (%) m/e 226 (M^+, 18.61), 211 (100.00), 197 (46.67), 196 (67.86), 181 (49.63), 171 (94.6), 145 (47.58), 128 (44.64), 115 (48.73). HRMS (EI) calcd. for $C_{16}H_{18}O$: 226.1358, Found: 226.1360.

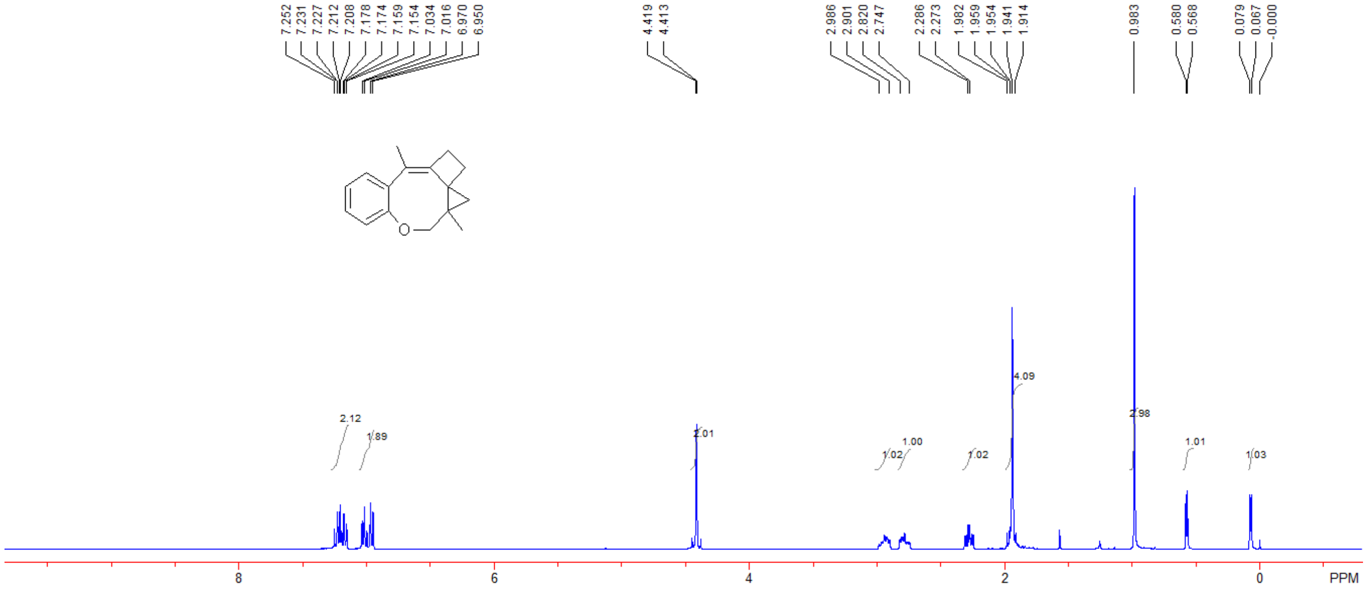

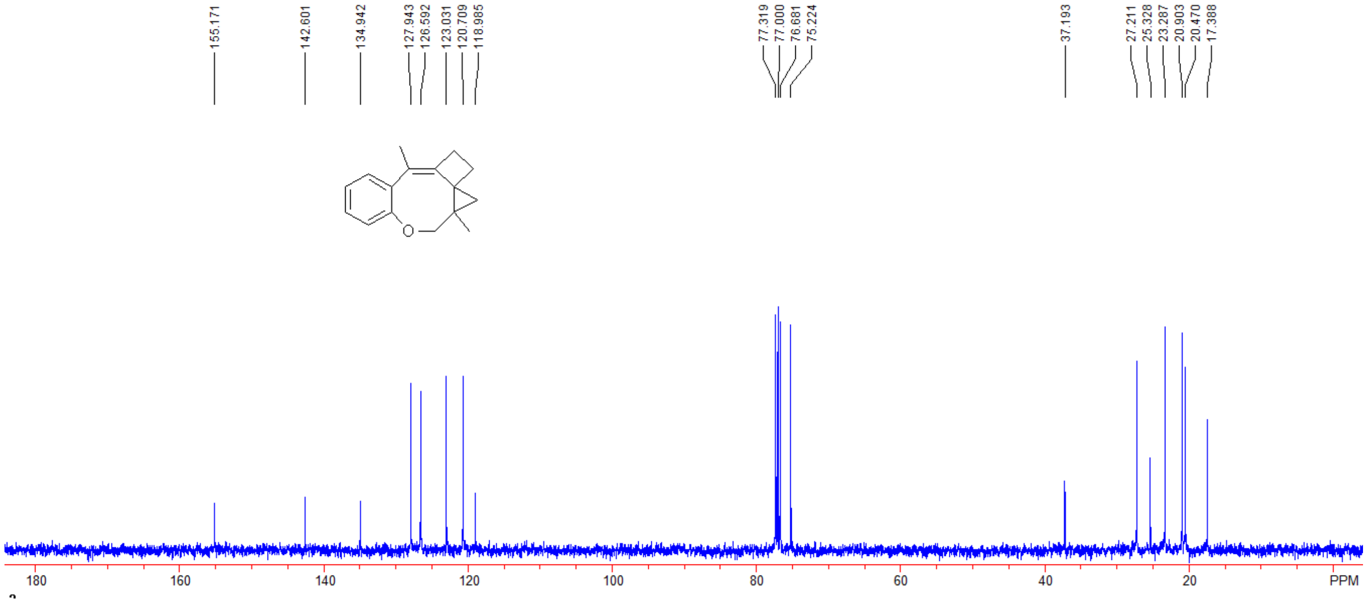

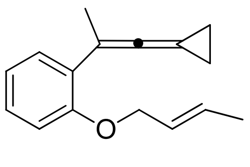

Compound **1r**. 500 mg, yield: 73%; colorless oil. ^{1}H NMR ($CDCl_3$, 400 MHz, TMS) δ 1.48-1.50 (m, 2H, CH_2), 1.52-1.54 (m, 2H, CH_2), 1.74 (d, J = 6.4 Hz, 3H, CH_3), 2.18 (s, 3H, CH_3), 4.42-4.43 (m, 2H, CH_2), 5.63-5.70 (s, 1H, =CH), 5.76-5.85 (s, 1H, =CH), 6.83 (d, J = 8.0 Hz, 1H, Ar), 6.90 (t, J = 7.2 Hz, 1H, Ar), 7.12-7.16 (m, 1H, Ar), 7.22-7.26 (m, 1H, Ar). ^{13}C NMR ($CDCl_3$, 100 MHz, TMS) δ 7.5, 17.8, 20.0, 69.0, 75.2, 100.3, 112.4, 120.5, 126.4, 127.5, 128.3, 128.6, 129.8, 156.0, 190.7. IR (neat) ν 3052, 3022, 2980, 2914, 2855, 2008, 1593, 1577, 1489, 1445, 1375, 1247, 1224, 1073, 1003, 964, 745 cm^{-1}. MS (%) m/e 226 (M^+, 1.03), 171 (100.00), 128 (57.57), 152 (22.02), 115 (20.14), 127 (17.73), 153 (14.98), 172 (13.76), 55 (13.23). HRMS (EI) calcd. for $C_{16}H_{18}O$: 226.1358, Found: 226.1353.

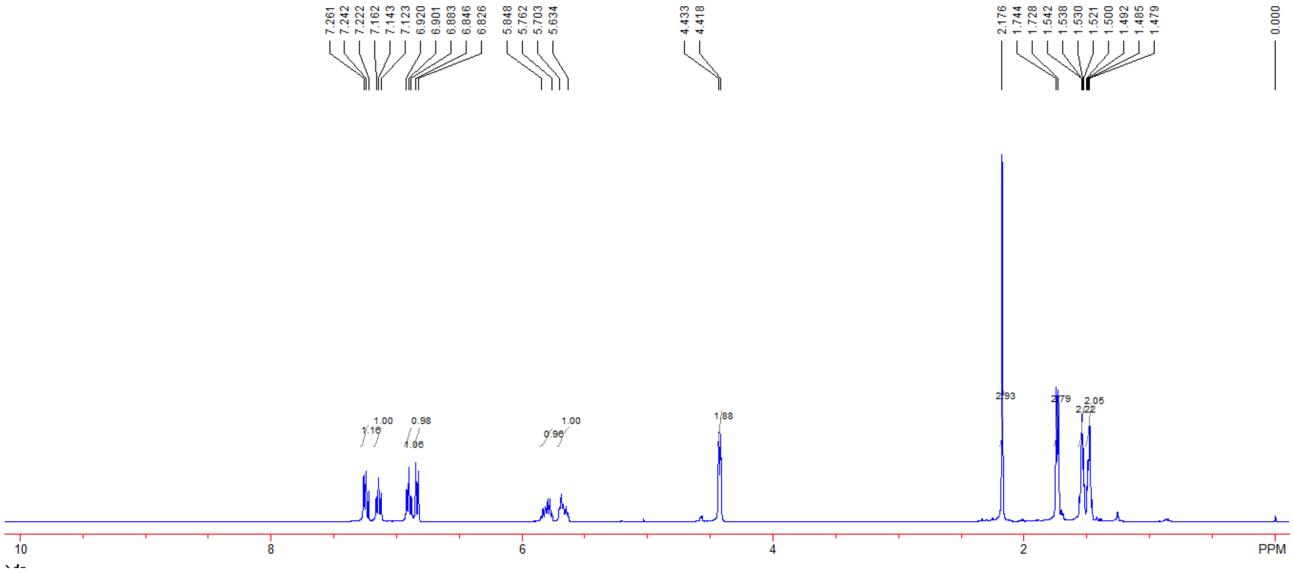

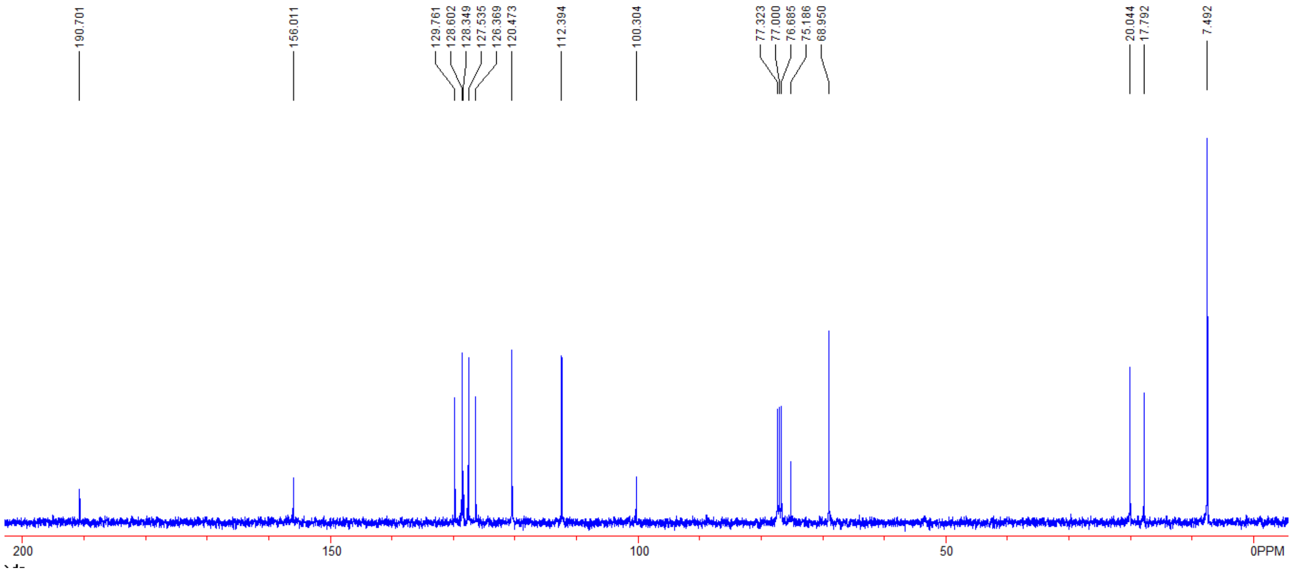

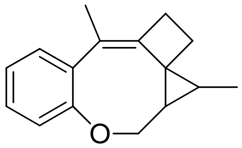

Compound **2r**. 193 mg, yield: 82%; colorless oil. ^{1}H NMR ($CDCl_3$, 400 MHz, TMS) δ 0.54-0.58 (m, 1H, CH_2), 0.69-0.72 (m, 1H, CH_2), 0.79 (d, J = 5.6 Hz, 3H, CH_3), 1.92 (s, 3H, CH_3), 1.95-2.01 (m, 1H, CH_2), 2.17-2.23 (m, 1H, CH_2), 2.72-2.80 (m, 1H, CH_2), 2.14-2.23 (m, 1H, CH_2), 4.36-4.39 (m, 1H, CH_2), 4.80-4.84 (m, 1H, CH_2), 6.94-6.96 (m, 1H, Ar), 6.99-7.03 (m, 1H, Ar), 7.16-7.20 (m, 1H, Ar), 7.21-7.24 (m, 2H, Ar). ^{13}C NMR ($CDCl_3$, 100 MHz, TMS) δ 15.0, 17.5, 18.6, 22.6, 27.3, 31.9, 37.8, 70.6, 118.6, 121.3, 122.9, 126.7, 128.0, 135.2, 142.9, 155.6. IR (neat) ν 3064, 3016, 2926, 2862, 1569, 1486, 1440, 1373, 1282, 1209, 1074, 1030, 971, 773, 749 cm^{-1}. HRMS (EI) calcd. for $C_{16}H_{18}O$: 226.1358, Found: 226.1355.

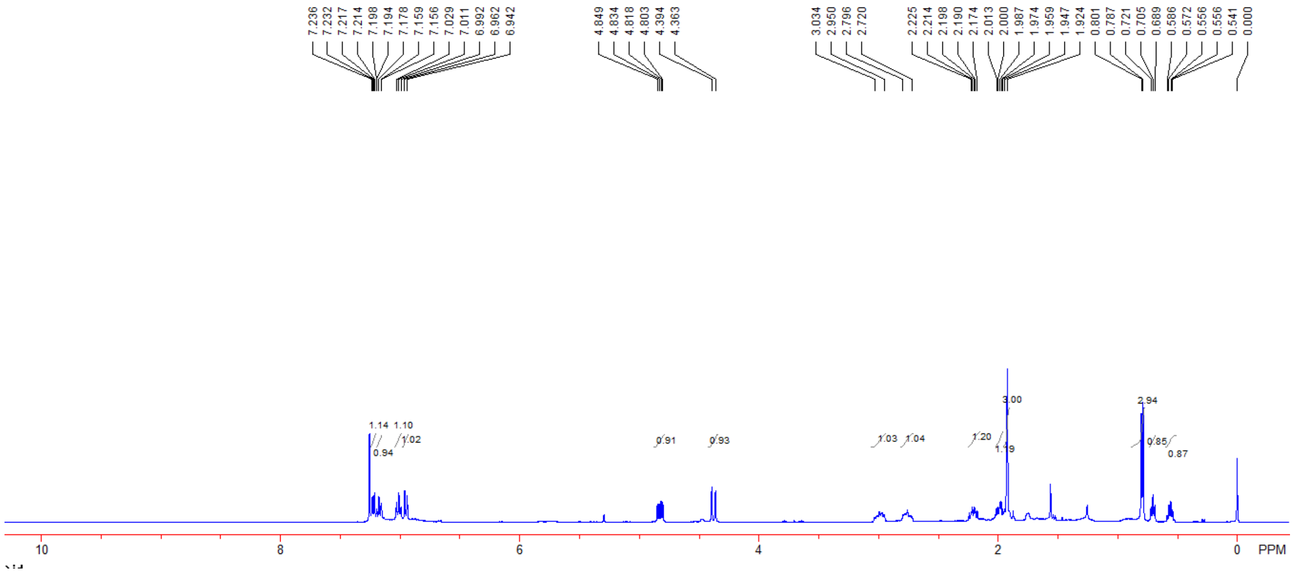

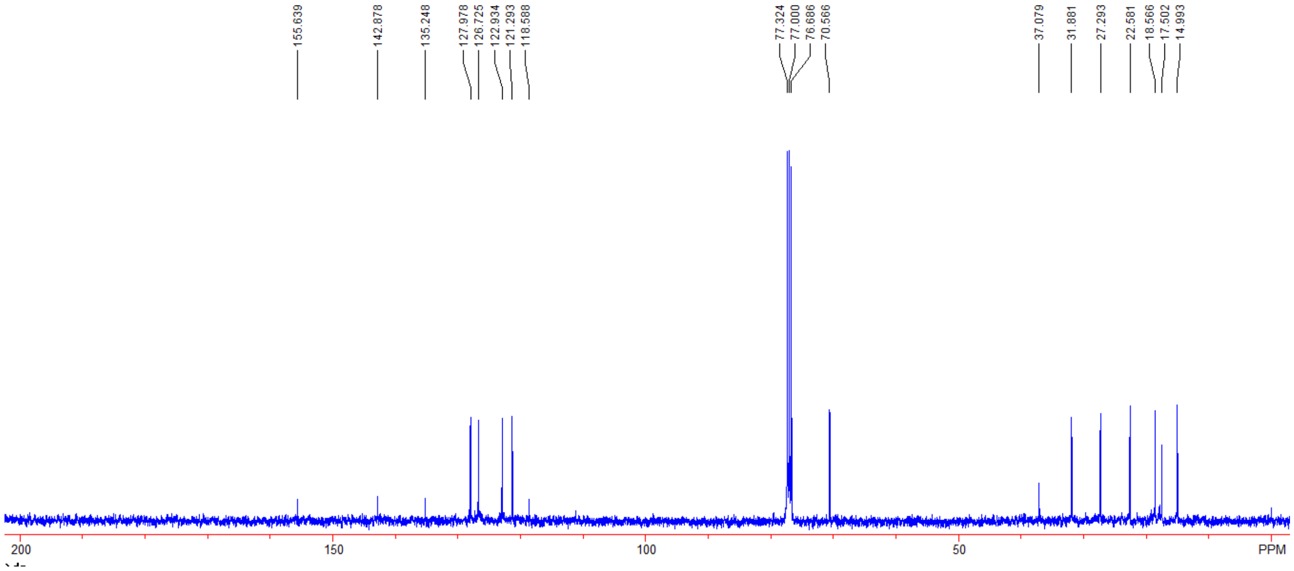

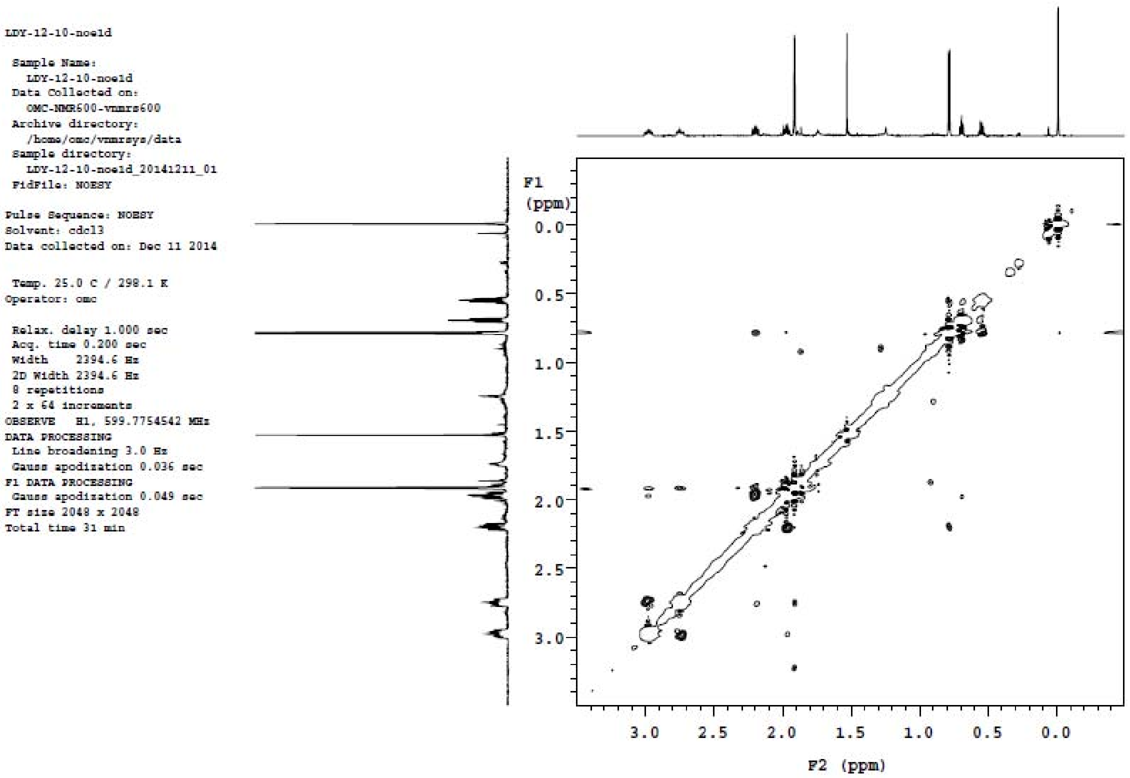
LDY-12-10-noe1d
Sample Name:
LDY-12-10-noe1d
Data Collected on:
OMC-NMR600-vnmrs600
Archive directory:
/home/omc/vnmrsys/data
Sample directory:
LDY-12-10-noe1d_20141211_01
FidFile: NOESY
Pulse Sequence: NOESY
Solvent: cdcl3
Data collected on: Dec 11 2014
Temp. 25.0 C / 298.1 K
Operator: omc
Relax. delay 1.000 sec
Acq. time 0.200 sec
Width 2394.6 Hz
2D Width 2394.6 Hz
8 repetitions
2 x 64 increments
OBSERVE H1, 599.7754542 MHz
DATA PROCESSING
Line broadening 3.0 Hz
Gauss apodization 0.036 sec
F1 DATA PROCESSING
Gauss apodization 0.049 sec
FT size 2048 x 2048
Total time 31 min
F1 (ppm)
F2 (ppm)

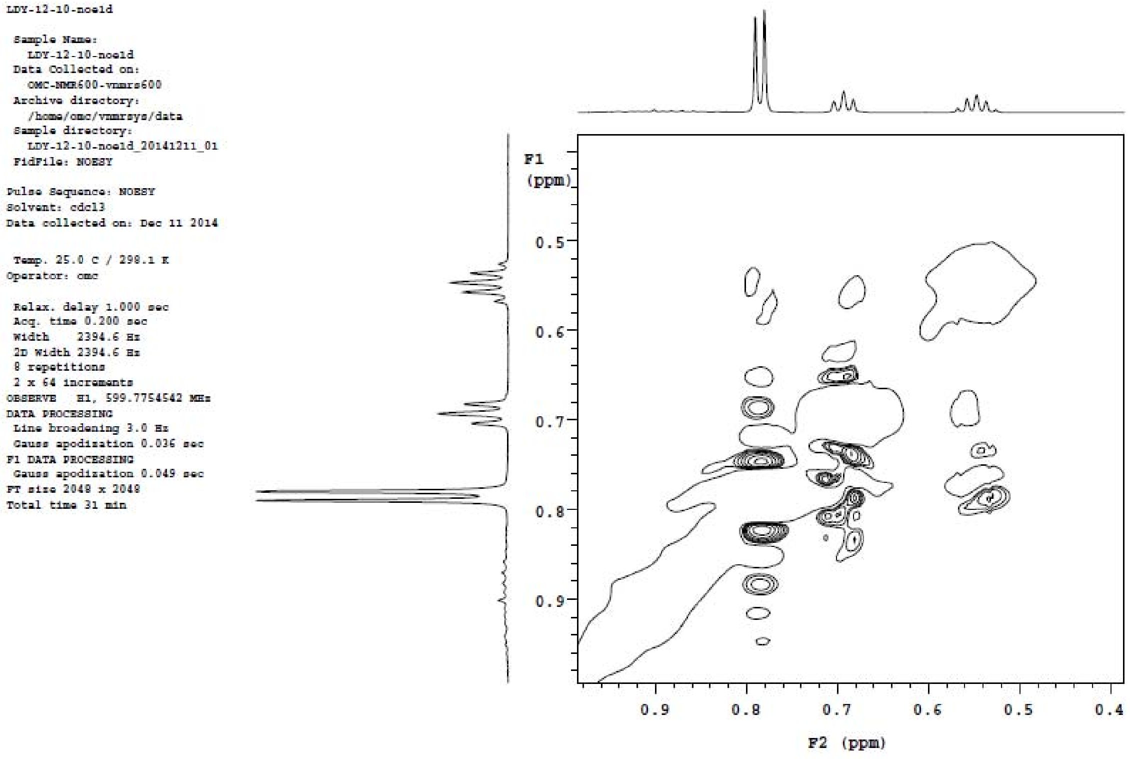
LDY-12-10-noe1d
Sample Name:
LDY-12-10-noe1d
Data Collected on:
OMC-NMR600-vnmrs600
Archive directory:
/home/omc/vnmrsys/data
Sample directory:
LDY-12-10-noe1d_20141211_01
FidFile: NOESY
Pulse Sequence: NOESY
Solvent: cdcl3
Data collected on: Dec 11 2014
Temp. 25.0 C / 298.1 K
Operator: omc
Relax. delay 1.000 sec
Acq. time 0.200 sec
Width 2394.6 Hz
2D Width 2394.6 Hz
8 repetitions
2 x 64 increments
OBSERVE H1, 599.7754542 MHz
DATA PROCESSING
Line broadening 3.0 Hz
Gauss apodization 0.036 sec
F1 DATA PROCESSING
Gauss apodization 0.049 sec
FT size 2048 x 2048
Total time 31 min
F1 (ppm)
F2 (ppm)

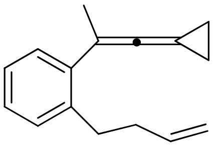

Compound **1s**. 525 mg, yield: 83%; colorless oil. ^{1}H NMR ($CDCl_3$, 400 MHz, TMS) δ 1.43-1.53 (m, 4H, $2CH_2$), 2.13 (s, 3H, CH_3), 2.29-2.34 (m, 2H, CH_2), 2.82-2.86 (m, 2H, CH_2), 4.96-5.06 (m, 2H, $=CH_2$), 5.85-5.92 (m, 1H, =CH), 7.16-7.21 (m, 3H, Ar), 7.28-7.30 (m, 1H, Ar). ^{13}C NMR ($CDCl_3$, 100 MHz, TMS) δ 7.0, 21.7, 32.9, 35.2, 76.4, 101.4, 114.4, 125.9, 126.7, 127.8, 129.8, 138.67, 138.71, 139.5, 188.7. IR (neat) ν 3059, 2979, 2910, 2857, 2016, 1639, 1483, 1437, 1412, 1367, 1073, 1040, 994, 906, 756 cm^{-1}. HRMS (EI) calcd. for $C_{16}H_{18}$: 210.1409, Found: 210.1406.

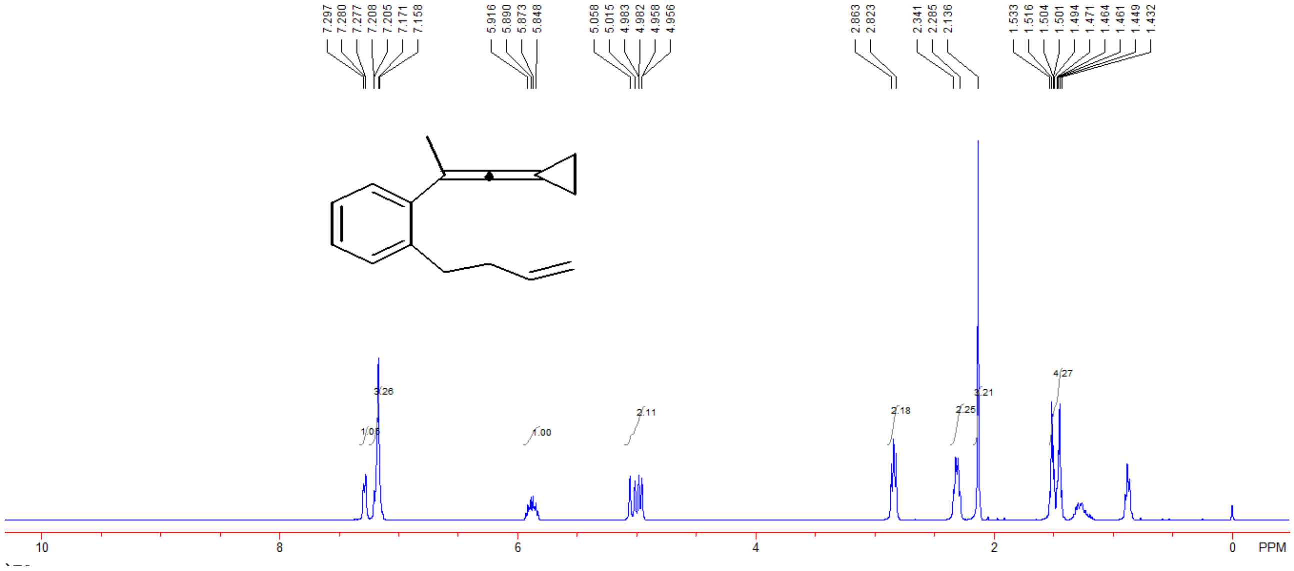

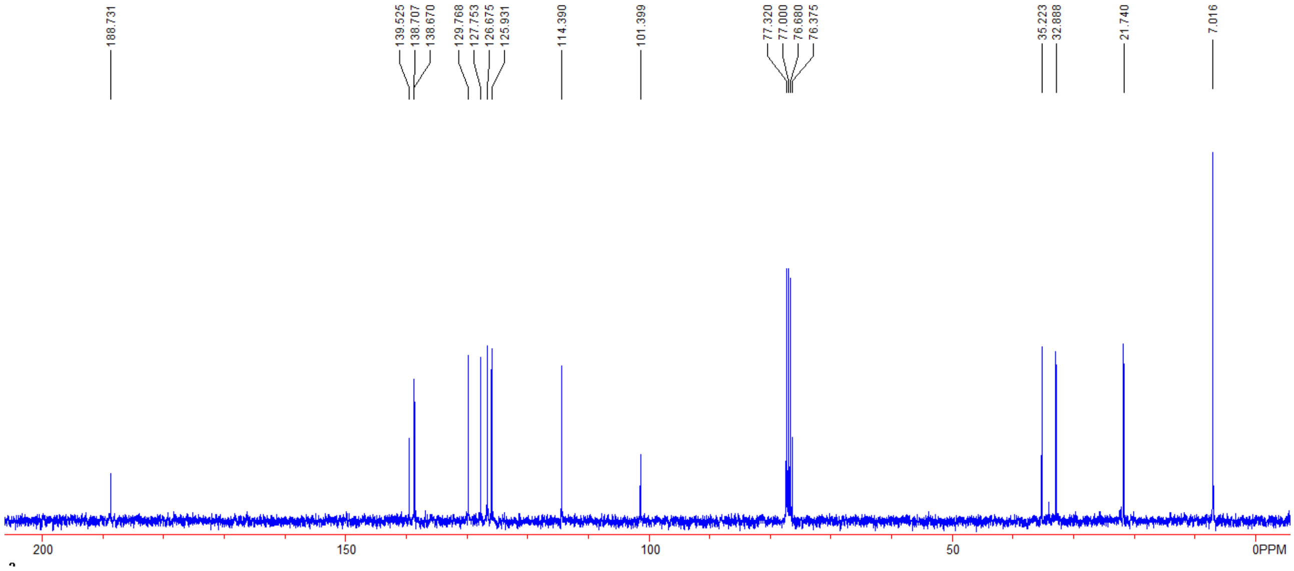

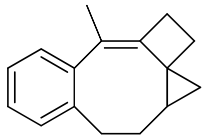

Compound **2s**. 98 mg, yield: 93%; colorless oil. ^{1}H NMR ($CDCl_3$, 400 MHz, TMS) δ -0.14-(-0.12) (dd, J_1 = 9.2 Hz, J_2 = 7.6 Hz, 1H, CH_2), 0.20-0.24 (dd, J_1 = 9.2 Hz, J_2 = 7.6 Hz, 1H, CH_2), 1.02-1.08 (dd, J_1 = 16.8 Hz, J_2 = 7.6 Hz, 1H, CH), 1.92 (s, 3H, CH_3), 1.97-2.07 (m, 2H, CH_2), 2.10-2.17 (m, 1H, CH_2), 2.43-2.49 (m, 1H, CH_2), 2.54-2.59 (m, 1H, CH_2), 2.72-2.87 (m, 1H, CH_2), 2.90-2.98 (m, 1H, CH_2), 7.09-7.14 (m, 3H, Ar), 7.22-7.24 (m, 1H, Ar). ^{13}C NMR ($CDCl_3$, 100 MHz, TMS) δ 15.3, 18.4, 24.0, 27.2, 27.6, 28.4, 29.9, 32.8, 122.2, 124.9, 126.2, 126.5, 129.0, 139.5, 140.7, 142.4. IR (neat) ν 3058, 2914, 2849, 1487, 1443, 1372, 1279, 1242, 1178, 1088, 1044, 1024, 2012, 915, 841, 750, 711 cm^{-1}. HRMS (EI) calcd. for $C_{16}H_{18}$: 210.1409, Found: 210.1404.

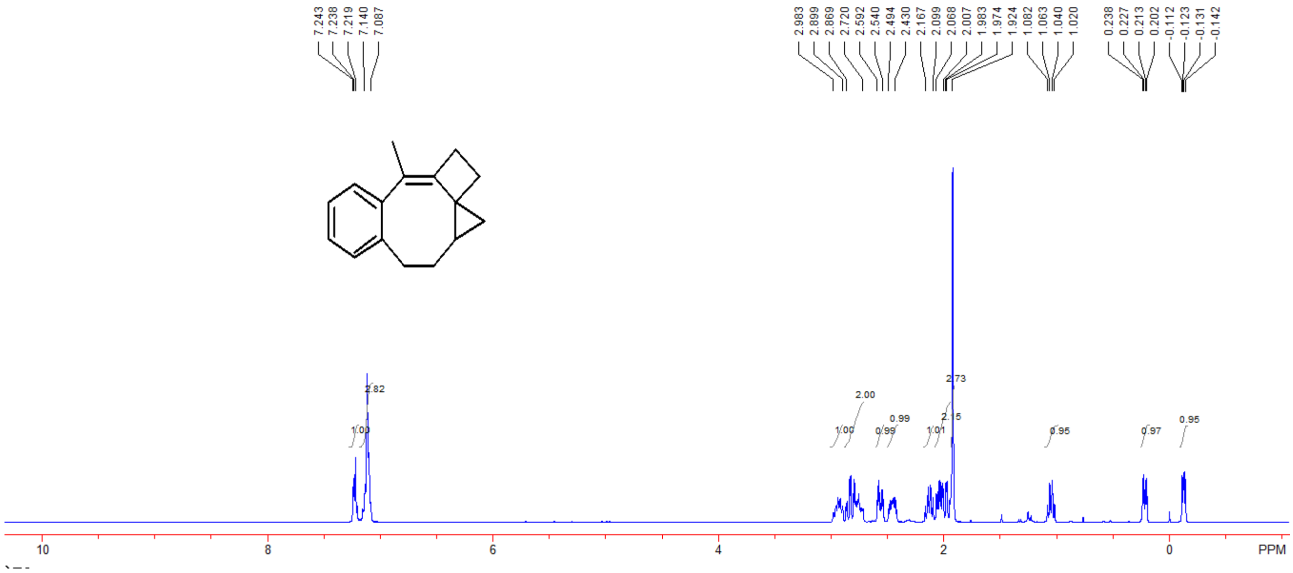

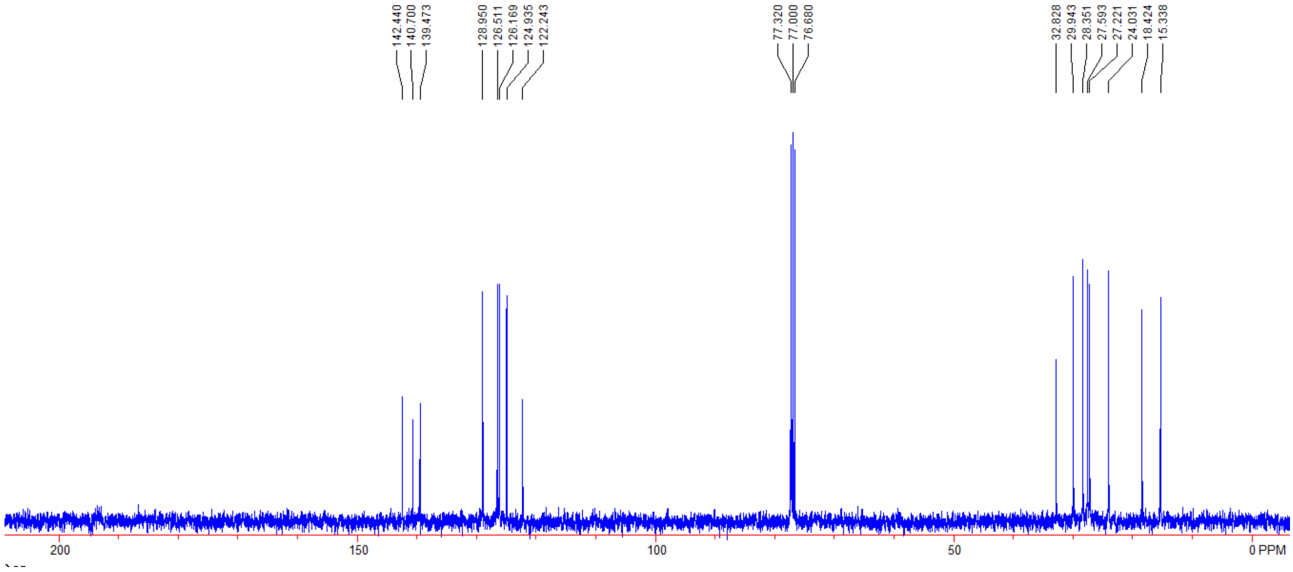

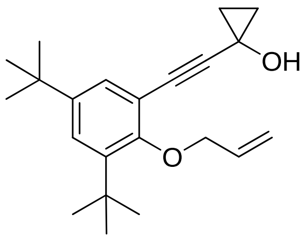

Compound **S3r**. 3.586 g, yield: 55%; yellow oil. ^{1}H NMR ($CDCl_3$, 400 MHz, TMS) δ 1.17-1.11 (m, 2H, CH_2), 1.14-1.18 (m, 2H, CH_2), 1.28 (s, 9H, ^{t}Bu), 1.38 (s, 9H, ^{t}Bu), 2.72 (br, 1H, OH), 4.72 (d, J = 4.8 Hz, 2H, CH_2), 5.27 (dd, J_1 = 1.2 Hz, J_2 = 10.4 Hz, 1H, =CH_2), 5.48 (dd, J_1 = 1.2 Hz, J_2 = 17.2 Hz, 1H, =CH_2), 6.10-6.18 (m, 1H, =CH), 7.26-7.30 (m, 2H, Ar). ^{13}C NMR ($CDCl_3$, 100 MHz, TMS) δ 17.5, 30.6, 31.3, 34.4, 35.2, 46.2, 73.3, 80.3, 94.9, 116.1, 116.5, 124.8, 128.7, 134.3, 141.9, 145.3, 156.9. IR (neat) ν 3254, 2959, 2906, 2869, 2226, 1437, 1387, 1241, 1228, 1202, 1120, 981, 969, 910, 881, 743 cm^{-1}. MS (%) m/e 326 (M$^+$, 3.31), 311 (13.05), 269 (14.41), 255 (16.51), 213 (10.93), 128 (11.92), 57 (100.00), 55 (18.49), 41 (29.32). HRMS (EI) calcd. for $C_{22}H_{30}O_2$: 326.2246, Found: 326.2249.

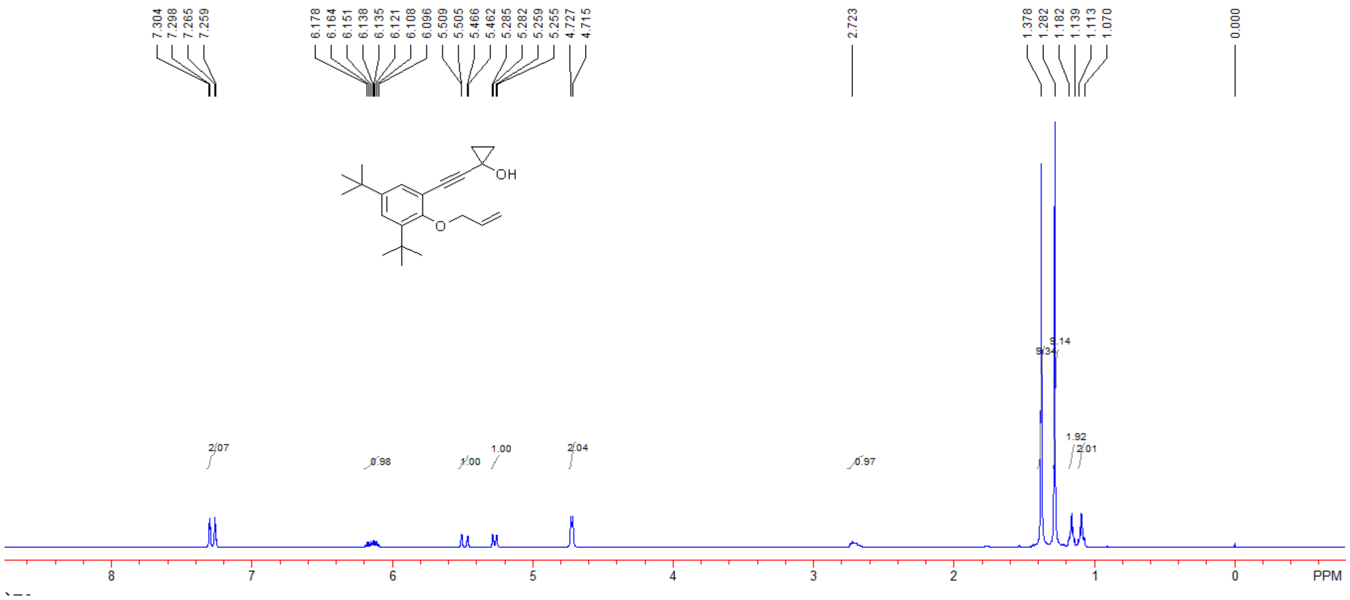

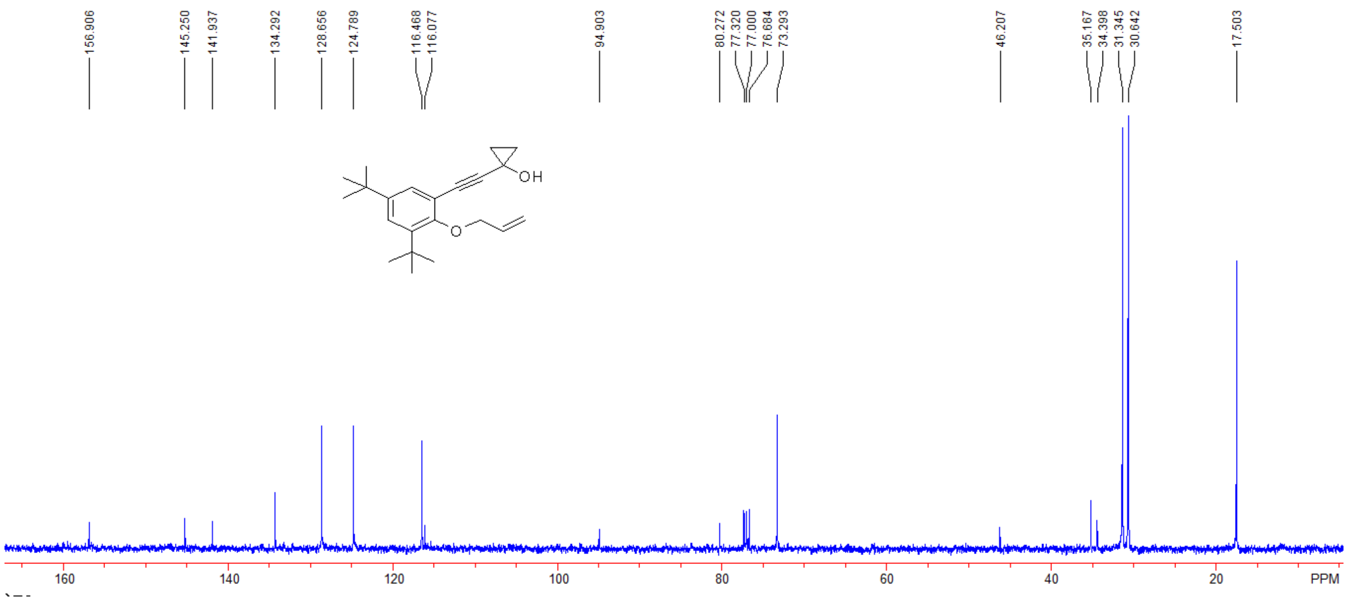

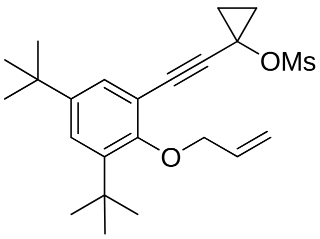

Compound **S4r**. 3.672 g, yield: 83%; yellow oil. ^{1}H NMR ($CDCl_3$, 400 MHz, TMS) δ 1.29 (s, 9H, ^{t}Bu), 1.34-1.38 (m, 11H, CH_2, ^{t}Bu), 1.64-1.67 (m, 2H, CH_2), 3.24 (s, 3H, CH_3), 4.72-4.73 (m, 2H, CH_2), 5.28-5.31 (m, 1H, =CH_2), 5.47-5.52 (m, 1H, =CH_2), 6.09-6.19 (m, 1H, =CH), 7.25 (d, J = 2.4 Hz, 1H, Ar), 7.37 (d, J = 2.4 Hz, 1H, Ar). ^{13}C NMR ($CDCl_3$, 100 MHz, TMS) δ 16.8, 30.5, 31.3, 34.3, 35.2, 39.9, 54.9, 73.6, 84.2, 89.6, 114.9, 116.8, 125.9, 128.8, 133.9, 142.3, 145.5, 157.4. IR (neat) ν 2960, 2906, 2869, 2226, 1438, 1362, 1201, 1173, 1158, 1122, 938, 909, 881, 795, 730 cm^{-1}. MS (%) m/e 404 (M^{+}, 3.44), 269 (15.83), 268 (18.17), 253 (30.75), 128 (12.61), 79 (12.29), 57 (100.00), 55 (12.61), 41 (26.12). HRMS (EI) calcd. for $C_{23}H_{32}O_4S$: 404.2021, Found: 404.2025.

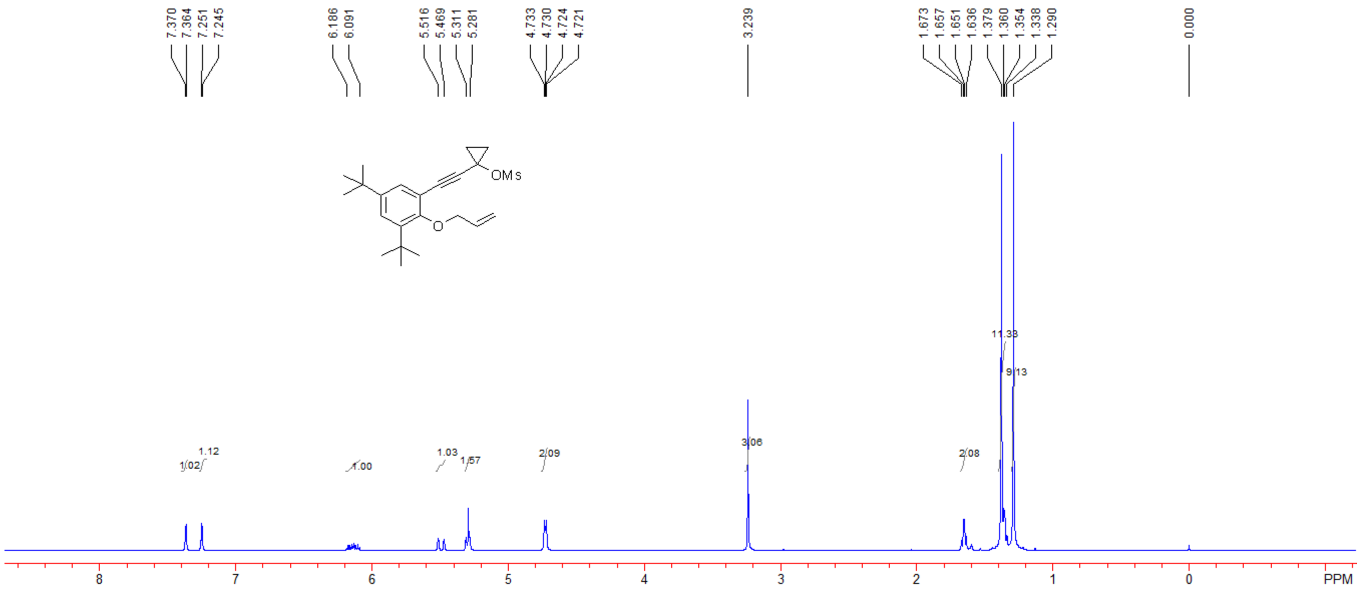

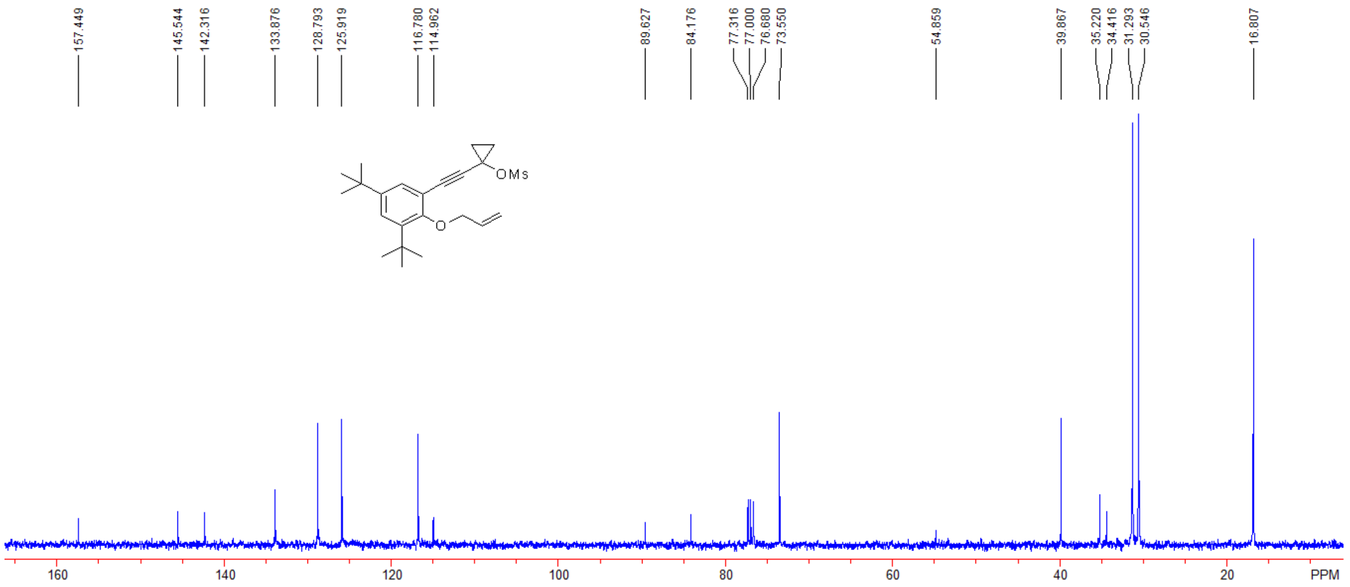

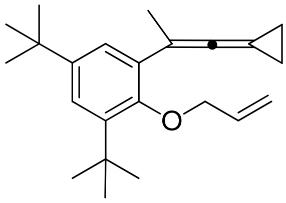

Compound **3a**. 745 mg, yield: 77%; colorless oil. ^{1}H NMR ($CDCl_3$, 400 MHz, TMS) δ 1.29 (s, 9H, ^{t}Bu), 1.40 (s, 9H, ^{t}Bu), 1.47-1.55 (m, 4H, 2CH_2), 2.14 (s, 3H, CH_3), 4.52-4.53 (m, 2H, CH_2), 5.23 (dd, J_1 = 1.6 Hz, J_2 = 10.8 Hz, 1H, =CH_2), 5.46 (dd, J_1 = 1.6 Hz, J_2 = 17.2 Hz, 1H, =CH_2), 6.05-6.14 (m, 1H, =CH), 7.12 (d, J = 2.4 Hz, 1H, Ar), 7.24 (d, J = 2.4 Hz, 1H, Ar). ^{13}C NMR ($CDCl_3$, 100 MHz, TMS) δ 7.3, 19.6, 30.8, 31.5, 34.4, 35.3, 72.4, 75.9, 103.1, 115.8, 123.1, 125.4, 132.9, 134.7, 142.1, 145.0, 153.1, 189.5. IR (neat) ν 2957, 2908, 2868, 2013, 1476, 1435, 1408,

1361, 1223, 1209, 1071, 987, 880, 863, 825, 741 cm^{-1}. MS (%) m/e 284 (25.33), 283 (83.71), 197 (31.15), 165 (24.06), 115 (24.30), 91 (25.91), 57 (100.00), 41 (25.32). HRMS (EI) calcd. for $C_{23}H_{32}O$: 324.2453, Found: 324.2452.

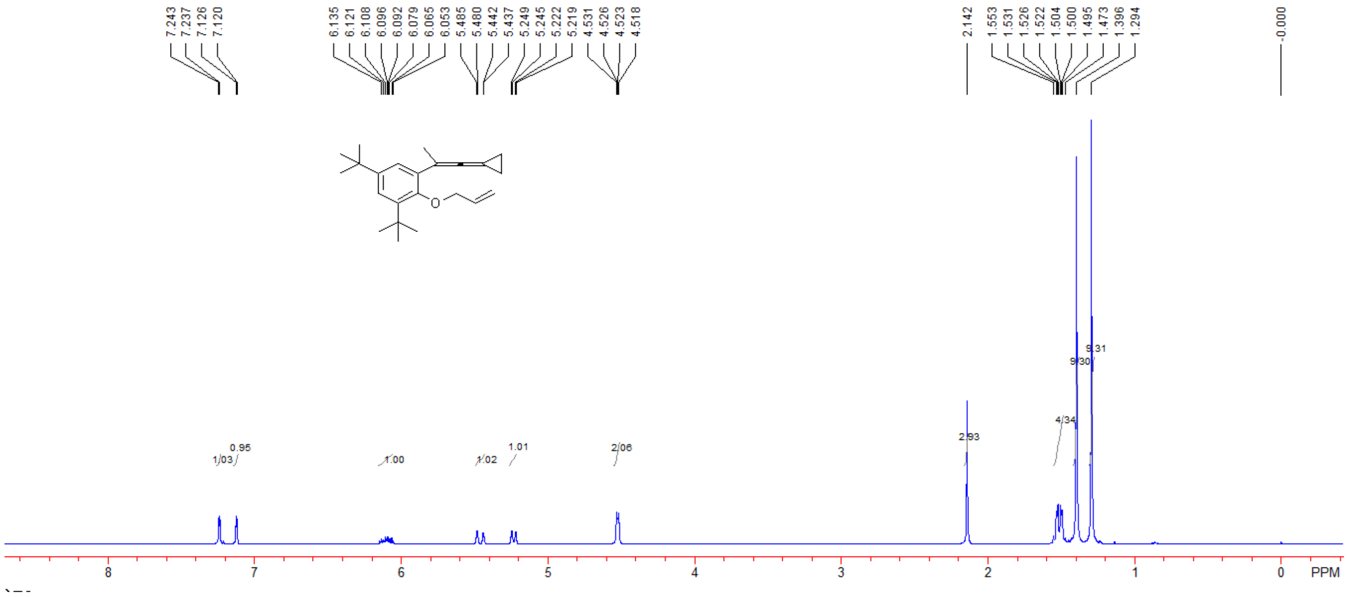

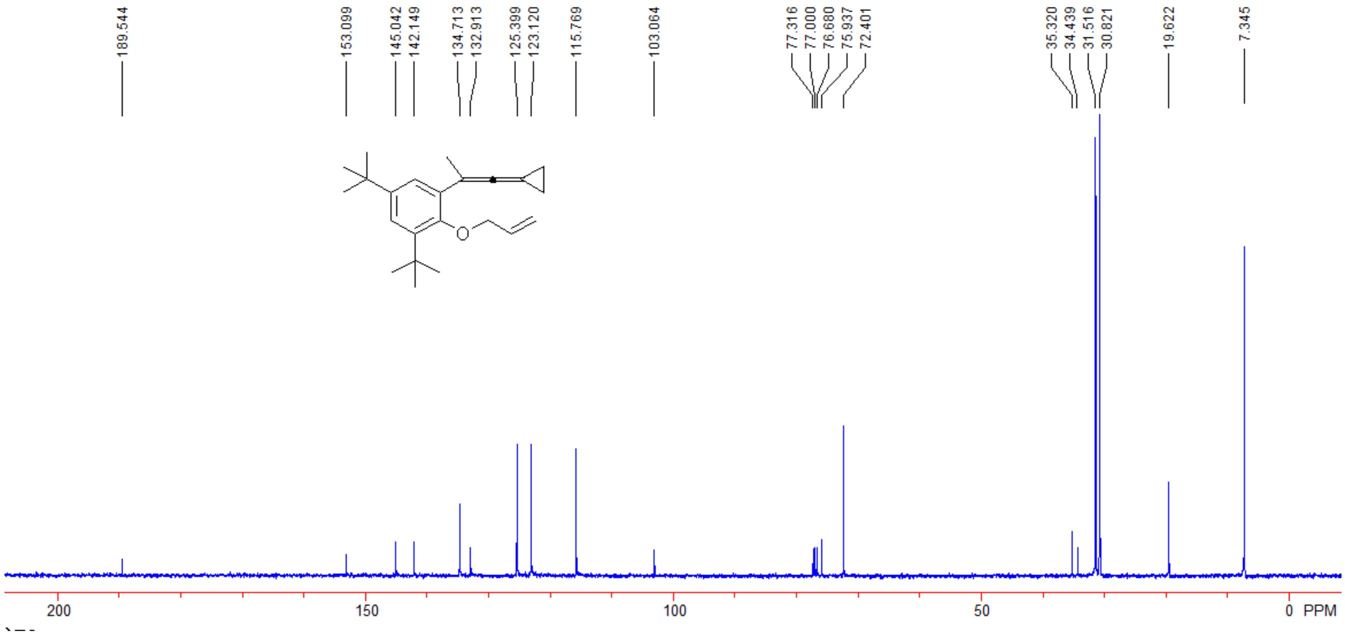

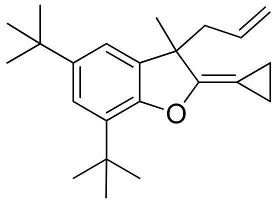

Compound **4a**. 133 mg, yield: 83%; colorless oil. ^{1}H NMR ($CDCl_3$, 400 MHz, TMS) δ 1.30 (s, 9H, ^{t}Bu), 1.39 (s, 12H, CH_3, ^{t}Bu), 2.34-2.50 (m, 4H, $2CH_2$), 2.79-2.89 (m, 2H, CH_2), 4.90 (s, 1H, $=CH_2$),

4.93-4.94 (m, 1H, $=CH_2$), 5.60-5.69 (m, 1H, =CH), 7.11-7.12 (m, 1H, Ar), 7.16-7.17 (m, 1H, Ar). ^{13}C NMR ($CDCl_3$, 100 MHz, TMS) δ 23.7, 28.3, 30.1, 31.2, 31.5, 34.5, 35.4, 37.6, 47.8, 115.9, 116.5, 121.7, 122.2, 129.2, 135.9, 136.5, 142.1, 144.1, 149.2. IR (neat) ν 2956, 2929, 2868, 1747, 1635, 1451, 1433, 1361, 1301, 1211, 1109, 1023, 910, 817, 762 cm^{-1}. MS (%) m/e 324 (M^+, 0.2), 284 (21.15), 283 (100.00), 268 (3.06), 267 (3.89), 253 (3.03), 134 (4.55), 120 (5.28), 57 (5.82). HRMS (EI) calcd. for $C_{23}H_{32}O$: 324.2453, Found: 324.2456; Enantiomeric excess was determined by HPLC with a Chiralcel PC-3 column [λ = 230 nm; eluent: water/methanol = 4/1; Flow rate: 0.7 mL/min; t_{minor} = 19.06 min, t_{major} = 21.90 min; ee% = 67%; $[\alpha]^{20}_D$ = -0.8235 (c 0.70, CH_2Cl_2)].

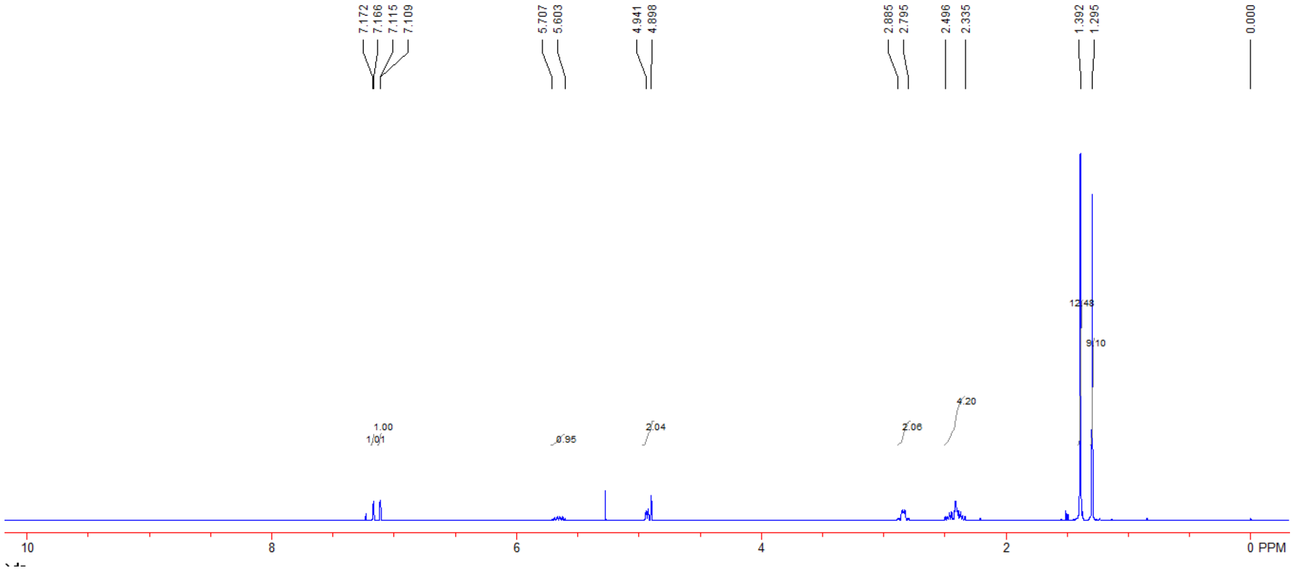

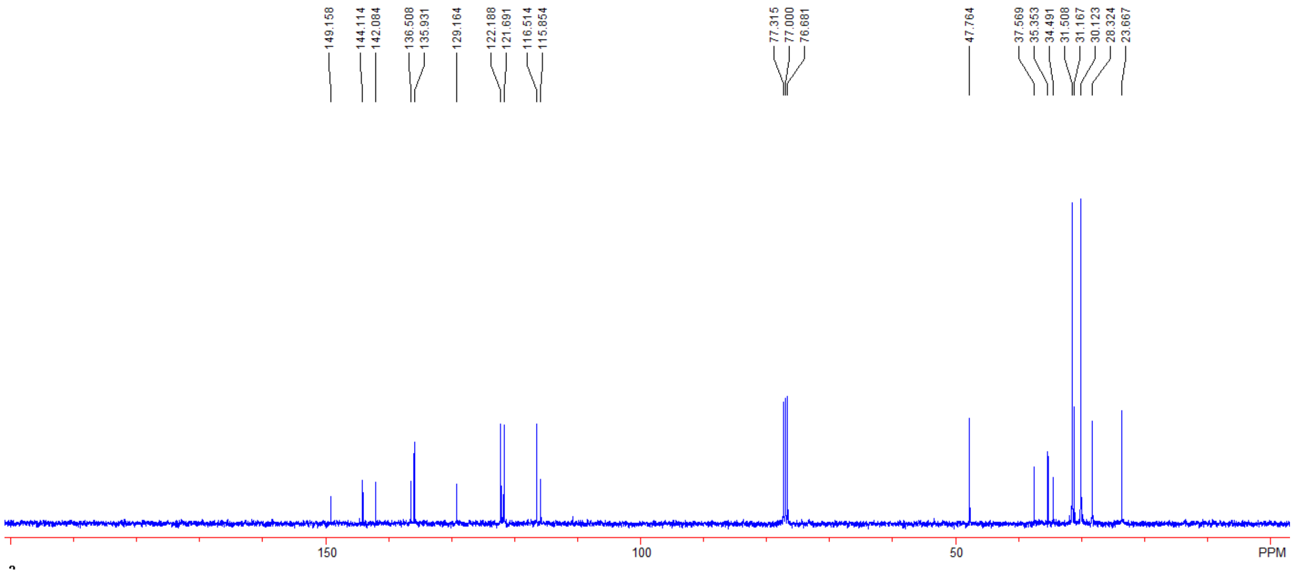

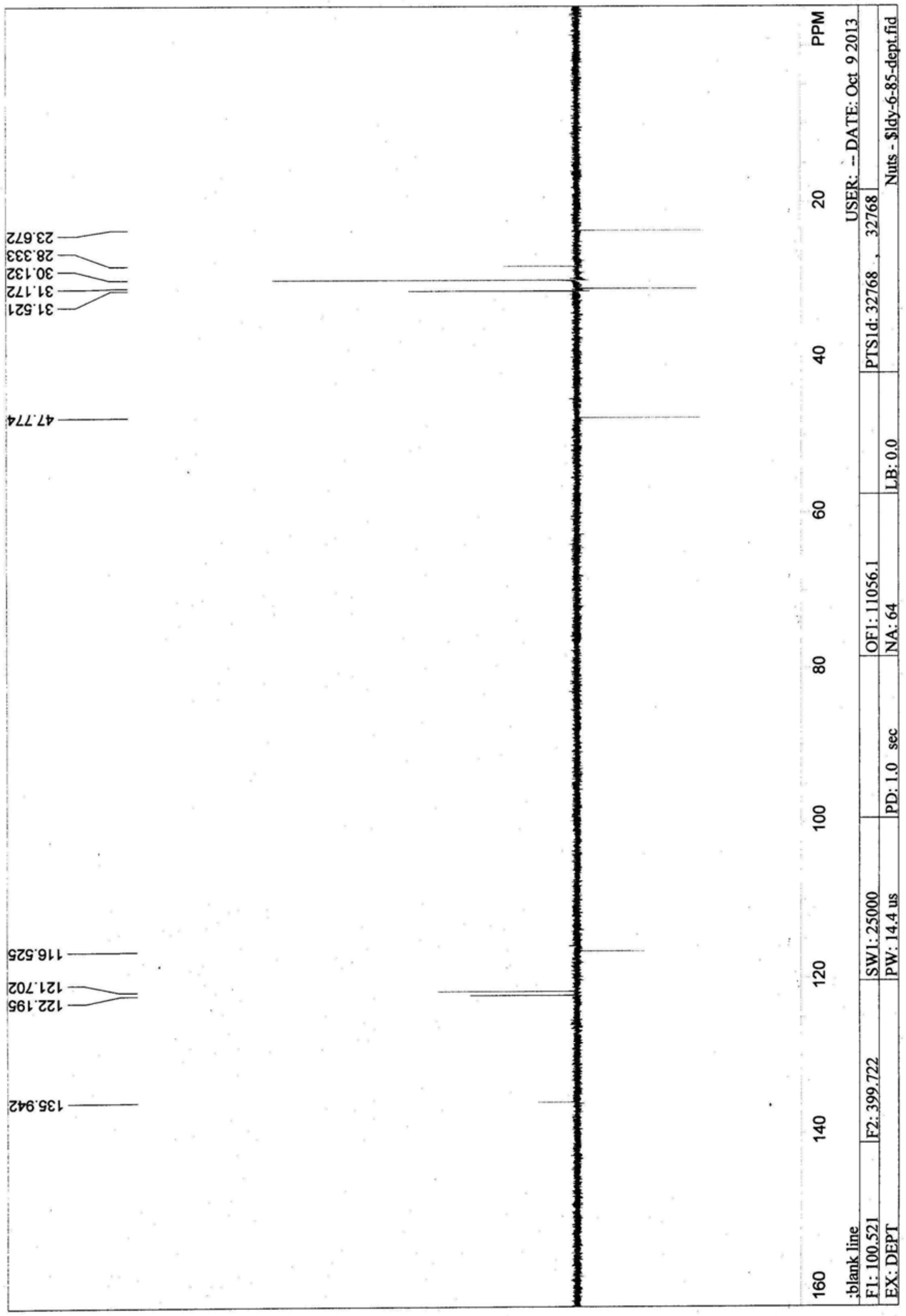
23.672
28.333
30.132
31.172
31.521
47.774
116.525
121.702
122.195
135.942
PPM
20
40
60
80
100
120
140
160
:blank line
F1: 100.521
F2: 399.722
SW1: 25000
OF1: 11056.1
PTS1d: 32768 , 32768
USER: -- DATE: Oct 9 2013
EX: DEPT
PW: 14.4 us
PD: 1.0 sec
NA: 64
LB: 0.0
Nuts - $ldy-6-85-dept.fid

C23H32O.

ldy-20140910

Sample Name:
ldy-20140910
Data Collected on:
Agilent-NMR-vnmrs400
Archive directory:
/home/omcl/vnmrsys/data
Sample directory:
ldy-20140910_20140910_01
FidFile: NOESY_01

Pulse Sequence: NOESY
Solvent: CDCl3
Data collected on: Sep 10 2014

Operator: omcl

Relax. delay 2.000 sec
Acq. time 0.246 sec
Width 4166.7 Hz
2D Width 4166.7 Hz
16 repetitions
2 x 128 increments
OBSERVE H1, 399.7200249 MHz
DATA PROCESSING
Line broadening 3.0 Hz
Gauss apodization 0.038 sec
F1 DATA PROCESSING
Gauss apodization 0.018 sec
FT size 2048 x 2048
Total time 3 hr, 25 min

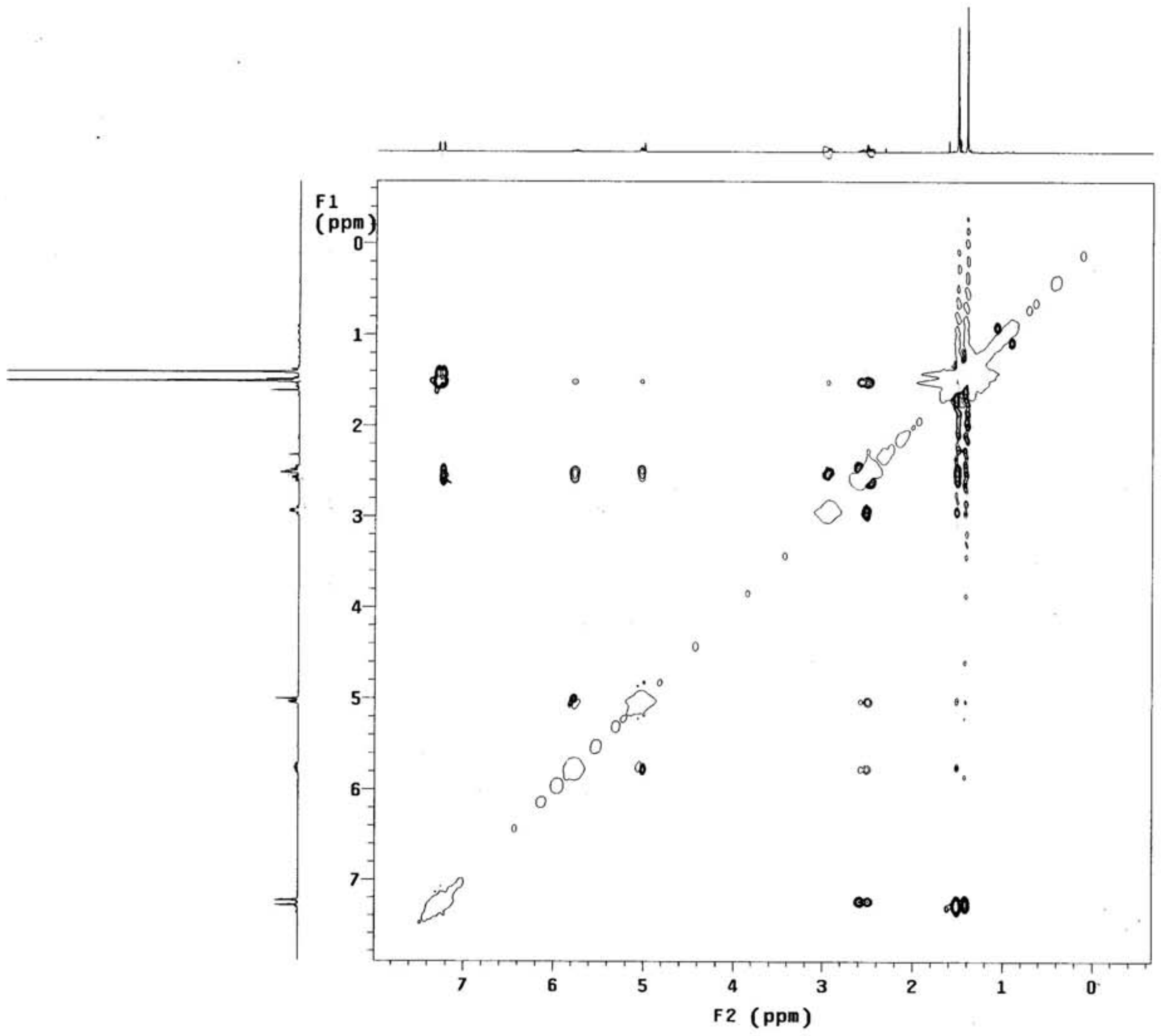

C23H32O

ldy-2014-9-9

Sample Name:
ldy-2014-9-9
Data Collected on:
Agilent-NMR-vnmrs400
Archive directory:
/home/omc1/vnmrsys/data
Sample directory:
ldy-2014-9-9_20140909_01
FidFile: gCOSY_01

Pulse Sequence: gCOSY
Solvent: CDCl3
Data collected on: Sep 9 2014

Operator: omc1

Relax. delay 1.000 sec
Acq. time 0.248 sec
Width 4139.1 Hz
2D Width 4139.1 Hz
2 repetitions
128 increments
OBSERVE H1, 399.7200249 MHz
DATA PROCESSING
Sq. sine bell 0.080 sec
F1 DATA PROCESSING
Sq. sine bell 0.031 sec
FT size 2048 x 2048
Total time 5 min 46 sec

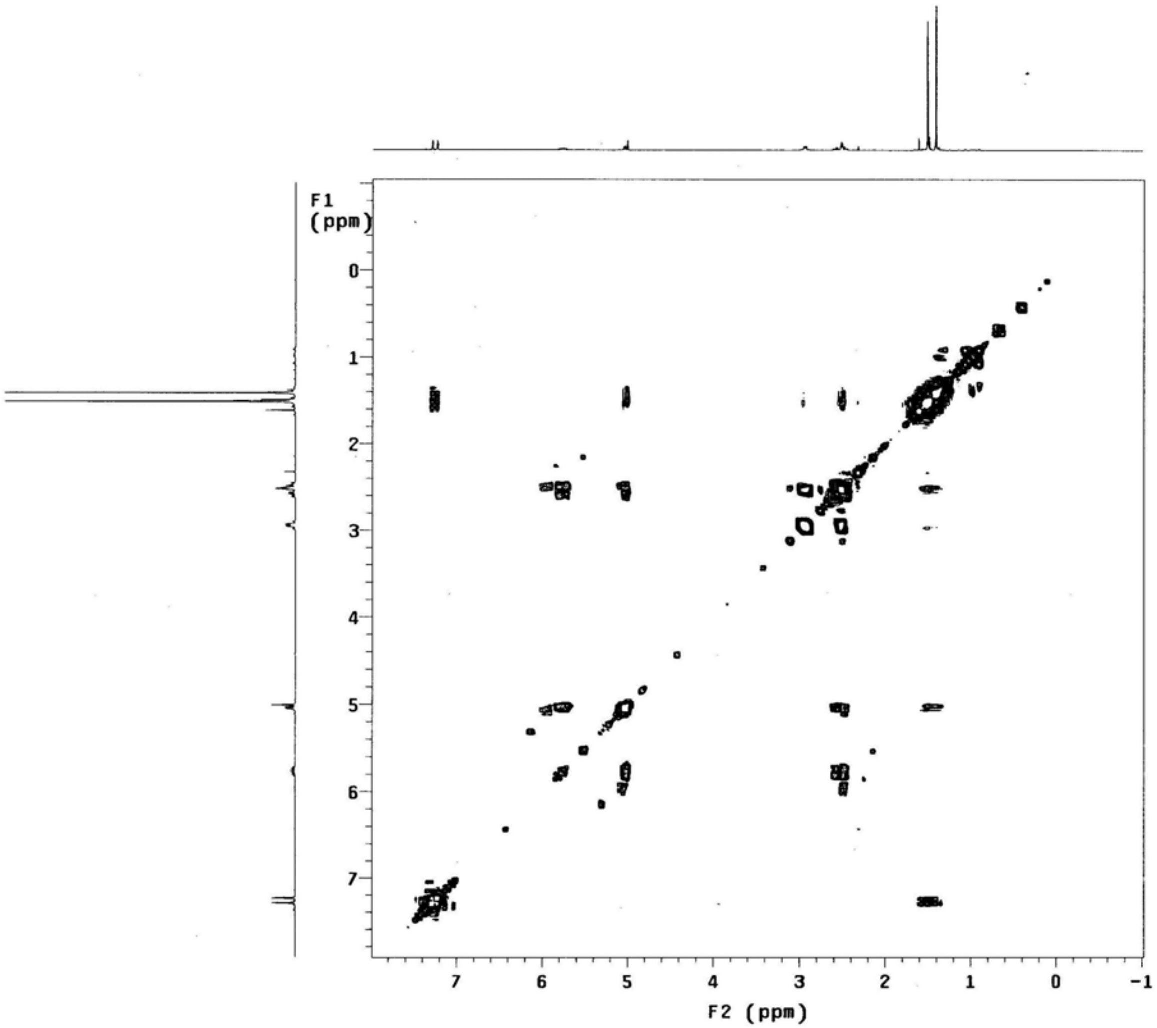

$C_{23}H_{32}O$.

ldy-20140910

Sample Name:
ldy-20140910
Data Collected on:
Agilent-NMR-vnmrs400
Archive directory:
/home/omc1/vnmrsys/data
Sample directory:
ldy-20140910_20140910_01
FidFile: gHSQCAD_01

Pulse Sequence: gHSQCAD
Solvent: CDCl3
Data collected on: Sep 11 2014

Operator: omc1

Relax. delay 1.000 sec
Acq. time 0.213 sec
Width 4807.7 Hz
2D Width 20100.5 Hz
16 repetitions
2 x 128 increments
OBSERVE H1, 399.7200249 MHz
DECOUPLE C13, 100.5187261 MHz
Power 36 dB
on during acquisition
off during delay
W40_4nuc modulated
DATA PROCESSING
Gauss apodization 0.074 sec
F1 DATA PROCESSING
Gauss apodization 0.006 sec
FT size 2048 x 2048
Total time 1 hr, 23 min

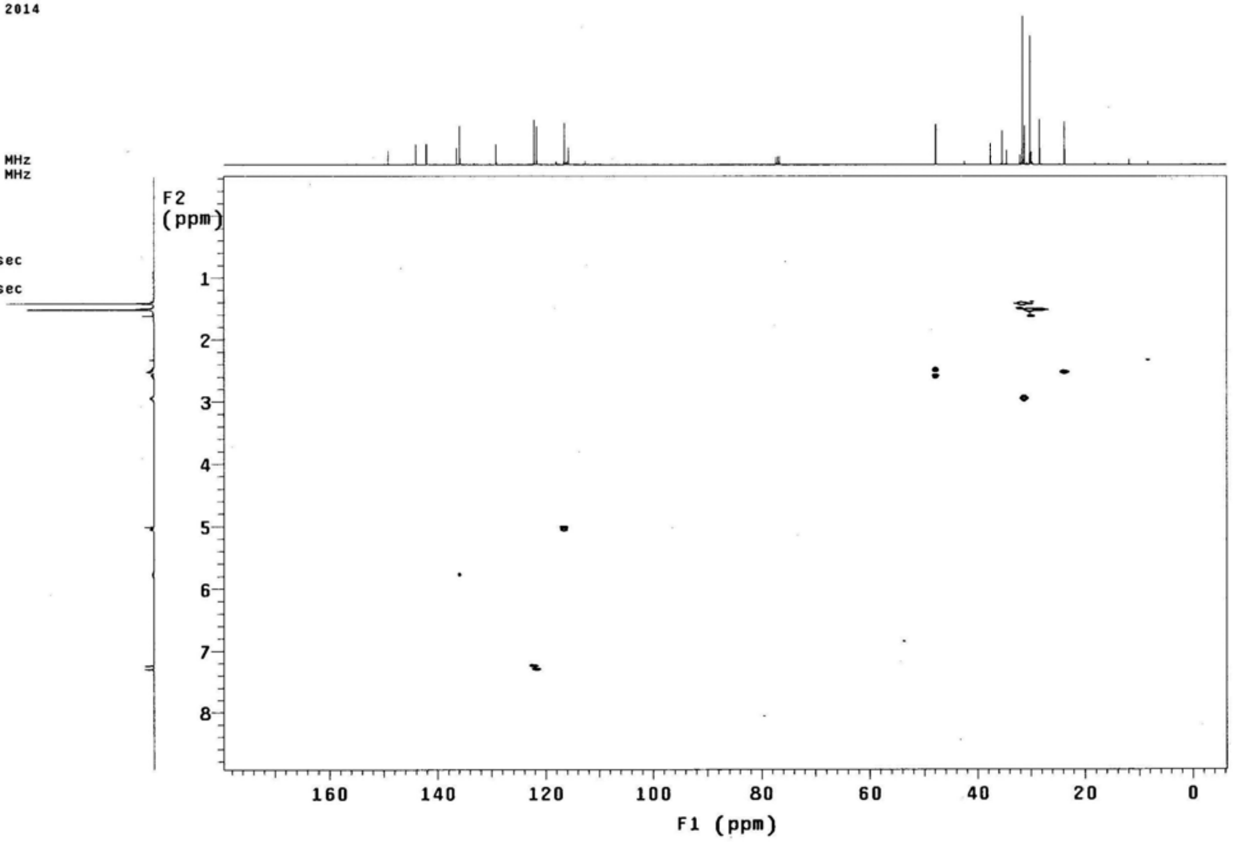

ldy-20140910

Sample Name:
ldy-20140910
Data Collected on:
Agilent-NMR-vnmrs400
Archive directory:
/home/omc1/vnmrsys/data
Sample directory:
ldy-20140910_20140910_01
FidFile: gHSQCAD_01

Pulse Sequence: gHSQCAD
Solvent: CDCl3
Data collected on: Sep 11 2014

Operator: omc1

Relax. delay 1.000 sec
Acq. time 0.213 sec
Width 4807.7 Hz
2D Width 20100.5 Hz
16 repetitions
2 x 128 increments
OBSERVE H1, 399.7200249 MHz
DECOUPLE C13, 100.5187261 MHz
Power 36 dB
on during acquisition
off during delay
W40_4nuc modulated
DATA PROCESSING
Gauss apodization 0.074 sec
F1 DATA PROCESSING
Gauss apodization 0.006 sec
FT size 2048 x 2048
Total time 1 hr, 23 min

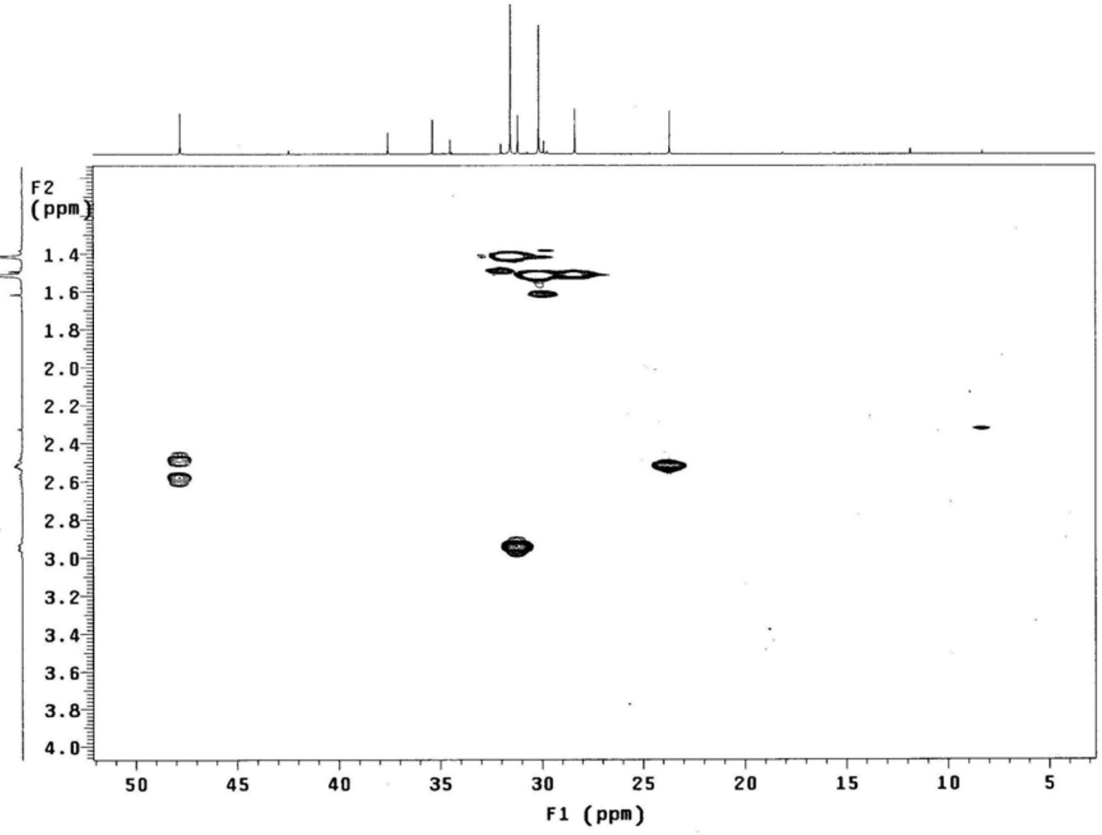

ldy-20140910

Sample Name:
ldy-20140910
Data Collected on:
Agilent-NMR-vnmrs400
Archive directory:
/home/omc1/vnmrsys/data
Sample directory:
ldy-20140910_20140910_01
FidFile: gHSQCAD_01

Pulse Sequence: gHSQCAD
Solvent: CDCl3
Data collected on: Sep 11 2014

Operator: omc1

Relax. delay 1.000 sec
Acq. time 0.213 sec
Width 4807.7 Hz
2D Width 20100.5 Hz
16 repetitions
2 x 128 increments
OBSERVE H1, 399.7200249 MHz
DECOUPLE C13, 100.5187261 MHz
Power 36 dB
on during acquisition
off during delay
W40_4nuc modulated
DATA PROCESSING
Gauss apodization 0.074 sec
F1 DATA PROCESSING
Gauss apodization 0.006 sec
FT size 2048 x 2048
Total time 1 hr, 23 min

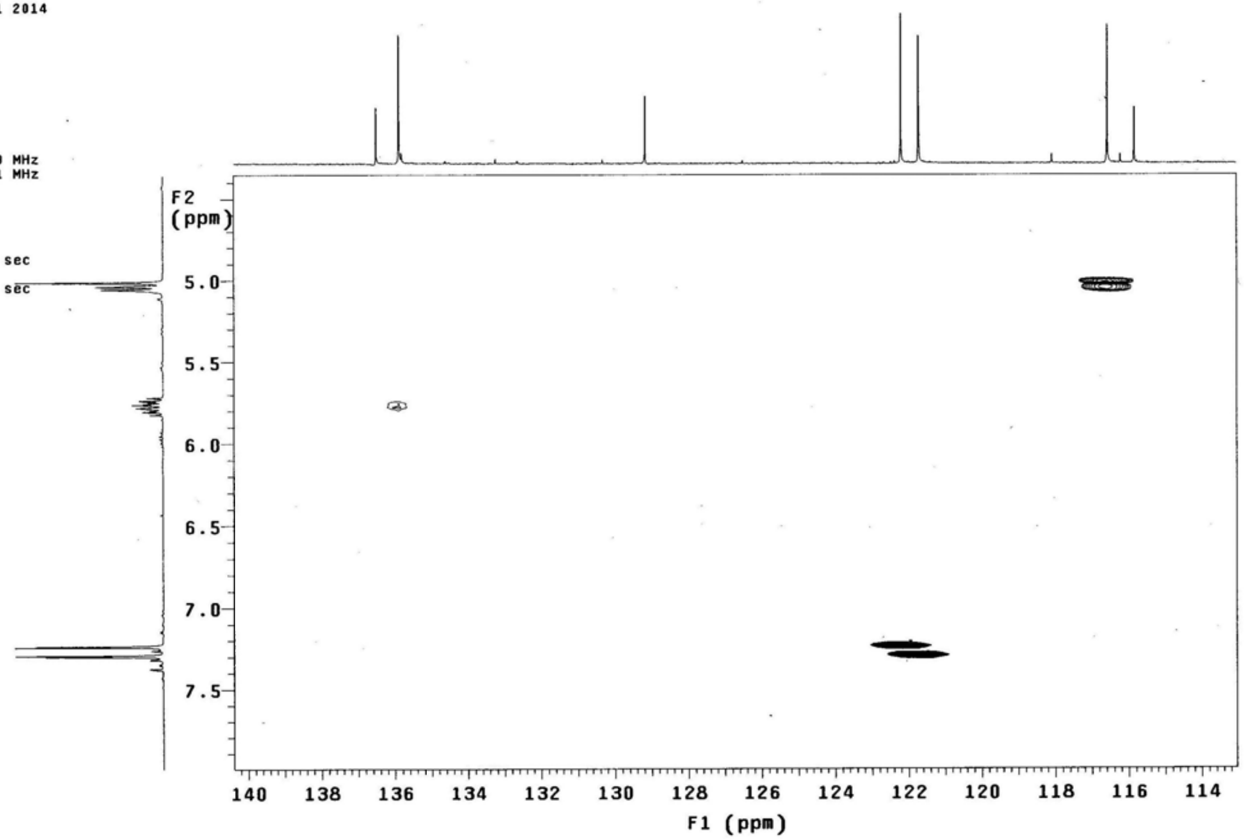

$C_{23}H_{32}O$

ldy-20140910

Sample Name:
ldy-20140910
Data Collected on:
Agilent-NMR-vnmrs400
Archive directory:
/home/omc1/vnmrsys/data
Sample directory:
ldy-20140910_20140910_01
FidFile: gHMBCAD_01

Pulse Sequence: gHMBCAD
Solvent: CDCl3
Data collected on: Sep 11 2014

Operator: omc1

Relax. delay 1.000 sec
Acq. time 0.213 sec
Width 4807.7 Hz
2D Width 24125.5 Hz
16 repetitions
2 x 128 increments
OBSERVE H1, 399.7200249 MHz
DATA PROCESSING
Sq. sine bell 0.080 sec
F1 DATA PROCESSING
Gauss apodization 0.005 sec
FT size 2048 x 2048
Total time 1 hr, 25 min

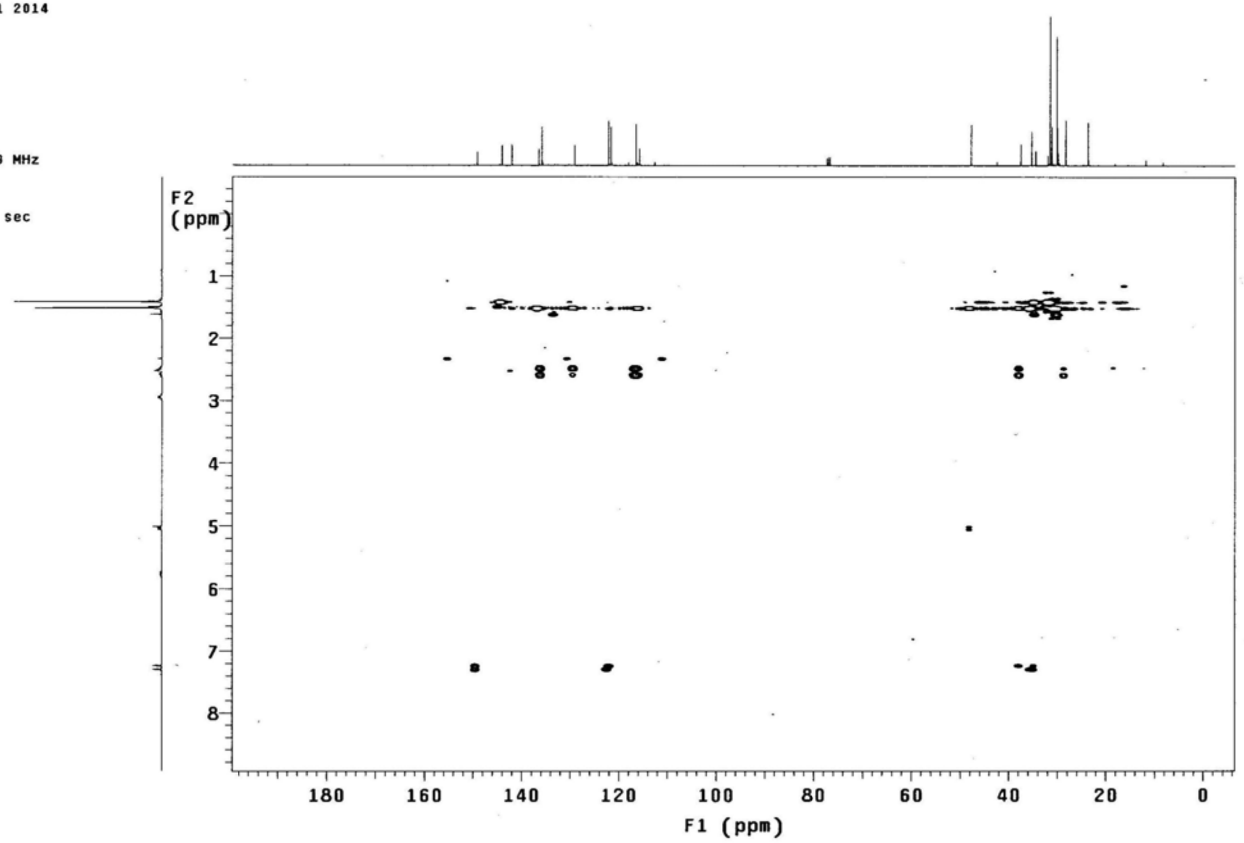

ldy-20140910

Sample Name:
ldy-20140910
Data Collected on:
Agilent-NMR-vnmrs400
Archive directory:
/home/omc1/vnmrsys/data
Sample directory:
ldy-20140910_20140910_01
FidFile: gHMBCAD_01

Pulse Sequence: gHMBCAD
Solvent: CDCl3
Data collected on: Sep 11 2014

Operator: omc1

Relax. delay 1.000 sec
Acq. time 0.213 sec
Width 4807.7 Hz
2D Width 24125.5 Hz
16 repetitions
2 x 128 increments
OBSERVE H1, 399.7200249 MHz
DATA PROCESSING
Sq. sine bell 0.080 sec
F1 DATA PROCESSING
Gauss apodization 0.005 sec
FT size 2048 x 2048
Total time 1 hr, 25 min

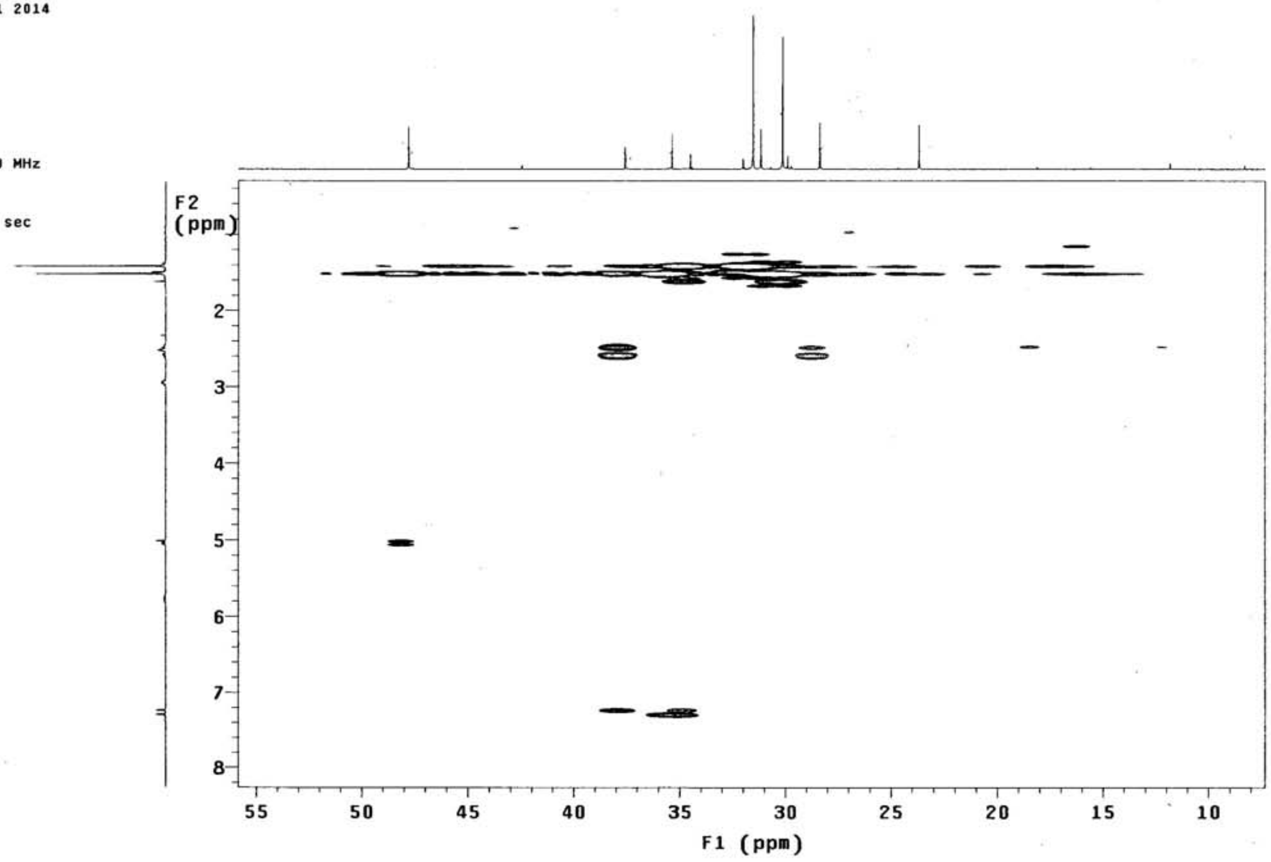

ldy-20140910

Sample Name:
ldy-20140910
Data Collected on:
Agilent-NMR-vnmrs400
Archive directory:
/home/omc1/vnmrsys/data
Sample directory:
ldy-20140910_20140910_01
FidFile: gHMBCAD_01

Pulse Sequence: gHMBCAD
Solvent: CDCl3
Data collected on: Sep 11 2014

Operator: omc1

Relax. delay 1.000 sec
Acq. time 0.213 sec
Width 4807.7 Hz
2D Width 24125.5 Hz
16 repetitions
2 x 128 increments
OBSERVE H1, 399.7200249 MHz
DATA PROCESSING
Sq. sine bell 0.080 sec
F1 DATA PROCESSING
Gauss apodization 0.005 sec
FT size 2048 x 2048
Total time 1 hr, 25 min

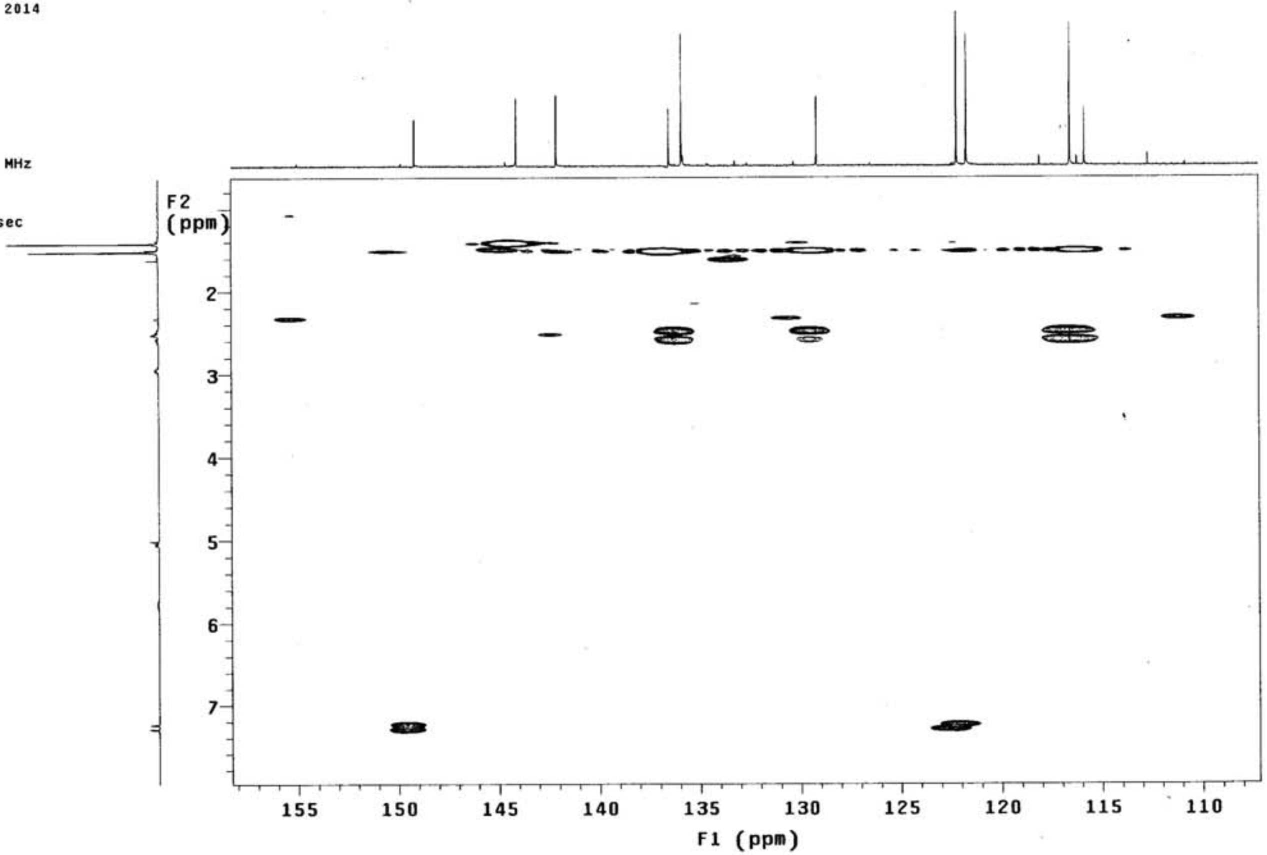

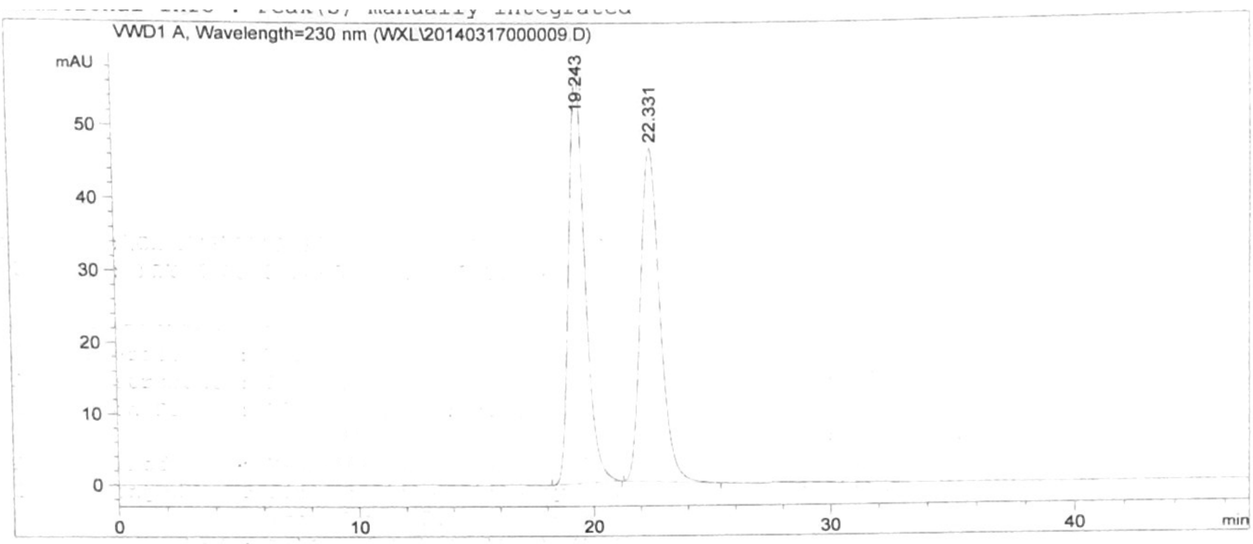


```
=====================================================================
                         Area Percent Report
=====================================================================

Sorted By             :      Signal
Multiplier:                  :      1.0000
Dilution:                    :      1.0000
Use Multiplier & Dilution Factor with ISTDs


Signal 1: VWD1 A, Wavelength=230 nm
```

Peak #	RetTime [min]	Type	Width [min]	Area [mAU*s]	Height [mAU]	Area %
1	19.243	MM	0.8082	2754.12793	56.79293	50.7969
2	22.331	BB	0.8232	2667.71094	46.57891	49.2031

Totals : 5421.83887 103.37184

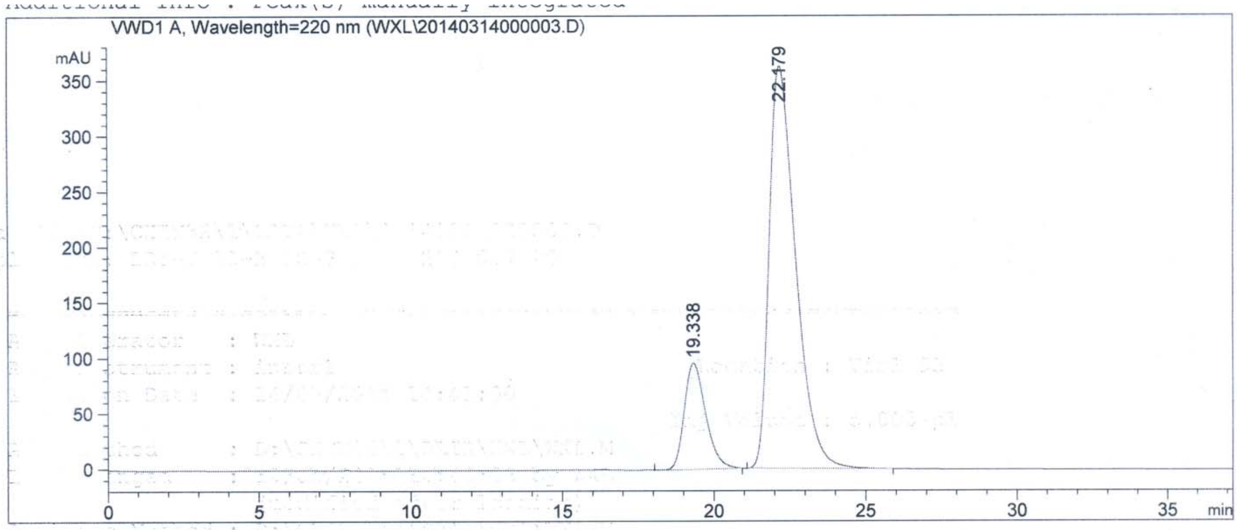

Area Percent Report

Sorted By : Signal
Multiplier: : 1.0000
Dilution: : 1.0000
Use Multiplier & Dilution Factor with ISTDs

Signal 1: VWD1 A, Wavelength=220 nm

Peak #	RetTime [min]	Type	Width [min]	Area [mAU*s]	Height [mAU]	Area %
1	19.338	MM	0.8164	4684.03076	95.62507	17.6107
2	22.179	MM	1.0094	2.19136e4	361.83035	82.3893
Totals :				2.65977e4	457.45542	

*** End of Report ***

Translation: Chiralcel PC-3 column [λ = 230 nm; eluent: water/methanol = 4/1; Flow rate: 0.7 mL/min; t_{minor} = 19.06 min, t_{major} = 21.90 min; ee% = 67%.]

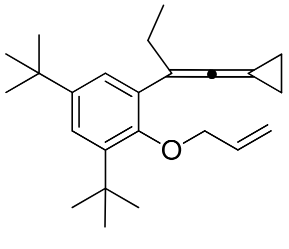

Compound **3b**. 372 mg, yield: 55%; colorless oil. ^{1}H NMR ($CDCl_3$, 400 MHz, TMS) δ 1.04 (t, J = 7.6 Hz, 3H, CH_3), 1.29 (s, 9H, ^{t}Bu), 1.39 (s, 9H, ^{t}Bu), 1.50-1.54 (m, 4H, 2CH_2), 2.48 (q, J = 7.6 Hz, 2H, CH_2), 4.51-4.53 (m, 2H, CH_2), 5.22 (dd, J_1 = 1.6 Hz, J_2 = 10.8 Hz, 1H, =CH_2), 5.44 (dd, J_1 =

1.6 Hz, J_2 = 17.2 Hz, 1H, =CH_2), 6.04-6.13 (m, 1H, =CH), 7.10 (d, J = 2.4 Hz, 1H, Ar), 7.24 (d, J = 2.4 Hz, 1H, Ar). ^{13}C NMR ($CDCl_3$, 100 MHz, TMS) δ 7.0, 12.4, 25.2, 30.5, 31.2, 34.1, 35.0, 72.2, 76.4, 109.8, 115.5, 122.7, 125.5, 132.2, 134.4, 141.7, 144.7, 152.7, 188.3. IR (neat) ν 2960, 2904, 2870, 2016, 1643, 1345, 1370, 1361, 1224, 1204, 1014, 982, 909, 850, 651 cm^{-1}. MS (%) m/e 338 (M^+, 0.45), 298 (23.46), 297 (100.00), 281 (6.66), 267 (9.33), 185 (6.74), 165 (7.25), 57 (71.09), 41 (15.36). HRMS (EI) calcd. for $C_{24}H_{34}O$: 338.2610, Found: 338.2614.

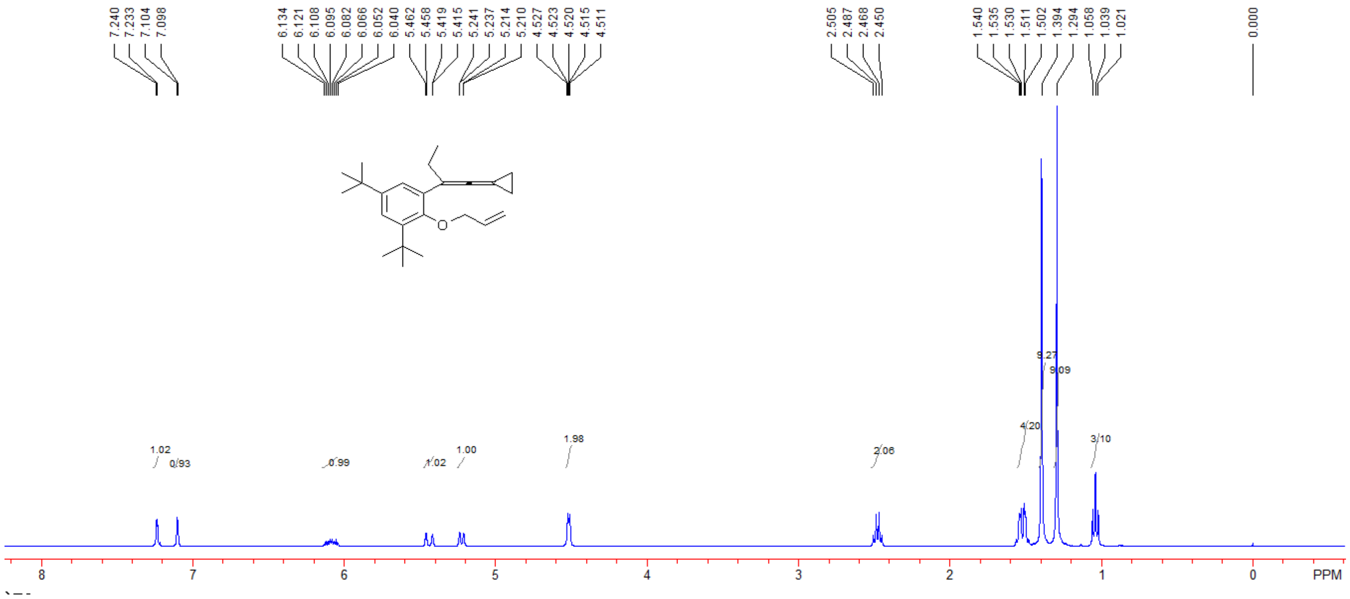

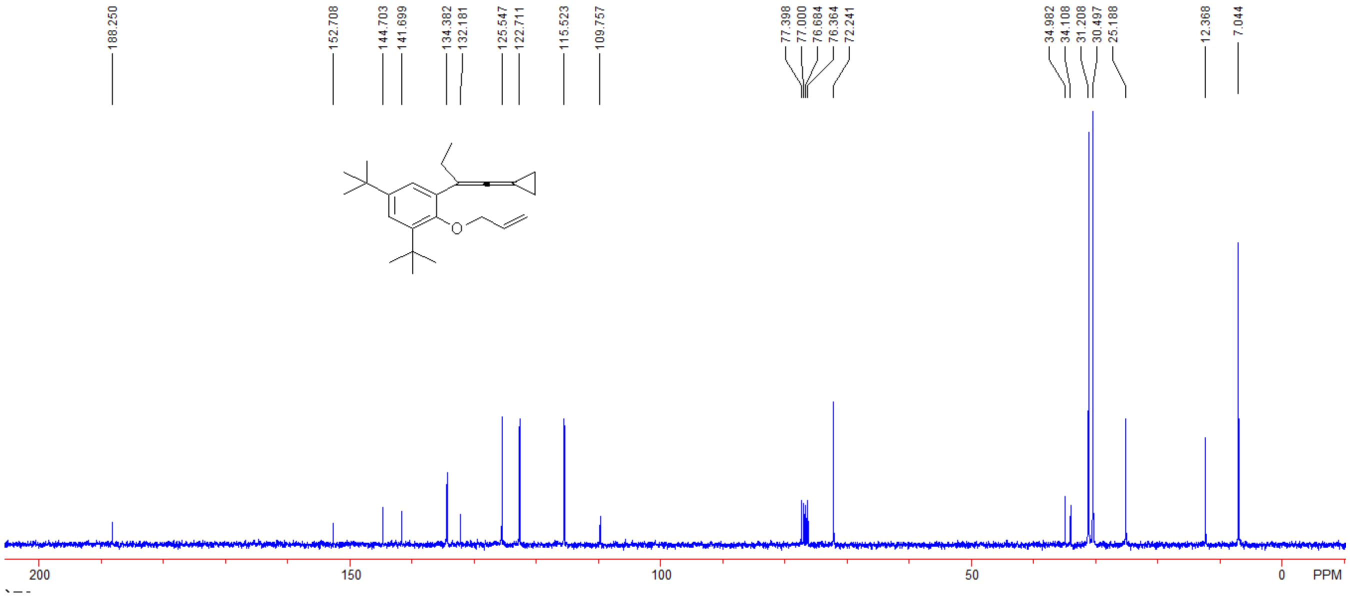

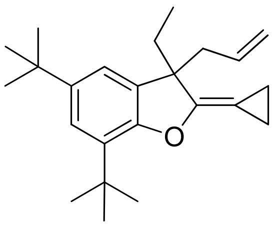

Compound **4b**. 125 mg, yield: 74%; colorless oil. ^{1}H NMR ($CDCl_3$, 400 MHz, TMS) δ 0.69 (t, *J* = 7.2 Hz, 3H, CH_3), 1.28 (s, 9H, ^{t}Bu), 1.39 (s, 9H, ^{t}Bu), 1.67 (q, *J* = 7.2 Hz, 2H, CH_2), 2.36-2.46 (m, 4H, $2CH_2$), 2.87-2.88 (m, 2H, CH_2), 4.87-4.93 (m, 2H, =CH_2), 5.59-5.68 (m, 1H, =CH), 7.03 (d, *J* = 2.4 Hz, 1H, Ar), 7.14 (d, *J* = 2.4 Hz, 1H, Ar). ^{13}C NMR ($CDCl_3$, 100 MHz, TMS) δ 9.9, 23.9, 30.1, 31.2, 31.5, 33.3, 34.4, 35.2, 42.6, 46.6, 113.6, 116.3, 121.4, 121.9, 126.7, 136.0, 136.3, 144.0, 144.3, 150.4. IR (neat) ν 2959, 2930, 2871, 1746, 1455, 1433, 1362, 1307, 1280, 1210, 1103, 907, 879, 817, 733 cm^{-1}. MS (%) m/e 338 (M$^+$, 0.22), 309 (5.52), 298 (24.03), 297 (100.00), 281 (4.91), 141 (5.89), 127 (3.96), 57 (8.54), 41 (3.70). HRMS (EI) calcd. for $C_{24}H_{34}O$: 338.2610, Found: 338.2615.

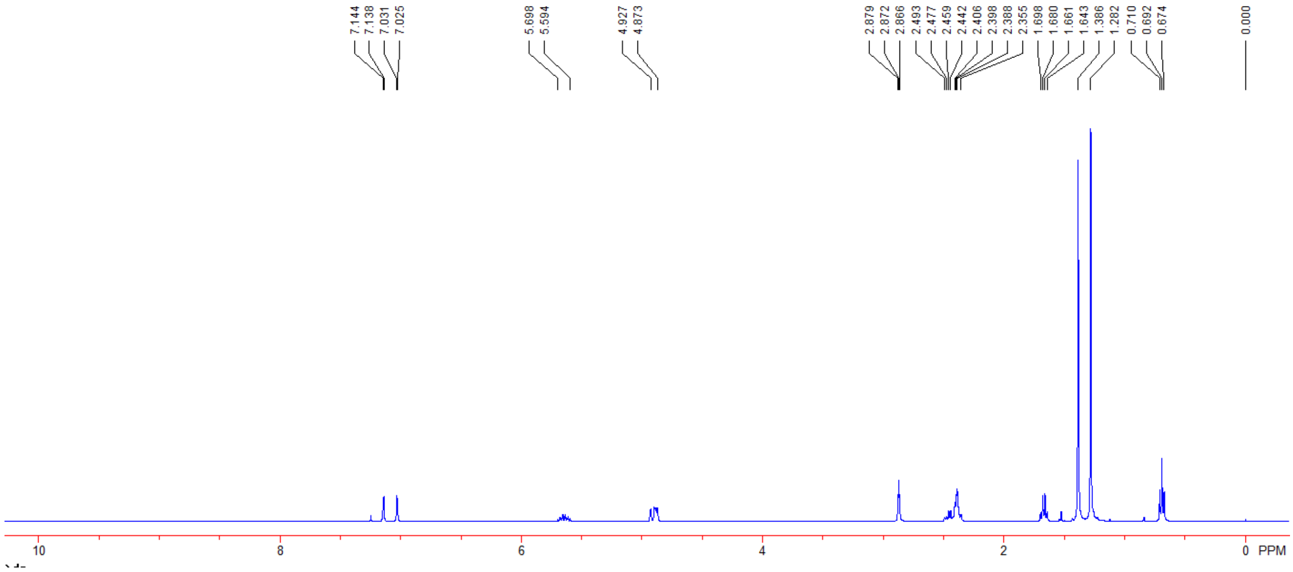

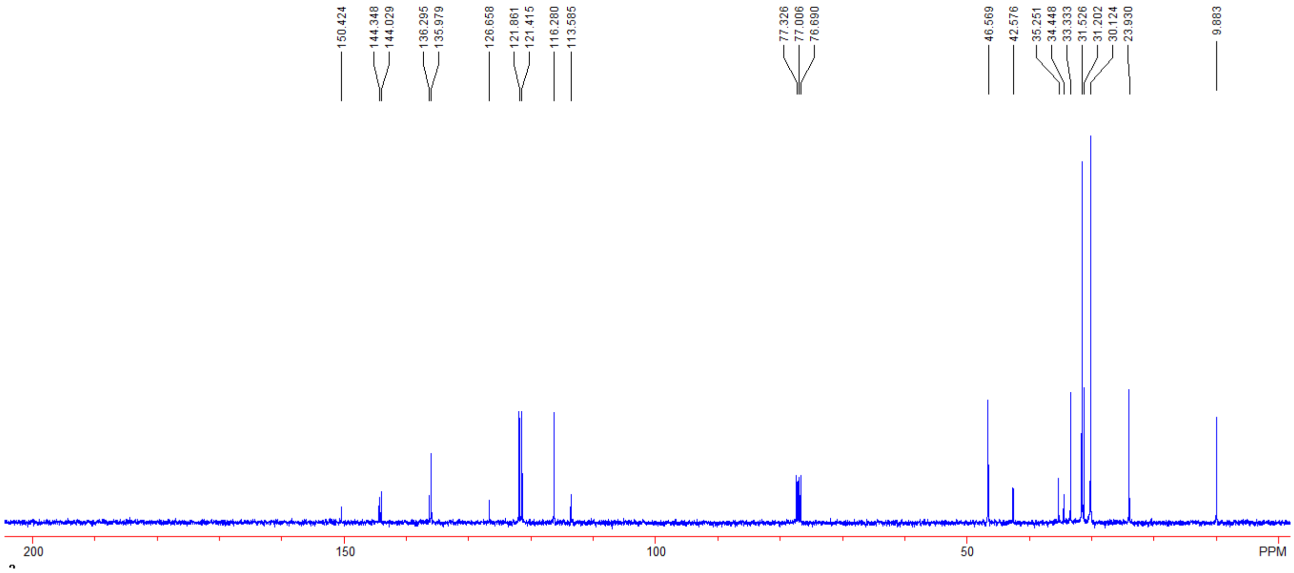

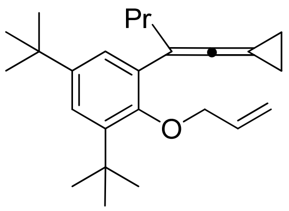

Compound **3c**. 404 mg, yield: 57%; colorless oil. ^{1}H NMR ($CDCl_3$, 400 MHz, TMS) δ 0.93 (t, J = 7.6 Hz, 3H, CH_3), 1.29 (s, 9H, ^{t}Bu), 1.39 (s, 9H, ^{t}Bu), 1.43-1.55 (m, 6H, 3CH_2), 2.44 (t, J = 7.6 Hz, 2H, CH_2), 4.52-4.54 (m, 2H, CH_2), 5.23 (dd, J_1 = 1.2 Hz, J_2 = 10.8 Hz, 1H, =CH_2), 5.45 (dd, J_1 = 1.2 Hz, J_2 = 17.2 Hz, 1H, =CH_2), 6.04-6.14 (m, 1H, =CH), 7.09-7.10 (m, 1H, Ar), 7.23 (d, J = 2.4 Hz, 1H, Ar). ^{13}C NMR ($CDCl_3$, 100 MHz, TMS) δ 7.3, 13.9, 21.4, 30.8, 31.5, 34.42, 34.46, 35.3, 72.5, 77.1, 108.4, 115.8, 123.0, 125.9, 132.5, 134.7, 142.0, 145.0, 153.0, 188.8. IR (neat) ν 2956, 2869, 2012, 1463, 1435, 1390, 1361, 1222, 1204, 1107, 1004, 987, 916, 880, 740 cm^{-1}. MS (%) m/e 352 (M^+, 1.60), 312 (24.56), 311 (100.00), 296 (5.88), 281 (9.13), 199 (9.30), 165 (7.57), 57 (79.09), 41 (14.65). HRMS (EI) calcd. for $C_{25}H_{36}O$: 352.2766, Found: 352.2762.

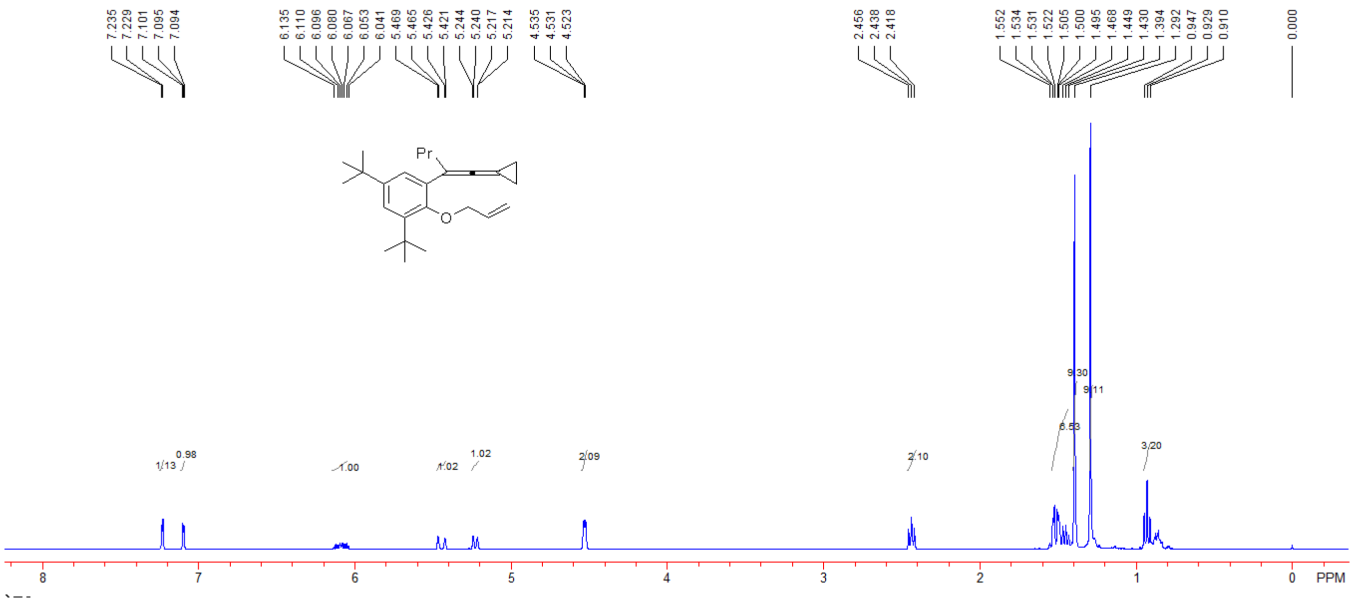

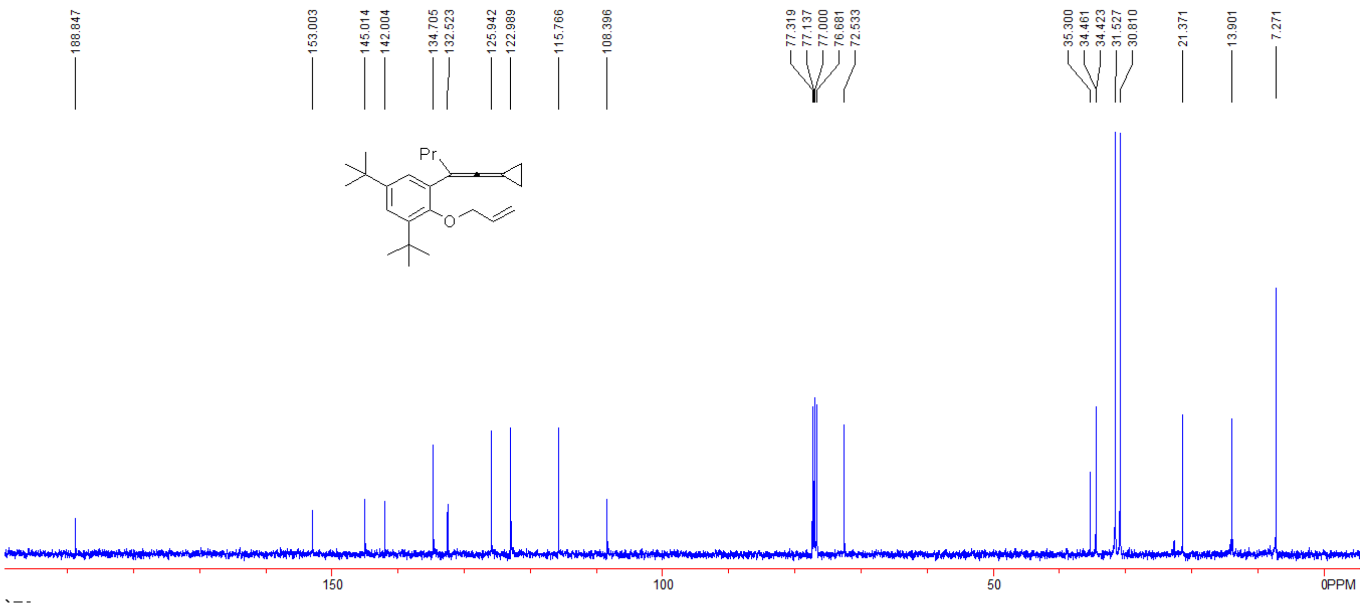

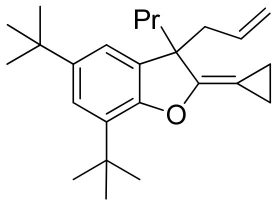

Compound **4c**. 123 mg, yield: 70%; colorless oil. ^{1}H NMR ($CDCl_3$, 400 MHz, TMS) δ 0.81 (t, J = 7.2 Hz, 3H, CH_3), 0.95-1.04 (m, 1H, CH_2), 1.17-1.26 (m, 1H, CH_2), 1.28 (s, 9H, ^{t}Bu), 1.38 (s, 9H, ^{t}Bu), 1.60-1.64 (m, 2H, CH_2), 2.36-2.45 (m, 4H, 2CH_2), 2.85-2.87 (m, 2H, CH_2), 4.88-4.93 (m, 2H, =CH_2), 5.61-5.67 (m, 1H, =CH), 7.05 (d, J = 2.4 Hz, 1H, Ar), 7.14 (d, J = 2.4 Hz, 1H, Ar). ^{13}C NMR ($CDCl_3$, 100 MHz, TMS) δ 14.6, 18.9, 24.0, 30.1, 31.2, 31.5, 34.4, 35.2, 42.0, 43.4, 46.8,

114.1, 116.3, 121.4, 121.9, 127.1, 135.9, 136.3, 143.8, 144.0, 150.2. IR (neat) ν 2954, 2929, 2870, 1746, 1639, 1433, 1362, 1308, 1284, 1210, 1111, 993, 909, 817, 765 cm^{-1}. MS (%) m/e 352 (M^+, 0.31), 312 (24.42), 311 (100.00), 309 (5.85), 255 (6.19), 148 (5.00), 134 (5.53), 57 (18.14), 41 (5.90). HRMS (EI) calcd. for $C_{25}H_{36}O$: 352.2766, Found: 352.2764.

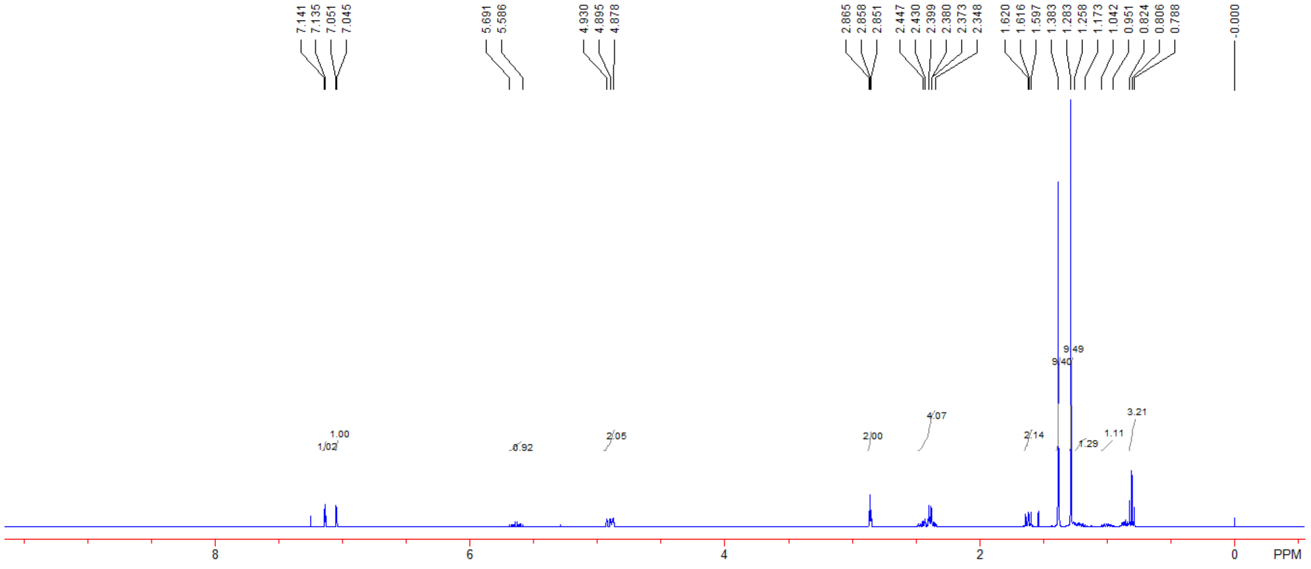

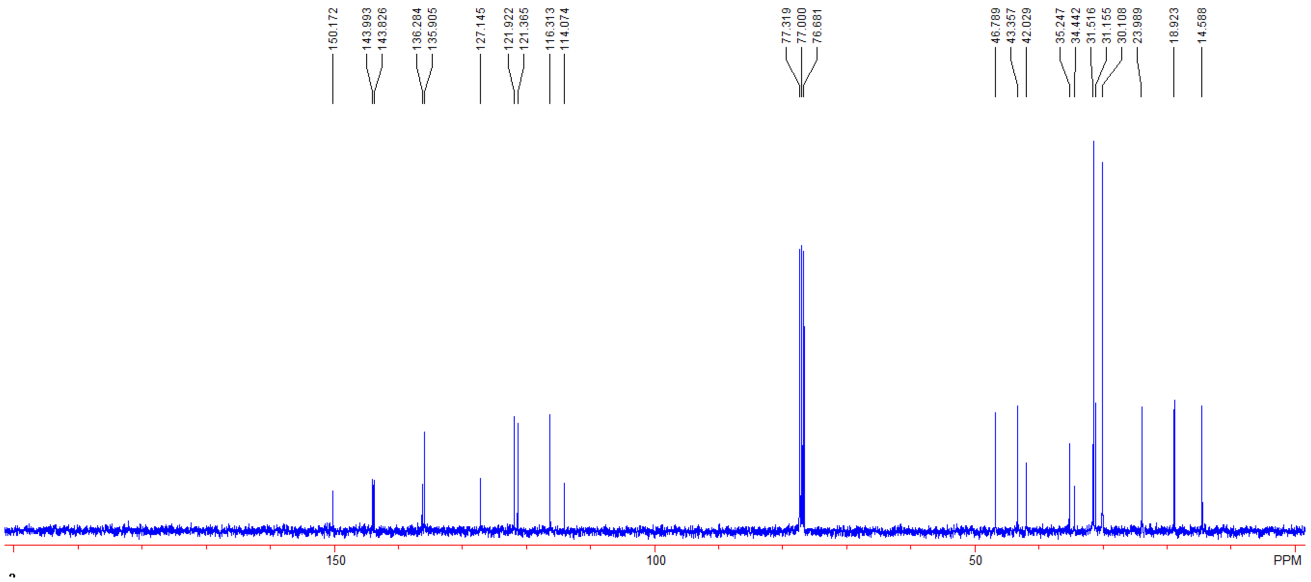

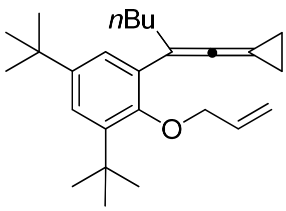

Compound **3d**. 437 mg, yield: 60%; colorless oil. ^{1}H NMR ($CDCl_3$, 400 MHz, TMS) δ 0.88 (t, J = 6.8 Hz, 3H, CH_3), 1.29 (s, 9H, ^{t}Bu), 1.34-1.43 (m, 13H, 2CH_2, ^{t}Bu), 1.49-1.55 (m, 4H, 2CH_2), 2.46 (t, J = 6.8 Hz, 2H, CH_2), 4.52 (d, J = 5.2 Hz, 2H, CH_2), 5.23 (dd, J_1 = 1.2 Hz, J_2 = 10.8 Hz, 1H,

=CH_2), 5.45 (dd, J_1 = 2.0 Hz, J_2 = 15.2 Hz, 1H, =CH_2), 6.04-6.13 (m, 1H, =CH), 7.09 (d, J = 2.4 Hz, 1H, Ar), 7.23 (d, J = 2.4 Hz, 1H, Ar). ^{13}C NMR ($CDCl_3$, 100 MHz, TMS) δ 7.3, 14.1, 22.4, 30.3, 30.8, 31.5, 32.0, 34.4, 35.3, 72.5, 77.1, 108.5, 115.7, 123.0, 125.9, 132.5, 134.7, 142.0, 145.0, 153.0, 188.8. IR (neat) ν 2961, 2924, 2871, 2023, 1644, 1465, 1434, 1361, 1227, 1203, 1100, 984, 905, 878, 866 cm^{-1}. MS (%) m/e 367 (M^++1, 0.23), 326 (16.58), 325 (64.71), 284 (9.49), 283 (49.45), 213 (8.28), 120 (15.85), 57 (100.00), 41 (19.17). HRMS (EI) calcd. for $C_{26}H_{38}O$: 366.2923, Found: 366.2919.

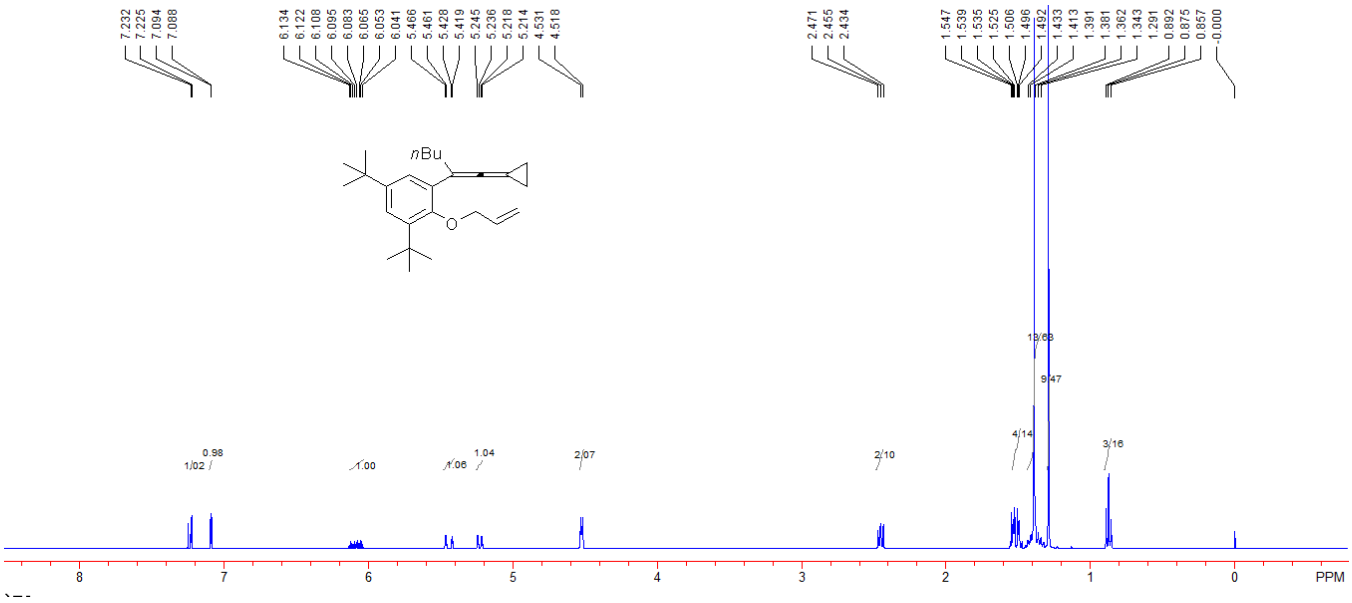

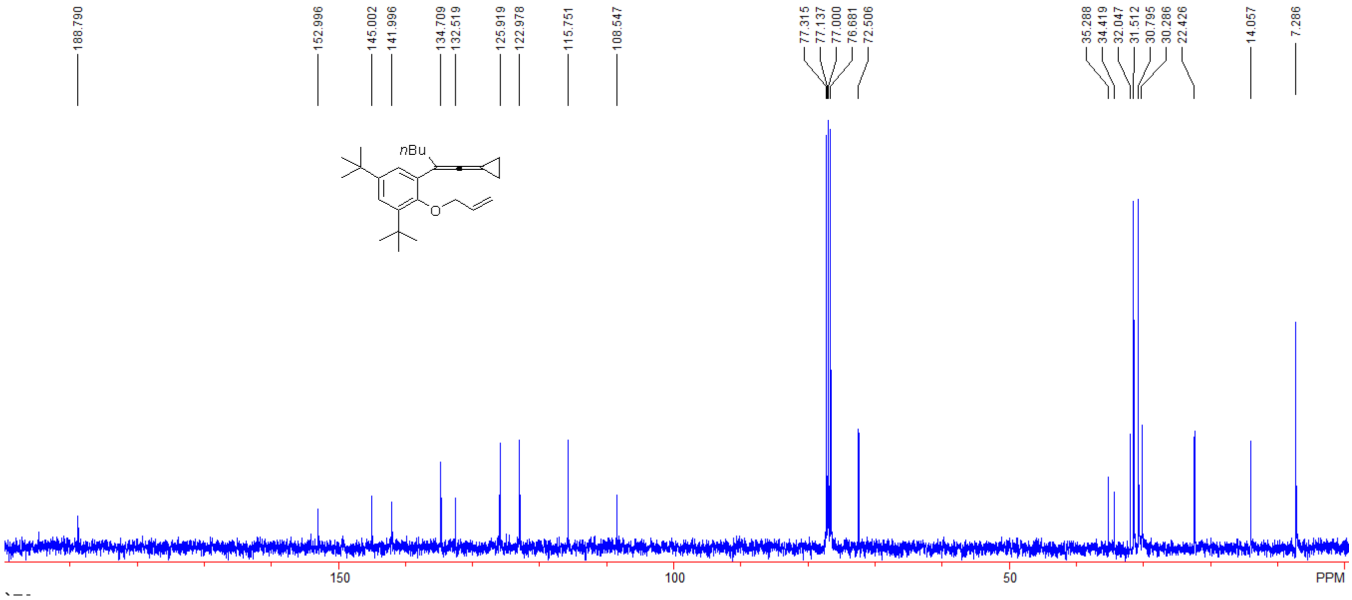

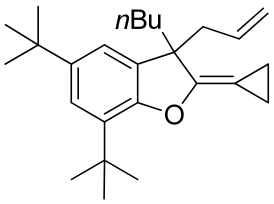

Compound **4d**. 150 mg, yield: 82%; colorless oil. ^{1}H NMR ($CDCl_3$, 400 MHz, TMS) δ 0.81 (t, *J* = 7.2 Hz, 3H, CH_3), 1.19-1.26 (m, 4H, CH_2), 1.28 (s, 9H, ^{t}Bu), 1.39 (s, 9H, ^{t}Bu), 1.62-1.65 (m, 2H, CH_2), 2.34-2.47 (m, 4H, 2CH_2), 2.86-2.87 (m, 2H, CH_2), 4.87-4.92 (m, 2H, =CH_2), 5.58-5.69 (m, 1H, =CH), 7.04 (d, *J* = 2.4 Hz, 1H, Ar), 7.14 (d, *J* = 2.4 Hz, 1H, Ar). ^{13}C NMR ($CDCl_3$, 100 MHz, TMS) δ 14.0, 23.0, 24.0, 27.6, 30.1, 31.1, 31.5, 34.4, 35.2, 40.4, 41.9, 46.9, 114.1, 116.3, 121.3, 121.9, 127.1, 135.9, 136.3, 143.9, 144.0, 150.2. IR (neat) ν 2956, 2926, 2858, 1746, 1455, 1434, 1361, 1309, 1262, 1211, 1112, 993, 909, 879, 817 cm^{-1}. MS (%) m/e 366 (M^+, 0.17), 326 (25.73), 325 (100.00), 309 (10.99), 283 (10.11), 269 (9.40), 155 (9.10), 120 (8.43), 57 (24.71). HRMS (EI) calcd. for $C_{26}H_{38}O$: 366.2923, Found: 366.2924.

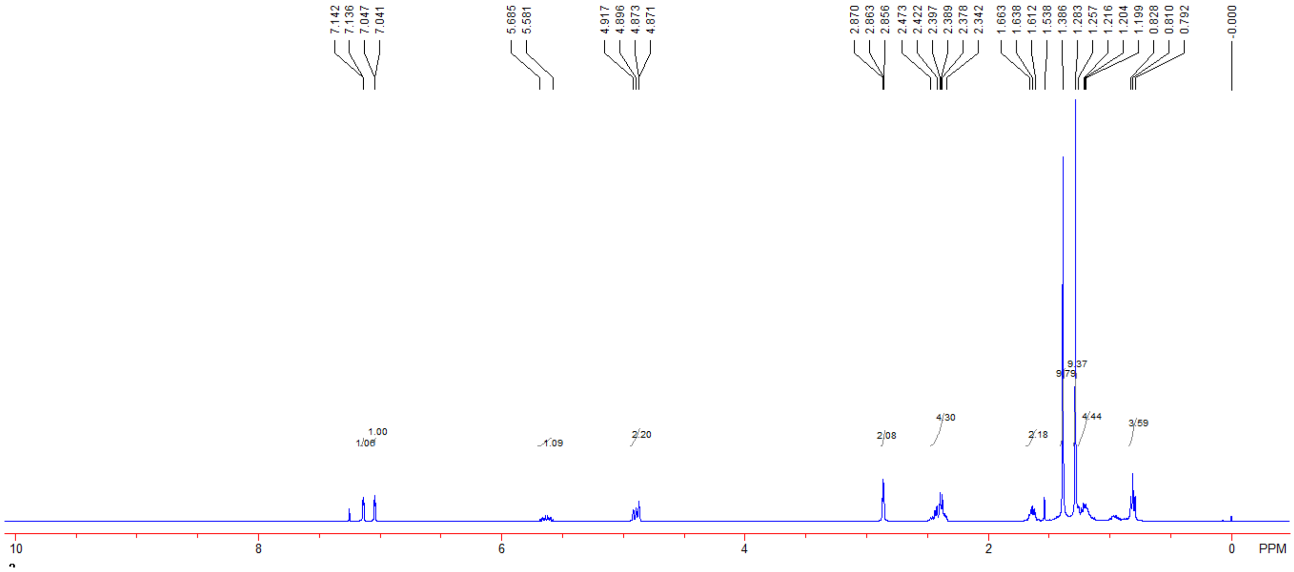

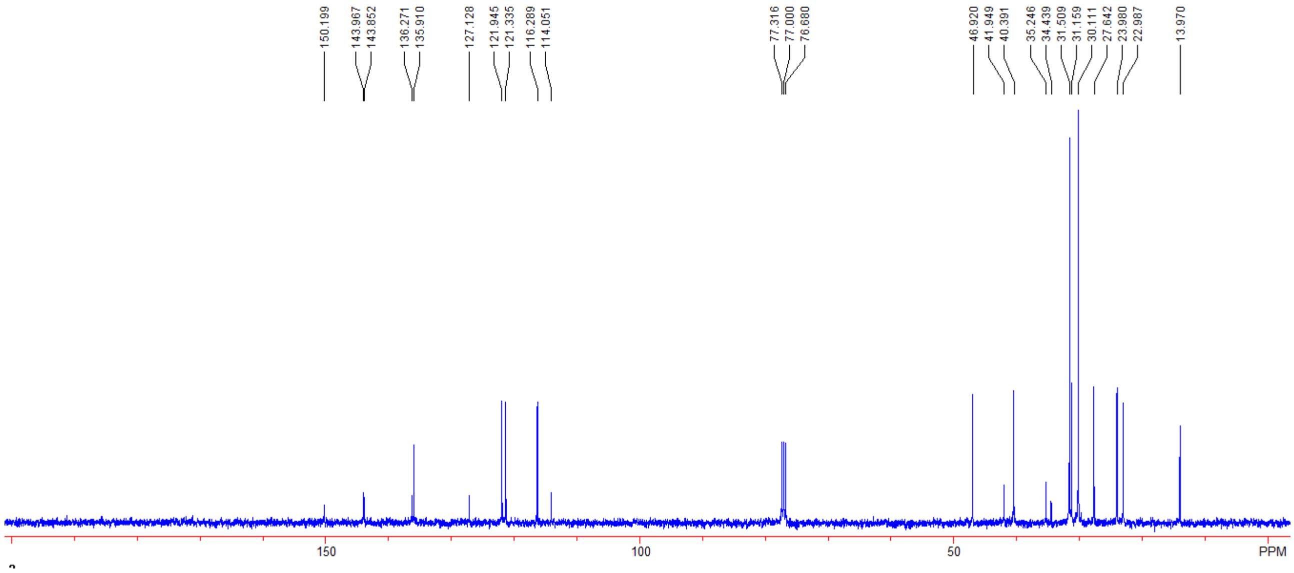

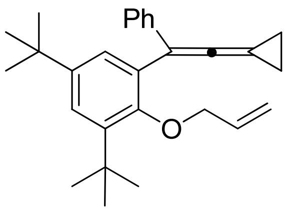

Compound **3e**. 604 mg, yield: 78%; white solid; MP: 119-120 °C. ^{1}H NMR ($CDCl_3$, 400 MHz, TMS) δ 1.29 (s, 9H, ^{t}Bu), 1.40 (s, 9H, ^{t}Bu), 1.64-1.67 (m, 2H, CH_2), 1.72-1.76 (m, 2H, CH_2), 4.44-4.46 (m, 2H, CH_2), 5.00-5.03 (m, 1H, =CH_2), 5.11-5.16 (m, 1H, =CH_2), 5.73-5.82 (m, 1H, =CH), 7.16-7.17 (m, 2H, Ar), 7.23-7.30 (m, 4H, Ar), 7.325-7.331 (m, 1H, Ar). ^{13}C NMR ($CDCl_3$, 100 MHz, TMS) δ 8.7, 30.8, 31.5, 34.5, 35.3, 72.6, 78.5, 109.2, 115.8, 123.5, 126.2, 126.9, 127.2, 128.0, 129.7, 134.4, 137.6, 142.1, 145.0, 153.8, 189.9. IR (neat) ν 2957, 2867, 1996, 1592, 1476, 1437, 1361, 1306, 1232, 1125, 1077, 988, 968, 915, 760, 691 cm^{-1}. MS (%) m/e 386 (M^+, 0.21), 345 (15.23), 234 (4.81), 233 (25.19), 215 (8.87), 115 (5.70), 71 (4.62), 57 (100.00), 41 (10.56). HRMS (EI) calcd. for $C_{28}H_{34}O$: 386.2610, Found: 386.2613.

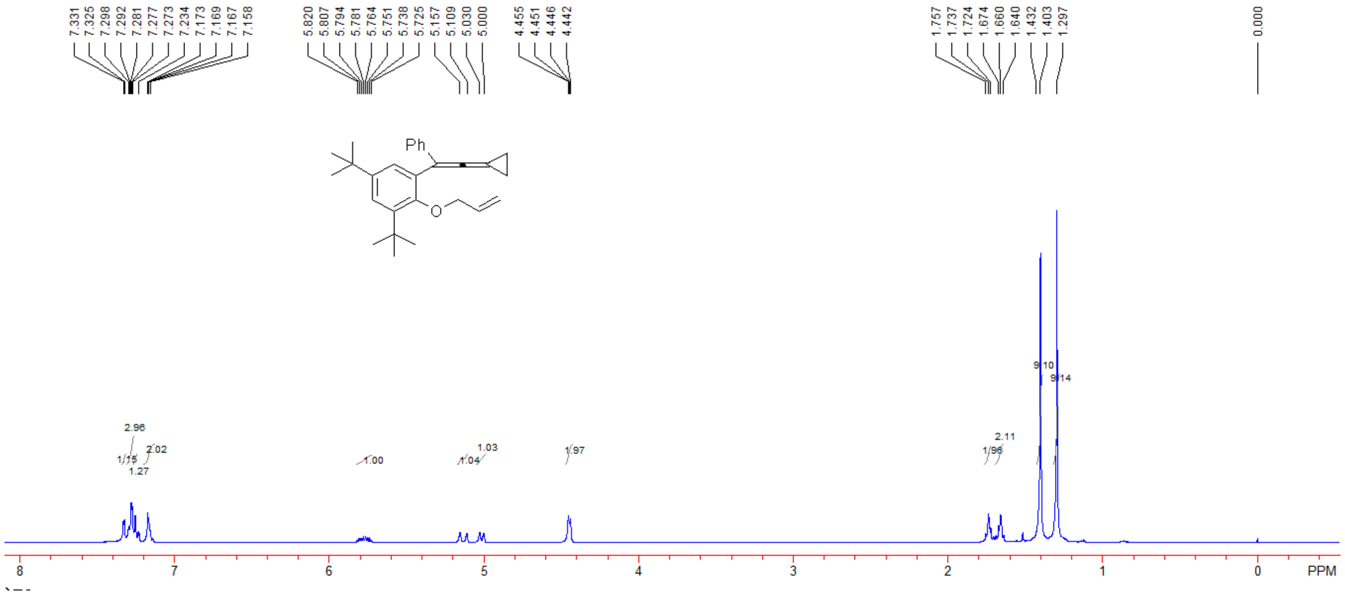

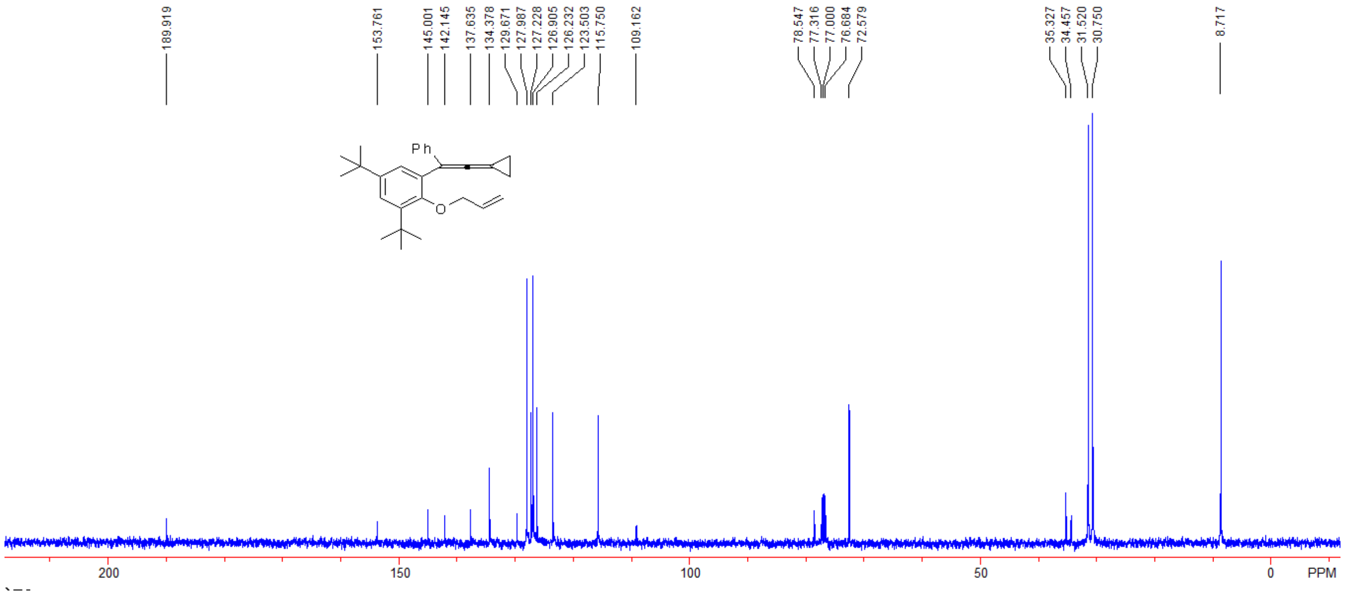

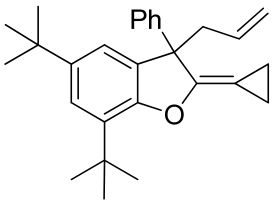

Compound **4e**. 123 mg, yield: 64%; colorless oil. ^{1}H NMR ($CDCl_3$, 400 MHz, TMS) δ 1.17 (s, 9H, ^{t}Bu), 1.42 (s, 9H, ^{t}Bu), 2.25-2.28 (m, 1H, CH_2), 2.37-2.41 (m, 1H, CH_2), 2.88-2.92 (m, 2H, CH_2), 4.92-4.50 (m, 2H, $=CH_2$), 5.59-5.70 (m, 1H, =CH), 6.88-6.89 (m, 1H, Ar), 7.14-7.17 (m, 2H, Ar), 7.27-7.31 (m, 2H, Ar), 7.34-7.36 (m, 2H, Ar). ^{13}C NMR ($CDCl_3$, 100 MHz, TMS) δ 23.8, 30.1, 31.2, 31.4, 34.4, 35.4, 44.0, 46.0, 114.1, 116.9, 121.7, 124.7, 125.7, 127.2, 127.6, 128.1, 135.6, 136.5,

143.4, 144.1, 147.2, 149.3. IR (neat) ν 2957, 2930, 2866, 1742, 1433, 1391, 1344, 1298, 1282, 1211, 1124, 1092, 909, 817, 698 cm^{-1}. MS (%) m/e 385 (M^+-1, 0.56), 345 (73.55), 325 (84.09), 205 (48.60), 86 (41.60), 84 (69.02), 57 (100.00), 43 (22.76), 41 (31.11). HRMS (EI) calcd. for $C_{28}H_{34}O$: 386.2610, Found: 386.2614; Enantiomeric excess was determined by HPLC with a Chiralcel PC-3 column [λ = 254 nm; eluent: Acetonitrile/water = 7/3; Flow rate: 0.7 mL/min; t_{minor} = 12.08 min, t_{major} = 10.15 min; ee% = 10%; $[\alpha]^{20}_D$ = 0.0753 (c 0.85, CH_2Cl_2)].

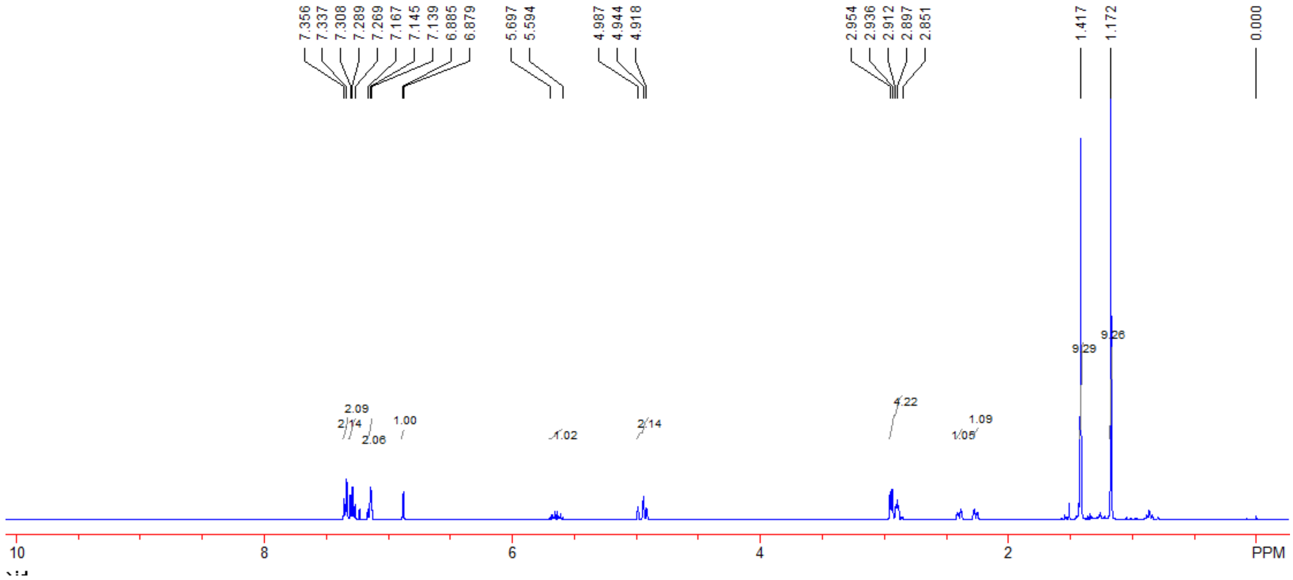

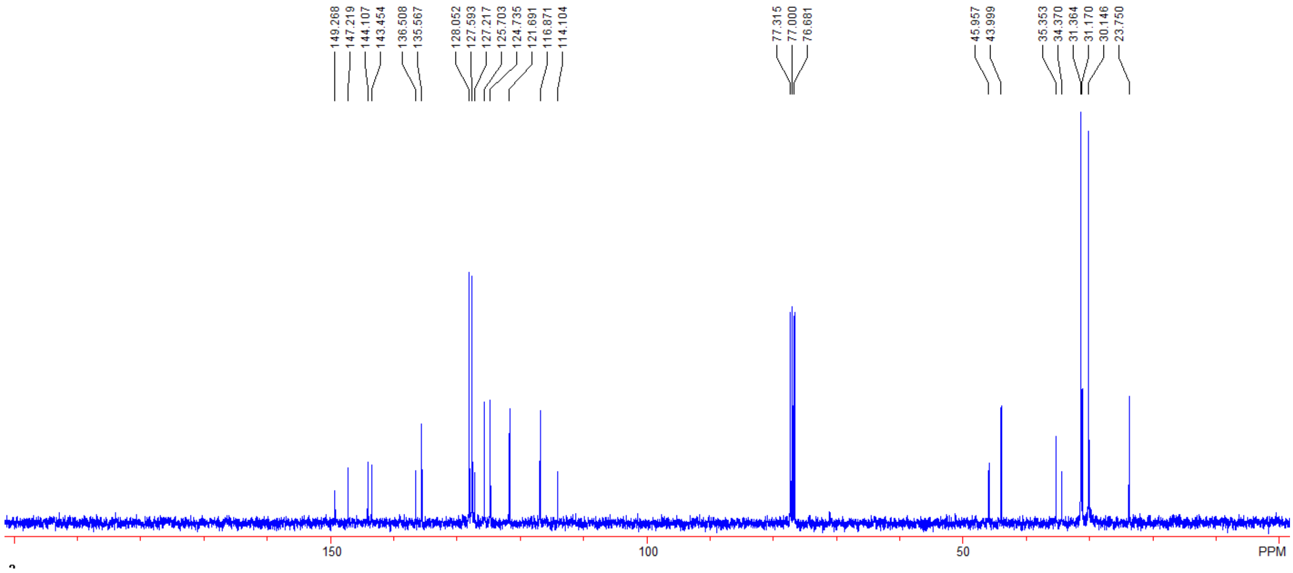

Additional Info : Peak(s) Manually Integrated

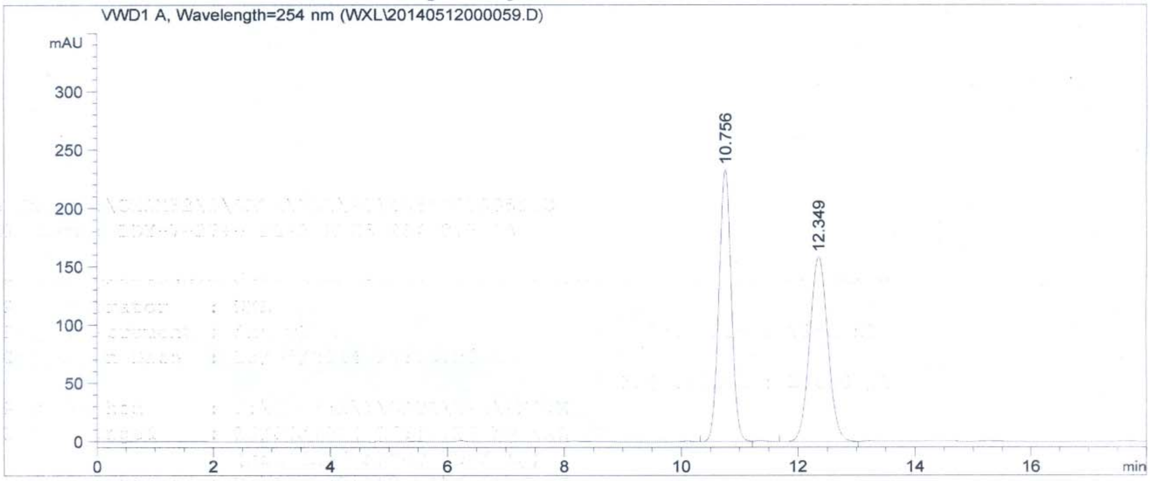

===

Area Percent Report

===

Sorted By : Signal
Multiplier: : 1.0000
Dilution: : 1.0000
Use Multiplier & Dilution Factor with ISTDs

Signal 1: VWD1 A, Wavelength=254 nm

Peak #	RetTime [min]	Type	Width [min]	Area [mAU*s]	Height [mAU]	Area %
1	10.756	BV	0.2278	3436.82349	233.37276	50.0329
2	12.349	BV	0.3365	3432.30005	158.58882	49.9671

Totals : 6869.12354 391.96158

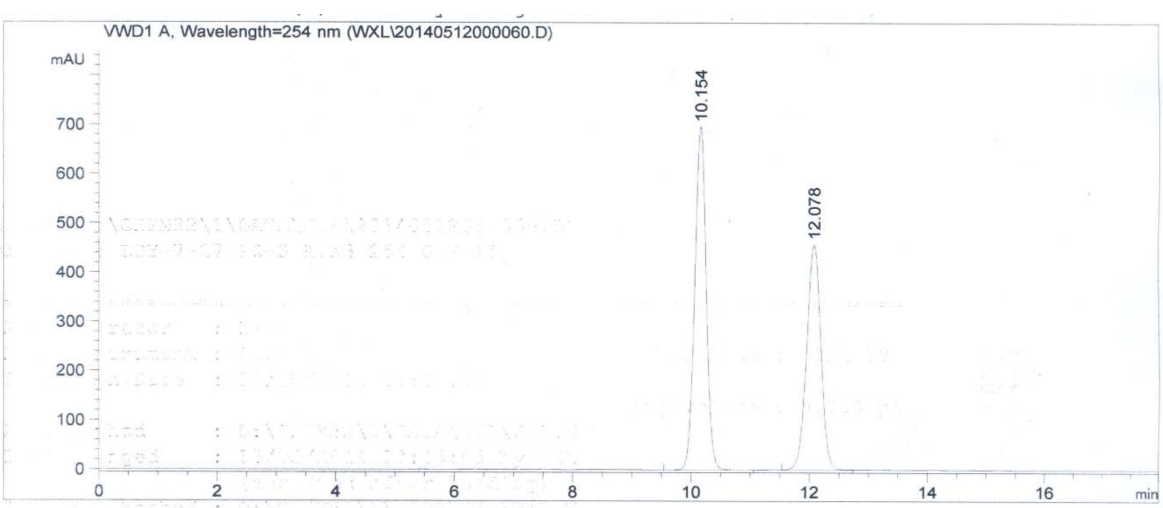


```
=====================================================================
                       Area Percent Report
=====================================================================

Sorted By             :      Signal
Multiplier:                  :      1.0000
Dilution:                    :      1.0000
Use Multiplier & Dilution Factor with ISTDs


Signal 1: VWD1 A, Wavelength=254 nm

Peak RetTime Type  Width     Area      Height     Area
  #   [min]        [min]   [mAU*s]     [mAU]        %
----|-------|----|-------|----------|----------|--------|
   1  10.154 VB    0.2074 9344.22949  696.85278  54.8849
   2  12.078 VV    0.2595 7680.89844  458.32516  45.1151

Totals :                  1.70251e4  1155.17795
```

Translation: a Chiralcel PC-3 column [λ = 254 nm; eluent: Acetonitrile/water = 7/3; Flow rate: 0.7 mL/min; t_{minor} = 12.08 min, t_{major} = 10.15 min; ee% = 10%.]

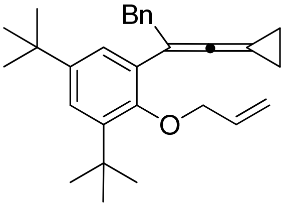

Compound **3f**. 732 mg, yield: 61%; colorless oil. ^{1}H NMR (CDCl$_3$, 400 MHz, TMS) δ 1.24 (s, 9H, ^{t}Bu), 1.34-1.37 (m, 2H, CH$_2$), 1.39 (s, 9H, ^{t}Bu), 1.43-1.48 (m, 2H, CH$_2$), 3.79 (s, 2H, CH$_2$), 4.55-4.57 (m, 2H, CH$_2$), 5.25-5.28 (m, 1H, =CH$_2$), 5.47-5.53 (m, 1H, =CH$_2$), 6.09-6.16 (m, 1H, =CH), 7.016-7.022 (m, 1H, Ar), 7.08-7.12 (m, 1H, Ar), 7.15-7.21 (m, 4H, Ar), 7.26-7.27 (m, 1H, Ar). ^{13}C NMR (CDCl$_3$, 100 MHz, TMS) δ 7.4, 30.8, 31.4, 34.4, 35.3, 39.4, 72.6, 77.6, 108.0, 115.8, 123.1, 125.7, 126.2, 127.8, 129.2, 131.8, 134.6, 140.1, 142.0, 145.1, 153.1, 189.8. IR (neat) ν 3027, 2958, 2901, 2019, 1645, 1465, 1435, 1360, 1230, 1129, 1060, 977, 916, 748, 697 cm^{-1}. MS (%) m/e

400 (M+, 0.36), 360 (28.80), 359 (95.99), 243 (9.54), 165 (9.00), 129 (22.05), 91 (24.95), 57 (100.00), 41 (15.53). HRMS (EI) calcd. for $C_{29}H_{36}O$: 400.2766, Found: 400.2763.

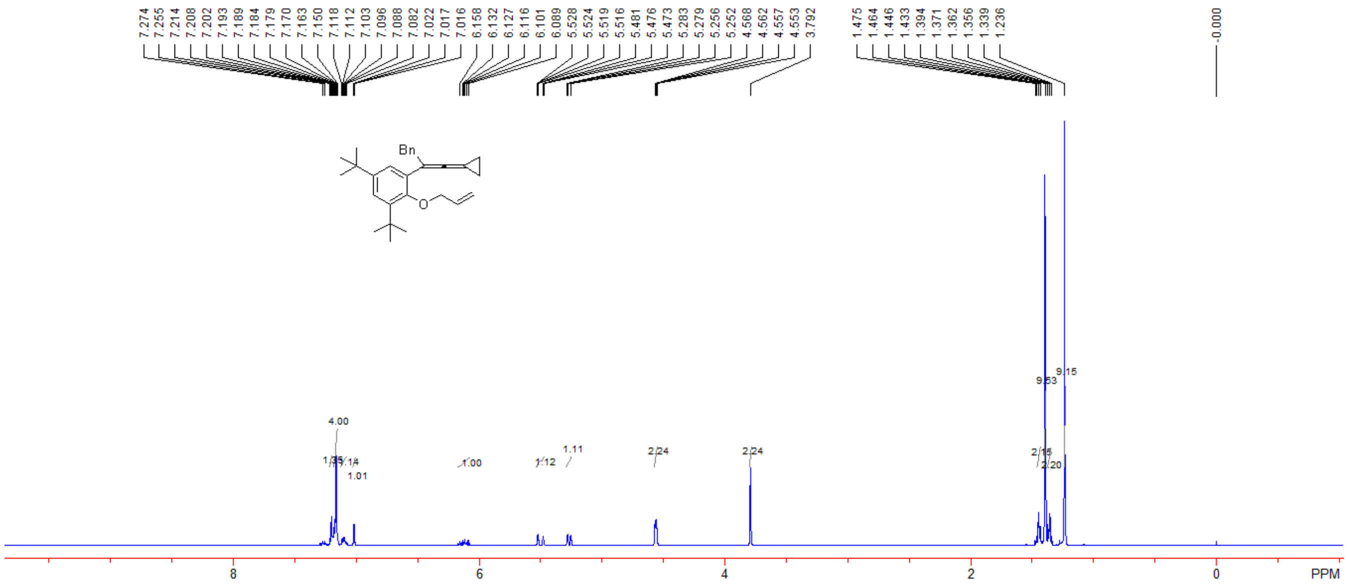

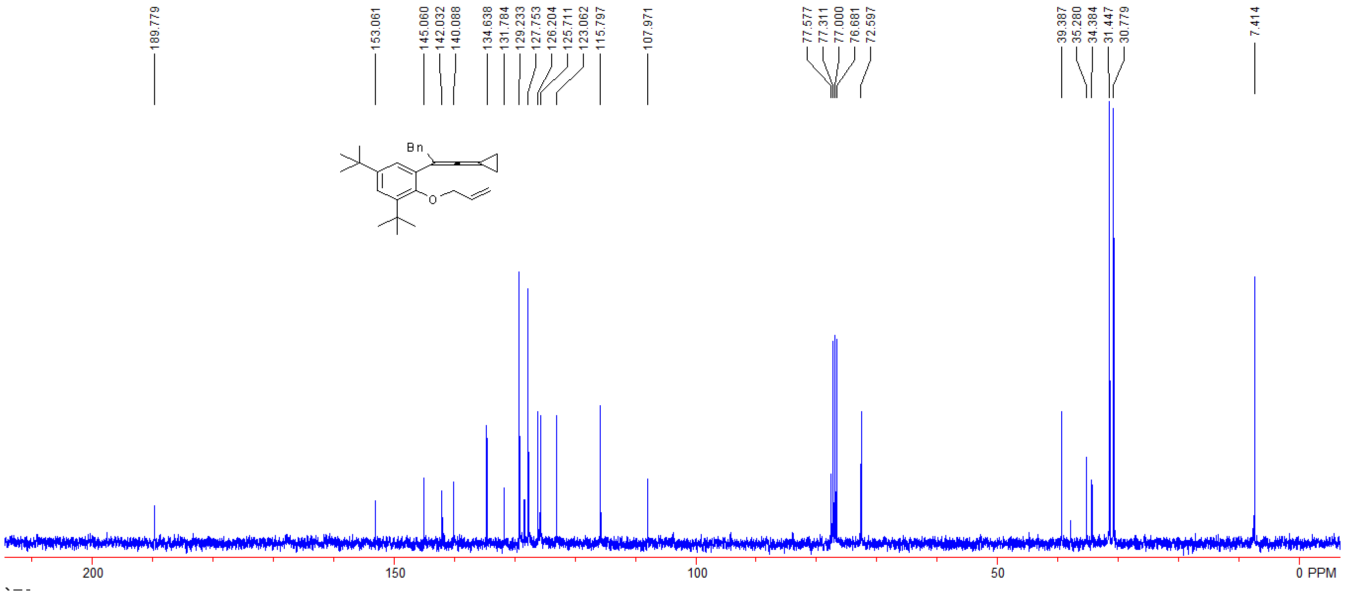

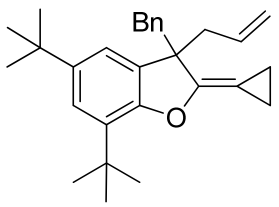

Compound **4f**. 162 mg, yield: 81%; colorless oil. ^{1}H NMR ($CDCl_3$, 400 MHz, TMS) δ 1.23 (s, 9H, ^{t}Bu), 1.32 (s, 9H, ^{t}Bu), 2.41-2.42 (m, 2H, CH_2), 2.56-2.61 (m, 1H, CH_2), 2.69-2.75 (m, 3H, CH_2), 2.86-2.87 (m, 2H, CH_2), 4.94-5.04 (m, 2H, $=CH_2$), 5.69-5.79 (m, 1H, =CH), 6.64-6.66 (m, 2H, Ar),

7.02-7.04 (m, 3H, Ar), 7.11 (d, *J* = 2.4 Hz, H, Ar), 7.15 (d, *J* = 2.4 Hz, H, Ar). ^{13}C NMR ($CDCl_3$, 100 MHz, TMS) δ 24.3, 29.9, 30.9, 31.5, 34.5, 35.1, 43.6, 44.8, 48.7, 112.9, 116.7, 121.4, 122.4, 125.6, 125.9, 127.2, 130.0, 135.8, 136.4, 138.7, 143.9, 144.4, 150.2. IR (neat) ν 3028, 2957, 2927, 1745, 1603, 1476, 1434, 1362, 1305, 1211, 1095, 1003, 910, 754, 699 cm^{-1}. MS (%) m/e 400 (M^+, 0.17), 345 (13.64), 309 (11.94), 91 (28.65), 86 (62.17), 84 (100.00), 57 (19.87), 49 (11.93), 47 (14.52). HRMS (EI) calcd. for $C_{29}H_{36}O$: 400.2766, Found: 400.2765.

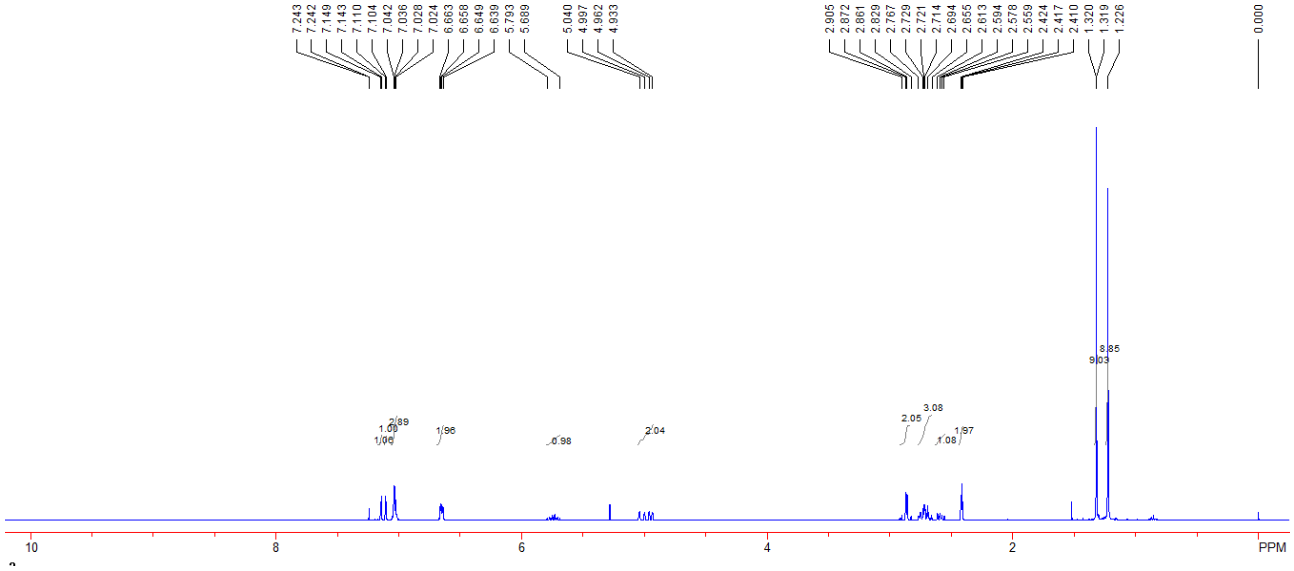

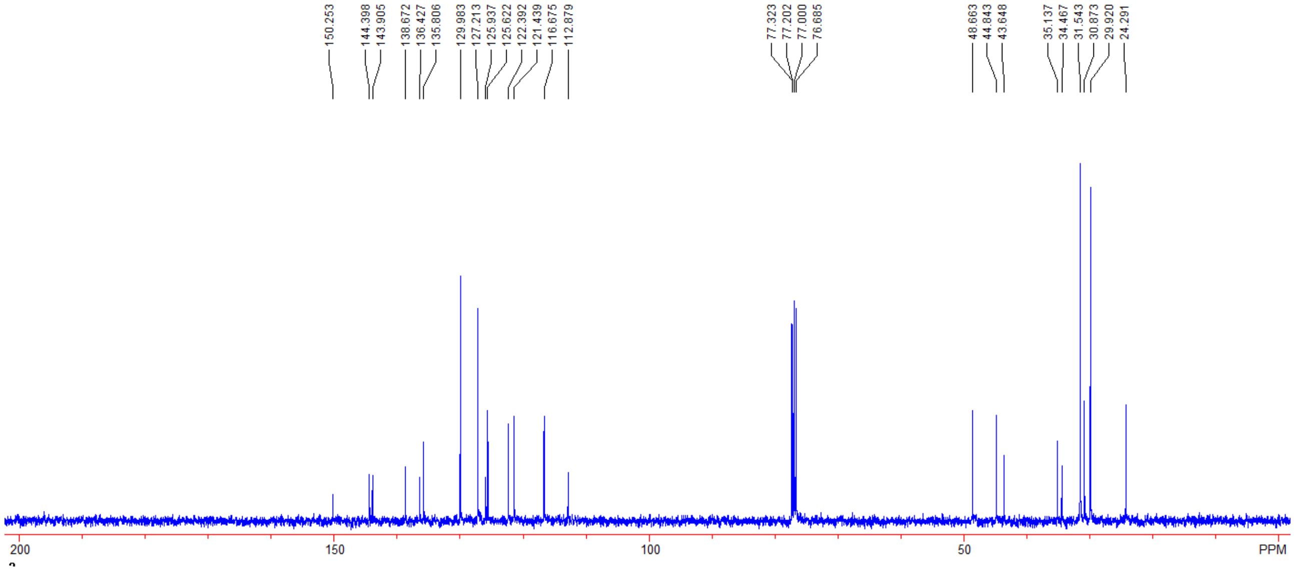

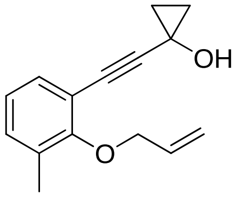

Compound **S3s**. 4.133 g, yield: 86%; yellow oil (viscous oil contains little DCM). ^{1}H NMR ($CDCl_3$,

400 MHz, TMS) δ 1.05-1.09 (m, 2H, CH_2), 1.15-1.18 (m, 2H, CH_2), 2.25 (s, 3H, CH_3), 3.30 (br, 1H, OH), 4.54-4.55 (m, 2H, CH_2), 5.23-5.25 (m, 1H, $=CH_2$), 5.39-5.43 (m, 1H, $=CH_2$), 6.06-6.14 (m, 1H, =CH), 6.90-6.94 (m, 1H, Ar), 7.09-7.11 (m, 1H, Ar), 7.20-7.22 (m, 1H, Ar). ^{13}C NMR ($CDCl_3$, 100 MHz, TMS) δ 16.2, 17.5, 45.9, 73.8, 78.8, 95.1, 116.6, 117.4, 123.5, 130.9, 131.1, 131.4, 134.0, 158.0. IR (neat) ν 3360, 3012, 2854, 2246, 1461, 1418, 1374, 1257, 1206, 1085, 983, 908, 871, 779, 728 cm^{-1}. MS (%) m/e 228 (M^+, 2.64), 171 (53.51), 145 (83.75), 129 (57.77), 128 (58.03), 115 (100.00), 91 (53.24), 77 (59.58), 55 (71.99). HRMS (EI) calcd. for $C_{15}H_{15}O_2[M-H]^+$: 227.1072, Found: 227.1071.

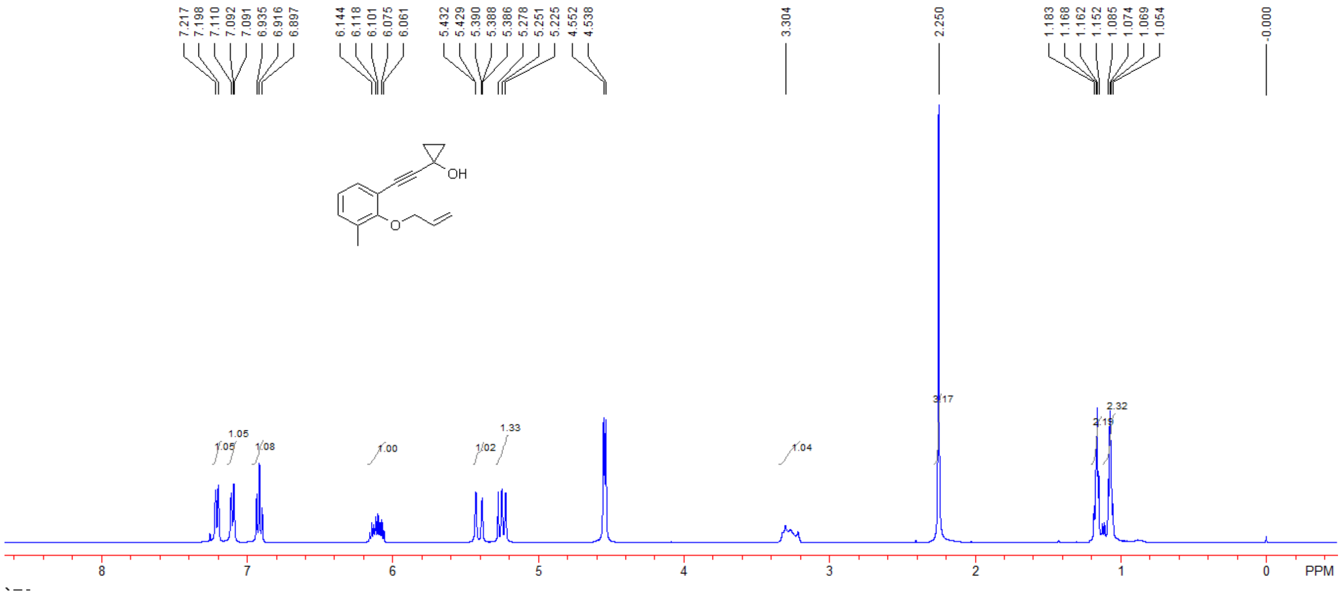

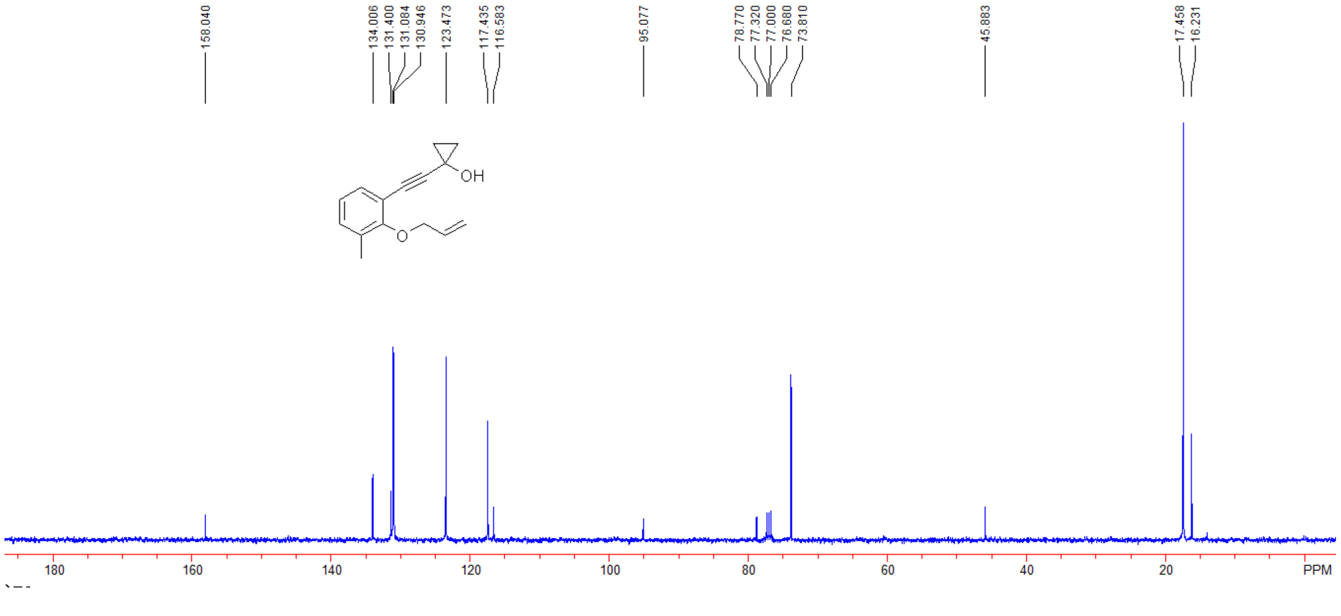

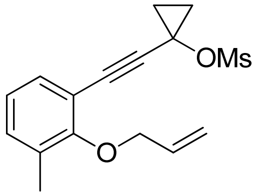

Compound **S4s**. 4.896 g, yield: 89%; yellow oil. ^{1}H NMR ($CDCl_3$, 400 MHz, TMS) δ 1.33-1.37 (m, 2H, CH_2), 1.63-1.67 (m, 2H, CH_2), 2.27 (s, 3H, CH_3), 3.26 (s, 3H, CH_3), 4.53-4.55 (m, 2H, CH_2), 5.26-5.29 (m, 1H, =CH_2), 5.41-5.46 (m, 1H, =CH_2), 6.08-6.15 (m, 1H, =CH), 6.95-6.98 (m, 1H, Ar), 7.17-7.19 (m, 1H, Ar), 7.22-7.24 (m, 1H, Ar). ^{13}C NMR ($CDCl_3$, 100 MHz, TMS) δ 16.1, 16.8, 39.8, 54.7, 74.0, 83.0, 89.4, 115.4, 117.7, 123.7, 131.0, 131.7, 132.2, 133.7, 158.6. IR (neat) ν 3016, 2931, 1862, 2255, 1461, 1362, 1204, 1160, 1085, 976, 937, 907, 885, 809, 728 cm^{-1}. MS (%) m/e 306 (M^+, 1.88), 185 (51.08), 142 (69.19), 141 (91.96), 129 (82.85), 128 (93.56), 115 (100.00), 102 (66.49), 55 (55.16). HRMS (EI) calcd. for $C_{16}H_{18}O_4S$: 306.0926, Found: 306.0927.

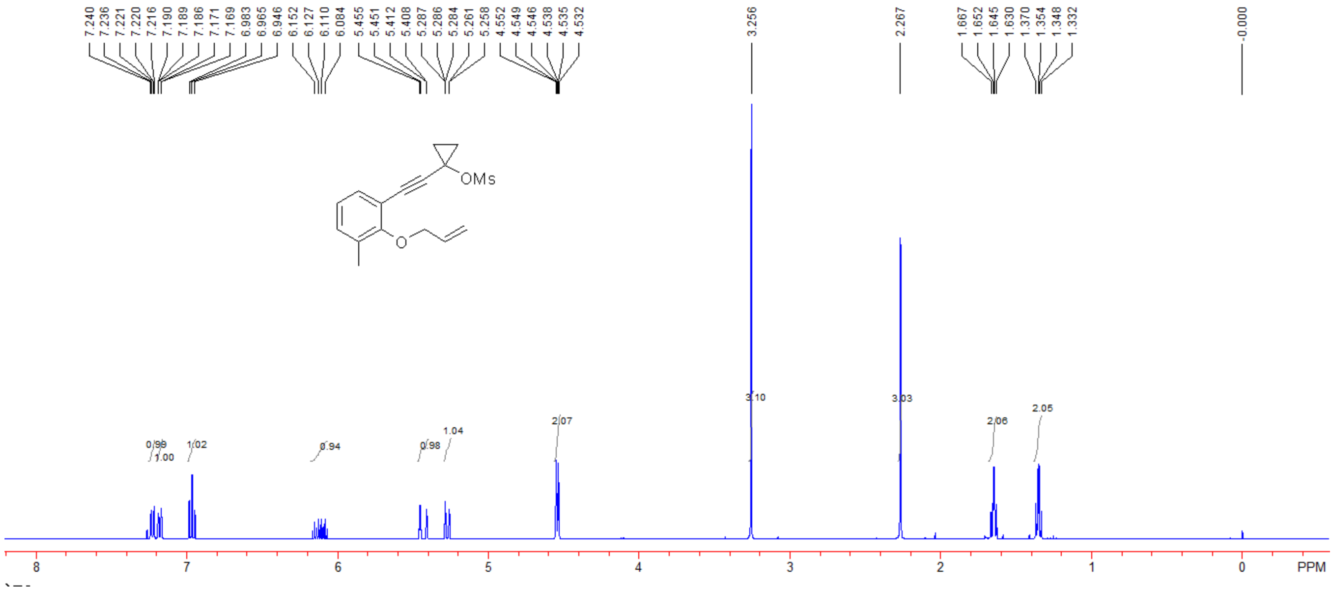

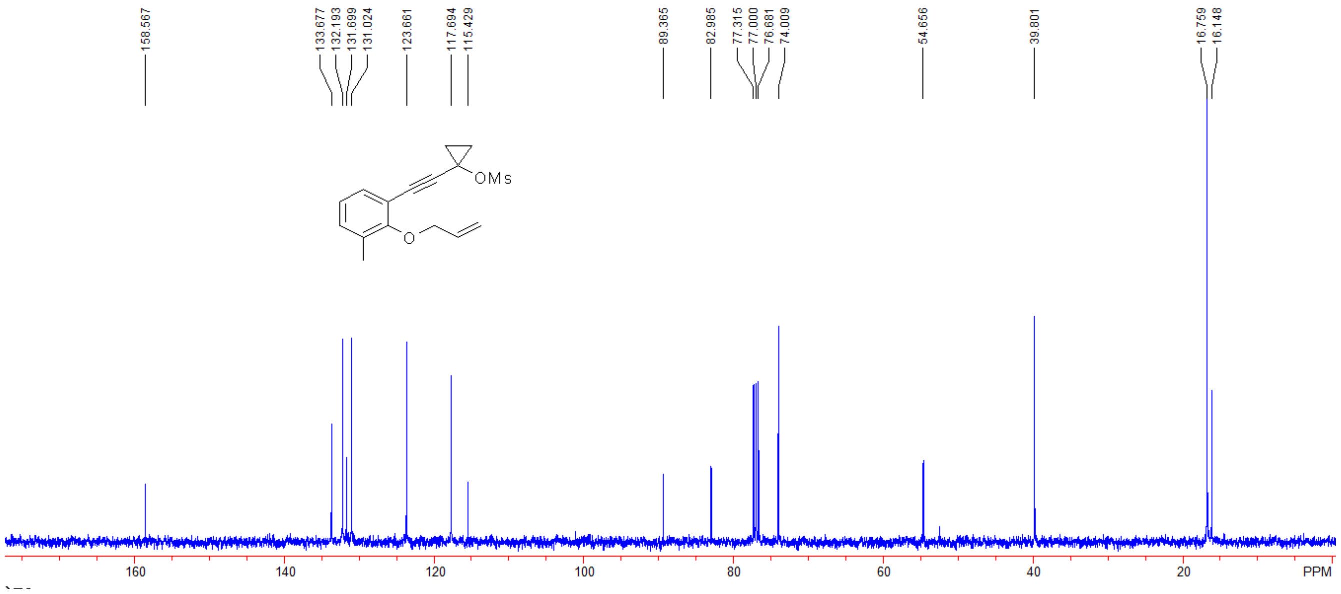

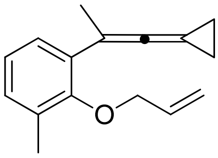

Compound **3g**. 522 mg, yield: 77%; colorless oil. ^{1}H NMR ($CDCl_3$, 400 MHz, TMS) δ 1.54-1.56 (m, 4H, $2CH_2$), 2.18 (s, 3H, CH_3), 2.29 (s, 3H, CH_3), 4.37-4.39 (m, 2H, CH_2), 5.21-5.24 (m, 1H, $=CH_2$), 5.38-5.42 (m, 1H, $=CH_2$), 6.04-6.14 (m, 1H, =CH), 6.97-7.01 (m, 1H, Ar), 7.06-7.08 (m, 1H, Ar), 7.13-7.14 (m, 1H, Ar). ^{13}C NMR ($CDCl_3$, 100 MHz, TMS) δ 7.5, 16.4, 20.2, 73.0, 75.5, 100.3, 116.7, 123.7, 126.8, 129.6, 131.8, 132.9, 134.4, 154.7, 190.3. IR (neat) ν 2981, 2915, 2857, 2008, 1646, 1442, 1418, 1254, 1206, 1104, 1064, 986, 922, 864, 780, 756 cm^{-1}. MS (%) m/e 226 (M^+, 0.80), 186 (16.14), 185 (100.00), 170 (19.92), 169 (14.69), 142 (42.47), 141 (28.96), 128 (16.82), 115 (23.40). HRMS (EI) calcd. for $C_{16}H_{18}O$: 226.1358, Found: 226.1354.

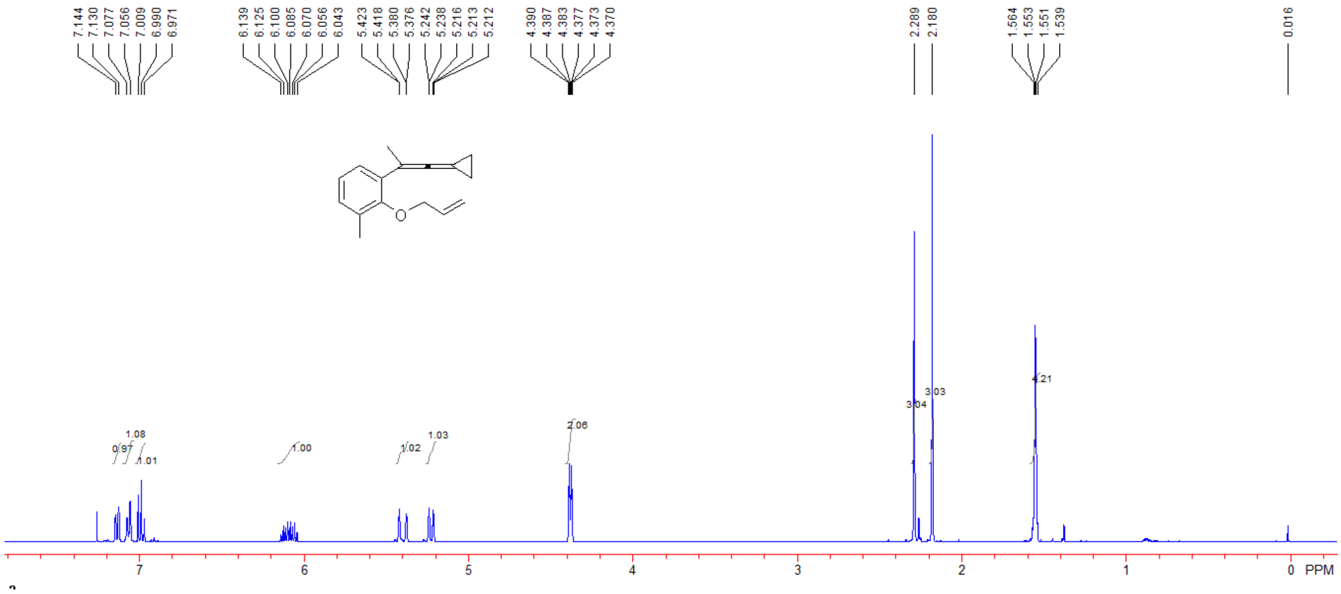

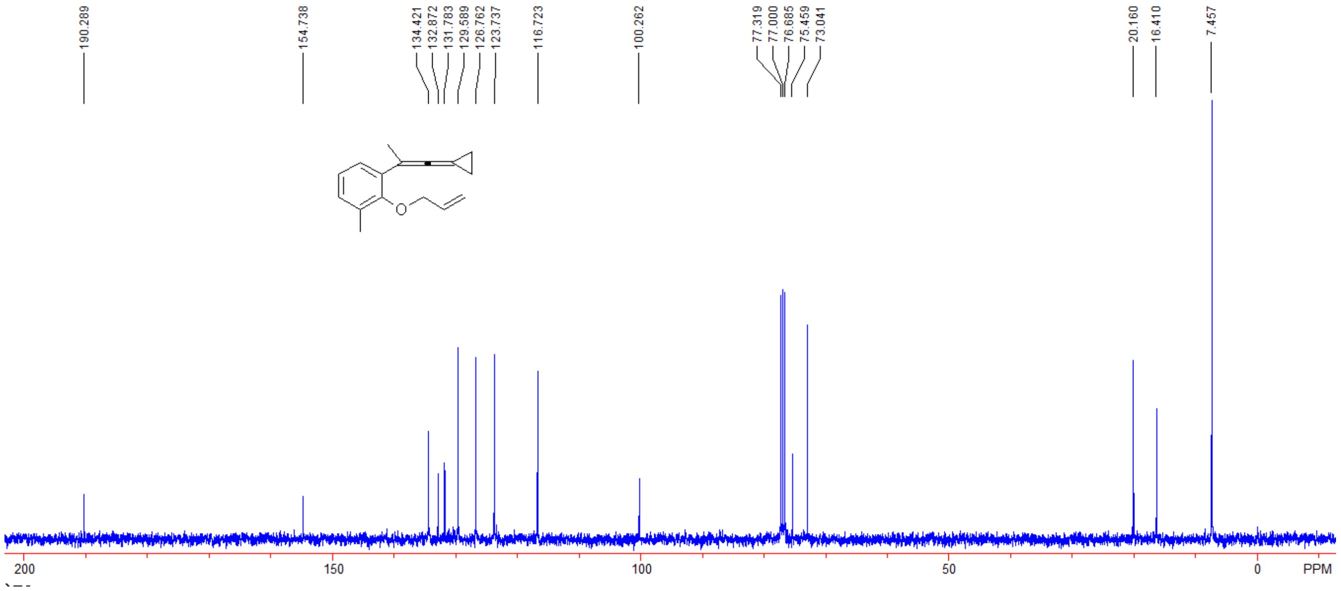

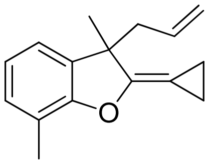

Compound **4g**. 166 mg, yield: 85%; colorless oil. ^{1}H NMR ($CDCl_3$, 400 MHz, TMS) δ 1.38 (s, 3H, CH_3), 2.23 (s, 3H, CH_3), 2.34-2.49 (m, 4H, $2CH_2$), 2.82-2.90 (m, 2H, CH_2), 4.90-4.96 (m, 2H, $=CH_2$), 5.57-5.65 (m, 1H, =CH), 6.91-6.94 (m, 1H, Ar), 6.97-6.99 (m, 1H, Ar), 7.08-7.10 (m, 1H, Ar). ^{13}C NMR ($CDCl_3$, 100 MHz, TMS) δ 16.5, 23.4, 28.6, 31.1, 37.1, 47.1, 116.6, 116.7, 122.5, 125.1, 126.1, 128.5, 129.4, 135.8, 142.6, 150.8. IR (neat) ν 2957, 2925, 2852, 1743, 1639, 1459, 1353, 1296, 1195, 1168, 1095, 1022, 912, 824, 741 cm^{-1}. MS (%) m/e 226 (M^+, 0.34), 186 (16.04),

185 (100.00), 170 (3.43), 152 (3.27), 142 (7.67), 141 (7.06), 128 (4.76), 115 (5.70). HRMS (EI) calcd. for $C_{16}H_{18}O$: 226.1358, Found: 226.1354.

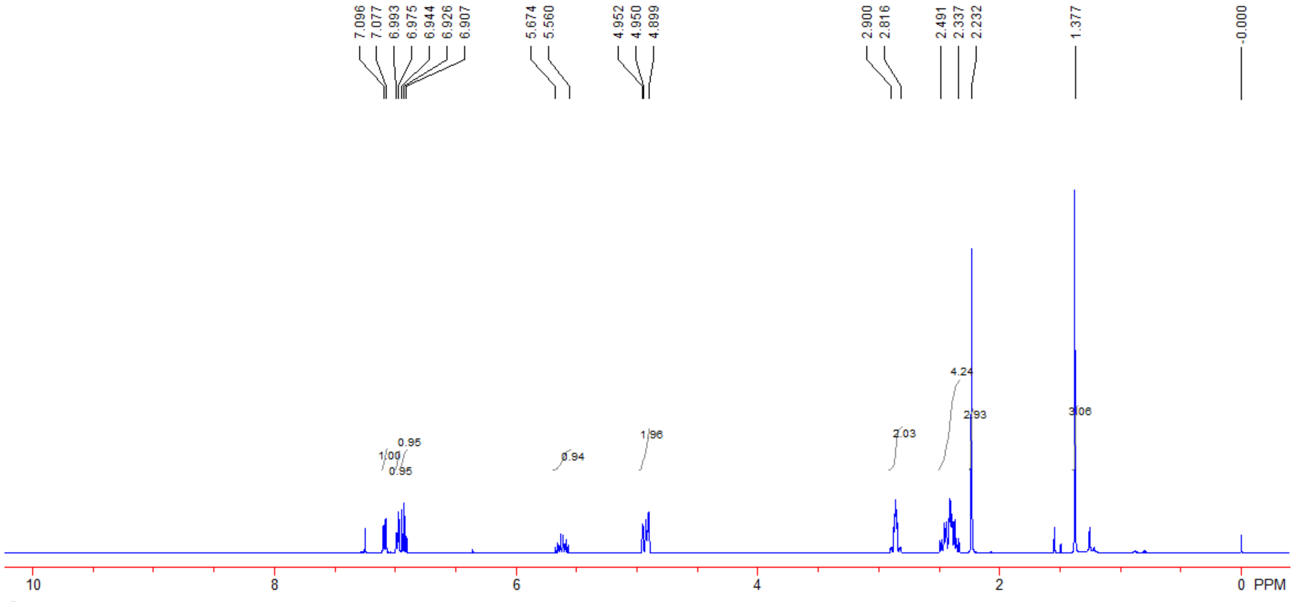

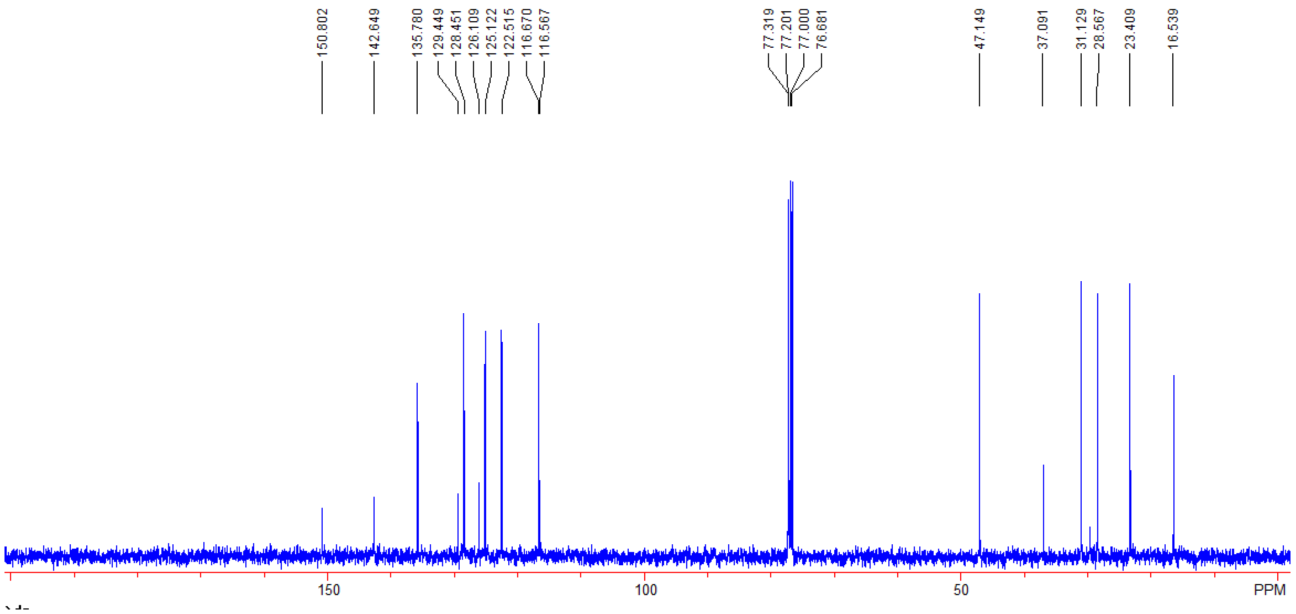

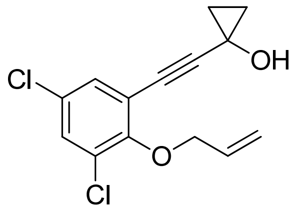

Compound **S3t**. 2.830 g, yield: 53%; yellow oil. ^{1}H NMR ($CDCl_3$, 400 MHz, TMS) δ 1.10-1.13 (m, 2H, CH_2), 1.21-1.23 (m, 2H, CH_2), 3.10 (br, 1H, OH), 4.59-4.62 (m, 2H, CH_2), 5.26-5.31 (m, 1H, $=CH_2$), 5.43-5.46 (m, 1H, $=CH_2$), 6.06-6.17 (m, 1H, =CH), 7.23-7.27 (m, 1H, Ar), 7.30-7.34 (m, 1H,

Ar). ^{13}C NMR ($CDCl_3$, 100 MHz, TMS) δ 17.7, 45.8, 74.6, 76.7, 97.5, 118.6, 120.1, 128.9, 129.1, 129.9, 131.1, 133.1, 154.3. IR (neat) ν 3307, 3064, 2927, 2226, 1577, 1551, 1444, 1401, 1238, 1206, 1174, 1028, 976, 855, 774 cm^{-1}. MS (%) m/e 282 (M^+, 0.97), 213 (36.57), 201 (33.32), 199 (50.72), 178 (34.36), 149 (39.85), 115 (48.59), 55 (100.00), 41 (53.14). HRMS (EI) calcd. for $C_{14}H_{12}O_2Cl_2$: 282.0214, Found: 282.0219.

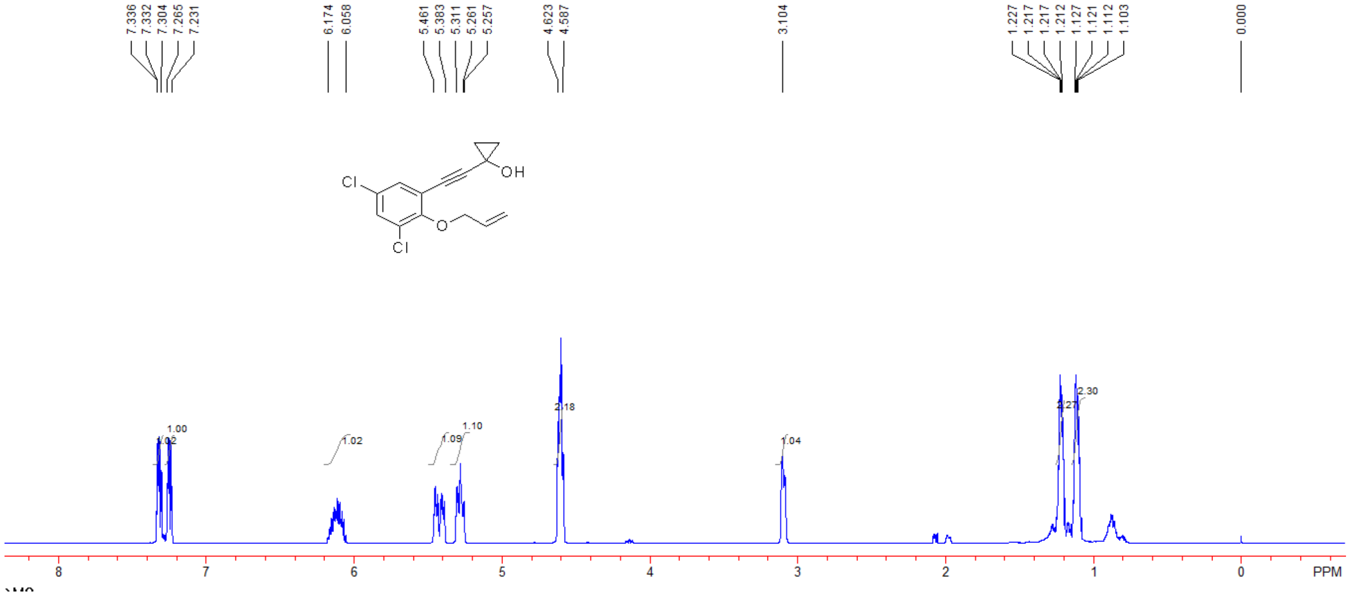

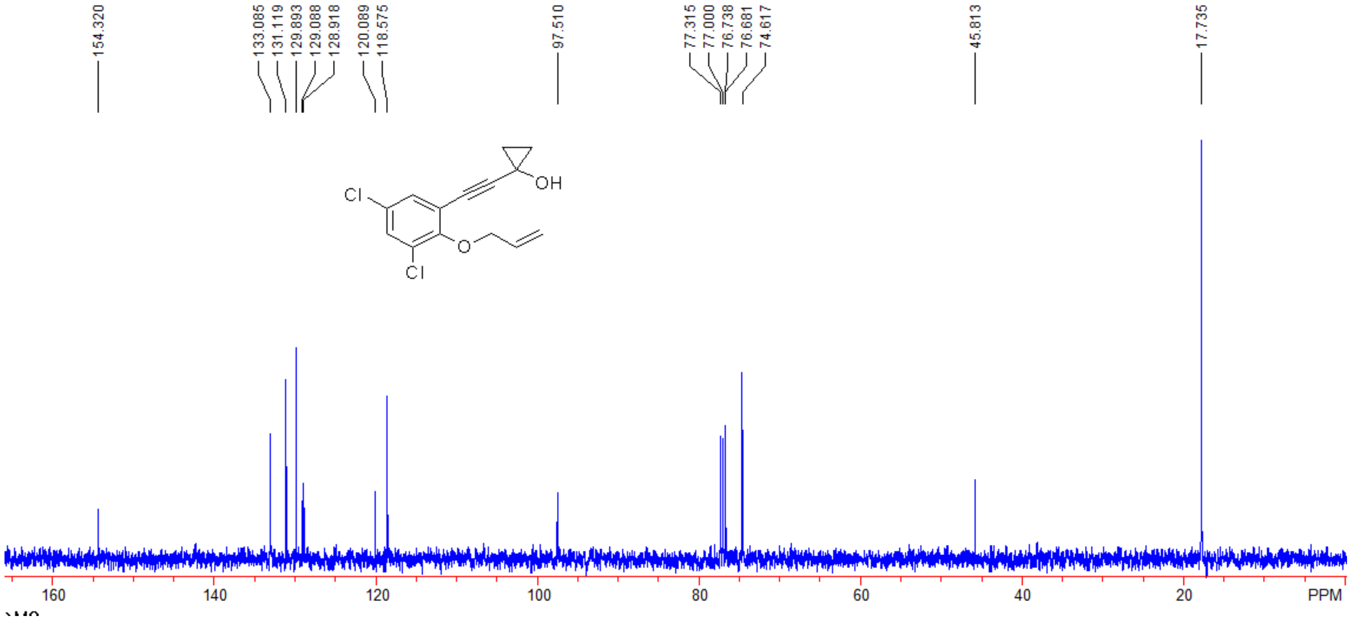

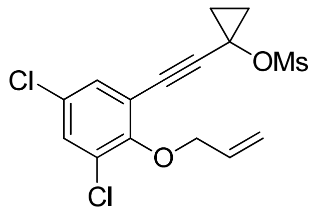

Compound **S4t**. 3.321 g, yield: 92%; yellow oil. ^{1}H NMR ($CDCl_3$, 400 MHz, TMS) δ 1.35-1.39 (m, 2H, CH_2), 1.66-1.70 (m, 2H, CH_2), 3.23 (s, 3H, CH_3), 4.60-4.62 (m, 2H, CH_2), 5.28-5.31 (m, 1H, $=CH_2$), 5.40-5.45 (m, 1H, $=CH_2$), 6.07-6.17 (m, 1H, =CH), 7.27 (d, J = 2.4 Hz, 1H, Ar), 7.37 (d, J = 2.4 Hz, 1H, Ar). ^{13}C NMR ($CDCl_3$, 100 MHz, TMS) δ 17.0, 39.9, 54.0, 74.9, 80.3, 91.9, 119.0, 119.1, 129.1, 129.3, 130.9, 131.1, 132.8, 154.8. IR (neat) ν 3068, 3020, 2939, 2230, 1551, 1444, 1362, 1220, 1160, 1037, 973, 938, 873, 801, 774 cm^{-1}. MS (%) m/e 360 (M^+, 2.08), 189 (84.63), 162 (68.59), 161 (55.13), 149 (54.07), 126 (59.15), 121 (55.37), 55 (70.09), 41 (100.00). HRMS (EI) calcd. for $C_{15}H_{14}O_4Cl_2S$: 359.9990, Found: 359.9992.

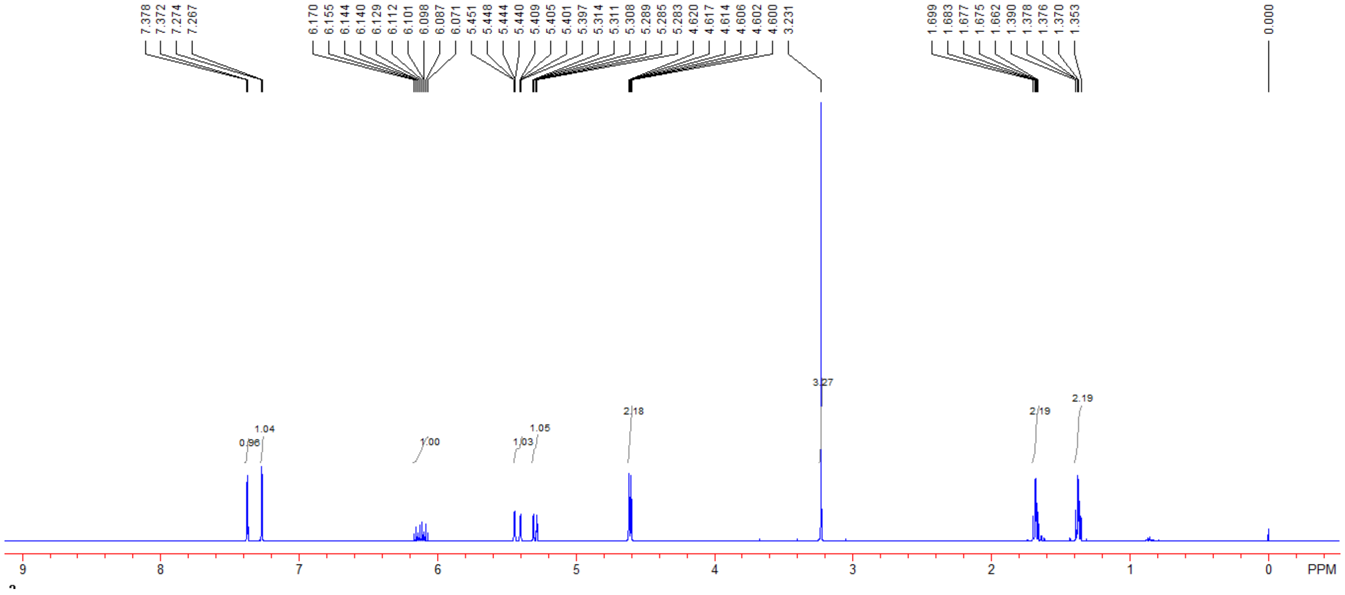

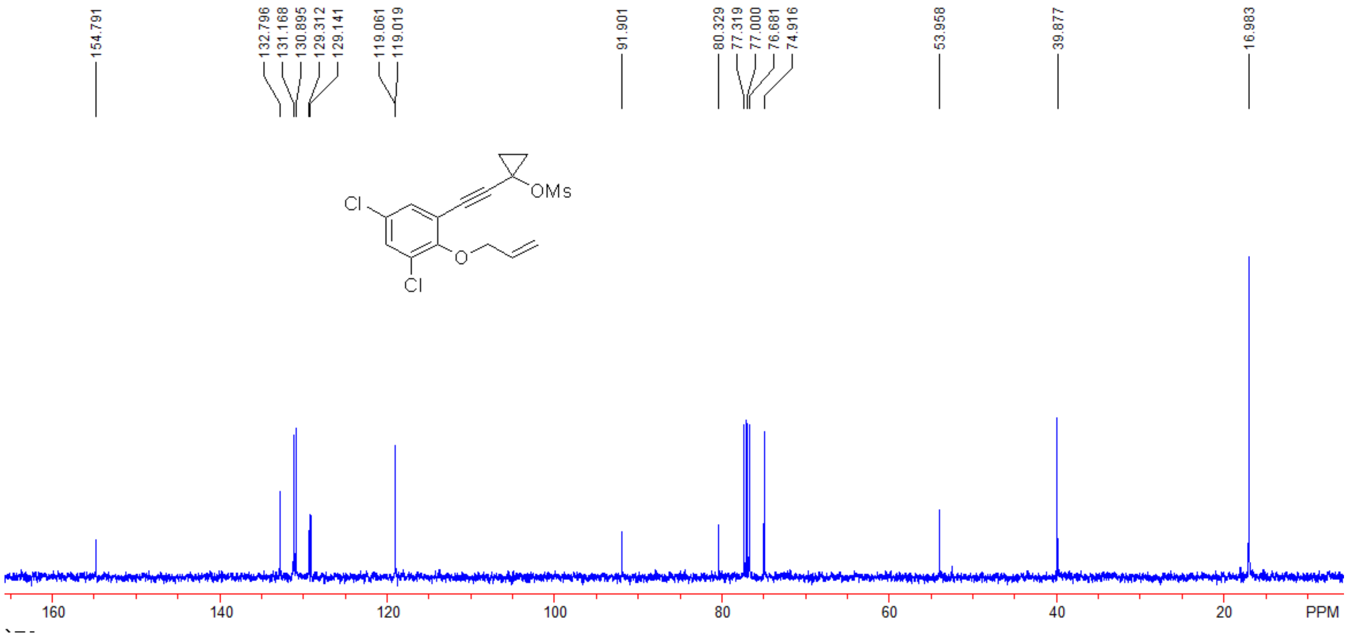

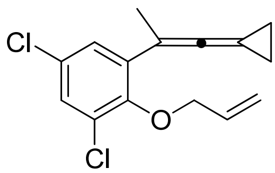

Compound **3h**. 983 mg, yield: 70%; colorless oil. ^{1}H NMR ($CDCl_3$, 400 MHz, TMS) δ 1.59 (s, 4H, $2CH_2$), 2.13 (s, 3H, CH_3), 4.42-4.44 (m, 2H, CH_2), 5.23 (dd, J_1 = 1.6 Hz, J_2 = 10.8 Hz, 1H, $=CH_2$), 5.36 (dd, J_1 = 1.6 Hz, J_2 = 18.4 Hz, 1H, $=CH_2$), 6.02-6.12 (m, 1H, =CH), 7.14-7.15 (m, 1H, Ar), 7.23-7.26 (m, 1H, Ar). ^{13}C NMR ($CDCl_3$, 100 MHz, TMS) δ 8.0, 19.8, 73.8, 76.3, 98.6, 117.9, 127.2, 127.8, 129.1, 129.5, 133.4, 136.4, 150.9, 190.8. IR (neat) ν 2984, 2913, 2861, 2005, 1575, 1440, 1418, 1370, 1246, 1222, 981, 925, 853, 825, 772 cm^{-1}. MS (%) m/e 280 (M^+, 0.42), 241 (64.76), 239 (100.00), 204 (72.04), 189 (34.96), 169 (95.26), 168 (31.45), 141 (34.13), 139 (43.08). HRMS (EI) calcd. for $C_{15}H_{14}OCl_2$: 280.0422, Found: 280.0421.

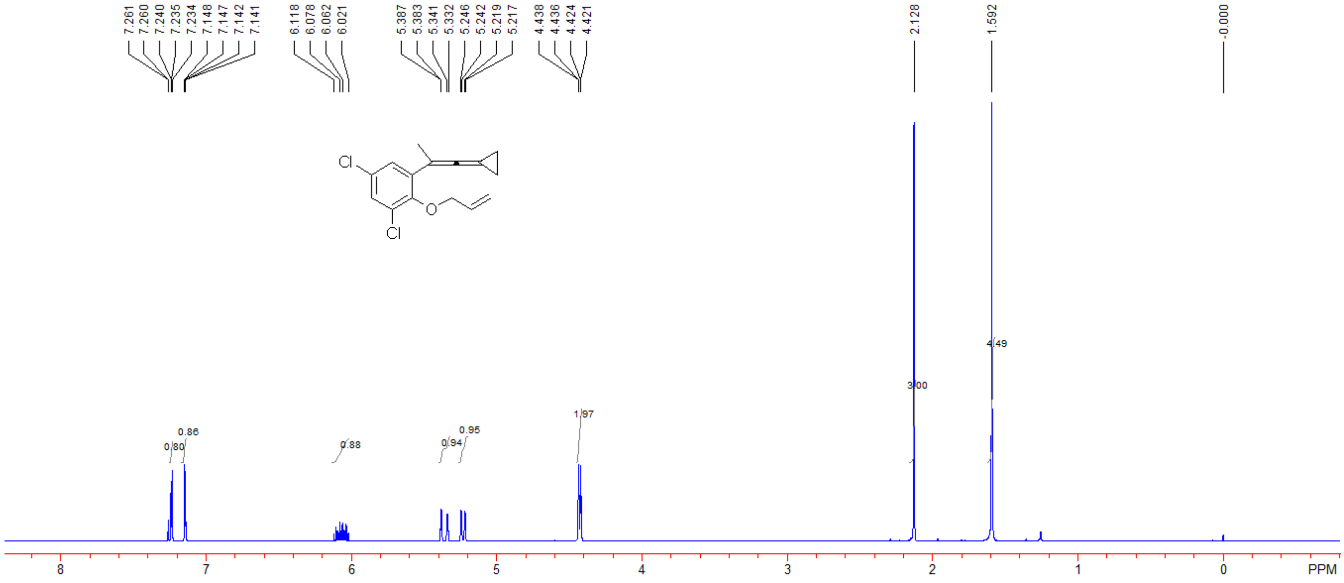

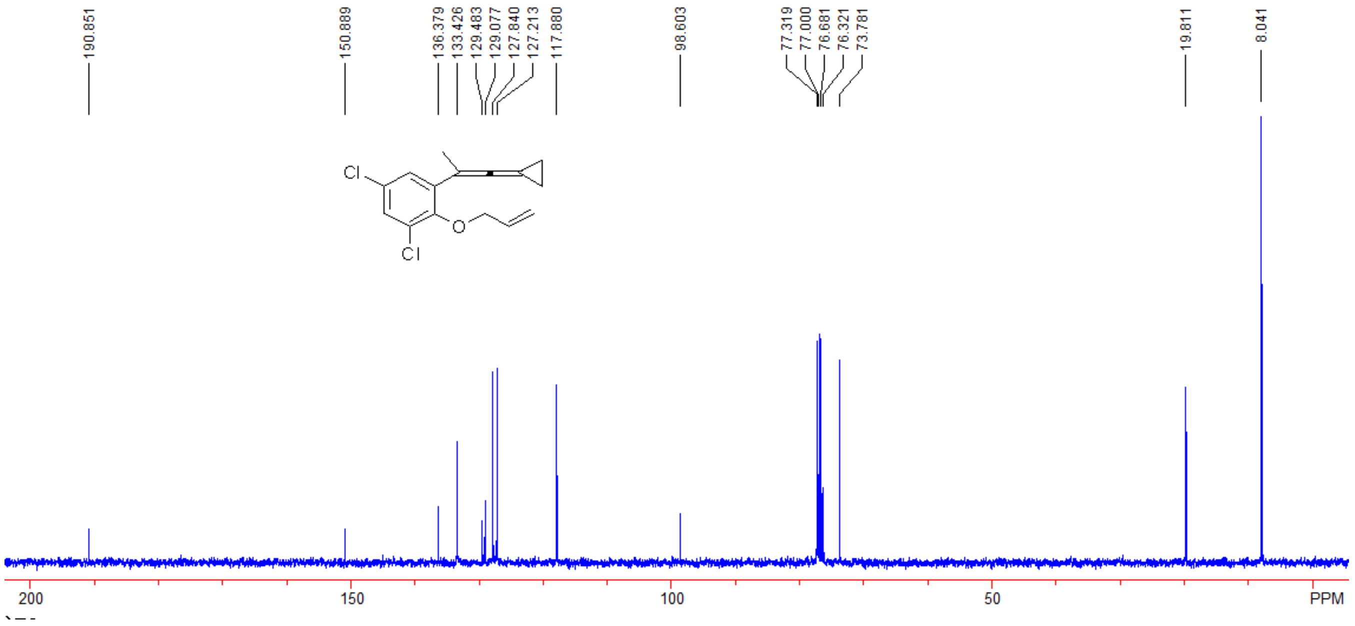

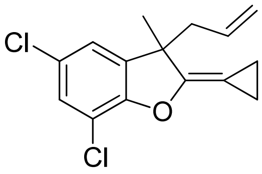

Compound **4h**. 146 mg, yield: 79%; colorless oil. ^{1}H NMR ($CDCl_3$, 400 MHz, TMS) δ 1.38 (s, 3H, CH_3), 2.39-2.42 (m, 4H, 2CH_2), 2.86-2.94 (m, 2H, CH_2), 4.92-4.97 (m, 2H, =CH_2), 5.52-5.62 (m, 1H, =CH), 7.11 (d, *J* = 2.4 Hz, 1H, Ar), 7.22 (d, *J* = 2.4 Hz, 1H, Ar). ^{13}C NMR ($CDCl_3$, 100 MHz, TMS) δ 23.3, 28.4, 31.2, 38.0, 46.9, 116.8, 117.7, 122.9, 126.1, 127.6, 127.8, 132.9, 134.6, 142.2, 147.3. IR (neat) ν 2963, 2928, 2852, 2360, 1741, 1552, 1434, 1346, 1292, 1222, 1075, 915, 859, 771, 763 cm^{-1}. MS (%) m/e 280 (M^+, 1.41), 243 (10.78), 241 (66.96), 240 (14.89), 239 (100.00), 169 (20.58), 141 (11.05), 139 (13.63), 115 (10.99). HRMS (EI) calcd. for $C_{15}H_{14}OCl_2$: 280.0422, Found: 280.0425.

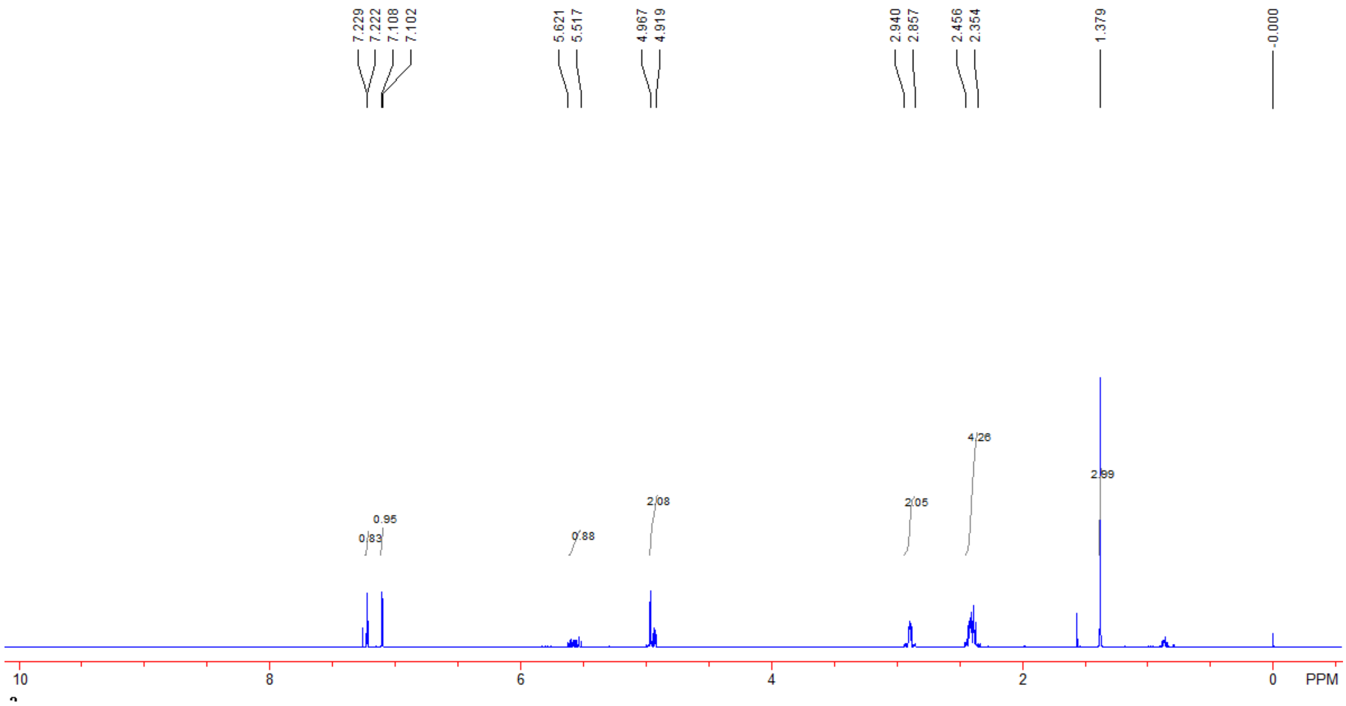

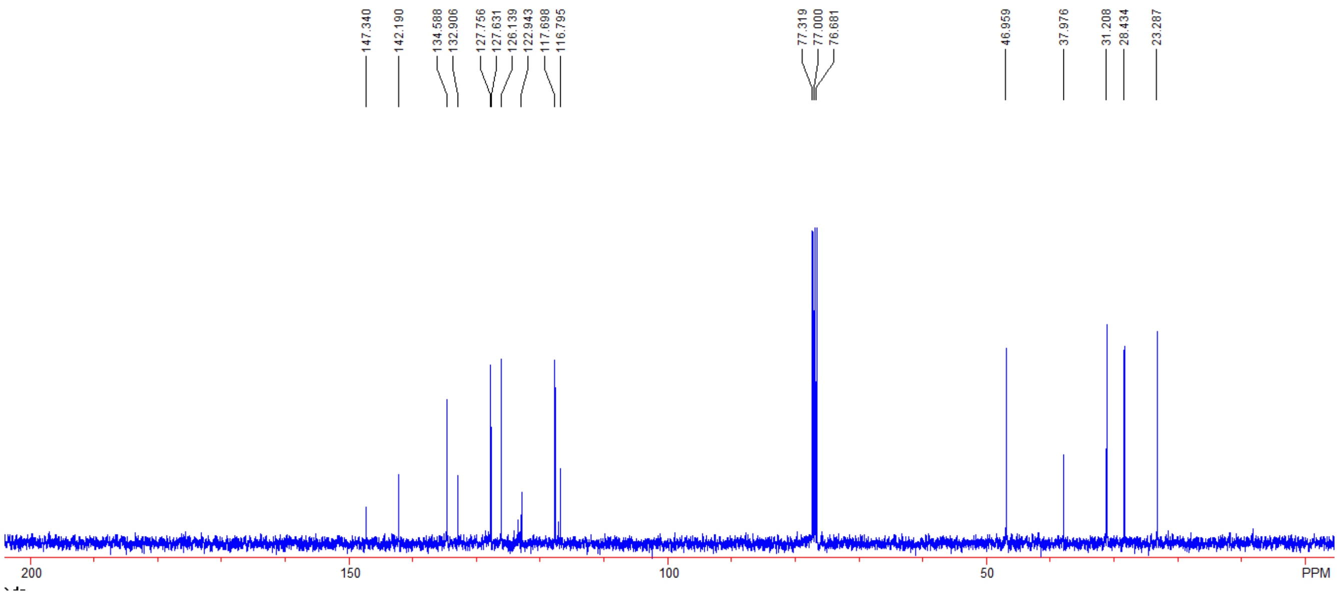

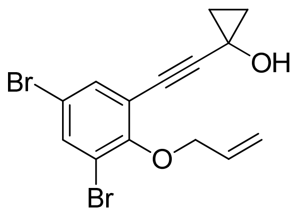

Compound **S3u**. 1.425 g, yield: 35%; yellow oil. ^{1}H NMR ($CDCl_3$, 400 MHz, TMS) δ 1.07-1.11 (m, 2H, CH_2), 1.19-1.22 (m, 2H, CH_2), 3.24 (br, 1H, OH), 4.59 (d, J = 6.0 Hz, 2H, CH_2), 5.27 (dd, J_1 = 1.2 Hz, J_2 = 10.4 Hz, 1H, =CH_2), 5.42 (dd, J_1 = 1.2 Hz, J_2 = 17.2 Hz, 1H, =CH_2), 6.06-6.16 (m, 1H, =CH), 7.42 (d, J = 2.4 Hz, 1H, Ar), 6.71 (d, J = 2.4 Hz, 1H, Ar). ^{13}C NMR ($CDCl_3$, 100 MHz, TMS)

δ 17.7, 45.7, 74.5, 76.7, 97.7, 116.3, 118.5, 118.6, 120.3, 133.0, 134.7, 135.4, 155.8. IR (neat) ν 3378, 3084, 3008, 2955, 2220, 1709, 1573, 1537, 1440, 1418, 1234, 1161, 1047, 977, 858, 752, 712 cm^{-1}. MS (%) m/e 169 (25.46), 128 (23.76), 115 (77.93), 114 (42.86), 87 (32.31), 86 (33.64), 55 (100.00), 41 (47.45). HRMS (EI) calcd. for $C_{14}H_{12}O_2Br_2$: 369.9204, Found: 369.9202.

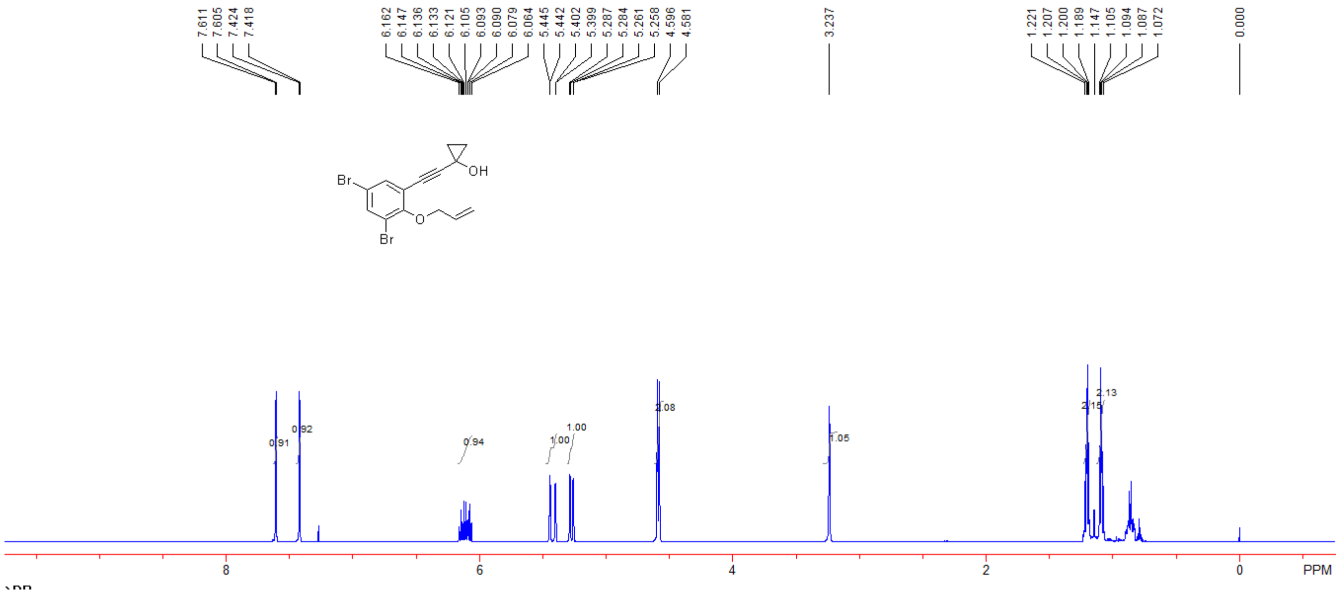

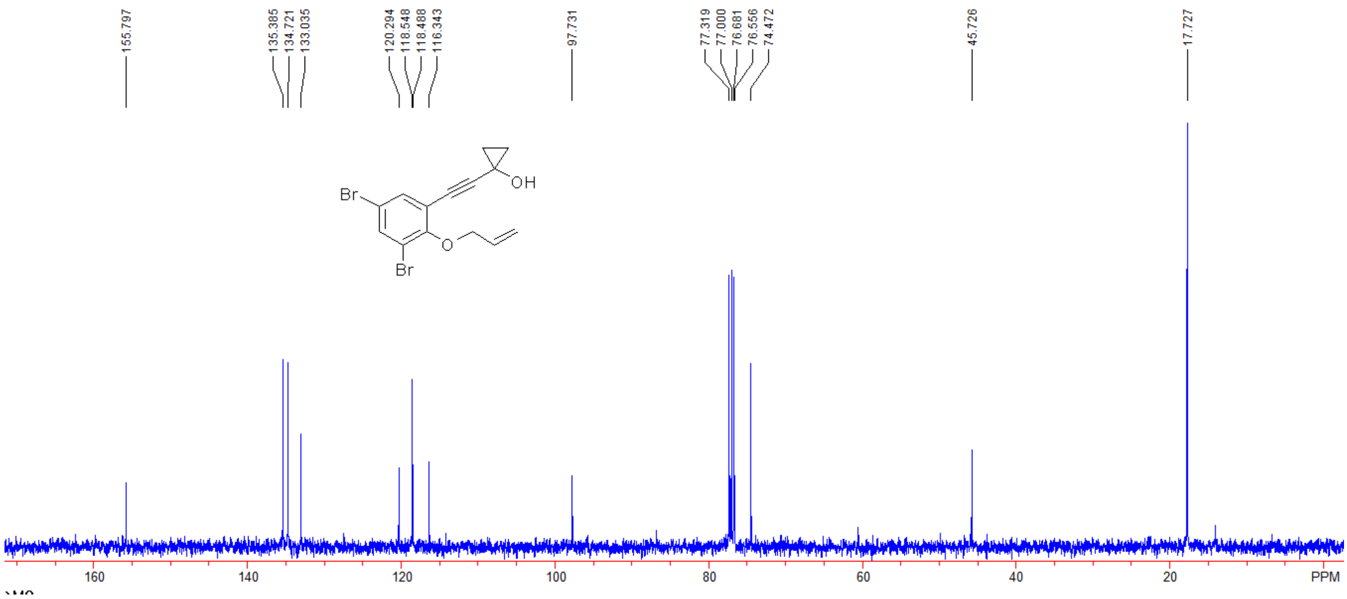

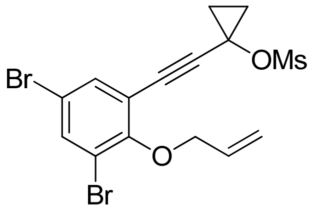

Compound **S4u**. 1.575 g, yield: 92%; yellow oil. ^{1}H NMR ($CDCl_3$, 400 MHz, TMS) δ 1.35-1.39 (m,

2H, CH_2), 1.66-1.70 (m, 2H, CH_2), 3.22 (s, 3H, CH_3), 4.60-4.62 (m, 2H, CH_2), 5.28-5.32 (m, 1H, =CH_2), 5.42-5.47 (m, 1H, =CH_2), 6.08-6.17 (m, 1H, =CH), 7.46 (d, J = 2.4 Hz, 1H, Ar), 7.67 (d, J = 2.4 Hz, 1H, Ar). ^{13}C NMR ($CDCl_3$, 100 MHz, TMS) δ 16.9, 39.8, 53.8, 74.7, 80.1, 92.1, 116.5, 118.6, 118.9, 119.2, 132.7, 134.8, 136.4, 156.2. IR (neat) ν 3089, 3016, 2931, 2226, 1730, 1538, 1442, 1362, 1205, 1171, 1154, 974, 936, 863, 797 cm^{-1}. MS (%) m/e 448 (M^+, 0.02), 165 (46.15), 155 (47.86), 127 (47.46), 126 (100.00), 114 (74.85), 86 (60.81), 55 (79.92), 41 (93.89). HRMS (EI) calcd. for $C_{15}H_{14}O_4Br_2S$: 447.8980, Found: 447.8978.

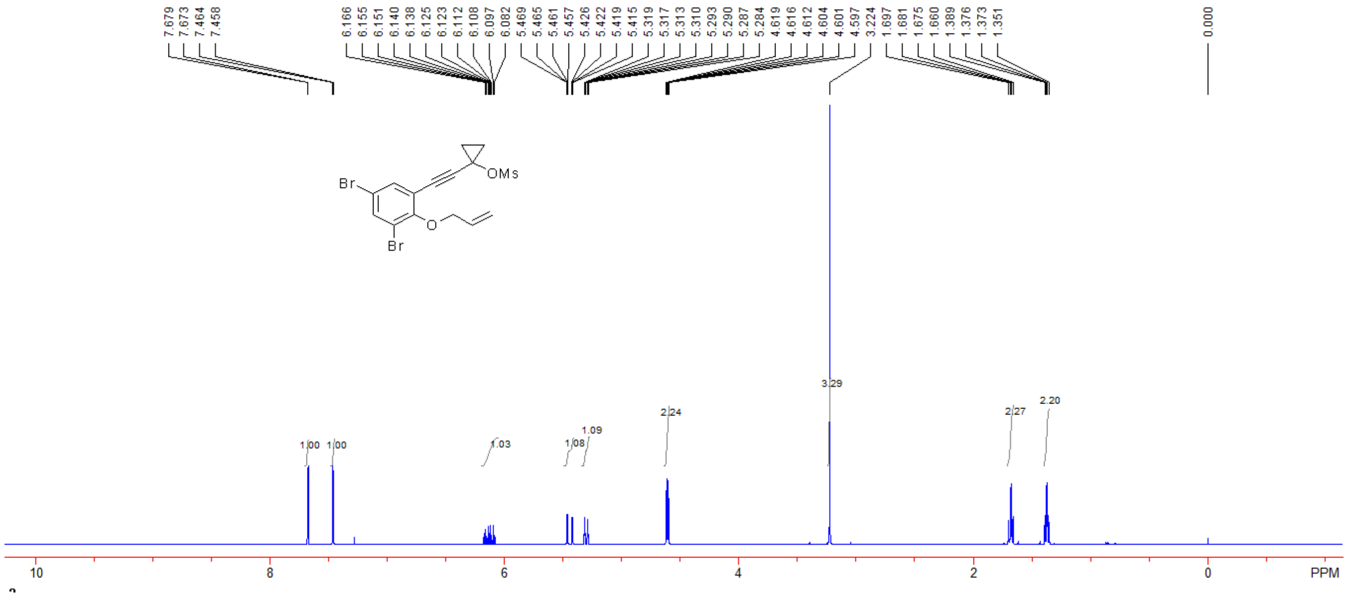

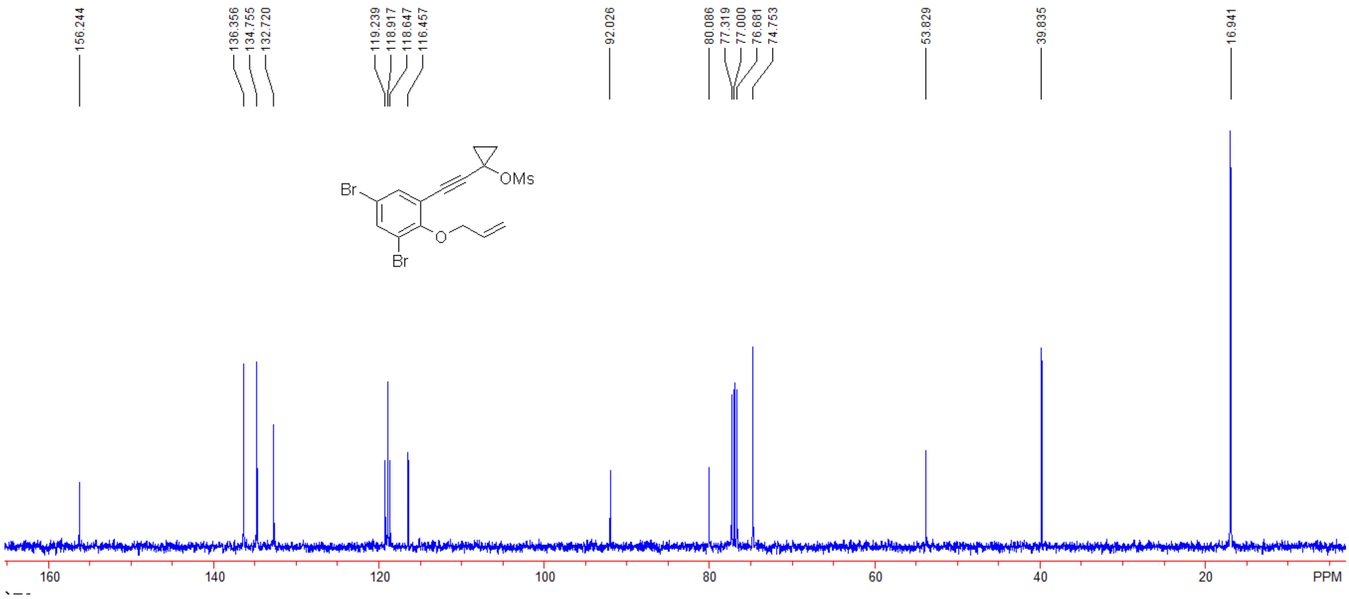

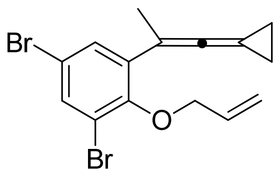

Compound **3i**. 980 mg, yield: 75%; yellow solid; MP: 55-56 °C. ^{1}H NMR ($CDCl_3$, 400 MHz, TMS) δ 1.59 (s, 4H, $2CH_2$), 2.12 (s, 3H, CH_3), 4.42 (d, J = 6.4 Hz, 2H, CH_2), 5.23 (dd, J_1 =1.2 Hz, J_2 = 10.4 Hz, 1H, $=CH_2$), 5.37 (dd, J_1 = 1.2 Hz, J_2 = 17.2 Hz, 1H, $=CH_2$), 6.03-6.13 (m, 1H, =CH), 7.33 (d, J = 2.4 Hz, 1H, Ar), 7.54 (d, J = 2.4 Hz, 1H, Ar). ^{13}C NMR ($CDCl_3$, 100 MHz, TMS) δ 8.0, 19.7, 73.6, 76.4, 98.6, 116.9, 117.9, 119.3, 130.9, 133.3, 133.4, 136.7, 152.3, 190.7. IR (neat) ν 2983, 2901, 2855, 2001, 1713, 1567, 1433, 1242, 1227, 1094, 919, 984, 858, 805, 713 cm^{-1}. MS (%) m/e 368 (M^+, 0.14), 329 (43.87), 327 (23.08), 235 (22.81), 233 (22.95), 169 (100.00), 168 (23.05), 141 (25.26), 139 (28.56). HRMS (EI) calcd. for $C_{15}H_{14}OBr_2$: 367.9411, Found: 367.9408.

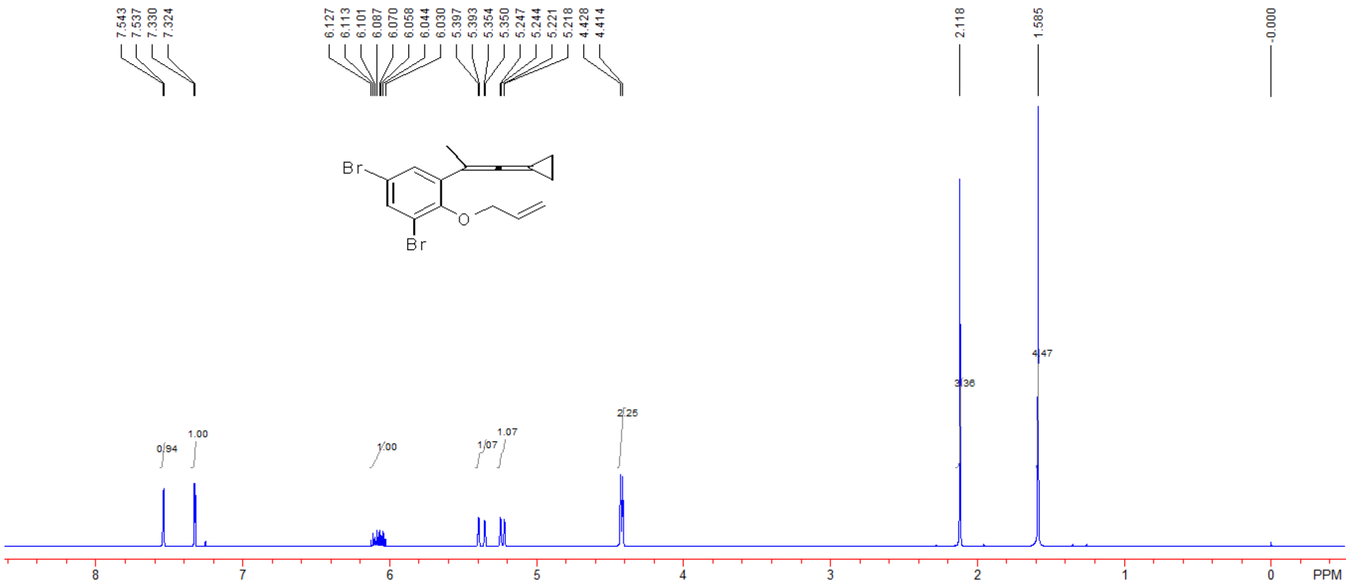

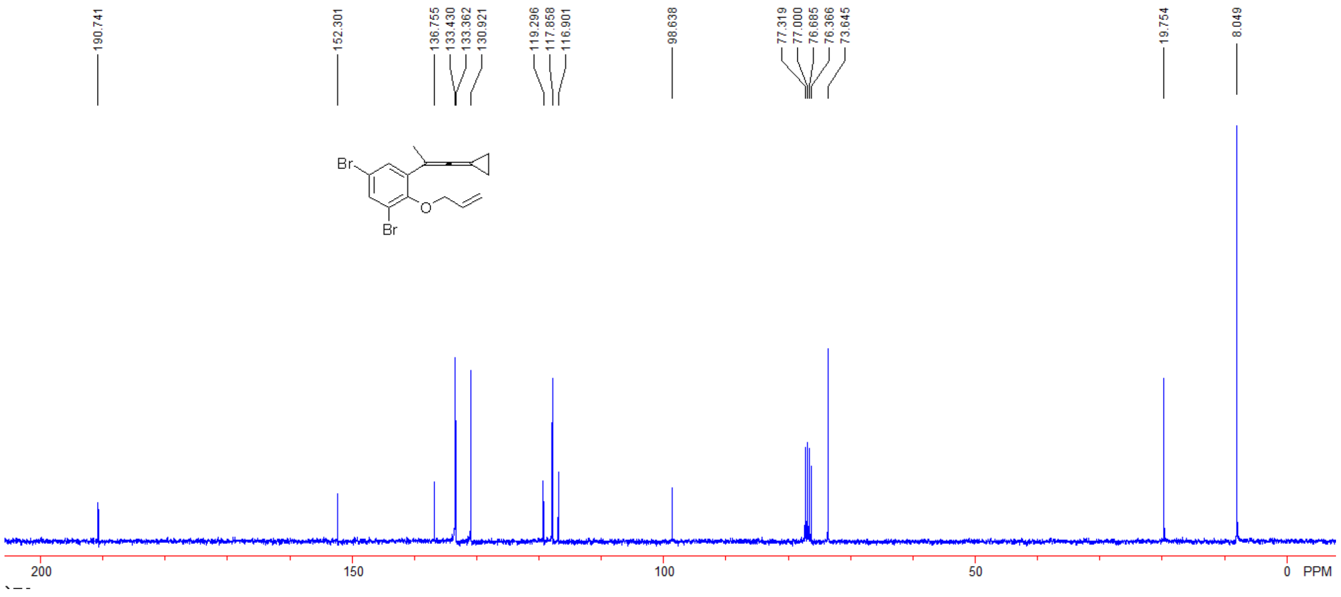

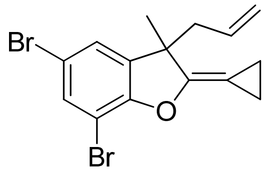

Compound **4i**. 146 mg, yield: 79%; colorless oil. ^{1}H NMR ($CDCl_3$, 400 MHz, TMS) δ 1.38 (s, 3H, CH_3), 2.33-2.44 (m, 4H, 2CH_2), 2.86-2.94 (m, 2H, CH_2), 4.93-4.97 (m, 2H, =CH_2), 5.52-5.62 (m, 1H, =CH), 7.28 (d, J = 2.4 Hz, 1H, Ar), 7.52 (d, J = 2.4 Hz, 1H, Ar). ^{13}C NMR ($CDCl_3$, 100 MHz, TMS) δ 23.3, 28.5, 31.3, 38.0, 47.0, 112.2, 115.2, 116.9, 117.7, 129.7, 129.8, 133.3, 134.6, 142.2, 148.6. IR (neat) ν 3074, 2961, 2926, 2851, 1740, 1543, 1426, 1372, 1345, 1216, 1176, 1071, 914, 860, 729 cm^{-1}. MS (%) m/e 368 (M^+, 0.45), 331 (52.56), 329 (100.00), 327 (53.02), 169 (100.00), 141 (25.26), 139 (28.56), 115 (24.44), 69 (16.96). HRMS (EI) calcd. for $C_{15}H_{14}OBr_2$: 367.9411, Found: 367.9410.

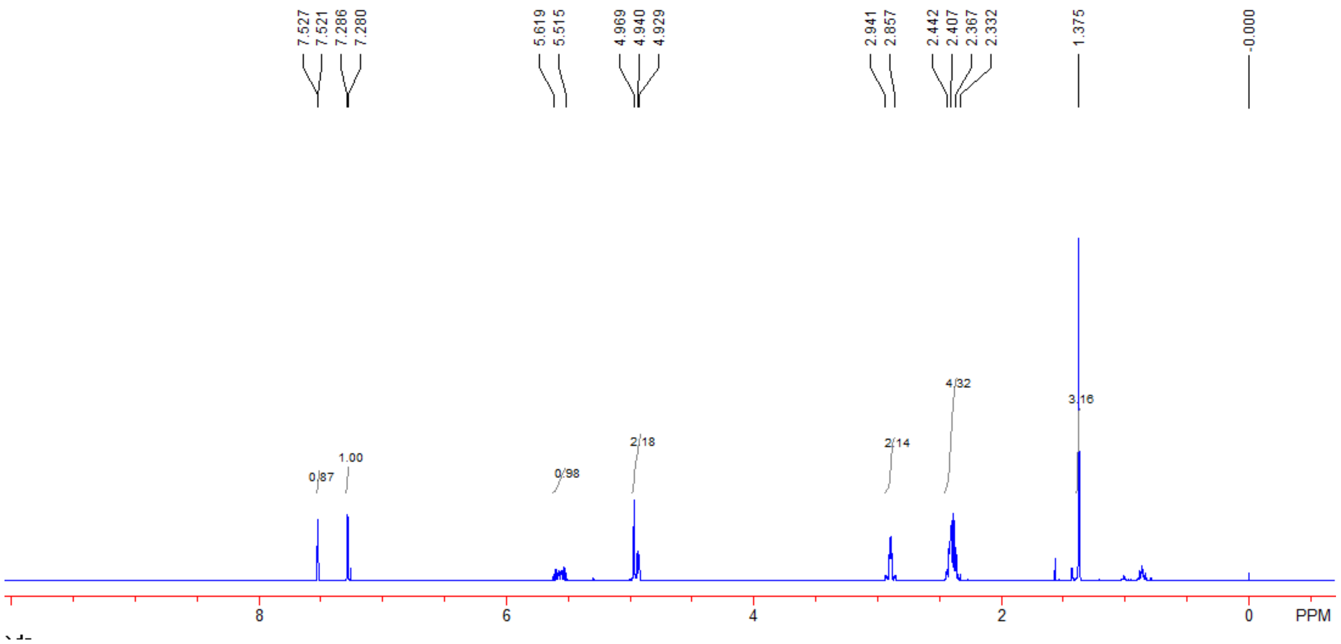

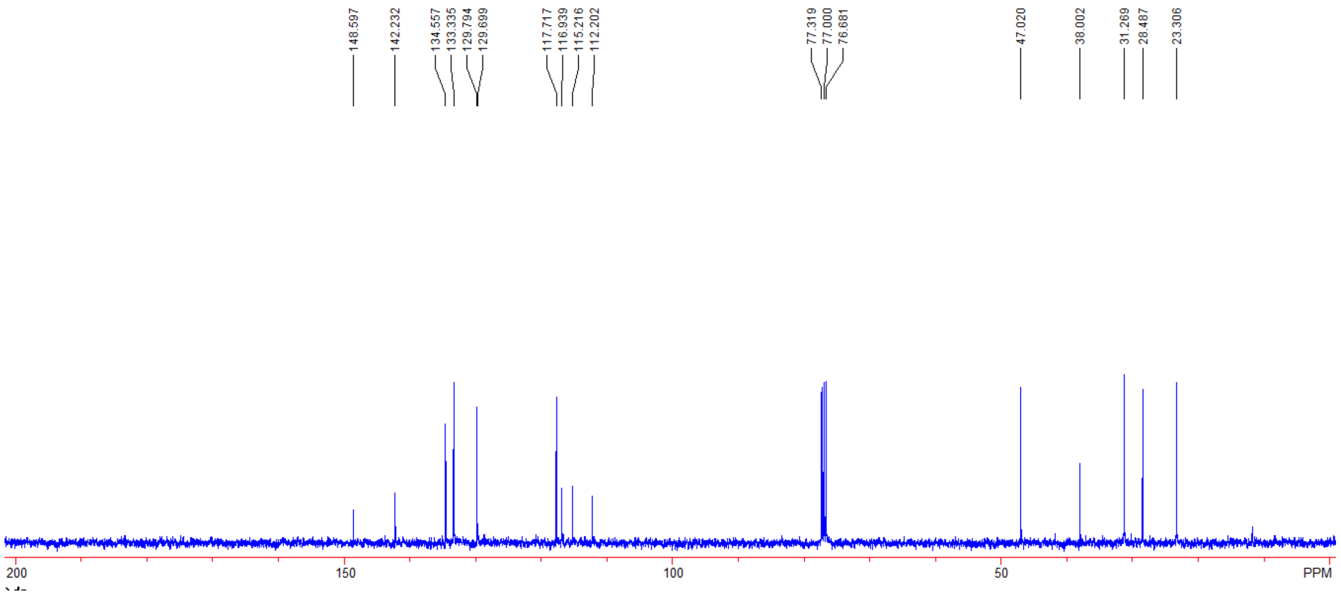

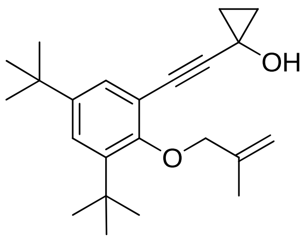

Compound **S3v**. 2.723 g, yield: 57%; yellow oil. ^{1}H NMR ($CDCl_3$, 400 MHz, TMS) δ 1.07-1.08 (m, 2H, CH_2), 1.14-1.15 (m, 2H, CH_2), 1.28 (s, 9H, ^{t}Bu), 1.38 (s, 9H, ^{t}Bu), 1.87 (s, 3H, CH_3), 2.70 (br, 1H, OH), 4.61 (s, 2H, CH_2), 4.98 (s, 1H, $=CH_2$), 5.22 (s, 1H, $=CH_2$), 7.26 (d, J = 2.0 Hz, 1H, Ar), 7.30 (d, J = 2.0 Hz, 1H, Ar). ^{13}C NMR ($CDCl_3$, 100 MHz, TMS) δ 17.4, 19.6, 30.6, 31.3, 34.4, 35.1,

46.2, 75.6, 80.2, 94.8, 111.0, 116.1, 124.8, 128.7, 141.8, 141.9, 145.2, 156.9. IR (neat) ν 3284, 2960, 2869, 1436, 1413, 1381, 1361, 1240, 1167, 1124, 1038, 995, 969, 881 cm^{-1}. MS (%) m/e 340 (M^+, 5.76), 325 (72.24), 297 (34.55), 283 (38.69), 269 (64.15), 213 (23.31), 57 (100.00), 55 (42.83), 41 (24.25). HRMS (EI) calcd. for $C_{23}H_{32}O_2$: 340.2402, Found: 340.2399.

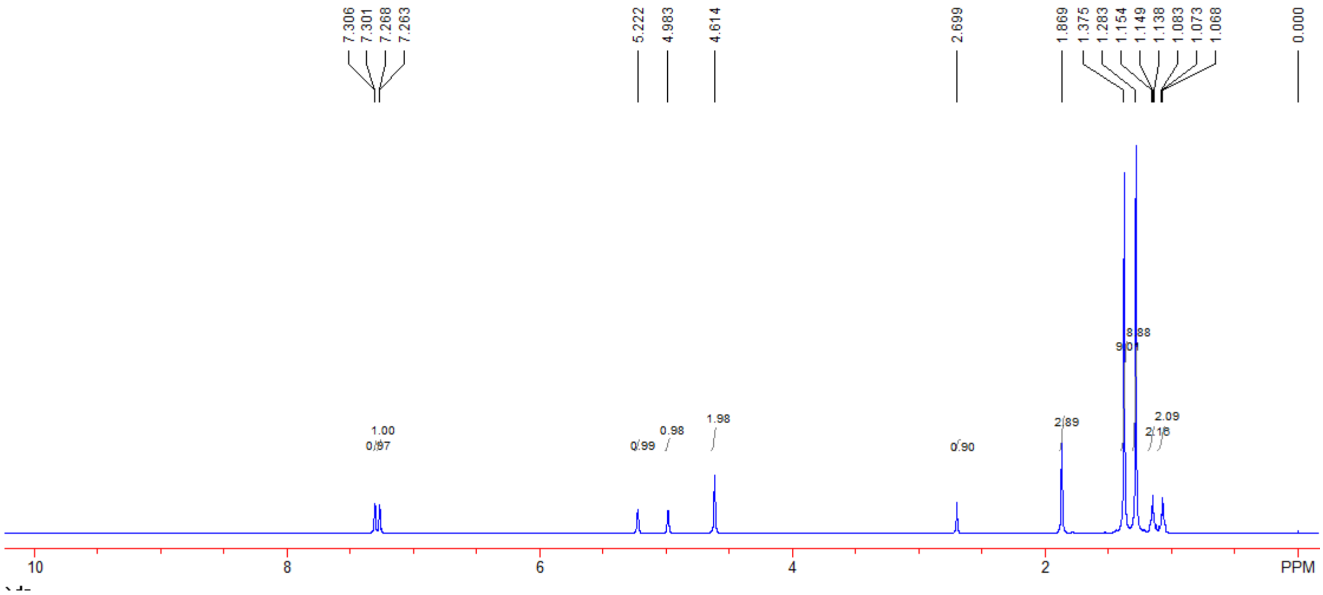

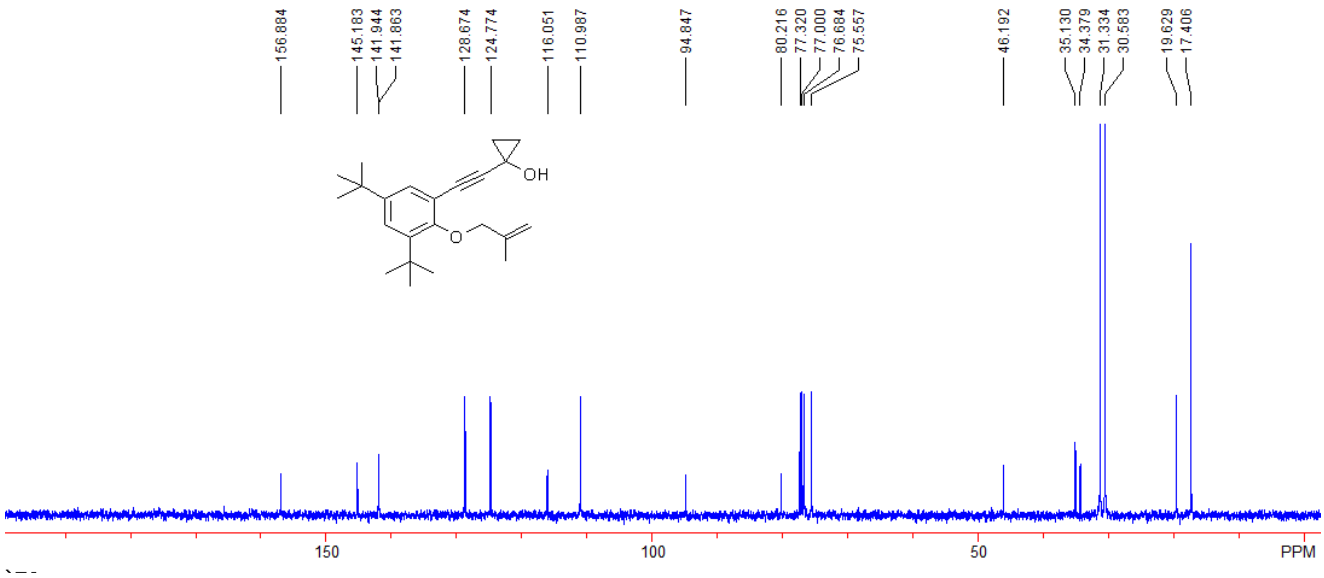

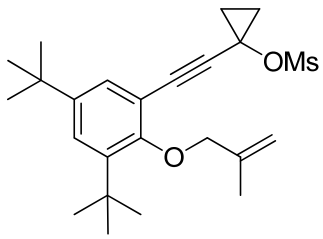

Compound **S4v**. 4.076 g, yield: 92%; yellow oil. ^{1}H NMR ($CDCl_3$, 400 MHz, TMS) δ 1.29 (s, 9H,

^{t}Bu), 1.32-1.37 (m, 11H, ^{t}Bu, CH_2), 1.63-1.66 (m, 2H, CH_2), 1.87 (s, 3H, CH_3), 3.23 (s, 3H, CH_3), 4.62 (s, 2H, CH_2), 5.00 (s, 1H, =CH_2), 5.20 (s, 1H, =CH_2), 7.25 (d, J = 2.0 Hz, 1H, Ar), 7.37 (s, 1H, Ar). ^{13}C NMR ($CDCl_3$, 100 MHz, TMS) δ 16.8, 19.5, 30.5, 31.3, 34.4, 35.2, 39.8, 54.9, 75.8, 84.1, 89.6, 111.2, 114.9, 125.9, 128.9, 141.3, 142.3, 145.5, 157.4. IR (neat) ν 2961, 2904, 2864, 2219, 1436, 1362, 1230, 1173, 1158, 1037, 939, 907, 883, 766, 729 cm^{-1}. MS (%) m/e 418 (M^+, 9.35), 269 (35.44), 268 (48.67), 253 (82.44), 86 (33.15), 84 (48.21), 57 (100.00), 55 (35.94), 41 (25.38). HRMS (EI) calcd. for $C_{24}H_{34}O_4S$: 418.2178, Found: 418.2176.

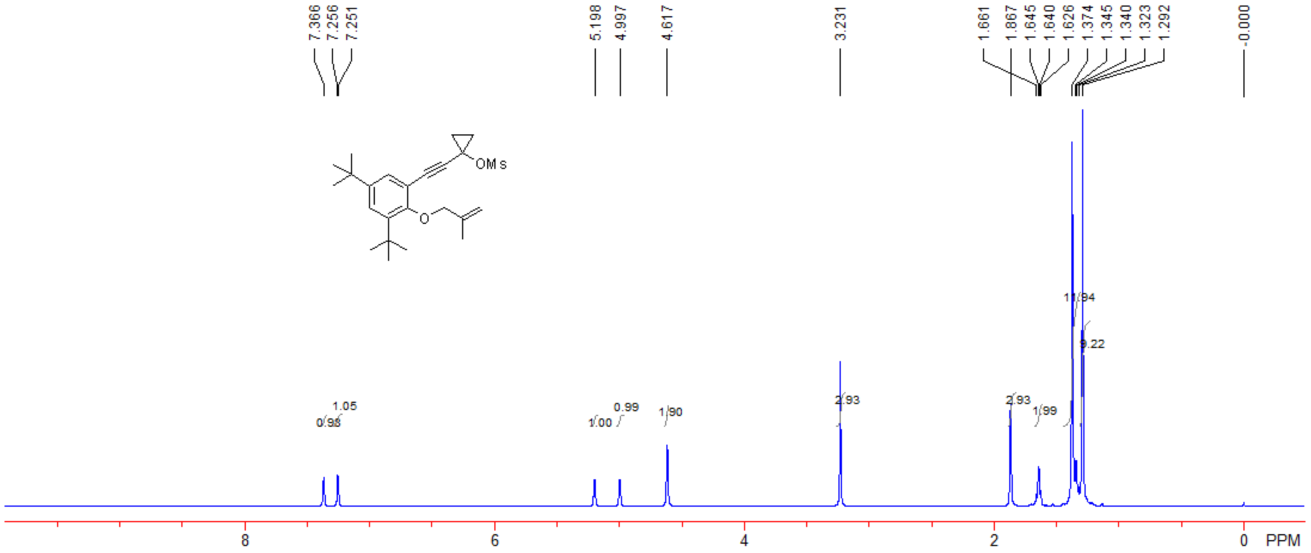

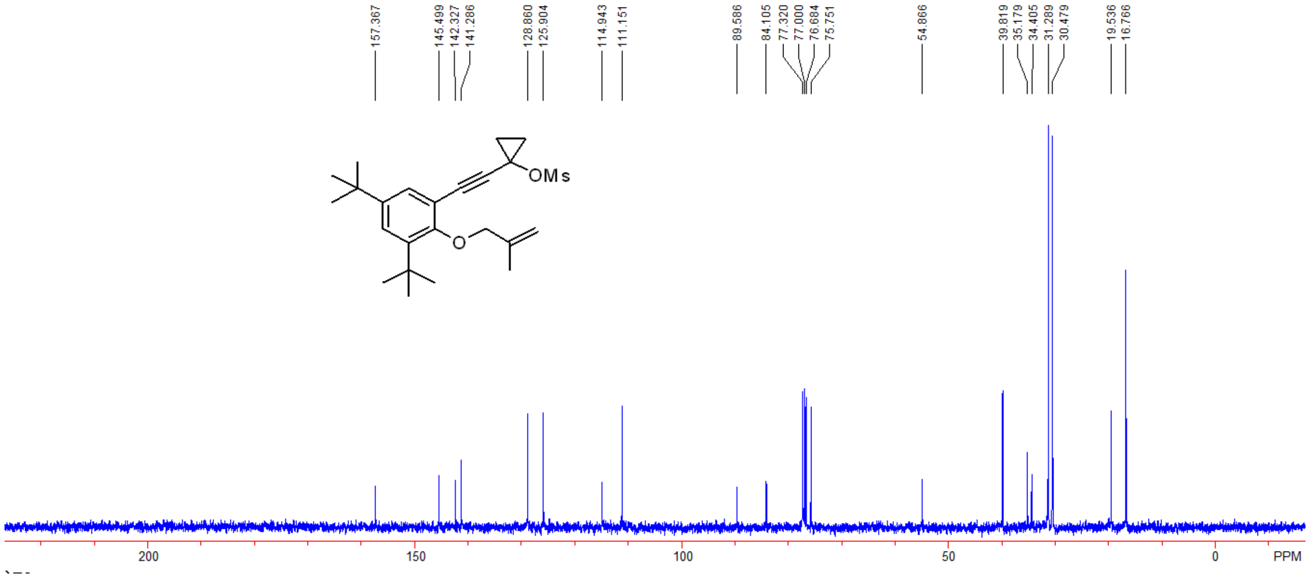

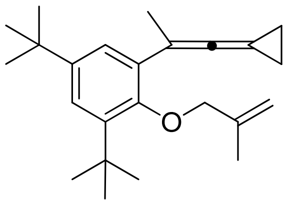

Compound **3j**. 710 mg, yield: 70%; colorless oil. ^{1}H NMR ($CDCl_3$, 400 MHz, TMS) δ 1.28-1.33 (m, 9H, ^{t}Bu), 1.38-1.42 (m, 9H, ^{t}Bu), 1.50-1.52 (m, 4H, $2CH_2$), 1.84-1.86 (m, 3H, CH_3), 2.14-2.17 (m, 3H, CH_3), 4.42-4.45 (m, 2H, CH_2), 4.94-4.97 (m, 1H, $=CH_2$), 5.18-5.21 (m, 1H, $=CH_2$), 7.13-7.16 (m, 1H, Ar), 7.25-7.27 (m, 1H, Ar). ^{13}C NMR ($CDCl_3$, 100 MHz, TMS) δ 7.3, 19.7, 19.8, 30.8, 31.5, 34.4, 35.3, 75.0, 75.9, 103.2, 110.7, 123.1, 125.4, 133.0, 142.0, 142.2, 145.0, 153.1, 189.5. IR (neat) ν 2960, 2901, 2868, 2014, 1654, 1434, 1361, 1224, 1208, 1129, 1046, 997, 906, 880, 730 cm^{-1}. MS (%) m/e 338 (M^+, 0.52), 284 (22.56), 283 (100.00), 267 (7.36), 253 (8.71), 171 (6.35), 57 (29.75), 55 (10.03), 41 (6.55). HRMS (EI) calcd. for $C_{24}H_{34}O$: 338.2610, Found: 338.2615.

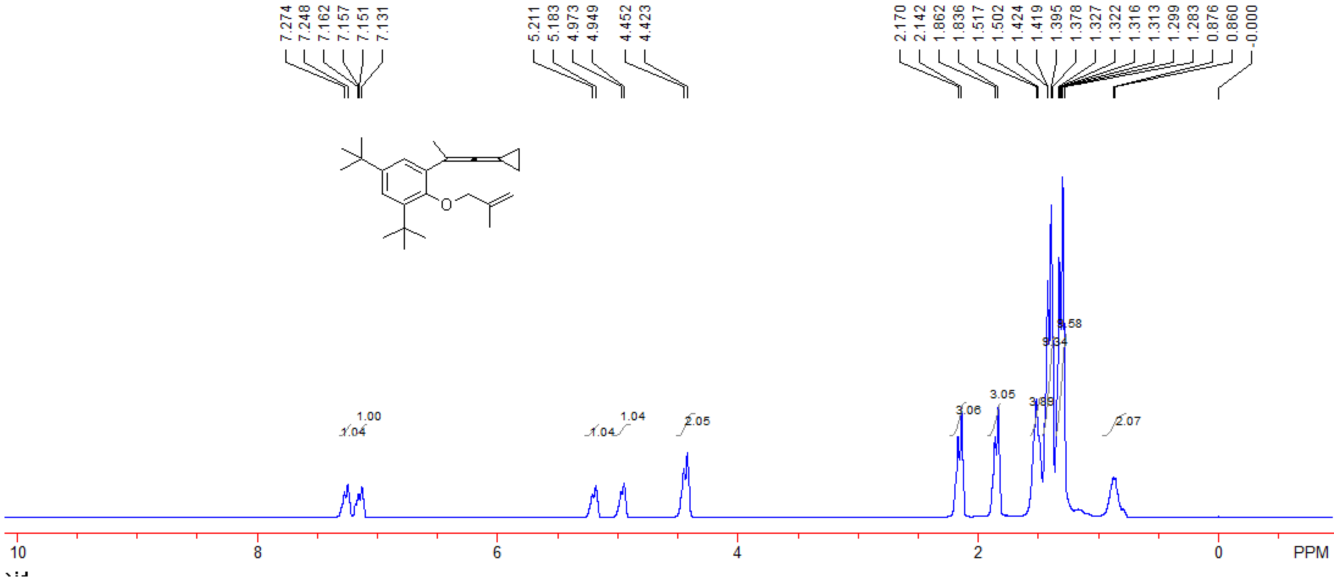

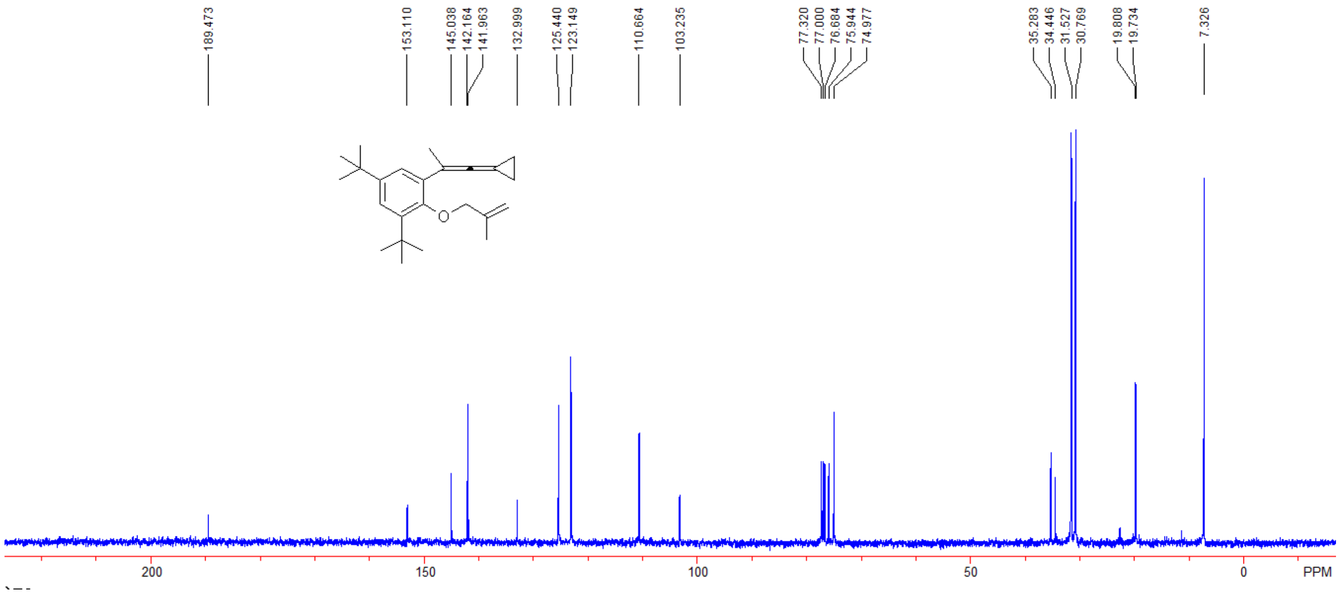

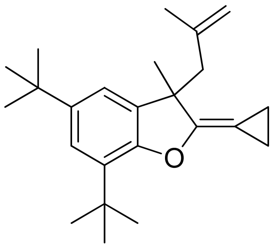

Compound **4j**. 152 mg, yield: 90%; colorless oil. ^{1}H NMR ($CDCl_3$, 400 MHz, TMS) δ 1.29 (s, 9H, ^{t}Bu), 1.33 (s, 3H, CH_3), 1.38 (s, 9H, ^{t}Bu), 1.43 (s, 3H, CH_3), 2.37-2.41 (m, 2H, CH_2), 2.42-2.43 (m, 2H, CH_2), 2.81-2.83 (m, 2H, CH_2), 4.44-4.45 (m, 1H, =CH_2), 4.64-4.65 (m, 1H, =CH_2), 7.15 (s, 2H, Ar). ^{13}C NMR ($CDCl_3$, 100 MHz, TMS) δ 23.3, 23.5, 29.8, 30.0, 31.0, 31.5, 34.4, 35.3, 38.0, 50.8, 113.7, 115.8, 121.0, 122.6, 129.0, 130.5, 142.1, 143.8, 143.9, 149.3. IR (neat) ν 2958, 2919, 2860, 1748, 1639, 1452, 1433, 1361, 1302, 1214, 1164, 1094, 907, 881, 732 cm^{-1}. MS (%) m/e 338 (M^+, 0.11), 284 (22.26), 283 (100.00), 281 (2.16), 268 (2.86), 267 (4.03), 253 (3.13), 211 (2.09), 57 (3.19). HRMS (EI) calcd. for $C_{24}H_{34}O$: 338.2610, Found: 338.2608.

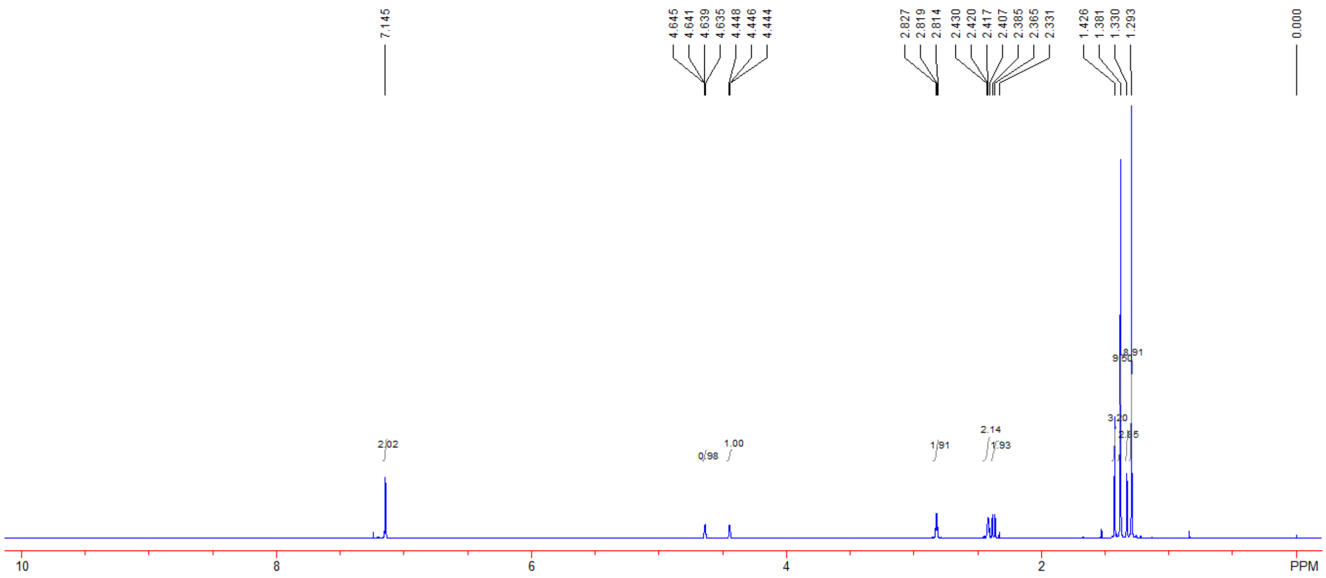

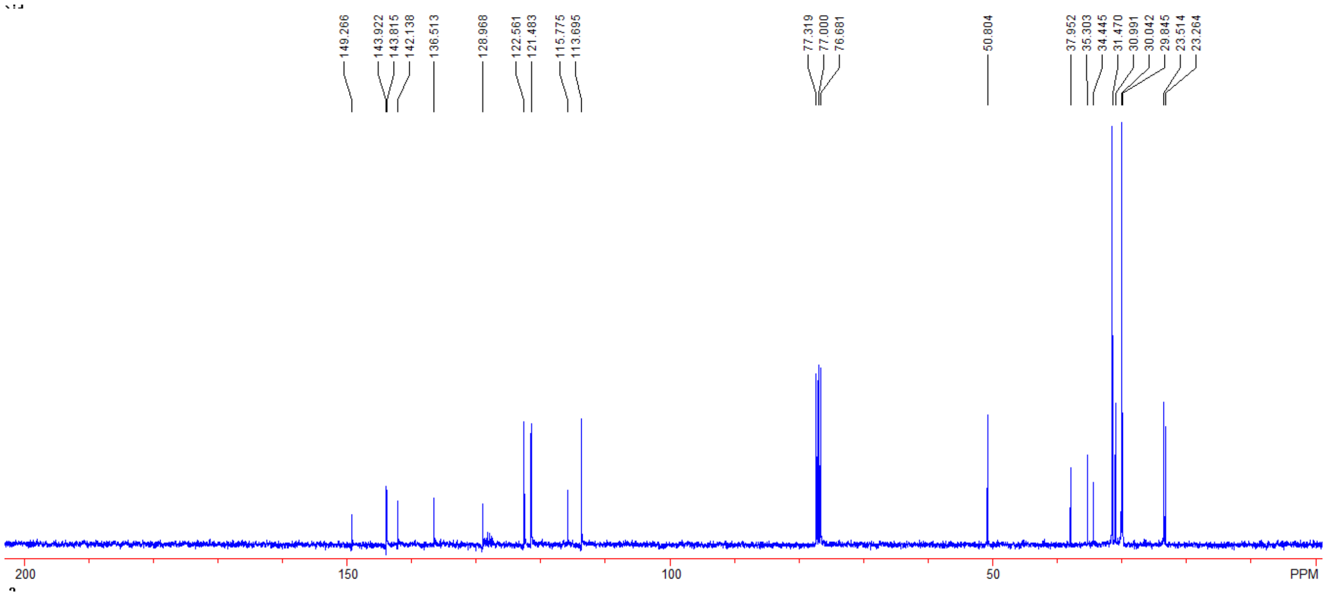

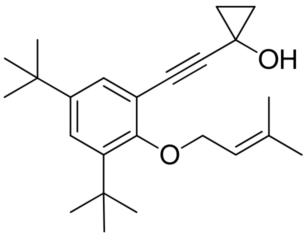

Compound **S3w**. 4.751 g, yield: 67%; yellow oil. ^{1}H NMR ($CDCl_3$, 400 MHz, TMS) δ 1.10-1.12 (m, 2H, CH_2), 1.15-1.17 (m, 2H, CH_2), 1.28 (s, 9H, ^{t}Bu), 1.38 (s, 9H, ^{t}Bu), 1.75 (s, 3H, CH_3), 1.81 (s, 3H, CH_3), 2.71 (br, 1H, OH), 4.72 (d, J = 6.4 Hz, 2H, CH_2), 5.62-5.65 (m, 1H, =CH_2), 7.27-7.30 (m, 2H, Ar). ^{13}C NMR ($CDCl_3$, 100 MHz, TMS) δ 17.5, 18.4, 25.8, 30.7, 31.3, 34.3, 35.1, 46.2, 70.3, 80.6, 94.7, 116.2, 121.1, 124.8, 128.9, 136.2, 141.9, 145.0, 157.3. IR (neat) ν 3387, 2960, 2910, 2869, 1436, 1412, 1379, 1361, 1228, 1201, 1166, 1122, 965, 880, 766 cm^{-1}. MS (%) m/e 354 (M^{+},

0.39), 286 (17.90), 272 (20.61), 271 (100.00), 229 (19.10), 215 (21.29), 69 (19.93), 57 (27.54), 41 (20.17). HRMS (EI) calcd. for $C_{24}H_{34}O_2$: 354.2559, Found: 354.2563.

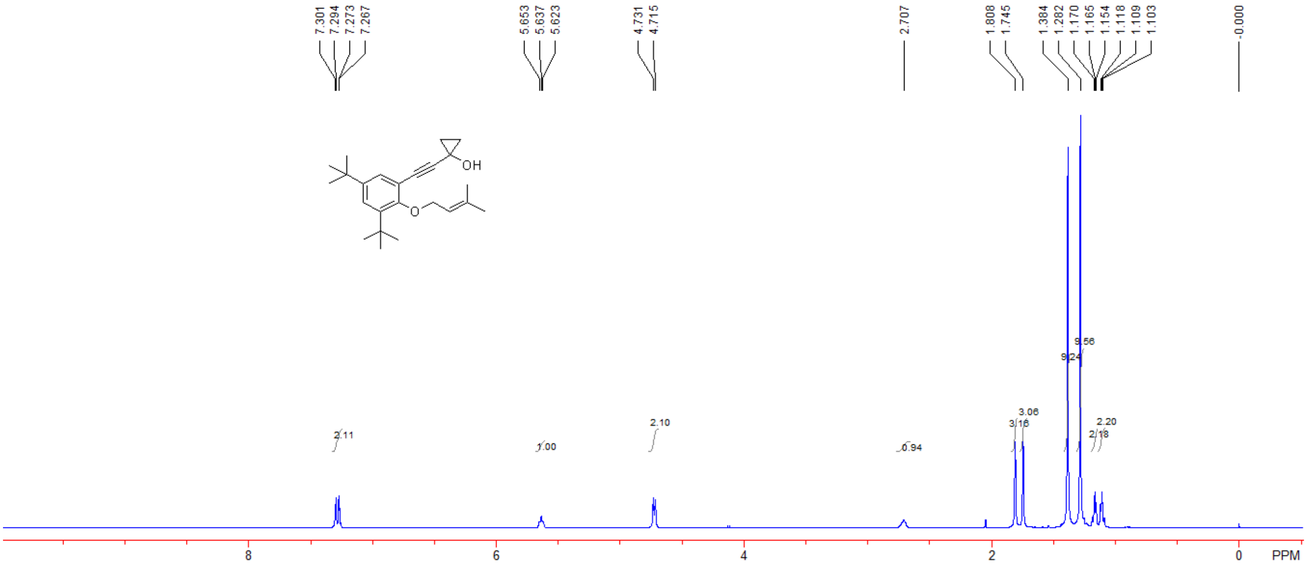

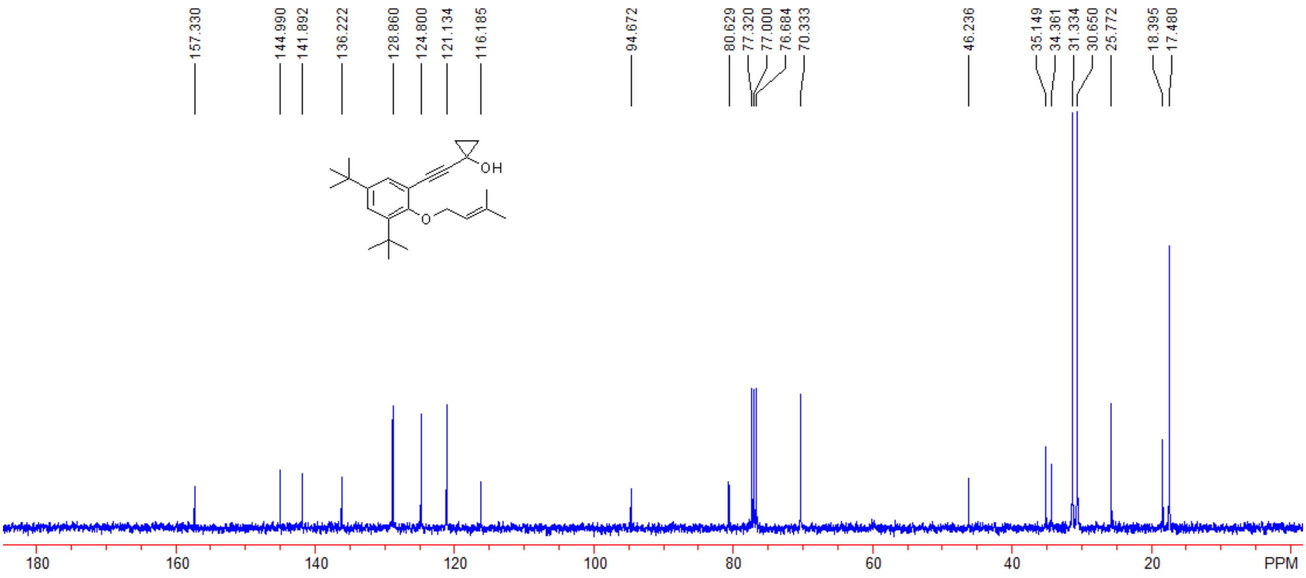

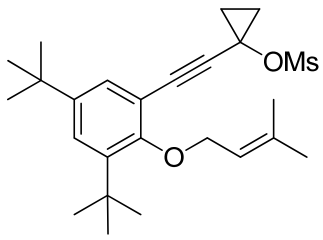

Compound **S4w**. 5.325 g, yield: 92%; yellow oil. ^{1}H NMR ($CDCl_3$, 400 MHz, TMS) δ 1.29 (s, 9H, ^{t}Bu), 1.35-1.40 (m, 11H, ^{t}Bu, CH_2), 1.65-1.68 (m, 2H, CH_2), 1.75 (s, 3H, CH_3), 1.82 (s, 3H, CH_3), 3.25 (s, 3H, CH_3), 4.71 (d, J = 6.4 Hz, 2H, CH_2), 5.60-5.63 (m, 1H, =CH_2), 7.26 (d, J = 2.4 Hz, 1H, Ar), 7.36 (d, J = 2.4 Hz, 1H, Ar). ^{13}C NMR ($CDCl_3$, 100 MHz, TMS) δ 16.8, 18.4, 25.7, 30.6, 31.3,

34.3, 35.2, 39.8, 55.0, 70.7, 84.7, 89.4, 115.0, 120.7, 125.9, 128.9, 136.6, 142.3, 145.3, 157.9. IR (neat) ν 2961, 2908, 2868, 2219, 1437, 1363, 1228, 1200, 1173, 1158, 1036, 939, 882, 821, 729 cm^{-1}. MS (%) m/e 418 (0.14), 364 (40.17), 268 (29.41), 253 (74.89), 243 (100.00), 229 (21.52), 69 (40.91), 57 (47.89), 41 (31.95). HRMS (EI) calcd. for $C_{25}H_{36}O_4S$: 432.2334, Found: 432.2332.

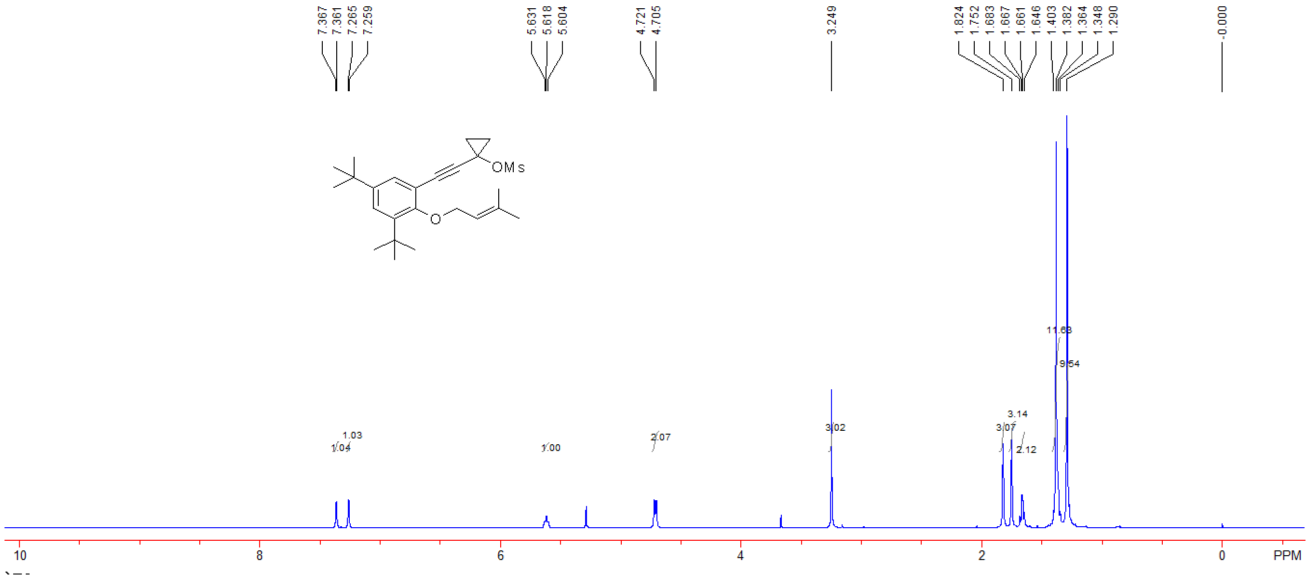

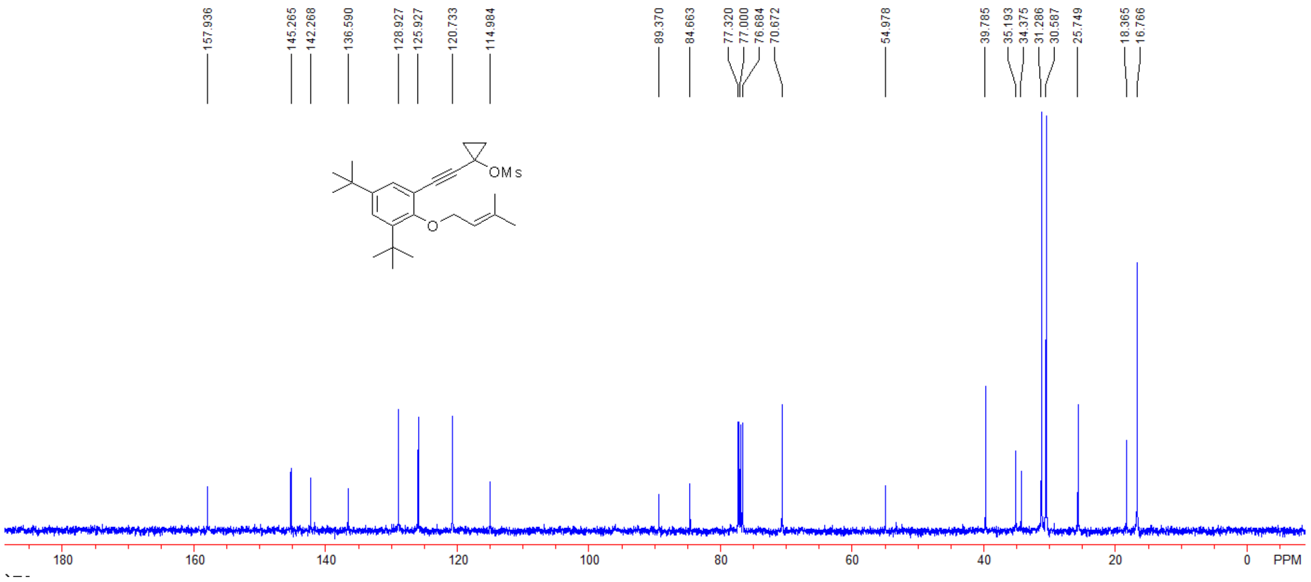

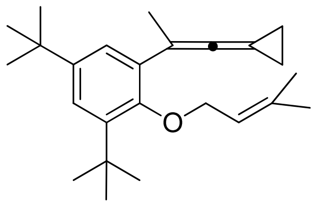

Compound **3k**. 875 mg, yield: 82%; colorless oil. ^{1}H NMR ($CDCl_3$, 400 MHz, TMS) δ 1.29 (s, 9H, ^{t}Bu), 1.41 (s, 9H, ^{t}Bu), 1.50-1.53 (m, 4H, $2CH_2$), 1.71 (s, 3H, CH_3), 1.80 (s, 3H, CH_3), 2.16 (s, 3H, CH_3), 4.53 (d, J = 6.4 Hz, 2H, CH_2), 5.56-5.59 (m, 1H, $=CH_2$), 7.12 (d, J = 2.4 Hz, 1H, Ar), 7.24 (d,

J = 2.4 Hz, 1H, Ar). ^{13}C NMR ($CDCl_3$, 100 MHz, TMS) δ 7.1, 18.1, 19.7, 25.9, 30.9, 31.5, 34.4, 35.3, 69.6, 75.9, 103.5, 121.5, 123.2, 125.5, 133.2, 135.5, 142.1, 144.9, 153.5, 189.4. IR (neat) ν 2960, 2910, 2867, 2014, 1676, 1433, 1361, 1222, 1179, 1071, 976, 907, 880, 731 cm^{-1}. MS (%) m/e 352 (M^+, 0.64), 284 (23.73), 283 (100.00), 269 (14.19), 253 (8.40), 171 (6.95), 69 (17.66), 57 (33.28), 41 (20.26). HRMS (EI) calcd. for $C_{25}H_{36}O$: 352.2766, Found: 352.2765.

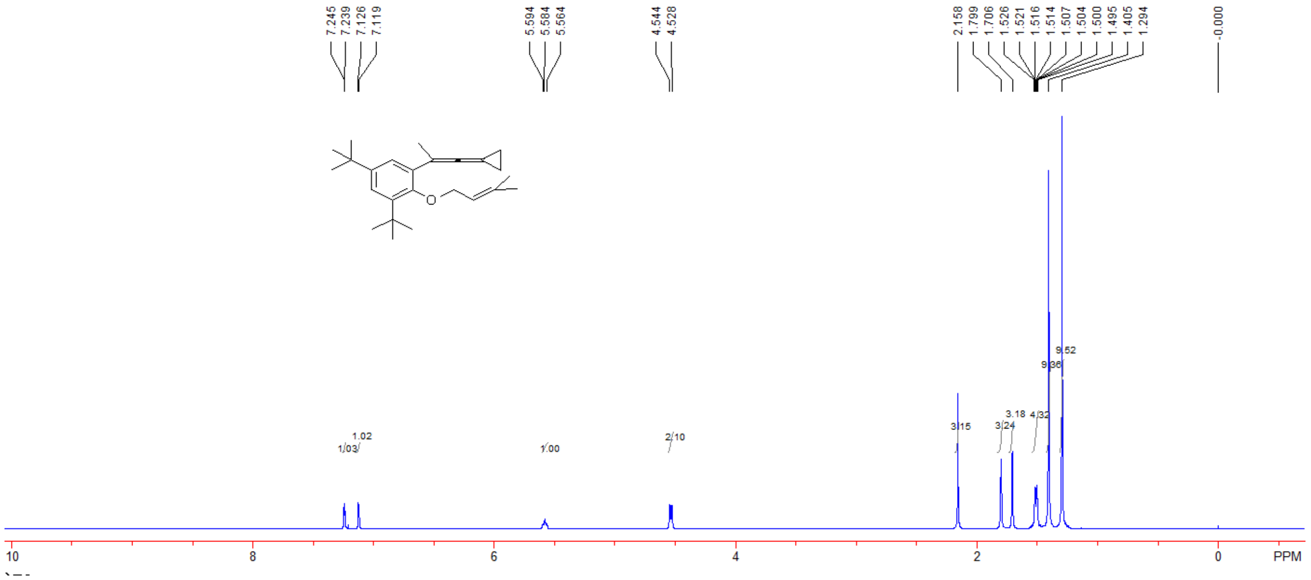

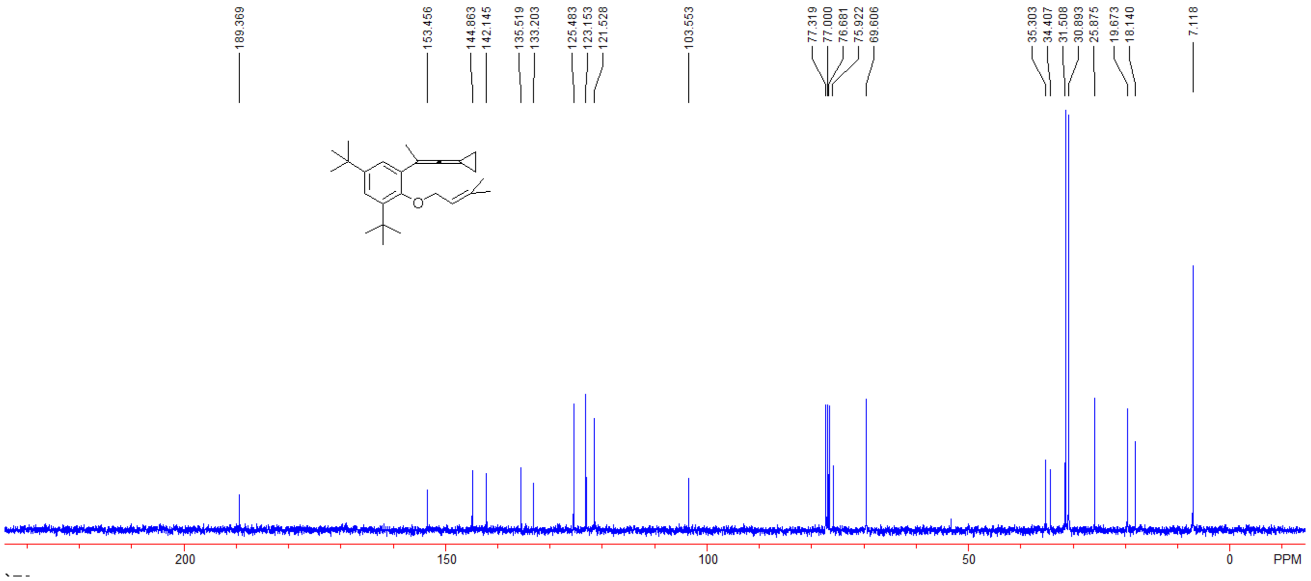

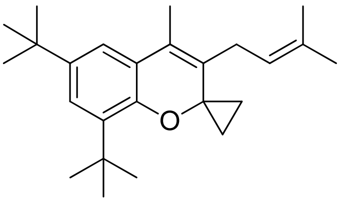

Compound **5k**. 136 mg, yield: 77%; light yellow solid; MP: 125-126 °C. ^{1}H NMR ($CDCl_3$, 400 MHz, TMS) δ 0.77-0.80 (m, 2H, CH_2), 1.16-1.19 (m, 2H, CH_2), 1.31-1.32 (m, 18H, 2^tBu), 1.67 (s,

3H, CH_3), 1.69 (s, 3H, CH_3), 2.05 (s, 3H, CH_3), 2.67-2.69 (m, 2H, CH_2), 5.00-5.03 (m, 1H, $=CH_2$), 7.11 (d, J = 2.4 Hz, 1H, Ar), 7.16 (d, J = 2.4 Hz, 1H, Ar). ^{13}C NMR ($CDCl_3$, 100 MHz, TMS) δ 11.5, 14.1, 17.9, 25.7, 27.1, 30.0, 31.6, 34.5, 34.6, 60.5, 118.2, 122.1, 122.3, 125.6, 125.9, 131.6, 131.8, 135.7, 142.6, 149.6. IR (neat) ν 2952, 2910, 2866, 1439, 1390, 1376, 1360, 1262, 1240, 1118, 1048, 967, 944, 873, 766 cm^{-1}. MS (%) m/e 352 (M^+, 21.46), 337 (49.95), 324 (27.10), 323 (100.00), 309 (51.04), 283 (30.01), 57 (28.64), 41 (16.78). HRMS (EI) calcd. for $C_{25}H_{36}O$: 352.2766, Found: 352.2762.

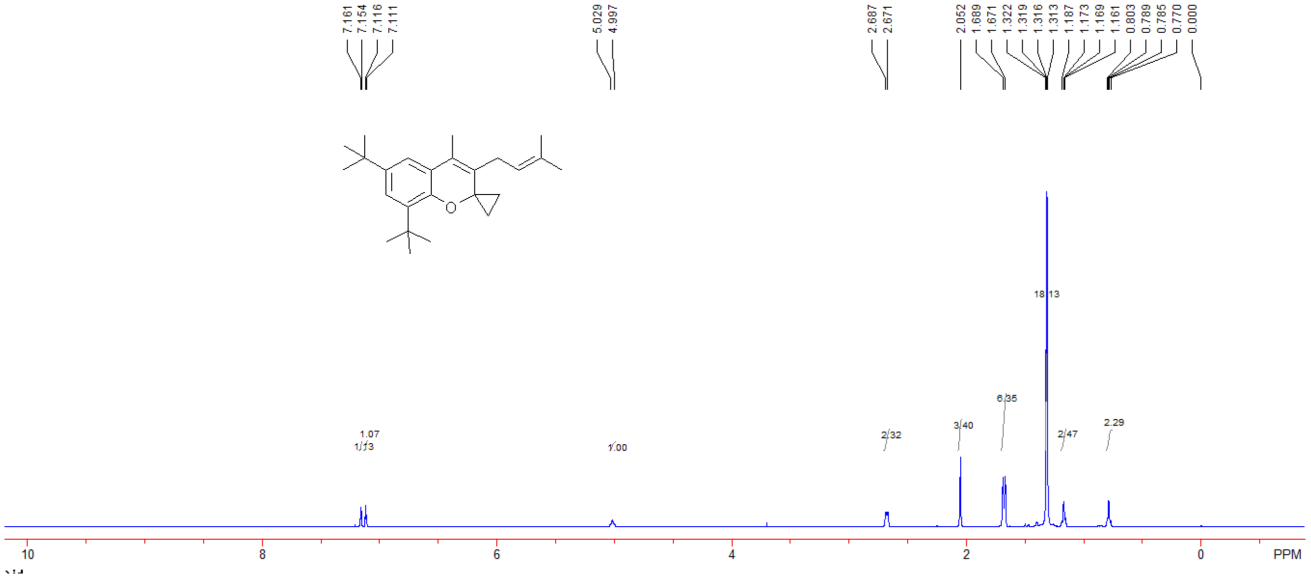

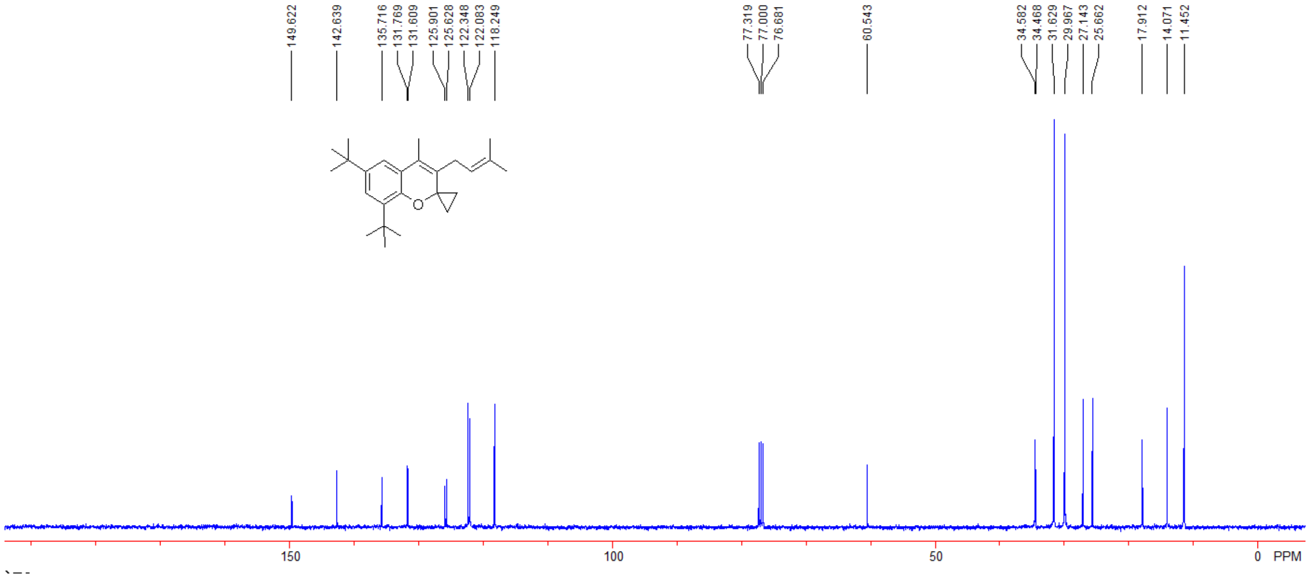

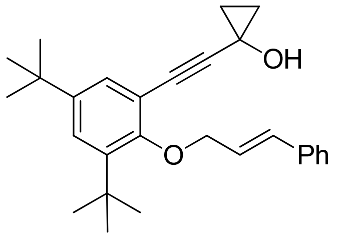

Compound **S3x**. 5.628 g, yield: 56%; yellow oil. ^{1}H NMR ($CDCl_3$, 400 MHz, TMS) δ 1.09-1.10 (m, 2H, CH_2), 1.13-1.14 (m, 2H, CH_2), 1.29 (s, 9H, ^{t}Bu), 1.41 (s, 9H, ^{t}Bu), 2.62 (br, 1H, OH), 4.88-4.89 (m, 2H, CH_2), 6.50-6.57 (m, 1H, =CH), 6.80-6.84 (m, 1H, =CH), 7.25-7.35 (m, 5H, Ar), 7.44-7.46 (m, 2H, Ar). ^{13}C NMR ($CDCl_3$, 100 MHz, TMS) δ 17.6, 30.7, 31.3, 34.4, 35.2, 46.2, 73.3, 80.3, 95.0, 116.1, 124.8, 125.6, 126.5, 127.7, 128.6, 128.7, 131.8, 136.7, 142.0, 145.3, 157.0. IR (neat) ν 3247, 2959, 2902, 2869, 1436, 1372, 1360, 1235, 1201, 1167, 1120, 1025, 963, 880, 745, 692 cm^{-1}. MS (%) m/e 402 (M^{+}, 0.89), 346 (5.21), 345 (13.08), 118 (10.54), 117 (100.00), 115 (18.00), 91 (9.48), 57 (21.68), 41 (6.96). HRMS (EI) calcd. for $C_{28}H_{34}O_2$: 402.2559, Found: 402.2561.

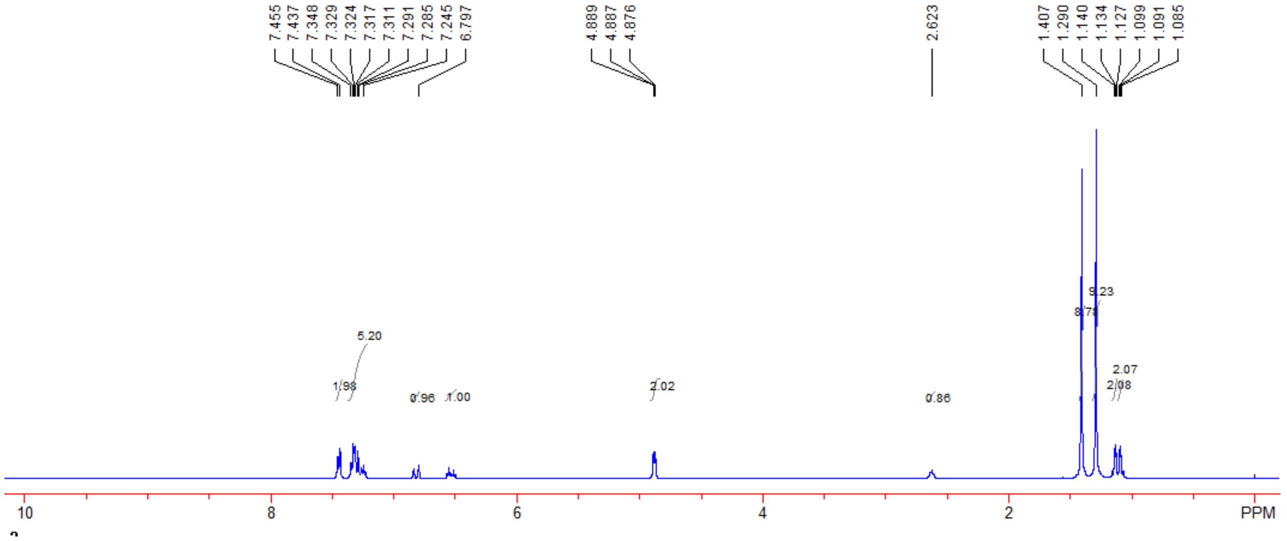

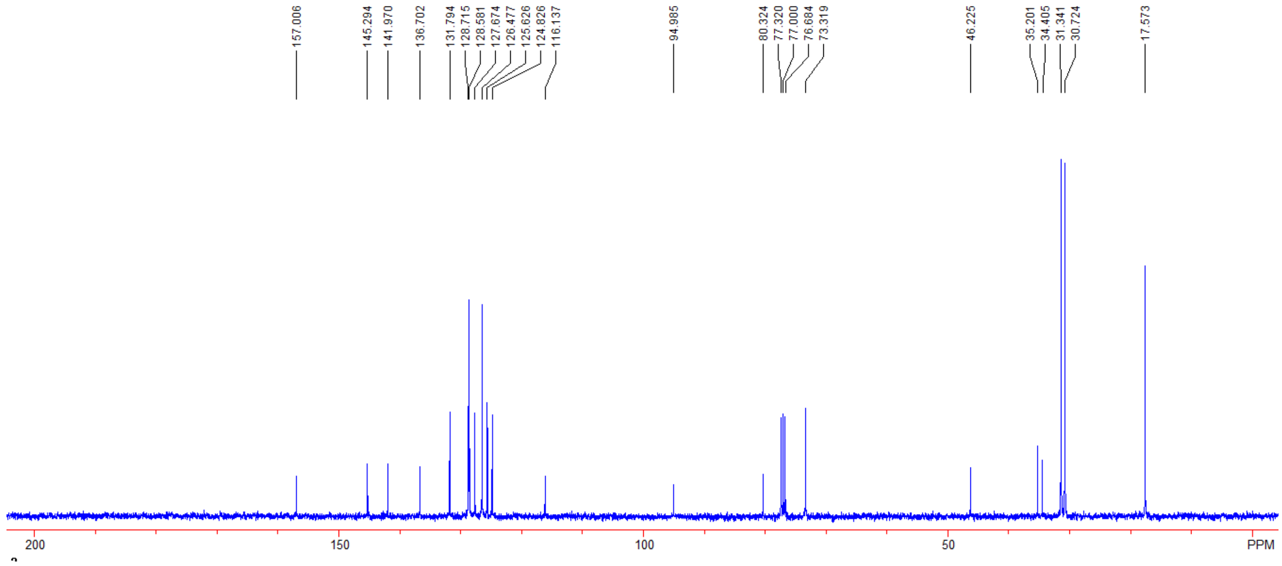

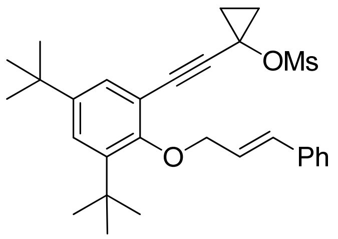

Compound **S4x**. 7.566 g, yield: 87%; yellow oil. ^{1}H NMR ($CDCl_3$, 400 MHz, TMS) δ 1.29 (s, 9H, ^{t}Bu), 1.34-1.38 (m, 2H, CH_2), 1.41 (s, 9H, ^{t}Bu), 1.63-1.66 (m, 2H, CH_2), 3.23 (s, 3H, CH_3), 4.88-4.90 (m, 2H, CH_2), 6.51-6.57 (m, 1H, =CH), 6.81-6.85 (m, 1H, =CH), 7.27-7.28 (m, 2H, Ar), 7.33-7.39 (m, 3H, Ar), 7.46-7.48 (m, 2H, Ar). ^{13}C NMR ($CDCl_3$, 100 MHz, TMS) δ 16.8, 30.7, 31.3, 34.4, 35.3, 39.9, 54.9, 73.7, 84.2, 89.7, 115.0, 125.1, 126.0, 126.5, 127.8, 128.6, 128.9, 132.3, 136.6, 142.4, 145.6, 157.5. IR (neat) ν 3024, 2960, 2869, 2225, 1436, 1358, 1237, 1201, 1173, 1159, 1119, 964, 936, 881, 804, 748 cm^{-1}. MS (%) m/e 480 (M^{+}, 0.23), 118 (11.31), 117 (100.00), 115 (19.09), 91 (10.17), 57 (19.98), 44 (15.62), 43 (11.32), 41 (10.45). HRMS (EI) calcd. for $C_{29}H_{36}O_4S$: 480.2334, Found: 480.2335.

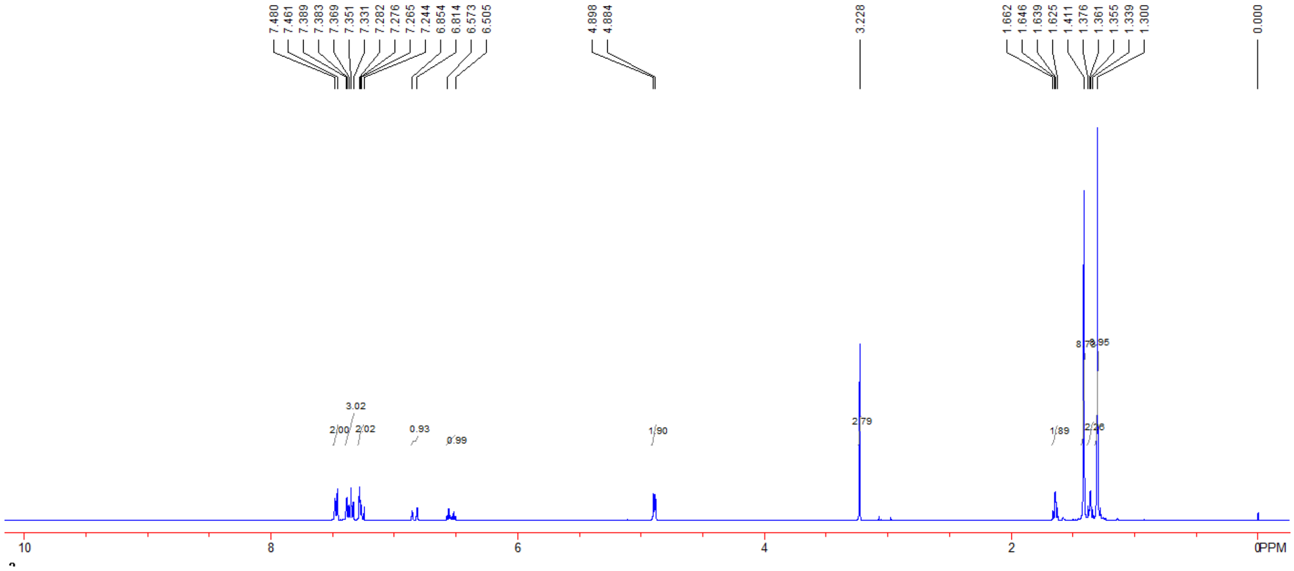

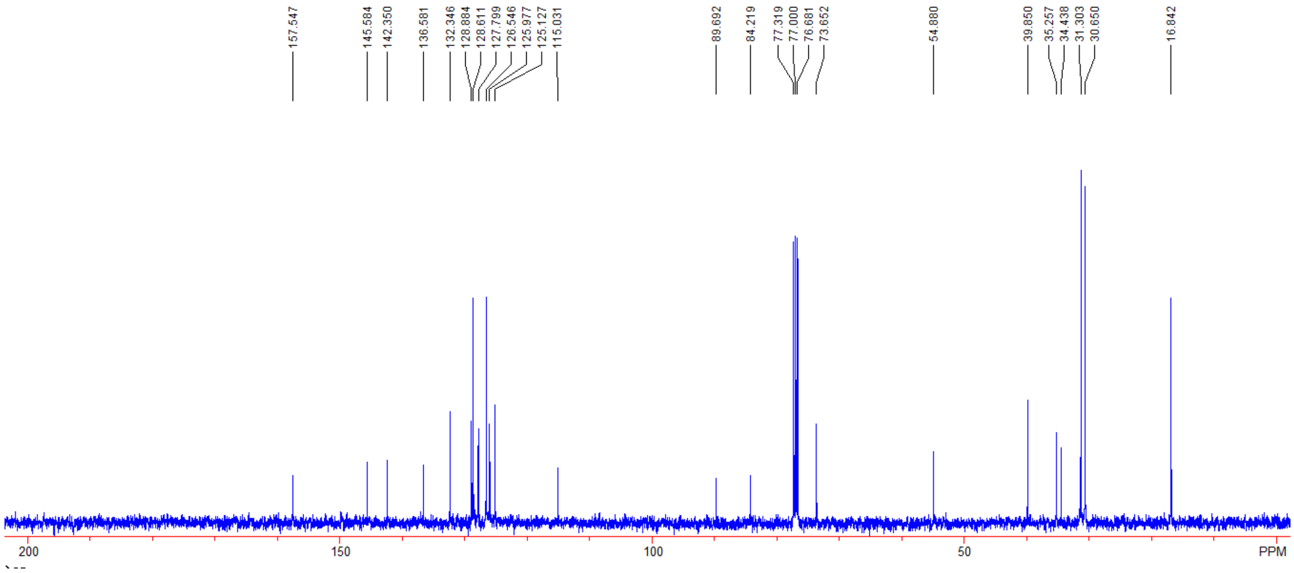

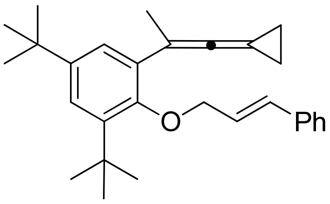

Compound **3l**. 1.003 g, yield: 83%; colorless oil. ^{1}H NMR ($CDCl_3$, 400 MHz, TMS) δ 1.31 (s, 9H, ^{t}Bu), 1.43 (m, 9H, ^{t}Bu), 1.51-1.54 (m, 4H, 2CH_2), 2.19 (s, 3H, CH_3), 4.69-4.70 (m, 2H, CH_2), 6.45-6.52 (m, 1H, =CH), 6.76-6.80 (m, 1H, =CH), 7.19-7.26 (m, 3H, Ar), 7.31-7.34 (m, 2H, Ar), 7.43-7.45 (m, 2H, Ar). ^{13}C NMR ($CDCl_3$, 100 MHz, TMS) δ 7.4, 19.8, 30.9, 31.5, 34.4, 35.3, 72.4, 76.0, 102.9, 123.1, 125.3, 126.2, 126.4, 127.5, 128.5, 131.1, 133.0, 137.0, 142.2, 145.1, 153.2, 189.6. IR (neat) ν 2958, 2868, 2014, 1703, 1598, 1476, 1433, 1361, 1222, 1129, 984, 964, 907, 730, 697 cm^{-1}. MS (%) m/e 400 (M$^+$, 11.62), 371 (20.58), 284 (26.43), 283 (100.00), 117 (82.42), 115 (29.95), 91 (28.74), 57 (63.71), 41 (19.76). HRMS (EI) calcd. for $C_{29}H_{36}O$: 400.2766, Found: 400.2763.

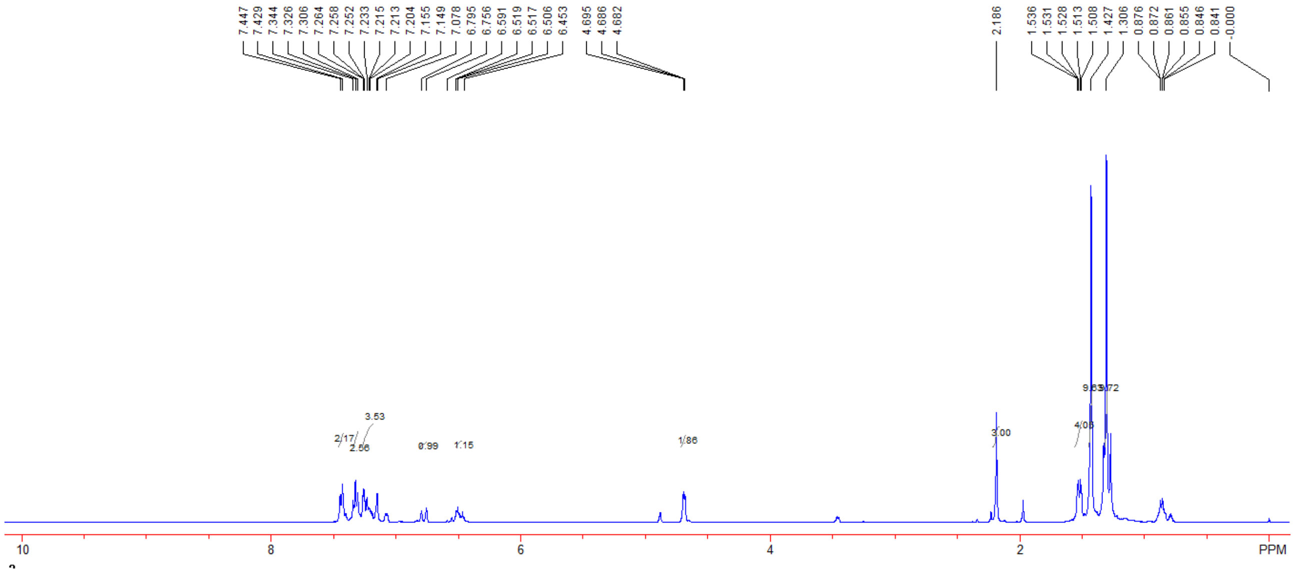

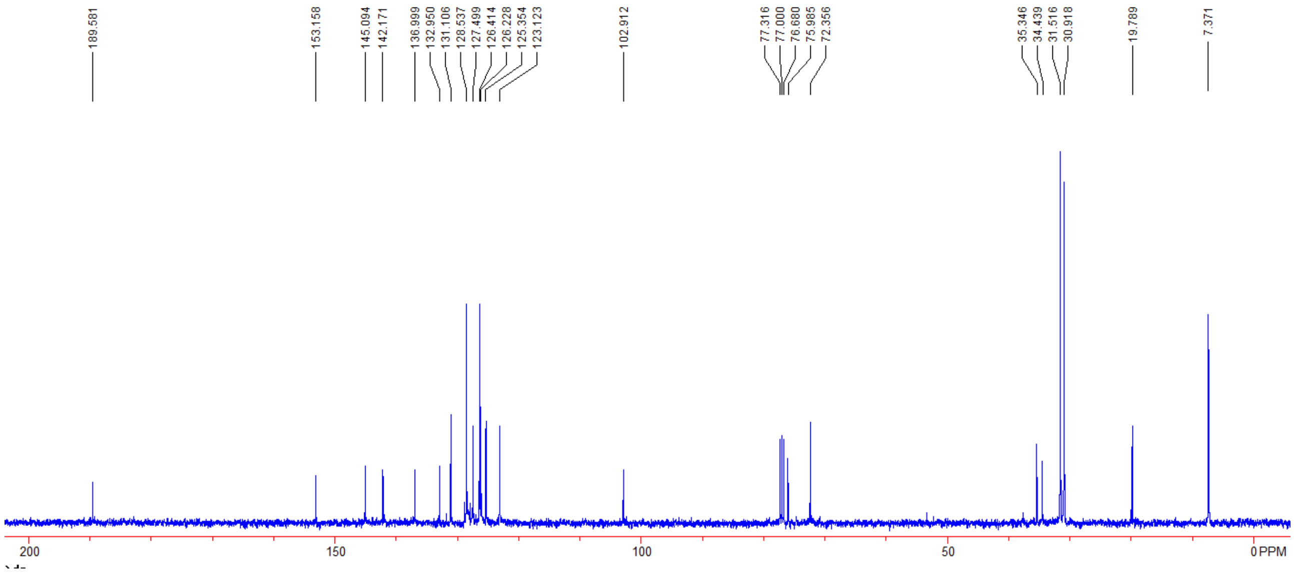

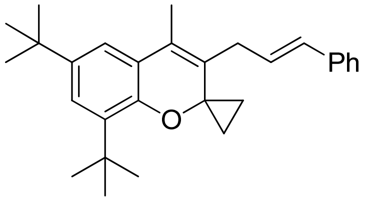

Compound **5l**. 132 mg, yield: 66%; colorless oil. ^{1}H NMR ($CDCl_3$, 400 MHz, TMS) δ 0.81-0.85 (m, 2H, CH_2), 1.20-1.21 (m, 2H, CH_2), 1.33 (s, 18H, 2^{t}Bu), 2.10 (s, 3H, CH_3), 2.88-2.89 (m, 2H, CH_2), 6.13-6.20 (m, 1H, =CH), 6.45-6.49 (m, 1H, =CH), 7.16-7.20 (m, 3H, Ar), 7.26-7.34 (m, 4H, Ar). ^{13}C NMR ($CDCl_3$, 100 MHz, TMS) δ 11.7, 14.2, 29.9, 31.1, 31.6, 34.4, 34.6, 60.4, 118.3, 122.7, 125.7, 126.0, 127.0, 127.2, 128.5, 129.3, 130.6, 135.9, 137.4, 142.7, 149.8. IR (neat) ν 2999, 2953, 2867, 2904, 1598, 1440, 1390, 1360, 1265, 1239, 1118, 965, 907, 732, 690 cm^{-1}. MS (%) m/e 400 (M^{+}, 23.24), 372 (33.19), 371 (100.00), 309 (37.49), 295 (18.87), 117 (17.23), 91 (29.41), 57

(43.19). HRMS (EI) calcd. for $C_{29}H_{36}O$: 400.2766, Found: 400.2764.

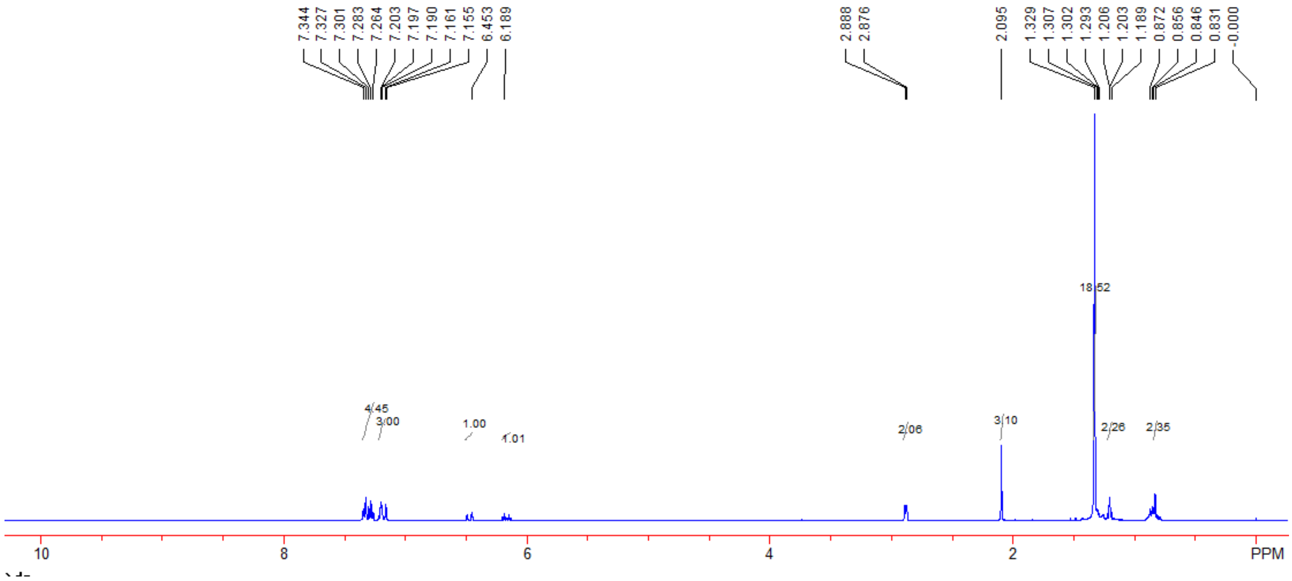

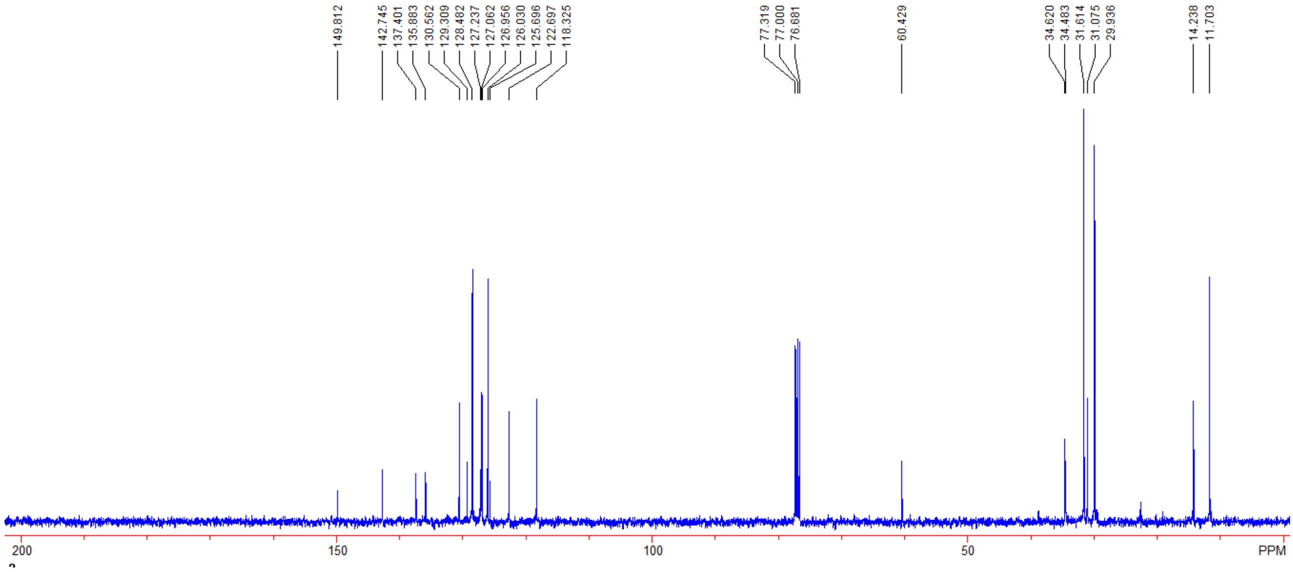

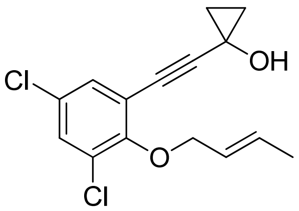

Compound **S3y**. 3.220 g, yield: 92%; yellow oil. ^{1}H NMR ($CDCl_3$, 400 MHz, TMS) δ 1.08-1.12 (m, 2H, CH_2), 1.20-1.23 (m, 2H, CH_2), 1.73 (d, J = 5.6 Hz, 3H, CH_3), 3.10 (br, 1H, OH), 4.52 (d, J = 5.2 Hz, 2H, CH_2), 5.77-5.81 (m, 2H, 2=CH), 7.23 (d, J = 2.4 Hz, 1H, Ar), 7.30 (d, J = 2.4 Hz, 1H, Ar). ^{13}C NMR ($CDCl_3$, 100 MHz, TMS) δ 17.7, 17.8, 45.8, 74.7, 76.9, 97.3, 120.2, 126.0, 128.8, 129.2, 129.8, 131.1, 131.6, 154.4. IR (neat) ν 3384, 2940, 2870, 2223, 1550, 1461, 1403, 1367, 1239, 1174, 1024, 960, 857, 731, 666 cm^{-1}. MS (%) m/e 296 (M^+, 0.94), 242 (26.66), 215 (47.73),

213 (60.75), 201 (35.06), 199 (53.41), 186 (26.85), 115 (25.46), 55 (100.00). HRMS (EI) calcd. for $C_{15}H_{14}O_2Cl_2$: 296.0371, Found: 296.0374.

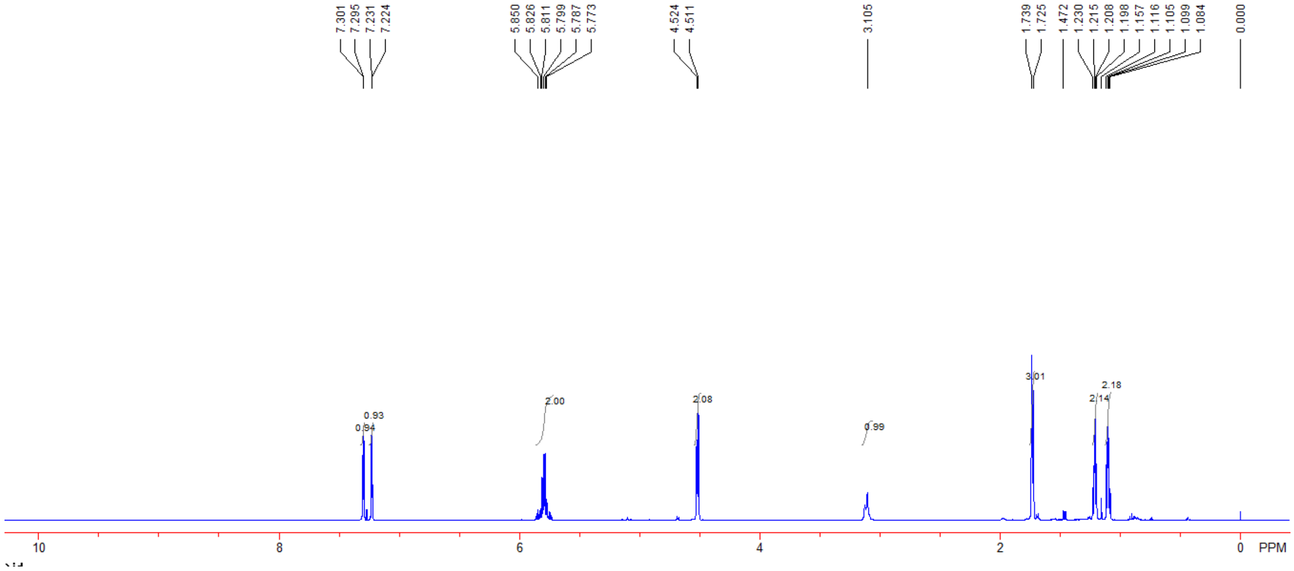

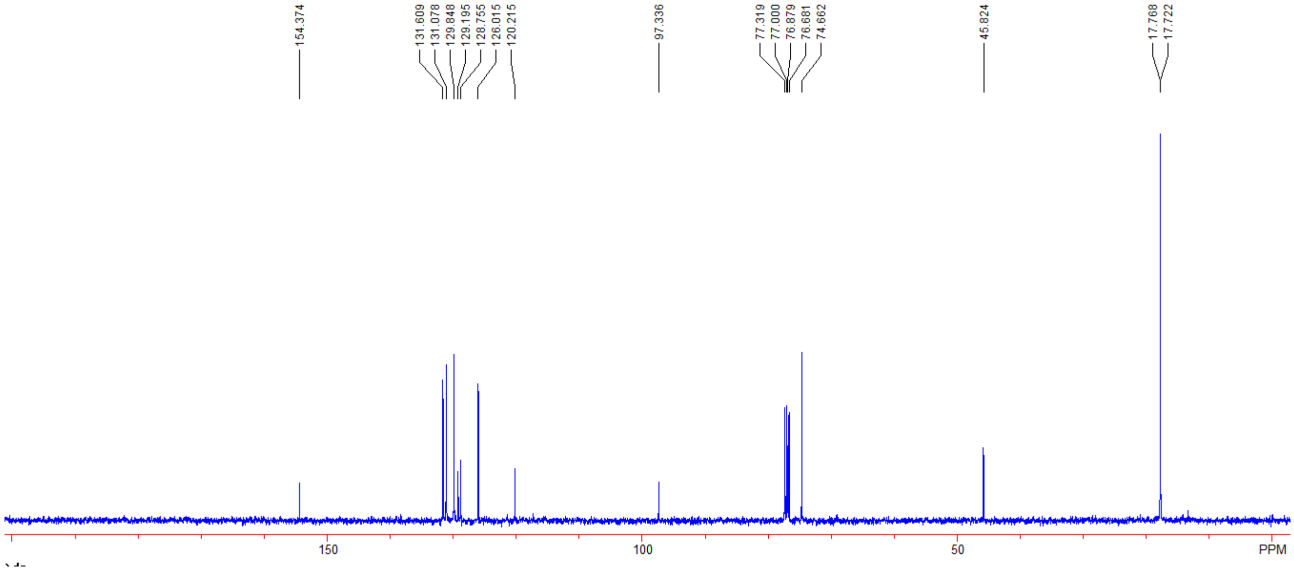

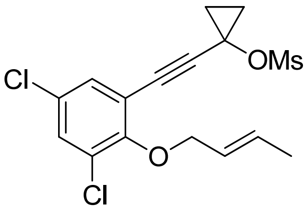

Compound **S4y**. 3.178 g, yield: 90%; yellow oil. ^{1}H NMR ($CDCl_3$, 400 MHz, TMS) δ 1.36-1.39 (m, 2H, CH_2), 1.67-1.71 (m, 2H, CH_2), 1.75 (d, J = 5.2 Hz, 3H, CH_3), 3.25 (s, 3H, CH_3), 4.53-4.54 (m, 2H, CH_2), 5.81-5.84 (m, 2H, 2=CH), 7.26 (d, J = 2.4 Hz, 1H, Ar), 7.37 (d, J = 2.4 Hz, 1H, Ar). ^{13}C NMR ($CDCl_3$, 100 MHz, TMS) δ 17.0, 17.8, 39.9, 54.0, 75.0, 80.6, 91.7, 119.2, 125.7, 129.0, 129.4, 130.8, 131.1, 132.1, 154.9. IR (neat) ν 3016, 2939, 2870, 2255, 1551, 1439, 1363, 1247, 1160, 1037,

940, 872, 802, 730, 669 cm^{-1}. MS (%) m/e 241 (51.21), 224 (44.08), 215 (44.97), 213 (66.33), 201 (59.63), 199 (92.56), 189 (58.61), 55 (100.00). HRMS (EI) calcd. for $C_{16}H_{16}O_4SCl_2$: 374.0146, Found: 374.0150.

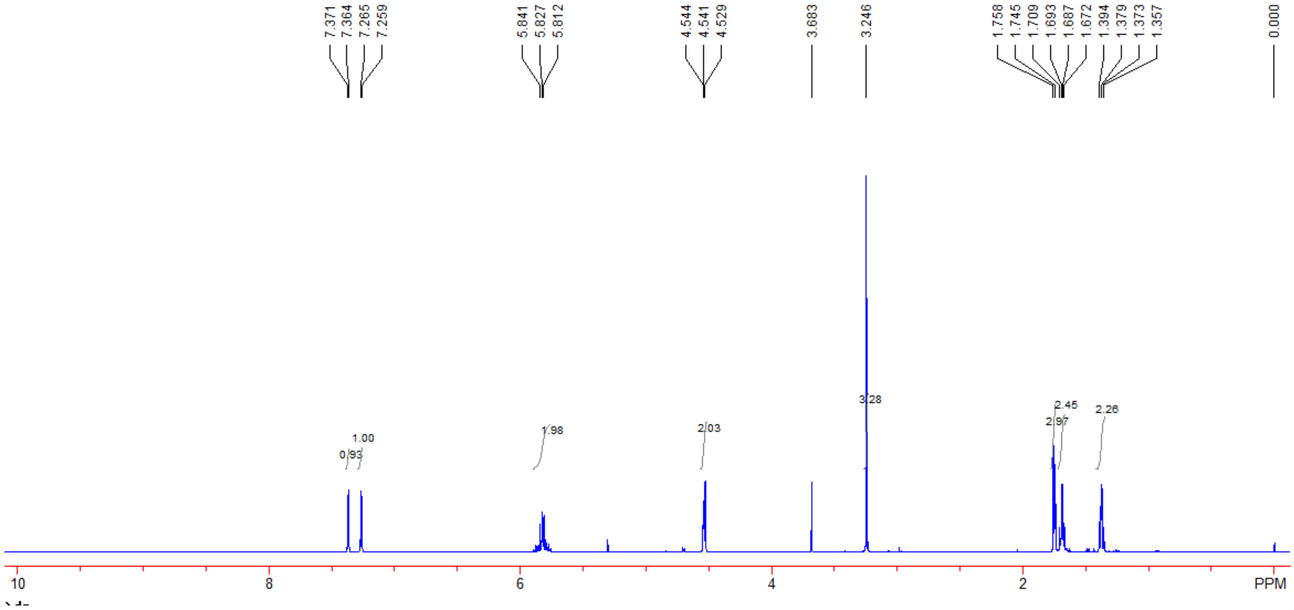

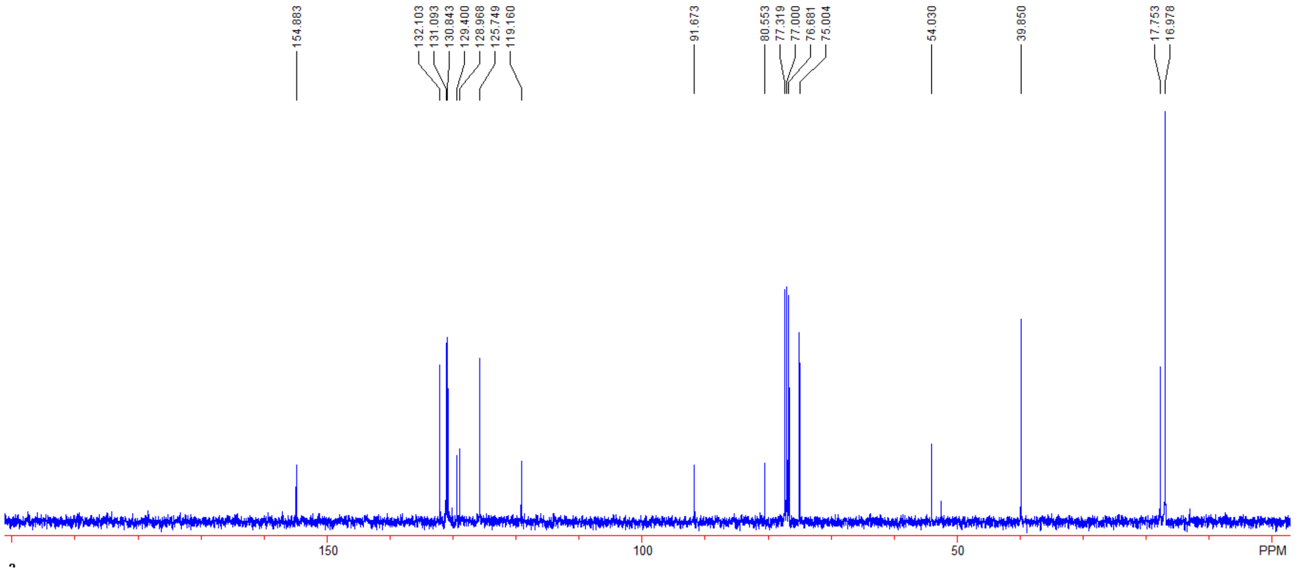

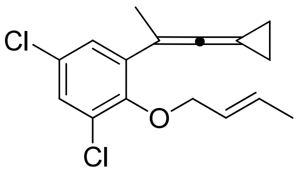

Compound **3m**. 720 g, yield: 82%; colorless oil. ^{1}H NMR ($CDCl_3$, 400 MHz, TMS) δ 1.59 (s, 4H, $2CH_2$), 1.73 (d, J = 4.4 Hz, 3H, CH_3), 2.13-2.14 (m, 3H, CH_3), 4.35-4.36 (m, 2H, CH_2), 5.71-5.81 (m, 2H, 2=CH), 7.14 (d, J = 2.4 Hz, 1H, Ar), 7.23 (d, J = 2.4 Hz, 1H, Ar). ^{13}C NMR ($CDCl_3$, 100

MHz, TMS) δ 8.0, 17.8, 19.7, 73.8, 76.3, 98.8, 126.3, 127.2, 127.8, 128.9, 129.5, 130.8, 136.5, 150.9, 190.8. IR (neat) ν 3016, 2983, 2850, 2007, 1575, 1556, 1440, 1366, 1246, 1222, 1182, 1071, 963, 852, 825, 731 cm^{-1}. MS (%) m/e 294 (M^+, 1.5), 241 (67.06), 239 (100.00), 204 (50.88), 203 (19.62), 169 (54.15), 141 (21.90), 139 (27.27), 55 (47.13). HRMS (EI) calcd. for $C_{16}H_{16}OCl_2$: 294.0578, Found: 294.0576.

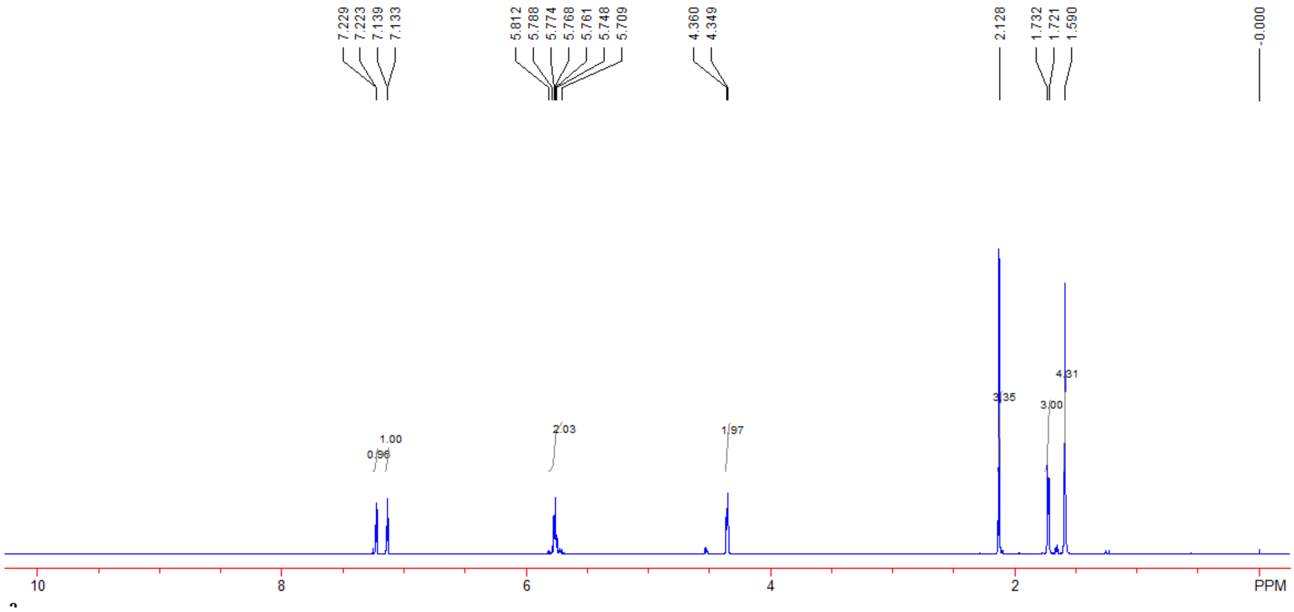

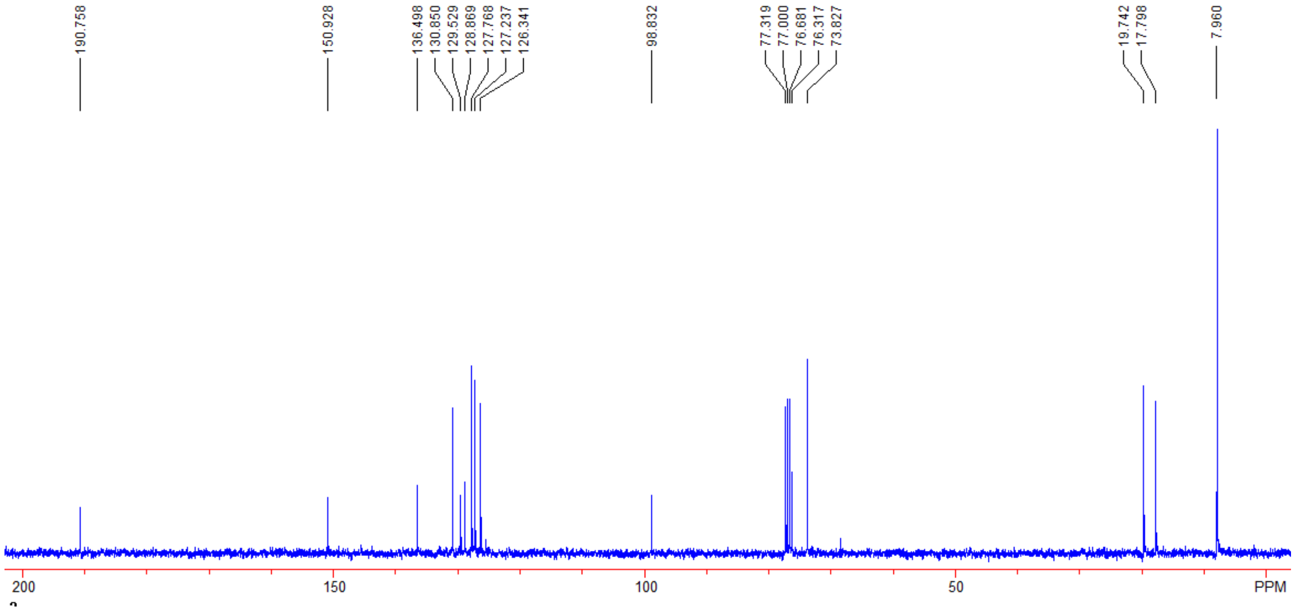

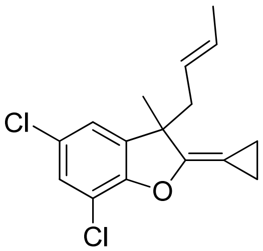

Compound **4m**. 22 mg, yield: 15%; colorless oil. ^{1}H NMR ($CDCl_3$, 400 MHz, TMS) δ 1.34 (s, 3H, CH_3), 1.56-1.58 (m, 3H, CH_3), 2.29-2.33 (m, 2H, CH_2), 2.38-2.44 (m, 2H, CH_2), 2.85-2.93 (m, 2H, CH_2), 5.15-5.23 (m, 1H, =CH), 5.31-5.39 (m, 1H, =CH), 7.09 (d, J = 2.4 Hz, 1H, Ar), 7.22 (d, J = 2.4 Hz, 1H, Ar). ^{13}C NMR ($CDCl_3$, 100 MHz, TMS) δ 18.0, 23.4, 28.3, 31.2, 38.1, 45.9, 117.1, 122.8, 126.2, 126.8, 127.5, 127.6, 128.3, 133.3, 141.9, 147.3. IR (neat) ν 3024, 2961, 2928, 1742, 1552, 1434, 1347, 1290, 1222, 1176, 1073, 964, 859, 826, 761 cm^{-1}. MS (%) m/e 294 (M^+, 0.77), 243 (11.12), 242 (9.14), 241 (63.79), 240 (14.87), 239 (100.00), 204 (7.21), 169 (11.76), 139 (7.85). HRMS (EI) calcd. for $C_{16}H_{16}OCl_2$: 294.0578, Found: 294.0580.

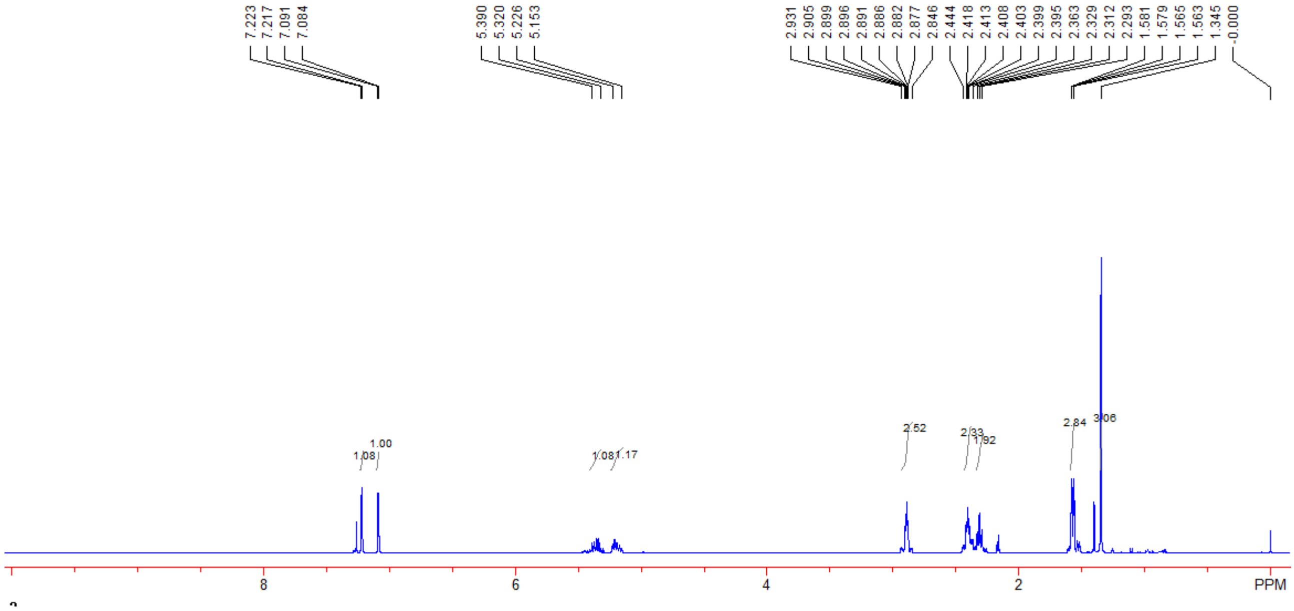

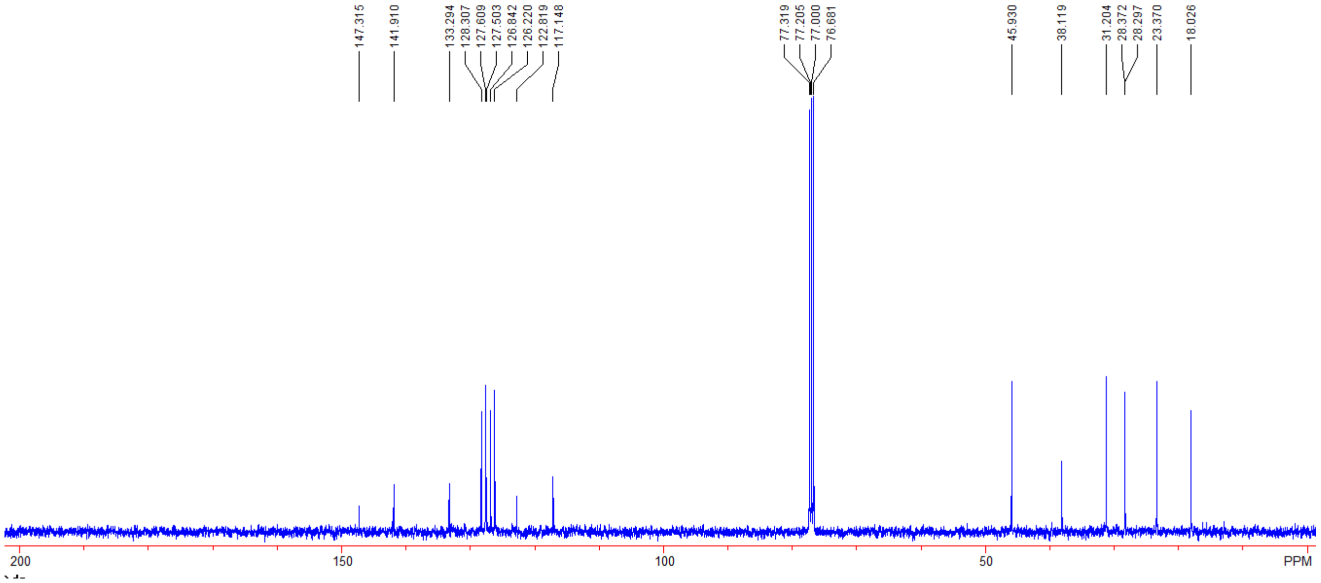

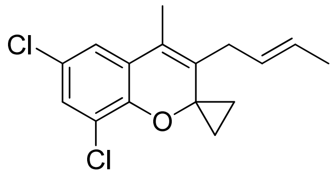

Compound **5m**. 113 mg, yield: 77%; colorless oil. ^{1}H NMR ($CDCl_3$, 400 MHz, TMS) δ 0.86-0.89 (m, 2H, CH_2), 1.15-1.19 (m, 2H, CH_2), 1.65-1.67 (m, 3H, CH_3), 1.98 (s, 3H, CH_3), 2.61 (d, J = 5.6 Hz, 2H, CH_2), 5.32-5.39 (m, 1H, =CH), 5.49-5.58 (m, 1H, =CH), 7.04 (d, J = 2.4 Hz, 1H, Ar), 7.13 (d, J = 2.4 Hz, 1H, Ar). ^{13}C NMR ($CDCl_3$, 100 MHz, TMS) δ 12.5, 13.6, 17.9, 30.8, 61.7, 121.3, 121.6, 124.7, 126.2, 126.5, 126.7, 127.5, 128.8, 133.5, 148.1. IR (neat) ν 3016, 2917, 2850, 1560, 1455, 1379, 1257, 1208, 1135, 1011, 965, 943, 906, 853, 731 cm^{-1}. MS (%) m/e 294 (M$^+$, 6.37), 281 (11.82), 279 (19.54), 267 (65.42), 266 (20.27), 265 (100.00), 251 (17.19), 230 (23.96), 216 (14.00). HRMS (EI) calcd. for $C_{16}H_{16}OCl_2$: 294.0578, Found: 294.0583.

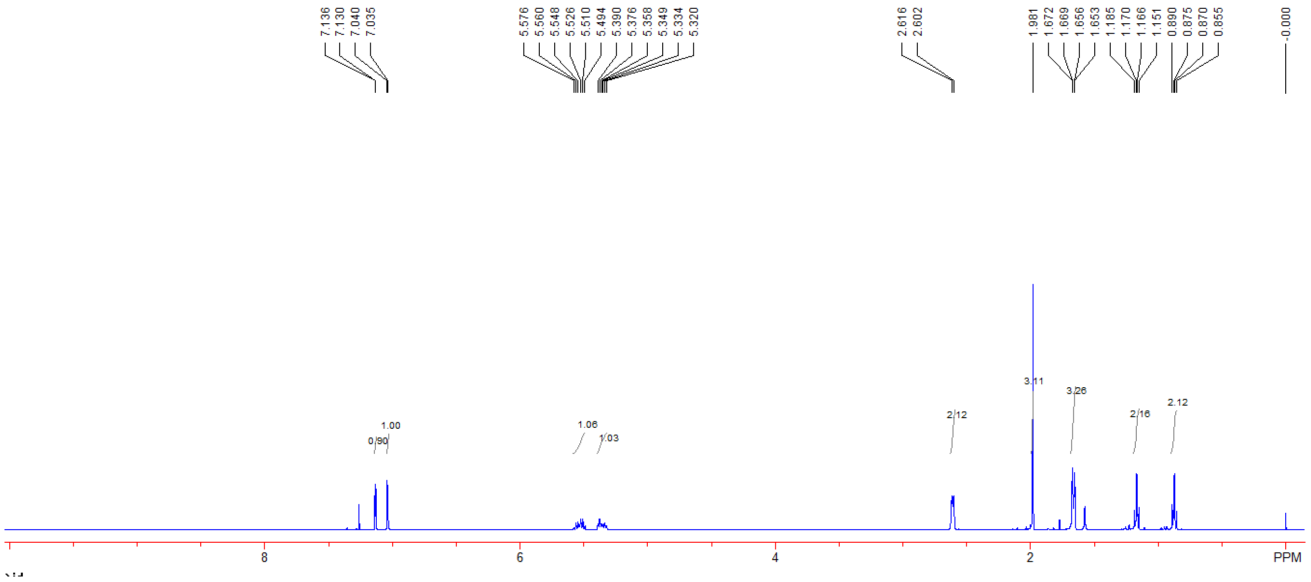

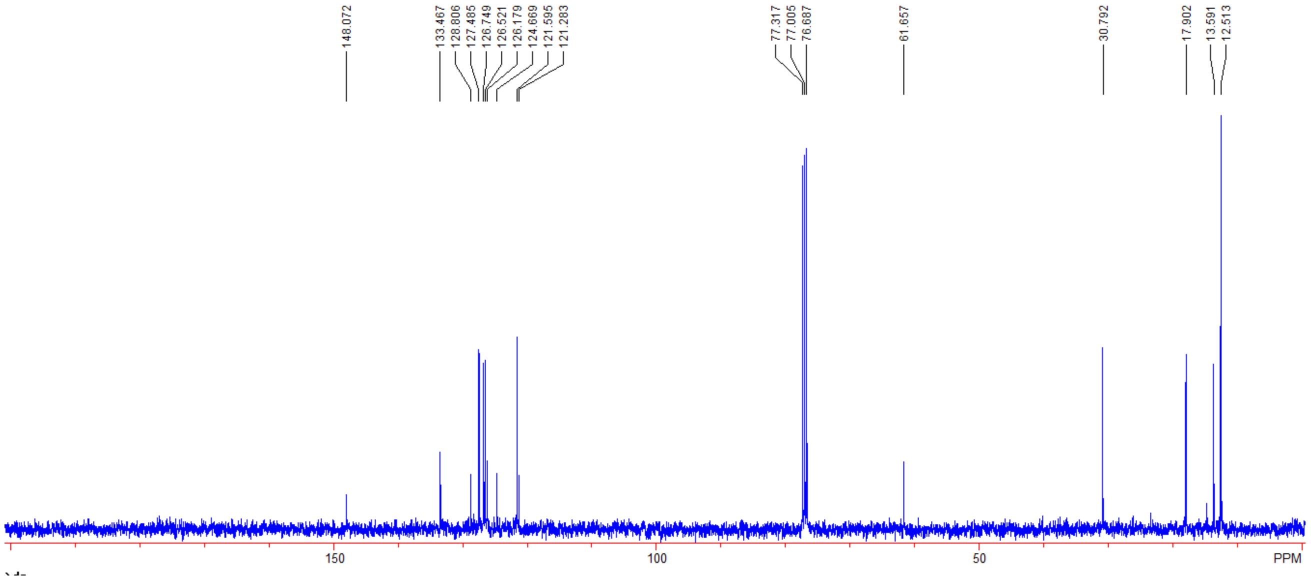

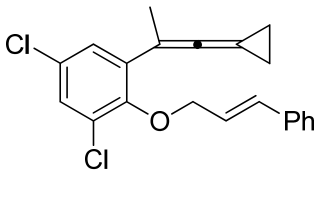

Compound **3n**. 875 mg, yield: 82%; colorless oil. ^{1}H NMR ($CDCl_3$, 400 MHz, TMS) δ 1.58 (s, 4H, $2CH_2$), 2.15 (s, 3H, CH_3), 4.59-4.60 (m, 2H, CH_2), 6.43 (dt, J_1 = 6.4 Hz, J_2 = 15.6 Hz, 1H, =CH), 6.64 (d, J_1 = 15.6 Hz, 1H, =CH), 7.15-7.16 (m, 1H, Ar), 7.23-7.26 (m, 2H, Ar), 7.30-7.33 (m, 2H, Ar), 7.38-7.40 (m, 2H, Ar). ^{13}C NMR ($CDCl_3$, 100 MHz, TMS) δ 8.1, 19.8, 73.6, 76.4, 98.7, 124.5, 126.5, 127.2, 127.9, 128.6, 129.1, 129.5, 133.1, 136.4, 136.5, 150.8, 190.9. IR (neat) ν 3060, 2984, 2912, 2005, 1575, 1556, 1493, 1441, 1368, 1225, 1065, 963, 825, 747, 690 cm^{-1}. MS (%) m/e 356 (M^{+}, 0.86), 241 (23.53), 239 (37.23), 204 (14.30), 169 (11.62), 118 (10.45), 117 (100.00), 115

(34.34), 91 (12.88). HRMS (EI) calcd. for $C_{21}H_{18}OCl_2$: 356.0732, Found: 356.0735.

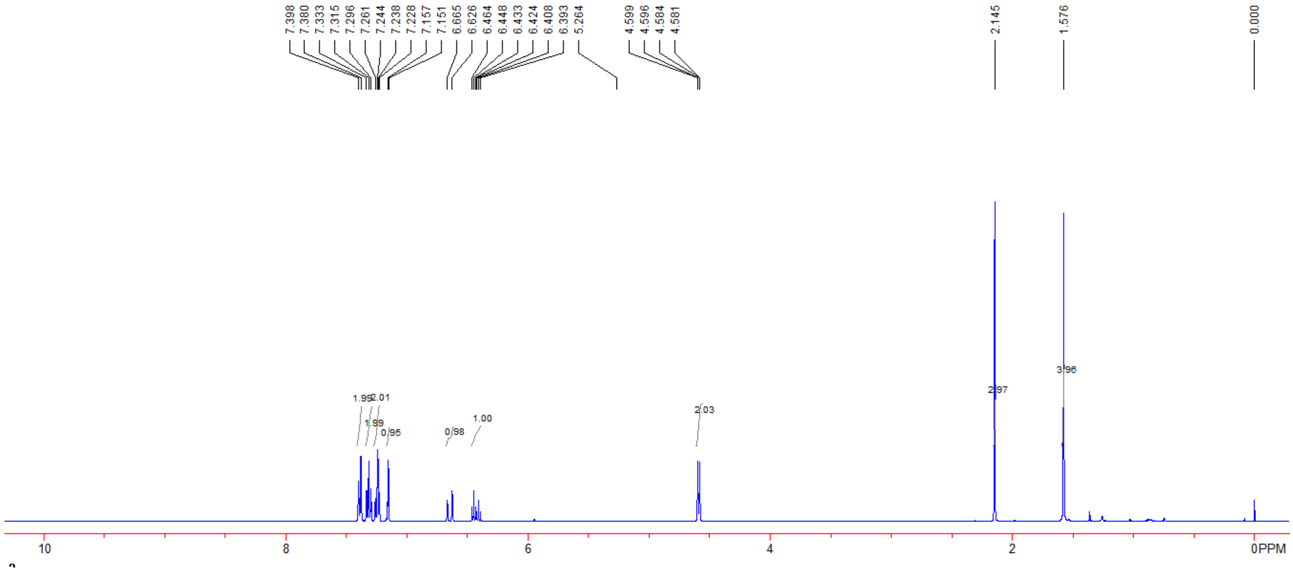

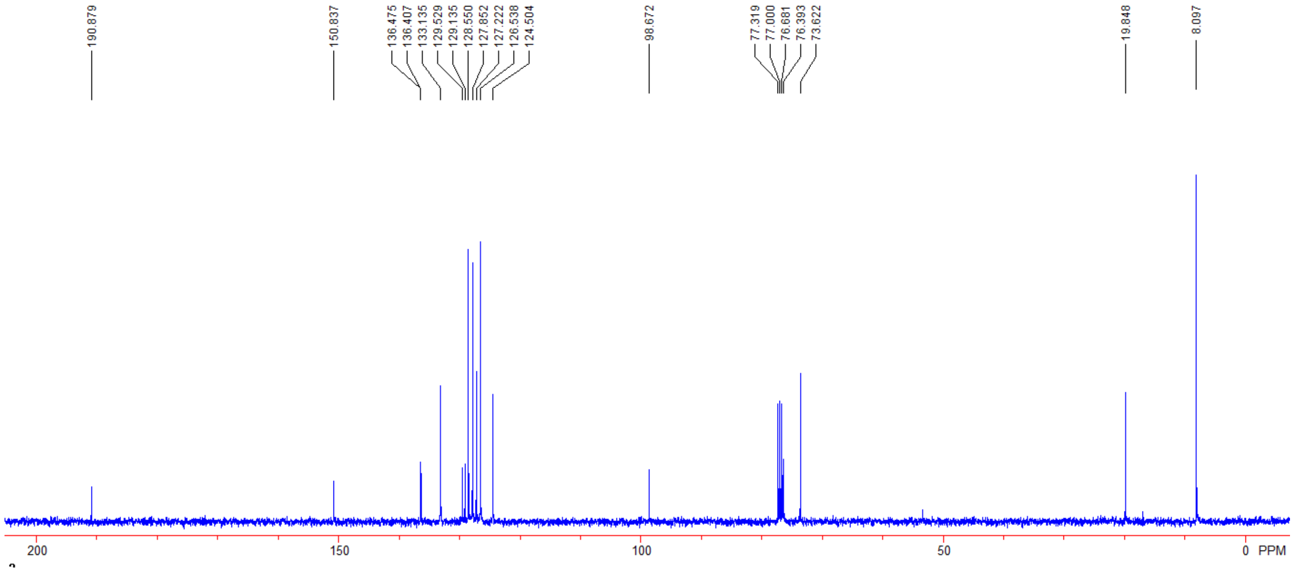

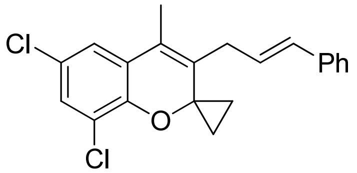

Compound **5n**. 128 mg, yield: 72%; colorless oil. ^{1}H NMR ($CDCl_3$, 400 MHz, TMS) δ 0.90-0.94 (m, 2H, CH_2), 1.19-1.22 (m, 2H, CH_2), 2.04 (s, 3H, CH_3), 2.84-2.86 (m, 2H, CH_2), 6.13 (dt, J_1 = 6.4 Hz, J_2 = 15.6 Hz, 1H, =CH), 6.45 (d, J_1 = 15.6 Hz, 1H, =CH), 7.06-7.07 (m, 1H, Ar), 7.15-7.16 (m, 1H, Ar), 7.19-7.23 (m, 1H, Ar), 7.28-7.35 (m, 4H, Ar). ^{13}C NMR ($CDCl_3$, 100 MHz, TMS) δ 12.6, 13.8, 31.1, 61.7, 121.4, 121.7, 125.5, 125.8, 126.1, 126.3, 127.3, 127.7, 128.5, 128.7, 131.2, 132.6, 137.1, 148.2. IR (neat) ν 3080, 3024, 2923, 2851, 1560, 1495, 1456, 1405, 1298, 1258, 1134, 965,

853, 768, 734, 690 cm^{-1}. MS (%) m/e 356 (M^+, 19.27), 329 (67.60), 328 (23.97), 327 (100.00), 292 (23.14), 267 (23.04), 265 (42.99), 251 (20.11). HRMS (EI) calcd. for $C_{21}H_{18}OCl_2$: 356.0735, Found: 356.0734.

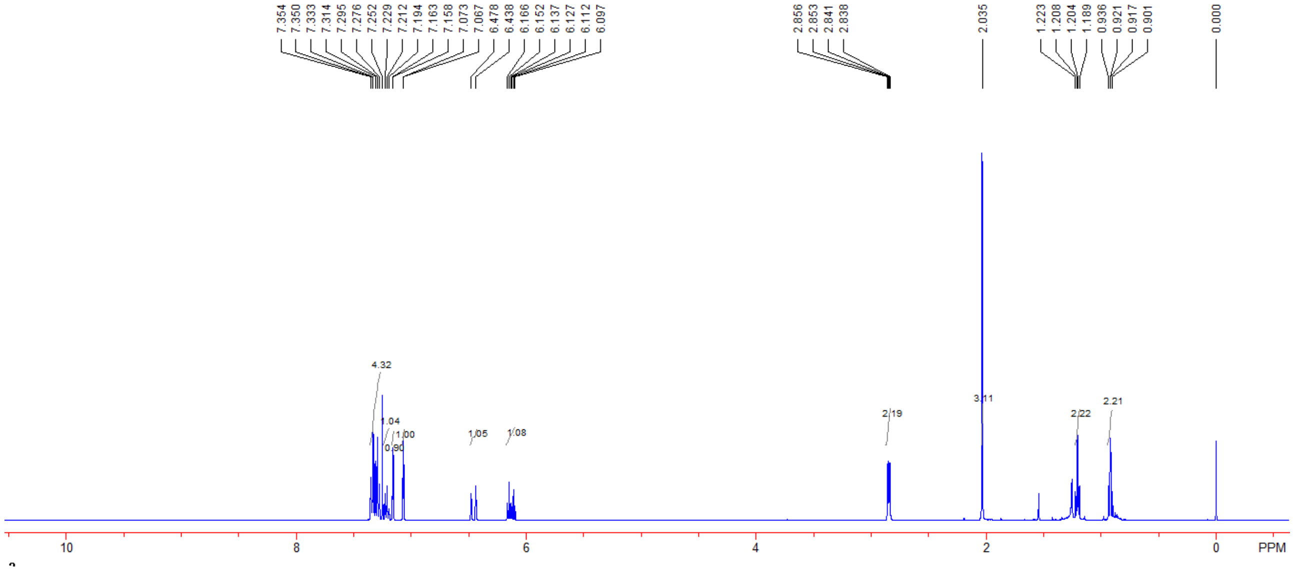

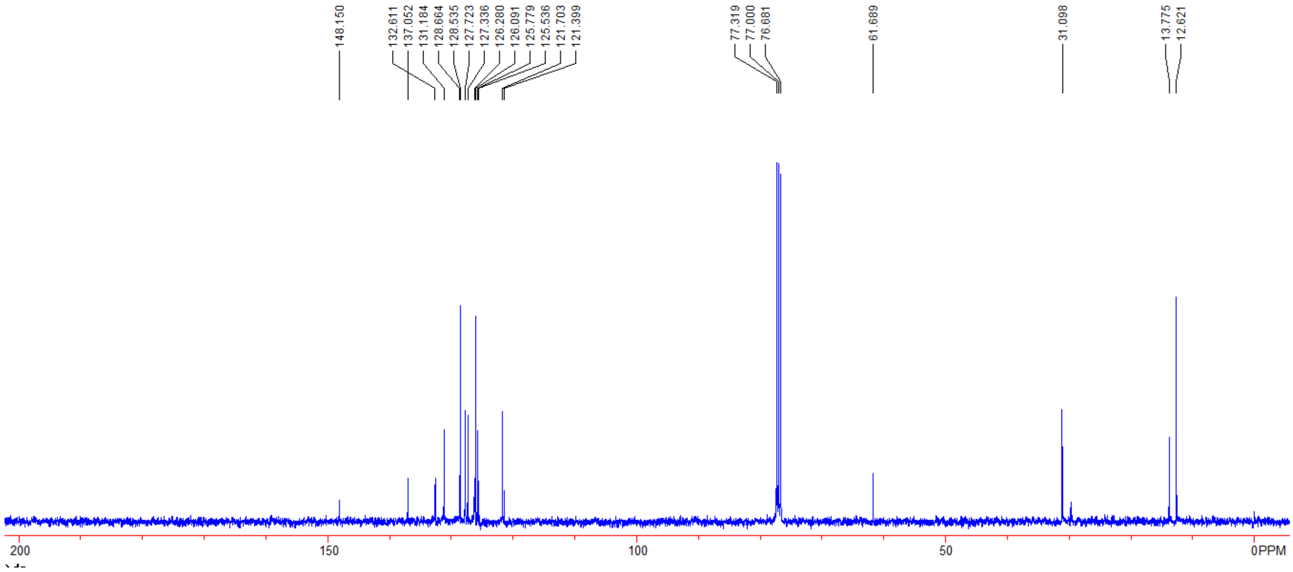

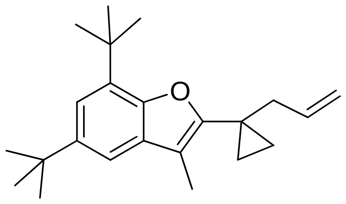

Compound **6**. 266 mg, yield: 82%; colorless oil. ^{1}H NMR ($CDCl_3$, 400 MHz, TMS) δ 0.79-0.82 (m, 2H, CH_2), 0.934-0.97 (m, 2H, CH_2), 1.37 (s, 9H, ^{t}Bu), 1.49 (s, 9H, ^{t}Bu), 2.21 (s, 3H, CH_3), 2.35-2.37 (m, 2H, CH_2), 4.93-5.00 (m, 2H, =CH_2), 5.80-5.90 (m, 2H, =CH_2), 7.189-7.193 (m, 1H, Ar), 7.25-7.26 (m, 2H, Ar). ^{13}C NMR ($CDCl_3$, 100 MHz, TMS) δ 8.27, 11.8, 18.1, 29.9, 32.0, 34.4,

34.8, 42.4, 110.8, 112.7, 116.2, 118.1, 130.3, 133.2, 135.9, 144.7, 149.8, 155.0. IR (neat) ν 3079, 2954, 2904, 2867, 1599, 1480, 1459, 1417, 1361, 1243, 1202, 1141, 1105, 1022, 995, 911, 863, 832, 767, 657 cm^{-1}. MS (%) m/e 324 (M^+, 60.22), 309 (100.00), 310 (25.50), 325 (16.51), 295 (10.03), 283 (10.96), 253 (7.70), 57 (21.38). HRMS (EI) calcd. for $C_{23}H_{32}O$: 324.2453, Found: 324.2452.

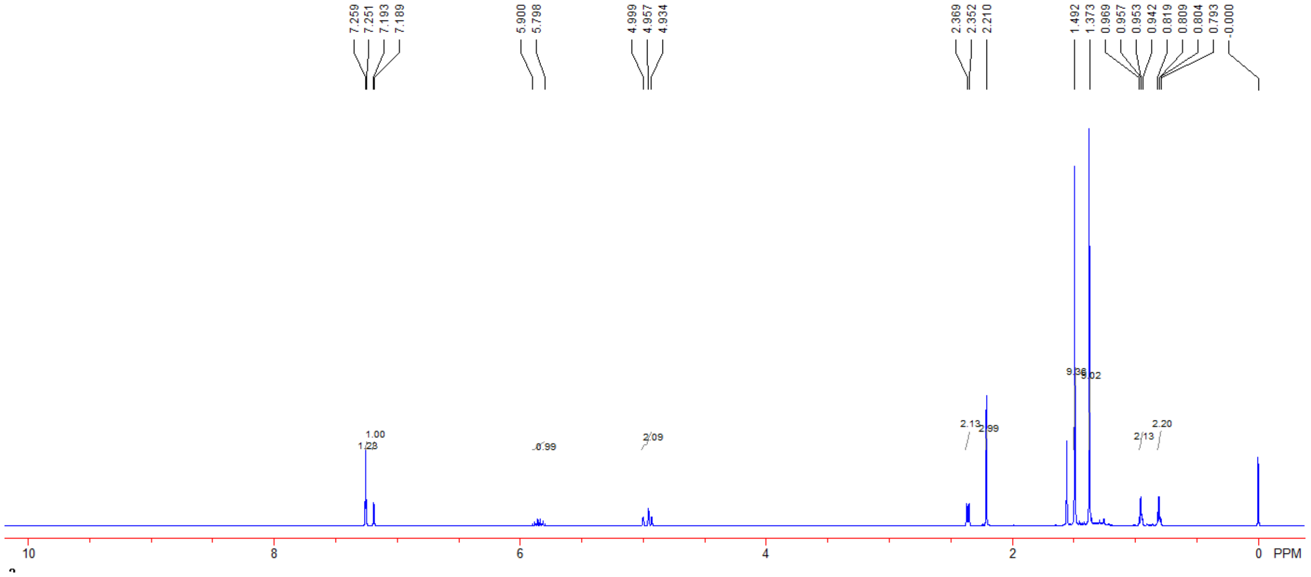

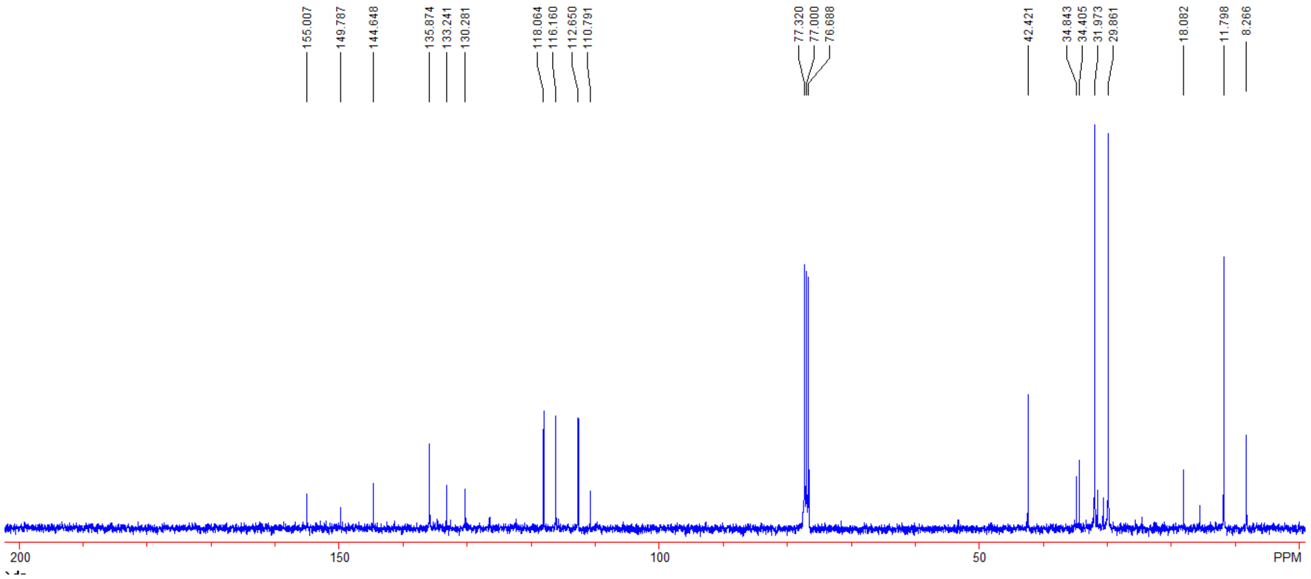

LDY-6-85-BR-2D

Sample Name:
LDY-6-85-BR-2D
Data Collected on:
OMC-NMR600-vnmrs600
Archive directory:
/home/omc/vnmrsys/data
Sample directory:
LDY-6-85-BR-2D_20141211_01
FidFile: NOESY_01

Pulse Sequence: NOESY
Solvent: cdcl3
Data collected on: Dec 11 2014

Temp. 25.0 C / 298.1 K
Operator: omc

Relax. delay 1.500 sec
Acq. time 0.271 sec
Width 5681.8 Hz
2D Width 5681.8 Hz
4 repetitions
2 x 128 increments
OBSERVE H1, 599.7754542 MHz
DATA PROCESSING
Line broadening 3.0 Hz
Gauss apodization 0.041 sec
F1 DATA PROCESSING
Gauss apodization 0.012 sec
FT size 4096 x 4096
Total time 38 min

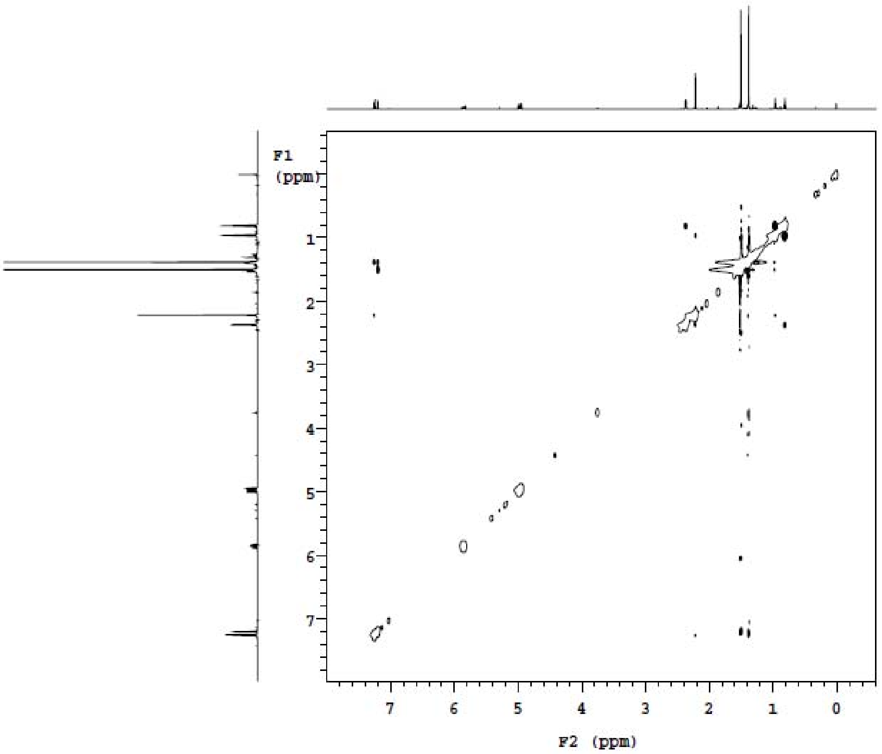

Plotname: --Not assigned--

LDY-6-85-BR-2D

Sample Name:
LDY-6-85-BR-2D
Data Collected on:
OMC-NMR600-vnmrs600
Archive directory:
/home/omc/vnmrsys/data
Sample directory:
LDY-6-85-BR-2D_20141210_01
FidFile: gCOSY_01

Pulse Sequence: gCOSY
Solvent: cdcl3
Data collected on: Dec 10 2014

Temp. 25.0 C / 298.1 K
Operator: omc

Relax. delay 1.000 sec
Acq. time 0.247 sec
Width 6218.9 Hz
2D Width 6218.9 Hz
2 repetitions
128 increments
OBSERVE H1, 599.7754542 MHz
DATA PROCESSING
Sq. sine bell 0.080 sec
F1 DATA PROCESSING
Sq. sine bell 0.021 sec
FT size 4096 x 4096
Total time 5 min 11 sec

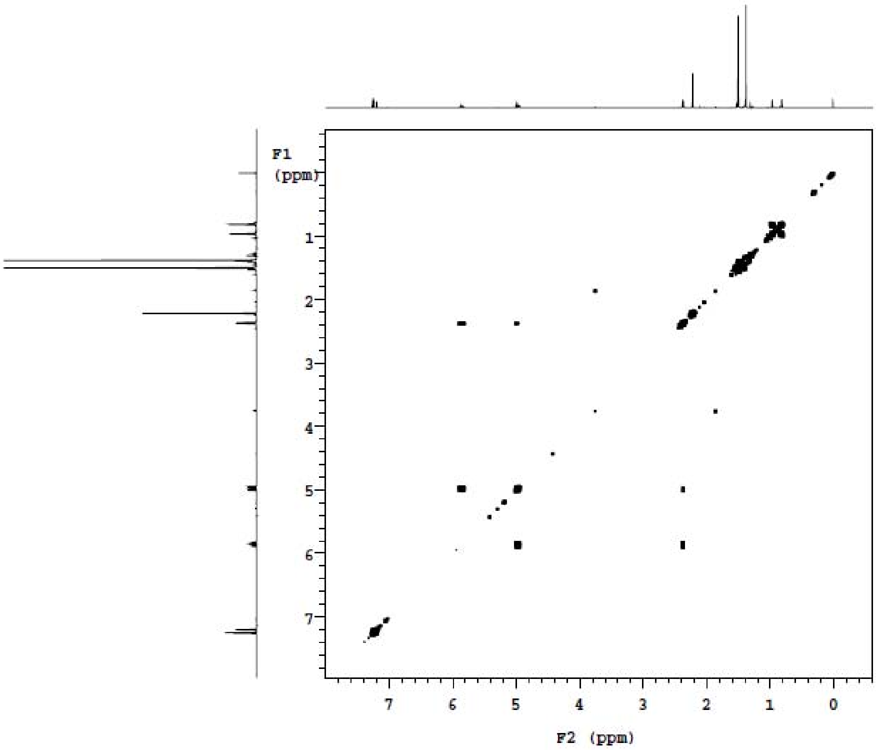

Plotname: --Not assigned--

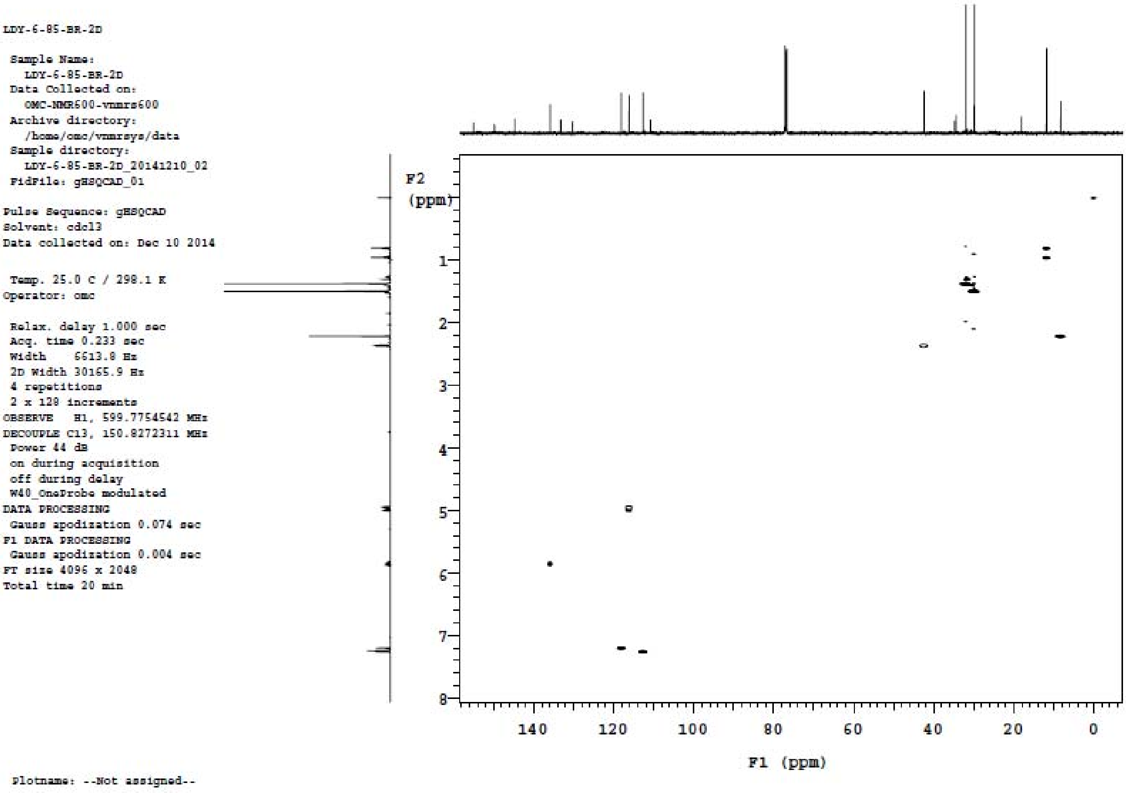
LDY-6-85-BR-2D
Sample Name:
LDY-6-85-BR-2D
Data Collected on:
OMC-NMR600-vnmrs600
Archive directory:
/home/omc/vnmrsys/data
Sample directory:
LDY-6-85-BR-2D_20141210_02
FidFile: gHSQCAD_01
Pulse Sequence: gHSQCAD
Solvent: cdcl3
Data collected on: Dec 10 2014
Temp. 25.0 C / 298.1 K
Operator: omc
Relax. delay 1.000 sec
Acq. time 0.233 sec
Width 6613.8 Hz
2D Width 30165.9 Hz
4 repetitions
2 x 128 increments
OBSERVE H1, 599.7754542 MHz
DECOUPLE C13, 150.8272311 MHz
Power 44 dB
on during acquisition
off during delay
W40_OneProbe modulated
DATA PROCESSING
Gauss apodization 0.074 sec
F1 DATA PROCESSING
Gauss apodization 0.004 sec
FT size 4096 x 2048
Total time 20 min
Plotname: --Not assigned--
F2 (ppm)
F1 (ppm)

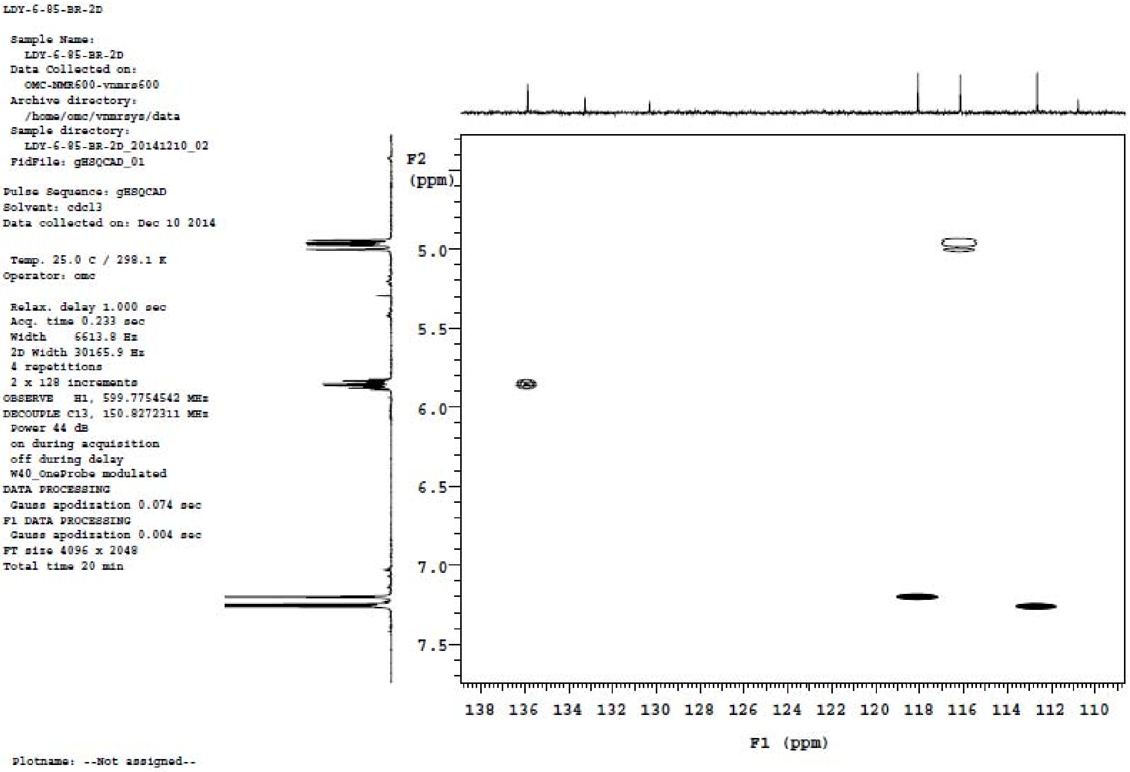
LDY-6-85-BR-2D
Sample Name:
LDY-6-85-BR-2D
Data Collected on:
OMC-NMR600-vnmrs600
Archive directory:
/home/omc/vnmrsys/data
Sample directory:
LDY-6-85-BR-2D_20141210_02
FidFile: gHSQCAD_01
Pulse Sequence: gHSQCAD
Solvent: cdcl3
Data collected on: Dec 10 2014
Temp. 25.0 C / 298.1 K
Operator: omc
Relax. delay 1.000 sec
Acq. time 0.233 sec
Width 6613.8 Hz
2D Width 30165.9 Hz
4 repetitions
2 x 128 increments
OBSERVE H1, 599.7754542 MHz
DECOUPLE C13, 150.8272311 MHz
Power 44 dB
on during acquisition
off during delay
W40_OneProbe modulated
DATA PROCESSING
Gauss apodization 0.074 sec
F1 DATA PROCESSING
Gauss apodization 0.004 sec
FT size 4096 x 2048
Total time 20 min
Plotname: --Not assigned--
F2 (ppm)
F1 (ppm)

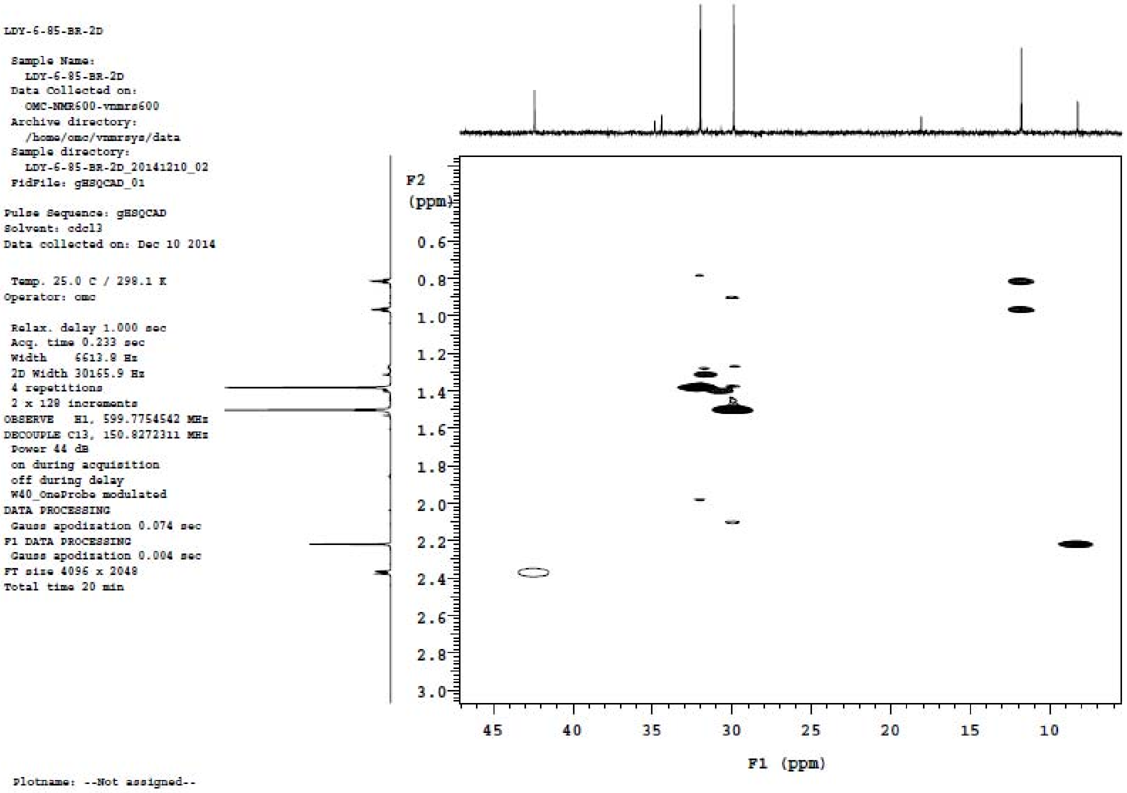
LDY-6-85-BR-2D
Sample Name:
LDY-6-85-BR-2D
Data Collected on:
OMC-NMR600-vnmrs600
Archive directory:
/home/omc/vnmrsys/data
Sample directory:
LDY-6-85-BR-2D_20141210_02
FidFile: gHSQCAD_01
Pulse Sequence: gHSQCAD
Solvent: cdcl3
Data collected on: Dec 10 2014
Temp. 25.0 C / 298.1 K
Operator: omc
Relax. delay 1.000 sec
Acq. time 0.233 sec
Width 6613.8 Hz
2D Width 30165.9 Hz
4 repetitions
2 x 128 increments
OBSERVE H1, 599.7754542 MHz
DECOUPLE C13, 150.8272311 MHz
Power 44 dB
on during acquisition
off during delay
W40_OneProbe modulated
DATA PROCESSING
Gauss apodization 0.074 sec
F1 DATA PROCESSING
Gauss apodization 0.004 sec
FT size 4096 x 2048
Total time 20 min
F2 (ppm)
F1 (ppm)
Plotname: --Not assigned--

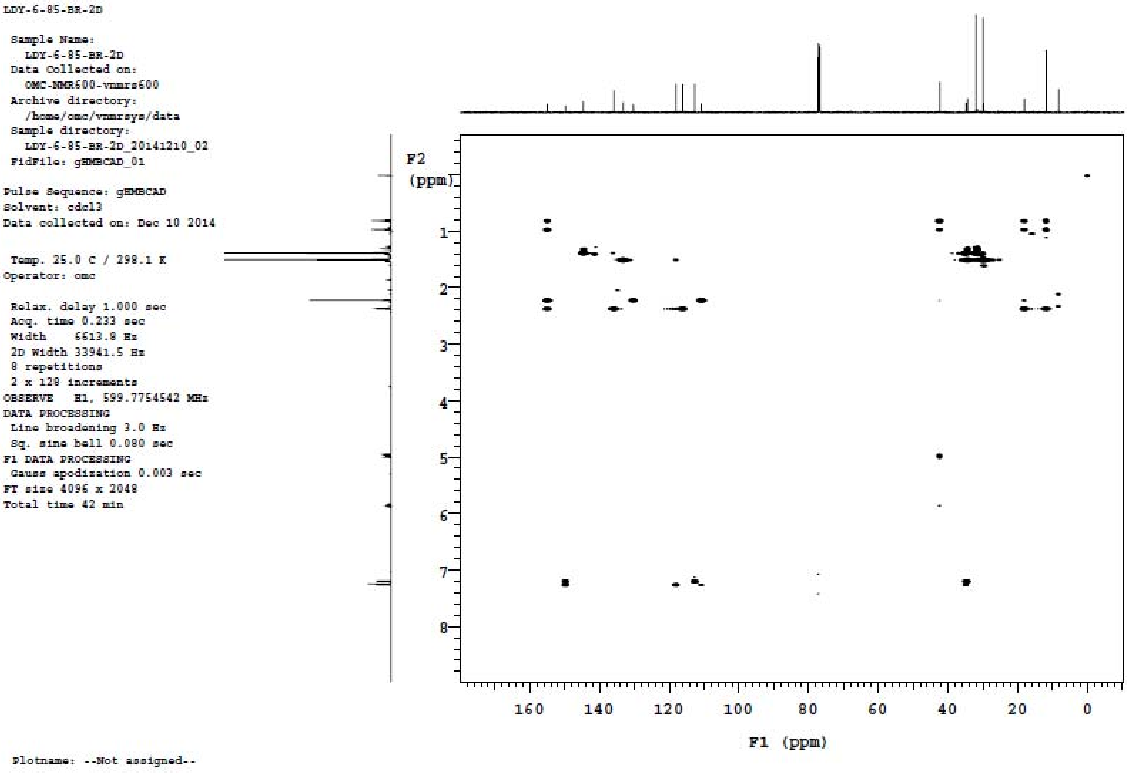
LDY-6-85-BR-2D
Sample Name:
LDY-6-85-BR-2D
Data Collected on:
OMC-NMR600-vnmrs600
Archive directory:
/home/omc/vnmrsys/data
Sample directory:
LDY-6-85-BR-2D_20141210_02
FidFile: gHMBCAD_01
Pulse Sequence: gHMBCAD
Solvent: cdcl3
Data collected on: Dec 10 2014
Temp. 25.0 C / 298.1 K
Operator: omc
Relax. delay 1.000 sec
Acq. time 0.233 sec
Width 6613.8 Hz
2D Width 33941.5 Hz
8 repetitions
2 x 128 increments
OBSERVE H1, 599.7754542 MHz
DATA PROCESSING
Line broadening 3.0 Hz
Sq. sine bell 0.080 sec
F1 DATA PROCESSING
Gauss apodization 0.003 sec
FT size 4096 x 2048
Total time 42 min
F2 (ppm)
F1 (ppm)
Plotname: --Not assigned--

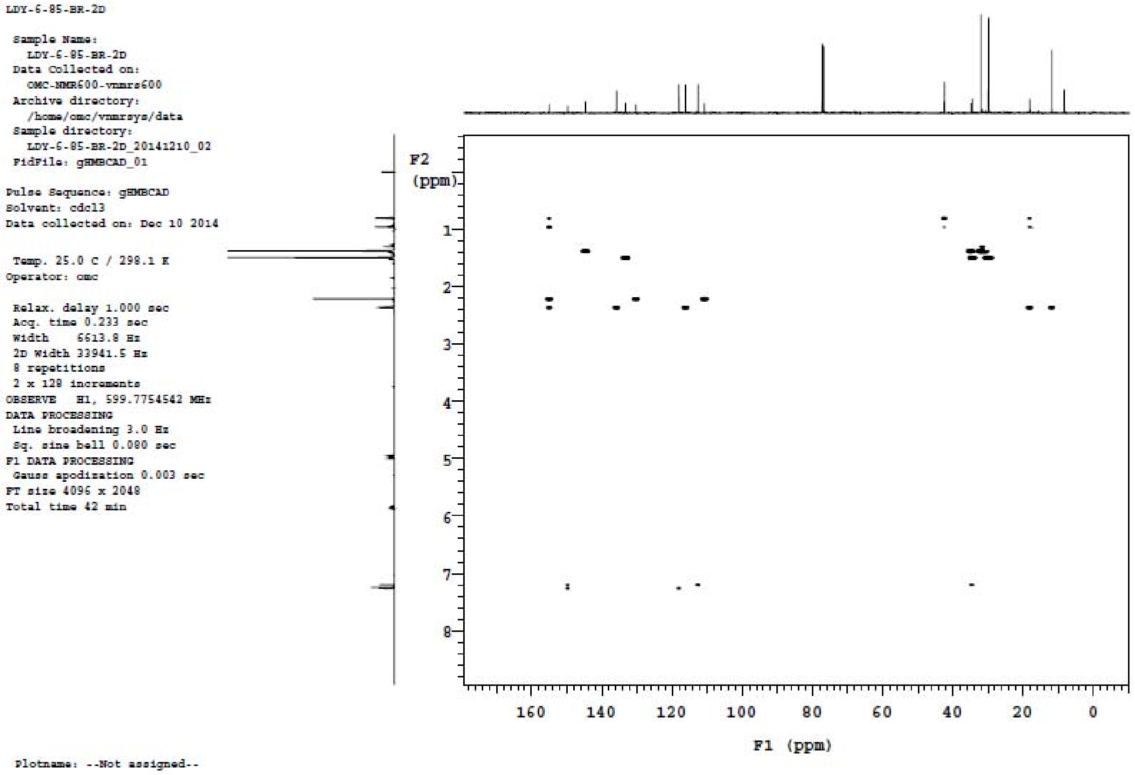
LDY-6-85-BR-2D
Sample Name:
LDY-6-85-BR-2D
Data Collected on:
OMC-NMR600-vnmrs600
Archive directory:
/home/omc/vnmrsys/data
Sample directory:
LDY-6-85-BR-2D_20141210_02
FidFile: gHMBCAD_01
Pulse Sequence: gHMBCAD
Solvent: cdcl3
Data collected on: Dec 10 2014
Temp. 25.0 C / 298.1 K
Operator: omc
Relax. delay 1.000 sec
Acq. time 0.233 sec
Width 6613.8 Hz
2D Width 33941.5 Hz
8 repetitions
2 x 128 increments
OBSERVE H1, 599.7754542 MHz
DATA PROCESSING
Line broadening 3.0 Hz
Sq. sine bell 0.080 sec
F1 DATA PROCESSING
Gauss apodization 0.003 sec
FT size 4096 x 2048
Total time 42 min
F2 (ppm)
F1 (ppm)
Plotname: --Not assigned--

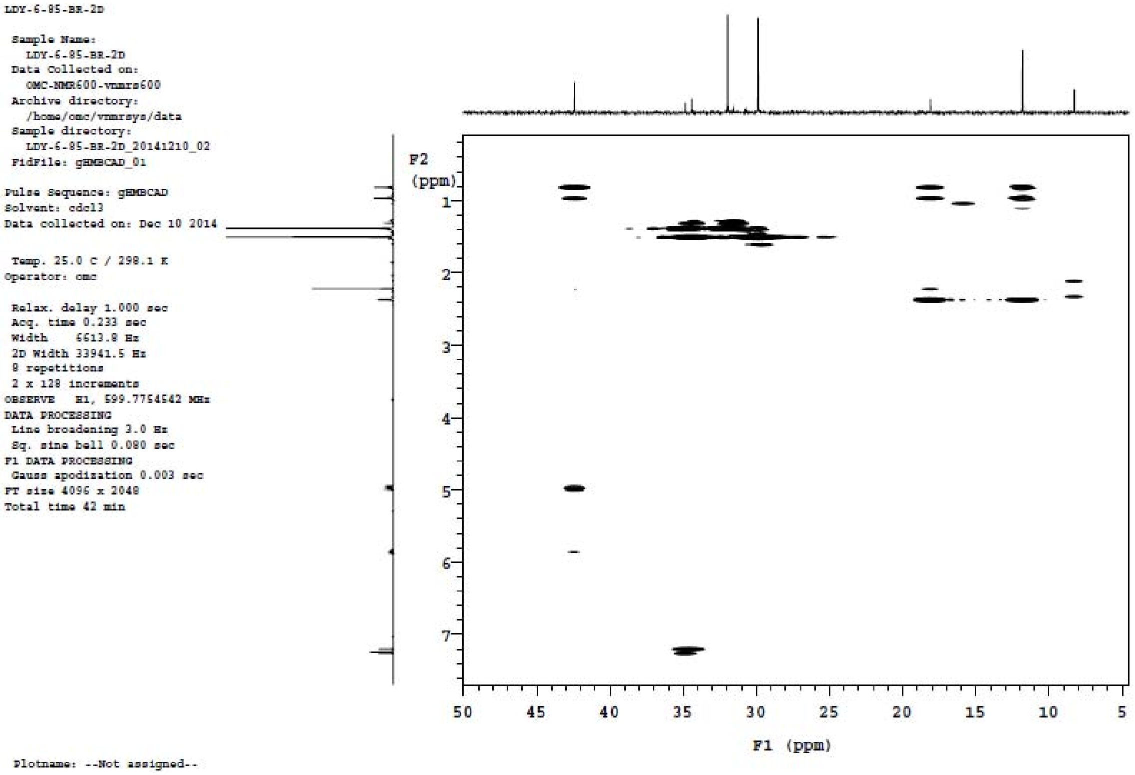
LDY-6-85-BR-2D
Sample Name:
LDY-6-85-BR-2D
Data Collected on:
OMC-NMR600-vnmrs600
Archive directory:
/home/omc/vnmrsys/data
Sample directory:
LDY-6-85-BR-2D_20141210_02
FidFile: gHMBCAD_01
Pulse Sequence: gHMBCAD
Solvent: cdcl3
Data collected on: Dec 10 2014
Temp. 25.0 C / 298.1 K
Operator: omc
Relax. delay 1.000 sec
Acq. time 0.233 sec
Width 6613.8 Hz
2D Width 33941.5 Hz
8 repetitions
2 x 128 increments
OBSERVE H1, 599.7754542 MHz
DATA PROCESSING
Line broadening 3.0 Hz
Sq. sine bell 0.080 sec
F1 DATA PROCESSING
Gauss apodization 0.003 sec
FT size 4096 x 2048
Total time 42 min
F2 (ppm)
F1 (ppm)
Plotname: --Not assigned--

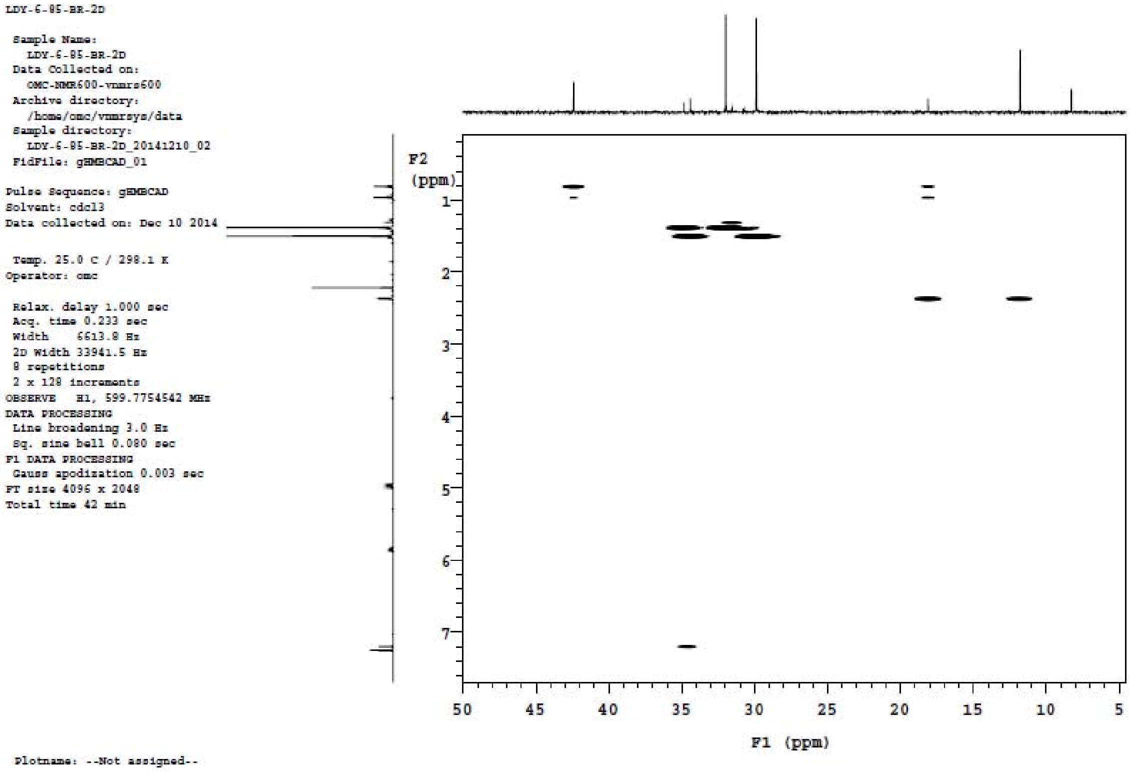
LDY-6-85-BR-2D
Sample Name:
LDY-6-85-BR-2D
Data Collected on:
OMC-NMR600-vnmrs600
Archive directory:
/home/omc/vnmrsys/data
Sample directory:
LDY-6-85-BR-2D_20141210_02
FidFile: gHMBCAD_01
Pulse Sequence: gHMBCAD
Solvent: cdcl3
Data collected on: Dec 10 2014
Temp. 25.0 C / 298.1 K
Operator: omc
Relax. delay 1.000 sec
Acq. time 0.233 sec
Width 6613.8 Hz
2D Width 33941.5 Hz
8 repetitions
2 x 128 increments
OBSERVE H1, 599.7754542 MHz
DATA PROCESSING
Line broadening 3.0 Hz
Sq. sine bell 0.080 sec
F1 DATA PROCESSING
Gauss apodization 0.003 sec
FT size 4096 x 2048
Total time 42 min
F2 (ppm)
F1 (ppm)
Plotname: --Not assigned--

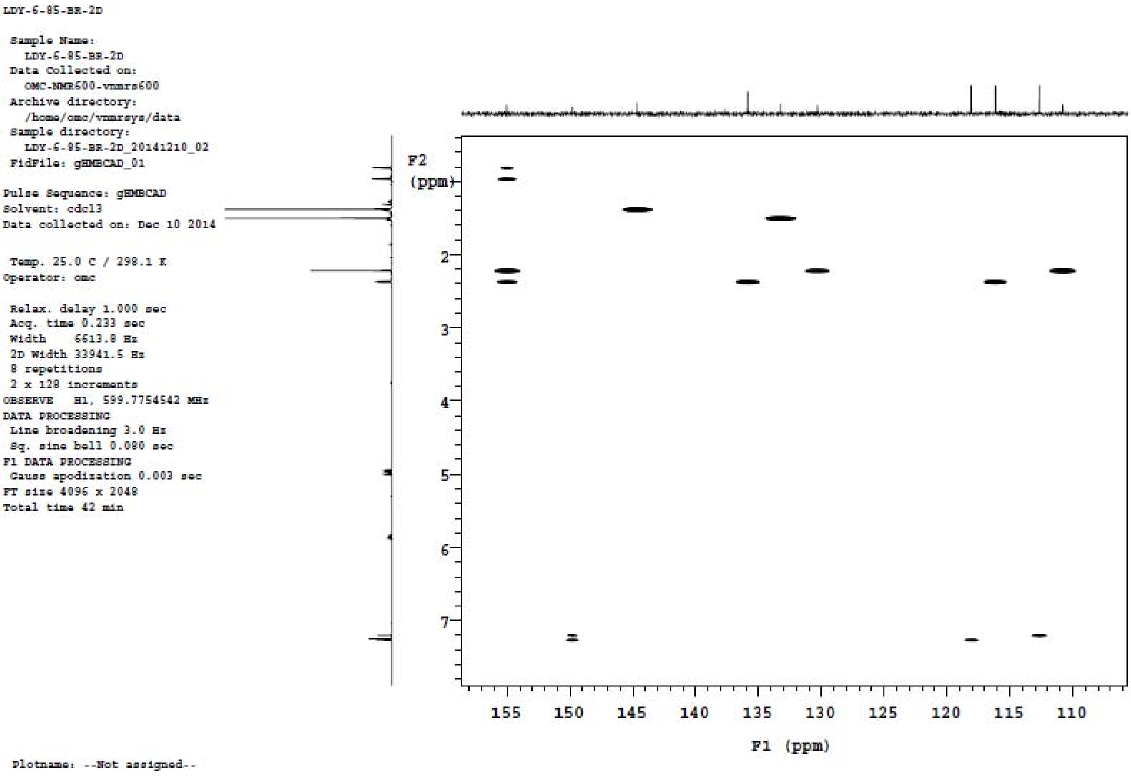
LDY-6-85-BR-2D
Sample Name:
LDY-6-85-BR-2D
Data Collected on:
OMC-NMR600-vnmrs600
Archive directory:
/home/omc/vnmrsys/data
Sample directory:
LDY-6-85-BR-2D_20141210_02
FidFile: gHMBCAD_01
Pulse Sequence: gHMBCAD
Solvent: cdcl3
Data collected on: Dec 10 2014
Temp. 25.0 C / 298.1 K
Operator: omc
Relax. delay 1.000 sec
Acq. time 0.233 sec
Width 6613.8 Hz
2D Width 33941.5 Hz
8 repetitions
2 x 128 increments
OBSERVE H1, 599.7754542 MHz
DATA PROCESSING
Line broadening 3.0 Hz
Sq. sine bell 0.080 sec
F1 DATA PROCESSING
Gauss apodization 0.003 sec
FT size 4096 x 2048
Total time 42 min
F2 (ppm)
F1 (ppm)
Plotname: --Not assigned--

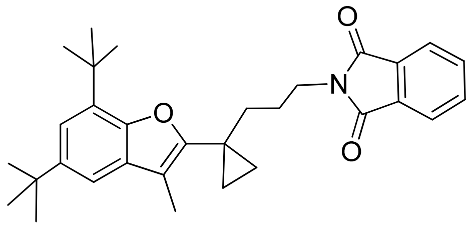

Compound **S8a**. 35 mg, yield: 75%; colorless oil. ^{1}H NMR ($CDCl_3$, 400 MHz, TMS) δ 0.75-0.77 (m, 2H, CH_2), 0.93-0.95 (m, 2H, CH_2), 1.37 (s, 9H, ^{t}Bu), 1.46 (s, 9H, ^{t}Bu), 1.64-1.66 (m, 2H, CH_2), 1.80-1.84 (m, 2H, CH_2), 2.21 (m, 2H, CH_2), 3.63-3.66 (m, 3H, CH_3), 7.24-7.25 (m, 2H, Ar), 7.68-7.70 (m, 2H, Ar), 7.80-7.82 (m, 2H, Ar). ^{13}C NMR ($CDCl_3$, 100 MHz, TMS) δ 8.26, 12.3, 18.0, 26.9, 29.7, 29.9, 32.0, 34.4, 34.8, 36.2, 38.0, 111.0, 112.7, 118.1, 123.1, 130.2, 132.1, 133.2, 133.8, 144.7, 149.8, 154.6, 168.3. IR (neat) ν 3080, 2953, 2867, 1772, 1712, 1613, 1594, 1466, 1436, 1394, 1361, 1243, 1100, 1033, 864, 718 cm^{-1}. MS (%) m/e 471 (M^+, 82.66), 472 (29.52), 311 (25.73), 298 (72.42), 295 (25.02), 283 (100.00), 160 (68.60), 57 (51.49). HRMS (EI) calcd. for $C_{31}H_{37}NO_3$: 471.2773, Found: 471.2771.

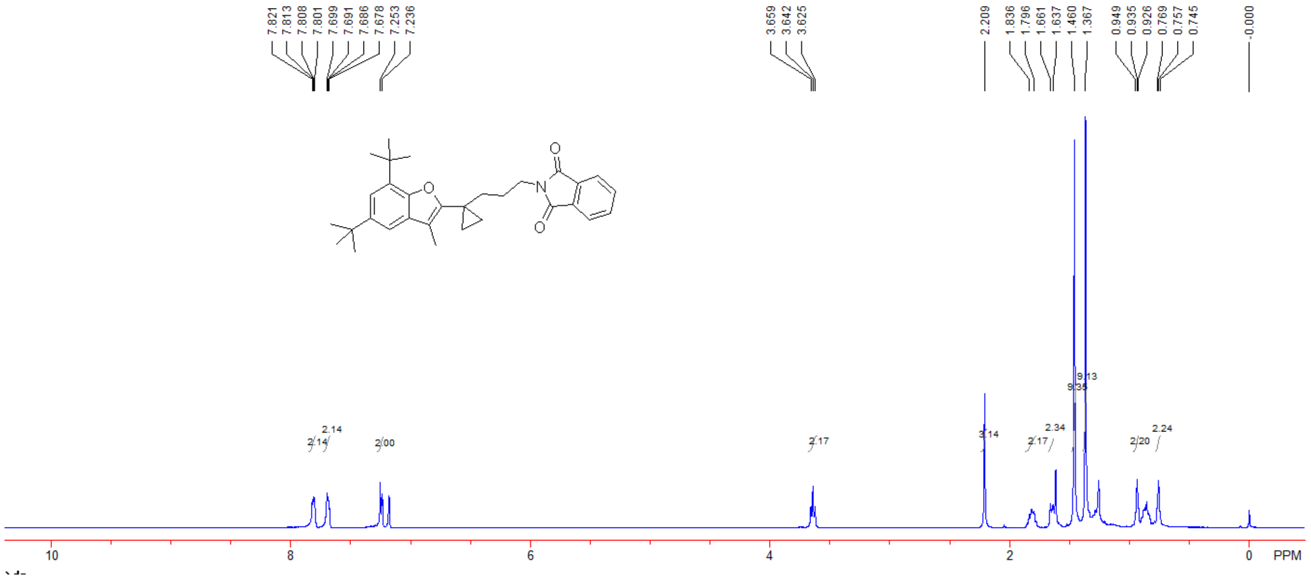

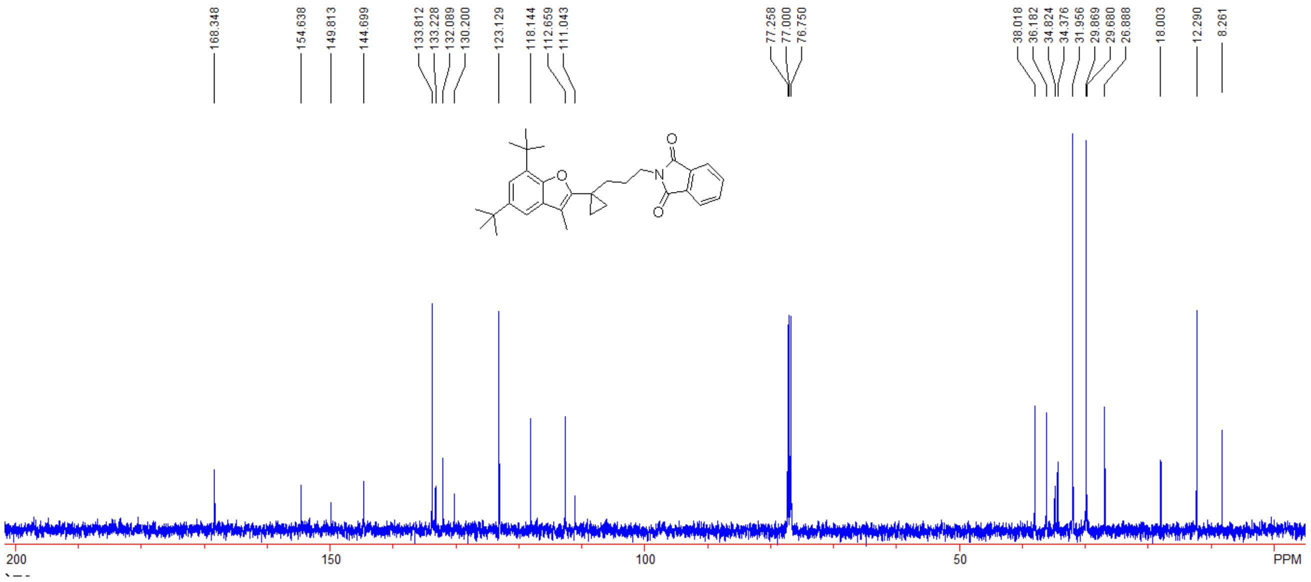

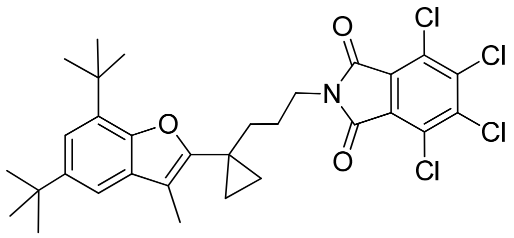

Compound **S8b**. 42 mg, yield: 70%; yellow solid, MP: 210-212 °C. ^{1}H NMR ($CDCl_3$, 400 MHz, TMS) δ 0.51-0.52 (m, 2H, CH_2), 0.55-0.56 (m, 2H, CH_2), 1.29 (s, 9H, ^{t}Bu), 1.38 (s, 9H, ^{t}Bu), 1.45 (m, 2H, CH_2), 2.08-2.14 (m, 5H, CH_3, CH_2), 3.89 (t, J = 6.8 Hz, 2H, CH_2), 6.87-6.88 (m, 1H, Ar), 7.21-7.22 (m, 1H, Ar). ^{13}C NMR ($CDCl_3$, 100 MHz, TMS) δ 8.6, 14.2, 21.0, 24.2, 29.0, 30.9, 31.5, 31.9, 34.4, 35.2, 36.6, 122.7, 125.3, 127.6, 129.6, 133.7, 138.9, 140.0, 141.4, 145.0, 152.7, 163.4, 171.2. IR (neat) ν 2954, 1772, 1715, 1431, 1400, 1374, 1358, 1221, 1200, 1144, 1058, 1024, 924, 751 cm^{-1}. HRMS (EI) calcd. for $(C_{31}H_{37}NO_3+NH_4)^+$: 641.1865, Found: 641.1866.

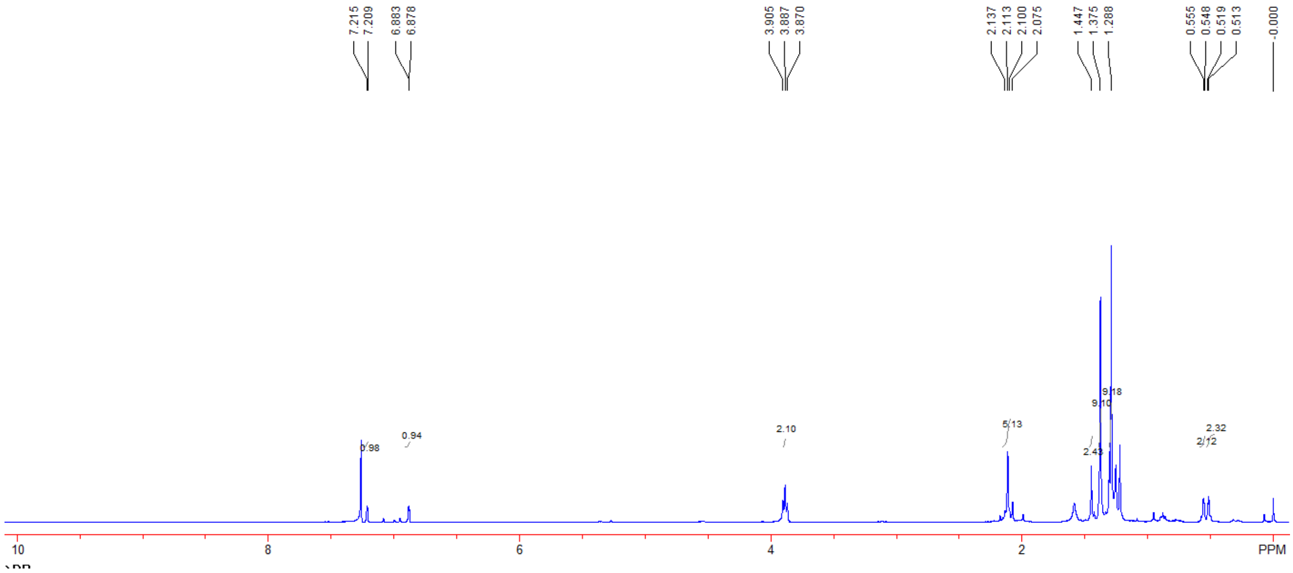
7.215
7.209
6.883
6.878
3.905
3.887
3.870
2.137
2.113
2.100
2.075
1.447
1.375
1.288
0.555
0.548
0.519
0.513
-0.000
0.98
0.94
2.10
5.13
2.43
9.10
9.18
2.32
2.12
10
8
6
4
2
PPM

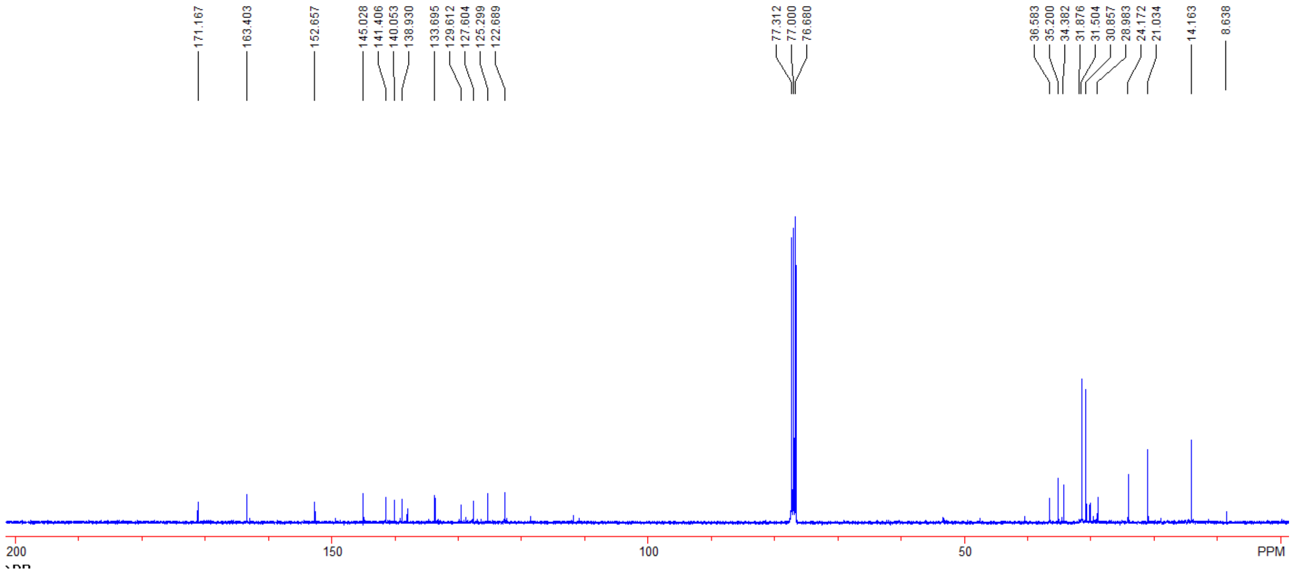
171.167
163.403
152.657
145.028
141.406
140.053
138.930
133.695
129.612
127.604
125.299
122.689
77.312
77.000
76.680
36.583
35.200
34.382
31.876
31.504
30.857
28.983
24.172
21.034
14.163
8.638
200
150
100
50
PPM

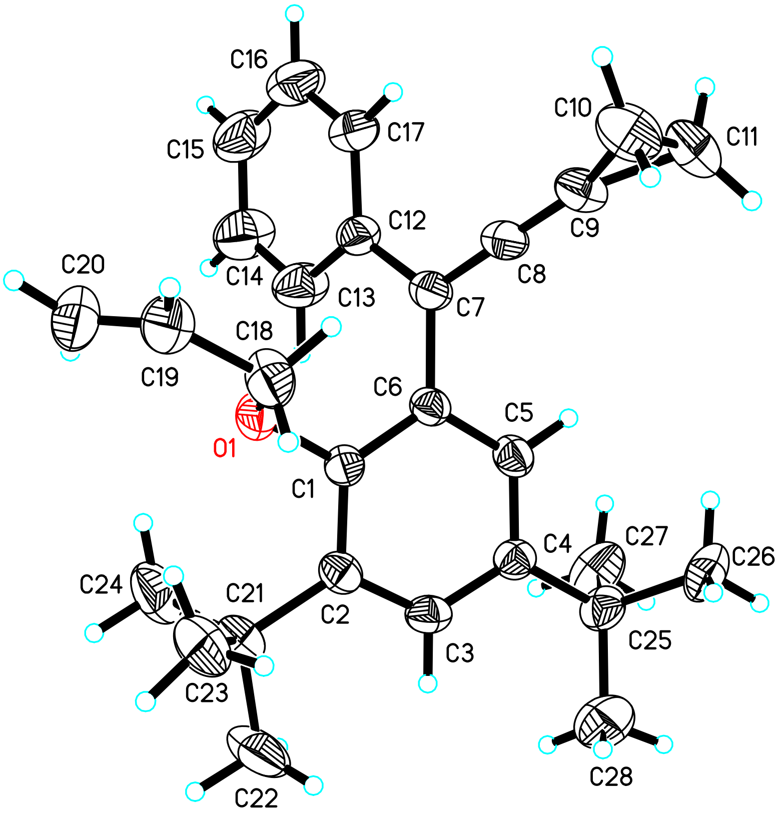

The crystal data of **3e** have been deposited in CCDC with number 996502. Empirical Formula: $C_{28}H_{34}O$; Formula Weight: 386.55; Crystal Color, Habit: colorless; Crystal System: Monoclinic; Crystal size: 0.211 x 0.165 x 0.123; Lattice Parameters: a = 23.302(4)Å, b = 11.4445(17)Å, c = 18.904(3)Å, α = 90°, β = 104.415(6)°, γ = 90°, V = 4882.7(14)Å^3; Space group: C 2/c; Z = 8; D_{calc} = 1.052 g/cm^3; F_{000} = 1680; Final R indices [I>2sigma(I)]: R1 = 0.0669; wR2 = 0.1756.

Distance between O1 and C8: 3.846 Å

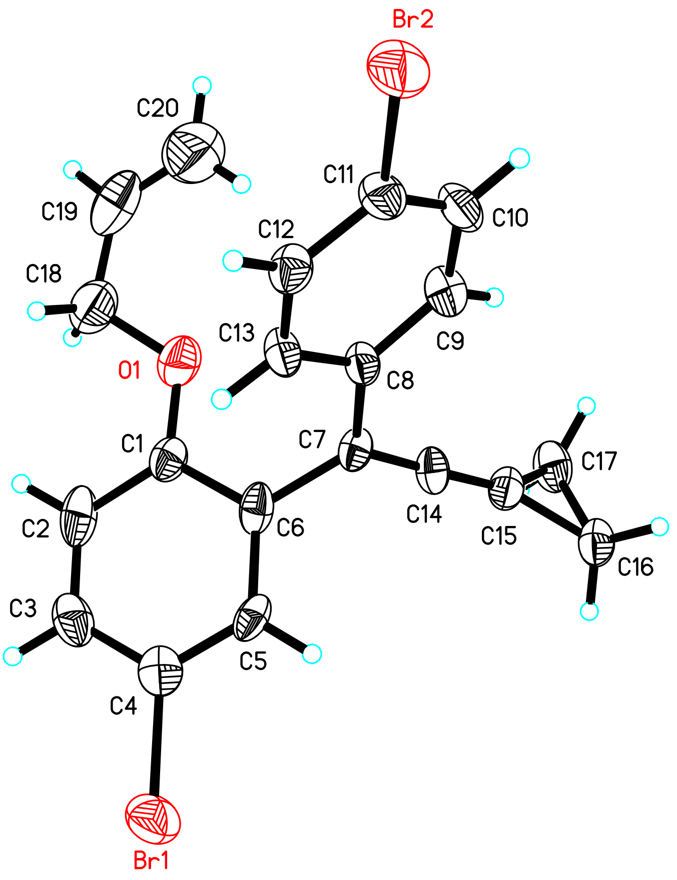

The crystal data of **1n** have been deposited in CCDC with number 980558. Empirical Formula: $C_{20}H_{16}Br_2O$; Formula Weight: 432.15; Crystal Color, Habit: colorless; Crystal System: Triclinic; Crystal size: 0.186 x 0.145 x 0.121; Lattice Parameters: a = 7.1574(15)Å, b = 11.250(2)Å, c = 22.434(5)Å, α = 84.231(5)°, β = 84.177(5)°, γ = 89.834(5)°, V = 1787.9(6)Å^3; Space group: P-1; Z = 4; D_{calc} = 1.605 g/cm^3; F_{000} = 856; Final R indices [I>2sigma(I)]: R1 = 0.0788; wR2 = 0.2136.

Distance between O1 and C14: 3.624 Å

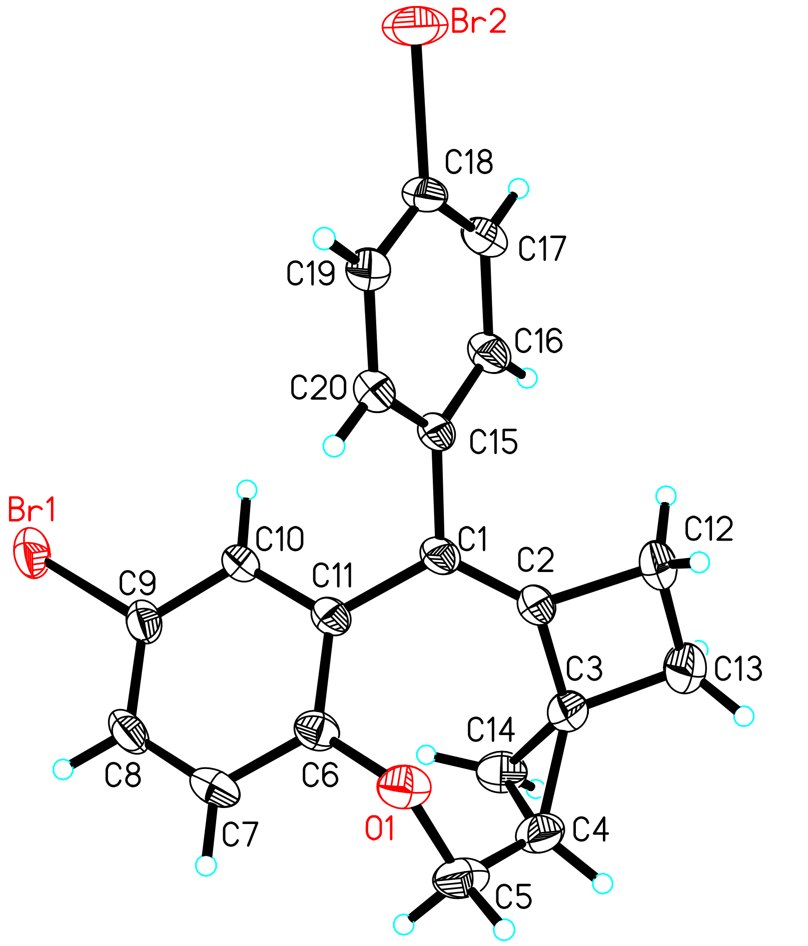

The crystal data of **2n** have been deposited in CCDC with number 997410. Empirical formula: $C_{20}H_{16}Br_2O$, Formula weight: 432.15, Temperature: 296(2) K, Wavelength: 1.54178 Å, Crystal system, space group: Triclinic, P-1; Unit cell dimensions: a = 6.0698(10) Å, alpha = 67.027(10) deg. b = 11.4589(10) Å, beta = 85.348(10) deg. c = 13.8421(3) Å, gamma = 76.337(10) deg. Volume: 861.26(2) $Å^3$, Z, Calculated density: 2, 1.666 Mg/m^3, F(000): 428, Crystal size: 0.25 x 0.15 x 0.12 mm, Final R indices [I>2sigma(I)]: R1 = 0.0364, wR2 = 0.1006; R indices (all data): R1 = 0.0373, wR2 = 0.1015.

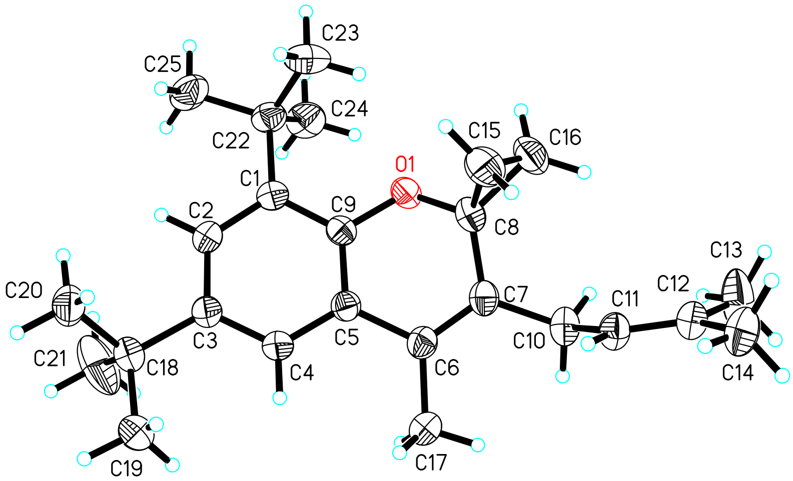

The crystal data of **5k** have been deposited in CCDC with number 996499. Empirical Formula: $C_{25}H_{36}O$, Formula weight: 352.54, Temperature: 296(2) K, Wavelength: 1.54178 Å, Crystal system, space group: Monoclinic, P 21/c, Unit cell dimensions: a = 14.4666(5) Å, alpha = 90 deg. b = 10.0707(3) Å, beta = 109.5080(10) deg. c = 16.4037(6) Å, gamma = 90 deg. Volume: 2252.65(13) Å^3, Z, Calculated density: 4, 1.039 Mg/m^3, F(000): 776; Crystal size: 0.50 x 0.32 x 0.24 mm; Final R indices [I>2sigma(I)]: R1 = 0.0584, wR2 = 0.1683; R indices (all data): R1 = 0.0599, wR2 = 0.1702.

The structures of **4a** and **6** were confirmed by Full-spectra analysis.

a) Chiral-HPLC analysis has inferred that compound **4** is a chiral product while compound **6** is not the case.

b) Here are the carbons and hydrogens of **4a** marked in the spectra (partially) judged by HSQC.

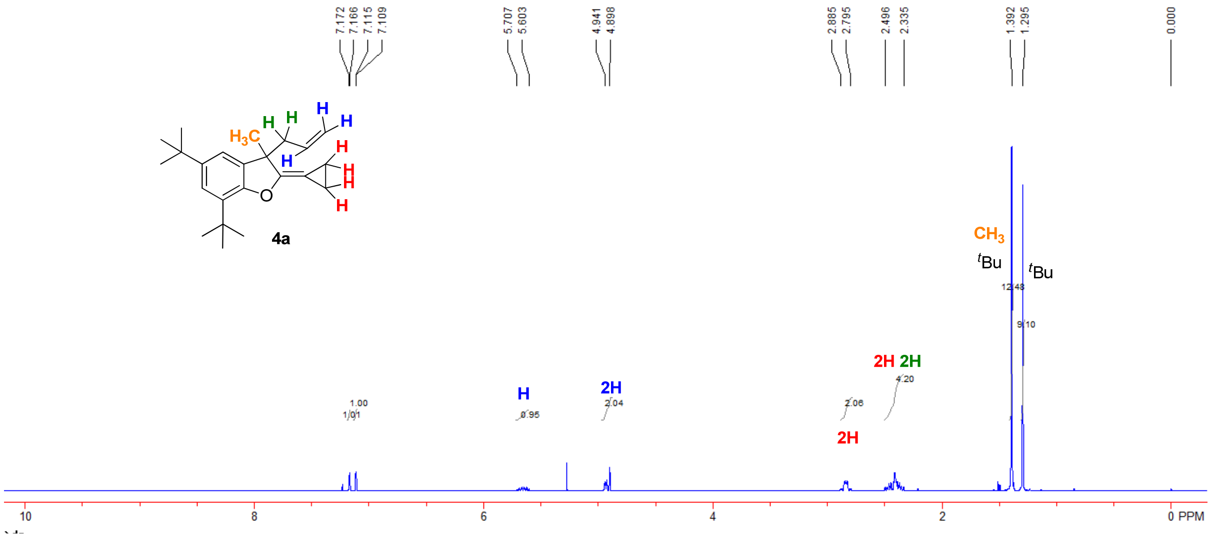

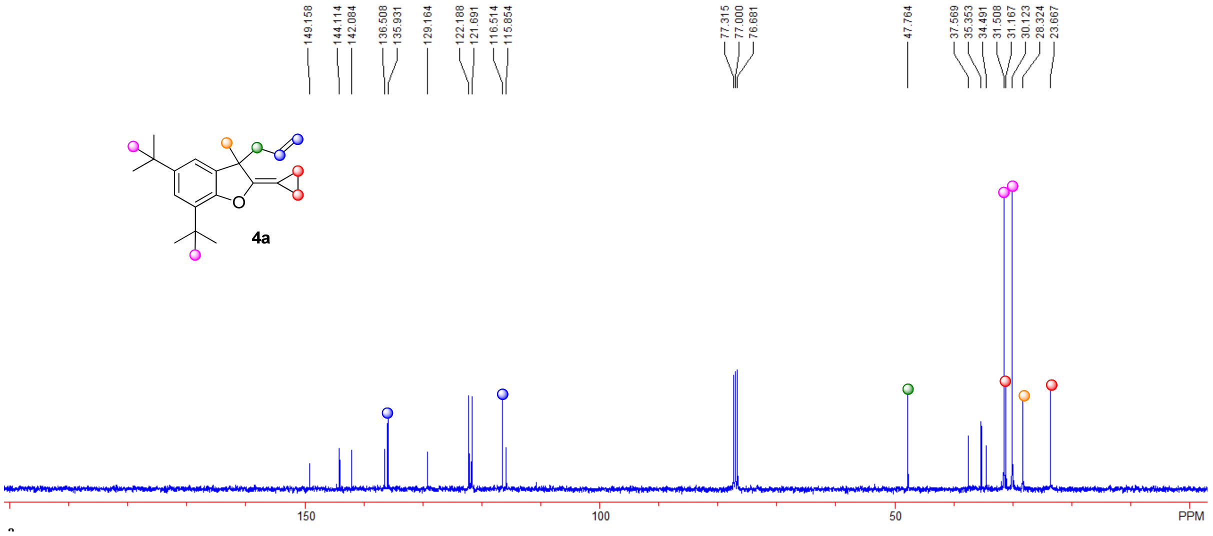

There is an obvious correlation between CH_2-H and Me-C judge from HMBC.

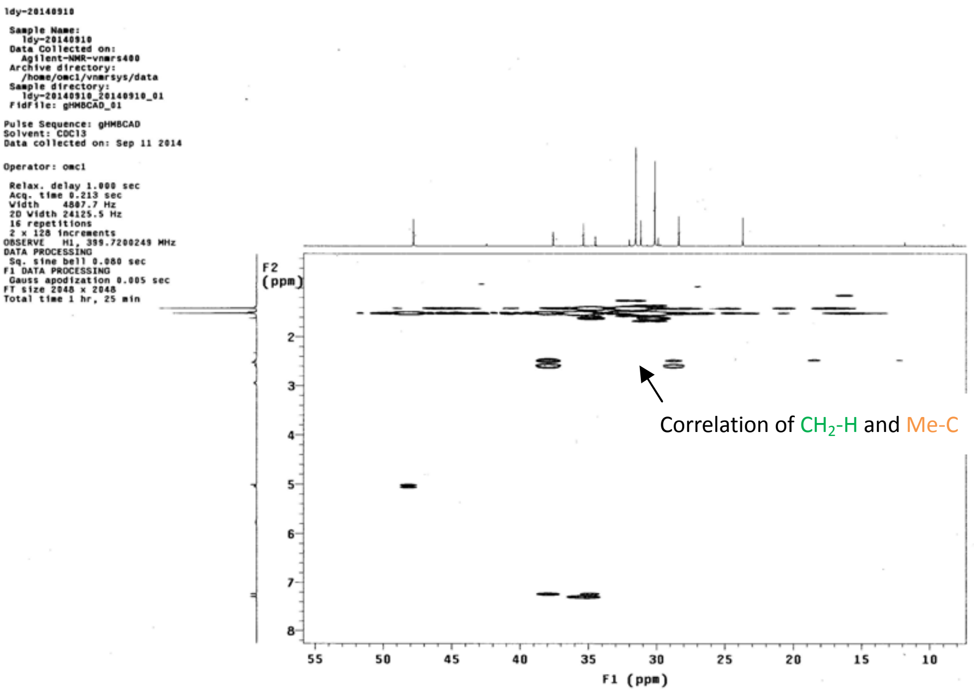

c) Here are the carbons and hydrogens of **6** marked in the spectra (partially) judged by HSQC.

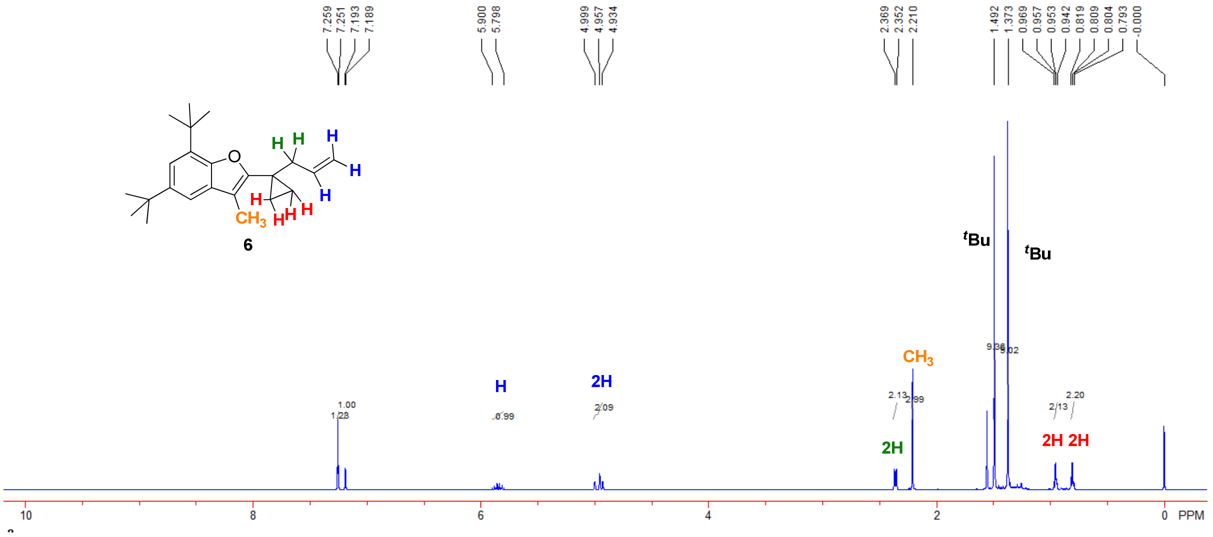

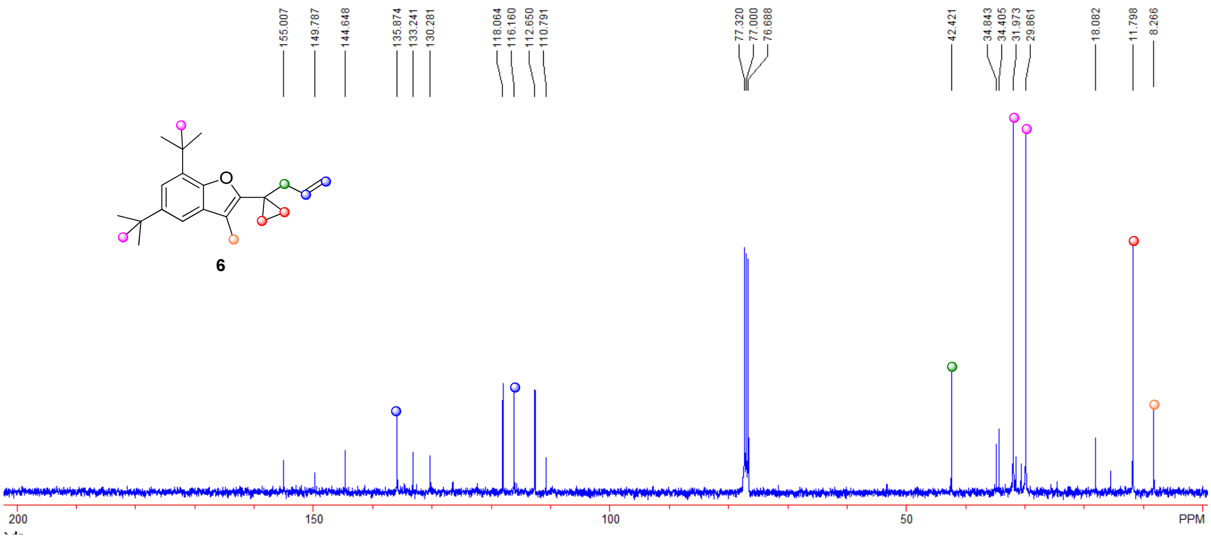

There is no correlation between CH_2-H and Me-C judge from HMBC.

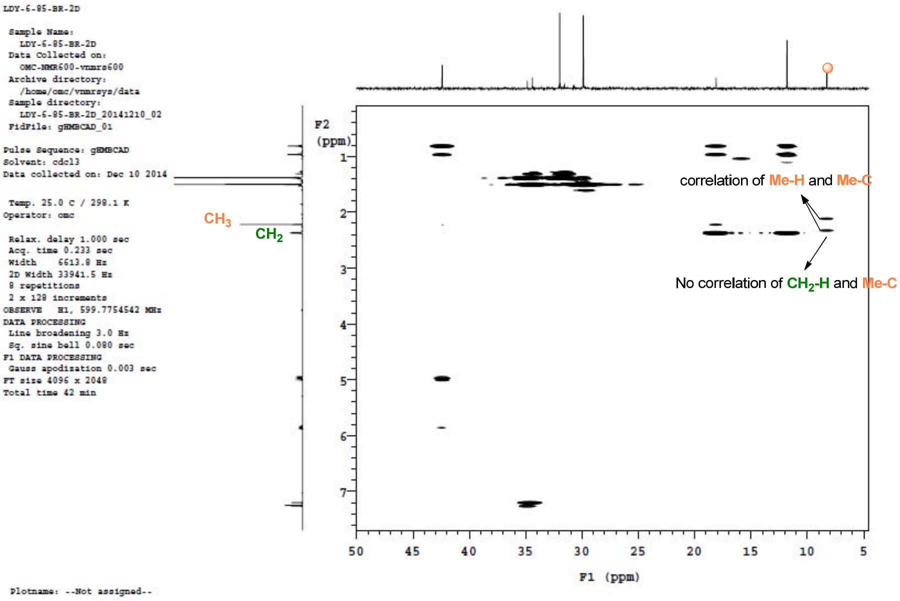

Calculation Details

The geometries of compounds not involving **Au** atom have been optimized at B3LYP/6-31+G(d) level; and the geometries of compounds involving **Au** atom have been optimized at B3LYP/6-31+G(d)/SDD level. The subsequent frequency calculations on the stationary points were carried out at the same level of theory to ascertain the nature of the stationary points as minima on the respective potential energy surfaces. The conformational space of flexible systems has first been searched manually. Thermochemical corrections to 298.15 K have been calculated for all minima from unscaled vibrational frequencies obtained at this same level. The thermochemical corrections have been combined with single-point energies calculated at the B3LYP/6-311+G(d,p)/SDD)// B3LYP/6-31+G(d)/SDD level to yield free energy G_{298} at 298.15 K. All quantum mechanical calculations have been performed with Gaussian 09.[5]

Table S2. The total energies, enthalpies and free energies of **1a** and **2a**.

	E_{tot} (E_h)[a]	H_{298}[a]	G_{298}[a]
**1a**	-656.3435715	-656.061145	-656.123772
**2a**	-656.3851386	-656.099716	-656.152477

[a] Calculated at the B3LYP/6-31+G(d) level of theory

Table S3. The total energies, enthalpies and free energies of all species corresponding to intermediates **D** and **E**.

***Ortho*-substituent**		E_{tot} (E_h)[a]	H_{298}[a]	G_{298}[a]
**F**	**D**	-1083.071707	-1082.73795	-1082.81737
	**E**	-1083.084999	-1082.74911	-1082.82362
**H**	**D**	-983.8141304	-983.472791	-983.549372
	**E**	-983.8273646	-983.483985	-983.556316
**Cl**	**D**	-1443.422776	-1443.09012	-1443.17048
	**E**	-1443.437299	-1443.10257	-1443.17745
**Br**	**D**	-3557.343428	-3557.01087	-3557.09228
	**E**	-3557.357159	-3557.02269	-3557.09886
***t*Bu**	**D**	-1141.091593	-1140.63218	-1140.72118
	**E**	-1141.106079	-1140.64459	-1140.72908

[a] Calculated at the B3LYP/6-311+G(d,p)/SDD// B3LYP/6-31+G(d)/SDD level of theory

The thermodynamic stability of the subsequent oxonium intermediate **E** with different ortho-substituents was investigated by DFT calculations (Table S4). The results showed that the

ortho-substituents indeed influence the thermodynamic stability of oxonium intermediates especially in the comparison of ^{t}Bu with F. **E**'s formation is thermodynamically more favorable, which may account for the substrate **3** with ^{t}Bu substituent to preferentially undergo the non-carbene pathways. The reactions were probably controlled by kinetic factors judged by the reaction conditions (5 minutes at room temperature). Therefore, reactivities of substrates with H, Cl, Br substituents could not be interpreted very well in terms of the stabilities of intermediates. After all, we believe that the carbene/non-carbene processes was determined at the very beginning of the reaction due to the nucleophilicity of the O atom.

Table S4. $\Delta G_{(E\text{-}D)}$ corresponding to intermediates **D** and **E**.

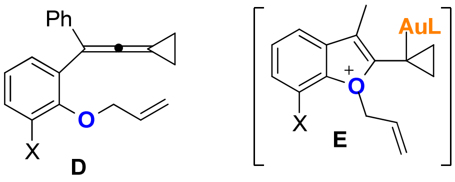

X	$\Delta G_{(E\text{-}D),298}$ (kcal/mol)[a]
F	-3.90
H	-4.34
Cl	-4.35
Br	-4.11
*t*Bu	-4.93

[a] Calculated at B3LYP/6-311+G(d,p)/SDD// B3LYP/6-31+G(d)/SDD

A calculation of Mulliken Charge on O atom with the affection of X atom (group) and Y atom based on B3LYP/6-31+G(d) level

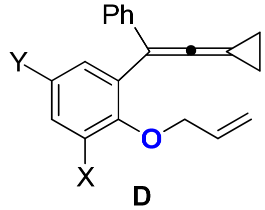

D

X	Y	Mulliken Charge on O atom[a]
F	H	-0.244
H	H	-0.301
Cl	H	-0.308
Br	H	-0.315
*t*Bu	H	-0.347
H	F	-0.242
H	Cl	-0.247
H	Br	-0.248

[a] Calculated at B3LYP/6-31+G(d) level

Table S5. The total energies, enthalpies and free energies of all species shown in Scheme 6.

	E_{tot} (E_h)[a]	H_{298}[a]	G_{298}[a]
**3k-D**	-1646.207133	-1645.502322	-1645.620185
**3k-TS1**	-1646.198514	-1645.494036	-1645.607942
**3k-I**	-1646.213042	-1645.506227	-1645.618406
**3k-TS2**	-1646.208886	-1645.504629	-1645.616572
**5k-P**	-1646.268264	-1645.563031	-1645.676592
**3k-TS1’**	-1646.192284	-1645.487409	-1645.602359
**3k-F**	-1646.200175	-1645.493235	-1645.607485
**3k-TS2’**	-1646.199690	-1645.494524	-1645.605824
**4k-P**	-1646.270579	-1645.561158	-1645.674408

a. Calculated at B3LYP/6-31+G(d)/SDD level

Table S6. The total energies, enthalpies and free energies of all species shown in Scheme 7.

	E_{tot} (E_h)[a]	H_{298}[a]	G_{298}[a]
**3a-D**	-1567.569262	-1566.923857	-1567.034687
**3a-TS1**	-1567.560393	-1566.914922	-1567.019938
**3a-I**	-1567.572427	-1566.924658	-1567.030376
**3a-TS2**	-1567.558151	-1566.913959	-1567.021311
**5a-P**	-1567.630929	-1566.982946	-1567.088562
**3a-TS1’**	-1567.557489	-1566.91197	-1567.02053
**3a-F**	-1567.567768	-1566.921287	-1567.025324
**3a-TS2’**	-1567.559413	-1566.915123	-1567.024677
**4a-P**	-1567.633353	-1566.985276	-1567.091485

a. Calculated at B3LYP/6-31+G(d)/SDD level

Archive Entries

1a

1\1\GINC-SHI_02\FOpt\RB3LYP\6-31+G(d)\C15H16O1\YIN\26-Jan-2015\0\\#p o
 pt b3lyp/6-31+g(d) geom=check\\vdcp_me_3\\0,1\C,0.0154529567,-0.014677
 5644,-0.0042907707\C,0.069743215,-0.0004236659,1.5252553722\H,0.958438
 7918,-0.030638619,-0.5497473973\H,-0.7775951206,0.5493759285,-0.493866
 9223\H,-0.6872437593,0.5729282308,2.0591597469\H,1.0483142822,-0.00843
 11972,2.0036933905\C,-0.3582190697,-1.2113460822,0.786821033\C,-0.7608
 478363,-2.4394169549,0.814145241\C,-1.1737647584,-3.6925055662,0.85392
 07177\C,-0.173323881,-4.8030316192,0.7702087389\C,0.730436007,-4.85206
 487,-0.2987342665\C,-0.1003766487,-5.8261866943,1.7463159455\C,1.67614
 64406,-5.8740972631,-0.4243576998\H,0.6754095377,-4.0681142816,-1.0493
 714519\C,0.8398239851,-6.8573919346,1.623901547\C,1.7230419999,-6.8792
 39234,0.5389569284\H,2.3595868915,-5.8842682221,-1.2689858786\H,0.9028
 25985,-7.6397480052,2.3716777469\H,2.4464630633,-7.6868533419,0.459397
 7978\O,-0.9671885084,-5.7189277143,2.8027142792\C,-0.9416425736,-6.720
 5831832,3.824039623\H,-1.1363833453,-7.7043009586,3.3688873165\H,0.046
 7616739,-6.7512068642,4.3027596139\C,-2.0074405718,-6.3901666265,4.826
 0118443\C,-1.7892701147,-6.2863211605,6.138963759\H,-3.0107547754,-6.2
 573261679,4.4221258196\H,-2.5972557818,-6.080908512,6.8361495133\H,-0.
 7947638529,-6.4045111684,6.5656815341\C,-2.6533925777,-4.0350037646,0.
 9038475849\H,-2.8911957401,-4.611549742,1.8029433839\H,-3.2615435934,-
 3.125572246,0.8958356485\H,-2.9313781977,-4.6467453817,0.0346182293\\V
 ersion=EM64L-G09RevA.01\State=1-A\HF=-656.3435715\RMSD=5.595e-09\RMSF=
 2.331e-06\Dipole=0.03525,-0.2268,0.3766786\Quadrupole=-1.9340626,1.893
 8614,0.0402011,-0.8726637,-1.8882157,-4.0098918\PG=C01 [X(C15H16O1)]\\
 @

2a

1\1\GINC-SHI_02\FOpt\RB3LYP\6-31+G(d)\C15H16O1\YIN\15-Jan-2015\0\\#p o
 pt b3lyp/6-31+g(d)\\Title Card Required\\0,1\C,-0.0009413976,0.0491309
 85,0.0646170109\C,0.0517557135,-0.0717298998,1.4561936882\C,1.27322370
 58,-0.0156776148,2.165376564\C,2.4279590882,0.1882843484,1.3726731493\
 C,2.3810096638,0.3308090716,-0.0132188256\C,1.1581067153,0.2556234059,
 -0.6814834311\H,-0.9711989241,-0.0356504802,-0.4174082419\H,3.39673131
 34,0.2478141813,1.8545934545\H,3.3026545578,0.4928942551,-0.5663142211
 \H,1.1056197849,0.3484133462,-1.7630973153\O,-1.1365718414,-0.30889758
 14,2.1126991108\C,1.4347086466,-0.1651864085,3.6406251383\C,0.52828068
 96,0.0468264736,4.6238738693\C,0.8017600878,0.1845403523,6.1281375064\
 C,-0.507643659,1.0194374211,6.2421867547\H,1.7297555729,0.6887961407,6
 .4167974989\H,0.7348022827,-0.7673694409,6.6717944263\H,-0.3051232113,
 2.0970302564,6.2345504541\H,-1.1812662539,0.7833252492,7.0729525408\C,
 -1.8992919085,0.8498333165,2.4806920594\H,-1.4044896935,1.7557513714,2
 .1091192227\H,-2.8837226069,0.7708602966,1.9992507553\C,-0.899360364,0
 .4762875497,4.8603404305\C,-2.1187746425,-0.4313739092,4.7207508286\C,

-2.0472197357,0.8826221736,3.9837293786\H,-2.0026833786,-1.3362841856,
4.1326874803\H,-2.7759840206,-0.5104386134,5.5844741532\H,-2.669920433
2,1.698792602,4.3533308722\C,2.8375555567,-0.5505570483,4.1000917413\H
,3.244476753,-1.3606246317,3.4835221343\H,3.5469207385,0.2876883699,4.
0445372135\H,2.8314372369,-0.9015004047,5.1340380893\\Version=EM64L-G0
9RevA.01\State=1-A\HF=-656.3851386\RMSD=5.436e-09\RMSF=4.634e-06\Dipol
e=-0.0856314,0.5009593,0.2970494\Quadrupole=2.2429127,-4.5802753,2.337
3626,-1.826781,-0.5658551,-0.974652\PG=C01 [X(C15H16O1)]\\@

***Ortho*-substituent F-D**

1\1\GINC-SHI_02\SP\RB3LYP\GenECP\C20H17Au1F1O1(1+)\YIN\21-Jan-2015\0\\
#p geom=check b3lyp/genecp\\vdcp_ph_f_int1_2-sp\\1,1\C,0,-0.1302505041
,-0.0005817107,-0.2124021126\C,0,-0.1322421411,0.0952820651,1.31223433
61\H,0,0.8191533048,-0.0007333021,-0.7446333862\H,0,-0.9486493018,0.48
0967367,-0.7411558611\H,0,-0.9466945832,0.6486035189,1.7722179028\H,0,
0.8163164755,0.1565705572,1.8423770145\C,0,-0.4251353803,-1.1962859783
,0.6271587099\C,0,-0.1787916669,-2.5265826954,0.706948919\C,0,0.232610
5646,-3.7896388627,0.6319899877\C,0,-0.7861920921,-4.8793182109,0.7203
952338\C,0,-0.7741099405,-5.925516506,-0.2068067878\C,0,-1.8469028493,
-4.8132001726,1.6681991579\C,0,-1.8267107543,-6.8474149471,-0.24220242
85\H,0,0.0379609969,-6.0002997403,-0.9240507329\C,0,-2.9006078135,-5.7
460864655,1.6198847126\H,0,-1.7209989189,-4.2099407461,2.5683073556\C,
0,-2.9003519623,-6.7494377409,0.65076847\H,0,-1.8127694788,-7.64080656
77,-0.9842558814\H,0,-3.6822451069,-5.7100456716,2.3736854797\H,0,-3.7
106542596,-7.4710261165,0.6106148091\C,0,1.6714023816,-4.0781134379,0.
3695242421\C,0,2.3178832733,-3.5116432625,-0.7398805231\C,0,2.37286115
08,-4.9393904442,1.2497822962\C,0,3.6575988582,-3.7927339257,-1.005478
3359\H,0,1.752038024,-2.8694662581,-1.4088695469\C,0,3.7113899807,-5.2
220891591,0.9327677748\C,0,4.355276183,-4.657336726,-0.1610297237\H,0,
4.1512898749,-3.3590482323,-1.8689848581\H,0,5.3935883915,-4.918686425
2,-0.340549374\O,0,1.6745527079,-5.42977235,2.3011217742\C,0,2.3376208
557,-5.9287301789,3.5027931636\H,0,2.7436519953,-6.9242145425,3.308647
8755\H,0,3.1606174287,-5.2479010535,3.7484551441\C,0,1.3172718098,-5.9
572810469,4.5976053042\C,0,0.9804996477,-7.0665604342,5.2614438756\H,0
,0.8750819276,-4.9982028912,4.8673246765\H,0,0.2719015647,-7.045317525
1,6.0849969357\H,0,1.4137345834,-8.0343783691,5.0164766441\F,0,4.41430
01852,-6.0863098737,1.7071290826\Au,0,-2.3664896732,-2.36704033,0.9180
485846\\Version=EM64L-G09RevA.01\State=1-A\HF=-1083.0717073\RMSD=2.445
e-09\Dipole=-0.967585,1.0476387,-0.4246671\Quadrupole=2.9188587,6.5544
858,-9.4733445,0.5745209,-4.3826372,-4.3042094\PG=C01 [X(C20H17Au1F1O1
)]\\@

***Ortho*-substituent F-E**

1\1\GINC-SHI_03\SP\RB3LYP\GenECP\C20H17Au1Br1O1(1+)\YIN\05-May-2015\0\
\#p b3lyp/genecp geom=check\\Title Card Required\\1,1\C,0,-0.426394500

1,-0.2831089794,-0.1270417182\C,0,-0.3911608485,-0.2346234319,1.373665
5399\H,0,0.5059253209,-0.1890757231,-0.6770890816\H,0,-1.2889364167,0.
1357204237,-0.6342380804\H,0,-1.2263895062,0.2231461896,1.8931869264\H
,0,0.5689282861,-0.1007182119,1.8670184019\C,0,-0.6298602146,-1.565432
5986,0.6757564886\C,0,0.4207350583,-2.5576049663,0.6220287692\C,0,2.16
24096052,-4.0124174752,0.2497439453\C,0,3.1519082233,-4.827337249,-0.3
116563458\C,0,2.0410379137,-3.9349315543,1.639223776\C,0,4.0046384944,
-5.5181680154,0.5483709135\H,0,3.247329832,-4.9175413228,-1.3879843238
\C,0,2.9152161665,-4.5676228746,2.5145646886\C,0,3.9001341017,-5.38050
95127,1.93768293\H,0,4.5957312992,-5.8974634813,2.5900899653\O,0,0.952
8987204,-3.0634367204,1.9471222095\C,0,-0.0967700905,-3.4922218311,3.0
708669039\H,0,-1.0214196397,-3.6116685795,2.5074027549\H,0,0.311136268
6,-4.4497251591,3.3872115415\C,0,-0.1559558187,-2.4656935826,4.1319193
046\C,0,-1.3012164735,-1.8505427986,4.4513097475\H,0,0.7495658633,-2.2
984296612,4.7086536378\H,0,-1.3577878049,-1.1741881687,5.2995326168\H,
0,-2.2195690533,-2.016348207,3.8924927008\C,0,1.1619991042,-3.14148586
87,-0.3551333487\Au,0,-2.5422594756,-2.3497780105,0.6773040822\H,0,4.7
774124075,-6.1629653841,0.1415027851\C,0,1.0207581902,-2.9070622852,-1
.807674105\C,0,2.1531434568,-2.5898856483,-2.5812782088\C,0,-0.2322674
707,-3.0053224905,-2.4353103065\C,0,2.0289288643,-2.3715804568,-3.9539
687583\H,0,3.1252637927,-2.4876709929,-2.106367605\C,0,-0.3493628737,-
2.7989072988,-3.8105298665\H,0,-1.1112364003,-3.2572675678,-1.84856561
84\C,0,0.7791420779,-2.480160971,-4.5721638473\H,0,2.9072125446,-2.115
2404685,-4.5396018942\H,0,-1.3213352757,-2.8884230512,-4.2874590117\H,
0,0.6854083261,-2.3159767469,-5.6418466088\Br,0,2.8978020558,-4.354249
1029,4.4009242028\\Version=EM64L-G09RevA.01\State=1-A\HF=-3557.3571591
\RMSD=6.634e-09\Dipole=0.7235333,-0.13295,0.1737702\Quadrupole=3.34984
74,-13.817358,10.4675106,-11.59832,-3.7331545,-1.1121865\PG=C01 [X(C20
H17Au1Br1O1)]\\@

***Ortho*-substituent H-D**

1\1\GINC-SHI_02\SP\RB3LYP\GenECP\C20H18Au1O1(1+)\YIN\21-Jan-2015\0\\#p
 Geom=check b3lyp/genecp\\vdcp_ph_h_int1-sp\\1,1\C,0,0.0331680384,0.04
45681399,0.0092939797\C,0,0.0153541783,0.0344475122,1.537450392\H,0,0.
9882367092,0.0303678016,-0.5125636367\H,0,-0.7511009541,0.6060676449,-
0.4916317355\H,0,-0.7750673416,0.5980046319,2.0259389327\H,0,0.9579889
933,0.0071903547,2.0807629249\C,0,-0.3350892433,-1.1886650756,0.760440
0111\C,0,-0.1509583667,-2.5331696535,0.7526265044\C,0,0.2101223546,-3.
8037167773,0.5929723415\C,0,-0.8493392551,-4.8568410938,0.6079168512\C
,0,-0.8641209843,-5.8457959918,-0.3808193585\C,0,-1.9152805574,-4.8107
06109,1.5490137155\C,0,-1.9457405271,-6.7281988484,-0.4782675453\H,0,-
0.0485472349,-5.9037941518,-1.0956146387\C,0,-2.998074922,-5.703172703
4,1.4392357006\H,0,-1.7833100545,-4.2554017874,2.47852145\C,0,-3.02233
5148,-6.6479520031,0.4131327483\H,0,-1.9526423113,-7.4754270396,-1.266
9379493\H,0,-3.7859692397,-5.6835455503,2.1871425496\H,0,-3.8559127903

,-7.3381987531,0.3251166093\C,0,1.6374092567,-4.1268921682,0.318639394
8\C,0,2.3208527154,-3.5098189925,-0.7398310246\C,0,2.3160952618,-5.070
866902,1.1289160318\C,0,3.6499346244,-3.8283415928,-1.0200981254\H,0,1
.7875411566,-2.799031621,-1.366035359\C,0,3.6478092777,-5.395833344,0.
8395114887\C,0,4.3035669768,-4.7740801779,-0.2275145299\H,0,4.16226681
43,-3.3554946528,-1.8520338822\H,0,5.3367030177,-5.0376620673,-0.43589
57942\O,0,1.6010035263,-5.5871311331,2.1619654333\C,0,2.2173511625,-6.
5693714993,3.0249884177\H,0,2.4853516278,-7.4567155144,2.4381638151\H,
0,3.1328538003,-6.1333954294,3.4482248828\C,0,1.2444088855,-6.91088415
92,4.1113976393\C,0,0.8191986588,-8.1522741248,4.3597218224\H,0,0.9193
552147,-6.0801466333,4.7371644924\H,0,0.1504158604,-8.3692579797,5.188
1273246\H,0,1.1350638937,-8.9996571713,3.7541008445\Au,0,-2.3280699239
,-2.2709888069,0.9464403503\H,0,4.182024582,-6.1218115202,1.4409547319
\\Version=EM64L-G09RevA.01\State=1-A\HF=-983.8141304\RMSD=6.844e-09\Di
pole=-0.246414,0.4816614,-0.1148448\Quadrupole=4.8126908,9.5086432,-14
.321334,-3.8313315,-2.5296033,-3.8101736\PG=C01 [X(C20H18Au1O1)]\\@

***Ortho*-substituent H-E**

1\1\GINC-A326\SP\RB3LYP\GenECP\C20H18Au1O1(1+)\SIOCWY\04-May-2015\0\\#
p b3lyp/genecp geom=check\\Title Card Required\\1,1\C,0,0.0619871429,0
.0917648571,0.0391577941\C,0,0.0118864037,0.1062900804,1.5402583074\H,
0,1.0340700044,0.0758296034,-0.4429601516\H,0,-0.7026753394,0.64615461
42,-0.5016441403\H,0,-0.7845054102,0.6599382267,2.0297470847\H,0,0.950
4607698,0.0962503091,2.0837719253\C,0,-0.3852330837,-1.160032293,0.793
4518921\C,0,-1.778713464,-1.5395010854,0.7997549779\C,0,-2.7860104943,
-1.62351481,1.7095621284\C,0,-2.6597082018,-1.3579107643,3.157757946\C
,0,-3.5821382498,-0.5112578159,3.8003656918\C,0,-1.6335364887,-1.95180
38477,3.9120652658\C,0,-3.4723968002,-0.2608544343,5.1689337271\H,0,-4
.3687331707,-0.0287304194,3.226320428\C,0,-1.5353058288,-1.7079083835,
5.2826495759\H,0,-0.9238031839,-2.6169093769,3.4280076485\C,0,-2.45185
63178,-0.8610411161,5.9134150329\H,0,-4.1826976411,0.4034257075,5.6529
468719\H,0,-0.7446990143,-2.181114263,5.8582102822\H,0,-2.3718086961,-
0.6698059756,6.9797183665\C,0,-4.0214386494,-2.0089309813,1.0367557714
\C,0,-5.3186062453,-2.2702604013,1.5006889192\C,0,-3.7688403313,-2.159
9841944,-0.32527143\C,0,-6.2914678515,-2.654681306,0.5768154063\H,0,-5
.5539320051,-2.1871649934,2.5563357601\C,0,-4.6999023517,-2.5316979566
,-1.2769824386\C,0,-5.9902481493,-2.779710973,-0.7882544016\H,0,-7.300
2395858,-2.8634419935,0.9193757008\H,0,-6.7674798157,-3.0735515808,-1.
4868810558\O,0,-2.3936995381,-1.8554078476,-0.5457907905\C,0,-1.618045
4208,-2.4821853526,-1.7113506966\H,0,-0.5977175742,-2.4892248057,-1.32
6490764\H,0,-2.0032425527,-3.5002719859,-1.7889136576\C,0,-1.787398754
4,-1.6634621563,-2.9400828207\C,0,-2.1841601219,-2.1914067066,-4.10294
97121\H,0,-1.4839713027,-0.6203539998,-2.8760382301\H,0,-2.2112110228,
-1.5967808542,-5.0113299074\H,0,-2.4704167723,-3.2375151304,-4.1970177
034\Au,0,0.9220040696,-2.7554297392,0.8986833728\H,0,-4.4687719231,-2.

```
6135737899,-2.3313266642\\Version=EM64L-G09RevA.01\State=1-A\HF=-983.8
273646\RMSD=1.532e-09\Dipole=-1.5659027,0.1481195,-1.6675028\Quadrupol
e=3.8439253,-19.0393625,15.1954372,0.3615893,4.0992982,6.3245877\PG=C0
1 [X(C20H18Au1O1)]\\@
```

***Ortho*-substituent Cl-D**

```
1\1\GINC-SHI_02\SP\RB3LYP\GenECP\C20H17Au1Cl1O1(1+)\YIN\21-Jan-2015\0\
\#p geom=check b3lyp/genecp\\vdcp_ph_cl_int1-sp\\1,1\C,0,-0.004035312,
-0.0708775027,0.0063375103\C,0,0.0892118949,-0.0369879046,1.5312971443
\H,0,0.9099466514,-0.0779026515,-0.5846287005\H,0,-0.8379793352,0.4558
807743,-0.4500912968\H,0,-0.6794935817,0.5197773463,2.0605300486\H,0,1
.0684367921,-0.0260097626,2.0062025375\C,0,-0.282303661,-1.290724798,0
.8154851606\C,0,-0.0546150037,-2.6291221334,0.8300620614\C,0,0.3510871
538,-3.8868899274,0.6967756078\C,0,-0.6448743559,-4.990359359,0.813373
112\C,0,-0.6580957333,-6.0222165533,-0.131039012\C,0,-1.664258478,-4.9
531198531,1.803968312\C,0,-1.701668565,-6.9551035001,-0.1398710314\H,0
,0.1219638697,-6.0733232324,-0.885145012\C,0,-2.7090869349,-5.89363565
86,1.7823707694\H,0,-1.5164541588,-4.3491668756,2.6999804142\C,0,-2.73
84542639,-6.8819209246,0.7973489738\H,0,-1.7115239264,-7.7348979629,-0
.8963600512\H,0,-3.461639146,-5.876835083,2.5659108983\H,0,-3.54413184
2,-7.6096578293,0.7771193386\C,0,1.780059616,-4.1338242836,0.335159779
1\C,0,2.2920862434,-3.6425404926,-0.8726903491\C,0,2.6032861814,-4.854
9862269,1.2353255211\C,0,3.6193392624,-3.8853357305,-1.224492827\H,0,1
.6357021364,-3.0990799249,-1.5466761114\C,0,3.9531419429,-5.0467828512
,0.8805094176\C,0,4.444244266,-4.5834157195,-0.3448871913\H,0,4.013544
9726,-3.5279971355,-2.1705881895\H,0,5.4878848045,-4.7576352042,-0.586
6716092\O,0,2.0222505769,-5.2112347833,2.4113756721\C,0,2.2086267778,-
6.5442756582,2.9801735429\H,0,2.3130004086,-7.274993361,2.1710248781\H
,0,3.1254426274,-6.5401776414,3.5746961311\C,0,1.0242860634,-6.8430701
669,3.8466779887\C,0,0.3030928332,-7.9640145962,3.7593050633\H,0,0.810
3300446,-6.1077438958,4.6222698175\H,0,-0.5035021618,-8.1770894745,4.4
557503092\H,0,0.5080454592,-8.7222849869,3.0057583517\Au,0,-2.21869057
99,-2.4211054672,1.1576316747\Cl,0,5.0981140658,-5.7875472455,1.977442
968\\Version=EM64L-G09RevA.01\State=1-A\HF=-1443.4227755\RMSD=3.837e-0
9\Dipole=-1.4341699,0.8906827,-0.6650788\Quadrupole=2.3433262,9.923984
,-12.2673102,-0.797582,-7.0108965,-2.8569845\PG=C01 [X(C20H17Au1Cl1O1)
```

***Ortho*-substituent Cl-E**

```
1\1\GINC-SHI_03\SP\RB3LYP\GenECP\C20H17Au1Cl1O1(1+)\YIN\04-May-2015\0\
\#p b3lyp/genecp geom=check\\Title Card Required\\1,1\C,0,-0.252400404
3,0.237578444,-0.118110664\C,0,-0.2050920667,0.2244377734,1.3833059928
\H,0,0.6863980728,0.3199851099,-0.6564614546\H,0,-1.09452666,0.7236907
979,-0.6062334804\H,0,-1.0128470952,0.6965899819,1.935604208\H,0,0.764
3258533,0.2905409569,1.865438054\C,0,-0.5381673866,-1.0597551688,0.631
5398228\C,0,-1.8977909502,-1.5493605858,0.724495816\C,0,-2.802326416,-
```

1.7284388272,1.7230425162\C,0,-2.5553873847,-1.5166333393,3.1641790519
\C,0,-3.4424669202,-0.731071971,3.9241240725\C,0,-1.4436990245,-2.0995
6254,3.7958122347\C,0,-3.2153359055,-0.531422406,5.286468282\H,0,-4.29
34077154,-0.2535904821,3.4458015138\C,0,-1.2278033277,-1.9075072952,5.
1609943644\H,0,-0.7570810575,-2.7139938784,3.2201686084\C,0,-2.1110039
099,-1.1225318574,5.9086272884\H,0,-3.8994249331,0.086549963,5.8610798
3\H,0,-0.3710442093,-2.3719941983,5.6410011937\H,0,-1.9395209552,-0.97
13139358,6.9705521524\C,0,-4.0857652472,-2.1166354271,1.1460396153\C,0
,-5.3299636026,-2.3989807463,1.7206564555\C,0,-3.9479188605,-2.1525349
471,-0.2427216747\C,0,-6.4015418938,-2.6775640338,0.8712558341\H,0,-5.
455229936,-2.3991187996,2.7977171025\C,0,-5.0134571573,-2.3676892691,-
1.1082946421\C,0,-6.2545286736,-2.6466165009,-0.5208603667\H,0,-7.3768
288633,-2.9051409242,1.2901986404\H,0,-7.1064852929,-2.8266112989,-1.1
682734671\O,0,-2.5958494038,-1.8540984398,-0.5802818065\C,0,-1.8386468
883,-2.8861565479,-1.5829602249\H,0,-1.24190226,-3.4549003671,-0.87063
66117\H,0,-2.6658265381,-3.4632639834,-1.9847047405\C,0,-1.0632157432,
-2.1411237375,-2.5895257088\C,0,-1.3682030364,-2.186743777,-3.89380507
83\H,0,-0.174842268,-1.6210390435,-2.2450327118\H,0,-0.7340120777,-1.7
075446683,-4.6343058068\H,0,-2.240171644,-2.7166764218,-4.2678093033\A
u,0,0.9155948406,-2.5272430654,0.6370841888\Cl,0,-4.8830941134,-2.2518
470016,-2.8410065455\\Version=EM64L-G09RevA.01\State=1-A\HF=-1443.4372
985\RMSD=4.571e-09\Dipole=-1.2397639,-0.0315469,-0.6847163\Quadrupole=
7.4788458,-19.7025565,12.2237107,1.5117065,-1.5485378,5.8404306\PG=C01
 [X(C20H17Au1Cl1O1)]\\@

***Ortho*-substituent Br-D**

1\1\GINC-SHI_02\SP\RB3LYP\GenECP\C20H17Au1Br1O1(1+)\YIN\21-Jan-2015\0\
 \#p geom=check b3lyp/genecp\\vdcp_ph_br_int1-sp\\1,1\C,0,0.0021813684,
0.0110828207,0.0268443894\C,0,-0.0433427119,-0.0602552698,1.5533258504
\H,0,0.9651049216,0.0217841599,-0.480457595\H,0,-0.7775601875,0.588048
2163,-0.4634673085\H,0,-0.8478755502,0.4774909499,2.0477748389\H,0,0.8
873252422,-0.1063419995,2.1157289714\C,0,-0.3681393124,-1.2533044439,0
.7211822378\C,0,-0.1613282596,-2.5964277957,0.6660018352\C,0,0.2573557
667,-3.8464264398,0.5155463953\C,0,-0.7353005966,-4.9581587271,0.48285
02423\C,0,-0.6503902637,-5.9412513262,-0.510597958\C,0,-1.8247447788,-
4.9908841671,1.3910124912\C,0,-1.6629425489,-6.8982178829,-0.641890325
4\H,0,0.1874591496,-5.9387207794,-1.2019211261\C,0,-2.8362902161,-5.95
49650447,1.2496180241\H,0,-1.778446795,-4.3999655491,2.3060167401\C,0,
-2.7641074778,-6.8978785006,0.2220780631\H,0,-1.5950258332,-7.64090477
44,-1.4318295849\H,0,-3.6478468619,-5.9909280476,1.9711820591\H,0,-3.5
44005355,-7.6453188154,0.1112123229\C,0,1.7226348042,-4.0752817038,0.3
210912591\C,0,2.3918173034,-3.5169840074,-0.7773538177\C,0,2.428515222
2,-4.8291989789,1.2864602997\C,0,3.7616334567,-3.7209851764,-0.9410168
927\H,0,1.830002111,-2.9464107628,-1.5118266326\C,0,3.8178621606,-4.97
72246689,1.1298031014\C,0,4.4732826362,-4.4440572889,0.016837535\H,0,4

.2792380421,-3.3089464689,-1.8017951097\H,0,5.5450662417,-4.5805849878,-0.0826950034\O,0,1.7395865887,-5.2688035352,2.3778466096\C,0,1.577404305,-6.7091226536,2.5634431066\H,0,0.7633397845,-7.0501613714,1.9132669336\H,0,2.5023228826,-7.213148832,2.2664874494\C,0,1.2702316759,-6.9602542305,4.0056266912\C,0,0.1920041064,-7.6269158599,4.4269292144\H,0,2.007563592,-6.5909032741,4.7168393737\H,0,0.026543767,-7.8335227559,5.4806875432\H,0,-0.5542848629,-8.0085114839,3.7319990484\Au,0,-2.3381124804,-2.3395262443,0.796338657\Br,0,4.8647157537,-5.8288553037,2.4725429079\\Version=EM64L-G09RevA.01\State=1-A\HF=-3557.3434276\RMSD=6.057e-09\Dipole=-2.0595617,1.3764438,-1.1572044\Quadrupole=5.8930697,8.3451019,-14.2381715,-1.478071,-3.3871242,-3.2254692\PG=C01 [X(C20H17Au1Br1O1)]\\@

***Ortho*-substituent Br-E**

1\1\GINC-SHI_03\SP\RB3LYP\GenECP\C20H17Au1Br1O1(1+)\YIN\04-May-2015\0\\#p b3lyp/genecp\\Title Card Required\\1,1\C\C,1,1.5025428\H,1,1.08538529,2,117.98956451\H,1,1.08790442,2,118.48846351,3,-145.15943791,0\H,2,1.08652561,1,118.78233026,3,145.30378633,0\H,2,1.08470289,1,118.16474507,3,-0.5076061,0\C,1,1.52463478,2,60.4939577,6,107.65746473,0\C,7,1.44809196,1,119.65893937,2,105.76009813,0\C,8,1.35883803,7,135.72655741,1,-114.46071101,0\C,9,1.47741828,8,125.96210286,7,-4.24043412,0\C,10,1.40773789,9,120.05377935,8,130.09879679,0\C,10,1.40516083,9,120.78151333,8,-50.26495902,0\C,11,1.39542982,10,120.28295509,9,179.83144066,0\H,11,1.08664591,10,120.07702785,9,-1.9026579,0\C,12,1.39542742,10,120.3425188,9,-179.1890488,0\H,12,1.08638932,10,119.75145888,9,-0.03228924,0\C,15,1.39828578,12,120.1684638,10,-0.63314239,0\H,13,1.08630134,11,119.68835121,10,179.23805928,0\H,15,1.08636359,12,119.70455904,10,179.3019932,0\H,17,1.08626834,15,120.07818161,12,-179.75544579,0\C,9,1.45979257,8,109.23711,7,173.34298652,0\C,21,1.39903586,9,132.19424155,8,-179.06227637,0\C,21,1.39623264,9,108.34926352,8,-1.14189495,0\C,22,1.39528649,21,118.22195518,9,176.24518904,0\H,22,1.08428833,21,120.69063439,9,-3.64744001,0\C,23,1.38800003,21,123.27415195,9,-173.40630601,0\C,24,1.40009739,22,121.26249632,21,-1.69264885,0\H,24,1.08561355,22,119.83538485,21,179.67300649,0\H,27,1.08473181,24,120.17664606,22,-177.28559755,0\O,23,1.42775166,21,108.81337941,9,3.14609839,0\C,30,1.6237097,23,116.51119639,21,126.40420764,0\H,31,1.08987074,30,100.54531326,23,-99.06552846,0\H,31,1.08603062,30,102.30156069,23,16.86296134,0\C,31,1.473666,30,110.37472509,23,139.16566644,0\C,34,1.33983496,31,121.775302,30,-117.89111909,0\H,34,1.08535261,31,117.20847862,30,68.05544167,0\H,35,1.08635246,34,121.01933619,31,-173.75016454,0\H,35,1.08662589,34,122.2481685,31,4.77518047,0\Au,7,2.06625138,1,118.35621574,2,-105.22817607,0\Br,26,1.89614359,23,124.08823175,21,172.49219469,0\\Version=EM64L-G09RevA.01\State=1-A\HF=-3557.3564973\RMSD=2.140e-09\Dipole=-0.8474309,0.0324004,-0.2829039\Quadrupole=7.0400093,-19.800931,12.7609217,-1.0765958,-1.3366211,6.2902306\PG=C01 [X(C20H17Au1Br1O1)]\\@

***Ortho*-substituent *t*Bu-D**

1\1\GINC-SHI_02\SP\RB3LYP\GenECP\C24H26Au1O1(1+)\YIN\21-Jan-2015\0\\#p
 b3lyp/genecp geom=check\\vdcp_ph_bu_int1-sp\\1,1\C,0,0.6582720244,-0.
2536740574,0.2951632059\C,0,0.7476288987,-0.4196010169,1.8132967952\H,
0,1.5350401603,-0.4484941946,-0.3195219254\H,0,0.0060721326,0.53237053
6,-0.0760421535\H,0,0.1573731233,0.2645474014,2.4169192327\H,0,1.68634
36697,-0.7339452622,2.2653068953\C,0,0.0520100015,-1.4339016768,0.9720
694229\C,0,-0.0541355207,-2.786862228,0.8267915236\C,0,0.0833998802,-4
.0762222267,0.5668373223\C,0,-1.0868764871,-4.9942485388,0.5678371156\
C,0,-1.2253487655,-5.9531616041,-0.4461237827\C,0,-2.1065638256,-4.875
0057454,1.541731896\C,0,-2.3862064512,-6.7280182969,-0.5273467296\H,0,
-0.4397072763,-6.0723612,-1.18640779\C,0,-3.2681621369,-5.6540233229,1
.4515453189\H,0,-1.9226001104,-4.2961055082,2.4465274339\C,0,-3.415703
8045,-6.5704940987,0.4076876352\H,0,-2.4893760248,-7.4523486425,-1.330
3703621\H,0,-4.0322723638,-5.5713187733,2.2196567107\H,0,-4.3134212035
,-7.1776186972,0.3372815715\C,0,1.472291527,-4.5060831284,0.1793337232
\C,0,1.9288129986,-4.2465471352,-1.1166605183\C,0,2.3072588441,-5.1286
782878,1.1395192624\C,0,3.2131394734,-4.6531121978,-1.4706044899\H,0,1
.2792764324,-3.7534840362,-1.8352106684\C,0,3.6516222072,-5.4442081175
,0.8305978135\C,0,4.0407384198,-5.227151025,-0.5088426871\H,0,3.579739
6158,-4.5001111004,-2.4814299923\H,0,5.0450987588,-5.5002867031,-0.810
3646248\O,0,1.7421512423,-5.3180127566,2.383699549\C,0,1.2284103948,-6
.6466599332,2.6562649698\H,0,0.4590946257,-6.9070375292,1.9190207302\H
,0,2.0476954346,-7.3706789082,2.5598495351\C,0,0.6726795321,-6.6571935
452,4.0478218155\C,0,-0.531452534,-7.1403592519,4.3662579441\H,0,1.334
4908989,-6.2751919796,4.8242326427\H,0,-0.8718342155,-7.1817822528,5.3
973986361\H,0,-1.2108800385,-7.5330462736,3.6117540631\Au,0,-2.0811988
851,-1.989985103,1.1749511816\C,0,4.7255483519,-5.9832228149,1.8144597
986\C,0,4.4123472347,-5.7284387087,3.307297213\C,0,4.9145337962,-7.503
1238285,1.5814426898\C,0,6.0812045957,-5.274651404,1.5411149385\H,0,4.
1722368009,-4.6774938538,3.499617343\H,0,3.5920191136,-6.3373477328,3.
6873809438\H,0,5.2998716723,-5.9833545829,3.8971773349\H,0,5.217563008
1,-7.7159699296,0.5498151015\H,0,5.6939244274,-7.8912246638,2.24805615
93\H,0,3.9946109146,-8.0644658087,1.7807552575\H,0,6.8159218344,-5.605
3789287,2.282939362\H,0,6.5011355075,-5.5049916684,0.5579291905\H,0,5.
9855815211,-4.1856935324,1.6252581593\\Version=EM64L-G09RevA.01\State=
1-A\HF=-1141.0915927\RMSD=6.181e-09\Dipole=-1.1913416,1.1878979,-0.533
3119\Quadrupole=10.2066532,4.9316041,-15.1382573,-4.7308963,-2.5682979
,0.2244506\PG=C01 [X(C24H26Au1O1)]\\@

***Ortho*-substituent *t*Bu-E**

1\1\GINC-SHI_03\SP\RB3LYP\GenECP\C24H26Au1O1(1+)\YIN\04-May-2015\0\\#p
 b3lyp/genecp geom=check\\Title Card Required\\1,1\C,0,-0.1591696172,-
0.1107347167,-0.0862356968\C,0,0.0213550102,-0.0003068043,1.4033144845

\H,0,0.7234042213,-0.1678414751,-0.718015708\H,0,-1.0027463627,0.39894
20425,-0.5391075438\H,0,-0.6949434533,0.5901852117,1.9648781874\H,0,1.
0350321978,0.0319108925,1.7972304544\C,0,-0.4516664264,-1.3146692213,0
.8065120074\C,0,0.460073166,-2.4375728777,0.7236021097\C,0,1.982988168
2,-4.1012486173,0.2629030442\C,0,2.736238286,-5.0976983862,-0.35881988
59\C,0,2.2189811395,-3.7910178937,1.6062179228\C,0,3.7138327105,-5.729
3877989,0.4077439607\H,0,2.5585525036,-5.3678102247,-1.3940094539\C,0,
3.2750804592,-4.2801359672,2.3817186508\C,0,3.9794416202,-5.3123253084
,1.714077479\H,0,4.7982170741,-5.7911916137,2.2368886537\O,0,1.1853530
748,-2.8315113487,2.0041241639\C,0,0.2446434979,-3.3888580846,3.126167
3769\H,0,-0.5035682018,-3.9784699351,2.594267434\H,0,0.9282505996,-4.0
364582018,3.668353636\C,0,-0.3238638657,-2.310921547,3.9734769244\C,0,
-1.6089622528,-2.3140836076,4.3428944053\H,0,0.3658817087,-1.579671183
6,4.3860361703\H,0,-1.9878856416,-1.5940935426,5.0624300703\H,0,-2.318
4918029,-3.0464162032,3.9655710708\C,0,3.7899875563,-3.7919557251,3.76
05183902\C,0,3.3936240606,-2.3273294066,4.044158031\H,0,3.8784940379,-
1.9929706433,4.9671856447\H,0,3.7200532871,-1.6608546654,3.2373342393\
H,0,2.3219699186,-2.190985171,4.186275106\C,0,3.2889039232,-4.71913364
24,4.8955330938\H,0,3.7357629341,-4.4131538104,5.8483267963\H,0,2.2010
438552,-4.6905459426,5.0259866019\H,0,3.573521093,-5.7604797451,4.7098
504122\C,0,5.3425088316,-3.8287437556,3.7721591222\H,0,5.7458640375,-4
.8450365428,3.7514859738\H,0,5.7648881805,-3.271689365,2.9287147218\H,
0,5.701686012,-3.3656234273,4.6970887068\C,0,0.9432364047,-3.225857702
5,-0.266999049\Au,0,-2.4448899119,-1.817129819,1.0112732384\H,0,4.3079
843427,-6.5299561265,-0.022598783\C,0,0.5499706618,-3.1702017321,-1.69
21159364\C,0,1.5385581761,-3.0461328739,-2.6865039348\C,0,-0.798502506
4,-3.2512677223,-2.075185858\C,0,1.1796177202,-2.9966629637,-4.0342260
352\H,0,2.585150685,-2.964828266,-2.4053572439\C,0,-1.1521630165,-3.21
58525505,-3.4248254033\H,0,-1.5697082555,-3.3512959472,-1.3164940741\C
,0,-0.1654103727,-3.0861761429,-4.4067391368\H,0,1.9499683039,-2.88828
17819,-4.7925011161\H,0,-2.1981567339,-3.2893139746,-3.7091079734\H,0,
-0.4426644562,-3.0538382838,-5.4565758381\\Version=EM64L-G09RevA.01\St
ate=1-A\HF=-1141.1060789\RMSD=8.038e-09\Dipole=1.2593495,-0.6247013,0.
9896444\Quadrupole=5.4658234,-15.2367501,9.7709268,-11.2905557,4.73069
04,0.1944192\PG=C01 [X(C24H26Au1O1)]\\@

3k-D

1\1\GINC-SHI_03\FOpt\RB3LYP\GenECP\C28H45Au1O1P1(1+)\YIN\06-May-2015\0
\\#p opt b3lyp/genecp\\Title Card Required\\1,1\C,-0.0245313703,-0.048
5183956,0.0666338728\C,0.0044794027,-0.0523803254,1.4851234223\C,1.242
8599113,-0.025243821,2.2487687142\C,1.3174648032,-0.8690396534,3.39116
09986\C,2.4018104867,0.7160418923,1.8622504413\C,2.5093500881,-1.08318
51104,4.0673664771\H,0.4346923028,-1.4221105348,3.6820315985\C,3.63614
03887,0.5160949114,2.5228360486\C,3.6411577256,-0.3898942943,3.5941079
493\H,4.5779358546,-0.5553444021,4.1096079721\O,2.3364885485,1.6452488

784,0.8580095414\C,1.4769311975,2.8020176572,1.0778009529\H,0.44092188
2,2.4517474902,1.1750503272\H,1.7657235151,3.2780513195,2.0200476778\C
,1.6165018652,3.7035595576,-0.1062795026\C,1.880066606,5.0233506098,-0
.1078581258\H,1.4655162931,3.2028781075,-1.0628238379\C,4.9403358092,1
.2308054845,2.0857969064\C,5.2600358738,0.8808833289,0.6103238718\H,6.
1879282566,1.3790257474,0.3046650554\H,5.4111910847,-0.199378089,0.490
0307247\H,4.4651651874,1.2042118666,-0.065274249\C,4.8116508443,2.7659
276281,2.2448889866\H,5.7786773969,3.2374556587,2.0333061211\H,4.08166
87528,3.1894044244,1.5538326873\H,4.5295916296,3.034217568,3.270498676
\C,6.1524057558,0.7854998715,2.9337165644\H,6.0439605038,1.0520612838,
3.9918160883\H,6.3416863474,-0.2920286713,2.8613770956\H,7.0485566037,
1.2947405996,2.563671495\C,2.6349737789,-2.0279254496,5.2772411763\C,3
.6506542901,-3.1512878029,4.9537059647\C,3.1315852539,-1.2226100958,6.
5035783489\C,1.2904888972,-2.6850394243,5.6459585825\H,3.3220626336,-3
.7460611342,4.0930742141\H,4.6467806246,-2.7545041399,4.7288307919\H,3
.7516076591,-3.8257829084,5.8120255802\H,2.4268760419,-0.4243981662,6.
7650339021\H,3.2313253642,-1.8844780632,7.3717462731\H,4.1100750293,-0
.7632750219,6.3265468799\H,1.427604658,-3.3346454252,6.5171096773\H,0.
5281611084,-1.9425125658,5.9123809059\H,0.8998246662,-3.3103018147,4.8
337450689\C,-1.3078139485,-0.0631075506,2.2187428152\H,-1.6853489474,0
.9693348026,2.255090566\H,-2.0579758421,-0.6539274385,1.683571876\H,-1
.2240702088,-0.398380545,3.253317711\Au,-1.6543990547,0.712086464,-1.0
245311261\C,0.9640099775,-0.6608789669,-0.6378464852\C,2.0918859811,-1
.6043809463,-0.6712133584\C,1.4879523477,-1.0106374384,-1.9558876594\H
,3.0740252335,-1.2569427784,-0.3559610514\H,1.8973789555,-2.6532491718
,-0.4491972543\H,0.9001751492,-1.6646521187,-2.5995046842\H,2.06308100
95,-0.2565710469,-2.4942838365\C,2.1065672576,5.8711517783,1.120603129
8\H,3.0501980491,6.4252048419,1.031359571\H,1.3127395346,6.6248881653,
1.2155581734\H,2.1365688304,5.3026304843,2.0533331421\C,1.9564289324,5
.7839223905,-1.4109519397\H,1.2228241379,6.6015479387,-1.4322184599\H,
2.944455349,6.2489550307,-1.5303673552\H,1.7767449446,5.1380642728,-2.
2763241478\P,-3.4443060201,1.5651022811,-2.3022501649\C,-3.546953466,0
.8696608787,-4.0034714258\H,-3.665701309,-0.217132639,-3.9545528652\H,
-4.3972565973,1.2974671879,-4.5464234929\H,-2.6262422151,1.0904388083,
-4.5522785668\C,-5.107696629,1.2491507906,-1.5793055069\H,-5.177895462
2,1.7093067283,-0.5888558601\H,-5.8925755999,1.6641635401,-2.221675108
6\H,-5.2688754546,0.1721925227,-1.4707699182\C,-3.3970693516,3.3879075
617,-2.5547125654\H,-2.4751169852,3.6684798604,-3.0731744321\H,-4.2561
035907,3.7183914374,-3.1494149878\H,-3.4155392181,3.8979733539,-1.5867
136893\\Version=EM64L-G09RevA.01\State=1-A\HF=-1646.2071328\RMSD=3.681
e-09\RMSF=3.946e-06\Dipole=-2.0004373,0.2448647,-0.9211799\Quadrupole=
11.8728399,-13.3152465,1.4424066,-6.5439586,28.7230755,-12.1280389\PG=
C01 [X(C28H45Au1O1P1)]\\@

3k-TS1

1\1\GINC-SHI_03\FTS\RB3LYP\GenECP\C28H45Au1O1P1(1+)\YIN\07-May-2015\0\
\#p opt=(calcfc,ts,noeigen) b3lyp/genecp\\3k_au_pme3_ts2\\1,1\C,0.6705
199483,-0.5278638143,0.411936782\C,-0.2475798797,-1.260935765,-0.30505
1458\C,-1.7012601127,-0.972806008,-0.2136104693\C,-2.6202966173,-2.015
6828077,-0.4422427494\C,-2.2177597408,0.3013992824,0.1249369884\C,-3.9
961313832,-1.836466814,-0.3044362137\H,-2.2358573054,-2.9900943898,-0.
7101061503\C,-3.5947807362,0.540116237,0.3042241281\C,-4.4390946496,-0
.5630511423,0.0816147497\H,-5.5019157214,-0.4239973105,0.2206966358\O,
-1.3017556957,1.371370373,0.2850033349\C,-0.7969959337,1.8836342957,-1
.0145175839\H,-0.2000693525,1.078060088,-1.4559471066\H,-1.672532526,2
.0466554564,-1.6430455737\C,0.0222751426,3.11047112,-0.7899666855\C,-0
.11328247,4.3039549263,-1.3997654457\H,0.8542699081,2.9829074564,-0.09
95913564\C,-4.1964490224,1.8928003663,0.7767154431\C,-3.6167269687,2.2
828441017,2.1595066132\H,-4.0991674266,3.1999741152,2.5177787474\H,-3.
8045597555,1.4978717532,2.9023307043\H,-2.542315569,2.4754170082,2.107
0401893\C,-3.9089621694,3.0313392359,-0.2307538098\H,-4.4711267391,3.9
269574548,0.05894331\H,-2.853207853,3.3060120477,-0.2479606602\H,-4.22
49928873,2.7569878511,-1.2446361354\C,-5.7319323978,1.8116768685,0.937
5362079\H,-6.2404478889,1.596645645,-0.0093593477\H,-6.0373027414,1.06
26823894,1.6769253768\H,-6.1007754674,2.7808862256,1.2892782875\C,-5.0
159458575,-2.9661516048,-0.5438618292\C,-5.7975018234,-3.2410406067,0.
7648774902\C,-6.0062708488,-2.5368046278,-1.6544080187\C,-4.3406287618
,-4.2794419093,-0.9854038643\H,-5.1216596428,-3.5518567946,1.570494947
8\H,-6.3479054991,-2.3585256176,1.1089125441\H,-6.526888288,-4.0441374
552,0.6064923632\H,-5.4817562944,-2.3377413999,-2.5965281482\H,-6.7368
870588,-3.3338604807,-1.8360584459\H,-6.5648361777,-1.6341540132,-1.38
34163078\H,-5.1057081151,-5.0447118453,-1.1555559972\H,-3.7843104798,-
4.1610540831,-1.9232804505\H,-3.6563928182,-4.6690649366,-0.2217992341
\C,0.189979844,-2.3619882579,-1.2397579806\H,1.2795341276,-2.403418907
6,-1.3036606556\H,-0.1593002654,-3.3403344446,-0.8847671176\H,-0.21707
38753,-2.2259276805,-2.2493019934\Au,2.7550537853,-0.6382547957,0.1813
01347\C,0.1902203769,0.3973234257,1.3623377342\C,-0.3664008978,0.36294
79765,2.7291328523\C,0.6933426289,1.3943409597,2.3174299381\H,-1.36039
69451,0.7544138413,2.9195071092\H,-0.0968349859,-0.5047688091,3.327607
1537\H,1.7297437304,1.2984067575,2.6358780467\H,0.3319607047,2.4154938
408,2.2370920181\C,-1.1629395763,4.6508650659,-2.4268800312\H,-1.72327
72123,5.5414109662,-2.1133155947\H,-0.6841677379,4.9064996268,-3.38185
09957\H,-1.8812392902,3.8512840234,-2.6209175605\C,0.8514640915,5.4259
648086,-1.0969932069\H,1.3811394573,5.7418229242,-2.0059405037\H,0.313
5567958,6.3093451796,-0.7272217092\H,1.5968110936,5.1401623484,-0.3480
707435\P,5.092295929,-0.7915574507,-0.0907627561\C,5.8025608148,-2.413
4752966,0.4126307882\H,5.3542565028,-3.2164733123,-0.1805408825\H,6.88
83612876,-2.4247154051,0.2653265916\H,5.5837247187,-2.6039786641,1.467
8361963\C,5.6602612914,-0.5607445746,-1.8267232593\H,5.3504091102,0.42
34046509,-2.1914902713\H,6.7513913433,-0.6382578613,-1.8911291062\H,5.

2104069301,-1.3226016053,-2.4708676023\C,6.0576050009,0.4476814351,0.8
695867246\H,7.1314751625,0.3236380235,0.6899379734\H,5.7626656885,1.45
96694945,0.5754031167\H,5.8600090477,0.3279278459,1.939277578\\Version
=EM64L-G09RevA.01\State=1-A\HF=-1646.1985137\RMSD=8.888e-09\RMSF=1.101
e-06\Dipole=2.2334105,0.2675576,-0.0642526\Quadrupole=38.6597265,-10.9
206217,-27.7391048,-4.3477874,-0.3410549,-0.3173523\PG=C01 [X(C28H45Au
1O1P1)]\\@

3k-I

1\1\GINC-SHI_03\FOpt\RB3LYP\GenECP\C28H45Au1O1P1(1+)\YIN\15-May-2015\0
\\#p opt b3lyp/genecp\\vdcp_ph_tbu_3a_au_int_h\\1,1\C,-0.1066641096,-0
.0327880373,0.0232059458\C,-0.0882774492,-0.0159875569,1.3863252124\C,
1.2058436423,-0.0043376446,2.1102856042\C,1.315453993,-0.4106909665,3.
4491556221\C,2.3956466757,0.3664416792,1.4590155899\C,2.5499123015,-0.
4558326535,4.1057783541\H,0.4156419268,-0.7016720507,3.9732172118\C,3.
6733379596,0.3281204993,2.0280672574\C,3.6876915964,-0.0938234973,3.37
28400225\H,4.6490926135,-0.1412441283,3.8697580921\O,2.196806675,0.897
3273353,0.1185749138\C,1.8381334998,2.4361522275,0.1800285305\H,0.8571
844643,2.4611354237,0.64740318\H,2.6061667542,2.766155437,0.8784872089
\C,1.9338614692,3.0874662174,-1.139121243\C,0.9242183097,3.7048019227,
-1.7914332982\H,2.934712319,3.1509752024,-1.5624927398\C,5.0441676055,
0.6734173199,1.3839541\C,5.0185341847,1.006422169,-0.1225992325\H,6.04
95081963,1.1626506871,-0.4587426799\H,4.5998023455,0.1947891875,-0.724
8185171\H,4.4707550446,1.9247784889,-0.3457957279\C,5.6507941955,1.893
6123949,2.1223137354\H,6.6353328532,2.1324899588,1.7034489218\H,5.0162
656342,2.7819933261,2.0143705523\H,5.7804694255,1.7057725924,3.1924136
235\C,5.9899229847,-0.5462980443,1.5472180991\H,6.1725647103,-0.804040
818,2.5941344682\H,5.5800822625,-1.4332540683,1.0501915179\H,6.9613513
394,-0.3216240936,1.092364533\C,2.6925569603,-0.8884743472,5.578125776
4\C,3.6124438456,-2.1316487901,5.6638982393\C,3.3153248982,0.271180924
8,6.394089674\C,1.3366244691,-1.250517253,6.2162273553\H,3.1988646649,
-2.9694858449,5.0902192085\H,4.6192490388,-1.9274991578,5.283367995\H,
3.7134947302,-2.4530358811,6.7071547945\H,2.6818744961,1.1654126924,6.
3571758136\H,3.423569949,-0.0232643599,7.4446166782\H,4.3090873702,0.5
472149123,6.0249062695\H,1.4918146021,-1.5438440325,7.2601913864\H,0.6
412016163,-0.4024936047,6.21675192\H,0.8546896583,-2.0949499699,5.7089
903426\C,-1.3480731693,-0.0955184869,2.2188937631\H,-2.2304118108,0.00
83877654,1.5822753161\H,-1.42967214,-1.0576943603,2.742374652\H,-1.382
6800649,0.6914980784,2.9834026349\Au,-1.8181814827,-0.3336015915,-1.12
3650793\C,1.2198539598,0.0398227287,-0.6409890936\C,1.8911639965,-1.16
09090788,-1.2704286538\C,1.500143556,0.027182624,-2.1136423375\H,2.948
7818394,-1.3170860931,-1.082722907\H,1.2944101806,-2.0667637195,-1.306
7723673\H,0.6321271256,-0.0383577479,-2.7635407969\H,2.3054316417,0.65
16300203,-2.4843297448\C,1.2023089995,4.4545495326,-3.0695904789\H,0.9
024647192,5.5065423707,-2.9732364603\H,2.2590872722,4.4237620755,-3.35

01270462\H,0.6109347513,4.0373763747,-3.895991323\C,-0.5107976409,3.74
97387568,-1.333909942\H,-1.1746952812,3.4897329096,-2.1678433257\H,-0.
737799209,3.0744304285,-0.5067361501\H,-0.77719441,4.7715555093,-1.030
8582357\P,-3.7807192187,-0.7586806154,-2.3761335118\C,-5.3192725252,-0
.0741295595,-1.6287693084\H,-6.1926235301,-0.3165166588,-2.2446936149\
H,-5.4638264997,-0.4915034937,-0.6274286645\H,-5.2381344965,1.01353767
34,-1.5379906491\C,-3.7832927829,-0.0864474554,-4.0916494003\H,-2.9502
796732,-0.511552247,-4.6601740484\H,-4.7221747135,-0.3283950579,-4.602
3167931\H,-3.663047661,1.00101518,-4.0656265416\C,-4.1520310919,-2.549
2061551,-2.6000848825\H,-5.0754347532,-2.6874304161,-3.1737737771\H,-3
.3272074349,-3.0366637012,-3.1290831663\H,-4.26334496,-3.0304391299,-1
.6234641919\\Version=EM64L-G09RevA.01\State=1-A\HF=-1646.2130415\RMSD=
7.389e-09\RMSF=3.908e-06\Dipole=-0.9221906,0.7236979,-1.615155\Quadrup
ole=18.3260258,-19.7396781,1.4136523,9.3688849,26.5355395,-4.9139946\P
G=C01 [X(C28H45Au1O1P1)]\\@

3k-TS2

1\1\GINC-SHI_02\FTS\RB3LYP\GenECP\C28H45Au1O1P1(1+)\YIN\14-May-2015\0\
\#p opt=(calcfc,ts,noeigen) b3lyp/genecp\\vdcp_ph_tbu_3a_au_int_h\\1,1
\C,0.6060304192,-0.297812244,0.1260366178\C,-0.2806742464,-1.161192187
8,-0.4573228424\C,-1.7299891709,-0.985112429,-0.2374275844\C,-2.657954
0644,-2.0077627167,-0.4911384816\C,-2.219107084,0.2510803776,0.2454275
891\C,-4.026838679,-1.8222790156,-0.2834253576\H,-2.2916311633,-2.9574
4389,-0.856480532\C,-3.5832364327,0.5014512115,0.4876753282\C,-4.44375
08134,-0.5723632178,0.2017584746\H,-5.5033804178,-0.4259359509,0.37452
66219\O,-1.2713180411,1.2724019415,0.374966063\C,-0.9653563023,2.05689
31538,-1.5572674538\H,-0.947003007,1.037977961,-1.9227662002\H,-1.9419
134912,2.5175622923,-1.4786823169\C,0.2079401303,2.8291903505,-1.65658
01037\C,0.2752406101,4.2033037562,-1.5669215407\H,1.1301405849,2.28705
05925,-1.8530747519\C,-4.2311374653,1.8037280503,1.0318110981\C,-3.247
569054,2.8918046882,1.5131408967\H,-3.8276587654,3.7305806583,1.914976
9414\H,-2.5923035497,2.5317701985,2.3120835026\H,-2.6184768742,3.28146
12068,0.7101304163\C,-5.1145752907,2.4222067182,-0.081212398\H,-5.6100
347804,3.3281329663,0.2883466496\H,-4.5159041015,2.7000409456,-0.95832
46876\H,-5.8921874659,1.7303562187,-0.4204183341\C,-5.1229141376,1.456
4276374,2.2536833211\H,-5.9403531219,0.7746038945,2.0041021331\H,-4.53
14006481,0.9933091672,3.0519565695\H,-5.5742708441,2.3716540002,2.6539
326496\C,-5.0693384883,-2.9216102444,-0.5637618579\C,-5.8289044349,-3.
2591944565,0.743372889\C,-6.0759540617,-2.4164226931,-1.6268862981\C,-
4.4252100755,-4.2188115586,-1.0916726937\H,-5.1413134124,-3.6205405395
,1.5171383695\H,-6.3624366372,-2.3913504235,1.1462003448\H,-6.57056979
57,-4.0446163898,0.5553342169\H,-5.5686114108,-2.1777698577,-2.5692827
768\H,-6.8261640361,-3.1891655479,-1.8326856457\H,-6.6093124708,-1.518
8416939,-1.2952314335\H,-5.205959477,-4.964017393,-1.2798874623\H,-3.8
925987324,-4.0594602319,-2.0370487509\H,-3.7254960675,-4.6547474961,-0

.3685922082\C,0.1634078276,-2.3540325443,-1.2734469042\H,1.241161559,-
2.313689779,-1.45189819\H,-0.0515658951,-3.2998581162,-0.7575285212\H,
-0.3443378539,-2.3967953019,-2.2459530637\Au,2.6666592444,-0.572609536
7,0.1203262312\C,-0.0037350363,0.8108916116,0.9048679138\C,0.073182721
7,0.8508486459,2.4264514491\C,0.7527828451,1.9097416513,1.596661678\H,
-0.8256299969,1.1286897592,2.9678787199\H,0.6737609854,0.0777705711,2.
8954356847\H,1.83536436,1.8946751315,1.5060354849\H,0.3002790749,2.896
8180414,1.5988003899\C,1.5866068884,4.8959839597,-1.7817848332\H,1.844
2864208,5.5031201709,-0.9022393524\H,2.4031652949,4.197172287,-1.97934
34565\H,1.514712392,5.5991802963,-2.6231909563\C,-0.8882455112,5.10079
49207,-1.2765711471\H,-1.0078669761,5.8315416359,-2.0878372011\H,-1.83
64270014,4.5800372491,-1.1398166491\H,-0.6850545433,5.6866016128,-0.36
92804814\P,5.002109816,-0.9722049365,0.1485759174\C,5.5890992959,-2.10
48270159,-1.1810207986\H,6.6716831447,-2.2598762244,-1.1109048942\H,5.
0847280418,-3.0722788275,-1.0941739317\H,5.3523371986,-1.6824873397,-2
.1625877352\C,6.0646808931,0.5215767665,-0.0425411201\H,5.8607935241,1
.2293052346,0.7671264368\H,7.1263648883,0.2509727491,-0.0193494193\H,5
.8447612017,1.0147125109,-0.9945774053\C,5.6150054737,-1.7555282907,1.
6996278956\H,6.6950051542,-1.9336237056,1.6466172396\H,5.4018500127,-1
.1063697743,2.5546294155\H,5.103522923,-2.7097652225,1.8595146232\\Ver
sion=EM64L-G09RevA.01\State=1-A\HF=-1646.2088856\RMSD=8.504e-09\RMSF=1
.536e-06\Dipole=2.0688518,2.2450131,-0.8447463\Quadrupole=29.8447293,2
.4454437,-32.2901729,-4.6475987,0.1482151,-6.6992138\PG=C01 [X(C28H45A
u1O1P1)]\\@

5k-P

1\1\GINC-SHI_02\FOpt\RB3LYP\GenECP\C28H45Au1O1P1(1+)\YIN\16-May-2015\0
\\#p opt b3lyp/genecp\\Title Card Required\\1,1\C,0.0714720505,-0.0285
237436,-0.0678326362\C,0.044505753,0.0319404807,1.3329882312\C,1.33221
20745,0.0439219233,2.0684306699\C,1.4131084532,-0.2549893175,3.4394084
485\C,2.4864945559,0.4879483024,1.3815397245\C,2.6055622874,-0.1030956
221,4.144238229\H,0.5248869757,-0.6052242081,3.9498938807\C,3.70587426
,0.7039429792,2.0549790371\C,3.7082901688,0.3847817252,3.4231361247\H,
4.6374865195,0.5281726217,3.9632306121\O,2.3597188475,0.7984877159,0.0
496197925\C,-1.1113352776,0.3659032329,-0.9508542809\H,-1.0361224313,-
0.1743165753,-1.9040370817\H,-2.0521786405,0.0436652473,-0.5021492301\
C,-1.1098305593,1.8623938863,-1.2003531577\C,-2.153000977,2.7084591534
,-1.1237458059\H,-0.1415933433,2.2742225634,-1.4841400456\C,5.02200496
72,1.2658520254,1.4527767601\C,4.9881193695,1.5874919669,-0.0569945129
\H,5.967098451,1.9894186667,-0.3431733143\H,4.8052428792,0.6983606928,
-0.667706674\H,4.231979021,2.3356266781,-0.3073509689\C,5.3729300048,2
.5848070608,2.1899218252\H,6.3093848418,2.9957194944,1.7944044527\H,4.
58771643,3.3362597602,2.0456175369\H,5.5050233823,2.4402149822,3.26715
04957\C,6.1593287053,0.2354720821,1.6738646683\H,6.3352398483,0.020673
7226,2.7327814158\H,5.9343297182,-0.7129652192,1.1711131187\H,7.096902

8128,0.6226188433,1.2579769137\C,2.7410525573,-0.4165838557,5.64680626
97\C,3.8470147317,-1.4786910246,5.8611087621\C,3.1178558886,0.87692503
42,6.4106805625\C,1.431076698,-0.9622438802,6.2484725825\H,3.606684060
6,-2.4115520083,5.3365729412\H,4.8233978377,-1.1355081491,5.5024509462
\H,3.9501155686,-1.7069261455,6.9286676485\H,2.3481671669,1.6475886754
,6.2849084714\H,3.2177209021,0.6690240106,7.4827772406\H,4.0695213289,
1.2951887766,6.0653324682\H,1.5768335631,-1.1757127113,7.3130923743\H,
0.6096861123,-0.2394983057,6.1723273832\H,1.1167156995,-1.8974697914,5
.7679406652\C,-1.1962123383,0.44720725,2.1011018423\H,-2.1184132199,0.
2851304917,1.5421594997\H,-1.2834572098,-0.0706396927,3.0590308507\H,-
1.1190377577,1.5207149998,2.319153901\Au,-0.3279920853,-2.24441242,0.7
194775409\C,1.4544378457,-0.0092211336,-0.684268594\C,2.0875007736,-1.
1894206846,-1.4036681484\C,1.7197753151,0.0647619125,-2.1597930373\H,3
.1371700321,-1.3770575602,-1.1956701197\H,1.4854065627,-2.0796663618,-
1.5707133051\H,0.88435673,0.0284314365,-2.8513151371\H,2.524208632,0.7
309582975,-2.4574367399\C,-1.9657463207,4.1777614609,-1.4254279811\H,-
2.2699397231,4.7947649153,-0.5687852756\H,-0.9262017454,4.4188603506,-
1.668315149\H,-2.5950197952,4.4858142664,-2.2715385801\C,-3.5702721965
,2.321618029,-0.7757407666\H,-4.2438968117,2.5631030519,-1.6091121267\
H,-3.6993201107,1.2597601281,-0.5486458621\H,-3.9273493638,2.900293445
8,0.0869834758\P,-0.7415574825,-4.5333397809,0.9063200621\C,-2.1450047
418,-4.9303747409,2.0233015835\H,-3.0622922102,-4.4633228407,1.6524155
622\H,-2.2921742838,-6.0148854523,2.0766891351\H,-1.9426275896,-4.5482
369006,3.0283413661\C,0.6885165568,-5.4804240781,1.5640661672\H,0.9538
364118,-5.1134661822,2.5599887483\H,0.4408998867,-6.5459367707,1.62860
98244\H,1.5543429405,-5.3537400441,0.90723731\C,-1.1557685281,-5.32995
56453,-0.6964358009\H,-1.3404594812,-6.4003059135,-0.5516968731\H,-2.0
506759445,-4.8682590385,-1.1242537105\H,-0.3289180141,-5.2039262541,-1
.4018726608\\Version=EM64L-G09RevA.01\State=1-A\HF=-1646.268264\RMSD=9
.148e-09\RMSF=3.197e-06\Dipole=-1.8398376,-2.9370625,-0.3773187\Quadru
pole=-3.633984,14.7871296,-11.1531456,11.5707245,7.5269361,0.2443347\P
G=C01 [X(C28H45Au1O1P1)]\\@

3k-TS1’

1\1\GINC-SHI_02\FTS\RB3LYP\GenECP\C28H45Au1O1P1(1+)\YIN\07-May-2015\0\
\#p b3lyp/genecp opt=(calcfc,ts,noeigen)\\3k_au_pme3_ts1\\1,1\C,1.4022
066062,-2.3574317281,2.4572460648\C,1.3726688638,-0.9278321415,2.74193
02566\H,0.5117743793,-2.9499200526,2.6447934735\H,2.3418866672,-2.8991
238058,2.4981945342\H,2.2890333531,-0.4206435248,3.0247730658\H,0.4611
850491,-0.4891501598,3.1361492901\C,1.323371869,-1.3355358851,1.219838
7484\C,0.071091923,-1.359250075,0.6382541511\C,-2.1720808883,-0.994159
5318,-0.2250120602\C,-3.3218149396,-1.4408900738,-0.8790110817\C,-2.17
36743567,0.2255362986,0.4802299753\C,-4.5007534224,-0.6869868862,-0.80
65851305\H,-3.2975747232,-2.3765963404,-1.4238536293\C,-3.366992774,0.
9186838892,0.7375434778\C,-4.488858484,0.4422814454,0.0245363893\H,-5.

4196958648,0.9839018773,0.1488023444\O,-0.9065643041,0.5773505284,0.95
04563192\C,-0.0821511278,1.3771744322,-0.010779377\H,0.898913171,1.374
6308859,0.459988984\H,-0.0342997939,0.785831453,-0.9319221868\C,-0.580
9263037,2.7492528027,-0.3152478526\C,-0.0066249353,3.9124967818,0.0525
329186\H,-1.4491664238,2.7922276666,-0.9706366013\C,-3.5975278045,2.04
66574557,1.7763242837\C,-4.0088916277,3.3595634746,1.0689164414\H,-4.2
336159542,4.1311593261,1.8152671476\H,-3.2056774336,3.7355992069,0.427
9929163\H,-4.9031944785,3.2277504541,0.4498114515\C,-4.7541414959,1.60
29093577,2.7148908677\H,-4.911655175,2.3667768349,3.4850837749\H,-5.70
42257335,1.4715865371,2.1889331714\H,-4.5139669869,0.6592404291,3.2183
410437\C,-2.3854014346,2.3256351705,2.6894684132\H,-2.054685922,1.4192
751076,3.2082169929\H,-1.5356754891,2.7383374785,2.14533169\H,-2.68183
88744,3.0555128629,3.452016805\C,-5.7918627595,-1.087060221,-1.5470693
567\C,-6.915351497,-1.3744617488,-0.5209090642\C,-6.2278788777,0.07302
95519,-2.4758390589\C,-5.5997081819,-2.34737931,-2.4137631036\H,-6.635
2139969,-2.1936118085,0.1519734692\H,-7.1457356918,-0.4981964313,0.094
5382162\H,-7.8361697096,-1.6634463954,-1.0412921955\H,-5.4558454153,0.
2929526696,-3.2229173527\H,-7.1473859593,-0.1964144453,-3.0088961694\H
,-6.4274504883,0.9940130021,-1.9172416513\H,-6.5338520465,-2.576974638
2,-2.9379122024\H,-4.8229879638,-2.2073770137,-3.1755331294\H,-5.34204
22485,-3.2271438295,-1.8116660661\C,-0.9411510474,-1.785138446,-0.0953
173226\C,-0.8272825336,-3.1693639424,-0.7162344912\H,-0.9425760342,-3.
0979582308,-1.8041869307\H,0.1402156164,-3.6297367679,-0.5040549025\H,
-1.6164690069,-3.8246430226,-0.3306312289\Au,3.0127690973,-1.047736703
2,-0.009886284\C,1.1929246751,4.0441690801,0.9585212879\H,2.017848566,
4.5452161342,0.4339959227\H,0.9433502834,4.6805369502,1.8181596121\H,1
.563870518,3.0927757876,1.3472210167\C,-0.5617977267,5.2277121883,-0.4
374123444\H,-0.8722739792,5.8568563064,0.4079883169\H,0.2054229168,5.7
942980884,-0.9823065757\H,-1.4209251938,5.0937782697,-1.1014416222\P,4
.9160788108,-0.7958066889,-1.3745279751\C,5.9103919844,-2.3347876375,-
1.5498477755\H,6.7773150611,-2.1631733173,-2.1976648637\H,6.2597951457
,-2.6666742894,-0.5672807091\H,5.2940590463,-3.1295157897,-1.981257439
\C,6.122762914,0.4611847691,-0.7805011599\H,6.9801379265,0.5230286337,
-1.4599544965\H,5.6420064258,1.4425856821,-0.7227493718\H,6.4792034055
,0.1935994366,0.2190010679\C,4.5472689762,-0.2908619993,-3.1061066786\
H,4.0278624947,0.6723935087,-3.1117408475\H,5.4721262332,-0.2007475071
,-3.6870106383\H,3.8994431303,-1.0341204267,-3.5809489749\\Version=EM6
4L-G09RevA.01\State=1-A\HF=-1646.1922843\RMSD=8.229e-09\RMSF=3.778e-06
\Dipole=2.6399791,-0.3865855,-0.7539638\Quadrupole=34.442973,-11.34805
76,-23.0949154,-5.3423566,-8.8873365,3.7683815\PG=C01 [X(C28H45Au1O1P1
)]\\@

3k-F

1\1\GINC-SHI_03\FOpt\RB3LYP\GenECP\C28H45Au1O1P1(1+)\YIN\06-May-2015\0
\\#p opt b3lyp/genecp\\Title Card Required\\1,1\C,-0.0608963017,-0.076

7533993,0.2591996954\C,-0.1079938392,-0.1446159016,1.7509452263\H,0.91
79600242,-0.0522394072,-0.2156363302\H,-0.8283006522,0.478093961,-0.27
21712644\H,-0.9030394323,0.3728933455,2.2794966486\H,0.8446610532,-0.1
348420419,2.2764476104\C,-0.484683119,-1.3938660614,0.9622848078\C,0.4
961754502,-2.4645142198,0.866459871\C,2.0779999121,-4.0677685357,0.367
9831565\C,2.876836648,-4.9986601908,-0.2957146327\C,2.3979031929,-3.67
827425,1.6678381795\C,3.9915847636,-5.5298781784,0.3674533192\H,2.6297
615161,-5.2890631949,-1.3097475768\C,3.5555622831,-4.0551094038,2.3513
161782\C,4.2967733501,-5.0316646634,1.6471099488\H,5.1951625993,-5.401
2260153,2.1247707698\O,1.3425961697,-2.7761053534,2.1176209186\C,0.498
0203934,-3.4420196782,3.2680307454\H,-0.1312347436,-4.1643963562,2.749
9819853\H,1.2850347838,-3.9549058227,3.8152376956\C,-0.2165334199,-2.4
416109535,4.0866696481\C,-1.4943907068,-2.5373658083,4.505148799\H,0.3
926631436,-1.6259369794,4.4683296722\C,4.1229085765,-3.4953294901,3.68
04146139\C,3.5493468019,-2.1063515519,4.0379803654\H,4.0907136368,-1.7
058167852,4.9017035014\H,3.6703211032,-1.3966184954,3.2117466576\H,2.4
950344343,-2.132347136,4.3158519052\C,3.8608792243,-4.4892135361,4.838
3891013\H,4.3197046365,-4.1175985234,5.7622057008\H,2.7918650579,-4.63
10917585,5.0382402294\H,4.2898472777,-5.4734971525,4.6209336503\C,5.65
78113962,-3.3040970954,3.5464275922\H,6.1993194894,-4.2486277544,3.443
9443299\H,5.9082119454,-2.6708973781,2.6880374729\H,6.0395436084,-2.81
59525411,4.4497173556\C,4.9065736007,-6.5918783211,-0.2731656097\C,6.3
345509204,-6.0149109292,-0.4379528419\C,4.9572606117,-7.8431234437,0.6
379091191\C,4.4031675045,-7.03065164,-1.6625096102\H,6.329132439,-5.12
48595065,-1.0781830558\H,6.7803825119,-5.7357608189,0.5228295567\H,6.9
900220377,-6.7613712537,-0.9018086125\H,3.9591195802,-8.2777858375,0.7
68919755\H,5.6028962294,-8.6078690622,0.1904842332\H,5.3578452605,-7.6
144550625,1.6315172242\H,5.0714958335,-7.7984245611,-2.0670131967\H,3.
396709779,-7.46404669,-1.617374486\H,4.3923409367,-6.2003359215,-2.378
7116662\C,0.938634548,-3.2922702261,-0.1019543632\C,0.4049050843,-3.34
28087782,-1.5017010004\H,0.1385126695,-4.3710342323,-1.7776469655\H,-0
.4837379447,-2.7165369268,-1.6089662879\H,1.1577449098,-2.9969480284,-
2.221665294\Au,-2.5056108218,-1.9413923311,0.6878893977\C,-2.455757605
6,-3.6375563553,4.1333301916\H,-2.6479159325,-4.2808822462,5.003100583
6\H,-3.4213232385,-3.2060932213,3.8427233548\H,-2.1122601248,-4.273461
0359,3.3137153502\C,-2.0504751135,-1.5145738578,5.4639472173\H,-2.9194
722789,-1.0096286719,5.0204767487\H,-2.403897717,-1.9947369276,6.38615
44353\H,-1.3118203719,-0.7547756589,5.7349199841\P,-4.7649958106,-2.43
65459272,0.2064254857\C,-5.1206519053,-2.5185815023,-1.5993994093\H,-4
.5227015098,-3.3100789309,-2.0617479798\H,-6.1823441323,-2.7225678635,
-1.7786681252\H,-4.85488808,-1.5682063524,-2.0724334997\C,-5.967753213
3,-1.1906670228,0.8356843745\H,-6.9901536917,-1.4545059867,0.542629529
3\H,-5.9139239206,-1.1346766981,1.9273603581\H,-5.7248018806,-0.203545
8052,0.4302773924\C,-5.4045972395,-4.0392057845,0.8543853918\H,-6.4478
43088,-4.1908629753,0.5550846275\H,-4.8004049741,-4.8654918196,0.46700

11751\H,-5.3447693935,-4.0525606293,1.9471276434\\Version=EM64L-G09Rev
A.01\State=1-A\HF=-1646.1995726\RMSD=8.361e-09\RMSF=2.852e-06\Dipole=-
1.6304464,-0.521404,0.4462497\Quadrupole=35.3230542,-24.3267693,-10.99
62849,-15.0621034,8.2781642,3.5468738\PG=C01 [X(C28H45Au1O1P1)]\\@

3k-TS2'

1\1\GINC-SHI_03\FTS\RB3LYP\GenECP\C28H45Au1O1P1(1+)\YIN\15-May-2015\0\
\#p opt=(calcfc,ts,noeigen) b3lyp/genecp\\Title Card Required\\1,1\C,1
.3132177214,0.349927896,2.9418060958\C,1.2073119704,1.6356455977,2.185
4285743\H,0.464353592,0.0514414295,3.5534607591\H,2.2775812193,0.05026
94633,3.3411868254\H,2.1006119325,2.2417069562,2.0650223421\H,0.295645
5113,2.2160787794,2.310089687\C,1.1286802276,0.3253370842,1.4069559963
\C,-0.1914614592,-0.1177689537,0.9571149653\C,-2.1526664161,-1.1317030
695,0.3644015493\C,-3.1727383814,-2.0589697359,0.1479934919\C,-2.38614
64007,0.2363401796,0.1969166555\C,-4.4369924982,-1.5968188607,-0.23331
43936\H,-2.9738254543,-3.1142147211,0.2925610064\C,-3.6551369418,0.781
7382859,-0.0487318586\C,-4.6342666112,-0.2042608611,-0.2976493507\H,-5
.6326677231,0.1443311911,-0.5244015947\O,-1.133657433,0.9186118512,0.4
474425946\C,-0.4318268545,1.6874382322,-0.9879261341\H,0.4758161037,1.
0973696976,-1.0596309478\H,-1.2179670607,1.3552874245,-1.6605422236\C,
-0.2852445889,3.1170613429,-0.8206164475\C,0.8875013961,3.8084862538,-
0.7965219671\H,-1.19880314,3.7007663315,-0.8209710583\C,-4.0689900108,
2.2712464707,-0.0086176065\C,-3.287650375,3.0295453223,1.0908388413\H,
-3.577158555,4.086824501,1.0874101815\H,-3.5328881164,2.6226155774,2.0
790076245\H,-2.2050602919,2.9783541442,0.9782613216\C,-3.8802740304,2.
9180191993,-1.4036791828\H,-4.165008048,3.9767255662,-1.3718024802\H,-
2.8530581345,2.8604051244,-1.7724230894\H,-4.5178360929,2.422105985,-2
.1442768679\C,-5.567103942,2.4275271879,0.3577469395\H,-6.2391163124,2
.081052847,-0.4336581961\H,-5.8162083996,1.8961877432,1.2827547321\H,-
5.7838777474,3.4902079415,0.5131229754\C,-5.6128566096,-2.5467181223,-
0.53299324\C,-6.7657481021,-2.2815377777,0.4665849276\C,-6.1159647139,
-2.298998158,-1.976631132\C,-5.2060639238,-4.0288413594,-0.4143103603\
H,-6.4385446658,-2.4464795294,1.4999435705\H,-7.1476900822,-1.25748934
11,0.3937994545\H,-7.6022711284,-2.9608230251,0.2641581829\H,-5.321377
873,-2.485560591,-2.7089467666\H,-6.9510793986,-2.971734254,-2.2048553
289\H,-6.4706386725,-1.2722460689,-2.118595787\H,-6.0663393707,-4.6643
168188,-0.6511546398\H,-4.4025203612,-4.2918188482,-1.1128975249\H,-4.
8815057635,-4.2866507795,0.6008888897\C,-0.8016746197,-1.3192071956,0.
864717038\C,-0.2243246757,-2.6355606215,1.2853222413\H,-0.2439377837,-
3.3529567137,0.4545514834\H,0.8099732729,-2.5266209196,1.619947504\H,-
0.8048806126,-3.0752390769,2.1065193663\Au,2.7639157984,-0.4122990427,
0.2921450771\C,2.2637860749,3.2144852815,-0.8844919377\H,2.6957212441,
3.4473897974,-1.8682000325\H,2.9217961888,3.6817395914,-0.1416761362\H
,2.2979572017,2.132933534,-0.7371422787\C,0.8531853397,5.3124147128,-0
.748264791\H,1.3664942866,5.6688802016,0.1553451326\H,1.3973055969,5.7

398907116,-1.6008274585\H,-0.1647433436,5.7107421779,-0.7488039946\P,4
.6189929053,-1.3383490606,-0.8495431116\C,4.4291477291,-3.1242753442,-
1.2603685526\H,3.5589555396,-3.2674140208,-1.9085666945\H,5.3212086986
,-3.5033709356,-1.7716584149\H,4.2724691929,-3.7009790268,-0.343548066
3\C,6.1934064265,-1.2654879541,0.1036393454\H,7.0127349718,-1.72444463
77,-0.4611250985\H,6.4498620064,-0.224132794,0.3218889508\H,6.07583696
44,-1.7949710155,1.0542088576\C,5.0395002998,-0.556700792,-2.465143924
6\H,5.9017177746,-1.0532421876,-2.9244686952\H,4.18573991,-0.626832202
4,-3.146391704\H,5.2774356224,0.5013808012,-2.3175671109\\Version=EM64
L-G09RevA.01\State=1-A\HF=-1646.1996897\RMSD=3.663e-09\RMSF=5.407e-07\
Dipole=1.8072194,0.6690852,-1.3775896\Quadrupole=31.6007613,-1.0594692
,-30.5412921,-5.8661343,-7.1866544,-2.3273488\PG=C01 [X(C28H45Au1O1P1)
]\\@

4k-P

1\1\GINC-A250\FOpt\RB3LYP\GenECP\C28H45Au1O1P1(1+)\SIOCWY\07-May-2015\
0\\#p opt b3lyp/genecp\\Title Card Required\\1,1\C,-0.1424554526,-0.22
13298551,-0.1583497522\C,-0.199044957,-0.2313963572,1.3603359916\H,0.8
411010762,-0.2149684765,-0.6205848103\H,-0.8852275131,0.3639200197,-0.
6929349413\H,-0.9746455547,0.3548109121,1.8447645195\H,0.7437232134,-0
.2547818149,1.9033341508\C,-0.5899279335,-1.4607830443,0.5790380857\C,
0.0565822519,-2.71053732,0.6397359512\C,1.0009638612,-4.8279555126,0.3
357602819\C,1.563695074,-6.0459649703,-0.0407067954\C,0.8615361047,-4.
5318930022,1.6807009548\C,1.9624422641,-6.9496737163,0.9574998044\H,1.
6872926289,-6.2790276513,-1.0903397808\C,1.2267373897,-5.365916044,2.7
391492067\C,1.7769285507,-6.5850665055,2.3082768281\H,2.0876118674,-7.
2902823672,3.0686211489\O,0.3144425285,-3.2325090881,1.829513639\C,-0.
665832238,-3.9667033766,-1.4607791004\H,-1.4598335913,-4.4474609994,-0
.8824320308\H,-1.0622286146,-2.9993387528,-1.8003157764\C,-0.295751179
2,-4.7887219471,-2.6659347295\C,-0.7266296411,-6.0240574732,-2.9864266
341\H,0.3551309164,-4.2890980843,-3.3835380648\C,1.0526698775,-4.98466
21936,4.2195641403\C,1.8940477262,-3.7218508479,4.5326365398\H,1.78567
11544,-3.4552633116,5.5906543735\H,2.9579567389,-3.9018650964,4.338760
0807\H,1.5777839267,-2.8600369936,3.9372674834\C,-0.4427856295,-4.7043
345738,4.5121336109\H,-0.5732278458,-4.4488945221,5.5704576451\H,-0.82
73559869,-3.8699923025,3.917628848\H,-1.0541965363,-5.5902938026,4.301
9824634\C,1.5174067821,-6.1126752413,5.1618495132\H,0.9443797986,-7.03
57581104,5.0155932478\H,2.5822493496,-6.3411163561,5.0387966084\H,1.37
02147929,-5.7972684275,6.2005726302\C,2.5985688246,-8.3146312651,0.624
9514477\C,4.0094784754,-8.3964224013,1.2588228634\C,1.713591837,-9.450
9400705,1.1946797315\C,2.7457837999,-8.5358538345,-0.8934459592\H,4.66
11938397,-7.602163437,0.876028886\H,3.9770897677,-8.3080702835,2.35000
92597\H,4.4740331617,-9.360315571,1.0194376638\H,0.7064231373,-9.42202
26146,0.761748684\H,2.155209088,-10.4266755818,0.9596703276\H,1.613409

7973,-9.3870936407,2.2834860221\H,3.2047098954,-9.5134513986,-1.077480
6384\H,1.7767818056,-8.5282637946,-1.4070612721\H,3.3898763002,-7.7800
473607,-1.3585951817\C,0.5185399318,-3.641117209,-0.4802054372\C,1.698
4072228,-2.9734322603,-1.2415270701\H,2.1665369823,-3.7164217383,-1.89
25382117\H,1.350005904,-2.1410232089,-1.8598552712\H,2.4657835459,-2.6
08527327,-0.5507868203\Au,-2.6988767251,-1.9492515849,0.5803708852\C,-
1.6458083567,-6.8729486629,-2.1417623809\H,-2.5800201627,-7.0796548663
,-2.6824534211\H,-1.1849920735,-7.8491876474,-1.9400530476\H,-1.901111
1465,-6.4237258273,-1.1789286749\C,-0.3079591039,-6.6671481276,-4.2881
445391\H,0.2095161943,-7.6193901308,-4.1076478929\H,-1.1850106179,-6.9
027683615,-4.9065933443\H,0.3561707303,-6.0223889259,-4.8722066122\P,-
5.0246624696,-2.2344498637,0.6579324578\C,-5.8148739923,-2.4388395155,
-0.9899543279\H,-5.4113269505,-3.323323199,-1.4920587192\H,-6.89952186
68,-2.5522860225,-0.8828135053\H,-5.6077295991,-1.5639117517,-1.613643
3665\C,-5.8989005685,-0.8098739645,1.4240744732\H,-6.979357076,-0.9911
458292,1.4457498004\H,-5.5416196116,-0.6588198208,2.447229162\H,-5.701
6578981,0.1013454365,0.8512401739\C,-5.5710434109,-3.6979319649,1.6288
121819\H,-5.2137324194,-3.6163630893,2.6598015807\H,-6.6647781433,-3.7
659108211,1.6340798392\H,-5.1588629894,-4.6122335058,1.1914061595\\Ver
sion=EM64L-G09RevA.01\State=1-A\HF=-1646.2705788\RMSD=8.949e-09\RMSF=2
.987e-06\Dipole=-2.3503234,0.9061175,-0.4217243\Quadrupole=19.3426336,
-4.7561072,-14.5865264,-19.7729956,0.4448985,1.166346\PG=C01 [X(C28H45
Au1O1P1)]\\@

3a-D

1\1\GINC-SHI_02\FOpt\RB3LYP\GenECP\C26H41Au1O1P1(1+)\YIN\12-Jun-2015\0
\\#p opt b3lyp/genecp\\Title Card Required\\1,1\C,-0.0115737958,-0.004
4049858,0.0157787181\C,-0.0079626722,0.0098042473,1.4339000515\C,1.218
4369024,0.0117440232,2.2183511548\C,1.2522985165,-0.8281495513,3.36499
55622\C,2.4034809561,0.7167842276,1.8453480172\C,2.4291780517,-1.07493
0422,4.0570319093\H,0.349937455,-1.353823008,3.6464848315\C,3.62184104
08,0.4876170209,2.5234484492\C,3.5858259285,-0.4157188476,3.5972568766
\H,4.5104615647,-0.6064903137,4.1254668005\O,2.3787019032,1.6395037372
,0.8294655069\C,1.5542109123,2.816775398,1.0197102149\H,0.5078553948,2
.5081184835,1.1425802786\H,1.8703763444,3.3392809876,1.931070643\C,1.6
988860928,3.6854133854,-0.1913635857\C,2.0071517255,4.9842698441,-0.14
52764893\H,1.5095139461,3.196138708,-1.1464172309\C,4.9512720885,1.168
7055688,2.1100220628\C,5.2858025055,0.8256314145,0.635997628\H,6.23380
25053,1.2987692,0.3538896241\H,5.4066102471,-0.2572805076,0.5054382398
\H,4.514489497,1.1804721973,-0.0509067293\C,4.8607700496,2.7050625397,
2.2843457534\H,5.8419248293,3.1539814319,2.0895329211\H,4.1510878181,3
.1556355483,1.5891292066\H,4.5722227541,2.9698293042,3.3089585465\C,6.
1382647052,0.6821920575,2.9708145768\H,6.0243458206,0.9447708083,4.029
2796017\H,6.296231594,-0.4000146243,2.8931399424\H,7.0534322838,1.1673
699433,2.6156671162\C,2.5146918799,-2.0260102629,5.2654450766\C,3.4981

088986,-3.1783491685,4.9447644403\C,3.0251630888,-1.2407437897,6.49916
87977\C,1.1476865534,-2.6429656567,5.6200917403\H,3.1606083576,-3.7564
767057,4.076303644\H,4.5083007532,-2.8111471792,4.7330490749\H,3.56858
88808,-3.8617295116,5.7989725261\H,2.3445734094,-0.4200708641,6.755036
1399\H,3.0928547636,-1.9071453671,7.3670027489\H,4.0207260857,-0.81399
94579,6.3351113515\H,1.2595486015,-3.3057642545,6.4848507122\H,0.40815
4403,-1.8790678236,5.8900294916\H,0.7418855597,-3.2476606622,4.7997122
77\C,-1.3315457602,0.0479437157,2.1456061717\H,-1.6701396431,1.0940777
341,2.1782649577\H,-2.0941611973,-0.5142338215,1.597857599\H,-1.277366
0895,-0.2907309716,3.1811407315\Au,-1.5980974621,0.7892794964,-1.11604
55727\C,0.9724844839,-0.6523100542,-0.6635481019\C,2.0766934036,-1.623
878803,-0.6653929422\C,1.51394778,-1.027508411,-1.9673452447\H,3.05959
53902,-1.2961573375,-0.3319316494\H,1.8524230939,-2.6656184076,-0.4380
828386\H,0.9232572796,-1.6734982719,-2.6163785954\H,2.1175964393,-0.29
2701937,-2.5012204573\P,-3.3296924639,1.6853290612,-2.4440364039\C,-3.
3884428264,1.005966475,-4.1535220387\H,-3.5337583096,-0.0781488112,-4.
1191063001\H,-4.2096513977,1.4583526249,-4.72073578\H,-2.4446280562,1.
210388469,-4.6682921634\C,-5.0213577153,1.3957961413,-1.7782749563\H,-
5.1152435476,1.849423098,-0.786811161\H,-5.7772395282,1.8308161592,-2.
4417904682\H,-5.206071097,0.321256022,-1.6844014476\C,-3.2358852874,3.
5086624122,-2.67966003\H,-2.2892261567,3.7735648433,-3.1604935789\H,-4
.0642081199,3.8605876184,-3.3048516972\H,-3.2810446274,4.0134989361,-1
.7098141368\H,2.0647201396,5.5868760255,-1.0475956535\H,2.2103667821,5
.4954060239,0.7938581707\\Version=EM64L-G09RevA.01\State=1-A\HF=-1567.
5692621\RMSD=9.416e-09\RMSF=4.990e-06\Dipole=-1.9026529,0.3380462,-0.9
930591\Quadrupole=11.3609514,-16.7143341,5.3533827,-9.6220825,29.45389
06,-13.1458201\PG=C01 [X(C26H41Au1O1P1)]\\@

3a-TS1

1\1\GINC-SHI_02\FTS\RB3LYP\GenECP\C26H41Au1O1P1(1+)\YIN\15-Jun-2015\0\
\#p opt=(calcfc,ts,noeigen) b3lyp/genecp\\3k_au_pme3_ts2\\1,1\C,0.6608
453409,-0.1770212827,0.3124906982\C,-0.250717757,-1.0331235063,-0.2605
610031\C,-1.7085550473,-0.7547489281,-0.2056091454\C,-2.6122253044,-1.
8345271018,-0.2451843568\C,-2.2443133904,0.5495181871,-0.0811892274\C,
-3.9899557785,-1.6531239477,-0.1309397793\H,-2.2146990682,-2.83528556,
-0.3433791556\C,-3.6233529585,0.7985157805,0.0616948835\C,-4.451405687
4,-0.3387383164,0.0316855457\H,-5.5162281115,-0.1935219142,0.147782513
9\O,-1.3376837109,1.6425088521,-0.1046369814\C,-0.8553429964,1.9446962
823,-1.4690895048\H,-0.3022286839,1.0673009442,-1.8200847002\H,-1.7385
120582,2.077467942,-2.0969427515\C,0.0143314075,3.1607910466,-1.452618
2248\C,-0.2625697538,4.2741372512,-2.1382734592\H,0.9439360187,3.08423
72915,-0.8915237775\C,-4.2450545307,2.2027608269,0.3051718963\C,-3.652
1774858,2.8350106527,1.5889114368\H,-4.144188693,3.7937397569,1.791197
3383\H,-3.817512014,2.187885315,2.4589881494\H,-2.5819701069,3.0299020
402,1.4873681198\C,-4.0007939246,3.155432621,-0.8902921339\H,-4.587223

7854,4.0715236555,-0.7542021564\H,-2.9546391384,3.4558382262,-0.968420
2592\H,-4.3151030226,2.6988199413,-1.8367202018\C,-5.7753683054,2.1220
459051,0.508594776\H,-6.2958629557,1.7445305734,-0.3790029556\H,-6.051
4092057,1.500353029,1.3677316215\H,-6.1584647118,3.1292393973,0.702953
3182\C,-4.9946849379,-2.8209922442,-0.1629546484\C,-5.7844678793,-2.85
82159703,1.1686954622\C,-5.9796667855,-2.6172154629,-1.3405069078\C,-4
.3013165039,-4.1852817156,-0.3475351321\H,-5.1122760933,-3.0001271304,
2.0232747808\H,-6.3534634884,-1.9373510391,1.3368014466\H,-6.499124432
9,-3.6895506491,1.1586384797\H,-5.4497812706,-2.5959804644,-2.30021395
35\H,-6.7037004872,-3.4397905554,-1.3716014877\H,-6.5448676144,-1.6835
022203,-1.2474048399\H,-5.0569316665,-4.9781045088,-0.3694583043\H,-3.
7444277323,-4.240879159,-1.2907915027\H,-3.6147309512,-4.414374535,0.4
765694546\C,0.1961064089,-2.2729471547,-0.9961575103\H,1.2845864953,-2
.3000314739,-1.0818090321\H,-0.1173545327,-3.1793024223,-0.4615841848\
H,-0.2375202631,-2.3301388343,-2.0019875543\Au,2.7475522692,-0.3107524
673,0.1103416717\C,0.1768708059,0.8942385903,1.0933483051\C,-0.3570838
031,1.0968890597,2.4548659958\C,0.6756037784,2.0547846136,1.8459216266
\H,-1.3560636292,1.4989169329,2.5887727394\H,-0.0621398878,0.355295071
9,3.1942888387\H,1.7185089057,2.0332187521,2.1567141507\H,0.2953183473
,3.0398126761,1.5907584868\P,5.0880802241,-0.4952731015,-0.1089556605\
C,5.7829683206,-2.0528953795,0.5836437742\H,5.338428999,-2.9165379858,
0.0795317206\H,6.8702415068,-2.0830850075,0.4501041372\H,5.5518125245,
-2.1221043366,1.6510029013\C,5.6916379399,-0.460597316,-1.8476582175\H
,5.4067128022,0.4832186386,-2.3226649428\H,6.7823053294,-0.5618517479,
-1.8793786812\H,5.2411647269,-1.2804973778,-2.4155372562\C,6.039021758
1,0.8400227902,0.7290426836\H,7.1158768241,0.6922419185,0.5901391169\H
,5.7580874444,1.8139803865,0.3165084058\H,5.8160341926,0.8413136388,1.
8004468862\H,0.4210041256,5.1186596019,-2.149798584\H,-1.1738653505,4.
3792426278,-2.7233671881\\Version=EM64L-G09RevA.01\State=1-A\HF=-1567.
5603927\RMSD=8.302e-09\RMSF=1.119e-06\Dipole=2.2039128,0.373309,-0.061
3902\Quadrupole=42.1044491,-14.0116201,-28.092829,-2.7359257,-0.240923
,-0.027588\PG=C01 [X(C26H41Au1O1P1)]\\@

3a-I

1\1\GINC-SHI_02\FOpt\RB3LYP\GenECP\C28H45Au1O1P1(1+)\YIN\06-May-2015\0
\\#p opt b3lyp/genecp\\vdcp_ph_tbu_3a_au_int_h\\1,1\C,-0.0154706017,0.
0654463197,-0.040087113\C,0.0258114571,0.0719505467,1.3228592419\C,1.3
321076221,0.0651184761,2.0252748992\C,1.4596282094,-0.3365515486,3.363
9871314\C,2.5138963399,0.431228037,1.3557394067\C,2.7029598083,-0.3804
613817,4.0035562867\H,0.5660399329,-0.621671173,3.9018838172\C,3.80026
32638,0.3849618844,1.9051488721\C,3.8320425256,-0.0277267362,3.2522090
306\H,4.7993502689,-0.075842507,3.7371353405\O,2.2990658965,0.96112176
77,0.018462058\C,1.9062142555,2.4978269901,0.0957370562\H,1.0115290146
,2.4769713351,0.7167892229\H,2.7551157314,2.8926138934,0.6456840002\C,
1.6786612857,3.0933115355,-1.2334119203\C,2.4373363576,4.044932373,-1.

8226079154\H,0.7566888031,2.7930494868,-1.7258346338\C,5.1587728519,0.
7252152067,1.2332418127\C,5.1122048568,0.9573005395,-0.2921654667\H,6.
1344302178,1.1306800637,-0.6463282019\H,4.7267813101,0.08568492,-0.830
1939256\H,4.5204528528,1.8274618461,-0.580812104\C,5.7349945597,2.0011
771512,1.8974510389\H,6.7119002598,2.2432278492,1.4626004047\H,5.07691
53601,2.865905561,1.7462317833\H,5.8700960309,1.8748868192,2.976255634
8\C,6.1431439803,-0.4534110104,1.4596571555\H,6.3566357053,-0.63425954
42,2.5163597974\H,5.7505887115,-1.3830926905,1.0317083492\H,7.09817661
05,-0.2322898271,0.969873869\C,2.8646601304,-0.8026275687,5.4769887195
\C,3.7807776525,-2.0487689224,5.559740895\C,3.5032528812,0.3607429328,
6.2752803776\C,1.5162811282,-1.1538816218,6.1365159348\H,3.3563817754,
-2.8890745536,4.9977507534\H,4.7831551476,-1.8513924498,5.1642174768\H
,3.8942853933,-2.3629937711,6.6038862152\H,2.8738551558,1.2578622628,6
.238329198\H,3.6224675838,0.0748185313,7.3269823207\H,4.4941701729,0.6
286660048,5.8922941534\H,1.6846904875,-1.4408507806,7.1801891706\H,0.8
249493131,-0.3025443734,6.1407239377\H,1.0232217553,-1.9994613507,5.64
2053421\C,-1.2209644009,0.0171606047,2.1762509387\H,-2.1119095211,0.12
78148408,1.5529489006\H,-1.3062610715,-0.9401455757,2.7079596486\H,-1.
2327111009,0.8102682361,2.9351223866\Au,-1.7554371988,-0.102011364,-1.
1683251272\C,1.3055097549,0.1011514236,-0.719421036\C,1.9539504431,-1.
1199809247,-1.3298537234\C,1.577028354,0.0592830924,-2.1928908329\H,3.
0098357575,-1.2910150142,-1.1465851794\H,1.341595411,-2.0160164731,-1.
3477285782\H,0.7050580242,-0.0068802137,-2.8377883155\H,2.3900761914,0
.6667637229,-2.5759787048\C,2.0032347623,4.6411319259,-3.1382658627\H,
2.7892822239,4.5164950892,-3.8951349933\H,1.0825211584,4.1873890737,-3
.516365588\H,1.83810319,5.722087538,-3.0371099719\C,3.7122705945,4.626
7592625,-1.2685958923\H,3.5986967057,5.7098207863,-1.1289098697\H,4.02
88233272,4.1989030322,-0.3152561088\H,4.5309924453,4.4921014201,-1.987
4846245\P,-3.761654212,-0.3227170627,-2.4041461526\C,-5.2282532595,0.4
426104569,-1.5935616995\H,-6.1268659228,0.3077491722,-2.2060785869\H,-
5.3933939411,-0.0173902802,-0.6142587016\H,-5.0553135581,1.5129436317,
-1.444436584\C,-3.7337379844,0.4348734282,-4.0832421635\H,-2.940684947
7,-0.0208361654,-4.6842981358\H,-4.6934901782,0.2896557479,-4.59176194
14\H,-3.5320945761,1.5076751388,-4.0039207989\C,-4.2795920772,-2.06422
83504,-2.7084269602\H,-5.2202546289,-2.0983774608,-3.2695587109\H,-3.5
058249989,-2.5896505792,-3.2767376158\H,-4.4141591732,-2.5831464268,-1
.7542978757\\Version=EM64L-G09RevA.01\State=1-A\HF=-1646.2129194\RMSD=
9.268e-09\RMSF=2.819e-06\Dipole=-0.5840734,0.9468033,-1.6096182\Quadru
pole=20.9205133,-20.1434143,-0.7770989,13.2189853,24.2360278,-6.997764
1\PG=C01 [X(C28H45Au1O1P1)]\\@

3a-TS2

1\1\GINC-A213\FTS\RB3LYP\GenECP\C26H41Au1O1P1(1+)\SIOCWY\17-Jun-2015\0
\\#p b3lyp/genecp opt=(calcfc,ts,noeigen)\\3a_au_pme3_ts6\\1,1\C,0.563
6263361,0.0865177768,0.0292667384\C,-0.3152684787,-0.8460439668,-0.493

7554633\C,-1.7458477307,-0.728273619,-0.2194493459\C,-2.6427504716,-1.
7995778358,-0.3906474153\C,-2.2593845691,0.5305007117,0.1974557877\C,-
4.0072397183,-1.645182197,-0.1598545952\H,-2.2504507243,-2.7591211545,
-0.70025979\C,-3.6370995558,0.7575392738,0.402139285\C,-4.4571516397,-
0.3653680489,0.2262550685\H,-5.5181056432,-0.2418824986,0.411128786\O,
-1.3598428725,1.5613441819,0.2930404157\C,-0.7670387866,2.0220552061,-
2.1666307691\H,-0.925212069,0.9711619133,-2.369600763\H,-1.6323471161,
2.6176992674,-1.9006232028\C,0.4596794236,2.6408842108,-2.4530683976\C
,0.6287256954,3.9967014907,-2.3610747605\H,1.2911047899,2.0160054652,-
2.7714492866\C,-4.3221537984,2.0835641346,0.8285612306\C,-3.3872625843
,3.3000009887,1.0014439373\H,-3.9972610166,4.1652039919,1.2856385785\H
,-2.642556912,3.1478757707,1.78745793\H,-2.8598362929,3.5574260598,0.0
779393196\C,-5.3740340304,2.4693856263,-0.2434925064\H,-5.8841106262,3
.3942079511,0.0504709107\H,-4.900665164,2.6407741871,-1.2181042423\H,-
6.1402736075,1.6993284276,-0.3768598341\C,-5.0338084334,1.8618749387,2
.1886082932\H,-5.7895432585,1.07182085,2.1411420848\H,-4.3139986967,1.
5902731683,2.9697680931\H,-5.5390929565,2.7836146333,2.500184787\C,-5.
0175008154,-2.7978685179,-0.3016366878\C,-5.6910678591,-3.0559016871,1
.0694251461\C,-6.0993672529,-2.4172904625,-1.3429499502\C,-4.349277093
1,-4.1079635663,-0.7627781464\H,-4.9496703381,-3.3385353721,1.82606870
73\H,-6.2278432667,-2.1746499916,1.4371810922\H,-6.4168665671,-3.87327
26596,0.9838742629\H,-5.6522867001,-2.2275902267,-2.3260208519\H,-6.81
95459897,-3.2367612718,-1.4513851753\H,-6.6614717318,-1.5236815376,-1.
0503561602\H,-5.1072097802,-4.8944259269,-0.8470048597\H,-3.8757475565
,-4.0045979191,-1.7466482929\H,-3.5947240894,-4.4592171488,-0.04878377
94\C,0.1634914579,-2.0441942825,-1.2776410561\H,1.2229888045,-1.943582
2671,-1.5268264314\H,0.0467394217,-2.9716122176,-0.6997781245\H,-0.402
805295,-2.1758981735,-2.2084038453\Au,2.6277491025,-0.1628637401,0.048
6545366\C,-0.0619706444,1.199480969,0.7664500559\C,0.1193771719,1.3398
517541,2.2845334642\C,0.675383123,2.3755109292,1.3473536931\H,-0.76879
72137,1.5909290146,2.8565011637\H,0.7949281062,0.6359132747,2.76025491
13\H,1.7488359796,2.4169341796,1.1873504116\H,0.1624137102,3.332057649
3,1.3076775553\P,4.9718334983,-0.5038269579,0.1354573485\C,5.493304953
6,-2.2160485455,-0.296996391\H,6.5818001387,-2.320486293,-0.2268271685
\H,5.0220072493,-2.9310245374,0.3845201229\H,5.1776866434,-2.455736206
8,-1.3171569198\C,5.9639962357,0.5661781736,-0.9891480946\H,5.78807035
36,1.6201279224,-0.7522515836\H,7.033603147,0.3521750184,-0.8846570563
\H,5.6669393739,0.3923021167,-2.0280849437\C,5.7107119174,-0.213394207
2,1.7975089803\H,6.7903534296,-0.4005474025,1.7807033464\H,5.533525763
9,0.8205320546,2.1096975164\H,5.2456804977,-0.8778737678,2.5322763899\
H,1.5662101268,4.4743457884,-2.6297834157\H,-0.1759555055,4.6439101368
,-2.0204076441\\Version=EM64L-G09RevA.01\State=1-A\HF=-1567.5581514\RM
SD=7.096e-09\RMSF=1.653e-06\Dipole=2.3109632,1.0303644,-0.8896993\Quad
rupole=37.2779167,-10.2497715,-27.0281452,-0.8038402,-0.9108673,-4.687

5a-P

1\1\GINC-A113\FOpt\RB3LYP\GenECP\C26H41Au1O1P1(1+)\SIOCWY\14-Jun-2015\
0\\#p opt b3lyp/genecp\\Title Card Required\\1,1\C,0.0290175428,-0.028
9715286,-0.0176530779\C,0.0183574516,-0.0180910099,1.3851939255\C,1.31
39972842,-0.0119114861,2.101845914\C,1.4220512651,-0.3674784673,3.4577
511221\C,2.4466493484,0.4938861734,1.4198736153\C,2.617664727,-0.20563
43596,4.1541009204\H,0.5532221249,-0.7726024743,3.9615056243\C,3.66806
95178,0.7188447385,2.0866632102\C,3.6947872565,0.3492481515,3.44169694
45\H,4.6236735595,0.5033157902,3.9792654971\O,2.2931995684,0.851782781
5,0.1024445497\C,-1.1624877271,0.4095843019,-0.8686482308\H,-1.1051203
83,-0.0830151259,-1.847170122\H,-2.1084621092,0.0930693795,-0.42184829
65\C,-1.16791611,1.9140240105,-1.0561864539\C,-2.194068963,2.707569048
5,-0.7369006932\H,-0.2636872232,2.3471830902,-1.4825988577\C,4.9549476
458,1.3523615424,1.4932444972\C,4.9223880919,1.6451766293,-0.022573087
1\H,5.8888780899,2.076155288,-0.308783172\H,4.7784450876,0.7367218122,
-0.6161074497\H,4.1427014999,2.3600284007,-0.2947981979\C,5.209504605,
2.6993986892,2.2184053379\H,6.1296644252,3.1599161075,1.839382701\H,4.
384822681,3.40088496,2.0457064535\H,5.3224073098,2.5734294187,3.300473
4896\C,6.1545936203,0.4014014143,1.7407922427\H,6.3401919688,0.2163859
805,2.8033921665\H,5.9962549001,-0.5679525921,1.2528787457\H,7.0673124
795,0.8427275411,1.3241729618\C,2.7886189443,-0.5882097249,5.636827229
8\C,3.905667283,-1.6524174093,5.7703797879\C,3.1761981096,0.6674406715
,6.4558065781\C,1.4958049987,-1.1717049762,6.2400581896\H,3.6560317529
,-2.5606472312,5.2082158283\H,4.8694199152,-1.2852864833,5.4013203775\
H,4.039790646,-1.9315116613,6.8222922462\H,2.4005966536,1.4391093836,6
.3861368562\H,3.3008321291,0.4053306762,7.5132440738\H,4.1186844458,1.
1075989452,6.1128648639\H,1.6666049792,-1.4344579028,7.2897515002\H,0.
668454619,-0.4520846079,6.2152022138\H,1.1771865986,-2.085875846,5.723
249418\C,-1.2193407429,0.3437834239,2.184302603\H,-2.1484764859,0.1574
814656,1.6439758714\H,-1.266337612,-0.1893896228,3.1363665033\H,-1.174
481774,1.4163240172,2.4161967127\Au,-0.3545208532,-2.2719542207,0.6567
318173\C,1.4078398258,0.0340715477,-0.6441384487\C,2.0693037616,-1.116
9969072,-1.3826615096\C,1.6613688007,0.136794718,-2.1197889375\H,3.125
7625657,-1.2764188903,-1.1860193037\H,1.4930183346,-2.0225042783,-1.55
80567092\H,0.8234410177,0.0838069913,-2.8077457232\H,2.4451950312,0.82
80257782,-2.4154892954\P,-0.7791152304,-4.5653211166,0.7600119637\C,-2
.0931607352,-5.0055057569,1.9656485621\H,-3.029442825,-4.5065360571,1.
6984082556\H,-2.2541131865,-6.0894452235,1.9704783487\H,-1.8018214231,
-4.6838713622,2.9699807751\C,0.6835246472,-5.5632722636,1.2496502734\H
,1.0334060465,-5.2547444211,2.2392919344\H,0.4218019219,-6.6270033981,
1.2773108862\H,1.4967020432,-5.4140784866,0.532993091\C,-1.3334901526,
-5.2730735713,-0.8424043088\H,-1.522843796,-6.3471082153,-0.7355372067
\H,-2.2522223119,-4.7794858377,-1.1730336621\H,-0.5638751873,-5.122209
298,-1.6053728533\H,-2.1524181706,3.7804063366,-0.9041720033\H,-3.1174
351652,2.3173152205,-0.3123728329\\Version=EM64L-G09RevA.01\State=1-A\

HF=-1567.6309291\RMSD=8.986e-09\RMSF=3.286e-06\Dipole=-1.8613489,-2.86
31227,-0.5690389\Quadrupole=-2.8152648,11.7759003,-8.9606355,15.963685
2,6.6628505,3.543412\PG=C01 [X(C26H41Au1O1P1)]\\@

3a-TS1'

1\1\GINC-SHI_02\FTS\RB3LYP\GenECP\C26H41Au1O1P1(1+)\YIN\18-Jun-2015\0\
\#p opt=(calcfc,ts,noeigen) b3lyp/genecp\\Title Card Required\\1,1\C,1
.4871206757,-0.2421899192,3.1778320403\C,1.2950018656,1.1277686324,2.6
98099936\H,0.6709548939,-0.7332684262,3.6994970738\H,2.4813085771,-0.5
902292915,3.4392565544\H,2.1487479771,1.7961316597,2.6469373549\H,0.34
2649647,1.615349427,2.8859390195\C,1.2908821398,0.0044085688,1.6103891
317\C,0.0421261675,-0.4486484849,1.1981272187\C,-2.1833024912,-0.95698
80144,0.4264104586\C,-3.2650887643,-1.8134555191,0.215056819\C,-2.3036
593974,0.4222920452,0.2005607521\C,-4.4850382127,-1.290336212,-0.22857
18656\H,-3.1483758713,-2.8745043131,0.3982462568\C,-3.534446017,1.0217
805526,-0.1153446302\C,-4.578492546,0.1042007535,-0.3593085295\H,-5.53
74132006,0.5141246428,-0.6501983288\O,-1.078086168,1.0881330178,0.3930
599386\C,-0.4219124885,1.5005810869,-0.874863631\H,0.0510842867,0.6013
356771,-1.2861182419\H,-1.2062364264,1.8268734027,-1.5580425801\C,0.57
08256796,2.5916912176,-0.6223935567\C,0.5890365565,3.7334920311,-1.316
7219764\H,1.3297408579,2.3958865664,0.13006947\C,-3.85059504,2.5392693
565,-0.1740242034\C,-2.8687568258,3.3916179329,0.6605722779\H,-3.22749
79175,4.4268140045,0.6844854073\H,-2.8148097129,3.0351377304,1.6958710
722\H,-1.8563531519,3.4119002036,0.258190552\C,-3.8571721806,3.0294526
488,-1.6433175571\H,-4.1368439531,4.0890380091,-1.683532268\H,-2.87650
08669,2.9301039955,-2.1222819689\H,-4.5793725154,2.4675984818,-2.24642
65317\C,-5.2602033401,2.8044497359,0.4203209238\H,-6.0692314941,2.3909
611467,-0.1889132162\H,-5.3507615744,2.3966053434,1.4333964692\H,-5.42
80192406,3.8855231277,0.475497396\C,-5.7086386812,-2.1754153295,-0.534
5757249\C,-6.8824396452,-1.7827236546,0.396133616\C,-6.1306881683,-1.9
698360365,-2.0103587065\C,-5.4099779339,-3.673213666,-0.3252228482\H,-
6.6103144052,-1.9084553035,1.4507612019\H,-7.193616263,-0.7425230073,0
.2511499965\H,-7.7525650323,-2.4180192606,0.1928295501\H,-5.3213617739
,-2.2499011236,-2.6952299856\H,-7.0023818387,-2.5926829783,-2.24347818
35\H,-6.4029762145,-0.9294116172,-2.2189473558\H,-6.3014185585,-4.2614
040636,-0.5686619549\H,-4.59958033,-4.0257014613,-0.9748734276\H,-5.14
59231619,-3.8998954695,0.7148700925\C,-0.8846312702,-1.3644593688,0.96
72605266\C,-0.5924325952,-2.8086875135,1.3297909617\H,-0.6821133405,-3
.4415036483,0.4388540147\H,0.4145500811,-2.9286824389,1.7347394519\H,-
1.3123266653,-3.1669120175,2.0747508631\Au,2.9372159071,-0.2578634369,
0.3143374245\P,4.8058411281,-0.5728025085,-1.0861307654\C,4.939566714,
-2.2601823329,-1.8101091598\H,4.0569988021,-2.4763825504,-2.4200979897
\H,5.8347741097,-2.3419387933,-2.436780655\H,4.9954662014,-3.004995688
6,-1.0102252872\C,6.4180174597,-0.3137287612,-0.2363160262\H,7.2506025
344,-0.4751556309,-0.9302270297\H,6.4740154408,0.7061803536,0.15642528

92\H,6.5130101187,-1.0105977589,0.6021026283\C,4.8673330972,0.55433395
25,-2.5398341689\H,5.7675984688,0.3684756408,-3.1362793447\H,3.9849685
892,0.3991624757,-3.1682569035\H,4.8702339526,1.5956586378,-2.20369020
81\H,1.3553552534,4.4855657961,-1.1499994922\H,-0.1539409081,3.9593607
461,-2.0791174366\\Version=EM64L-G09RevA.01\State=1-A\HF=-1567.5574885
\RMSD=9.610e-09\RMSF=1.072e-06\Dipole=2.2070719,-0.338939,-0.5966016\Q
uadrupole=37.2833957,-15.0436587,-22.239737,-2.2715968,-6.547408,-2.80
73035\PG=C01 [X(C26H41Au1O1P1)]\\@

3a-F

1\1\GINC-SHI_02\FOpt\RB3LYP\GenECP\C26H41Au1O1P1(1+)\YIN\19-Jun-2015\0
\\#p opt b3lyp/genecp\\Title Card Required\\1,1\C,-0.0007992503,-0.063
7955761,-0.2967351862\C,0.0809206631,0.051418356,1.1910124459\H,0.9352
393213,-0.1131038808,-0.8488027797\H,-0.8013105925,0.4471992327,-0.822
8292545\H,-0.6599941096,0.6497725294,1.7129079047\H,1.074559252,0.0940
19356,1.6332694662\C,-0.3834825487,-1.277606842,0.5774481356\C,0.59498
468,-2.3525238017,0.5639859012\C,2.1616488216,-4.0262456202,0.32397073
84\C,3.0125630444,-4.996865411,-0.2055904801\C,2.3134957777,-3.6213985
815,1.649751427\C,4.00965626,-5.5419625552,0.6140097544\H,2.898920869,
-5.3034189682,-1.2384328712\C,3.3648429008,-4.0006504737,2.4874800224\
C,4.1610145295,-5.0154999142,1.911356235\H,4.9817600253,-5.3920487309,
2.5073967305\O,1.2638488508,-2.6542683121,1.9164987764\C,0.2090674418,
-3.0775659206,2.9686842122\H,0.7746576655,-3.137854082,3.8925967021\H,
-0.4465498596,-2.2075186948,2.9681658298\C,-0.4755073701,-4.3454008072
,2.6030309164\C,-0.4043899745,-5.447630708,3.3575494444\H,-1.098827062
7,-4.3220341889,1.7120296356\C,3.7654722048,-3.3880293468,3.8540940217
\C,3.3664703063,-1.8961681785,3.9549794335\H,3.7546688387,-1.480678538
3,4.8910747222\H,3.7986497272,-1.3177056919,3.1302646343\H,2.288425229
8,-1.7223212682,3.9542950853\C,3.1536647775,-4.1947782618,5.0251625218
\H,3.5075322931,-3.79209989,5.9813891746\H,2.0594894571,-4.158418988,5
.0421169016\H,3.4492459124,-5.2485057504,4.9724642304\C,5.3075478611,-
3.4288794517,4.0222543341\H,5.6943345552,-4.4443528416,4.1500770152\H,
5.819375959,-2.9686281594,3.1701174135\H,5.5838613594,-2.8707763796,4.
9232286664\C,4.9682328019,-6.646533339,0.1271612404\C,6.4240120454,-6.
1182794849,0.149052418\C,4.8507974544,-7.8750498217,1.0627449365\C,4.6
451182805,-7.1044257938,-1.3089715652\H,6.5365250674,-5.2442289227,-0.
5031420707\H,6.7441341357,-5.830408387,1.1562986001\H,7.1109567691,-6.
8955241883,-0.2057870833\H,3.8292711248,-8.273613109,1.0654914704\H,5.
5233483988,-8.6713030953,0.7230385084\H,5.1212638948,-7.6336131685,2.0
965328585\H,5.3372345624,-7.9009568612,-1.602671783\H,3.6283770308,-7.
5068142472,-1.3935976426\H,4.7589948591,-6.291994834,-2.0366448527\C,1
.1299826048,-3.2186608439,-0.3183234617\C,0.7731694829,-3.2995786518,-
1.7721133251\H,0.4128112191,-4.304970517,-2.0249793296\H,-0.0091003852
,-2.5821624097,-2.0296468998\H,1.6475101212,-3.0992398815,-2.403911909
5\Au,-2.4358919349,-1.7783768965,0.617574062\P,-4.7571938957,-2.222663

891,0.5899369519\C,-5.7927584821,-0.7028658119,0.4966972291\H,-5.54850
41526,-0.1417587409,-0.4106137357\H,-6.8581027283,-0.9591576485,0.4820
971141\H,-5.5918355291,-0.0613435463,1.3602962619\C,-5.4048266622,-3.1
153250531,2.0658794114\H,-6.4865483128,-3.2692239536,1.9820566672\H,-4
.9132143422,-4.0886269791,2.1594271491\H,-5.1970705443,-2.5368222964,2
.9713849589\C,-5.3256470285,-3.241697888,-0.8354085649\H,-6.4105639617
,-3.3918210861,-0.7990058437\H,-5.0665545973,-2.7430919878,-1.77458096
68\H,-4.8303958355,-4.2175544615,-0.8178964886\H,0.1990656042,-5.49558
74279,4.260785148\H,-0.9646197292,-6.3427646732,3.1018396749\\Version=
EM64L-G09RevA.01\State=1-A\HF=-1567.5677683\RMSD=8.993e-09\RMSF=4.258e
-06\Dipole=-1.5770297,-0.5634482,0.5503674\Quadrupole=34.5836246,-19.5
284074,-15.0552172,-15.9165243,8.5619611,-0.541679\PG=C01 [X(C26H

3a-TS2’

1\1\GINC-SHI_02\SP\RB3LYP\GenECP\C26H41Au1O1P1(1+)\YIN\23-Jun-2015\0\\
#p b3lyp/genecp\\Title Card Required\\1,1\C,0,-1.396663,0.773647,-2.95
5501\C,0,-1.250554,2.019328,-2.128845\H,0,-0.564158,0.498617,-3.600857
\H,0,-2.371398,0.51855,-3.360923\H,0,-2.128182,2.638645,-1.96633\H,0,-
0.328967,2.588252,-2.230253\C,0,-1.185075,0.668411,-1.431742\C,0,0.138
709,0.215255,-1.003754\C,0,2.123924,-0.815841,-0.531511\C,0,3.267576,-
1.635154,-0.495381\C,0,2.22671,0.576545,-0.268057\C,0,4.472625,-1.0753
98,-0.075844\H,0,3.191883,-2.679264,-0.776582\C,0,3.411993,1.192005,0.
164774\C,0,4.496281,0.308289,0.260434\H,0,5.444191,0.723173,0.583382\O
,0,1.031219,1.163876,-0.531181\C,0,0.117488,0.534248,2.751878\H,0,1.08
0803,0.933069,3.063012\H,0,-0.768979,1.065161,3.088617\C,0,0.017817,-0
.587674,1.991515\C,0,1.161608,-1.322768,1.593429\H,0,-0.960567,-0.9404
31,1.67591\C,0,3.609851,2.689593,0.475564\C,0,2.317012,3.521792,0.3505
71\H,0,2.539081,4.561085,0.617853\H,0,1.917821,3.517764,-0.667629\H,0,
1.52872,3.16603,1.021738\C,0,4.138463,2.847547,1.922923\H,0,4.30288,3.
908228,2.144633\H,0,3.416714,2.462277,2.654515\H,0,5.088063,2.326338,2
.082225\C,0,4.649768,3.272841,-0.514009\H,0,5.623937,2.780455,-0.42706
\H,0,4.309326,3.166843,-1.550434\H,0,4.797106,4.340581,-0.313782\C,0,5
.776502,-1.889937,0.019018\C,0,6.843698,-1.26577,-0.915222\C,0,6.29536
2,-1.868301,1.478553\C,0,5.577291,-3.360163,-0.398141\H,0,6.504914,-1.
266817,-1.957743\H,0,7.082829,-0.233165,-0.639861\H,0,7.772785,-1.8449
76,-0.86033\H,0,5.566425,-2.31759,2.164232\H,0,7.22584,-2.442992,1.554
111\H,0,6.506743,-0.852219,1.828837\H,0,6.529547,-3.894931,-0.315436\H
,0,4.856677,-3.878321,0.246408\H,0,5.241653,-3.450779,-1.437892\C,0,0.
74178,-1.027785,-0.999707\C,0,0.173643,-2.303707,-1.526896\H,0,0.39455
3,-3.158302,-0.873002\H,0,-0.910878,-2.232105,-1.645868\H,0,0.605751,-
2.545625,-2.508554\Au,0,-2.869251,0.064334,-0.315624\P,0,-4.891184,-0.
524986,0.768115\C,0,-6.107414,-1.341229,-0.348814\H,0,-5.685966,-2.270
891,-0.743595\H,0,-7.035817,-1.568489,0.187128\H,0,-6.33543,-0.683762,
-1.193451\C,0,-5.814352,0.912774,1.456712\H,0,-6.75731,0.591008,1.9129
18\H,0,-5.206992,1.420772,2.21224\H,0,-6.031567,1.628215,0.657487\C,0,

-4.752427,-1.690713,2.190406\H,0,-5.739784,-1.907534,2.613767\H,0,-4.297534,-2.629631,1.859098\H,0,-4.121399,-1.256961,2.972618\H,0,2.140072,-1.041829,1.969258\H,0,1.055737,-2.344085,1.247517\\Version=EM64L-G09RevA.01\State=1-A\HF=-1567.5594131\RMSD=2.199e-09\Dipole=-1.1776851,-1.1652232,1.9751512\Quadrupole=39.9986597,-18.9068777,-21.091782,2.9562765,-5.5690298,1.509359\PG=C01 [X(C26H41Au1O1P1)]\\@

4a-P

1\1\GINC-A155\FOpt\RB3LYP\GenECP\C26H41Au1O1P1(1+)\SIOCWY\14-Jun-2015\0\\#p opt b3lyp/genecp\\Title Card Required\\1,1\C,0.0087868003,0.0290295111,0.0090642922\C,0.0102264208,0.0260540088,1.5291715624\H,0.9739762001,0.0357940037,-0.490555036\H,-0.7560215653,0.6093216346,-0.4992028309\H,-0.7488467753,0.611831009,2.0396029105\H,0.9728740367,0.0085848419,2.0363011151\C,-0.4050527269,-1.2076269747,0.7697675904\C,0.2400468843,-2.4577670944,0.8128850827\C,1.1481822358,-4.5895365684,0.4838327859\C,1.6713001799,-5.820965602,0.0945137278\C,1.0715292916,-4.2804006083,1.8311606737\C,2.0948252728,-6.7241731462,1.0833272831\H,1.7434549953,-6.0687436543,-0.9563483483\C,1.4686693175,-5.1109072692,2.8803853574\C,1.9762922508,-6.3441909309,2.4371711301\H,2.305250013,-7.049574368,3.1895490512\O,0.5483880521,-2.9725404065,1.9929574621\C,-0.5558193639,-3.7032118358,-1.2652177884\H,-1.3409047696,-4.190980805,-0.6760154191\H,-0.9522363268,-2.7326989453,-1.5950766496\C,-0.239172961,-4.540584949,-2.4760069711\C,-0.7780419377,-5.7386243818,-2.7239697762\H,0.4396416553,-4.105604486,-3.2092811375\C,1.3668016207,-4.7130784313,4.36318295\C,2.2317005609,-3.4542756531,4.6240699806\H,2.1795124472,-3.1799096377,5.6843785119\H,3.2829505779,-3.6434591904,4.3773535989\H,1.8912130468,-2.5939850013,4.0401443679\C,-0.1111969259,-4.4167738071,4.7222271139\H,-0.1889947259,-4.1464915241,5.7821280368\H,-0.5169272321,-3.5866439112,4.1356316772\H,-0.7394686023,-5.2997221366,4.5532769503\C,1.8665657291,-5.8356531866,5.2939676847\H,1.2800159177,-6.7551698511,5.1842026809\H,2.9225816184,-6.0740535176,5.122861265\H,1.77090584,-5.5087635256,6.3351014322\C,2.6854652266,-8.1054971392,0.7364721183\C,4.1232282726,-8.2094592955,1.3035781523\C,1.8052744433,-9.2159742613,1.3619594134\C,2.7528624599,-8.3475336476,-0.7844172738\H,4.7726604354,-7.4358043258,0.8771596939\H,4.1456317275,-8.1039323438,2.3935614174\H,4.5547700591,-9.1868933049,1.0576929142\H,0.7799408021,-9.1718647285,0.9755056503\H,2.215958871,-10.2032740061,1.1195968675\H,1.7568529694,-9.137653076,2.4535165099\H,3.1784780257,-9.3382497901,-0.977806012\H,1.7606198944,-8.3218101503,-1.2507129183\H,3.3925153585,-7.6142688917,-1.2903472986\C,0.6557422497,-3.397903262,-0.3203575554\C,1.8235243522,-2.7421598867,-1.1121454494\H,2.2782061803,-3.4916156475,-1.765549437\H,1.4661797985,-1.9126714248,-1.7295824893\H,2.6062456038,-2.375178021,-0.4401543754\Au,-2.5102389491,-1.7173064257,0.8494955076\P,-4.8265658264,-2.0516415241,0.9900391071\C,-5.6530408313,-2.2435619876,-0.6412662339\H,-5.2377295123,-3.1054315162,-1.1723941954\H,-6.7308850506,-2.3906

950613,-0.5094872099\H,-5.4874036864,-1.3502858862,-1.2511844619\C,-5.
7135315957,-0.6643428727,1.8081974173\H,-6.788383662,-0.8726283456,1.8
524230097\H,-5.3343615688,-0.5261136133,2.825270188\H,-5.5526636134,0.
2631184541,1.2502846217\C,-5.3095587123,-3.5475028186,1.9446512001\H,-
4.9208195017,-3.4803450404,2.9651847396\H,-6.4008589447,-3.6395260942,
1.9829020981\H,-4.8924755598,-4.4429258092,1.473999716\H,-0.5517477151
,-6.2868409065,-3.6346115156\H,-1.4686342963,-6.2127264148,-2.02857175
36\\Version=EM64L-G09RevA.01\State=1-A\HF=-1567.6333533\RMSD=1.877e-09
\RMSF=4.203e-06\Dipole=-2.2836297,0.8997821,-0.5336991\Quadrupole=20.7
583226,-5.7704114,-14.9879112,-18.6157243,-0.3963166,-1.0983182\PG=C01

Transformation of product 6

The compound **6** could be transformed to the alcoholic product **S7** via hydroboration/oxidation, which can be further converted into hydroxyl masked product **S8** through Mitsunobu reaction.

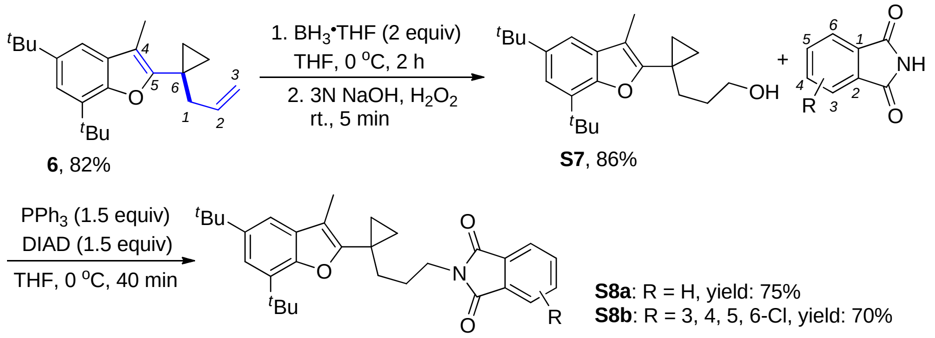

Procedures for the synthesis of compound S7 with modified reaction conditions:

To a flame dried, argon purged 50 mL flask was added a solution of **6** in THF (0.2 M, 5mL), set the flask in ice-water bath, then BH_3·THF (1.0 M, 1.2 mL) was added into the flask dropwise via syringe. The reaction mixture was stirred at room temperature for 2 hours. After that, 5 mL 3N NaOH was added and the resulting mixture was stirred at room temperature for 5 minutes, then 5 mL 30% H_2O_2 was added, and the reaction mixture was stirred for another 5 minutes. After all, the organic phase was dried over anhydrous Na_2SO_4 and concentrated under reduced pressure and the residue was purified by a silica gel flash column chromatography with petroleum ether-EtOAc (10:1) as an eluent.

Procedures for the synthesis of compound S8 with modified reaction conditions:

To a flame dried, argon purged Schlenk tube was added PPh_3 (39 mg, 0.15 mmol), solution of **S7** in THF (0.05 M, 2.0 mL), set the flask in ice-water bath, then DIAD (30 mg, 0.15 mmol) was added into the reaction tube dropwise. The reaction mixture was stirred at 0 °C for 30 minutes. After that, 0.5 mL water was added to quench the reaction. The organic phase was dried over anhydrous Na_2SO_4 and was concentrated under reduced pressure and the residue was purified by a silica gel flash column chromatography with petroleum ether-EtOAc (20:1) as an eluent.

References:

1. (a) Salaun, J.; Marguerite, J. *Org. Synth.* **1985**, 147-151. (b) Cambeiro1, X. C.; Pericàs, M. A. *Adv. Synth. Catal.* **2011**, *353*, 113-124.
2. (a) Hirano, K.; Biju, A.; Piel, T. I.; Glorius, F. *J. Am. Chem. Soc.* **2009**, *131,* 14190-14191. (b) McQuaid, K. M.; Long, J. Z.; Sames, D. *Org. Lett.* **2009**, *11*, 2972-2975. (c) Rohlmann, R.; Daniliuc, C.-G.; Mancheño, O. G. *Chem. Commun.* **2013**, *49*, 11665-11667. (d) Lovely, C. J.; Seshadri, H.; Wayland, B. R.; Cordes, A. W. *Org. Lett.* **2001**, *3*, 2607-2610. (e) Wijtmans, M.; Maussang, D.; Sirci, F.; Scholten, D. J.; Canals, M.; Delic, A. M.; Chong, M.; Chatalic, K. L. S.; Custers, H.; Janssen, E.; Graaf,; Smit, C. M. J.; Esch, I. J. P.; Leurs, R. *Eur. J. Med. Chem.* **2012**, *51*, 184-192. (f) Fu, J.; Shang, H.; Wang, Z.; Chang, L.; Shao, W.; Yang, Z.; Tang, Y. *Angew. Chem. Int. Ed.* **2013**, *52*, 4198-4202.
3. Hashmi, A. S. K.; Braun, I.; Nösel, P.; Schädlich, J.; Wieteck, M.; Rudolph, M.; Rominger, F. *Angew. Chem. Int. Ed.* **2012**, *51*, 4456-4460.
4. a) Yuan, W.; Dong, X.; Shi, M.; McDowell, P. G.; Li, G. *Org. Lett.* **2012**, *14*, 5582-5585; b) Yuan, W.; Dong, X.; Wei, Y.; Shi, M. *Chem. Eur. J.* **2012**, *18*, 10501-10505.
5. Frisch, M. J. et al. *Gaussian 09*, revision A.01; Gaussian, Inc.: Wallingford, CT, **2009**.
